# Identification of Smoking-Associated Transcriptome Aberration in Blood with Machine Learning Methods

**DOI:** 10.1155/2023/5333361

**Published:** 2023-01-04

**Authors:** FeiMing Huang, QingLan Ma, JingXin Ren, JiaRui Li, Fen Wang, Tao Huang, Yu-Dong Cai

**Affiliations:** ^1^School of Life Sciences, Shanghai University, Shanghai 200444, China; ^2^Advanced Research Computing, University of British Columbia, Vancouver, Canada; ^3^Guangdong AIB Polytechnic College, Guangzhou 510507, China; ^4^Bio-Med Big Data Center, CAS Key Laboratory of Computational Biology, Shanghai Institute of Nutrition and Health, University of Chinese Academy of Sciences, Chinese Academy of Sciences, Shanghai 200031, China; ^5^CAS Key Laboratory of Tissue Microenvironment and Tumor, Shanghai Institute of Nutrition and Health, University of Chinese Academy of Sciences, Chinese Academy of Sciences, Shanghai 200031, China

## Abstract

Long-term cigarette smoking causes various human diseases, including respiratory disease, cancer, and gastrointestinal (GI) disorders. Alterations in gene expression and variable splicing processes induced by smoking are associated with the development of diseases. This study applied advanced machine learning methods to identify the isoforms with important roles in distinguishing smokers from former smokers based on the expression profile of isoforms from current and former smokers collected in one previous study. These isoforms were deemed as features, which were first analyzed by the Boruta to select features highly correlated with the target variables. Then, the selected features were evaluated by four feature ranking algorithms, resulting in four feature lists. The incremental feature selection method was applied to each list for obtaining the optimal feature subsets and building high-performance classification models. Furthermore, a series of classification rules were accessed by decision tree with the highest performance. Eventually, the rationality of the mined isoforms (features) and classification rules was verified by reviewing previous research. Features such as isoforms ENST00000464835 (expressed by LRRN3), ENST00000622663 (expressed by SASH1), and ENST00000284311 (expressed by GPR15), and pathways (cytotoxicity mediated by natural killer cell and cytokine–cytokine receptor interaction) revealed by the enrichment analysis, were highly relevant to smoking response, suggesting the robustness of our analysis pipeline.

## 1. Introduction

Tabaco smoking is among the leading causes of premature mortality in the world, and this condition can be avoided [1]. It has been demonstrated to be associated with human diseases such as respiratory disease, cardiovascular, cancer, and gastrointestinal (GI) disorders [2–5]. According to the World Health Organization (WHO), smoking causes over US$500 billion economic loss globally annually.

Although cigarette smoke is deemed as the main risk factor for chronic obstructive pulmonary disease, which increases oxidative stress in the airway epithelium, the pathogenesis of most smoking-induced diseases has not been fully determined [5]. A recent article systematically reviewed previous studies on smoking-associated DNA methylation and the alteration of gene expression in human blood samples, in which 1,758 genes with differentially methylated sites and differentially expressed genes between smokers and nonsmokers were reported [6]. Therefore, gene expression alterations are important for smoking response. Considering that alternative splicing is applied by up to 95% of human genes for producing proteins with different functions, a recent study has focused on identifying smoking-associated isoforms and found that 3′ UTR lengthening was widely associated with cigarette smoking [7, 8]. A total of 945 differentially expressed isoforms were identified in this study by using the classic statistic method. Machine learning could be applied without preexisting knowledge to analyze RNA-seq data and deal with a large number of variables in a much smaller sample size [9].

In the present study, we applied the Boruta [10] and four feature ranking algorithms to identify the isoforms with important roles in distinguishing smokers from former smokers based on the expression data of 85,437 isoforms on current and former smokers collected in the previous study [8]. The incremental feature selection (IFS) method [11] was employed to further analyze the results yielded by above methods for extracting optimal features, building efficient classification models, and interesting classification rules. The literature review and further comparison of these features identified by four feature ranking methods demonstrated strong biological relevance of these features (isoforms) with smoking response.

## 2. Materials and Methods

### 2.1. Data

The RNA-seq data in whole-blood samples on 454 current and 767 former smokers were accessed from the Gene Expression Omnibus (GEO) database under the accession number GSE171730 [8]. We separated the samples into two classes based on their smoking history: current smokers and former smokers. Each sample contains 85,437 transcript features. Such data was deeply analyzed by investigating a binary classification problem containing two classes (current smokers and former smokers) and 85,437 features. The purpose of this study was to discover essential transcript features that can classify smokers and reveal different patterns for current and former smokers.

### 2.2. Boruta Feature Filtering

As lots of features were involved for each smoker sample and only a few of them have strong associations with smoke, the irrelevant features should be screened out first and excluded. Here, we selected the Boruta method [10, 12, 13] to complete this task.

The Boruta is a feature selection method using random forest (RF), which can be used to confirm whether original features are statistically more important than the random features in the prediction. Given a dataset, the Boruta first generates a random feature for each original one. Its values are produced by shuffling numbers under the original feature. RF is performed on such extended dataset to evaluate the importance of all original and random features. Original features with importance remarkably better than the highest importance on random features are labelled as confirmed, while those that perform worse are categorized as rejected. Confirmed features are excluded from the dataset, and the updated dataset is put into the next round. After a number of rounds, features in the rejection region are dropped, and confirmed features in each round are kept. Unlike wrapper approaches, which try to locate some powerfully relevant features, the Boruta chooses features that are strongly or weakly important to achieve the best classification accuracy.

The Boruta program downloaded from https://github.com/scikit-learn-contrib/boruta_py was used in the present study for analyzing the RNA-seq data. Default settings were adopted.

### 2.3. Feature Ranking Algorithms

Relevant features were selected by the Boruta method. However, their contributions for prediction were not the same. To clearly classify features with their importance, four feature ranking algorithms were employed, including max-relevance and min-redundancy (mRMR) [14], Monte Carlo feature selection (MCFS) [15], light gradient boosting machine (LightGBM) [16], and least absolute shrinkage and selection operator (LASSO) [17]. As each algorithm has its own defects and merits, the bias may be produced by only using one feature ranking algorithm. Each algorithm can give a part of contributions for discovering essential features. A full and systemic evaluation on features can be obtained by the usage of different algorithms. A brief description on these algorithms was as below.

#### 2.3.1. Max-Relevance and Min-Redundancy

The mRMR algorithm aims at determining the feature subset that has the highest correlation with the target variable and the lowest correlation between the features in this set [14, 18–21]. mRMR uses mutual information to quantify feature-target and feature-feature correlations. However, it is difficult to obtain such feature subset. mRMR adopts a heuristic way to generate a feature list, which is constructed by repeatedly choosing one feature with trade-off on maximum correlation to the target variable and minimum redundancy to features that have been chosen. Such list was termed as mRMR feature list. We utilized the mRMR tool retrieved from Hanchuan Peng's web (http://home.penglab.com/proj/mRMR/in this work), which was run under default parameters.

#### 2.3.2. Monte Carlo Feature Selection

MCFS is a method for ranking features by randomly selecting features to build multiple decision trees [15]. It is commonly used to process biological data [22–24]. In the present research, *m* transcript features are chosen at random to build *t* classification trees for *s* times. Each tree is trained and tested using randomly selected training and test data from the entire dataset. As a result, *s* × *t* classification trees are built. The relative significance (RI) of a given feature *g* is assessed based on how many trees select this feature and how much it contributes to prediction:

(1) $$\begin{array}{c} {\text{RI}}_{g}=\overset{\text{st}}{\underset{\tau =1}{\sum }}{\left(\text{wAcc}\right)}^{u}\underset{n_{g}\left(\tau \right)}{\sum }\text{I}\text{G}\left(n_{g}\left(\tau \right)\right){\left(\frac{\text{no}.\text{in }n_{g}\left(\tau \right)}{\text{no}.\text{in }\tau }\right)}^{v}, \end{array}$$

where wAcc is the weighted accuracy, IG(*n*_*g*_(*τ*)) represents the information gain (IG) of node *n*_*g*_(*τ*), (no.in *n*_*g*_(*τ*)) denotes the number of samples in node *n*_*g*_(*τ*), and (no.in *τ*) stands for the number of samples in the tree root. *u* and *v* are two parameters. In this study, we used the MCFS program obtained from http://www.ipipan.eu/staff/m.draminski/mcfs.html, which was performed with default parameters. The list generated by MCFS was called the MCFS feature list.

#### 2.3.3. Light Gradient Boosting Machine

LightGBM algorithm is an ensemble method using gradient boosting framework. It improves the gradient boosting decision tree and has the advantages, such as high efficiency, support for parallelism, low GPU memory consumption, and large-scale data processing [16]. LightGBM can estimate the feature importance based on the times appearing in the ensemble trees, and the high frequency indicates the importance of the feature. The study adopted the program of LightGBM (https://lightgbm.readthedocs.io/en/latest/) in Python, and we ran it with default parameters. For an easy description, the list produced by LightGBM was described as the LightGBM feature list.

#### 2.3.4. Least Absolute Shrinkage and Selection Operator

In 1996, Robert Tibshirani proposed a new feature selection technique called the minimum absolute compression method or LASSO [17]. It employs the L1 paradigm to develop a penalty function, which can selectively eliminate features by assigning a higher penalty on features with higher coefficients and greater prediction errors, leading to a model with fewer features and that effectively reduces overfitting. Clearly, features with high coefficients do not contribute favorably to the prediction and should be scaled down. Consequently, features can be ranked according to their coefficients. In this study, the LASSO package collected in the scikit-learn was adopted, which was performed using its default parameters. Likewise, the list derived by LASSO was called the LASSO feature list.

### 2.4. Incremental Feature Selection

The feature ranking algorithms only sorted features in lists. It was still a problem for the selection of essential features. Therefore, the IFS method was employed to determine essential features from each list based on given classification algorithms [11], which can be further used to build the efficient classification models [25–28]. From each feature list, the IFS method first divides the feature list into *n* feature subsets whose feature numbers differ by 1 in turn. Subsequently, various feature subsets were then used to construct the models using a single classification algorithm, and the effectiveness of classification was assessed using tenfold cross-validation [29]. The optimal model can then be identified based on its performance. In addition, features employed in best model were referred to as the optimal features.

### 2.5. Synthetic Minority Oversampling Technique

In the investigated data, former smokers were 1.7 times as many as current smokers. It was not a balanced dataset, which may produce bias in a model that is built directly based on it. In view of this, we employed the powerful oversampling algorithm, synthetic minority oversampling technique (SMOTE) [30, 31], to tackle this problem. To equalize the distribution of data among various classes, this method synthesizes new samples of a minority class. It selects one sample from the minority class as a seed sample and then randomly chooses one of its *k* closest neighbors. The following is the synthesis equation:

(2) $$\begin{array}{c} \;s=x+\beta \left(x-y\right), \end{array}$$

where *x* stands for the coordinates of the seed sample in the Euclidean space, *y* represents the coordinates of a randomly selected *k*-nearest neighbor of *x*, and *β* is an arbitrary number between 0 and 1.

Here, the SMOTE program reported at https://github.com/scikitlearn-contrib/imbalanced-learn was used. The default parameters were applied to run the program.

### 2.6. Classification Algorithm

The IFS technique required the use of at least one classification algorithm to construct the model on each feature subset. Four classification algorithms were used in this instance: the decision tree (DT), the random forest (RF), the *k*-nearest neighbor (KNN), and the support vector machine (SVM) [32–35]. These classification algorithms are theoretically sound and are widely used in machine learning.

#### 2.6.1. *k*-Nearest Neighbor

KNN is a classic classification algorithm. For a test sample, *k* training samples that are nearest to the test sample are chosen based on a distance metric, and the class of the test sample is established based on the classes of these *k* training samples.

#### 2.6.2. Random Forest

RF constructs many DTs to form a forest by using bootstrap aggregation. Besides the sample selection, features are also randomly selected when constructing each DT. When classifying a sample, each tree makes a prediction, and the class with the highest votes is considered the final decision of RF.

#### 2.6.3. Support Vector Machine

SVM is a powerful classification algorithm. According to the distributions of training samples, it finds the optimal hyperplane for classifying samples in different classes. In many instances, it is difficult to build such a hyperplane in the original feature space. In order to map samples into a new space with a higher dimension, where the hyperplane is simple to construct, the kernel approach is used. We can establish its class based on which side of the hyperplane it is on.

#### 2.6.4. Decision Tree

DT is a relatively simple classification algorithm that is utilized as a predictive model in medical diagnostics and biomarker screening [36]. Different from above three algorithms, the classification principle is much easier to be understood, which is the greatest merit of DT. By learning training samples, a tree is built. Such tree gives a completely open procedure for classifying test samples. This provides opportunities to figure out its classification principle. Thus, it is deemed as a white-box algorithm. A set of rules can also be used to represent DT in addition to its tree-like structure. Each rule shows a route from the root to a single leaf. For various classes, these rules can suggest multiple patterns.

In this study, the above algorithms are implemented by using the scikit-learn package written in Python [37].

### 2.7. Performance Evaluation

F1-measure is the primary metric utilized in this study to assess how well classification models perform [38–40]. In fact, it is computed by combining the values of precision and recall. It indicates that the model is good when the F1-measure is high. These measurements can be calculated as follows:

(3) $$\begin{array}{c} \text{F}1-\text{measure}=\frac{2\times \text{recall}\times \text{precision}}{\text{ recall}+\text{precision}},\\ \text{Precision}=\frac{\text{TP}}{\text{TP}+\text{FP}},\\ \text{Recall}=\frac{\text{TP}}{\text{TP}+\text{FN}}, \end{array}$$

where true positive (TP) is the number of positive samples that are correctly labelled as positive samples, false positive (FP) is the number of negative samples that are incorrectly labelled as positive samples, and false negative (FN) indicates the number of positive samples that are mistakenly labelled as negative samples.

Besides, the following measurements are also widely used for binary classification, including sensitivity (SN), specificity (SP), accuracy (ACC), and the Matthews correlation coefficient (MCC), where SN is same as recall, and others can be computed by

(4) $$\begin{array}{c} \text{Specificity}=\frac{\text{TN}}{\text{TN}+\text{FP}},\\ \text{ACC}=\frac{\text{TP}+\text{TN}}{\text{TP}+\text{FP}+\text{TN}+\text{FN}},\\ \text{MCC}=\frac{\text{TP}\times \text{TN}-\text{FP}\times \text{FN}}{\sqrt{\left(\text{TP}+\text{FP}\right)\left(\text{TP}+\text{FN}\right)\left(\text{TN}+\text{FP}\right)\left(\text{TN}+\text{FN}\right)}}, \end{array}$$

where TP, FP, and FN are same as those in the above paragraph and TN indicates the number of negative samples that are correctly labelled as negative samples. They were also provided in this study to display a more complete evaluation on different models.

### 2.8. Functional Enrichment Analysis

Through the above machine learning algorithms, some essential features (transcripts) can be screened out. These transcripts were converted to the corresponding genes via “bitr” from the clusterProfiler package in R [41]. Then, the enrichment analysis of gene ontology (GO) terms and Kyoto Encyclopedia of Genes and Genomes (KEGG) pathways was performed on these genes. The FDR, used to adjust the *p* value, was picked up as the key indicator for selecting enriched GO terms and KEGG pathways. Its cutoff was set to 0.05.

## 3. Results

**Figure 1:**
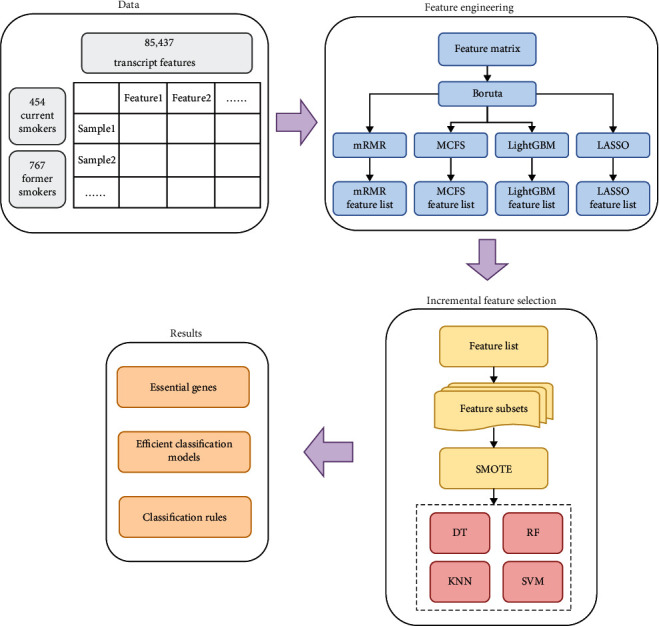
Process flow diagram for the full analysis. After being first filtered by the Boruta, the 85,437 transcript features from the 1221 smokers were then sorted by feature importance using four feature ranking algorithms: mRMR, MCFS, LightGBM, and LASSO. The IFS computational framework was then performed based on four sorted feature sets, and four effective classification algorithms were used in this process. Eventually, the essential genes (converted from important features) and the classification rules were extracted.

In the current study, we developed a computational pipeline, which combined some feature analysis methods with the classification models, to investigate the RNA-seq data of two kinds of smokers.  Figure 1 shows the entire procedures. The detailed results were displayed in this section.

### 3.1. Results of Feature Selection Methods

The 85,437 original transcript features were first filtered using the Boruta. 370 features were screened out, which were deemed to be highly correlated with the target variables. Subsequently, the 370 transcript features were ranked using four feature ranking algorithms, resulting in four feature lists (mRMR, MCFS, LightGBM, and LASSO feature lists), which are shown in Table S1. In Discussion, we performed a biological analysis of some top-ranked features yielded by four ranking algorithms.

### 3.2. Results of IFS Method and Feature Intersection

**Figure 2:**
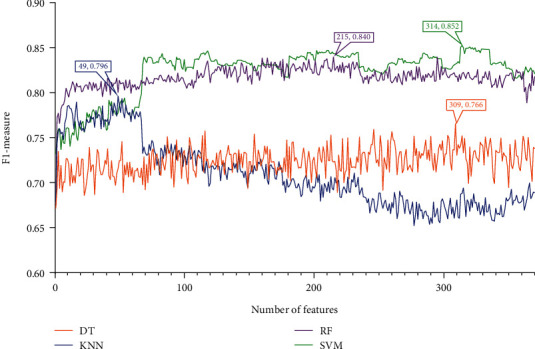
IFS curves for showing the performance of four classification algorithms according to F1-measure on the mRMR feature list.

**Figure 3:**
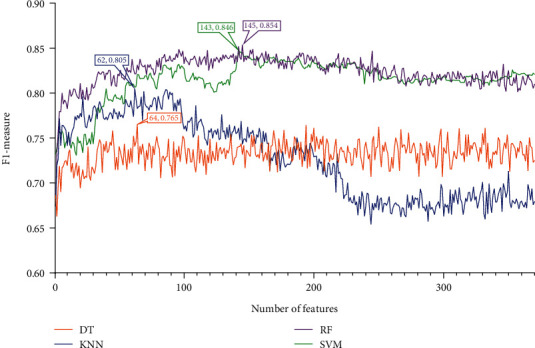
IFS curves for showing the performance of four classification algorithms according to F1-measure on the MCFS feature list.

**Figure 4:**
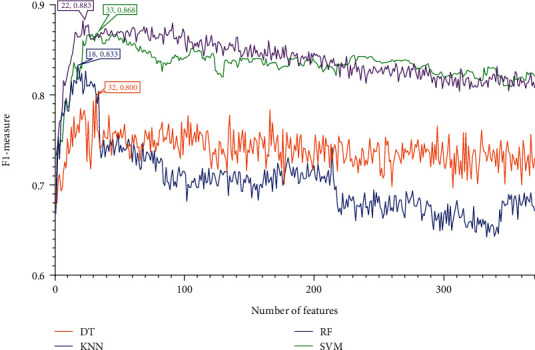
IFS curves for showing the performance of four classification algorithms according to F1-measure on the LightGBM feature list.

**Figure 5:**
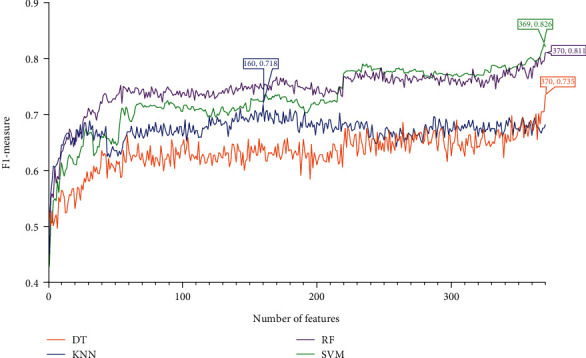
IFS curves for showing the performance of four classification algorithms according to F1-measure on the LASSO feature list.

Four feature lists were obtained by using four feature ranking algorithms. Each of them was fed into the IFS computational framework and processed in the same manner. First, 370 feature subsets were generated, each containing a few of the most prominent features from the original feature list. One of the four classification algorithms listed in Classification Algorithm was used to build a model for each feature subset, and it was then tested using tenfold cross-validation. The model's performance was mainly measured by F1-measure. To display how the performance changed with different feature subsets, an IFS curve was created. All these curves under various feature lists and classification algorithms are shown in Figures 2–5, and the detailed performance is listed in Table S2.

**Figure 6:**
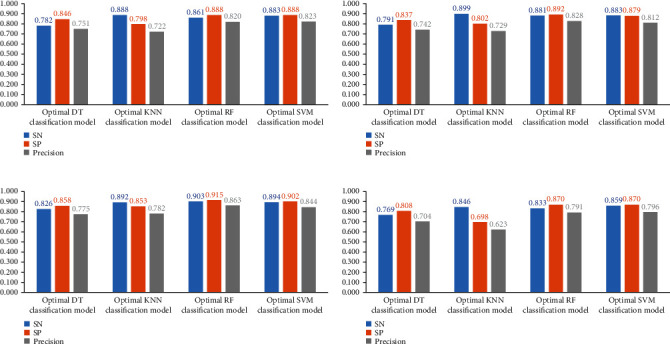
Performance of the optimal models based on different feature lists. (a) mRMR feature list, (b) MCFS feature list, (c) LightGBM feature list, and (d) LASSO feature list.

For the IFS results on the mRMR feature list, Figure 2 shows the performance of four classification algorithms under various feature subsets. It can be shown that when the top 309, 49, 215, and 341 features in the mRMR feature list were adopted, DT, KNN, RF, and SVM produced the greatest F1-measure values of 0.766, 0.796, 0.840, and 0.852. These features made up the best feature set for the corresponding classification algorithm and can be used to create the best classification models.  Table 1 lists the specific overall performance of these models, including ACC, MCC, and F1-measure, while Figure 6(a) illustrates other measurements (SN, SP, and precision). Clearly, the optimal SVM model provided the best performance among all optimal models. Thus, we set its optimal features (top 314 features in the mRMR feature list) as the optimal features extracted from the mRMR feature list.

For the IFS results on the MCFS feature list, the IFS curves are illustrated in Figure 3. With the same argument, the RF model using top 145 features yielded the best performance according to Figures 3 and 6 and Table 1. These 145 features constituted the optimal features extracted from the MCFS feature list.

Referring to the IFS results on the rest feature lists (LightGBM and LASSO feature lists), we can conclude that RF model with top 22 features in the LightGBM feature list and SVM model with top 369 features in the LASSO feature list provided the highest performance, refer to Figures 4–6 and Table 1. These features made up the optimal feature sets derived from the above two feature lists.

**Figure 7:**
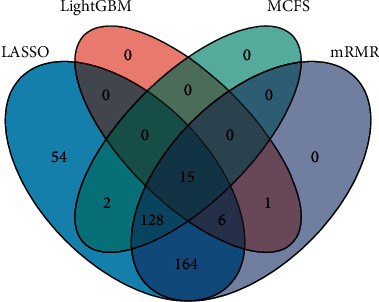
Venn diagram of the optimal feature subsets obtained from the mRMR, MCFS, LightGBM, and LASSO feature lists. The circles indicate transcripts that were identified as optimal features by different ranking algorithms.

With the above analysis, four optimal feature subsets were obtained from four feature lists. The Venn diagram was plotted to show the relationships between them, as illustrated in Figure 7. Detailed intersection results of four optimal feature subsets are shown in Table S3. We can see that 15 transcript features occurred in all four optimal feature subsets, which were deemed to be highly associated with smoking and would be analyzed biologically in the subsequent discussion section.

### 3.3. Classification Rules

**Figure 8:**
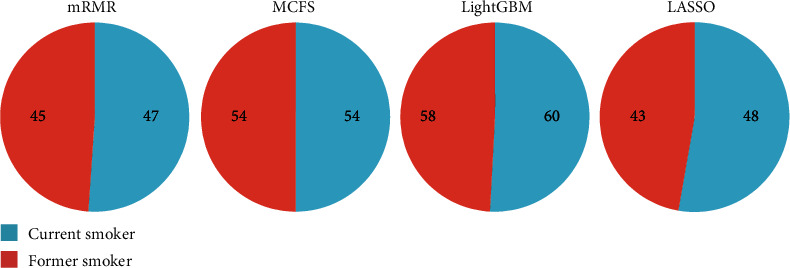
Number of rules utilized to distinguish each class in each classification rule set extracted based on the mRMR, MCFS, LightGBM, and LASSO feature lists. A large number of classification rules were built to describe two kinds of smokers. The number of classification rules for each kind of smokers was approximately the same, indicating that our method was unbiased.

According to the results listed in the above section, DT generally yielded the lowest performance. However, it can offer more insights than other three classification algorithms as it is a white-box algorithm. Thus, DT was picked up again in this section. The IFS findings show that the numbers of optimal features for DT on mRMR, MCFS, LightGBM, and LASSO feature lists were 309, 64, 32, and 370, respectively. We used these features to represent all smoker samples, and DT was applied to such data for generating four trees. From these trees, four sets of classification rules were accessed, as shown in Table S4. The numbers of rules used to distinguish two types of samples in each set of rules are shown in Figure 8. The detailed analysis of the rules that can distinguish the two classes with the largest number of samples would be provided in Discussion.

### 3.4. Results of Enrichment Analysis

**Figure 9:**
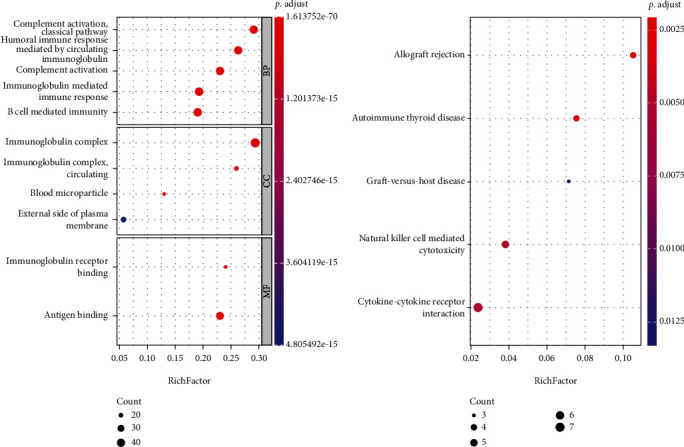
Analysis of KEGG pathway and gene ontology enrichment using the union of four best feature subsets determined by four feature ranking algorithms. The GO terms and KEGG pathways were filtered by the criterion of FDR < 0.05. The top 5 significant GO enrichment results and KEGG pathway enrichment results are shown.

Four optimal feature subsets extracted from four feature lists were merged into one set, resulting in 370 features. The corresponding genes to the transcripts in the merged set were picked up for enrichment analysis. The significant enrichment results after FDR estimation are shown in Table S5. Furthermore, the top 5 entries of each of the three parts of GO enrichment results and the top 5 pathways of KEGG enrichment results were selected for visual presentation, as shown in Figure 9. GO enrichment results show that immunoglobulin complex, complement activation, and humoral immune response mediated by circulating were significantly enriched by the identified genes. The cytotoxicity caused by natural killer cells, cytokine-cytokine receptor interaction, and viral protein-cytokine interaction was all considerably enriched according to the KEGG enrichment. Further analysis indicated the association of these results with different smoking populations in Discussion.

## 4. Discussion

### 4.1. Functional Enrichment Analyses

To illustrate the biological significance of the features and rules identified in this study, we clustered the 370 features with GO terms and KEGG pathway, respectively. As expected, all the top-ranked GO terms are involved in immune response such as complement activation, humoral immune response, and immunoglobulin complex. Cigarette smoke exposure can affect immune response significantly and may cause multiple diseases [42]. Similarly, most of the enriched KEGG pathways also represented immune responses, such as natural killer cell-mediated cytotoxicity, interaction of cytokine-cytokine receptor, and interaction between viral protein and cytokine receptor. These pathways have been reported to be related to cigarette smoke in previous studies. For instance, cigarette smoke inhibits NK cell ability to kill tumor cell lines and increases the amount of proinflammatory cytokines while downregulates the anti-inflammatory cytokines [43, 44]. Smoke was also found to increase the viral load and cell death, which leads to more severe viral myocarditis [45]. Interestingly, the features were also enriched in pathways of diseases, such as graft-versus-host disease, autoimmune thyroid disease, and type I diabetes mellitus. Smoking was found to be able to both increase and decrease the risks of autoimmune thyroid diseases [46]. Moreover, the animal models treated with nicotine were found to alter the expression of pancreatic cytokine, which leads to a lower incidence of type I [47].

### 4.2. Analysis of Highly Ranked Transcripts in Four Algorithms

As shown above, these 370 features were ranked by four algorithms, and each of which produced a set of optimal features. We compared the four sets and investigated their biological significance related to smoking. Interestingly, the optimal features by LASSO included 369 features, and the mRMR produced an optimal set of 314 features, indicating that these two methods tend to capture features comprehensively. By contrast, the optimal feature sets have 22 and 145 features by LightGBM and MCFS, respectively, and the optimal models provided higher F1-measure (Table 1) than the optimal models on the optimal feature sets of LASSO and mRMR, indicating that these two feature ranking algorithms can capture the features with higher effects.

Meanwhile, 15 features were shared by all four optimal feature sets, suggesting the significance of these features in responding to smoking. Based on the review of literature, we found that 12 features were proved to be relevant to smoking. Features ENST00000464835 and ENST00000308478 are two transcripts of gene *LRRN3*, which are most significantly associated with smoking [48]. ENST00000622663 and ENST00000367467 were two transcripts of the gene *SASH1*, whose expression changes were associated with smoking in human monocytes [49]. Two GWAS studies found that gene *GPR15* is strongly associated with smoking, and its expressed transcript ENST00000284311 is among the optimal features by all four methods in this study [50, 51]. Other features including ENST00000392054 (expressed by PID1), ENST00000586582 (expressed by SEMA6B), ENST00000393590 (expressed by P2RY6), ENST00000422622 (expressed by SSPN), ENST00000339223 (expressed by FPR3), ENST00000341184 (expressed by MGAT3), and ENST00000316418 (expressed by AHRR) are supported by previous studies [52–58]. Therefore, the features identified using all four methods have strong associations with smoking.

In comparison with LASSO or mRMR, the two other algorithms LightGBM and MCFS produced relative smaller number of optimal features. However, these two methods shared only half or less optimal features with each other. A further investigation unveiled that those optimal features by MCFS but not LightGBM were either transcripts of immunoglobulins or unclear in the relevance to smoking. This result indicated the bias or capability of MCFS methods in capturing the features involved in immune response. By contrast, those optimal features identified by LightGBM but not MCFS were involved in diverse functions or bioprocesses. For example, ENST00000297785 was a transcript expressed by the gene *ALDH1*, which was upregulated by smoking, while another feature ENST00000423064, a transcript of *HGF*, was found to be upregulated in male smokers [59]. And many risk factors including smoking were found to be associated with serum *HGF* [60]. Therefore, different algorithms identify features with different focus, and a combination of multiple algorithms is recommended for a comprehensive analysis to complement with each other.

### 4.3. Analysis of Classification Rules

Further analysis on the rules was also conducted to investigate the relevance to smoking. The top-ranked rule in each of the four algorithms received much more passed courts than the second, indicating that the top rule outperformed all the others in terms of precision. Therefore, we focused on the gene expression pattern in the top rule of each algorithm. Among the 38 features in the four top rules, only two features were shared by all four top rules from four algorithms, ENST00000284311 and ENST00000586582, which are the transcripts of *GPR15* and *SEMA6B*. Both features were strongly associated with smoking responses by previous studies [50–52]. The only feature shared by the three top rules was ENST00000390539, which is a transcript of immunoglobulin. Immunoglobulin has four other features, indicating that these immunoglobulin isoforms might play a more important role in smoking response than others. Other than the immune response, the epigenetic change is also a key player in smoking response. Our study revealed four features from these 38 top-rank rules, namely, ENST00000395002 from FAM13A, ENST00000394718 from AKAP5, ENST00000341184 from MGAT3, and ENST00000316418 from AHRR. These genes were associated with smoking response [57, 61–63]. Moreover, 17 features have not been studied, indicating their roles in smoking response. These genes or features could be good candidates and further investigated in future studies to unveil the mechanisms of smoking response.

## 5. Conclusions

In this study, some widely used machine learning methods were applied on transcript expression data to reveal the essential features of different populations with different smoking history. Three aspects of the results were obtained. First, a list of features that could be used to determine the difference between current and former smokers were extracted. These features provided a more detailed description of the alteration of biological processes in the human body by smoking at the transcript level. Second, efficient classification models were built to identify current and former smokers. Finally, specific classification rules for distinguishing current smokers from former smokers were built. These rules quantitatively described the role of transcript expression in differentiating smoking populations, thus providing a theoretical basis for the treatment of smoking-related diseases.

## Figures and Tables

**Table 1:** Performance of the optimal models based on various classification algorithms and lists yielded by various feature ranking algorithms.

Feature ranking algorithms	Classification algorithms	Number of features	F1-measure	MCC	ACC
mRMR	DT	309	0.766	0.623	0.822
	KNN	49	0.796	0.665	0.831
	SVM	314	0.852	0.761	0.886
	RF	215	0.840	0.742	0.878

MCFS	DT	64	0.765	0.620	0.820
	KNN	62	0.805	0.679	0.838
	SVM	143	0.846	0.750	0.880
	RF	145	0.854	0.764	0.888

LightGBM	DT	32	0.800	0.676	0.846
	KNN	18	0.833	0.728	0.867
	SVM	33	0.868	0.788	0.899
	RF	22	0.883	0.811	0.911

LASSO	DT	370	0.735	0.568	0.794
	KNN	160	0.718	0.525	0.753
	SVM	369	0.826	0.718	0.866
	RF	370	0.811	0.695	0.856

## Data Availability

The original data used to support the findings of this study are available at the GEO database (https://www.ncbi.nlm.nih.gov/geo/query/acc.cgi?acc=GSE171730).

## Supplementary Materials

Supplementary Materials Table S1: feature ranking results obtained by mRMR, MCFS, LightGBM, and LASSO methods. Table S2: IFS results on different feature lists. Table S3: intersection of the optimal feature subsets extracted from mRMR, MCFS, LightGBM, and LASSO feature lists. The features that appear in 4, 3, 2, and 1 optimal feature subsets are shown. Table S4: classification rules generated by the optimal DT model. Table S5: GO and KEGG enrichment results after merging the optimal feature subsets of the four feature ranking algorithms.

## References

[1] Samet J. M. (2013). Tobacco smoking: the leading cause of preventable disease worldwide. *Thoracic Surgery Clinics*.

[2] Chatziioannou A., The EnviroGenomarkers project consortium, Georgiadis P. (2017). Blood-based omic profiling supports female susceptibility to tobacco smoke- induced cardiovascular diseases. *Scientific Reports*.

[3] Forey B. A., Thornton A. J., Lee P. N. (2011). Systematic review with meta-analysis of the epidemiological evidence relating smoking to COPD, chronic bronchitis and emphysema. *BMC Pulmonary Medicine*.

[4] Lee K. W., Pausova Z. (2013). Cigarette smoking and DNA methylation. *Frontiers in Genetics*.

[5] Li L. F., Chan R. L. Y., Lu L. (2014). Cigarette smoking and gastrointestinal diseases: the causal relationship and underlying molecular mechanisms (review). *International Journal of Molecular Medicine*.

[6] Silva C. P., Kamens H. M. (2021). Cigarette smoke-induced alterations in blood: a review of research on DNA methylation and gene expression. *Experimental and Clinical Psychopharmacology*.

[7] Jiang W., Chen L. (2021). Alternative splicing: human disease and quantitative analysis from high- throughput sequencing. *Computational and Structural Biotechnology Journal*.

[8] Xu Z., Platig J., Lee S. (2021). Cigarette smoking-associated isoform switching and 3′ UTR lengthening via alternative polyadenylation. *Genomics*.

[9] Bzdok D., Altman N., Krzywinski M. (2018). Statistics versus machine learning. *Nature Methods*.

[10] Kursa M. B., Rudnicki W. R. (2010). Feature selection with theBorutapackage. *Journal of Statistical Software*.

[11] Liu H. A., Setiono R. (1998). Incremental feature selection. *Applied Intelligence*.

[12] Chen L., Li Z., Zeng T. (2021). Predicting gene phenotype by multi-label multi-class model based on essential functional features. *Molecular Genetics and Genomics*.

[13] Zhang Y.-H., Li H., Zeng T. (2020). Identifying transcriptomic signatures and rules for SARS-CoV-2 infection. *Frontiers in Cell and Development Biology*.

[14] Peng H., Long F., Ding C. (2005). Feature selection based on mutual information: criteria of max-dependency, max-relevance, and min-redundancy. *IEEE Transactions on Pattern Analysis and Machine Intelligence*.

[15] Draminski M., Rada-Iglesias A., Enroth S., Wadelius C., Koronacki J., Komorowski J. (2008). Monte Carlo feature selection for supervised classification. *Bioinformatics*.

[16] Ke G., Meng Q., Finley T. (2017). LightGBM: a highly efficient gradient boosting decision tree. *Advances in Neural Information Processing Systems30 (NIP 2017)*.

[17] Tibshirani R. (1996). Regression shrinkage and selection via the lasso: a retrospective. *Journal of the Royal Statistical Society: Series B (Methodological)*.

[18] Yuan F., Lu L., Zhang Y. H., Wang S. P., Cai Y. D. (2018). Data mining of the cancer-related lncRNAs GO terms and KEGG pathways by using mRMR method. *Mathematical Biosciences*.

[19] Yu X., Pan X. Y., Zhang S. Q. (2020). Identification of gene signatures and expression patterns during epithelial-to-mesenchymal transition from single-cell expression atlas. *Frontiers in Genetics*.

[20] Zhao X., Chen L., Lu J. (2018). A similarity-based method for prediction of drug side effects with heterogeneous information. *Mathematical Biosciences*.

[21] Chen L., Li Z. D., Zhang S. Q., Zhang Y.-H., Huang T., Cai Y.-D. (2022). Predicting RNA 5-methylcytosine sites by using essential sequence features and distributions. *BioMed Research International*.

[22] Chen L., Li J. R., Zhang Y. H. (2018). Identification of gene expression signatures across different types of neural stem cells with the Monte-Carlo feature selection method. *Journal of Cellular Biochemistry*.

[23] Chen X., Jin Y., Feng Y. (2019). Evaluation of plasma extracellular vesicle microRNA signatures for lung adenocarcinoma and granuloma with Monte-Carlo feature selection method. *Frontiers in Genetics*.

[24] Li J., Lu L., Zhang Y. H. (2020). Identification of leukemia stem cell expression signatures through Monte Carlo feature selection strategy and support vector machine. *Cancer Gene Therapy*.

[25] Zhang Y. H., Guo W., Zeng T. (2021). Identification of microbiota biomarkers with orthologous gene annotation for type 2 diabetes. *Frontiers in Microbiology*.

[26] Zhang Y. H., Li Z., Zeng T. (2020). Distinguishing glioblastoma subtypes by methylation signatures. *Frontiers in Genetics*.

[27] Ding S., Wang D., Zhou X. (2022). Predicting heart cell types by using transcriptome profiles and a machine learning method. *Life*.

[28] Zhou X., Ding S., Wang D. (2022). Identification of cell markers and their expression patterns in skin based on single-cell RNA-sequencing profiles. *Life*.

[29] Kohavi R. (1995). A study of cross-validation and bootstrap for accuracy estimation and model selection. *International Joint Conference on Artificial Intelligence*.

[30] Chawla N. V., Bowyer K. W., Hall L. O., Kegelmeyer W. P. (2002). SMOTE: synthetic minority over-sampling technique. *Journal of Artificial Intelligence Research*.

[31] Pan X., Chen L., Liu M., Niu Z., Huang T., Cai Y. D. (2022). Identifying protein subcellular locations with embeddings-based node2loc. *IEEE/ACM Transactions on Computational Biology and Bioinformatics*.

[32] Breiman L. (2001). Random forests. *Machine Learning*.

[33] Cortes C., Vapnik V. (1995). Support-vector networks. *Machine Learning*.

[34] Cover T., Hart P. (1967). Nearest neighbor pattern classification. *IEEE Transactions on Information Theory*.

[35] Safavian S. R., Landgrebe D. (1991). A survey of decision tree classifier methodology. *IEEE Transactions on Systems, Man, and Cybernetics*.

[36] Chen L., Li Z., Zeng T. (2021). Identifying COVID-19-specific transcriptomic biomarkers with machine learning methods. *BioMed Research International*.

[37] Pedregosa F., Varoquaux G., Gramfort A. (2011). scikit-learn: machine learning in python. *Journal of Machine Learning Research*.

[38] Powers D. (2011). Evaluation: from precision, recall and f-measure to ROC., informedness, markedness & correlation. *Journal of Machine Learning Technologies*.

[39] Yang Y., Chen L. (2022). Identification of drug–disease associations by using multiple drug anddisease networks. *Current Bioinformatics*.

[40] Ran B., Chen L., Li M., Han Y., Dai Q. (2022). Drug-drug interactions prediction using fingerprint only. *Computational and Mathematical Methods in Medicine*.

[41] Wu T., Hu E., Xu S. (2021). clusterProfiler 4.0: a universal enrichment tool for interpreting omics data. *The Innovations*.

[42] Lugg S. T., Scott A., Parekh D., Naidu B., Thickett D. R. (2022). Cigarette smoke exposure and alveolar macrophages: mechanisms for lung disease. *Thorax*.

[43] O'Shea D., Cawood T. J., O'Farrelly C., Lynch L. (2010). Natural killer cells in obesity: impaired function and increased susceptibility to the effects of cigarette smoke. *PLoS One*.

[44] Arnson Y., Shoenfeld Y., Amital H. (2010). Effects of tobacco smoke on immunity, inflammation and autoimmunity. *Journal of Autoimmunity*.

[45] Bae S., Ke Q., Koh Y. Y. (2010). Exacerbation of acute viral myocarditis by tobacco smoke is associated with increased viral load and cardiac apoptosis. *Canadian Journal of Physiology and Pharmacology*.

[46] Wiersinga W. M. (2016). Clinical relevance of environmental factors in the pathogenesis of autoimmune thyroid disease. *Endocrinology and Metabolism*.

[47] Mabley J. G., Pacher P., Southan G. J., Salzman A. L., Szabó C. (2002). Nicotine reduces the incidence of type I diabetes in mice. *The Journal of Pharmacology and Experimental Therapeutics*.

[48] Beineke P., Fitch K., Tao H. (2012). A whole blood gene expression-based signature for smoking status. *BMC Medical Genomics*.

[49] Lorenz D. R., Misra V., Gabuzda D. (2019). Transcriptomic analysis of monocytes from HIV-positive men on antiretroviral therapy reveals effects of tobacco smoking on interferon and stress response systems associated with depressive symptoms. *Human Genomics*.

[50] Andersen A. M., Lei M. K., Beach S. R. H., Philibert R. A. (2021). Inflammatory biomarker relationships with helper T cell GPR15 expression and cannabis and tobacco smoking. *Journal of Psychosomatic Research*.

[51] Vink J. M., Jansen R., Brooks A. (2017). Differential gene expression patterns between smokers and non-smokers: cause or consequence?. *Addiction Biology*.

[52] Cuppen B. V. J., Rossato M., Fritsch-Stork R. D. E. (2018). RNA sequencing to predict response to TNF-*α* inhibitors reveals possible mechanism for nonresponse in smokers. *Expert Review of Clinical Immunology*.

[53] Martin F., Talikka M., Hoeng J., Peitsch M. C. (2015). Identification of gene expression signature for cigarette smoke exposure response—from man to mouse. *Human & Experimental Toxicology*.

[54] Reynolds L. M., Lohman K., Pittman G. S. (2017). Tobacco exposure-related alterations in DNA methylation and gene expression in human monocytes: the multi-ethnic study of atherosclerosis (MESA). *Epigenetics*.

[55] Graff M., Fernández-Rhodes L., Liu S. (2013). Generalization of adiposity genetic loci to US Hispanic women. *Nutrition & Diabetes*.

[56] Pouwels S. D., Wiersma V. R., Fokkema I. E. (2021). Acute cigarette smoke-induced eQTL affects formyl peptide receptor expression and lung function. *Respirology*.

[57] Barcelona V., Huang Y., Brown K. (2019). Novel DNA methylation sites associated with cigarette smoking among African Americans. *Epigenetics*.

[58] Ohmomo H., Harada S., Komaki S. (2022). DNA methylation abnormalities and altered whole transcriptome profiles after switching from combustible tobacco smoking to heated tobacco products. *Cancer Epidemiology, Biomarkers & Prevention*.

[59] Patel M., Lu L., Zander D. S., Sreerama L., Coco D., Moreb J. S. (2008). ALDH1A1 and ALDH3A1 expression in lung cancers: correlation with histologic type and potential precursors. *Lung Cancer*.

[60] Torres L., Klingberg E., Nurkkala M., Carlsten H., Forsblad-d’Elia H. (2019). Hepatocyte growth factor is a potential biomarker for osteoproliferation and osteoporosis in ankylosing spondylitis. *Osteoporosis International*.

[61] Chen Q., de Vries M., Nwozor K. O. (2021). A protective role of FAM13A in human airway epithelial cells upon exposure to cigarette smoke extract. *Frontiers in Physiology*.

[62] Oldenburger A., Poppinga W. J., Kos F. (2014). A-kinase anchoring proteins contribute to loss of E-cadherin and bronchial epithelial barrier by cigarette smoke. *American Journal of Physiology. Cell Physiology*.

[63] Takeuchi F., Takano K., Yamamoto M. (2022). Clinical implication of smoking-related aryl-hydrocarbon receptor repressor (AHRR) hypomethylation in Japanese adults. *Circulation Journal*.

