# Supplementary material for: Identification of Smoking-Associated Transcriptome Aberration in Blood with Machine Learning Methods
Source: Biomed Res Int. 2023 Jan 4;2023:5333361. doi: 10.1155/2023/5333361 (PMC9833906; doi:10.1155/2023/5333361)
Supplement: Supplementary Materials — Table S1: feature ranking results obtained by mRMR, MCFS, LightGBM, and LASSO methods. Table S2: IFS results on different feature lists. Table S3: intersection of the optimal feature subsets extracted from mRMR, MCFS, LightGBM, and LASSO feature lists. The features that appear in 4, 3, 2, and 1 optimal feature subsets are shown. Table S4: classification rules generated by the optimal DT model. Table S5: GO and KEGG enrichment results after merging the optimal feature subsets of the four feature ranking algorithms. [file 5333361.f1.zip › Table S1 (1).pdf]

**Table S1:** Feature ranking results obtained by mRMR, MCFS, LightGBM, and LASSO methods.

| Rank | mRMR | MCFS | LightGBM | LASSO |
|---|---|---|---|---|
| 1 | ENST00000284311 | ENST00000284311 | ENST00000284311 | ENST00000633685 |
| 2 | ENST00000313401 | ENST00000308478 | ENST00000308478 | ENST00000528808 |
| 3 | ENST00000308478 | ENST00000316418 | ENST00000316418 | ENST00000395002 |
| 4 | ENST00000390548 | ENST00000586582 | ENST00000367467 | ENST00000297785 |
| 5 | ENST00000558197 | ENST00000422987 | ENST00000586582 | ENST00000324907 |
| 6 | ENST00000451085 | ENST00000390539 | ENST00000339223 | ENST00000476232 |
| 7 | ENST00000276974 | ENST00000464835 | ENST00000359228 | ENST00000631690 |
| 8 | ENST00000422987 | ENST00000451085 | ENST00000633685 | ENST00000442677 |
| 9 | ENST00000324907 | ENST00000487272 | ENST00000622663 | ENST00000424873 |
| 10 | ENST00000584793 | ENST00000367467 | ENST00000392054 | ENST00000272224 |
| 11 | ENST00000339223 | ENST00000244174 | ENST00000598234 | ENST00000593178 |
| 12 | ENST00000367051 | ENST00000390548 | ENST00000620457 | ENST00000430223 |
| 13 | ENST00000610495 | ENST00000550772 | ENST00000297785 | ENST00000262158 |
| 14 | ENST00000497872 | ENST00000339223 | ENST00000393590 | ENST00000623011 |
| 15 | ENST00000414455 | ENST00000390305 | ENST00000464591 | ENST00000615525 |
| 16 | ENST00000307428 | ENST00000390321 | ENST00000509152 | ENST00000324079 |
| 17 | ENST00000616417 | ENST00000390308 | ENST00000441556 | ENST00000648973 |
| 18 | ENST00000390325 | ENST00000617175 | ENST00000430223 | ENST00000377712 |
| 19 | ENST00000296029 | ENST00000633446 | ENST00000400072 | ENST00000312143 |
| 20 | ENST00000392055 | ENST00000341184 | ENST00000617716 | ENST00000244745 |
| 21 | ENST00000411764 | ENST00000523272 | ENST00000341184 | ENST00000490251 |
| 22 | ENST00000519554 | ENST00000367434 | ENST00000648322 | ENST00000390270 |
| 23 | ENST00000367256 | ENST00000390323 | ENST00000529814 | ENST00000620457 |
| 24 | ENST00000414273 | ENST00000492167 | ENST00000414455 | ENST00000473185 |
| 25 | ENST00000392054 | ENST00000441556 | ENST00000393203 | ENST00000440480 |
| 26 | ENST00000390285 | ENST00000390252 | ENST00000321016 | ENST00000311597 |
| 27 | ENST00000574371 | ENST00000473726 | ENST00000426706 | ENST00000482769 |
| 28 | ENST00000265022 | ENST00000390606 | ENST00000422622 | ENST00000478742 |
| 29 | ENST00000281821 | ENST00000390549 | ENST00000611977 | ENST00000594028 |
| 30 | ENST00000491977 | ENST00000390547 | ENST00000464835 | ENST00000611977 |
| 31 | ENST00000641136 | ENST00000610349 | ENST00000619589 | ENST00000618026 |
| 32 | ENST00000367245 | ENST00000359228 | ENST00000423064 | ENST00000616259 |
| 33 | ENST00000541272 | ENST00000380672 | ENST00000612073 | ENST00000507007 |
| 34 | ENST00000360851 | ENST00000396625 | ENST00000526004 | ENST00000396276 |
| 35 | ENST00000244174 | ENST00000497872 | ENST00000450871 | ENST00000426706 |
| 36 | ENST00000390308 | ENST00000547327 | ENST00000244174 | ENST00000443723 |
| 37 | ENST00000473185 | ENST00000634222 | ENST00000490251 | ENST00000298527 |
| 38 | ENST00000321535 | ENST00000558197 | ENST00000451085 | ENST00000498435 |
| 39 | ENST00000580335 | ENST00000496168 | ENST0000395002 | ENST00000359228 |

| | | | | |
|---|---|---|---|---|
| 40 | ENST00000513886 | ENST00000616417 | ENST00000394718 | ENST00000633092 |
| 41 | ENST00000468856 | ENST00000438425 | ENST00000390548 | ENST00000491761 |
| 42 | ENST00000632136 | ENST00000390309 | ENST00000377712 | ENST00000376840 |
| 43 | ENST00000611391 | ENST00000390285 | ENST00000648973 | ENST00000415351 |
| 44 | ENST00000611977 | ENST00000483158 | ENST00000643024 | ENST00000648322 |
| 45 | ENST00000473726 | ENST00000416931 | ENST00000559610 | ENST00000316623 |
| 46 | ENST00000221307 | ENST00000464162 | ENST00000507007 | ENST00000404989 |
| 47 | ENST00000312143 | ENST00000390290 | ENST00000464162 | ENST00000464591 |
| 48 | ENST00000617175 | ENST00000360851 | ENST00000321535 | ENST00000295633 |
| 49 | ENST00000367434 | ENST00000636279 | ENST00000607161 | ENST00000580335 |
| 50 | ENST00000357484 | ENST00000276974 | ENST00000564734 | ENST00000612073 |
| 51 | ENST00000446507 | ENST00000392054 | ENST00000536374 | ENST00000489175 |
| 52 | ENST00000373095 | ENST00000622663 | ENST00000476232 | ENST00000362032 |
| 53 | ENST00000641095 | ENST00000422622 | ENST00000390549 | ENST00000392040 |
| 54 | ENST00000291576 | ENST00000610495 | ENST00000324907 | ENST00000632136 |
| 55 | ENST00000618644 | ENST00000390237 | ENST00000286732 | ENST00000641095 |
| 56 | ENST00000390547 | ENST00000394329 | ENST00000604204 | ENST00000390549 |
| 57 | ENST00000285379 | ENST00000280258 | ENST00000600255 | ENST00000621803 |
| 58 | ENST00000622749 | ENST00000393590 | ENST00000522551 | ENST00000390547 |
| 59 | ENST00000316623 | ENST00000367929 | ENST00000489175 | ENST00000641136 |
| 60 | ENST00000291232 | ENST00000390319 | ENST00000357325 | ENST00000613640 |
| 61 | ENST00000394329 | ENST00000522551 | ENST00000244745 | ENST00000497872 |
| 62 | ENST00000392040 | ENST00000392040 | ENST00000641136 | ENST00000390539 |
| 63 | ENST00000359357 | ENST00000480786 | ENST00000635923 | ENST00000390323 |
| 64 | ENST00000295633 | ENST00000580335 | ENST00000482518 | ENST00000390321 |
| 65 | ENST00000621803 | ENST00000631869 | ENST00000480757 | ENST00000390237 |
| 66 | ENST00000321016 | ENST00000411764 | ENST00000453044 | ENST00000632774 |
| 67 | ENST00000341184 | ENST00000392055 | ENST00000394329 | ENST00000390601 |
| 68 | ENST00000450871 | ENST00000611977 | ENST00000380672 | ENST00000631869 |
| 69 | ENST00000375448 | ENST00000400072 | ENST00000359062 | ENST00000468494 |
| 70 | ENST00000390624 | ENST00000631065 | ENST00000324079 | ENST00000390256 |
| 71 | ENST00000580919 | ENST00000390294 | ENST00000263382 | ENST00000390629 |
| 72 | ENST00000359228 | ENST00000611391 | ENST00000634222 | ENST00000390624 |
| 73 | ENST00000632774 | ENST00000632774 | ENST00000610261 | ENST00000618644 |
| 74 | ENST00000537147 | ENST00000390306 | ENST00000534952 | ENST00000610349 |
| 75 | ENST00000451998 | ENST00000396618 | ENST00000513886 | ENST00000454421 |
| 76 | ENST00000396625 | ENST00000321535 | ENST00000506073 | ENST00000448155 |
| 77 | ENST00000492446 | ENST00000631690 | ENST00000481799 | ENST00000390598 |
| 78 | ENST00000444393 | ENST00000543780 | ENST00000480786 | ENST00000390283 |
| 79 | ENST00000390312 | ENST00000480492 | ENST00000477714 | ENST00000390625 |
| 80 | ENST00000324079 | ENST00000309575 | ENST00000444393 | ENST00000390594 |
| 81 | ENST00000311597 | ENST00000307428 | ENST00000439754 | ENST00000480492 |
| 82 | ENST00000390304 | ENST00000390314 | ENST00000424873 | ENST00000434710 |

| | | | | |
|---|---|---|---|---|
| 83 | ENST00000507411 | ENST00000324907 | ENST00000422987 | ENST00000611391 |
| 84 | ENST00000297785 | ENST00000393203 | ENST00000396276 | ENST00000492446 |
| 85 | ENST00000573760 | ENST00000390304 | ENST00000390310 | ENST00000492167 |
| 86 | ENST00000476232 | ENST00000559610 | ENST00000390308 | ENST00000483158 |
| 87 | ENST00000507874 | ENST00000507411 | ENST00000367929 | ENST00000496168 |
| 88 | ENST00000618003 | ENST00000558711 | ENST00000360851 | ENST00000390304 |
| 89 | ENST00000526097 | ENST00000594028 | ENST00000311597 | ENST00000390305 |
| 90 | ENST00000493819 | ENST00000296029 | ENST00000284984 | ENST00000464162 |
| 91 | ENST00000483295 | ENST00000632136 | ENST00000261381 | ENST00000390606 |
| 92 | ENST00000393203 | ENST00000313401 | ENST00000611391 | ENST00000491977 |
| 93 | ENST00000634222 | ENST00000274605 | ENST00000584793 | ENST00000493819 |
| 94 | ENST00000426706 | ENST00000442677 | ENST00000573760 | ENST00000390243 |
| 95 | ENST00000377712 | ENST00000367051 | ENST00000553927 | ENST00000479981 |
| 96 | ENST00000487272 | ENST00000297785 | ENST00000550402 | ENST00000390252 |
| 97 | ENST00000556751 | ENST00000506927 | ENST00000528808 | ENST00000390309 |
| 98 | ENST00000390323 | ENST00000513778 | ENST00000526893 | ENST00000390306 |
| 99 | ENST00000564734 | ENST00000541272 | ENST00000415351 | ENST00000390312 |
| 100 | ENST00000330953 | ENST00000329099 | ENST00000390304 | ENST00000390319 |
| 101 | ENST00000329099 | ENST00000281821 | ENST00000340342 | ENST00000390308 |
| 102 | ENST00000267814 | ENST00000259089 | ENST00000316623 | ENST00000473726 |
| 103 | ENST00000342032 | ENST00000618003 | ENST00000298223 | ENST00000390285 |
| 104 | ENST00000636279 | ENST00000290866 | ENST00000296029 | ENST00000620395 |
| 105 | ENST00000380672 | ENST00000509697 | ENST00000285379 | ENST00000390310 |
| 106 | ENST00000416931 | ENST00000390625 | ENST00000276974 | ENST00000390294 |
| 107 | ENST00000396618 | ENST00000616259 | ENST00000228705 | ENST00000390290 |
| 108 | ENST00000367467 | ENST00000305141 | ENST00000200457 | ENST00000390314 |
| 109 | ENST00000368237 | ENST00000574371 | ENST00000523272 | ENST00000541272 |
| 110 | ENST00000390270 | ENST00000612073 | ENST00000509697 | ENST00000357325 |
| 111 | ENST00000298527 | ENST00000519554 | ENST00000482769 | ENST00000394718 |
| 112 | ENST00000492167 | ENST00000324079 | ENST00000443723 | ENST00000556751 |
| 113 | ENST00000255409 | ENST00000643697 | ENST00000396625 | ENST00000294435 |
| 114 | ENST00000434710 | ENST00000295633 | ENST00000390321 | ENST00000612503 |
| 115 | ENST00000393590 | ENST00000641136 | ENST00000379982 | ENST00000313401 |
| 116 | ENST00000548358 | ENST00000221307 | ENST00000369718 | ENST00000368237 |
| 117 | ENST00000534952 | ENST00000321016 | ENST00000367051 | ENST00000396789 |
| 118 | ENST00000443723 | ENST00000405943 | ENST00000359357 | ENST00000321935 |
| 119 | ENST00000005178 | ENST00000390325 | ENST00000309575 | ENST00000264808 |
| 120 | ENST00000598234 | ENST00000560582 | ENST00000298527 | ENST00000368034 |
| 121 | ENST00000367929 | ENST00000375448 | ENST00000296028 | ENST00000550402 |
| 122 | ENST00000278919 | ENST00000448155 | ENST00000267814 | ENST00000380490 |
| 123 | ENST00000633446 | ENST00000482769 | ENST00000264790 | ENST00000446507 |
| 124 | ENST00000586582 | ENST00000509152 | ENST00000632136 | ENST00000305097 |
| 125 | ENST00000616259 | ENST00000526893 | ENST00000631869 | ENST00000367721 |

| | | | | |
|---|---|---|---|---|
| 126 | ENST00000390252 | ENST00000290902 | ENST00000623011 | ENST00000621600 |
| 127 | ENST00000558711 | ENST00000298223 | ENST00000622044 | ENST00000414455 |
| 128 | ENST00000559610 | ENST00000424873 | ENST00000616430 | ENST00000622663 |
| 129 | ENST00000547327 | ENST00000467942 | ENST00000580919 | ENST00000443956 |
| 130 | ENST00000464835 | ENST00000396789 | ENST00000555838 | ENST00000342032 |
| 131 | ENST00000256447 | ENST00000396578 | ENST00000540271 | ENST00000553927 |
| 132 | ENST00000286732 | ENST00000404989 | ENST00000519554 | ENST00000264377 |
| 133 | ENST00000643697 | ENST00000265022 | ENST00000506927 | ENST00000262139 |
| 134 | ENST00000250360 | ENST00000536374 | ENST00000497872 | ENST00000267814 |
| 135 | ENST00000471490 | ENST00000259989 | ENST00000493550 | ENST00000617716 |
| 136 | ENST00000371528 | ENST00000367256 | ENST00000491761 | ENST00000564734 |
| 137 | ENST00000379982 | ENST00000342032 | ENST00000468856 | ENST00000216341 |
| 138 | ENST00000615863 | ENST00000307851 | ENST00000433557 | ENST00000526004 |
| 139 | ENST00000305141 | ENST00000561418 | ENST00000405943 | ENST00000274605 |
| 140 | ENST00000505763 | ENST00000377712 | ENST00000404989 | ENST00000260526 |
| 141 | ENST00000448155 | ENST00000464591 | ENST00000392055 | ENST00000436743 |
| 142 | ENST00000480786 | ENST00000468856 | ENST00000390252 | ENST00000482518 |
| 143 | ENST00000480697 | ENST00000390243 | ENST00000367256 | ENST00000534952 |
| 144 | ENST00000498146 | ENST00000446507 | ENST00000312143 | ENST00000285379 |
| 145 | ENST00000404989 | ENST00000534952 | ENST00000280258 | ENST00000306051 |
| 146 | ENST00000390314 | ENST00000311597 | ENST00000260526 | ENST00000281821 |
| 147 | ENST00000405943 | ENST00000555838 | ENST00000240100 | ENST00000616430 |
| 148 | ENST00000441556 | ENST00000453044 | ENST00000633092 | ENST00000280258 |
| 149 | ENST00000334529 | ENST00000493550 | ENST00000618889 | ENST00000532234 |
| 150 | ENST00000560582 | ENST00000440480 | ENST00000617889 | ENST00000278919 |
| 151 | ENST00000277225 | ENST00000471490 | ENST00000615863 | ENST00000367929 |
| 152 | ENST00000261651 | ENST00000226279 | ENST00000612503 | ENST00000493550 |
| 153 | ENST00000442677 | ENST00000240100 | ENST00000610495 | ENST00000453044 |
| 154 | ENST00000424873 | ENST00000261381 | ENST00000547327 | ENST00000341935 |
| 155 | ENST00000615525 | ENST00000573760 | ENST00000507874 | ENST00000537147 |
| 156 | ENST00000422622 | ENST00000379982 | ENST00000502981 | ENST00000005178 |
| 157 | ENST00000390549 | ENST00000278919 | ENST00000498165 | ENST00000199389 |
| 158 | ENST00000438425 | ENST00000330953 | ENST00000498146 | ENST00000424347 |
| 159 | ENST00000561418 | ENST00000641095 | ENST00000479981 | ENST00000536374 |
| 160 | ENST00000544802 | ENST00000565135 | ENST00000448387 | ENST00000367245 |
| 161 | ENST00000508643 | ENST00000053243 | ENST00000440480 | ENST00000250360 |
| 162 | ENST00000390290 | ENST00000610261 | ENST00000403687 | ENST00000258807 |
| 163 | ENST00000522551 | ENST00000502981 | ENST00000399220 | ENST00000444393 |
| 164 | ENST00000400072 | ENST00000564734 | ENST00000396789 | ENST00000272163 |
| 165 | ENST00000622044 | ENST00000312143 | ENST00000392040 | ENST00000265022 |
| 166 | ENST00000555838 | ENST00000367255 | ENST00000390625 | ENST00000558711 |
| 167 | ENST00000622663 | ENST00000615525 | ENST00000390624 | ENST00000558197 |
| 168 | ENST00000620395 | ENST00000633092 | ENST00000390325 | ENST00000616417 |

| | | | | |
|---|---|---|---|---|
| 169 | ENST00000403687 | ENST00000434710 | ENST00000390314 | ENST00000480769 |
| 170 | ENST00000550772 | ENST00000390270 | ENST00000390294 | ENST00000264424 |
| 171 | ENST00000298223 | ENST00000532234 | ENST00000362032 | ENST00000380672 |
| 172 | ENST00000635923 | ENST00000619589 | ENST00000341935 | ENST00000484726 |
| 173 | ENST00000240100 | ENST00000277225 | ENST00000291232 | ENST00000307851 |
| 174 | ENST00000390598 | ENST00000525499 | ENST00000277225 | ENST00000367688 |
| 175 | ENST00000453044 | ENST00000371528 | ENST00000272224 | ENST00000547327 |
| 176 | ENST00000526004 | ENST00000305097 | ENST00000264808 | ENST00000617889 |
| 177 | ENST00000390237 | ENST00000379287 | ENST00000264246 | ENST00000296029 |
| 178 | ENST00000507007 | ENST00000503004 | ENST00000262158 | ENST00000296028 |
| 179 | ENST00000553927 | ENST00000390310 | ENST00000053243 | ENST00000366899 |
| 180 | ENST00000467942 | ENST00000390624 | ENST00000636279 | ENST00000291232 |
| 181 | ENST00000199389 | ENST00000264246 | ENST00000622749 | ENST00000548358 |
| 182 | ENST00000316418 | ENST00000484726 | ENST00000621803 | ENST00000615863 |
| 183 | ENST00000390294 | ENST00000620457 | ENST00000616259 | ENST00000613947 |
| 184 | ENST00000430223 | ENST00000492446 | ENST00000593178 | ENST00000439754 |
| 185 | ENST00000482518 | ENST00000367245 | ENST00000580335 | ENST00000307428 |
| 186 | ENST00000491761 | ENST00000648973 | ENST00000571489 | ENST00000650242 |
| 187 | ENST00000477714 | ENST00000244745 | ENST00000558711 | ENST00000619589 |
| 188 | ENST00000053243 | ENST00000493819 | ENST00000541272 | ENST00000369718 |
| 189 | ENST00000631690 | ENST00000423064 | ENST00000525499 | ENST00000373209 |
| 190 | ENST00000594028 | ENST00000378867 | ENST00000492446 | ENST00000433557 |
| 191 | ENST00000390243 | ENST00000498165 | ENST00000478742 | ENST00000448387 |
| 192 | ENST00000290866 | ENST00000341935 | ENST00000471490 | ENST00000359357 |
| 193 | ENST00000390601 | ENST00000250360 | ENST00000466159 | ENST00000466595 |
| 194 | ENST00000503771 | ENST00000399220 | ENST00000464589 | ENST00000319211 |
| 195 | ENST00000440480 | ENST00000390256 | ENST00000442677 | ENST00000367255 |
| 196 | ENST00000607161 | ENST00000367688 | ENST00000420836 | ENST00000367256 |
| 197 | ENST00000290902 | ENST00000284984 | ENST00000396618 | ENST00000469439 |
| 198 | ENST00000483158 | ENST00000489171 | ENST00000396578 | ENST00000461872 |
| 199 | ENST00000509152 | ENST00000306051 | ENST00000392993 | ENST00000466159 |
| 200 | ENST00000264808 | ENST00000617889 | ENST00000390319 | ENST00000396625 |
| 201 | ENST00000362032 | ENST00000480697 | ENST00000390283 | ENST00000302125 |
| 202 | ENST00000633685 | ENST00000635923 | ENST00000376840 | ENST00000503771 |
| 203 | ENST00000478742 | ENST00000544802 | ENST00000373095 | ENST00000255409 |
| 204 | ENST00000200457 | ENST00000461872 | ENST00000367721 | ENST00000472064 |
| 205 | ENST00000480492 | ENST00000420843 | ENST00000367434 | ENST00000371528 |
| 206 | ENST00000618026 | ENST00000477714 | ENST00000331289 | ENST00000340342 |
| 207 | ENST00000633092 | ENST00000491977 | ENST00000329099 | ENST00000291576 |
| 208 | ENST00000466595 | ENST00000476232 | ENST00000322019 | ENST00000256447 |
| 209 | ENST00000593178 | ENST00000381031 | ENST00000307851 | ENST00000396578 |
| 210 | ENST00000529814 | ENST00000272163 | ENST00000307407 | ENST00000359062 |
| 211 | ENST00000525499 | ENST00000529814 | ENST00000305141 | ENST00000177694 |

| | | | | |
|---|---|---|---|---|
| 212 | ENST00000506927 | ENST00000302125 | ENST00000291576 | ENST00000329099 |
| 213 | ENST00000244745 | ENST00000480769 | ENST00000281821 | ENST00000394329 |
| 214 | ENST00000390309 | ENST00000426706 | ENST00000264424 | ENST00000379757 |
| 215 | ENST00000631869 | ENST00000584793 | ENST00000264377 | ENST00000622044 |
| 216 | ENST00000390283 | ENST00000390312 | ENST00000250360 | ENST00000261381 |
| 217 | ENST00000415351<br>-0.00148 | ENST00000369718 | ENST00000005178 | ENST00000396618 |
| 218 | ENST00000489175 | ENST00000478742 | ENST00000650242 | ENST00000643697 |
| 219 | ENST00000420836 | ENST00000366899 | ENST00000632774 | ENST00000226279 |
| 220 | ENST00000479981 | ENST00000480757 | ENST00000631065 | ENST00000422987 |
| 221 | ENST00000390625 | ENST00000285379 | ENST00000616417 | ENST00000451085 |
| 222 | ENST00000263382 | ENST00000482518 | ENST00000613640 | ENST00000394684 |
| 223 | ENST00000390539 | ENST00000650242 | ENST00000610349 | ENST00000550772 |
| 224 | ENST00000390256 | ENST00000373095 | ENST00000594028 | ENST00000264246 |
| 225 | ENST00000498435 | ENST00000472064 | ENST00000565135 | ENST00000053243 |
| 226 | ENST00000612073 | ENST00000617716 | ENST00000560582 | ENST00000379982 |
| 227 | ENST00000390321 | ENST00000503771 | ENST00000514989 | ENST00000240100 |
| 228 | ENST00000359062 | ENST00000414455 | ENST00000492167 | ENST00000525499 |
| 229 | ENST00000259089 | ENST00000286732 | ENST00000489171 | ENST00000321535 |
| 230 | ENST00000390310 | ENST00000643024 | ENST00000487272 | ENST00000378867 |
| 231 | ENST00000472064 | ENST00000331289 | ENST00000484726 | ENST00000334529 |
| 232 | ENST00000484726 | ENST00000263382 | ENST00000480769 | ENST00000540271 |
| 233 | ENST00000448387 | ENST00000540271 | ENST00000480697 | ENST00000584793 |
| 234 | ENST00000523272 | ENST00000521304 | ENST00000472064 | ENST00000309575 |
| 235 | ENST00000650242 | ENST00000513886 | ENST00000469439 | ENST00000375448 |
| 236 | ENST00000646615 | ENST00000507007 | ENST00000454421 | ENST00000392993 |
| 237 | ENST00000260526 | ENST00000264377 | ENST00000446507 | ENST00000480757 |
| 238 | ENST00000618889 | ENST00000264424 | ENST00000434710 | ENST00000483295 |
| 239 | ENST00000321935 | ENST00000450871 | ENST00000429492 | ENST00000235382 |
| 240 | ENST00000561158 | ENST00000248598 | ENST00000424347 | ENST00000464302 |
| 241 | ENST00000423064 | ENST00000307407 | ENST00000394684 | ENST00000330953 |
| 242 | ENST00000513778 | ENST00000507874 | ENST00000390629 | ENST00000367434 |
| 243 | ENST00000543780 | ENST00000362032 | ENST00000390606 | ENST00000498146 |
| 244 | ENST00000264790 | ENST00000415351 | ENST00000390601 | ENST00000480697 |
| 245 | ENST00000514989 | ENST00000424347 | ENST00000390547 | ENST00000571489 |
| 246 | ENST00000521304 | ENST00000392993 | ENST00000390309 | ENST00000467942 |
| 247 | ENST00000272224 | ENST00000430223 | ENST00000390306 | ENST00000505763 |
| 248 | ENST00000509697 | ENST00000621600 | ENST00000390305 | ENST00000420843 |
| 249 | ENST00000264246 | ENST00000394684 | ENST00000390256 | ENST00000543780 |
| 250 | ENST00000637526 | ENST00000296028 | ENST00000381031 | ENST00000286732 |
| 251 | ENST00000613640 | ENST00000483295 | ENST00000367245 | ENST00000399220 |
| 252 | ENST00000341935 | ENST00000505763 | ENST00000357484 | ENST00000276974 |
| 253 | ENST00000454421 | ENST00000414273 | ENST00000295633 | ENST00000298223 |

| | | | | |
|---|---|---|---|---|
| 254 | ENST00000264377 | ENST00000550402 | ENST00000290902 | ENST00000305141 |
| 255 | ENST00000464591 | ENST00000616430 | ENST00000290866 | ENST00000555619 |
| 256 | ENST00000496168 | ENST00000633685 | ENST00000274605 | ENST00000357484 |
| 257 | ENST00000395002 | ENST00000444393 | ENST00000265022 | ENST00000284984 |
| 258 | ENST00000309575 | ENST00000448387 | ENST00000262139 | ENST00000464589 |
| 259 | ENST00000620457 | ENST00000618889 | ENST00000256447 | ENST00000379287 |
| 260 | ENST00000390594 | ENST00000228705 | ENST00000226279 | ENST00000322019 |
| 261 | ENST00000610261 | ENST00000368034 | ENST00000637526 | ENST00000477714 |
| 262 | ENST00000482769 | ENST00000342456 | ENST00000621600 | ENST00000514989 |
| 263 | ENST00000439754 | ENST00000466159 | ENST00000620395 | ENST00000277225 |
| 264 | ENST00000532234 | ENST00000260526 | ENST00000618026 | ENST00000498165 |
| 265 | ENST00000296028 | ENST00000255409 | ENST00000587916 | ENST00000310954 |
| 266 | ENST00000631065 | ENST00000621803 | ENST00000556751 | ENST00000526893 |
| 267 | ENST00000616430 | ENST00000489175 | ENST00000555619 | ENST00000560582 |
| 268 | ENST00000390306 | ENST00000613640 | ENST00000548358 | ENST00000264790 |
| 269 | ENST00000503004 | ENST00000310954 | ENST00000532234 | ENST00000513778 |
| 270 | ENST00000264424 | ENST00000556751 | ENST00000503771 | ENST00000221307 |
| 271 | ENST00000443956 | ENST00000340342 | ENST00000493819 | ENST00000373095 |
| 272 | ENST00000274605 | ENST00000264790 | ENST00000473726 | ENST00000290902 |
| 273 | ENST00000390305 | ENST00000199389 | ENST00000466595 | ENST00000509697 |
| 274 | ENST00000502981 | ENST00000464302 | ENST00000461872 | ENST00000489171 |
| 275 | ENST00000429492 | ENST00000373209 | ENST00000451998 | ENST00000622749 |
| 276 | ENST00000610349 | ENST00000612503 | ENST00000443956 | ENST00000262094 |
| 277 | ENST00000536374 | ENST00000468494 | ENST00000420843 | ENST00000228705 |
| 278 | ENST00000526893 | ENST00000200457 | ENST00000416931 | ENST00000506073 |
| 279 | ENST00000550402 | ENST00000357484 | ENST00000390539 | ENST00000259989 |
| 280 | ENST00000571489 | ENST00000498146 | ENST00000390270 | ENST00000248598 |
| 281 | ENST00000612503 | ENST00000615863 | ENST00000380490 | ENST00000423064 |
| 282 | ENST00000307407 | ENST00000376840 | ENST00000379287 | ENST00000643024 |
| 283 | ENST00000390319 | ENST00000454421 | ENST00000375448 | ENST00000519554 |
| 284 | ENST00000617716 | ENST00000261651 | ENST00000368237 | ENST00000429492 |
| 285 | ENST00000493550 | ENST00000395002 | ENST00000367688 | ENST00000263382 |
| 286 | ENST00000464302 | ENST00000623011 | ENST00000366899 | ENST00000331289 |
| 287 | ENST00000390629 | ENST00000622044 | ENST00000342456 | ENST00000637526 |
| 288 | ENST00000357325 | ENST00000571489 | ENST00000321935 | ENST00000216117 |
| 289 | ENST00000261381 | ENST00000291576 | ENST00000321348 | ENST00000381031 |
| 290 | ENST00000396578 | ENST00000479981 | ENST00000255409 | ENST00000451998 |
| 291 | ENST00000621600 | ENST00000262094 | ENST00000235382 | ENST00000610261 |
| 292 | ENST00000466159 | ENST00000469439 | ENST00000641095 | ENST00000509152 |
| 293 | ENST00000284984 | ENST00000439754 | ENST00000633446 | ENST00000502981 |
| 294 | ENST00000390606 | ENST00000491761 | ENST00000631690 | ENST00000259089 |
| 295 | ENST00000379757 | ENST00000443956 | ENST00000618003 | ENST00000526097 |
| 296 | ENST00000604204 | ENST00000604204 | ENST00000615525 | ENST00000342456 |

| | | | | |
|---|---|---|---|---|
| 297 | ENST00000600255 | ENST00000396276 | ENST00000558197 | ENST00000403687 |
| 298 | ENST00000623011 | ENST00000262158 | ENST00000537147 | ENST00000468856 |
| 299 | ENST00000396276 | ENST00000598234 | ENST00000521304 | ENST00000610495 |
| 300 | ENST00000340342 | ENST00000506073 | ENST00000513778 | ENST00000393158 |
| 301 | ENST00000464162 | ENST00000433557 | ENST00000508643 | ENST00000321348 |
| 302 | ENST00000464589 | ENST00000321348 | ENST00000507411 | ENST00000411764 |
| 303 | ENST00000481799 | ENST00000267814 | ENST00000505763 | ENST00000618003 |
| 304 | ENST00000587916 | ENST00000451998 | ENST00000498435 | ENST00000646615 |
| 305 | ENST00000302125 | ENST00000528808 | ENST00000496168 | ENST00000635923 |
| 306 | ENST00000461872 | ENST00000316623 | ENST00000491977 | ENST00000521304 |
| 307 | ENST00000262139 | ENST00000216117 | ENST00000483295 | ENST00000523272 |
| 308 | ENST00000331289 | ENST00000537147 | ENST00000468494 | ENST00000587916 |
| 309 | ENST00000305097 | ENST00000262139 | ENST00000467942 | ENST00000481799 |
| 310 | ENST00000280258 | ENST00000466595 | ENST00000448155 | ENST00000513886 |
| 311 | ENST00000490251 | ENST00000298527 | ENST00000436743 | ENST00000261651 |
| 312 | ENST00000468494 | ENST00000464589 | ENST00000411764 | ENST00000631065 |
| 313 | ENST00000648322 | ENST00000553927 | ENST00000393158 | ENST00000580919 |
| 314 | ENST00000226279 | ENST00000548358 | ENST00000390285 | ENST00000555838 |
| 315 | ENST00000396789 | ENST00000393158 | ENST00000373209 | ENST00000565135 |
| 316 | ENST00000306051 | ENST00000508643 | ENST00000368034 | ENST00000400072 |
| 317 | ENST00000373209 | ENST00000235382 | ENST00000334529 | ENST00000600255 |
| 318 | ENST00000228705 | ENST00000258807 | ENST00000330953 | ENST00000420836 |
| 319 | ENST00000376840 | ENST00000390601 | ENST00000313401 | ENST00000522551 |
| 320 | ENST00000613947 | ENST00000618026 | ENST00000307428 | ENST00000508643 |
| 321 | ENST00000528808 | ENST00000593178 | ENST00000305097 | ENST00000574371 |
| 322 | ENST00000540271 | ENST00000321935 | ENST00000278919 | ENST00000604204 |
| 323 | ENST00000321348 | ENST00000473185 | ENST00000262094 | ENST00000405943 |
| 324 | ENST00000617889 | ENST00000481799 | ENST00000221307 | ENST00000561418 |
| 325 | ENST00000235382 | ENST00000368237 | ENST00000199389 | ENST00000544802 |
| 326 | ENST00000294435 | ENST00000526004 | ENST00000646615 | ENST00000416931 |
| 327 | ENST00000619589 | ENST00000443723 | ENST00000643697 | ENST00000561158 |
| 328 | ENST00000342456 | ENST00000359062 | ENST00000618644 | ENST00000414273 |
| 329 | ENST00000216341 | ENST00000555619 | ENST00000561418 | ENST00000471490 |
| 330 | ENST00000480757 | ENST00000490251 | ENST00000550772 | ENST00000506927 |
| 331 | ENST00000394684 | ENST00000637526 | ENST00000544802 | ENST00000200457 |
| 332 | ENST00000643024 | ENST00000322019 | ENST00000543780 | ENST00000559610 |
| 333 | ENST00000262094 | ENST00000403687 | ENST00000526097 | ENST00000607161 |
| 334 | ENST00000433557 | ENST00000319211 | ENST00000503004 | ENST00000634222 |
| 335 | ENST00000565135 | ENST00000380490 | ENST00000483158 | ENST00000307407 |
| 336 | ENST00000436743 | ENST00000005178 | ENST00000473185 | ENST00000290866 |
| 337 | ENST00000498165 | ENST00000390594 | ENST00000438425 | ENST00000244174 |
| 338 | ENST00000379287 | ENST00000256447 | ENST00000414273 | ENST00000360851 |
| 339 | ENST00000310954 | ENST00000177694 | ENST00000390598 | ENST00000636279 |

| | | | | |
|---|---|---|---|---|
| 340 | ENST00000393158 | ENST00000607161 | ENST00000390594 | ENST00000618889 |
| 341 | ENST00000367688 | ENST00000367721 | ENST00000390312 | ENST00000573760 |
| 342 | ENST00000259989 | ENST00000420836 | ENST00000390290 | ENST00000633446 |
| 343 | ENST00000506073 | ENST00000359357 | ENST00000390237 | ENST00000529814 |
| 344 | ENST00000469439 | ENST00000357325 | ENST00000378867 | ENST00000598234 |
| 345 | ENST00000322019 | ENST00000561158 | ENST00000371528 | ENST00000480786 |
| 346 | ENST00000394718 | ENST00000498435 | ENST00000367255 | ENST00000390325 |
| 347 | ENST00000380490 | ENST00000648322 | ENST00000342032 | ENST00000390548 |
| 348 | ENST00000367255 | ENST00000436743 | ENST00000310954 | ENST00000438425 |
| 349 | ENST00000216117 | ENST00000526097 | ENST00000302125 | ENST00000316418 |
| 350 | ENST00000648973 | ENST00000334529 | ENST00000294435 | ENST00000503004 |
| 351 | ENST00000262158 | ENST00000613947 | ENST00000272163 | ENST00000393590 |
| 352 | ENST00000367721 | ENST00000622749 | ENST00000259989 | ENST00000507874 |
| 353 | ENST00000319211 | ENST00000379757 | ENST00000248598 | ENST00000393203 |
| 354 | ENST00000399220 | ENST00000580919 | ENST00000216341 | ENST00000507411 |
| 355 | ENST00000258807 | ENST00000291232 | ENST00000216117 | ENST00000450871 |
| 356 | ENST00000381031 | ENST00000272224 | ENST00000617175 | ENST00000617175 |
| 357 | ENST00000489171 | ENST00000390629 | ENST00000613947 | ENST00000392055 |
| 358 | ENST00000555619 | ENST00000620395 | ENST00000574371 | ENST00000422622 |
| 359 | ENST00000378867 | ENST00000216341 | ENST00000561158 | ENST00000464835 |
| 360 | ENST00000369718 | ENST00000390283 | ENST00000480492 | ENST00000367051 |
| 361 | ENST00000366899 | ENST00000294435 | ENST00000464302 | ENST00000487272 |
| 362 | ENST00000248598 | ENST00000600255 | ENST00000390323 | ENST00000339223 |
| 363 | ENST00000424347 | ENST00000646615 | ENST00000390243 | ENST00000341184 |
| 364 | ENST00000272163 | ENST00000514989 | ENST00000379757 | ENST00000441556 |
| 365 | ENST00000420843 | ENST00000587916 | ENST00000319211 | ENST00000321016 |
| 366 | ENST00000177694 | ENST00000394718 | ENST00000306051 | ENST00000586582 |
| 367 | ENST00000392993 | ENST00000264808 | ENST00000261651 | ENST00000308478 |
| 368 | ENST00000480769 | ENST00000390598 | ENST00000259089 | ENST00000367467 |
| 369 | ENST00000368034 | ENST00000429492 | ENST00000258807 | ENST00000392054 |
| 370 | ENST00000307851 | ENST00000618644 | ENST00000177694 | ENST00000284311 |
