# Supplementary material for: Identification of Smoking-Associated Transcriptome Aberration in Blood with Machine Learning Methods
Source: Biomed Res Int. 2023 Jan 4;2023:5333361. doi: 10.1155/2023/5333361 (PMC9833906; doi:10.1155/2023/5333361)
Supplement: Supplementary Materials — Table S1: feature ranking results obtained by mRMR, MCFS, LightGBM, and LASSO methods. Table S2: IFS results on different feature lists. Table S3: intersection of the optimal feature subsets extracted from mRMR, MCFS, LightGBM, and LASSO feature lists. The features that appear in 4, 3, 2, and 1 optimal feature subsets are shown. Table S4: classification rules generated by the optimal DT model. Table S5: GO and KEGG enrichment results after merging the optimal feature subsets of the four feature ranking algorithms. [file 5333361.f1.zip › Table S2 (1).pdf]

Table S2: IFS results on different feature lists.

(1) IFS results on the mRMR feature list

Classification algorithm	Number of features	SN	SP	ACC	MCC	Precision	F1-measure
DT	1	0.729	0.738	0.735	0.455	0.622	0.671
DT	2	0.747	0.759	0.754	0.494	0.647	0.693
DT	3	0.784	0.793	0.790	0.565	0.691	0.735
DT	4	0.742	0.764	0.756	0.495	0.651	0.693
DT	5	0.773	0.777	0.776	0.537	0.672	0.719
DT	6	0.707	0.797	0.763	0.499	0.673	0.690
DT	7	0.736	0.785	0.767	0.512	0.669	0.701
DT	8	0.733	0.786	0.767	0.511	0.670	0.700
DT	9	0.764	0.799	0.786	0.554	0.693	0.727
DT	10	0.769	0.794	0.785	0.552	0.688	0.726
DT	11	0.778	0.774	0.776	0.539	0.671	0.720
DT	12	0.727	0.786	0.764	0.505	0.668	0.696
DT	13	0.751	0.803	0.784	0.546	0.693	0.721
DT	14	0.722	0.797	0.769	0.513	0.678	0.699
DT	15	0.738	0.795	0.774	0.525	0.681	0.708
DT	16	0.725	0.794	0.768	0.512	0.676	0.699
DT	17	0.756	0.790	0.777	0.536	0.681	0.716
DT	18	0.775	0.790	0.785	0.554	0.686	0.728
DT	19	0.744	0.786	0.771	0.521	0.673	0.707
DT	20	0.747	0.786	0.771	0.523	0.674	0.708

DT	21	0.751	0.812	0.790	0.556	0.703	0.726
DT	22	0.749	0.814	0.790	0.556	0.704	0.726
DT	23	0.718	0.777	0.755	0.487	0.656	0.686
DT	24	0.727	0.780	0.760	0.498	0.661	0.693
DT	25	0.731	0.794	0.771	0.518	0.678	0.703
DT	26	0.756	0.789	0.776	0.534	0.679	0.715
DT	27	0.793	0.791	0.792	0.571	0.692	0.739
DT	28	0.751	0.787	0.774	0.529	0.677	0.712
DT	29	0.764	0.786	0.778	0.539	0.679	0.719
DT	30	0.758	0.794	0.781	0.542	0.685	0.720
DT	31	0.714	0.784	0.758	0.490	0.661	0.686
DT	32	0.756	0.789	0.776	0.534	0.679	0.715
DT	33	0.784	0.797	0.792	0.569	0.695	0.737
DT	34	0.784	0.789	0.787	0.560	0.687	0.733
DT	35	0.753	0.789	0.776	0.532	0.679	0.714
DT	36	0.756	0.777	0.769	0.521	0.667	0.709
DT	37	0.760	0.773	0.768	0.521	0.665	0.709
DT	38	0.747	0.798	0.779	0.536	0.686	0.715
DT	39	0.767	0.794	0.784	0.550	0.688	0.725
DT	40	0.771	0.781	0.777	0.540	0.676	0.720
DT	41	0.784	0.798	0.793	0.570	0.697	0.738
DT	42	0.758	0.773	0.767	0.519	0.664	0.708
DT	43	0.733	0.791	0.770	0.517	0.675	0.703
DT	44	0.753	0.807	0.787	0.552	0.698	0.725
DT	45	0.744	0.786	0.771	0.521	0.673	0.707

DT	46	0.738	0.793	0.772	0.522	0.678	0.707
DT	47	0.749	0.763	0.758	0.500	0.651	0.697
DT	48	0.756	0.780	0.771	0.524	0.670	0.710
DT	49	0.753	0.791	0.777	0.535	0.681	0.715
DT	50	0.773	0.786	0.781	0.547	0.682	0.724
DT	51	0.773	0.786	0.781	0.547	0.682	0.724
DT	52	0.815	0.781	0.794	0.580	0.688	0.746
DT	53	0.731	0.768	0.754	0.489	0.651	0.689
DT	54	0.767	0.785	0.778	0.540	0.678	0.720
DT	55	0.749	0.784	0.771	0.522	0.672	0.708
DT	56	0.775	0.781	0.779	0.544	0.677	0.723
DT	57	0.736	0.794	0.772	0.522	0.679	0.706
DT	58	0.753	0.804	0.785	0.550	0.695	0.723
DT	59	0.747	0.784	0.770	0.520	0.671	0.707
DT	60	0.756	0.791	0.778	0.537	0.682	0.717
DT	61	0.733	0.786	0.767	0.511	0.670	0.700
DT	62	0.764	0.776	0.771	0.528	0.669	0.713
DT	63	0.767	0.773	0.771	0.527	0.667	0.713
DT	64	0.753	0.793	0.778	0.536	0.683	0.716
DT	65	0.736	0.795	0.773	0.523	0.680	0.707
DT	66	0.760	0.767	0.764	0.514	0.658	0.706
DT	67	0.751	0.795	0.779	0.537	0.685	0.716
DT	68	0.733	0.781	0.763	0.505	0.665	0.697
DT	69	0.764	0.803	0.789	0.558	0.697	0.729
DT	70	0.722	0.784	0.761	0.498	0.664	0.692

DT	71	0.769	0.784	0.778	0.540	0.678	0.720
DT	72	0.729	0.790	0.767	0.512	0.673	0.700
DT	73	0.773	0.801	0.790	0.563	0.696	0.733
DT	74	0.742	0.790	0.772	0.524	0.677	0.708
DT	75	0.775	0.801	0.791	0.565	0.697	0.734
DT	76	0.762	0.820	0.799	0.575	0.715	0.738
DT	77	0.742	0.801	0.779	0.535	0.688	0.714
DT	78	0.767	0.801	0.788	0.557	0.695	0.729
DT	79	0.756	0.817	0.794	0.566	0.710	0.732
DT	80	0.769	0.787	0.781	0.545	0.682	0.723
DT	81	0.760	0.781	0.773	0.530	0.673	0.714
DT	82	0.744	0.787	0.771	0.523	0.675	0.708
DT	83	0.771	0.797	0.787	0.557	0.692	0.729
DT	84	0.780	0.772	0.775	0.538	0.669	0.720
DT	85	0.749	0.793	0.776	0.532	0.681	0.714
DT	86	0.751	0.810	0.788	0.553	0.700	0.725
DT	87	0.808	0.797	0.801	0.591	0.702	0.751
DT	88	0.778	0.795	0.789	0.561	0.692	0.732
DT	89	0.773	0.806	0.794	0.569	0.702	0.736
DT	90	0.751	0.774	0.766	0.514	0.663	0.705
DT	91	0.751	0.824	0.797	0.570	0.716	0.733
DT	92	0.727	0.784	0.762	0.502	0.665	0.695
DT	93	0.756	0.789	0.776	0.534	0.679	0.715
DT	94	0.806	0.793	0.798	0.585	0.697	0.748
DT	95	0.769	0.790	0.782	0.548	0.684	0.724

DT	96	0.789	0.790	0.790	0.566	0.690	0.736
DT	97	0.773	0.798	0.789	0.560	0.694	0.731
DT	98	0.716	0.812	0.776	0.525	0.693	0.704
DT	99	0.780	0.793	0.788	0.561	0.690	0.732
DT	100	0.780	0.799	0.792	0.568	0.697	0.736
DT	101	0.773	0.782	0.779	0.543	0.678	0.722
DT	102	0.729	0.802	0.775	0.525	0.685	0.707
DT	103	0.727	0.790	0.767	0.510	0.672	0.698
DT	104	0.784	0.819	0.806	0.593	0.719	0.750
DT	105	0.782	0.807	0.798	0.578	0.706	0.742
DT	106	0.762	0.814	0.794	0.568	0.708	0.734
DT	107	0.769	0.772	0.771	0.528	0.666	0.714
DT	108	0.756	0.784	0.773	0.528	0.674	0.712
DT	109	0.744	0.784	0.769	0.518	0.671	0.706
DT	110	0.771	0.793	0.785	0.552	0.688	0.727
DT	111	0.773	0.798	0.789	0.560	0.694	0.731
DT	112	0.760	0.804	0.788	0.556	0.697	0.727
DT	113	0.744	0.823	0.794	0.562	0.713	0.728
DT	114	0.804	0.803	0.803	0.594	0.707	0.753
DT	115	0.722	0.817	0.782	0.537	0.701	0.711
DT	116	0.800	0.816	0.810	0.604	0.720	0.758
DT	117	0.769	0.790	0.782	0.548	0.684	0.724
DT	118	0.760	0.803	0.787	0.554	0.696	0.726
DT	119	0.764	0.781	0.775	0.534	0.674	0.716
DT	120	0.767	0.781	0.776	0.536	0.674	0.718

DT	121	0.762	0.791	0.781	0.543	0.684	0.721
DT	122	0.758	0.803	0.786	0.552	0.695	0.725
DT	123	0.771	0.782	0.778	0.541	0.677	0.721
DT	124	0.744	0.807	0.784	0.545	0.695	0.719
DT	125	0.769	0.806	0.792	0.565	0.701	0.733
DT	126	0.753	0.789	0.776	0.532	0.679	0.714
DT	127	0.762	0.811	0.793	0.565	0.705	0.732
DT	128	0.769	0.811	0.795	0.571	0.706	0.736
DT	129	0.780	0.771	0.774	0.537	0.668	0.720
DT	130	0.775	0.785	0.781	0.548	0.681	0.725
DT	131	0.769	0.763	0.765	0.518	0.657	0.709
DT	132	0.760	0.806	0.789	0.557	0.698	0.728
DT	133	0.767	0.807	0.792	0.564	0.702	0.733
DT	134	0.793	0.795	0.794	0.575	0.696	0.742
DT	135	0.760	0.811	0.792	0.563	0.704	0.731
DT	136	0.769	0.803	0.790	0.562	0.698	0.732
DT	137	0.742	0.795	0.776	0.529	0.682	0.711
DT	138	0.773	0.798	0.789	0.560	0.694	0.731
DT	139	0.780	0.794	0.789	0.562	0.691	0.733
DT	140	0.771	0.802	0.790	0.563	0.697	0.732
DT	141	0.760	0.782	0.774	0.531	0.674	0.714
DT	142	0.780	0.801	0.793	0.569	0.698	0.737
DT	143	0.769	0.784	0.778	0.540	0.678	0.720
DT	144	0.771	0.802	0.790	0.563	0.697	0.732
DT	145	0.753	0.793	0.778	0.536	0.683	0.716

DT	146	0.762	0.814	0.794	0.568	0.708	0.734
DT	147	0.775	0.799	0.790	0.564	0.696	0.733
DT	148	0.775	0.797	0.789	0.561	0.693	0.732
DT	149	0.729	0.778	0.760	0.499	0.661	0.693
DT	150	0.760	0.798	0.784	0.548	0.690	0.723
DT	151	0.758	0.801	0.785	0.549	0.692	0.723
DT	152	0.742	0.797	0.776	0.531	0.684	0.712
DT	153	0.749	0.804	0.784	0.546	0.694	0.720
DT	154	0.797	0.812	0.807	0.598	0.715	0.754
DT	155	0.797	0.769	0.780	0.551	0.672	0.729
DT	156	0.742	0.799	0.778	0.534	0.686	0.713
DT	157	0.775	0.804	0.794	0.569	0.701	0.736
DT	158	0.771	0.799	0.789	0.560	0.694	0.731
DT	159	0.756	0.794	0.780	0.540	0.685	0.718
DT	160	0.749	0.810	0.787	0.551	0.700	0.723
DT	161	0.758	0.807	0.789	0.556	0.699	0.727
DT	162	0.756	0.832	0.803	0.583	0.727	0.741
DT	163	0.764	0.807	0.791	0.562	0.701	0.731
DT	164	0.760	0.827	0.802	0.581	0.722	0.740
DT	165	0.751	0.773	0.765	0.513	0.662	0.704
DT	166	0.767	0.815	0.797	0.573	0.710	0.737
DT	167	0.758	0.799	0.784	0.548	0.691	0.723
DT	168	0.773	0.784	0.780	0.545	0.679	0.723
DT	169	0.749	0.810	0.787	0.551	0.700	0.723
DT	170	0.740	0.807	0.782	0.541	0.694	0.716

DT	171	0.758	0.774	0.768	0.521	0.665	0.709
DT	172	0.749	0.799	0.781	0.540	0.688	0.717
DT	173	0.738	0.804	0.780	0.536	0.691	0.714
DT	174	0.742	0.810	0.785	0.546	0.698	0.719
DT	175	0.725	0.816	0.782	0.537	0.700	0.712
DT	176	0.756	0.782	0.772	0.527	0.673	0.712
DT	177	0.778	0.787	0.784	0.553	0.684	0.728
DT	178	0.760	0.778	0.771	0.527	0.670	0.712
DT	179	0.786	0.812	0.803	0.588	0.713	0.748
DT	180	0.731	0.797	0.772	0.521	0.680	0.705
DT	181	0.784	0.802	0.795	0.575	0.701	0.740
DT	182	0.731	0.791	0.769	0.515	0.675	0.702
DT	183	0.784	0.810	0.800	0.583	0.709	0.745
DT	184	0.780	0.815	0.802	0.585	0.714	0.745
DT	185	0.756	0.803	0.785	0.550	0.694	0.724
DT	186	0.758	0.808	0.790	0.558	0.701	0.728
DT	187	0.767	0.794	0.784	0.550	0.688	0.725
DT	188	0.756	0.790	0.777	0.536	0.681	0.716
DT	189	0.762	0.785	0.776	0.536	0.677	0.717
DT	190	0.753	0.793	0.778	0.536	0.683	0.716
DT	191	0.749	0.819	0.793	0.562	0.710	0.729
DT	192	0.791	0.793	0.792	0.571	0.693	0.739
DT	193	0.771	0.793	0.785	0.552	0.688	0.727
DT	194	0.742	0.786	0.770	0.519	0.673	0.706
DT	195	0.760	0.801	0.785	0.551	0.693	0.725

DT	196	0.773	0.804	0.793	0.567	0.701	0.735
DT	197	0.742	0.814	0.787	0.550	0.702	0.722
DT	198	0.727	0.793	0.768	0.512	0.675	0.700
DT	199	0.767	0.793	0.783	0.548	0.686	0.724
DT	200	0.762	0.804	0.789	0.558	0.698	0.728
DT	201	0.731	0.816	0.785	0.543	0.702	0.716
DT	202	0.740	0.787	0.770	0.519	0.673	0.705
DT	203	0.742	0.832	0.799	0.571	0.723	0.733
DT	204	0.769	0.816	0.799	0.577	0.712	0.739
DT	205	0.762	0.802	0.787	0.555	0.695	0.727
DT	206	0.771	0.785	0.780	0.544	0.680	0.722
DT	207	0.784	0.802	0.795	0.575	0.701	0.740
DT	208	0.762	0.811	0.793	0.565	0.705	0.732
DT	209	0.742	0.784	0.768	0.516	0.670	0.704
DT	210	0.775	0.821	0.804	0.589	0.720	0.747
DT	211	0.744	0.816	0.790	0.555	0.706	0.725
DT	212	0.747	0.814	0.789	0.554	0.703	0.724
DT	213	0.806	0.802	0.803	0.595	0.707	0.753
DT	214	0.767	0.793	0.783	0.548	0.686	0.724
DT	215	0.756	0.802	0.785	0.549	0.693	0.723
DT	216	0.753	0.806	0.786	0.551	0.697	0.724
DT	217	0.744	0.799	0.779	0.536	0.687	0.715
DT	218	0.707	0.795	0.762	0.497	0.672	0.689
DT	219	0.769	0.802	0.790	0.561	0.697	0.731
DT	220	0.736	0.808	0.781	0.538	0.694	0.714

DT	221	0.753	0.786	0.774	0.529	0.676	0.713
DT	222	0.786	0.815	0.804	0.591	0.715	0.749
DT	223	0.716	0.804	0.771	0.516	0.684	0.700
DT	224	0.747	0.791	0.775	0.529	0.679	0.711
DT	225	0.738	0.806	0.781	0.537	0.692	0.714
DT	226	0.793	0.810	0.803	0.591	0.711	0.750
DT	227	0.738	0.787	0.769	0.517	0.673	0.704
DT	228	0.762	0.803	0.788	0.556	0.696	0.728
DT	229	0.756	0.791	0.778	0.537	0.682	0.717
DT	230	0.740	0.808	0.783	0.542	0.696	0.717
DT	231	0.747	0.802	0.781	0.541	0.690	0.717
DT	232	0.722	0.825	0.787	0.546	0.710	0.716
DT	233	0.749	0.819	0.793	0.562	0.710	0.729
DT	234	0.751	0.806	0.785	0.549	0.696	0.722
DT	235	0.780	0.824	0.808	0.595	0.724	0.751
DT	236	0.727	0.808	0.778	0.530	0.692	0.709
DT	237	0.775	0.797	0.789	0.561	0.693	0.732
DT	238	0.769	0.807	0.793	0.566	0.702	0.734
DT	239	0.749	0.827	0.798	0.571	0.719	0.734
DT	240	0.736	0.820	0.789	0.552	0.708	0.721
DT	241	0.744	0.794	0.776	0.530	0.681	0.712
DT	242	0.760	0.816	0.795	0.569	0.710	0.734
DT	243	0.758	0.814	0.793	0.564	0.706	0.731
DT	244	0.784	0.806	0.798	0.579	0.705	0.742
DT	245	0.786	0.803	0.797	0.578	0.703	0.742

DT	246	0.800	0.819	0.812	0.607	0.723	0.759
DT	247	0.760	0.820	0.798	0.573	0.714	0.736
DT	248	0.758	0.798	0.783	0.546	0.689	0.722
DT	249	0.769	0.793	0.784	0.550	0.687	0.726
DT	250	0.742	0.807	0.783	0.543	0.695	0.718
DT	251	0.780	0.827	0.809	0.598	0.727	0.752
DT	252	0.771	0.814	0.798	0.576	0.710	0.739
DT	253	0.722	0.782	0.760	0.497	0.663	0.691
DT	254	0.767	0.799	0.787	0.556	0.693	0.728
DT	255	0.744	0.811	0.786	0.549	0.700	0.721
DT	256	0.744	0.799	0.779	0.536	0.687	0.715
DT	257	0.773	0.825	0.806	0.591	0.724	0.748
DT	258	0.758	0.780	0.771	0.526	0.671	0.711
DT	259	0.780	0.815	0.802	0.585	0.714	0.745
DT	260	0.795	0.819	0.810	0.603	0.722	0.757
DT	261	0.773	0.825	0.806	0.591	0.724	0.748
DT	262	0.797	0.799	0.799	0.584	0.702	0.746
DT	263	0.771	0.806	0.793	0.567	0.701	0.735
DT	264	0.733	0.801	0.776	0.527	0.685	0.709
DT	265	0.749	0.814	0.790	0.556	0.704	0.726
DT	266	0.760	0.816	0.795	0.569	0.710	0.734
DT	267	0.756	0.821	0.797	0.571	0.715	0.734
DT	268	0.733	0.804	0.778	0.532	0.689	0.711
DT	269	0.736	0.808	0.781	0.538	0.694	0.714
DT	270	0.762	0.806	0.790	0.559	0.699	0.729

DT	271	0.760	0.821	0.799	0.575	0.716	0.737
DT	272	0.736	0.807	0.781	0.537	0.693	0.714
DT	273	0.742	0.806	0.782	0.541	0.693	0.717
DT	274	0.749	0.803	0.783	0.544	0.692	0.720
DT	275	0.742	0.802	0.780	0.537	0.689	0.715
DT	276	0.740	0.799	0.777	0.532	0.686	0.712
DT	277	0.791	0.815	0.806	0.595	0.717	0.752
DT	278	0.744	0.825	0.795	0.565	0.716	0.730
DT	279	0.773	0.810	0.796	0.573	0.706	0.738
DT	280	0.751	0.786	0.773	0.527	0.675	0.711
DT	281	0.760	0.817	0.796	0.570	0.711	0.735
DT	282	0.747	0.817	0.791	0.558	0.708	0.727
DT	283	0.769	0.804	0.791	0.563	0.699	0.732
DT	284	0.769	0.801	0.789	0.559	0.695	0.730
DT	285	0.764	0.816	0.797	0.573	0.711	0.737
DT	286	0.780	0.819	0.804	0.590	0.718	0.748
DT	287	0.769	0.793	0.784	0.550	0.687	0.726
DT	288	0.764	0.816	0.797	0.573	0.711	0.737
DT	289	0.758	0.802	0.785	0.551	0.694	0.724
DT	290	0.784	0.821	0.808	0.596	0.722	0.752
DT	291	0.758	0.802	0.785	0.551	0.694	0.724
DT	292	0.740	0.802	0.779	0.535	0.689	0.713
DT	293	0.767	0.807	0.792	0.564	0.702	0.733
DT	294	0.756	0.782	0.772	0.527	0.673	0.712
DT	295	0.731	0.829	0.793	0.558	0.717	0.724

DT	296	0.758	0.825	0.800	0.577	0.720	0.738
DT	297	0.760	0.831	0.804	0.585	0.726	0.743
DT	298	0.789	0.790	0.790	0.566	0.690	0.736
DT	299	0.780	0.817	0.803	0.588	0.717	0.747
DT	300	0.756	0.795	0.781	0.541	0.686	0.719
DT	301	0.751	0.821	0.795	0.567	0.713	0.732
DT	302	0.758	0.815	0.794	0.565	0.708	0.732
DT	303	0.804	0.803	0.803	0.594	0.707	0.753
DT	304	0.797	0.804	0.802	0.589	0.707	0.749
DT	305	0.756	0.803	0.785	0.550	0.694	0.724
DT	306	0.749	0.793	0.776	0.532	0.681	0.714
DT	307	0.764	0.816	0.797	0.573	0.711	0.737
DT	308	0.769	0.811	0.795	0.571	0.706	0.736
DT	309	0.782	0.846	0.822	0.623	0.751	0.766
DT	310	0.751	0.819	0.794	0.564	0.710	0.730
DT	311	0.771	0.819	0.801	0.582	0.716	0.742
DT	312	0.773	0.810	0.796	0.573	0.706	0.738
DT	313	0.780	0.808	0.798	0.578	0.707	0.741
DT	314	0.775	0.831	0.810	0.599	0.730	0.752
DT	315	0.747	0.828	0.798	0.570	0.720	0.733
DT	316	0.791	0.811	0.803	0.591	0.712	0.749
DT	317	0.764	0.799	0.786	0.554	0.693	0.727
DT	318	0.760	0.797	0.783	0.547	0.689	0.723
DT	319	0.729	0.789	0.767	0.510	0.671	0.699
DT	320	0.780	0.806	0.796	0.575	0.704	0.740

DT	321	0.738	0.793	0.772	0.522	0.678	0.707
DT	322	0.756	0.803	0.785	0.550	0.694	0.724
DT	323	0.767	0.810	0.794	0.567	0.704	0.734
DT	324	0.773	0.806	0.794	0.569	0.702	0.736
DT	325	0.758	0.808	0.790	0.558	0.701	0.728
DT	326	0.760	0.803	0.787	0.554	0.696	0.726
DT	327	0.756	0.802	0.785	0.549	0.693	0.723
DT	328	0.782	0.812	0.801	0.584	0.711	0.745
DT	329	0.767	0.823	0.802	0.582	0.719	0.742
DT	330	0.771	0.817	0.800	0.580	0.714	0.742
DT	331	0.751	0.801	0.782	0.543	0.690	0.719
DT	332	0.791	0.814	0.805	0.594	0.715	0.751
DT	333	0.771	0.814	0.798	0.576	0.710	0.739
DT	334	0.767	0.801	0.788	0.557	0.695	0.729
DT	335	0.778	0.814	0.800	0.582	0.712	0.743
DT	336	0.791	0.811	0.803	0.591	0.712	0.749
DT	337	0.773	0.801	0.790	0.563	0.696	0.733
DT	338	0.738	0.790	0.771	0.520	0.675	0.705
DT	339	0.720	0.798	0.769	0.512	0.678	0.699
DT	340	0.773	0.806	0.794	0.569	0.702	0.736
DT	341	0.758	0.819	0.796	0.570	0.712	0.734
DT	342	0.751	0.824	0.797	0.570	0.716	0.733
DT	343	0.769	0.807	0.793	0.566	0.702	0.734
DT	344	0.769	0.833	0.809	0.596	0.732	0.750
DT	345	0.756	0.803	0.785	0.550	0.694	0.724

DT	346	0.751	0.824	0.797	0.570	0.716	0.733
DT	347	0.769	0.801	0.789	0.559	0.695	0.730
DT	348	0.758	0.810	0.790	0.559	0.702	0.729
DT	349	0.760	0.787	0.777	0.537	0.679	0.717
DT	350	0.718	0.793	0.765	0.504	0.672	0.694
DT	351	0.773	0.817	0.801	0.582	0.715	0.743
DT	352	0.753	0.798	0.781	0.542	0.688	0.719
DT	353	0.753	0.791	0.777	0.535	0.681	0.715
DT	354	0.756	0.803	0.785	0.550	0.694	0.724
DT	355	0.780	0.819	0.804	0.590	0.718	0.748
DT	356	0.749	0.820	0.794	0.563	0.711	0.730
DT	357	0.760	0.808	0.790	0.560	0.701	0.729
DT	358	0.749	0.798	0.780	0.538	0.687	0.717
DT	359	0.744	0.797	0.777	0.533	0.684	0.713
DT	360	0.749	0.820	0.794	0.563	0.711	0.730
DT	361	0.797	0.808	0.804	0.594	0.711	0.752
DT	362	0.778	0.817	0.803	0.586	0.716	0.746
DT	363	0.764	0.801	0.787	0.555	0.694	0.727
DT	364	0.749	0.811	0.788	0.553	0.701	0.724
DT	365	0.778	0.825	0.808	0.595	0.725	0.750
DT	366	0.760	0.816	0.795	0.569	0.710	0.734
DT	367	0.733	0.816	0.785	0.545	0.703	0.718
DT	368	0.718	0.804	0.772	0.518	0.685	0.701
DT	369	0.756	0.829	0.802	0.580	0.724	0.739
DT	370	0.769	0.814	0.797	0.574	0.709	0.738

KNN	1	0.744	0.734	0.738	0.465	0.624	0.679
KNN	2	0.802	0.790	0.794	0.578	0.693	0.744
KNN	3	0.826	0.789	0.803	0.599	0.698	0.757
KNN	4	0.824	0.797	0.807	0.605	0.706	0.760
KNN	5	0.824	0.786	0.800	0.594	0.695	0.754
KNN	6	0.830	0.784	0.801	0.597	0.694	0.756
KNN	7	0.806	0.793	0.798	0.585	0.697	0.748
KNN	8	0.839	0.791	0.809	0.614	0.704	0.766
KNN	9	0.839	0.801	0.815	0.623	0.713	0.771
KNN	10	0.868	0.797	0.823	0.645	0.716	0.785
KNN	11	0.872	0.794	0.823	0.647	0.715	0.786
KNN	12	0.844	0.815	0.826	0.643	0.730	0.782
KNN	13	0.872	0.787	0.819	0.640	0.708	0.782
KNN	14	0.859	0.789	0.815	0.629	0.707	0.775
KNN	15	0.835	0.785	0.803	0.603	0.697	0.760
KNN	16	0.863	0.786	0.815	0.630	0.705	0.776
KNN	17	0.881	0.793	0.826	0.654	0.716	0.790
KNN	18	0.861	0.778	0.809	0.620	0.697	0.770
KNN	19	0.846	0.778	0.803	0.606	0.693	0.762
KNN	20	0.872	0.765	0.805	0.617	0.688	0.769
KNN	21	0.857	0.760	0.796	0.597	0.679	0.758
KNN	22	0.850	0.772	0.801	0.603	0.688	0.761
KNN	23	0.852	0.763	0.796	0.596	0.680	0.757
KNN	24	0.857	0.769	0.802	0.607	0.687	0.763
KNN	25	0.850	0.789	0.812	0.621	0.704	0.770

KNN	26	0.855	0.778	0.807	0.614	0.695	0.767
KNN	27	0.852	0.781	0.808	0.615	0.697	0.767
KNN	28	0.839	0.804	0.817	0.627	0.718	0.774
KNN	29	0.855	0.785	0.811	0.621	0.702	0.771
KNN	30	0.861	0.782	0.812	0.624	0.701	0.773
KNN	31	0.828	0.794	0.807	0.606	0.704	0.761
KNN	32	0.872	0.785	0.817	0.637	0.706	0.780
KNN	33	0.852	0.789	0.812	0.623	0.705	0.772
KNN	34	0.861	0.787	0.815	0.630	0.706	0.776
KNN	35	0.855	0.791	0.815	0.628	0.708	0.774
KNN	36	0.841	0.778	0.802	0.602	0.692	0.759
KNN	37	0.888	0.780	0.820	0.646	0.705	0.786
KNN	38	0.837	0.786	0.805	0.606	0.699	0.762
KNN	39	0.863	0.776	0.808	0.620	0.695	0.770
KNN	40	0.846	0.789	0.810	0.617	0.703	0.768
KNN	41	0.866	0.774	0.808	0.620	0.694	0.771
KNN	42	0.837	0.790	0.808	0.610	0.702	0.764
KNN	43	0.857	0.784	0.811	0.622	0.701	0.771
KNN	44	0.870	0.798	0.825	0.649	0.718	0.787
KNN	45	0.857	0.789	0.814	0.627	0.706	0.774
KNN	46	0.868	0.803	0.827	0.652	0.723	0.789
KNN	47	0.879	0.784	0.819	0.642	0.706	0.783
KNN	48	0.855	0.790	0.814	0.626	0.707	0.774
KNN	49	0.888	0.798	0.831	0.665	0.722	0.796
KNN	50	0.866	0.802	0.826	0.649	0.721	0.787

KNN	51	0.859	0.793	0.817	0.633	0.710	0.778
KNN	52	0.877	0.803	0.830	0.660	0.725	0.794
KNN	53	0.855	0.798	0.819	0.635	0.715	0.778
KNN	54	0.852	0.789	0.812	0.623	0.705	0.772
KNN	55	0.872	0.795	0.824	0.648	0.716	0.786
KNN	56	0.877	0.778	0.815	0.635	0.701	0.779
KNN	57	0.879	0.771	0.811	0.629	0.694	0.776
KNN	58	0.881	0.773	0.813	0.633	0.697	0.778
KNN	59	0.861	0.773	0.806	0.615	0.692	0.767
KNN	60	0.885	0.774	0.816	0.639	0.699	0.781
KNN	61	0.866	0.784	0.814	0.630	0.703	0.776
KNN	62	0.861	0.778	0.809	0.620	0.697	0.770
KNN	63	0.881	0.774	0.814	0.635	0.698	0.779
KNN	64	0.866	0.782	0.813	0.628	0.702	0.775
KNN	65	0.859	0.785	0.812	0.625	0.703	0.773
KNN	66	0.881	0.774	0.814	0.635	0.698	0.779
KNN	67	0.879	0.759	0.803	0.617	0.683	0.769
KNN	68	0.811	0.761	0.780	0.556	0.668	0.732
KNN	69	0.826	0.759	0.784	0.567	0.670	0.740
KNN	70	0.826	0.747	0.776	0.555	0.659	0.733
KNN	71	0.815	0.754	0.776	0.552	0.662	0.731
KNN	72	0.826	0.743	0.774	0.551	0.656	0.731
KNN	73	0.841	0.748	0.783	0.571	0.664	0.742
KNN	74	0.833	0.744	0.777	0.559	0.659	0.735
KNN	75	0.848	0.750	0.786	0.579	0.667	0.747

KNN	76	0.844	0.750	0.785	0.574	0.666	0.744
KNN	77	0.837	0.739	0.776	0.558	0.655	0.735
KNN	78	0.817	0.754	0.777	0.554	0.663	0.732
KNN	79	0.806	0.742	0.766	0.531	0.649	0.719
KNN	80	0.813	0.748	0.772	0.544	0.657	0.726
KNN	81	0.784	0.746	0.760	0.515	0.646	0.708
KNN	82	0.830	0.752	0.781	0.565	0.665	0.738
KNN	83	0.830	0.747	0.778	0.559	0.660	0.736
KNN	84	0.811	0.751	0.773	0.545	0.658	0.727
KNN	85	0.855	0.734	0.779	0.569	0.655	0.742
KNN	86	0.826	0.741	0.772	0.549	0.653	0.730
KNN	87	0.848	0.742	0.781	0.571	0.660	0.743
KNN	88	0.833	0.744	0.777	0.559	0.659	0.735
KNN	89	0.822	0.751	0.777	0.555	0.661	0.733
KNN	90	0.848	0.743	0.782	0.572	0.662	0.743
KNN	91	0.819	0.744	0.772	0.546	0.655	0.728
KNN	92	0.839	0.738	0.776	0.558	0.655	0.736
KNN	93	0.844	0.746	0.782	0.570	0.663	0.742
KNN	94	0.830	0.728	0.766	0.540	0.643	0.725
KNN	95	0.828	0.747	0.777	0.557	0.660	0.734
KNN	96	0.841	0.737	0.776	0.559	0.654	0.736
KNN	97	0.830	0.733	0.769	0.545	0.648	0.728
KNN	98	0.848	0.735	0.777	0.564	0.655	0.739
KNN	99	0.817	0.728	0.761	0.527	0.640	0.718
KNN	100	0.839	0.729	0.770	0.549	0.647	0.731

KNN	101	0.833	0.731	0.769	0.546	0.647	0.728
KNN	102	0.830	0.717	0.759	0.529	0.635	0.719
KNN	103	0.848	0.724	0.770	0.553	0.645	0.733
KNN	104	0.846	0.742	0.781	0.569	0.660	0.741
KNN	105	0.830	0.729	0.767	0.541	0.644	0.726
KNN	106	0.837	0.728	0.768	0.546	0.645	0.729
KNN	107	0.844	0.728	0.771	0.552	0.647	0.732
KNN	108	0.828	0.750	0.779	0.560	0.662	0.736
KNN	109	0.815	0.731	0.762	0.529	0.642	0.718
KNN	110	0.828	0.738	0.771	0.548	0.652	0.729
KNN	111	0.841	0.737	0.776	0.559	0.654	0.736
KNN	112	0.833	0.742	0.776	0.556	0.656	0.734
KNN	113	0.859	0.711	0.766	0.551	0.637	0.732
KNN	114	0.828	0.707	0.752	0.517	0.626	0.713
KNN	115	0.844	0.707	0.758	0.532	0.630	0.721
KNN	116	0.830	0.699	0.748	0.512	0.620	0.710
KNN	117	0.837	0.720	0.763	0.538	0.639	0.725
KNN	118	0.830	0.709	0.754	0.522	0.628	0.715
KNN	119	0.806	0.698	0.738	0.487	0.612	0.696
KNN	120	0.808	0.701	0.741	0.493	0.616	0.699
KNN	121	0.846	0.699	0.753	0.526	0.624	0.718
KNN	122	0.844	0.701	0.754	0.527	0.626	0.719
KNN	123	0.837	0.701	0.752	0.520	0.624	0.715
KNN	124	0.830	0.709	0.754	0.522	0.628	0.715
KNN	125	0.828	0.718	0.759	0.529	0.635	0.719

KNN	126	0.837	0.707	0.755	0.526	0.628	0.718
KNN	127	0.833	0.712	0.757	0.526	0.631	0.718
KNN	128	0.824	0.690	0.740	0.496	0.611	0.702
KNN	129	0.833	0.711	0.756	0.525	0.630	0.717
KNN	130	0.839	0.712	0.759	0.533	0.633	0.722
KNN	131	0.848	0.703	0.757	0.532	0.628	0.722
KNN	132	0.833	0.696	0.747	0.511	0.619	0.710
KNN	133	0.817	0.700	0.744	0.500	0.617	0.703
KNN	134	0.824	0.720	0.758	0.526	0.635	0.717
KNN	135	0.837	0.700	0.751	0.519	0.623	0.714
KNN	136	0.841	0.696	0.750	0.520	0.621	0.715
KNN	137	0.830	0.707	0.753	0.519	0.626	0.714
KNN	138	0.852	0.699	0.756	0.533	0.626	0.722
KNN	139	0.826	0.714	0.756	0.523	0.631	0.716
KNN	140	0.833	0.718	0.761	0.533	0.636	0.721
KNN	141	0.822	0.711	0.752	0.515	0.627	0.711
KNN	142	0.850	0.722	0.770	0.553	0.644	0.733
KNN	143	0.844	0.695	0.750	0.521	0.621	0.715
KNN	144	0.813	0.721	0.755	0.516	0.633	0.712
KNN	145	0.822	0.713	0.753	0.517	0.629	0.713
KNN	146	0.817	0.704	0.746	0.504	0.620	0.705
KNN	147	0.826	0.709	0.753	0.517	0.627	0.713
KNN	148	0.808	0.707	0.744	0.498	0.620	0.702
KNN	149	0.797	0.705	0.740	0.486	0.616	0.695
KNN	150	0.824	0.705	0.749	0.512	0.623	0.710

KNN	151	0.815	0.716	0.753	0.513	0.629	0.710
KNN	152	0.846	0.700	0.754	0.528	0.625	0.719
KNN	153	0.826	0.703	0.749	0.511	0.622	0.710
KNN	154	0.837	0.709	0.757	0.528	0.630	0.719
KNN	155	0.848	0.687	0.747	0.517	0.616	0.714
KNN	156	0.819	0.718	0.756	0.520	0.633	0.714
KNN	157	0.850	0.703	0.758	0.534	0.629	0.723
KNN	158	0.850	0.699	0.755	0.531	0.626	0.721
KNN	159	0.857	0.703	0.760	0.541	0.630	0.726
KNN	160	0.819	0.724	0.759	0.525	0.637	0.717
KNN	161	0.828	0.708	0.753	0.518	0.627	0.713
KNN	162	0.800	0.721	0.750	0.504	0.629	0.704
KNN	163	0.841	0.708	0.758	0.531	0.630	0.721
KNN	164	0.808	0.708	0.745	0.499	0.621	0.702
KNN	165	0.804	0.704	0.741	0.491	0.617	0.698
KNN	166	0.830	0.716	0.758	0.528	0.634	0.719
KNN	167	0.833	0.716	0.759	0.530	0.634	0.720
KNN	168	0.835	0.705	0.753	0.522	0.626	0.716
KNN	169	0.819	0.722	0.758	0.524	0.636	0.716
KNN	170	0.806	0.714	0.749	0.504	0.626	0.705
KNN	171	0.811	0.708	0.746	0.501	0.622	0.704
KNN	172	0.833	0.703	0.751	0.517	0.624	0.713
KNN	173	0.841	0.713	0.761	0.536	0.635	0.723
KNN	174	0.830	0.708	0.753	0.520	0.627	0.715
KNN	175	0.830	0.712	0.756	0.524	0.630	0.717

KNN	176	0.813	0.692	0.737	0.488	0.610	0.697
KNN	177	0.826	0.687	0.739	0.496	0.610	0.702
KNN	178	0.797	0.694	0.732	0.475	0.606	0.689
KNN	179	0.841	0.675	0.737	0.500	0.605	0.704
KNN	180	0.813	0.688	0.735	0.484	0.607	0.695
KNN	181	0.815	0.688	0.735	0.487	0.608	0.696
KNN	182	0.817	0.692	0.739	0.492	0.611	0.699
KNN	183	0.830	0.687	0.740	0.500	0.611	0.704
KNN	184	0.824	0.695	0.743	0.501	0.615	0.704
KNN	185	0.830	0.681	0.736	0.494	0.606	0.701
KNN	186	0.811	0.690	0.735	0.484	0.607	0.694
KNN	187	0.808	0.692	0.735	0.484	0.609	0.694
KNN	188	0.826	0.682	0.735	0.491	0.606	0.699
KNN	189	0.822	0.677	0.731	0.482	0.601	0.694
KNN	190	0.795	0.687	0.727	0.466	0.601	0.684
KNN	191	0.800	0.687	0.729	0.470	0.602	0.687
KNN	192	0.828	0.686	0.739	0.497	0.609	0.702
KNN	193	0.830	0.690	0.742	0.503	0.613	0.705
KNN	194	0.828	0.699	0.747	0.509	0.619	0.709
KNN	195	0.817	0.682	0.732	0.482	0.603	0.694
KNN	196	0.839	0.677	0.737	0.499	0.606	0.704
KNN	197	0.804	0.691	0.733	0.478	0.606	0.691
KNN	198	0.806	0.684	0.730	0.474	0.602	0.689
KNN	199	0.824	0.678	0.732	0.485	0.602	0.696
KNN	200	0.806	0.694	0.735	0.483	0.609	0.694

KNN	201	0.828	0.674	0.731	0.486	0.601	0.696
KNN	202	0.852	0.678	0.743	0.513	0.610	0.711
KNN	203	0.837	0.671	0.733	0.492	0.601	0.700
KNN	204	0.848	0.678	0.741	0.509	0.609	0.709
KNN	205	0.797	0.674	0.720	0.456	0.592	0.679
KNN	206	0.835	0.670	0.731	0.488	0.600	0.698
KNN	207	0.804	0.675	0.723	0.463	0.594	0.684
KNN	208	0.822	0.687	0.737	0.492	0.608	0.699
KNN	209	0.828	0.673	0.731	0.484	0.600	0.696
KNN	210	0.824	0.666	0.725	0.474	0.594	0.690
KNN	211	0.841	0.669	0.733	0.494	0.601	0.701
KNN	212	0.808	0.681	0.728	0.473	0.600	0.689
KNN	213	0.837	0.686	0.742	0.505	0.612	0.707
KNN	214	0.824	0.674	0.730	0.481	0.599	0.694
KNN	215	0.828	0.665	0.726	0.477	0.594	0.692
KNN	216	0.850	0.669	0.736	0.502	0.603	0.706
KNN	217	0.826	0.683	0.736	0.492	0.607	0.700
KNN	218	0.824	0.687	0.738	0.494	0.609	0.700
KNN	219	0.852	0.661	0.732	0.497	0.598	0.703
KNN	220	0.804	0.677	0.724	0.465	0.595	0.684
KNN	221	0.808	0.670	0.722	0.463	0.592	0.683
KNN	222	0.802	0.694	0.734	0.479	0.608	0.691
KNN	223	0.830	0.670	0.730	0.484	0.598	0.696
KNN	224	0.828	0.666	0.726	0.478	0.595	0.692
KNN	225	0.800	0.690	0.731	0.473	0.604	0.688

KNN	226	0.835	0.687	0.742	0.505	0.612	0.706
KNN	227	0.804	0.688	0.731	0.476	0.604	0.690
KNN	228	0.813	0.677	0.727	0.473	0.598	0.689
KNN	229	0.828	0.688	0.740	0.499	0.611	0.703
KNN	230	0.824	0.687	0.738	0.494	0.609	0.700
KNN	231	0.839	0.691	0.746	0.513	0.617	0.711
KNN	232	0.826	0.674	0.731	0.483	0.600	0.695
KNN	233	0.824	0.681	0.734	0.488	0.604	0.697
KNN	234	0.828	0.694	0.744	0.504	0.615	0.706
KNN	235	0.786	0.701	0.733	0.472	0.609	0.687
KNN	236	0.797	0.698	0.735	0.479	0.609	0.691
KNN	237	0.804	0.698	0.737	0.485	0.611	0.695
KNN	238	0.800	0.682	0.726	0.465	0.598	0.684
KNN	239	0.786	0.696	0.730	0.467	0.605	0.684
KNN	240	0.800	0.679	0.724	0.463	0.596	0.683
KNN	241	0.786	0.683	0.722	0.454	0.595	0.677
KNN	242	0.797	0.681	0.724	0.462	0.596	0.682
KNN	243	0.778	0.670	0.710	0.433	0.583	0.666
KNN	244	0.782	0.683	0.720	0.450	0.594	0.675
KNN	245	0.795	0.683	0.725	0.462	0.598	0.682
KNN	246	0.789	0.688	0.726	0.461	0.600	0.681
KNN	247	0.773	0.683	0.717	0.441	0.591	0.670
KNN	248	0.771	0.679	0.713	0.435	0.587	0.667
KNN	249	0.775	0.695	0.725	0.455	0.601	0.677
KNN	250	0.808	0.681	0.728	0.473	0.600	0.689

KNN	251	0.815	0.673	0.726	0.472	0.596	0.688
KNN	252	0.778	0.694	0.725	0.456	0.600	0.678
KNN	253	0.767	0.694	0.721	0.445	0.597	0.671
KNN	254	0.791	0.678	0.720	0.453	0.592	0.677
KNN	255	0.786	0.690	0.726	0.460	0.600	0.681
KNN	256	0.775	0.687	0.720	0.447	0.595	0.673
KNN	257	0.775	0.678	0.714	0.438	0.588	0.669
KNN	258	0.786	0.682	0.721	0.453	0.594	0.677
KNN	259	0.762	0.687	0.715	0.435	0.590	0.665
KNN	260	0.784	0.687	0.723	0.456	0.597	0.678
KNN	261	0.764	0.698	0.722	0.447	0.599	0.672
KNN	262	0.804	0.687	0.731	0.475	0.603	0.689
KNN	263	0.808	0.664	0.717	0.456	0.587	0.680
KNN	264	0.771	0.665	0.704	0.421	0.577	0.660
KNN	265	0.780	0.673	0.713	0.437	0.585	0.669
KNN	266	0.824	0.668	0.726	0.475	0.595	0.691
KNN	267	0.806	0.675	0.724	0.465	0.595	0.685
KNN	268	0.824	0.657	0.719	0.465	0.587	0.686
KNN	269	0.793	0.671	0.717	0.449	0.588	0.675
KNN	270	0.782	0.664	0.708	0.431	0.579	0.665
KNN	271	0.795	0.679	0.722	0.459	0.595	0.680
KNN	272	0.802	0.673	0.721	0.459	0.592	0.681
KNN	273	0.780	0.677	0.715	0.441	0.588	0.670
KNN	274	0.784	0.660	0.706	0.429	0.577	0.665
KNN	275	0.786	0.671	0.714	0.442	0.586	0.672

KNN	276	0.791	0.681	0.722	0.456	0.594	0.679
KNN	277	0.749	0.675	0.703	0.410	0.577	0.652
KNN	278	0.782	0.657	0.704	0.424	0.574	0.662
KNN	279	0.795	0.675	0.720	0.455	0.592	0.679
KNN	280	0.764	0.671	0.706	0.421	0.579	0.659
KNN	281	0.791	0.677	0.719	0.452	0.591	0.677
KNN	282	0.786	0.665	0.710	0.436	0.581	0.669
KNN	283	0.791	0.662	0.710	0.438	0.581	0.670
KNN	284	0.764	0.673	0.707	0.423	0.580	0.660
KNN	285	0.780	0.670	0.711	0.435	0.583	0.667
KNN	286	0.782	0.670	0.712	0.437	0.584	0.669
KNN	287	0.775	0.668	0.708	0.428	0.580	0.664
KNN	288	0.778	0.664	0.706	0.426	0.578	0.663
KNN	289	0.760	0.665	0.700	0.411	0.573	0.653
KNN	290	0.778	0.660	0.704	0.423	0.575	0.661
KNN	291	0.760	0.668	0.702	0.413	0.575	0.655
KNN	292	0.815	0.653	0.713	0.453	0.582	0.679
KNN	293	0.762	0.679	0.710	0.427	0.584	0.662
KNN	294	0.780	0.666	0.708	0.431	0.580	0.665
KNN	295	0.786	0.671	0.714	0.442	0.586	0.672
KNN	296	0.756	0.679	0.708	0.421	0.582	0.658
KNN	297	0.767	0.661	0.700	0.413	0.572	0.655
KNN	298	0.786	0.687	0.724	0.458	0.598	0.679
KNN	299	0.808	0.668	0.720	0.460	0.590	0.682
KNN	300	0.800	0.666	0.716	0.450	0.586	0.677

KNN	301	0.782	0.675	0.715	0.442	0.588	0.671
KNN	302	0.780	0.669	0.710	0.434	0.582	0.667
KNN	303	0.764	0.673	0.707	0.423	0.580	0.660
KNN	304	0.786	0.674	0.716	0.445	0.588	0.673
KNN	305	0.800	0.679	0.724	0.463	0.596	0.683
KNN	306	0.775	0.670	0.709	0.431	0.582	0.665
KNN	307	0.786	0.674	0.716	0.445	0.588	0.673
KNN	308	0.771	0.666	0.705	0.423	0.578	0.660
KNN	309	0.786	0.669	0.713	0.440	0.584	0.670
KNN	310	0.802	0.673	0.721	0.459	0.592	0.681
KNN	311	0.769	0.671	0.708	0.426	0.581	0.662
KNN	312	0.762	0.677	0.708	0.424	0.582	0.660
KNN	313	0.804	0.683	0.728	0.471	0.600	0.687
KNN	314	0.802	0.687	0.730	0.473	0.603	0.688
KNN	315	0.815	0.684	0.733	0.483	0.605	0.694
KNN	316	0.778	0.703	0.731	0.465	0.608	0.682
KNN	317	0.764	0.678	0.710	0.428	0.584	0.662
KNN	318	0.802	0.673	0.721	0.459	0.592	0.681
KNN	319	0.789	0.682	0.722	0.455	0.595	0.678
KNN	320	0.773	0.679	0.714	0.437	0.588	0.668
KNN	321	0.791	0.687	0.726	0.462	0.599	0.682
KNN	322	0.782	0.678	0.717	0.445	0.590	0.672
KNN	323	0.817	0.669	0.724	0.470	0.594	0.688
KNN	324	0.791	0.678	0.720	0.453	0.592	0.677
KNN	325	0.784	0.687	0.723	0.456	0.597	0.678

KNN	326	0.784	0.668	0.711	0.437	0.583	0.669
KNN	327	0.791	0.679	0.721	0.454	0.593	0.678
KNN	328	0.778	0.666	0.708	0.429	0.580	0.664
KNN	329	0.780	0.674	0.713	0.439	0.586	0.669
KNN	330	0.793	0.670	0.716	0.448	0.587	0.675
KNN	331	0.769	0.674	0.709	0.428	0.583	0.663
KNN	332	0.802	0.678	0.724	0.464	0.596	0.684
KNN	333	0.789	0.660	0.708	0.433	0.578	0.667
KNN	334	0.764	0.690	0.717	0.439	0.593	0.668
KNN	335	0.784	0.683	0.721	0.452	0.594	0.676
KNN	336	0.769	0.679	0.713	0.433	0.587	0.665
KNN	337	0.767	0.668	0.704	0.420	0.577	0.658
KNN	338	0.773	0.658	0.701	0.417	0.573	0.658
KNN	339	0.762	0.670	0.704	0.418	0.578	0.657
KNN	340	0.793	0.664	0.712	0.441	0.583	0.672
KNN	341	0.782	0.662	0.707	0.429	0.578	0.665
KNN	342	0.786	0.660	0.707	0.431	0.578	0.666
KNN	343	0.780	0.664	0.707	0.429	0.578	0.664
KNN	344	0.793	0.671	0.717	0.449	0.588	0.675
KNN	345	0.769	0.673	0.708	0.427	0.582	0.662
KNN	346	0.773	0.668	0.707	0.426	0.579	0.662
KNN	347	0.786	0.669	0.713	0.440	0.584	0.670
KNN	348	0.789	0.692	0.728	0.465	0.603	0.683
KNN	349	0.775	0.700	0.728	0.460	0.605	0.680
KNN	350	0.797	0.678	0.722	0.459	0.594	0.681

KNN	351	0.802	0.678	0.724	0.464	0.596	0.684
KNN	352	0.817	0.679	0.731	0.480	0.601	0.693
KNN	353	0.789	0.675	0.717	0.448	0.590	0.675
KNN	354	0.789	0.687	0.725	0.460	0.599	0.681
KNN	355	0.802	0.671	0.720	0.457	0.591	0.680
KNN	356	0.800	0.686	0.728	0.469	0.601	0.686
KNN	357	0.793	0.679	0.722	0.456	0.594	0.679
KNN	358	0.797	0.670	0.717	0.452	0.589	0.677
KNN	359	0.793	0.677	0.720	0.454	0.592	0.678
KNN	360	0.789	0.684	0.723	0.457	0.597	0.679
KNN	361	0.797	0.687	0.728	0.468	0.601	0.686
KNN	362	0.758	0.700	0.722	0.443	0.599	0.669
KNN	363	0.773	0.695	0.724	0.453	0.600	0.676
KNN	364	0.784	0.694	0.727	0.462	0.602	0.681
KNN	365	0.802	0.695	0.735	0.480	0.609	0.692
KNN	366	0.806	0.705	0.743	0.495	0.618	0.700
KNN	367	0.778	0.703	0.731	0.465	0.608	0.682
KNN	368	0.786	0.699	0.731	0.469	0.607	0.685
KNN	369	0.800	0.692	0.732	0.476	0.606	0.689
KNN	370	0.793	0.699	0.734	0.476	0.609	0.689
RF	1	0.729	0.743	0.738	0.461	0.627	0.674
RF	2	0.824	0.816	0.819	0.626	0.726	0.772
RF	3	0.815	0.833	0.826	0.637	0.743	0.777
RF	4	0.802	0.827	0.817	0.618	0.732	0.766
RF	5	0.813	0.842	0.831	0.646	0.753	0.782

RF	6	0.822	0.841	0.834	0.652	0.754	0.786
RF	7	0.824	0.840	0.834	0.653	0.753	0.787
RF	8	0.833	0.841	0.838	0.662	0.756	0.792
RF	9	0.841	0.853	0.848	0.683	0.772	0.805
RF	10	0.830	0.854	0.845	0.675	0.771	0.800
RF	11	0.828	0.849	0.841	0.667	0.764	0.795
RF	12	0.844	0.846	0.845	0.678	0.764	0.802
RF	13	0.848	0.847	0.848	0.683	0.767	0.805
RF	14	0.844	0.862	0.855	0.696	0.783	0.812
RF	15	0.850	0.850	0.850	0.688	0.770	0.808
RF	16	0.839	0.847	0.844	0.675	0.765	0.800
RF	17	0.848	0.855	0.853	0.692	0.776	0.811
RF	18	0.841	0.855	0.850	0.686	0.775	0.807
RF	19	0.844	0.853	0.849	0.685	0.772	0.806
RF	20	0.846	0.853	0.850	0.687	0.773	0.808
RF	21	0.841	0.846	0.844	0.676	0.764	0.801
RF	22	0.839	0.850	0.846	0.678	0.768	0.802
RF	23	0.835	0.860	0.851	0.686	0.780	0.806
RF	24	0.846	0.854	0.851	0.689	0.774	0.808
RF	25	0.846	0.854	0.851	0.689	0.774	0.808
RF	26	0.846	0.846	0.846	0.680	0.765	0.803
RF	27	0.850	0.855	0.853	0.694	0.777	0.812
RF	28	0.848	0.853	0.851	0.689	0.773	0.809
RF	29	0.839	0.850	0.846	0.678	0.768	0.802
RF	30	0.852	0.853	0.853	0.693	0.774	0.811

RF	31	0.846	0.849	0.848	0.683	0.768	0.805
RF	32	0.846	0.857	0.853	0.692	0.777	0.810
RF	33	0.830	0.858	0.848	0.680	0.776	0.802
RF	34	0.830	0.860	0.849	0.683	0.779	0.804
RF	35	0.835	0.858	0.849	0.683	0.777	0.805
RF	36	0.850	0.857	0.854	0.696	0.778	0.813
RF	37	0.830	0.847	0.841	0.667	0.763	0.795
RF	38	0.837	0.855	0.848	0.682	0.774	0.804
RF	39	0.837	0.867	0.856	0.696	0.788	0.812
RF	40	0.835	0.853	0.846	0.677	0.770	0.801
RF	41	0.841	0.870	0.859	0.703	0.793	0.816
RF	42	0.841	0.866	0.857	0.698	0.788	0.814
RF	43	0.822	0.858	0.844	0.672	0.774	0.797
RF	44	0.839	0.864	0.855	0.695	0.786	0.812
RF	45	0.837	0.859	0.851	0.687	0.779	0.807
RF	46	0.844	0.859	0.853	0.693	0.780	0.811
RF	47	0.857	0.847	0.851	0.691	0.769	0.810
RF	48	0.857	0.857	0.857	0.701	0.780	0.816
RF	49	0.837	0.849	0.844	0.675	0.766	0.800
RF	50	0.852	0.857	0.855	0.697	0.779	0.814
RF	51	0.848	0.854	0.852	0.691	0.775	0.810
RF	52	0.857	0.847	0.851	0.691	0.769	0.810
RF	53	0.846	0.858	0.853	0.693	0.779	0.811
RF	54	0.846	0.845	0.845	0.678	0.763	0.803
RF	55	0.859	0.851	0.854	0.697	0.774	0.814

RF	56	0.841	0.853	0.848	0.683	0.772	0.805
RF	57	0.846	0.851	0.849	0.686	0.771	0.807
RF	58	0.855	0.855	0.855	0.698	0.778	0.814
RF	59	0.837	0.847	0.844	0.673	0.765	0.799
RF	60	0.839	0.860	0.853	0.690	0.781	0.809
RF	61	0.846	0.850	0.848	0.684	0.770	0.806
RF	62	0.855	0.847	0.850	0.689	0.768	0.809
RF	63	0.844	0.847	0.846	0.679	0.766	0.803
RF	64	0.850	0.847	0.848	0.685	0.767	0.807
RF	65	0.839	0.853	0.848	0.681	0.771	0.804
RF	66	0.846	0.854	0.851	0.689	0.774	0.808
RF	67	0.848	0.860	0.856	0.698	0.783	0.814
RF	68	0.837	0.864	0.854	0.693	0.785	0.810
RF	69	0.848	0.858	0.854	0.695	0.779	0.812
RF	70	0.844	0.855	0.851	0.688	0.775	0.808
RF	71	0.857	0.858	0.857	0.703	0.781	0.817
RF	72	0.841	0.866	0.857	0.698	0.788	0.814
RF	73	0.844	0.858	0.853	0.691	0.778	0.810
RF	74	0.841	0.862	0.854	0.694	0.783	0.811
RF	75	0.841	0.862	0.854	0.694	0.783	0.811
RF	76	0.839	0.862	0.853	0.692	0.782	0.810
RF	77	0.844	0.867	0.858	0.702	0.790	0.816
RF	78	0.850	0.860	0.857	0.700	0.783	0.815
RF	79	0.848	0.863	0.857	0.701	0.786	0.816
RF	80	0.844	0.864	0.857	0.699	0.786	0.814

RF	81	0.852	0.864	0.860	0.707	0.788	0.819
RF	82	0.848	0.868	0.861	0.707	0.792	0.819
RF	83	0.852	0.859	0.857	0.700	0.782	0.816
RF	84	0.852	0.855	0.854	0.696	0.777	0.813
RF	85	0.837	0.871	0.858	0.701	0.793	0.815
RF	86	0.850	0.855	0.853	0.694	0.777	0.812
RF	87	0.852	0.860	0.857	0.702	0.783	0.816
RF	88	0.857	0.862	0.860	0.707	0.786	0.820
RF	89	0.848	0.857	0.853	0.694	0.778	0.811
RF	90	0.852	0.863	0.859	0.705	0.787	0.818
RF	91	0.848	0.862	0.857	0.700	0.784	0.815
RF	92	0.848	0.860	0.856	0.698	0.783	0.814
RF	93	0.841	0.863	0.855	0.695	0.784	0.812
RF	94	0.841	0.863	0.855	0.695	0.784	0.812
RF	95	0.855	0.867	0.862	0.712	0.792	0.822
RF	96	0.848	0.858	0.854	0.695	0.779	0.812
RF	97	0.846	0.863	0.857	0.699	0.785	0.814
RF	98	0.850	0.863	0.858	0.703	0.786	0.817
RF	99	0.850	0.862	0.857	0.702	0.785	0.816
RF	100	0.848	0.857	0.853	0.694	0.778	0.811
RF	101	0.833	0.866	0.853	0.691	0.786	0.809
RF	102	0.835	0.858	0.849	0.683	0.777	0.805
RF	103	0.841	0.859	0.853	0.691	0.780	0.809
RF	104	0.837	0.864	0.854	0.693	0.785	0.810
RF	105	0.833	0.864	0.853	0.689	0.784	0.808

RF	106	0.841	0.867	0.857	0.700	0.789	0.814
RF	107	0.835	0.858	0.849	0.683	0.777	0.805
RF	108	0.841	0.864	0.856	0.697	0.786	0.813
RF	109	0.844	0.860	0.854	0.694	0.782	0.811
RF	110	0.852	0.874	0.866	0.717	0.800	0.825
RF	111	0.839	0.871	0.859	0.703	0.794	0.816
RF	112	0.859	0.858	0.858	0.705	0.782	0.818
RF	113	0.848	0.872	0.863	0.712	0.797	0.822
RF	114	0.844	0.868	0.859	0.703	0.791	0.817
RF	115	0.841	0.872	0.861	0.706	0.796	0.818
RF	116	0.857	0.870	0.865	0.717	0.796	0.825
RF	117	0.857	0.879	0.871	0.727	0.807	0.831
RF	118	0.866	0.877	0.873	0.733	0.807	0.835
RF	119	0.855	0.871	0.865	0.716	0.797	0.825
RF	120	0.839	0.863	0.854	0.693	0.784	0.811
RF	121	0.852	0.866	0.861	0.708	0.790	0.820
RF	122	0.844	0.876	0.864	0.713	0.801	0.822
RF	123	0.850	0.871	0.863	0.712	0.796	0.822
RF	124	0.857	0.871	0.866	0.718	0.797	0.826
RF	125	0.852	0.872	0.865	0.716	0.798	0.824
RF	126	0.859	0.874	0.868	0.723	0.801	0.829
RF	127	0.866	0.867	0.867	0.721	0.794	0.828
RF	128	0.855	0.870	0.864	0.715	0.795	0.824
RF	129	0.852	0.881	0.871	0.727	0.810	0.830
RF	130	0.852	0.879	0.869	0.723	0.806	0.829

RF	131	0.846	0.872	0.862	0.710	0.797	0.821
RF	132	0.861	0.874	0.869	0.725	0.801	0.830
RF	133	0.844	0.867	0.858	0.702	0.790	0.816
RF	134	0.852	0.870	0.863	0.713	0.795	0.823
RF	135	0.855	0.877	0.869	0.724	0.805	0.829
RF	136	0.841	0.871	0.860	0.705	0.794	0.817
RF	137	0.848	0.872	0.863	0.712	0.797	0.822
RF	138	0.852	0.872	0.865	0.716	0.798	0.824
RF	139	0.861	0.872	0.868	0.723	0.800	0.829
RF	140	0.852	0.872	0.865	0.716	0.798	0.824
RF	141	0.833	0.870	0.856	0.695	0.791	0.811
RF	142	0.837	0.875	0.861	0.705	0.798	0.817
RF	143	0.846	0.870	0.861	0.707	0.793	0.819
RF	144	0.870	0.871	0.871	0.730	0.800	0.833
RF	145	0.855	0.880	0.871	0.727	0.808	0.831
RF	146	0.846	0.875	0.864	0.713	0.800	0.822
RF	147	0.848	0.871	0.862	0.710	0.795	0.821
RF	148	0.855	0.879	0.870	0.725	0.807	0.830
RF	149	0.835	0.871	0.857	0.699	0.793	0.813
RF	150	0.846	0.868	0.860	0.705	0.792	0.818
RF	151	0.839	0.876	0.862	0.709	0.800	0.819
RF	152	0.835	0.864	0.853	0.691	0.785	0.809
RF	153	0.859	0.868	0.865	0.717	0.794	0.825
RF	154	0.852	0.880	0.870	0.725	0.808	0.830
RF	155	0.844	0.881	0.867	0.719	0.808	0.825

RF	156	0.835	0.879	0.862	0.708	0.803	0.819
RF	157	0.844	0.879	0.866	0.716	0.805	0.824
RF	158	0.839	0.867	0.857	0.698	0.789	0.813
RF	159	0.841	0.876	0.863	0.711	0.801	0.821
RF	160	0.870	0.879	0.876	0.739	0.809	0.839
RF	161	0.866	0.870	0.868	0.724	0.797	0.830
RF	162	0.868	0.867	0.867	0.723	0.794	0.829
RF	163	0.855	0.875	0.867	0.721	0.802	0.827
RF	164	0.870	0.879	0.876	0.739	0.809	0.839
RF	165	0.866	0.867	0.867	0.721	0.794	0.828
RF	166	0.859	0.872	0.867	0.721	0.799	0.828
RF	167	0.870	0.872	0.871	0.731	0.801	0.834
RF	168	0.852	0.866	0.861	0.708	0.790	0.820
RF	169	0.857	0.880	0.871	0.729	0.809	0.832
RF	170	0.863	0.877	0.872	0.731	0.807	0.834
RF	171	0.859	0.872	0.867	0.721	0.799	0.828
RF	172	0.859	0.875	0.869	0.725	0.802	0.830
RF	173	0.852	0.876	0.867	0.720	0.803	0.827
RF	174	0.859	0.883	0.874	0.734	0.813	0.835
RF	175	0.844	0.870	0.860	0.705	0.793	0.818
RF	176	0.872	0.875	0.874	0.736	0.805	0.837
RF	177	0.824	0.880	0.859	0.700	0.803	0.813
RF	178	0.848	0.875	0.865	0.715	0.800	0.824
RF	179	0.859	0.879	0.871	0.729	0.807	0.832
RF	180	0.852	0.877	0.868	0.722	0.805	0.828

RF	181	0.833	0.876	0.860	0.703	0.799	0.816
RF	182	0.846	0.881	0.868	0.721	0.808	0.827
RF	183	0.857	0.876	0.869	0.724	0.804	0.829
RF	184	0.863	0.875	0.871	0.728	0.803	0.832
RF	185	0.852	0.880	0.870	0.725	0.808	0.830
RF	186	0.844	0.879	0.866	0.716	0.805	0.824
RF	187	0.852	0.876	0.867	0.720	0.803	0.827
RF	188	0.861	0.868	0.866	0.719	0.795	0.827
RF	189	0.848	0.883	0.870	0.724	0.811	0.829
RF	190	0.857	0.872	0.867	0.720	0.799	0.827
RF	191	0.844	0.877	0.865	0.714	0.803	0.823
RF	192	0.857	0.883	0.873	0.732	0.812	0.834
RF	193	0.852	0.881	0.871	0.727	0.810	0.830
RF	194	0.839	0.884	0.867	0.718	0.811	0.825
RF	195	0.859	0.872	0.867	0.721	0.799	0.828
RF	196	0.866	0.880	0.875	0.737	0.810	0.837
RF	197	0.852	0.875	0.867	0.719	0.801	0.826
RF	198	0.857	0.871	0.866	0.718	0.797	0.826
RF	199	0.855	0.866	0.862	0.710	0.790	0.821
RF	200	0.850	0.871	0.863	0.712	0.796	0.822
RF	201	0.863	0.880	0.874	0.735	0.810	0.836
RF	202	0.852	0.870	0.863	0.713	0.795	0.823
RF	203	0.841	0.875	0.862	0.709	0.799	0.820
RF	204	0.850	0.867	0.861	0.708	0.791	0.820
RF	205	0.844	0.883	0.868	0.721	0.810	0.826

RF	206	0.863	0.872	0.869	0.725	0.800	0.831
RF	207	0.848	0.880	0.868	0.721	0.807	0.827
RF	208	0.857	0.885	0.875	0.735	0.816	0.836
RF	209	0.857	0.885	0.875	0.735	0.816	0.836
RF	210	0.863	0.883	0.876	0.738	0.813	0.838
RF	211	0.852	0.876	0.867	0.720	0.803	0.827
RF	212	0.850	0.879	0.868	0.722	0.806	0.827
RF	213	0.850	0.872	0.864	0.714	0.798	0.823
RF	214	0.850	0.877	0.867	0.720	0.804	0.827
RF	215	0.861	0.888	0.878	0.742	0.820	0.840
RF	216	0.844	0.880	0.867	0.717	0.806	0.825
RF	217	0.852	0.876	0.867	0.720	0.803	0.827
RF	218	0.857	0.876	0.869	0.724	0.804	0.829
RF	219	0.844	0.877	0.865	0.714	0.803	0.823
RF	220	0.872	0.866	0.868	0.726	0.794	0.831
RF	221	0.846	0.875	0.864	0.713	0.800	0.822
RF	222	0.866	0.875	0.871	0.730	0.804	0.834
RF	223	0.852	0.868	0.862	0.711	0.793	0.822
RF	224	0.855	0.877	0.869	0.724	0.805	0.829
RF	225	0.863	0.871	0.868	0.724	0.798	0.830
RF	226	0.855	0.875	0.867	0.721	0.802	0.827
RF	227	0.866	0.874	0.871	0.729	0.802	0.833
RF	228	0.837	0.872	0.859	0.702	0.795	0.815
RF	229	0.859	0.876	0.870	0.726	0.804	0.831
RF	230	0.855	0.885	0.874	0.733	0.815	0.834

RF	231	0.850	0.876	0.867	0.718	0.802	0.826
RF	232	0.855	0.876	0.868	0.722	0.803	0.828
RF	233	0.868	0.874	0.871	0.731	0.802	0.834
RF	234	0.861	0.872	0.868	0.723	0.800	0.829
RF	235	0.841	0.881	0.867	0.717	0.808	0.824
RF	236	0.828	0.885	0.864	0.710	0.810	0.819
RF	237	0.828	0.883	0.862	0.707	0.807	0.817
RF	238	0.833	0.868	0.855	0.694	0.789	0.810
RF	239	0.841	0.871	0.860	0.705	0.794	0.817
RF	240	0.846	0.863	0.857	0.699	0.785	0.814
RF	241	0.848	0.868	0.861	0.707	0.792	0.819
RF	242	0.824	0.875	0.856	0.694	0.796	0.810
RF	243	0.839	0.874	0.861	0.706	0.797	0.818
RF	244	0.837	0.872	0.859	0.702	0.795	0.815
RF	245	0.844	0.874	0.862	0.710	0.798	0.820
RF	246	0.835	0.877	0.862	0.707	0.801	0.818
RF	247	0.850	0.875	0.866	0.717	0.801	0.825
RF	248	0.837	0.872	0.859	0.702	0.795	0.815
RF	249	0.837	0.868	0.857	0.698	0.790	0.813
RF	250	0.839	0.867	0.857	0.698	0.789	0.813
RF	251	0.839	0.874	0.861	0.706	0.797	0.818
RF	252	0.844	0.877	0.865	0.714	0.803	0.823
RF	253	0.841	0.866	0.857	0.698	0.788	0.814
RF	254	0.830	0.876	0.859	0.701	0.799	0.814
RF	255	0.844	0.879	0.866	0.716	0.805	0.824

RF	256	0.844	0.877	0.865	0.714	0.803	0.823
RF	257	0.828	0.880	0.861	0.704	0.803	0.816
RF	258	0.859	0.874	0.868	0.723	0.801	0.829
RF	259	0.835	0.871	0.857	0.699	0.793	0.813
RF	260	0.830	0.871	0.856	0.695	0.792	0.811
RF	261	0.835	0.870	0.857	0.697	0.791	0.812
RF	262	0.835	0.874	0.859	0.702	0.796	0.815
RF	263	0.841	0.872	0.861	0.706	0.796	0.818
RF	264	0.828	0.874	0.857	0.696	0.795	0.811
RF	265	0.835	0.883	0.865	0.713	0.808	0.821
RF	266	0.839	0.872	0.860	0.704	0.795	0.817
RF	267	0.839	0.864	0.855	0.695	0.786	0.812
RF	268	0.828	0.870	0.854	0.691	0.790	0.809
RF	269	0.848	0.876	0.866	0.717	0.802	0.824
RF	270	0.839	0.876	0.862	0.709	0.800	0.819
RF	271	0.835	0.870	0.857	0.697	0.791	0.812
RF	272	0.835	0.884	0.866	0.714	0.810	0.822
RF	273	0.844	0.879	0.866	0.716	0.805	0.824
RF	274	0.841	0.872	0.861	0.706	0.796	0.818
RF	275	0.857	0.870	0.865	0.717	0.796	0.825
RF	276	0.833	0.874	0.858	0.700	0.796	0.814
RF	277	0.837	0.875	0.861	0.705	0.798	0.817
RF	278	0.828	0.877	0.859	0.701	0.800	0.814
RF	279	0.850	0.881	0.870	0.725	0.809	0.829
RF	280	0.835	0.875	0.860	0.703	0.798	0.816

RF	281	0.846	0.884	0.870	0.724	0.812	0.828
RF	282	0.833	0.881	0.863	0.709	0.806	0.819
RF	283	0.828	0.875	0.857	0.698	0.797	0.812
RF	284	0.844	0.872	0.862	0.708	0.796	0.819
RF	285	0.844	0.862	0.855	0.696	0.783	0.812
RF	286	0.839	0.877	0.863	0.710	0.802	0.820
RF	287	0.844	0.868	0.859	0.703	0.791	0.817
RF	288	0.846	0.874	0.863	0.712	0.798	0.821
RF	289	0.846	0.871	0.862	0.708	0.795	0.820
RF	290	0.835	0.876	0.861	0.705	0.800	0.817
RF	291	0.837	0.877	0.862	0.709	0.802	0.819
RF	292	0.848	0.876	0.866	0.717	0.802	0.824
RF	293	0.826	0.877	0.858	0.699	0.800	0.813
RF	294	0.828	0.876	0.858	0.699	0.798	0.813
RF	295	0.859	0.889	0.878	0.742	0.821	0.840
RF	296	0.850	0.875	0.866	0.717	0.801	0.825
RF	297	0.830	0.876	0.859	0.701	0.799	0.814
RF	298	0.839	0.890	0.871	0.726	0.819	0.829
RF	299	0.839	0.876	0.862	0.709	0.800	0.819
RF	300	0.850	0.871	0.863	0.712	0.796	0.822
RF	301	0.855	0.875	0.867	0.721	0.802	0.827
RF	302	0.835	0.881	0.864	0.711	0.806	0.820
RF	303	0.833	0.879	0.862	0.706	0.803	0.817
RF	304	0.833	0.877	0.861	0.705	0.801	0.816
RF	305	0.846	0.871	0.862	0.708	0.795	0.820

RF	306	0.833	0.883	0.864	0.711	0.808	0.820
RF	307	0.844	0.875	0.863	0.711	0.800	0.821
RF	308	0.850	0.871	0.863	0.712	0.796	0.822
RF	309	0.830	0.881	0.862	0.708	0.806	0.818
RF	310	0.844	0.871	0.861	0.706	0.795	0.818
RF	311	0.837	0.867	0.856	0.696	0.788	0.812
RF	312	0.839	0.877	0.863	0.710	0.802	0.820
RF	313	0.835	0.872	0.858	0.700	0.795	0.814
RF	314	0.839	0.879	0.864	0.712	0.804	0.821
RF	315	0.837	0.883	0.866	0.715	0.809	0.823
RF	316	0.839	0.876	0.862	0.709	0.800	0.819
RF	317	0.837	0.872	0.859	0.702	0.795	0.815
RF	318	0.844	0.875	0.863	0.711	0.800	0.821
RF	319	0.844	0.877	0.865	0.714	0.803	0.823
RF	320	0.841	0.871	0.860	0.705	0.794	0.817
RF	321	0.839	0.871	0.859	0.703	0.794	0.816
RF	322	0.830	0.876	0.859	0.701	0.799	0.814
RF	323	0.835	0.875	0.860	0.703	0.798	0.816
RF	324	0.839	0.875	0.862	0.707	0.799	0.818
RF	325	0.841	0.871	0.860	0.705	0.794	0.817
RF	326	0.846	0.880	0.867	0.719	0.807	0.826
RF	327	0.833	0.867	0.854	0.692	0.788	0.809
RF	328	0.833	0.872	0.857	0.698	0.794	0.813
RF	329	0.830	0.870	0.855	0.693	0.790	0.810
RF	330	0.841	0.872	0.861	0.706	0.796	0.818

RF	331	0.833	0.877	0.861	0.705	0.801	0.816
RF	332	0.841	0.880	0.866	0.715	0.806	0.823
RF	333	0.848	0.866	0.859	0.704	0.789	0.817
RF	334	0.835	0.872	0.858	0.700	0.795	0.814
RF	335	0.850	0.874	0.865	0.715	0.799	0.824
RF	336	0.841	0.867	0.857	0.700	0.789	0.814
RF	337	0.826	0.867	0.852	0.686	0.786	0.806
RF	338	0.833	0.866	0.853	0.691	0.786	0.809
RF	339	0.830	0.868	0.854	0.692	0.789	0.809
RF	340	0.844	0.867	0.858	0.702	0.790	0.816
RF	341	0.828	0.864	0.851	0.685	0.783	0.805
RF	342	0.830	0.871	0.856	0.695	0.792	0.811
RF	343	0.839	0.876	0.862	0.709	0.800	0.819
RF	344	0.828	0.874	0.857	0.696	0.795	0.811
RF	345	0.819	0.872	0.853	0.687	0.791	0.805
RF	346	0.839	0.864	0.855	0.695	0.786	0.812
RF	347	0.839	0.871	0.859	0.703	0.794	0.816
RF	348	0.839	0.876	0.862	0.709	0.800	0.819
RF	349	0.839	0.876	0.862	0.709	0.800	0.819
RF	350	0.846	0.870	0.861	0.707	0.793	0.819
RF	351	0.850	0.870	0.862	0.711	0.794	0.821
RF	352	0.839	0.880	0.865	0.714	0.805	0.822
RF	353	0.837	0.872	0.859	0.702	0.795	0.815
RF	354	0.837	0.868	0.857	0.698	0.790	0.813
RF	355	0.844	0.871	0.861	0.706	0.795	0.818

RF	356	0.855	0.872	0.866	0.718	0.798	0.826
RF	357	0.844	0.876	0.864	0.713	0.801	0.822
RF	358	0.846	0.876	0.865	0.715	0.802	0.823
RF	359	0.844	0.876	0.864	0.713	0.801	0.822
RF	360	0.844	0.864	0.857	0.699	0.786	0.814
RF	361	0.848	0.875	0.865	0.715	0.800	0.824
RF	362	0.815	0.870	0.849	0.680	0.787	0.801
RF	363	0.819	0.874	0.853	0.689	0.793	0.806
RF	364	0.800	0.864	0.840	0.660	0.777	0.788
RF	365	0.826	0.864	0.850	0.683	0.783	0.804
RF	366	0.837	0.864	0.854	0.693	0.785	0.810
RF	367	0.819	0.867	0.849	0.681	0.785	0.802
RF	368	0.841	0.881	0.867	0.717	0.808	0.824
RF	369	0.824	0.872	0.854	0.691	0.792	0.808
RF	370	0.841	0.872	0.861	0.706	0.796	0.818
SVM	1	0.764	0.808	0.792	0.564	0.702	0.732
SVM	2	0.786	0.819	0.807	0.595	0.720	0.752
SVM	3	0.786	0.832	0.815	0.610	0.735	0.760
SVM	4	0.800	0.819	0.812	0.607	0.723	0.759
SVM	5	0.742	0.838	0.803	0.579	0.731	0.737
SVM	6	0.769	0.837	0.812	0.601	0.736	0.752
SVM	7	0.756	0.837	0.807	0.589	0.733	0.744
SVM	8	0.760	0.832	0.805	0.587	0.728	0.744
SVM	9	0.775	0.844	0.818	0.614	0.746	0.760
SVM	10	0.775	0.845	0.819	0.616	0.747	0.761

SVM	11	0.771	0.847	0.819	0.615	0.749	0.760
SVM	12	0.747	0.840	0.805	0.584	0.734	0.740
SVM	13	0.769	0.834	0.810	0.598	0.733	0.751
SVM	14	0.786	0.825	0.811	0.603	0.727	0.756
SVM	15	0.784	0.823	0.808	0.598	0.724	0.753
SVM	16	0.804	0.829	0.820	0.623	0.736	0.768
SVM	17	0.775	0.829	0.809	0.598	0.729	0.751
SVM	18	0.760	0.837	0.808	0.593	0.734	0.747
SVM	19	0.797	0.828	0.817	0.616	0.733	0.764
SVM	20	0.795	0.823	0.812	0.608	0.726	0.759
SVM	21	0.800	0.829	0.818	0.619	0.735	0.766
SVM	22	0.806	0.827	0.819	0.622	0.733	0.768
SVM	23	0.797	0.815	0.808	0.601	0.718	0.756
SVM	24	0.791	0.833	0.817	0.616	0.737	0.763
SVM	25	0.811	0.838	0.828	0.639	0.748	0.778
SVM	26	0.804	0.836	0.824	0.631	0.743	0.772
SVM	27	0.811	0.836	0.826	0.636	0.745	0.776
SVM	28	0.797	0.833	0.820	0.622	0.739	0.767
SVM	29	0.802	0.847	0.830	0.642	0.757	0.779
SVM	30	0.806	0.847	0.832	0.646	0.758	0.781
SVM	31	0.797	0.850	0.830	0.641	0.759	0.778
SVM	32	0.791	0.846	0.826	0.631	0.753	0.771
SVM	33	0.806	0.838	0.826	0.635	0.747	0.775
SVM	34	0.813	0.846	0.834	0.650	0.758	0.784
SVM	35	0.808	0.850	0.835	0.651	0.761	0.784

SVM	36	0.806	0.842	0.829	0.640	0.752	0.778
SVM	37	0.800	0.837	0.823	0.628	0.744	0.771
SVM	38	0.795	0.838	0.822	0.626	0.744	0.769
SVM	39	0.797	0.837	0.822	0.626	0.743	0.769
SVM	40	0.791	0.825	0.812	0.607	0.728	0.758
SVM	41	0.797	0.821	0.812	0.608	0.725	0.760
SVM	42	0.828	0.844	0.838	0.661	0.758	0.792
SVM	43	0.822	0.840	0.833	0.651	0.752	0.785
SVM	44	0.817	0.851	0.839	0.660	0.765	0.790
SVM	45	0.813	0.834	0.826	0.637	0.744	0.777
SVM	46	0.822	0.832	0.828	0.642	0.743	0.780
SVM	47	0.822	0.825	0.824	0.634	0.736	0.776
SVM	48	0.819	0.825	0.823	0.632	0.735	0.775
SVM	49	0.824	0.825	0.825	0.636	0.736	0.778
SVM	50	0.824	0.838	0.833	0.651	0.751	0.786
SVM	51	0.828	0.841	0.836	0.658	0.755	0.790
SVM	52	0.833	0.840	0.837	0.660	0.754	0.792
SVM	53	0.833	0.836	0.835	0.656	0.750	0.789
SVM	54	0.837	0.840	0.839	0.664	0.755	0.794
SVM	55	0.830	0.831	0.830	0.648	0.744	0.785
SVM	56	0.811	0.840	0.829	0.641	0.749	0.779
SVM	57	0.808	0.837	0.826	0.636	0.746	0.776
SVM	58	0.815	0.831	0.825	0.634	0.740	0.776
SVM	59	0.808	0.829	0.821	0.627	0.737	0.771
SVM	60	0.819	0.829	0.826	0.637	0.740	0.777

SVM	61	0.824	0.827	0.826	0.638	0.738	0.778
SVM	62	0.826	0.833	0.830	0.647	0.746	0.784
SVM	63	0.830	0.829	0.830	0.647	0.742	0.784
SVM	64	0.830	0.829	0.830	0.647	0.742	0.784
SVM	65	0.828	0.831	0.830	0.646	0.743	0.783
SVM	66	0.835	0.840	0.838	0.662	0.755	0.793
SVM	67	0.839	0.845	0.843	0.672	0.762	0.799
SVM	68	0.848	0.885	0.871	0.727	0.814	0.831
SVM	69	0.870	0.880	0.876	0.740	0.811	0.840
SVM	70	0.868	0.877	0.874	0.735	0.807	0.837
SVM	71	0.863	0.876	0.871	0.730	0.805	0.833
SVM	72	0.866	0.883	0.876	0.740	0.814	0.839
SVM	73	0.868	0.881	0.876	0.740	0.812	0.839
SVM	74	0.868	0.881	0.876	0.740	0.812	0.839
SVM	75	0.868	0.880	0.876	0.738	0.811	0.838
SVM	76	0.859	0.879	0.871	0.729	0.807	0.832
SVM	77	0.861	0.877	0.871	0.730	0.806	0.833
SVM	78	0.863	0.876	0.871	0.730	0.805	0.833
SVM	79	0.861	0.880	0.873	0.733	0.810	0.835
SVM	80	0.859	0.880	0.872	0.731	0.809	0.833
SVM	81	0.868	0.881	0.876	0.740	0.812	0.839
SVM	82	0.868	0.881	0.876	0.740	0.812	0.839
SVM	83	0.872	0.884	0.880	0.747	0.816	0.843
SVM	84	0.872	0.875	0.874	0.736	0.805	0.837
SVM	85	0.872	0.876	0.875	0.738	0.807	0.838

SVM	86	0.868	0.874	0.871	0.731	0.802	0.834
SVM	87	0.866	0.874	0.871	0.729	0.802	0.833
SVM	88	0.866	0.875	0.871	0.730	0.804	0.834
SVM	89	0.870	0.875	0.873	0.734	0.804	0.836
SVM	90	0.868	0.875	0.872	0.732	0.804	0.835
SVM	91	0.863	0.871	0.868	0.724	0.798	0.830
SVM	92	0.861	0.874	0.869	0.725	0.801	0.830
SVM	93	0.859	0.867	0.864	0.715	0.793	0.825
SVM	94	0.870	0.880	0.876	0.740	0.811	0.840
SVM	95	0.868	0.877	0.874	0.735	0.807	0.837
SVM	96	0.870	0.877	0.875	0.737	0.808	0.838
SVM	97	0.868	0.877	0.874	0.735	0.807	0.837
SVM	98	0.863	0.868	0.867	0.721	0.795	0.828
SVM	99	0.861	0.867	0.865	0.717	0.793	0.826
SVM	100	0.852	0.890	0.876	0.738	0.822	0.837
SVM	101	0.837	0.884	0.867	0.716	0.810	0.823
SVM	102	0.844	0.885	0.870	0.724	0.813	0.828
SVM	103	0.844	0.885	0.870	0.724	0.813	0.828
SVM	104	0.844	0.885	0.870	0.724	0.813	0.828
SVM	105	0.844	0.885	0.870	0.724	0.813	0.828
SVM	106	0.844	0.885	0.870	0.724	0.813	0.828
SVM	107	0.844	0.883	0.868	0.721	0.810	0.826
SVM	108	0.848	0.894	0.877	0.739	0.826	0.837
SVM	109	0.848	0.894	0.877	0.739	0.826	0.837
SVM	110	0.846	0.897	0.878	0.740	0.829	0.838

SVM	111	0.846	0.896	0.877	0.738	0.828	0.837
SVM	112	0.848	0.898	0.880	0.743	0.832	0.840
SVM	113	0.866	0.889	0.880	0.747	0.822	0.843
SVM	114	0.866	0.889	0.880	0.747	0.822	0.843
SVM	115	0.866	0.890	0.881	0.749	0.824	0.844
SVM	116	0.863	0.889	0.880	0.746	0.822	0.842
SVM	117	0.866	0.890	0.881	0.749	0.824	0.844
SVM	118	0.868	0.892	0.883	0.752	0.826	0.846
SVM	119	0.872	0.883	0.879	0.745	0.815	0.843
SVM	120	0.861	0.884	0.876	0.737	0.815	0.837
SVM	121	0.857	0.884	0.874	0.734	0.814	0.835
SVM	122	0.857	0.884	0.874	0.734	0.814	0.835
SVM	123	0.857	0.884	0.874	0.734	0.814	0.835
SVM	124	0.857	0.887	0.876	0.737	0.817	0.837
SVM	125	0.855	0.888	0.876	0.736	0.819	0.836
SVM	126	0.848	0.887	0.872	0.729	0.816	0.832
SVM	127	0.848	0.887	0.872	0.729	0.816	0.832
SVM	128	0.846	0.887	0.871	0.727	0.815	0.830
SVM	129	0.846	0.887	0.871	0.727	0.815	0.830
SVM	130	0.848	0.884	0.871	0.726	0.812	0.830
SVM	131	0.861	0.874	0.869	0.725	0.801	0.830
SVM	132	0.861	0.876	0.871	0.728	0.805	0.832
SVM	133	0.861	0.876	0.871	0.728	0.805	0.832
SVM	134	0.859	0.875	0.869	0.725	0.802	0.830
SVM	135	0.861	0.874	0.869	0.725	0.801	0.830

SVM	136	0.861	0.874	0.869	0.725	0.801	0.830
SVM	137	0.859	0.875	0.869	0.725	0.802	0.830
SVM	138	0.863	0.876	0.871	0.730	0.805	0.833
SVM	139	0.861	0.877	0.871	0.730	0.806	0.833
SVM	140	0.861	0.876	0.871	0.728	0.805	0.832
SVM	141	0.859	0.884	0.875	0.735	0.814	0.836
SVM	142	0.859	0.884	0.875	0.735	0.814	0.836
SVM	143	0.859	0.884	0.875	0.735	0.814	0.836
SVM	144	0.859	0.884	0.875	0.735	0.814	0.836
SVM	145	0.859	0.883	0.874	0.734	0.813	0.835
SVM	146	0.861	0.880	0.873	0.733	0.810	0.835
SVM	147	0.861	0.880	0.873	0.733	0.810	0.835
SVM	148	0.859	0.880	0.872	0.731	0.809	0.833
SVM	149	0.866	0.874	0.871	0.729	0.802	0.833
SVM	150	0.866	0.874	0.871	0.729	0.802	0.833
SVM	151	0.863	0.872	0.869	0.725	0.800	0.831
SVM	152	0.868	0.872	0.871	0.729	0.801	0.833
SVM	153	0.861	0.867	0.865	0.717	0.793	0.826
SVM	154	0.859	0.867	0.864	0.715	0.793	0.825
SVM	155	0.859	0.867	0.864	0.715	0.793	0.825
SVM	156	0.859	0.867	0.864	0.715	0.793	0.825
SVM	157	0.863	0.872	0.869	0.725	0.800	0.831
SVM	158	0.863	0.871	0.868	0.724	0.798	0.830
SVM	159	0.863	0.871	0.868	0.724	0.798	0.830
SVM	160	0.861	0.876	0.871	0.728	0.805	0.832

SVM	161	0.863	0.876	0.871	0.730	0.805	0.833
SVM	162	0.859	0.876	0.870	0.726	0.804	0.831
SVM	163	0.861	0.875	0.870	0.726	0.803	0.831
SVM	164	0.861	0.875	0.870	0.726	0.803	0.831
SVM	165	0.859	0.877	0.871	0.728	0.806	0.832
SVM	166	0.859	0.876	0.870	0.726	0.804	0.831
SVM	167	0.859	0.876	0.870	0.726	0.804	0.831
SVM	168	0.859	0.876	0.870	0.726	0.804	0.831
SVM	169	0.861	0.875	0.870	0.726	0.803	0.831
SVM	170	0.861	0.874	0.869	0.725	0.801	0.830
SVM	171	0.863	0.874	0.870	0.727	0.802	0.831
SVM	172	0.861	0.868	0.866	0.719	0.795	0.827
SVM	173	0.861	0.867	0.865	0.717	0.793	0.826
SVM	174	0.861	0.867	0.865	0.717	0.793	0.826
SVM	175	0.861	0.867	0.865	0.717	0.793	0.826
SVM	176	0.861	0.866	0.864	0.716	0.791	0.825
SVM	177	0.848	0.864	0.858	0.703	0.787	0.817
SVM	178	0.850	0.864	0.859	0.705	0.788	0.818
SVM	179	0.846	0.867	0.859	0.704	0.790	0.817
SVM	180	0.846	0.867	0.859	0.704	0.790	0.817
SVM	181	0.879	0.875	0.876	0.742	0.806	0.841
SVM	182	0.877	0.875	0.876	0.740	0.806	0.840
SVM	183	0.877	0.874	0.875	0.738	0.804	0.839
SVM	184	0.877	0.872	0.874	0.737	0.802	0.838
SVM	185	0.877	0.872	0.874	0.737	0.802	0.838

SVM	186	0.877	0.870	0.872	0.734	0.799	0.836
SVM	187	0.879	0.874	0.876	0.740	0.804	0.840
SVM	188	0.877	0.874	0.875	0.738	0.804	0.839
SVM	189	0.877	0.875	0.876	0.740	0.806	0.840
SVM	190	0.879	0.875	0.876	0.742	0.806	0.841
SVM	191	0.881	0.872	0.876	0.741	0.803	0.840
SVM	192	0.881	0.872	0.876	0.741	0.803	0.840
SVM	193	0.881	0.870	0.874	0.738	0.800	0.839
SVM	194	0.877	0.870	0.872	0.734	0.799	0.836
SVM	195	0.881	0.879	0.880	0.748	0.811	0.845
SVM	196	0.881	0.877	0.879	0.747	0.810	0.844
SVM	197	0.881	0.879	0.880	0.748	0.811	0.845
SVM	198	0.879	0.876	0.877	0.743	0.808	0.842
SVM	199	0.879	0.877	0.878	0.745	0.809	0.843
SVM	200	0.872	0.874	0.873	0.735	0.803	0.836
SVM	201	0.872	0.874	0.873	0.735	0.803	0.836
SVM	202	0.883	0.880	0.881	0.752	0.813	0.847
SVM	203	0.879	0.877	0.878	0.745	0.809	0.843
SVM	204	0.883	0.877	0.880	0.749	0.810	0.845
SVM	205	0.883	0.876	0.879	0.747	0.808	0.844
SVM	206	0.883	0.877	0.880	0.749	0.810	0.845
SVM	207	0.883	0.876	0.879	0.747	0.808	0.844
SVM	208	0.881	0.879	0.880	0.748	0.811	0.845
SVM	209	0.881	0.879	0.880	0.748	0.811	0.845
SVM	210	0.883	0.880	0.881	0.752	0.813	0.847

SVM	211	0.883	0.880	0.881	0.752	0.813	0.847
SVM	212	0.881	0.877	0.879	0.747	0.810	0.844
SVM	213	0.877	0.880	0.879	0.746	0.812	0.843
SVM	214	0.879	0.881	0.880	0.750	0.814	0.845
SVM	215	0.877	0.880	0.879	0.746	0.812	0.843
SVM	216	0.877	0.880	0.879	0.746	0.812	0.843
SVM	217	0.877	0.880	0.879	0.746	0.812	0.843
SVM	218	0.874	0.879	0.877	0.743	0.810	0.841
SVM	219	0.874	0.879	0.877	0.743	0.810	0.841
SVM	220	0.874	0.877	0.876	0.741	0.809	0.840
SVM	221	0.874	0.876	0.876	0.740	0.807	0.839
SVM	222	0.874	0.877	0.876	0.741	0.809	0.840
SVM	223	0.872	0.877	0.876	0.739	0.808	0.839
SVM	224	0.872	0.877	0.876	0.739	0.808	0.839
SVM	225	0.872	0.880	0.877	0.742	0.811	0.841
SVM	226	0.872	0.880	0.877	0.742	0.811	0.841
SVM	227	0.872	0.877	0.876	0.739	0.808	0.839
SVM	228	0.874	0.877	0.876	0.741	0.809	0.840
SVM	229	0.874	0.881	0.879	0.746	0.814	0.843
SVM	230	0.874	0.883	0.880	0.747	0.815	0.844
SVM	231	0.874	0.880	0.878	0.744	0.812	0.842
SVM	232	0.877	0.880	0.879	0.746	0.812	0.843
SVM	233	0.874	0.884	0.880	0.749	0.817	0.845
SVM	234	0.874	0.884	0.880	0.749	0.817	0.845
SVM	235	0.850	0.879	0.868	0.722	0.806	0.827

SVM	236	0.850	0.880	0.869	0.723	0.808	0.828
SVM	237	0.850	0.881	0.870	0.725	0.809	0.829
SVM	238	0.850	0.881	0.870	0.725	0.809	0.829
SVM	239	0.848	0.875	0.865	0.715	0.800	0.824
SVM	240	0.848	0.875	0.865	0.715	0.800	0.824
SVM	241	0.848	0.875	0.865	0.715	0.800	0.824
SVM	242	0.852	0.877	0.868	0.722	0.805	0.828
SVM	243	0.848	0.877	0.867	0.718	0.804	0.825
SVM	244	0.846	0.877	0.866	0.716	0.803	0.824
SVM	245	0.848	0.875	0.865	0.715	0.800	0.824
SVM	246	0.848	0.879	0.867	0.720	0.805	0.826
SVM	247	0.850	0.874	0.865	0.715	0.799	0.824
SVM	248	0.850	0.874	0.865	0.715	0.799	0.824
SVM	249	0.850	0.872	0.864	0.714	0.798	0.823
SVM	250	0.839	0.881	0.866	0.715	0.807	0.823
SVM	251	0.839	0.880	0.865	0.714	0.805	0.822
SVM	252	0.837	0.880	0.864	0.712	0.805	0.821
SVM	253	0.837	0.880	0.864	0.712	0.805	0.821
SVM	254	0.839	0.881	0.866	0.715	0.807	0.823
SVM	255	0.846	0.879	0.867	0.718	0.805	0.825
SVM	256	0.846	0.879	0.867	0.718	0.805	0.825
SVM	257	0.855	0.880	0.871	0.727	0.808	0.831
SVM	258	0.855	0.883	0.872	0.730	0.812	0.833
SVM	259	0.855	0.883	0.872	0.730	0.812	0.833
SVM	260	0.855	0.884	0.873	0.732	0.813	0.834

SVM	261	0.855	0.883	0.872	0.730	0.812	0.833
SVM	262	0.855	0.883	0.872	0.730	0.812	0.833
SVM	263	0.852	0.881	0.871	0.727	0.810	0.830
SVM	264	0.855	0.883	0.872	0.730	0.812	0.833
SVM	265	0.861	0.885	0.876	0.739	0.816	0.838
SVM	266	0.859	0.885	0.876	0.737	0.816	0.837
SVM	267	0.859	0.885	0.876	0.737	0.816	0.837
SVM	268	0.859	0.887	0.876	0.739	0.818	0.838
SVM	269	0.855	0.883	0.872	0.730	0.812	0.833
SVM	270	0.859	0.884	0.875	0.735	0.814	0.836
SVM	271	0.855	0.884	0.873	0.732	0.813	0.834
SVM	272	0.855	0.884	0.873	0.732	0.813	0.834
SVM	273	0.855	0.884	0.873	0.732	0.813	0.834
SVM	274	0.855	0.884	0.873	0.732	0.813	0.834
SVM	275	0.855	0.883	0.872	0.730	0.812	0.833
SVM	276	0.855	0.884	0.873	0.732	0.813	0.834
SVM	277	0.857	0.884	0.874	0.734	0.814	0.835
SVM	278	0.855	0.883	0.872	0.730	0.812	0.833
SVM	279	0.855	0.883	0.872	0.730	0.812	0.833
SVM	280	0.855	0.883	0.872	0.730	0.812	0.833
SVM	281	0.855	0.883	0.872	0.730	0.812	0.833
SVM	282	0.859	0.888	0.877	0.740	0.819	0.839
SVM	283	0.859	0.887	0.876	0.739	0.818	0.838
SVM	284	0.868	0.888	0.880	0.748	0.821	0.844
SVM	285	0.868	0.888	0.880	0.748	0.821	0.844

SVM	286	0.866	0.888	0.880	0.746	0.820	0.842
SVM	287	0.866	0.888	0.880	0.746	0.820	0.842
SVM	288	0.870	0.880	0.876	0.740	0.811	0.840
SVM	289	0.855	0.890	0.877	0.739	0.822	0.838
SVM	290	0.855	0.892	0.878	0.741	0.824	0.839
SVM	291	0.855	0.890	0.877	0.739	0.822	0.838
SVM	292	0.859	0.890	0.879	0.743	0.823	0.841
SVM	293	0.859	0.888	0.877	0.740	0.819	0.839
SVM	294	0.859	0.889	0.878	0.742	0.821	0.840
SVM	295	0.852	0.892	0.877	0.739	0.823	0.838
SVM	296	0.855	0.890	0.877	0.739	0.822	0.838
SVM	297	0.852	0.892	0.877	0.739	0.823	0.838
SVM	298	0.852	0.892	0.877	0.739	0.823	0.838
SVM	299	0.837	0.889	0.870	0.723	0.817	0.827
SVM	300	0.839	0.888	0.870	0.723	0.816	0.827
SVM	301	0.839	0.888	0.870	0.723	0.816	0.827
SVM	302	0.839	0.888	0.870	0.723	0.816	0.827
SVM	303	0.839	0.887	0.869	0.721	0.814	0.826
SVM	304	0.837	0.888	0.869	0.721	0.815	0.826
SVM	305	0.837	0.888	0.869	0.721	0.815	0.826
SVM	306	0.841	0.885	0.869	0.722	0.813	0.827
SVM	307	0.841	0.889	0.871	0.727	0.818	0.830
SVM	308	0.844	0.889	0.872	0.728	0.818	0.831
SVM	309	0.844	0.890	0.873	0.730	0.820	0.832
SVM	310	0.844	0.892	0.874	0.732	0.822	0.833

SVM	311	0.850	0.889	0.875	0.734	0.820	0.835
SVM	312	0.848	0.888	0.873	0.731	0.817	0.832
SVM	313	0.883	0.885	0.885	0.758	0.820	0.850
SVM	314	0.883	0.888	0.886	0.761	0.823	0.852
SVM	315	0.881	0.887	0.885	0.758	0.821	0.850
SVM	316	0.870	0.885	0.880	0.747	0.818	0.843
SVM	317	0.881	0.885	0.884	0.756	0.820	0.849
SVM	318	0.881	0.885	0.884	0.756	0.820	0.849
SVM	319	0.883	0.884	0.884	0.756	0.818	0.850
SVM	320	0.885	0.884	0.885	0.758	0.819	0.851
SVM	321	0.883	0.883	0.883	0.755	0.817	0.849
SVM	322	0.885	0.883	0.884	0.757	0.817	0.850
SVM	323	0.883	0.881	0.882	0.753	0.815	0.848
SVM	324	0.885	0.883	0.884	0.757	0.817	0.850
SVM	325	0.877	0.884	0.881	0.751	0.817	0.846
SVM	326	0.877	0.884	0.881	0.751	0.817	0.846
SVM	327	0.879	0.884	0.882	0.753	0.818	0.847
SVM	328	0.877	0.881	0.880	0.748	0.814	0.844
SVM	329	0.888	0.879	0.882	0.754	0.813	0.848
SVM	330	0.888	0.879	0.882	0.754	0.813	0.848
SVM	331	0.885	0.880	0.882	0.754	0.814	0.848
SVM	332	0.885	0.880	0.882	0.754	0.814	0.848
SVM	333	0.883	0.881	0.882	0.753	0.815	0.848
SVM	334	0.883	0.881	0.882	0.753	0.815	0.848
SVM	335	0.883	0.881	0.882	0.753	0.815	0.848

SVM	336	0.866	0.872	0.870	0.727	0.800	0.832
SVM	337	0.866	0.871	0.869	0.726	0.799	0.831
SVM	338	0.861	0.877	0.871	0.730	0.806	0.833
SVM	339	0.861	0.877	0.871	0.730	0.806	0.833
SVM	340	0.861	0.876	0.871	0.728	0.805	0.832
SVM	341	0.863	0.875	0.871	0.728	0.803	0.832
SVM	342	0.868	0.872	0.871	0.729	0.801	0.833
SVM	343	0.868	0.874	0.871	0.731	0.802	0.834
SVM	344	0.868	0.874	0.871	0.731	0.802	0.834
SVM	345	0.866	0.871	0.869	0.726	0.799	0.831
SVM	346	0.866	0.870	0.868	0.724	0.797	0.830
SVM	347	0.870	0.874	0.872	0.733	0.803	0.835
SVM	348	0.859	0.871	0.867	0.720	0.798	0.827
SVM	349	0.859	0.871	0.867	0.720	0.798	0.827
SVM	350	0.866	0.866	0.866	0.720	0.792	0.827
SVM	351	0.863	0.866	0.865	0.718	0.792	0.826
SVM	352	0.863	0.866	0.865	0.718	0.792	0.826
SVM	353	0.861	0.864	0.863	0.714	0.790	0.824
SVM	354	0.863	0.850	0.855	0.700	0.773	0.816
SVM	355	0.863	0.851	0.856	0.701	0.775	0.817
SVM	356	0.859	0.854	0.856	0.700	0.777	0.816
SVM	357	0.861	0.854	0.857	0.702	0.777	0.817
SVM	358	0.859	0.859	0.859	0.706	0.783	0.819
SVM	359	0.859	0.857	0.857	0.703	0.780	0.818
SVM	360	0.859	0.853	0.855	0.699	0.775	0.815

SVM	361	0.859	0.855	0.857	0.702	0.778	0.817
SVM	362	0.852	0.868	0.862	0.711	0.793	0.822
SVM	363	0.863	0.870	0.867	0.722	0.797	0.829
SVM	364	0.859	0.868	0.865	0.717	0.794	0.825
SVM	365	0.848	0.874	0.864	0.713	0.799	0.823
SVM	366	0.852	0.874	0.866	0.717	0.800	0.825
SVM	367	0.857	0.874	0.867	0.721	0.800	0.828
SVM	368	0.859	0.866	0.863	0.714	0.791	0.824
SVM	369	0.859	0.867	0.864	0.715	0.793	0.825
SVM	370	0.852	0.867	0.862	0.710	0.791	0.821

(2) IFS results on the MCFS feature list

**Classification algorithm**	**Number of features**	**SN**	**SP**	**ACC**	**MCC**	**Precision**	**F1-measure**
DT	1	0.758	0.734	0.743	0.478	0.628	0.687
DT	2	0.722	0.729	0.726	0.439	0.612	0.663
DT	3	0.762	0.746	0.752	0.494	0.640	0.695
DT	4	0.778	0.772	0.774	0.536	0.669	0.719
DT	5	0.740	0.760	0.753	0.489	0.646	0.690
DT	6	0.753	0.794	0.779	0.538	0.684	0.717
DT	7	0.753	0.797	0.781	0.541	0.687	0.718
DT	8	0.764	0.794	0.783	0.548	0.687	0.724
DT	9	0.767	0.794	0.784	0.550	0.688	0.725
DT	10	0.771	0.780	0.776	0.538	0.674	0.719
DT	11	0.782	0.793	0.789	0.563	0.691	0.733
DT	12	0.786	0.794	0.791	0.568	0.693	0.737

DT	13	0.751	0.787	0.774	0.529	0.677	0.712
DT	14	0.767	0.789	0.781	0.544	0.682	0.722
DT	15	0.756	0.771	0.765	0.514	0.661	0.705
DT	16	0.758	0.784	0.774	0.530	0.675	0.714
DT	17	0.797	0.757	0.772	0.539	0.661	0.723
DT	18	0.756	0.776	0.768	0.520	0.666	0.708
DT	19	0.775	0.774	0.775	0.537	0.670	0.719
DT	20	0.738	0.771	0.758	0.498	0.656	0.694
DT	21	0.733	0.797	0.773	0.523	0.681	0.706
DT	22	0.764	0.778	0.773	0.531	0.671	0.715
DT	23	0.738	0.789	0.770	0.518	0.674	0.705
DT	24	0.738	0.794	0.773	0.524	0.680	0.707
DT	25	0.749	0.780	0.768	0.518	0.668	0.706
DT	26	0.789	0.798	0.794	0.574	0.698	0.740
DT	27	0.747	0.786	0.771	0.523	0.674	0.708
DT	28	0.758	0.764	0.762	0.509	0.655	0.703
DT	29	0.773	0.774	0.774	0.535	0.670	0.718
DT	30	0.749	0.795	0.778	0.535	0.684	0.715
DT	31	0.764	0.771	0.768	0.522	0.663	0.710
DT	32	0.742	0.807	0.783	0.543	0.695	0.718
DT	33	0.789	0.790	0.790	0.566	0.690	0.736
DT	34	0.784	0.806	0.798	0.579	0.705	0.742
DT	35	0.789	0.811	0.803	0.589	0.712	0.748
DT	36	0.767	0.825	0.803	0.585	0.722	0.744
DT	37	0.749	0.789	0.774	0.528	0.677	0.711

DT	38	0.793	0.798	0.796	0.578	0.699	0.743
DT	39	0.789	0.789	0.789	0.564	0.688	0.735
DT	40	0.791	0.806	0.800	0.585	0.707	0.746
DT	41	0.764	0.811	0.794	0.567	0.705	0.734
DT	42	0.786	0.793	0.790	0.567	0.692	0.736
DT	43	0.784	0.811	0.801	0.585	0.711	0.746
DT	44	0.778	0.811	0.799	0.579	0.709	0.742
DT	45	0.791	0.825	0.812	0.607	0.728	0.758
DT	46	0.773	0.815	0.799	0.579	0.712	0.741
DT	47	0.747	0.797	0.778	0.535	0.685	0.714
DT	48	0.760	0.807	0.790	0.558	0.700	0.729
DT	49	0.749	0.794	0.777	0.534	0.683	0.714
DT	50	0.780	0.807	0.797	0.576	0.705	0.741
DT	51	0.771	0.806	0.793	0.567	0.701	0.735
DT	52	0.769	0.795	0.785	0.553	0.690	0.727
DT	53	0.747	0.806	0.784	0.545	0.695	0.720
DT	54	0.782	0.810	0.799	0.581	0.709	0.743
DT	55	0.778	0.786	0.783	0.551	0.683	0.727
DT	56	0.742	0.802	0.780	0.537	0.689	0.715
DT	57	0.760	0.821	0.799	0.575	0.716	0.737
DT	58	0.762	0.793	0.781	0.544	0.685	0.722
DT	59	0.782	0.802	0.794	0.573	0.700	0.739
DT	60	0.747	0.811	0.787	0.551	0.700	0.723
DT	61	0.800	0.807	0.804	0.594	0.710	0.752
DT	62	0.764	0.823	0.801	0.580	0.718	0.741

DT	63	0.769	0.827	0.805	0.589	0.724	0.746
DT	64	0.791	0.837	0.820	0.620	0.742	0.765
DT	65	0.762	0.791	0.781	0.543	0.684	0.721
DT	66	0.760	0.828	0.803	0.582	0.723	0.741
DT	67	0.753	0.819	0.794	0.566	0.711	0.732
DT	68	0.797	0.794	0.795	0.578	0.696	0.743
DT	69	0.780	0.795	0.790	0.563	0.693	0.734
DT	70	0.740	0.802	0.779	0.535	0.689	0.713
DT	71	0.775	0.804	0.794	0.569	0.701	0.736
DT	72	0.764	0.798	0.785	0.552	0.691	0.726
DT	73	0.780	0.814	0.801	0.584	0.712	0.744
DT	74	0.778	0.808	0.797	0.576	0.706	0.740
DT	75	0.751	0.795	0.779	0.537	0.685	0.716
DT	76	0.791	0.797	0.794	0.575	0.697	0.741
DT	77	0.782	0.789	0.786	0.558	0.687	0.731
DT	78	0.773	0.817	0.801	0.582	0.715	0.743
DT	79	0.804	0.802	0.803	0.593	0.706	0.752
DT	80	0.784	0.804	0.797	0.577	0.704	0.742
DT	81	0.762	0.820	0.799	0.575	0.715	0.738
DT	82	0.722	0.807	0.776	0.525	0.689	0.705
DT	83	0.769	0.797	0.786	0.555	0.691	0.728
DT	84	0.744	0.825	0.795	0.565	0.716	0.730
DT	85	0.740	0.820	0.790	0.556	0.709	0.724
DT	86	0.742	0.825	0.794	0.564	0.715	0.729
DT	87	0.751	0.817	0.793	0.562	0.709	0.729

DT	88	0.782	0.819	0.805	0.592	0.719	0.749
DT	89	0.742	0.810	0.785	0.546	0.698	0.719
DT	90	0.742	0.810	0.785	0.546	0.698	0.719
DT	91	0.731	0.799	0.774	0.524	0.683	0.706
DT	92	0.773	0.823	0.804	0.588	0.721	0.746
DT	93	0.736	0.797	0.774	0.525	0.682	0.708
DT	94	0.762	0.819	0.798	0.574	0.713	0.737
DT	95	0.769	0.798	0.787	0.556	0.692	0.729
DT	96	0.753	0.834	0.804	0.584	0.729	0.741
DT	97	0.749	0.810	0.787	0.551	0.700	0.723
DT	98	0.758	0.823	0.799	0.574	0.717	0.737
DT	99	0.795	0.811	0.805	0.595	0.713	0.752
DT	100	0.782	0.819	0.805	0.592	0.719	0.749
DT	101	0.764	0.812	0.794	0.568	0.707	0.734
DT	102	0.738	0.817	0.788	0.551	0.705	0.721
DT	103	0.764	0.819	0.799	0.576	0.714	0.738
DT	104	0.771	0.811	0.796	0.573	0.707	0.738
DT	105	0.749	0.810	0.787	0.551	0.700	0.723
DT	106	0.740	0.819	0.790	0.554	0.707	0.723
DT	107	0.751	0.831	0.801	0.577	0.724	0.737
DT	108	0.744	0.808	0.785	0.546	0.697	0.720
DT	109	0.716	0.825	0.785	0.540	0.708	0.712
DT	110	0.749	0.815	0.790	0.557	0.705	0.726
DT	111	0.769	0.838	0.812	0.602	0.738	0.753
DT	112	0.749	0.807	0.785	0.549	0.697	0.722

DT	113	0.749	0.833	0.802	0.579	0.726	0.738
DT	114	0.780	0.793	0.788	0.561	0.690	0.732
DT	115	0.758	0.793	0.780	0.540	0.684	0.719
DT	116	0.778	0.802	0.793	0.569	0.699	0.736
DT	117	0.736	0.799	0.776	0.528	0.684	0.709
DT	118	0.753	0.819	0.794	0.566	0.711	0.732
DT	119	0.744	0.812	0.787	0.550	0.701	0.722
DT	120	0.789	0.812	0.803	0.590	0.713	0.749
DT	121	0.747	0.808	0.785	0.548	0.698	0.721
DT	122	0.753	0.825	0.799	0.573	0.718	0.735
DT	123	0.760	0.806	0.789	0.557	0.698	0.728
DT	124	0.762	0.798	0.785	0.550	0.691	0.725
DT	125	0.784	0.811	0.801	0.585	0.711	0.746
DT	126	0.756	0.804	0.786	0.552	0.696	0.724
DT	127	0.780	0.801	0.793	0.569	0.698	0.737
DT	128	0.789	0.797	0.794	0.573	0.696	0.740
DT	129	0.747	0.827	0.797	0.569	0.718	0.732
DT	130	0.749	0.829	0.799	0.574	0.722	0.735
DT	131	0.767	0.817	0.799	0.576	0.713	0.739
DT	132	0.756	0.808	0.789	0.556	0.700	0.727
DT	133	0.758	0.823	0.799	0.574	0.717	0.737
DT	134	0.762	0.812	0.794	0.566	0.706	0.733
DT	135	0.749	0.834	0.803	0.580	0.728	0.738
DT	136	0.767	0.842	0.814	0.605	0.742	0.754
DT	137	0.769	0.802	0.790	0.561	0.697	0.731

DT	138	0.744	0.808	0.785	0.546	0.697	0.720
DT	139	0.744	0.821	0.793	0.561	0.712	0.728
DT	140	0.751	0.816	0.792	0.561	0.707	0.729
DT	141	0.769	0.816	0.799	0.577	0.712	0.739
DT	142	0.775	0.811	0.798	0.577	0.708	0.740
DT	143	0.751	0.836	0.804	0.584	0.730	0.740
DT	144	0.773	0.819	0.802	0.584	0.716	0.744
DT	145	0.771	0.801	0.790	0.561	0.696	0.731
DT	146	0.775	0.814	0.799	0.580	0.711	0.742
DT	147	0.738	0.810	0.783	0.542	0.696	0.717
DT	148	0.753	0.827	0.799	0.575	0.720	0.736
DT	149	0.784	0.793	0.790	0.565	0.691	0.735
DT	150	0.771	0.801	0.790	0.561	0.696	0.731
DT	151	0.760	0.808	0.790	0.560	0.701	0.729
DT	152	0.791	0.817	0.808	0.598	0.719	0.753
DT	153	0.762	0.824	0.801	0.580	0.719	0.740
DT	154	0.786	0.820	0.808	0.597	0.721	0.752
DT	155	0.786	0.785	0.785	0.558	0.684	0.732
DT	156	0.767	0.801	0.788	0.557	0.695	0.729
DT	157	0.769	0.803	0.790	0.562	0.698	0.732
DT	158	0.758	0.807	0.789	0.556	0.699	0.727
DT	159	0.773	0.816	0.800	0.581	0.713	0.742
DT	160	0.780	0.814	0.801	0.584	0.712	0.744
DT	161	0.762	0.797	0.784	0.549	0.689	0.724
DT	162	0.756	0.814	0.792	0.562	0.706	0.730

DT	163	0.758	0.819	0.796	0.570	0.712	0.734
DT	164	0.756	0.806	0.787	0.553	0.697	0.725
DT	165	0.791	0.803	0.799	0.582	0.704	0.745
DT	166	0.751	0.789	0.775	0.530	0.678	0.713
DT	167	0.778	0.810	0.798	0.577	0.707	0.741
DT	168	0.762	0.827	0.803	0.583	0.722	0.742
DT	169	0.791	0.825	0.812	0.607	0.728	0.758
DT	170	0.764	0.836	0.809	0.595	0.734	0.749
DT	171	0.780	0.799	0.792	0.568	0.697	0.736
DT	172	0.740	0.799	0.777	0.532	0.686	0.712
DT	173	0.784	0.817	0.805	0.592	0.718	0.749
DT	174	0.780	0.802	0.794	0.571	0.700	0.738
DT	175	0.771	0.803	0.791	0.564	0.699	0.733
DT	176	0.778	0.811	0.799	0.579	0.709	0.742
DT	177	0.786	0.831	0.814	0.609	0.733	0.759
DT	178	0.751	0.821	0.795	0.567	0.713	0.732
DT	179	0.775	0.825	0.807	0.593	0.724	0.749
DT	180	0.769	0.811	0.795	0.571	0.706	0.736
DT	181	0.749	0.816	0.791	0.559	0.707	0.727
DT	182	0.778	0.808	0.797	0.576	0.706	0.740
DT	183	0.753	0.806	0.786	0.551	0.697	0.724
DT	184	0.749	0.815	0.790	0.557	0.705	0.726
DT	185	0.793	0.812	0.805	0.594	0.714	0.752
DT	186	0.795	0.804	0.801	0.587	0.706	0.748
DT	187	0.760	0.820	0.798	0.573	0.714	0.736

DT	188	0.782	0.812	0.801	0.584	0.711	0.745
DT	189	0.740	0.823	0.792	0.559	0.712	0.726
DT	190	0.782	0.814	0.802	0.586	0.713	0.746
DT	191	0.760	0.789	0.778	0.538	0.680	0.718
DT	192	0.775	0.795	0.788	0.559	0.692	0.731
DT	193	0.740	0.836	0.800	0.574	0.727	0.734
DT	194	0.793	0.833	0.818	0.618	0.738	0.764
DT	195	0.733	0.814	0.784	0.542	0.700	0.716
DT	196	0.767	0.815	0.797	0.573	0.710	0.737
DT	197	0.738	0.812	0.785	0.545	0.699	0.718
DT	198	0.784	0.825	0.810	0.601	0.727	0.754
DT	199	0.782	0.814	0.802	0.586	0.713	0.746
DT	200	0.797	0.824	0.814	0.611	0.728	0.761
DT	201	0.760	0.817	0.796	0.570	0.711	0.735
DT	202	0.780	0.825	0.808	0.597	0.725	0.752
DT	203	0.780	0.812	0.800	0.582	0.711	0.744
DT	204	0.758	0.824	0.799	0.576	0.718	0.737
DT	205	0.771	0.829	0.808	0.594	0.728	0.749
DT	206	0.782	0.808	0.799	0.580	0.707	0.743
DT	207	0.789	0.817	0.807	0.596	0.719	0.752
DT	208	0.738	0.803	0.779	0.534	0.689	0.713
DT	209	0.778	0.806	0.795	0.573	0.703	0.738
DT	210	0.786	0.815	0.804	0.591	0.715	0.749
DT	211	0.789	0.803	0.798	0.580	0.703	0.744
DT	212	0.778	0.780	0.779	0.544	0.676	0.723

DT	213	0.782	0.790	0.787	0.560	0.688	0.732
DT	214	0.756	0.807	0.788	0.554	0.699	0.726
DT	215	0.753	0.811	0.790	0.557	0.702	0.727
DT	216	0.764	0.821	0.800	0.579	0.717	0.740
DT	217	0.775	0.825	0.807	0.593	0.724	0.749
DT	218	0.769	0.793	0.784	0.550	0.687	0.726
DT	219	0.747	0.811	0.787	0.551	0.700	0.723
DT	220	0.758	0.823	0.799	0.574	0.717	0.737
DT	221	0.758	0.828	0.802	0.580	0.723	0.740
DT	222	0.753	0.824	0.798	0.572	0.717	0.735
DT	223	0.775	0.803	0.793	0.568	0.700	0.736
DT	224	0.764	0.802	0.788	0.557	0.695	0.728
DT	225	0.747	0.810	0.786	0.549	0.699	0.722
DT	226	0.780	0.808	0.798	0.578	0.707	0.741
DT	227	0.758	0.819	0.796	0.570	0.712	0.734
DT	228	0.762	0.812	0.794	0.566	0.706	0.733
DT	229	0.722	0.831	0.790	0.552	0.716	0.719
DT	230	0.756	0.798	0.782	0.544	0.689	0.721
DT	231	0.780	0.803	0.794	0.572	0.701	0.738
DT	232	0.773	0.810	0.796	0.573	0.706	0.738
DT	233	0.758	0.808	0.790	0.558	0.701	0.728
DT	234	0.764	0.834	0.808	0.594	0.732	0.748
DT	235	0.780	0.821	0.806	0.592	0.721	0.749
DT	236	0.758	0.811	0.791	0.561	0.703	0.730
DT	237	0.744	0.812	0.787	0.550	0.701	0.722

DT	238	0.751	0.791	0.776	0.533	0.681	0.714
DT	239	0.762	0.821	0.799	0.577	0.716	0.739
DT	240	0.764	0.831	0.806	0.589	0.727	0.745
DT	241	0.764	0.816	0.797	0.573	0.711	0.737
DT	242	0.749	0.815	0.790	0.557	0.705	0.726
DT	243	0.749	0.824	0.796	0.568	0.716	0.732
DT	244	0.727	0.804	0.776	0.526	0.688	0.707
DT	245	0.764	0.832	0.807	0.591	0.729	0.746
DT	246	0.764	0.841	0.812	0.601	0.740	0.752
DT	247	0.762	0.799	0.785	0.552	0.692	0.725
DT	248	0.756	0.829	0.802	0.580	0.724	0.739
DT	249	0.756	0.831	0.803	0.581	0.725	0.740
DT	250	0.784	0.838	0.818	0.616	0.742	0.762
DT	251	0.775	0.798	0.790	0.562	0.694	0.733
DT	252	0.753	0.795	0.780	0.539	0.685	0.718
DT	253	0.773	0.816	0.800	0.581	0.713	0.742
DT	254	0.784	0.793	0.790	0.565	0.691	0.735
DT	255	0.778	0.817	0.803	0.586	0.716	0.746
DT	256	0.764	0.817	0.798	0.574	0.713	0.738
DT	257	0.773	0.801	0.790	0.563	0.696	0.733
DT	258	0.736	0.806	0.780	0.535	0.692	0.713
DT	259	0.742	0.799	0.778	0.534	0.686	0.713
DT	260	0.760	0.807	0.790	0.558	0.700	0.729
DT	261	0.744	0.802	0.781	0.539	0.690	0.716
DT	262	0.753	0.828	0.800	0.576	0.722	0.737

DT	263	0.764	0.838	0.811	0.598	0.737	0.750
DT	264	0.778	0.817	0.803	0.586	0.716	0.746
DT	265	0.767	0.812	0.795	0.570	0.707	0.736
DT	266	0.740	0.802	0.779	0.535	0.689	0.713
DT	267	0.786	0.832	0.815	0.610	0.735	0.760
DT	268	0.753	0.832	0.803	0.581	0.726	0.739
DT	269	0.758	0.832	0.804	0.585	0.727	0.742
DT	270	0.767	0.823	0.802	0.582	0.719	0.742
DT	271	0.747	0.828	0.798	0.570	0.720	0.733
DT	272	0.769	0.816	0.799	0.577	0.712	0.739
DT	273	0.784	0.828	0.812	0.604	0.730	0.756
DT	274	0.764	0.811	0.794	0.567	0.705	0.734
DT	275	0.758	0.817	0.795	0.568	0.711	0.733
DT	276	0.782	0.804	0.796	0.575	0.703	0.740
DT	277	0.771	0.823	0.803	0.586	0.720	0.745
DT	278	0.767	0.816	0.798	0.575	0.712	0.738
DT	279	0.760	0.795	0.782	0.545	0.687	0.722
DT	280	0.762	0.812	0.794	0.566	0.706	0.733
DT	281	0.731	0.801	0.775	0.525	0.685	0.707
DT	282	0.767	0.827	0.804	0.587	0.723	0.744
DT	283	0.749	0.838	0.805	0.585	0.733	0.741
DT	284	0.747	0.824	0.795	0.566	0.715	0.731
DT	285	0.753	0.821	0.796	0.569	0.714	0.733
DT	286	0.756	0.795	0.781	0.541	0.686	0.719
DT	287	0.764	0.832	0.807	0.591	0.729	0.746

DT	288	0.769	0.791	0.783	0.549	0.686	0.725
DT	289	0.760	0.833	0.806	0.588	0.729	0.744
DT	290	0.773	0.806	0.794	0.569	0.702	0.736
DT	291	0.758	0.815	0.794	0.565	0.708	0.732
DT	292	0.736	0.832	0.796	0.565	0.721	0.728
DT	293	0.793	0.795	0.794	0.575	0.696	0.742
DT	294	0.753	0.793	0.778	0.536	0.683	0.716
DT	295	0.756	0.797	0.781	0.543	0.687	0.720
DT	296	0.782	0.801	0.794	0.571	0.699	0.738
DT	297	0.780	0.827	0.809	0.598	0.727	0.752
DT	298	0.800	0.820	0.812	0.609	0.725	0.760
DT	299	0.711	0.821	0.781	0.531	0.702	0.707
DT	300	0.753	0.832	0.803	0.581	0.726	0.739
DT	301	0.762	0.816	0.796	0.571	0.710	0.735
DT	302	0.749	0.825	0.797	0.569	0.717	0.733
DT	303	0.784	0.829	0.812	0.605	0.731	0.757
DT	304	0.767	0.812	0.795	0.570	0.707	0.736
DT	305	0.733	0.812	0.783	0.541	0.698	0.715
DT	306	0.753	0.791	0.777	0.535	0.681	0.715
DT	307	0.753	0.807	0.787	0.552	0.698	0.725
DT	308	0.753	0.825	0.799	0.573	0.718	0.735
DT	309	0.751	0.817	0.793	0.562	0.709	0.729
DT	310	0.773	0.790	0.784	0.552	0.686	0.727
DT	311	0.758	0.829	0.803	0.582	0.724	0.741
DT	312	0.815	0.806	0.809	0.607	0.713	0.761

DT	313	0.771	0.803	0.791	0.564	0.699	0.733
DT	314	0.762	0.808	0.791	0.562	0.702	0.731
DT	315	0.775	0.801	0.791	0.565	0.697	0.734
DT	316	0.740	0.819	0.790	0.554	0.707	0.723
DT	317	0.795	0.814	0.807	0.598	0.716	0.754
DT	318	0.744	0.824	0.794	0.564	0.715	0.729
DT	319	0.806	0.803	0.804	0.596	0.708	0.754
DT	320	0.769	0.802	0.790	0.561	0.697	0.731
DT	321	0.767	0.823	0.802	0.582	0.719	0.742
DT	322	0.780	0.802	0.794	0.571	0.700	0.738
DT	323	0.775	0.817	0.802	0.584	0.715	0.744
DT	324	0.784	0.810	0.800	0.583	0.709	0.745
DT	325	0.767	0.819	0.799	0.578	0.715	0.740
DT	326	0.778	0.797	0.790	0.563	0.694	0.733
DT	327	0.753	0.806	0.786	0.551	0.697	0.724
DT	328	0.775	0.810	0.797	0.575	0.707	0.739
DT	329	0.778	0.795	0.789	0.561	0.692	0.732
DT	330	0.778	0.833	0.812	0.604	0.734	0.755
DT	331	0.778	0.812	0.799	0.580	0.710	0.742
DT	332	0.811	0.811	0.811	0.609	0.717	0.761
DT	333	0.762	0.795	0.783	0.547	0.688	0.723
DT	334	0.773	0.820	0.803	0.585	0.718	0.744
DT	335	0.744	0.801	0.780	0.537	0.688	0.715
DT	336	0.775	0.787	0.783	0.551	0.683	0.727
DT	337	0.738	0.810	0.783	0.542	0.696	0.717

DT	338	0.780	0.797	0.790	0.565	0.694	0.734
DT	339	0.753	0.819	0.794	0.566	0.711	0.732
DT	340	0.760	0.819	0.797	0.572	0.713	0.736
DT	341	0.764	0.807	0.791	0.562	0.701	0.731
DT	342	0.756	0.847	0.813	0.601	0.746	0.751
DT	343	0.797	0.801	0.799	0.585	0.703	0.747
DT	344	0.760	0.794	0.781	0.544	0.686	0.721
DT	345	0.747	0.808	0.785	0.548	0.698	0.721
DT	346	0.769	0.815	0.798	0.575	0.711	0.739
DT	347	0.778	0.816	0.802	0.585	0.715	0.745
DT	348	0.782	0.801	0.794	0.571	0.699	0.738
DT	349	0.782	0.827	0.810	0.600	0.727	0.754
DT	350	0.756	0.824	0.799	0.574	0.718	0.736
DT	351	0.742	0.798	0.777	0.532	0.685	0.712
DT	352	0.789	0.803	0.798	0.580	0.703	0.744
DT	353	0.771	0.821	0.803	0.585	0.719	0.744
DT	354	0.773	0.815	0.799	0.579	0.712	0.741
DT	355	0.786	0.797	0.793	0.571	0.696	0.738
DT	356	0.738	0.828	0.794	0.563	0.717	0.727
DT	357	0.784	0.801	0.794	0.573	0.699	0.739
DT	358	0.767	0.810	0.794	0.567	0.704	0.734
DT	359	0.769	0.828	0.806	0.590	0.726	0.747
DT	360	0.780	0.814	0.801	0.584	0.712	0.744
DT	361	0.751	0.820	0.794	0.565	0.712	0.731
DT	362	0.744	0.828	0.797	0.569	0.719	0.732

DT	363	0.749	0.811	0.788	0.553	0.701	0.724
DT	364	0.780	0.810	0.799	0.579	0.708	0.742
DT	365	0.716	0.828	0.786	0.543	0.711	0.714
DT	366	0.775	0.828	0.808	0.596	0.727	0.751
DT	367	0.764	0.823	0.801	0.580	0.718	0.741
DT	368	0.771	0.814	0.798	0.576	0.710	0.739
DT	369	0.736	0.823	0.790	0.555	0.711	0.723
DT	370	0.758	0.803	0.786	0.552	0.695	0.725
KNN	1	0.738	0.734	0.735	0.459	0.622	0.675
KNN	2	0.791	0.759	0.771	0.534	0.660	0.719
KNN	3	0.778	0.785	0.782	0.550	0.681	0.726
KNN	4	0.841	0.799	0.815	0.624	0.713	0.772
KNN	5	0.815	0.773	0.789	0.572	0.680	0.741
KNN	6	0.835	0.793	0.808	0.611	0.704	0.764
KNN	7	0.826	0.801	0.810	0.611	0.710	0.764
KNN	8	0.833	0.790	0.806	0.606	0.701	0.761
KNN	9	0.835	0.784	0.803	0.601	0.695	0.759
KNN	10	0.802	0.786	0.792	0.574	0.689	0.741
KNN	11	0.833	0.782	0.801	0.598	0.694	0.757
KNN	12	0.813	0.776	0.790	0.573	0.682	0.742
KNN	13	0.824	0.782	0.798	0.590	0.691	0.752
KNN	14	0.826	0.791	0.804	0.601	0.701	0.758
KNN	15	0.830	0.793	0.807	0.607	0.703	0.762
KNN	16	0.852	0.793	0.815	0.627	0.709	0.774
KNN	17	0.833	0.804	0.815	0.621	0.716	0.770

KNN	18	0.839	0.807	0.819	0.630	0.720	0.775
KNN	19	0.841	0.799	0.815	0.624	0.713	0.772
KNN	20	0.861	0.790	0.817	0.632	0.708	0.777
KNN	21	0.841	0.802	0.817	0.627	0.715	0.773
KNN	22	0.872	0.811	0.834	0.665	0.732	0.796
KNN	23	0.844	0.802	0.817	0.629	0.716	0.775
KNN	24	0.848	0.777	0.803	0.607	0.692	0.762
KNN	25	0.839	0.784	0.804	0.605	0.697	0.761
KNN	26	0.850	0.786	0.810	0.618	0.702	0.769
KNN	27	0.857	0.791	0.816	0.630	0.709	0.776
KNN	28	0.835	0.799	0.812	0.618	0.711	0.768
KNN	29	0.852	0.790	0.813	0.624	0.706	0.772
KNN	30	0.863	0.798	0.822	0.643	0.717	0.783
KNN	31	0.881	0.786	0.821	0.647	0.709	0.786
KNN	32	0.874	0.787	0.820	0.642	0.709	0.783
KNN	33	0.841	0.798	0.814	0.622	0.711	0.771
KNN	34	0.879	0.787	0.821	0.646	0.710	0.785
KNN	35	0.881	0.794	0.826	0.655	0.717	0.791
KNN	36	0.877	0.789	0.821	0.645	0.711	0.785
KNN	37	0.859	0.799	0.821	0.640	0.717	0.782
KNN	38	0.872	0.771	0.808	0.623	0.692	0.772
KNN	39	0.872	0.768	0.807	0.620	0.690	0.770
KNN	40	0.879	0.767	0.808	0.625	0.690	0.773
KNN	41	0.859	0.780	0.809	0.620	0.698	0.770
KNN	42	0.870	0.777	0.812	0.627	0.698	0.775

KNN	43	0.881	0.773	0.813	0.633	0.697	0.778
KNN	44	0.870	0.781	0.814	0.631	0.702	0.777
KNN	45	0.861	0.780	0.810	0.622	0.698	0.771
KNN	46	0.892	0.771	0.816	0.641	0.697	0.783
KNN	47	0.874	0.777	0.813	0.631	0.699	0.777
KNN	48	0.868	0.785	0.816	0.633	0.705	0.778
KNN	49	0.859	0.781	0.810	0.621	0.699	0.771
KNN	50	0.872	0.784	0.817	0.636	0.705	0.780
KNN	51	0.877	0.769	0.809	0.625	0.692	0.774
KNN	52	0.879	0.771	0.811	0.629	0.694	0.776
KNN	53	0.870	0.773	0.809	0.623	0.694	0.772
KNN	54	0.872	0.781	0.815	0.633	0.702	0.778
KNN	55	0.890	0.790	0.827	0.659	0.715	0.793
KNN	56	0.877	0.795	0.826	0.652	0.717	0.789
KNN	57	0.866	0.799	0.824	0.646	0.718	0.785
KNN	58	0.874	0.790	0.821	0.645	0.711	0.785
KNN	59	0.888	0.790	0.826	0.657	0.715	0.792
KNN	60	0.885	0.777	0.817	0.642	0.702	0.783
KNN	61	0.855	0.790	0.814	0.626	0.707	0.774
KNN	62	0.899	0.802	0.838	0.679	0.729	0.805
KNN	63	0.881	0.799	0.830	0.660	0.722	0.794
KNN	64	0.872	0.789	0.820	0.641	0.710	0.783
KNN	65	0.868	0.797	0.823	0.645	0.716	0.785
KNN	66	0.874	0.791	0.822	0.646	0.713	0.785
KNN	67	0.888	0.797	0.830	0.664	0.721	0.796

KNN	68	0.837	0.799	0.813	0.620	0.712	0.769
KNN	69	0.892	0.803	0.836	0.675	0.728	0.802
KNN	70	0.883	0.787	0.823	0.650	0.711	0.788
KNN	71	0.870	0.794	0.822	0.645	0.714	0.785
KNN	72	0.870	0.794	0.822	0.645	0.714	0.785
KNN	73	0.870	0.787	0.818	0.638	0.708	0.781
KNN	74	0.881	0.787	0.822	0.648	0.710	0.787
KNN	75	0.870	0.794	0.822	0.645	0.714	0.785
KNN	76	0.872	0.803	0.829	0.656	0.724	0.791
KNN	77	0.888	0.795	0.830	0.662	0.720	0.795
KNN	78	0.899	0.784	0.826	0.661	0.711	0.794
KNN	79	0.872	0.785	0.817	0.637	0.706	0.780
KNN	80	0.883	0.802	0.832	0.665	0.725	0.796
KNN	81	0.870	0.804	0.829	0.656	0.725	0.791
KNN	82	0.863	0.799	0.823	0.644	0.718	0.784
KNN	83	0.859	0.799	0.821	0.640	0.717	0.782
KNN	84	0.892	0.793	0.830	0.664	0.718	0.796
KNN	85	0.896	0.797	0.834	0.672	0.723	0.800
KNN	86	0.894	0.804	0.838	0.678	0.730	0.804
KNN	87	0.901	0.797	0.835	0.676	0.724	0.803
KNN	88	0.890	0.790	0.827	0.659	0.715	0.793
KNN	89	0.883	0.810	0.837	0.673	0.733	0.801
KNN	90	0.866	0.820	0.837	0.668	0.740	0.798
KNN	91	0.885	0.806	0.835	0.671	0.730	0.800
KNN	92	0.863	0.793	0.819	0.637	0.711	0.780

KNN	93	0.877	0.795	0.826	0.652	0.717	0.789
KNN	94	0.846	0.808	0.822	0.638	0.723	0.780
KNN	95	0.879	0.799	0.829	0.658	0.722	0.792
KNN	96	0.879	0.789	0.822	0.647	0.711	0.786
KNN	97	0.890	0.791	0.828	0.660	0.716	0.794
KNN	98	0.879	0.780	0.817	0.638	0.702	0.781
KNN	99	0.859	0.774	0.806	0.614	0.693	0.767
KNN	100	0.857	0.764	0.799	0.601	0.682	0.760
KNN	101	0.868	0.760	0.800	0.608	0.682	0.764
KNN	102	0.857	0.760	0.796	0.597	0.679	0.758
KNN	103	0.868	0.769	0.806	0.617	0.690	0.769
KNN	104	0.872	0.763	0.803	0.615	0.685	0.767
KNN	105	0.872	0.768	0.807	0.620	0.690	0.770
KNN	106	0.874	0.771	0.809	0.625	0.693	0.773
KNN	107	0.846	0.759	0.791	0.586	0.675	0.751
KNN	108	0.844	0.768	0.796	0.593	0.683	0.755
KNN	109	0.903	0.767	0.817	0.648	0.696	0.786
KNN	110	0.846	0.772	0.799	0.599	0.687	0.758
KNN	111	0.868	0.767	0.804	0.614	0.688	0.767
KNN	112	0.866	0.771	0.806	0.616	0.691	0.768
KNN	113	0.879	0.763	0.806	0.621	0.687	0.771
KNN	114	0.868	0.763	0.802	0.610	0.684	0.765
KNN	115	0.855	0.771	0.802	0.606	0.688	0.762
KNN	116	0.846	0.756	0.790	0.583	0.673	0.749
KNN	117	0.844	0.755	0.788	0.580	0.671	0.747

KNN	118	0.841	0.747	0.782	0.570	0.663	0.742
KNN	119	0.868	0.751	0.794	0.599	0.674	0.758
KNN	120	0.868	0.747	0.792	0.595	0.670	0.756
KNN	121	0.868	0.755	0.797	0.603	0.677	0.761
KNN	122	0.837	0.765	0.792	0.584	0.679	0.750
KNN	123	0.848	0.768	0.798	0.597	0.684	0.757
KNN	124	0.861	0.757	0.796	0.599	0.678	0.758
KNN	125	0.859	0.751	0.791	0.590	0.671	0.754
KNN	126	0.870	0.752	0.796	0.602	0.675	0.760
KNN	127	0.837	0.763	0.790	0.582	0.676	0.748
KNN	128	0.837	0.765	0.792	0.584	0.679	0.750
KNN	129	0.885	0.765	0.810	0.630	0.691	0.776
KNN	130	0.859	0.756	0.794	0.596	0.676	0.757
KNN	131	0.872	0.746	0.793	0.598	0.670	0.758
KNN	132	0.852	0.748	0.787	0.581	0.667	0.749
KNN	133	0.863	0.761	0.799	0.605	0.682	0.762
KNN	134	0.841	0.763	0.792	0.586	0.677	0.750
KNN	135	0.859	0.771	0.803	0.610	0.689	0.765
KNN	136	0.844	0.755	0.788	0.580	0.671	0.747
KNN	137	0.848	0.767	0.797	0.596	0.683	0.756
KNN	138	0.857	0.760	0.796	0.597	0.679	0.758
KNN	139	0.881	0.747	0.797	0.607	0.673	0.763
KNN	140	0.861	0.751	0.792	0.592	0.672	0.755
KNN	141	0.857	0.757	0.794	0.595	0.677	0.756
KNN	142	0.870	0.754	0.797	0.603	0.676	0.761

KNN	143	0.850	0.750	0.787	0.581	0.668	0.748
KNN	144	0.868	0.769	0.806	0.617	0.690	0.769
KNN	145	0.837	0.754	0.785	0.572	0.668	0.743
KNN	146	0.850	0.751	0.788	0.582	0.669	0.749
KNN	147	0.841	0.760	0.790	0.583	0.675	0.749
KNN	148	0.855	0.741	0.783	0.576	0.661	0.745
KNN	149	0.872	0.754	0.798	0.605	0.677	0.762
KNN	150	0.852	0.751	0.789	0.584	0.670	0.750
KNN	151	0.857	0.757	0.794	0.595	0.677	0.756
KNN	152	0.852	0.760	0.794	0.593	0.678	0.755
KNN	153	0.872	0.747	0.794	0.599	0.671	0.759
KNN	154	0.852	0.752	0.790	0.585	0.671	0.751
KNN	155	0.844	0.748	0.784	0.573	0.665	0.744
KNN	156	0.857	0.754	0.792	0.591	0.673	0.754
KNN	157	0.859	0.746	0.788	0.585	0.667	0.751
KNN	158	0.877	0.747	0.795	0.603	0.672	0.761
KNN	159	0.870	0.741	0.789	0.590	0.665	0.754
KNN	160	0.830	0.743	0.776	0.555	0.657	0.733
KNN	161	0.881	0.737	0.790	0.597	0.664	0.758
KNN	162	0.859	0.737	0.782	0.576	0.659	0.746
KNN	163	0.866	0.748	0.792	0.594	0.671	0.756
KNN	164	0.863	0.729	0.779	0.573	0.653	0.744
KNN	165	0.861	0.731	0.780	0.573	0.655	0.744
KNN	166	0.819	0.718	0.756	0.520	0.633	0.714
KNN	167	0.844	0.720	0.766	0.545	0.640	0.728

KNN	168	0.837	0.720	0.763	0.538	0.639	0.725
KNN	169	0.844	0.725	0.769	0.550	0.645	0.731
KNN	170	0.850	0.724	0.771	0.555	0.645	0.734
KNN	171	0.844	0.704	0.756	0.529	0.628	0.720
KNN	172	0.855	0.718	0.769	0.554	0.642	0.733
KNN	173	0.866	0.713	0.770	0.559	0.641	0.737
KNN	174	0.819	0.717	0.755	0.519	0.632	0.713
KNN	175	0.839	0.720	0.764	0.540	0.639	0.726
KNN	176	0.852	0.724	0.771	0.557	0.646	0.735
KNN	177	0.837	0.716	0.761	0.534	0.635	0.722
KNN	178	0.830	0.713	0.757	0.526	0.631	0.717
KNN	179	0.833	0.716	0.759	0.530	0.634	0.720
KNN	180	0.835	0.716	0.760	0.532	0.635	0.721
KNN	181	0.830	0.718	0.760	0.531	0.636	0.720
KNN	182	0.826	0.725	0.762	0.533	0.640	0.721
KNN	183	0.841	0.718	0.764	0.541	0.639	0.726
KNN	184	0.850	0.733	0.776	0.564	0.653	0.739
KNN	185	0.857	0.730	0.777	0.567	0.653	0.741
KNN	186	0.841	0.728	0.770	0.550	0.646	0.731
KNN	187	0.877	0.728	0.783	0.584	0.656	0.750
KNN	188	0.863	0.726	0.777	0.570	0.651	0.742
KNN	189	0.846	0.731	0.774	0.558	0.651	0.736
KNN	190	0.861	0.724	0.775	0.565	0.648	0.740
KNN	191	0.852	0.725	0.772	0.558	0.647	0.736
KNN	192	0.830	0.743	0.776	0.555	0.657	0.733

KNN	193	0.839	0.717	0.762	0.538	0.637	0.724
KNN	194	0.857	0.731	0.778	0.569	0.654	0.742
KNN	195	0.850	0.728	0.773	0.559	0.649	0.736
KNN	196	0.835	0.731	0.770	0.548	0.648	0.730
KNN	197	0.855	0.738	0.781	0.573	0.659	0.744
KNN	198	0.844	0.743	0.781	0.568	0.660	0.741
KNN	199	0.863	0.714	0.770	0.559	0.642	0.736
KNN	200	0.841	0.724	0.767	0.546	0.643	0.729
KNN	201	0.828	0.738	0.771	0.548	0.652	0.729
KNN	202	0.830	0.737	0.771	0.549	0.651	0.730
KNN	203	0.848	0.688	0.748	0.519	0.617	0.714
KNN	204	0.868	0.692	0.758	0.542	0.625	0.727
KNN	205	0.839	0.712	0.759	0.533	0.633	0.722
KNN	206	0.841	0.713	0.761	0.536	0.635	0.723
KNN	207	0.817	0.712	0.751	0.512	0.627	0.709
KNN	208	0.857	0.703	0.760	0.541	0.630	0.726
KNN	209	0.822	0.705	0.749	0.509	0.623	0.708
KNN	210	0.822	0.701	0.746	0.506	0.620	0.706
KNN	211	0.855	0.698	0.756	0.534	0.626	0.723
KNN	212	0.848	0.701	0.756	0.531	0.627	0.721
KNN	213	0.828	0.699	0.747	0.509	0.619	0.709
KNN	214	0.835	0.695	0.747	0.512	0.618	0.710
KNN	215	0.852	0.699	0.756	0.533	0.626	0.722
KNN	216	0.857	0.688	0.751	0.527	0.619	0.719
KNN	217	0.797	0.695	0.733	0.476	0.607	0.690

KNN	218	0.874	0.696	0.762	0.552	0.630	0.732
KNN	219	0.837	0.692	0.746	0.512	0.617	0.710
KNN	220	0.839	0.694	0.748	0.515	0.619	0.712
KNN	221	0.844	0.674	0.737	0.501	0.605	0.705
KNN	222	0.824	0.695	0.743	0.501	0.615	0.704
KNN	223	0.802	0.675	0.722	0.461	0.594	0.682
KNN	224	0.813	0.671	0.724	0.468	0.594	0.687
KNN	225	0.795	0.692	0.731	0.471	0.605	0.687
KNN	226	0.828	0.682	0.736	0.493	0.606	0.700
KNN	227	0.791	0.682	0.722	0.457	0.595	0.679
KNN	228	0.789	0.694	0.729	0.466	0.604	0.684
KNN	229	0.786	0.686	0.723	0.456	0.597	0.679
KNN	230	0.780	0.691	0.724	0.455	0.599	0.678
KNN	231	0.802	0.683	0.727	0.469	0.600	0.686
KNN	232	0.791	0.674	0.717	0.449	0.589	0.675
KNN	233	0.797	0.674	0.720	0.456	0.592	0.679
KNN	234	0.775	0.669	0.708	0.429	0.581	0.664
KNN	235	0.791	0.694	0.730	0.468	0.604	0.685
KNN	236	0.782	0.675	0.715	0.442	0.588	0.671
KNN	237	0.808	0.684	0.731	0.476	0.603	0.690
KNN	238	0.797	0.684	0.726	0.466	0.599	0.684
KNN	239	0.764	0.679	0.711	0.429	0.585	0.663
KNN	240	0.767	0.700	0.725	0.452	0.602	0.674
KNN	241	0.802	0.688	0.731	0.474	0.604	0.689
KNN	242	0.802	0.686	0.729	0.471	0.602	0.687

KNN	243	0.778	0.679	0.716	0.442	0.589	0.670
KNN	244	0.749	0.679	0.705	0.414	0.580	0.654
KNN	245	0.784	0.686	0.722	0.454	0.596	0.677
KNN	246	0.771	0.686	0.717	0.442	0.592	0.670
KNN	247	0.778	0.694	0.725	0.456	0.600	0.678
KNN	248	0.780	0.681	0.717	0.445	0.591	0.672
KNN	249	0.800	0.686	0.728	0.469	0.601	0.686
KNN	250	0.786	0.668	0.712	0.439	0.583	0.670
KNN	251	0.793	0.684	0.725	0.462	0.598	0.682
KNN	252	0.806	0.673	0.722	0.463	0.593	0.683
KNN	253	0.773	0.678	0.713	0.436	0.587	0.667
KNN	254	0.795	0.673	0.718	0.452	0.590	0.677
KNN	255	0.789	0.681	0.721	0.453	0.594	0.677
KNN	256	0.778	0.688	0.722	0.451	0.596	0.675
KNN	257	0.793	0.678	0.721	0.455	0.593	0.679
KNN	258	0.800	0.683	0.726	0.467	0.599	0.685
KNN	259	0.769	0.682	0.714	0.436	0.589	0.667
KNN	260	0.762	0.687	0.715	0.435	0.590	0.665
KNN	261	0.784	0.675	0.716	0.444	0.588	0.672
KNN	262	0.789	0.678	0.719	0.451	0.592	0.676
KNN	263	0.795	0.674	0.719	0.454	0.591	0.678
KNN	264	0.784	0.682	0.720	0.451	0.593	0.676
KNN	265	0.780	0.687	0.722	0.451	0.596	0.676
KNN	266	0.775	0.687	0.720	0.447	0.595	0.673
KNN	267	0.767	0.675	0.709	0.427	0.583	0.662

KNN	268	0.778	0.683	0.718	0.445	0.592	0.672
KNN	269	0.775	0.684	0.718	0.445	0.593	0.672
KNN	270	0.775	0.669	0.708	0.429	0.581	0.664
KNN	271	0.762	0.681	0.711	0.428	0.585	0.662
KNN	272	0.795	0.692	0.731	0.471	0.605	0.687
KNN	273	0.780	0.681	0.717	0.445	0.591	0.672
KNN	274	0.782	0.691	0.725	0.457	0.600	0.679
KNN	275	0.780	0.675	0.714	0.440	0.587	0.670
KNN	276	0.800	0.686	0.728	0.469	0.601	0.686
KNN	277	0.773	0.695	0.724	0.453	0.600	0.676
KNN	278	0.800	0.681	0.725	0.464	0.597	0.684
KNN	279	0.800	0.677	0.722	0.460	0.594	0.682
KNN	280	0.797	0.682	0.725	0.463	0.597	0.683
KNN	281	0.769	0.692	0.721	0.446	0.597	0.672
KNN	282	0.817	0.681	0.731	0.481	0.602	0.693
KNN	283	0.808	0.679	0.727	0.471	0.599	0.688
KNN	284	0.780	0.681	0.717	0.445	0.591	0.672
KNN	285	0.791	0.670	0.715	0.445	0.587	0.674
KNN	286	0.771	0.683	0.716	0.439	0.590	0.669
KNN	287	0.797	0.668	0.716	0.449	0.587	0.676
KNN	288	0.758	0.675	0.706	0.419	0.580	0.657
KNN	289	0.802	0.670	0.719	0.456	0.590	0.680
KNN	290	0.778	0.679	0.716	0.442	0.589	0.670
KNN	291	0.793	0.666	0.713	0.444	0.584	0.673
KNN	292	0.778	0.677	0.714	0.439	0.587	0.669

KNN	293	0.767	0.681	0.713	0.432	0.587	0.665
KNN	294	0.780	0.683	0.719	0.448	0.593	0.674
KNN	295	0.784	0.683	0.721	0.452	0.594	0.676
KNN	296	0.795	0.677	0.721	0.456	0.593	0.679
KNN	297	0.771	0.690	0.720	0.446	0.595	0.672
KNN	298	0.822	0.686	0.736	0.490	0.607	0.699
KNN	299	0.806	0.692	0.735	0.482	0.608	0.693
KNN	300	0.764	0.679	0.711	0.429	0.585	0.663
KNN	301	0.793	0.674	0.718	0.451	0.590	0.677
KNN	302	0.786	0.674	0.716	0.445	0.588	0.673
KNN	303	0.778	0.690	0.722	0.452	0.597	0.676
KNN	304	0.773	0.675	0.712	0.434	0.585	0.666
KNN	305	0.804	0.694	0.735	0.481	0.608	0.693
KNN	306	0.771	0.675	0.711	0.431	0.584	0.665
KNN	307	0.795	0.694	0.731	0.473	0.606	0.688
KNN	308	0.778	0.695	0.726	0.457	0.601	0.678
KNN	309	0.808	0.688	0.733	0.480	0.606	0.692
KNN	310	0.795	0.686	0.726	0.465	0.600	0.684
KNN	311	0.789	0.678	0.719	0.451	0.592	0.676
KNN	312	0.815	0.686	0.734	0.484	0.606	0.695
KNN	313	0.806	0.670	0.721	0.460	0.591	0.682
KNN	314	0.795	0.674	0.719	0.454	0.591	0.678
KNN	315	0.782	0.662	0.707	0.429	0.578	0.665
KNN	316	0.775	0.688	0.721	0.448	0.596	0.674
KNN	317	0.793	0.687	0.726	0.464	0.600	0.683

KNN	318	0.789	0.684	0.723	0.457	0.597	0.679
KNN	319	0.786	0.707	0.736	0.477	0.613	0.689
KNN	320	0.795	0.698	0.734	0.476	0.609	0.690
KNN	321	0.782	0.692	0.726	0.459	0.601	0.679
KNN	322	0.778	0.707	0.733	0.469	0.611	0.684
KNN	323	0.791	0.714	0.743	0.489	0.621	0.696
KNN	324	0.775	0.703	0.730	0.463	0.607	0.681
KNN	325	0.791	0.694	0.730	0.468	0.604	0.685
KNN	326	0.762	0.700	0.723	0.448	0.601	0.672
KNN	327	0.784	0.686	0.722	0.454	0.596	0.677
KNN	328	0.786	0.703	0.734	0.473	0.610	0.687
KNN	329	0.800	0.698	0.735	0.481	0.610	0.692
KNN	330	0.813	0.700	0.742	0.496	0.616	0.701
KNN	331	0.751	0.701	0.720	0.438	0.598	0.666
KNN	332	0.742	0.699	0.715	0.427	0.593	0.659
KNN	333	0.784	0.711	0.738	0.479	0.616	0.690
KNN	334	0.764	0.684	0.714	0.434	0.589	0.665
KNN	335	0.802	0.709	0.744	0.494	0.620	0.699
KNN	336	0.775	0.696	0.726	0.456	0.602	0.678
KNN	337	0.760	0.692	0.717	0.438	0.594	0.667
KNN	338	0.791	0.698	0.732	0.472	0.607	0.687
KNN	339	0.813	0.686	0.733	0.482	0.605	0.694
KNN	340	0.786	0.686	0.723	0.456	0.597	0.679
KNN	341	0.775	0.699	0.727	0.459	0.604	0.679
KNN	342	0.789	0.699	0.732	0.471	0.608	0.686

KNN	343	0.806	0.690	0.733	0.479	0.606	0.692
KNN	344	0.813	0.688	0.735	0.484	0.607	0.695
KNN	345	0.769	0.684	0.716	0.438	0.591	0.668
KNN	346	0.817	0.692	0.739	0.492	0.611	0.699
KNN	347	0.806	0.692	0.735	0.482	0.608	0.693
KNN	348	0.795	0.686	0.726	0.465	0.600	0.684
KNN	349	0.802	0.690	0.731	0.475	0.605	0.689
KNN	350	0.844	0.691	0.748	0.517	0.618	0.713
KNN	351	0.775	0.699	0.727	0.459	0.604	0.679
KNN	352	0.778	0.683	0.718	0.445	0.592	0.672
KNN	353	0.782	0.695	0.727	0.461	0.603	0.681
KNN	354	0.791	0.681	0.722	0.456	0.594	0.679
KNN	355	0.778	0.692	0.724	0.454	0.599	0.677
KNN	356	0.797	0.681	0.724	0.462	0.596	0.682
KNN	357	0.784	0.686	0.722	0.454	0.596	0.677
KNN	358	0.773	0.686	0.718	0.444	0.593	0.671
KNN	359	0.782	0.690	0.724	0.456	0.599	0.678
KNN	360	0.786	0.688	0.725	0.459	0.599	0.680
KNN	361	0.782	0.684	0.721	0.451	0.595	0.676
KNN	362	0.767	0.698	0.723	0.449	0.600	0.673
KNN	363	0.784	0.688	0.724	0.457	0.598	0.679
KNN	364	0.760	0.703	0.724	0.448	0.602	0.672
KNN	365	0.795	0.714	0.744	0.493	0.622	0.698
KNN	366	0.797	0.698	0.735	0.479	0.609	0.691
KNN	367	0.778	0.700	0.729	0.462	0.605	0.681

KNN	368	0.811	0.688	0.734	0.482	0.606	0.694
KNN	369	0.771	0.698	0.725	0.453	0.601	0.676
KNN	370	0.784	0.690	0.725	0.458	0.599	0.679
RF	1	0.729	0.743	0.738	0.461	0.627	0.674
RF	2	0.795	0.778	0.785	0.559	0.680	0.733
RF	3	0.800	0.814	0.808	0.602	0.717	0.756
RF	4	0.822	0.831	0.827	0.640	0.742	0.780
RF	5	0.808	0.842	0.830	0.642	0.752	0.779
RF	6	0.824	0.860	0.847	0.677	0.778	0.800
RF	7	0.817	0.847	0.836	0.656	0.760	0.788
RF	8	0.811	0.860	0.842	0.665	0.775	0.792
RF	9	0.819	0.849	0.838	0.659	0.762	0.790
RF	10	0.808	0.847	0.833	0.648	0.758	0.783
RF	11	0.824	0.851	0.841	0.666	0.766	0.794
RF	12	0.839	0.853	0.848	0.681	0.771	0.804
RF	13	0.826	0.853	0.843	0.670	0.768	0.796
RF	14	0.822	0.860	0.846	0.675	0.777	0.799
RF	15	0.835	0.858	0.849	0.683	0.777	0.805
RF	16	0.830	0.860	0.849	0.683	0.779	0.804
RF	17	0.824	0.858	0.845	0.674	0.774	0.798
RF	18	0.822	0.855	0.843	0.669	0.771	0.795
RF	19	0.822	0.853	0.841	0.666	0.767	0.794
RF	20	0.830	0.871	0.856	0.695	0.792	0.811
RF	21	0.819	0.854	0.841	0.665	0.769	0.793
RF	22	0.824	0.866	0.850	0.683	0.784	0.803

RF	23	0.819	0.857	0.843	0.668	0.772	0.795
RF	24	0.808	0.863	0.843	0.666	0.778	0.793
RF	25	0.835	0.860	0.851	0.686	0.780	0.806
RF	26	0.808	0.862	0.842	0.665	0.776	0.792
RF	27	0.817	0.864	0.847	0.676	0.781	0.799
RF	28	0.822	0.860	0.846	0.675	0.777	0.799
RF	29	0.837	0.864	0.854	0.693	0.785	0.810
RF	30	0.828	0.864	0.851	0.685	0.783	0.805
RF	31	0.826	0.868	0.853	0.688	0.788	0.806
RF	32	0.833	0.871	0.857	0.697	0.792	0.812
RF	33	0.841	0.874	0.862	0.708	0.797	0.819
RF	34	0.844	0.879	0.866	0.716	0.805	0.824
RF	35	0.841	0.877	0.864	0.712	0.803	0.822
RF	36	0.848	0.871	0.862	0.710	0.795	0.821
RF	37	0.837	0.884	0.867	0.716	0.810	0.823
RF	38	0.841	0.880	0.866	0.715	0.806	0.823
RF	39	0.837	0.871	0.858	0.701	0.793	0.815
RF	40	0.850	0.871	0.863	0.712	0.796	0.822
RF	41	0.833	0.884	0.865	0.713	0.809	0.821
RF	42	0.848	0.877	0.867	0.718	0.804	0.825
RF	43	0.846	0.879	0.867	0.718	0.805	0.825
RF	44	0.824	0.872	0.854	0.691	0.792	0.808
RF	45	0.837	0.871	0.858	0.701	0.793	0.815
RF	46	0.841	0.875	0.862	0.709	0.799	0.820
RF	47	0.835	0.863	0.853	0.690	0.783	0.808

RF	48	0.826	0.870	0.853	0.690	0.789	0.807
RF	49	0.839	0.879	0.864	0.712	0.804	0.821
RF	50	0.837	0.877	0.862	0.709	0.802	0.819
RF	51	0.844	0.881	0.867	0.719	0.808	0.825
RF	52	0.841	0.871	0.860	0.705	0.794	0.817
RF	53	0.835	0.876	0.861	0.705	0.800	0.817
RF	54	0.841	0.867	0.857	0.700	0.789	0.814
RF	55	0.839	0.875	0.862	0.707	0.799	0.818
RF	56	0.850	0.880	0.869	0.723	0.808	0.828
RF	57	0.841	0.880	0.866	0.715	0.806	0.823
RF	58	0.837	0.887	0.868	0.720	0.814	0.825
RF	59	0.852	0.870	0.863	0.713	0.795	0.823
RF	60	0.863	0.883	0.876	0.738	0.813	0.838
RF	61	0.850	0.877	0.867	0.720	0.804	0.827
RF	62	0.846	0.874	0.863	0.712	0.798	0.821
RF	63	0.841	0.877	0.864	0.712	0.803	0.822
RF	64	0.857	0.879	0.871	0.727	0.807	0.831
RF	65	0.839	0.885	0.868	0.720	0.812	0.826
RF	66	0.859	0.887	0.876	0.739	0.818	0.838
RF	67	0.850	0.885	0.872	0.729	0.814	0.832
RF	68	0.852	0.880	0.870	0.725	0.808	0.830
RF	69	0.855	0.877	0.869	0.724	0.805	0.829
RF	70	0.846	0.888	0.872	0.729	0.817	0.831
RF	71	0.844	0.874	0.862	0.710	0.798	0.820
RF	72	0.859	0.877	0.871	0.728	0.806	0.832

RF	73	0.850	0.880	0.869	0.723	0.808	0.828
RF	74	0.855	0.881	0.871	0.728	0.810	0.832
RF	75	0.846	0.881	0.868	0.721	0.808	0.827
RF	76	0.852	0.876	0.867	0.720	0.803	0.827
RF	77	0.852	0.866	0.861	0.708	0.790	0.820
RF	78	0.844	0.877	0.865	0.714	0.803	0.823
RF	79	0.861	0.883	0.875	0.736	0.813	0.836
RF	80	0.848	0.883	0.870	0.724	0.811	0.829
RF	81	0.866	0.883	0.876	0.740	0.814	0.839
RF	82	0.841	0.883	0.867	0.719	0.809	0.825
RF	83	0.863	0.885	0.877	0.741	0.817	0.839
RF	84	0.857	0.890	0.878	0.741	0.822	0.839
RF	85	0.870	0.885	0.880	0.747	0.818	0.843
RF	86	0.855	0.885	0.874	0.733	0.815	0.834
RF	87	0.859	0.885	0.876	0.737	0.816	0.837
RF	88	0.857	0.885	0.875	0.735	0.816	0.836
RF	89	0.859	0.892	0.880	0.745	0.825	0.841
RF	90	0.859	0.884	0.875	0.735	0.814	0.836
RF	91	0.870	0.874	0.872	0.733	0.803	0.835
RF	92	0.848	0.897	0.879	0.742	0.830	0.839
RF	93	0.866	0.880	0.875	0.737	0.810	0.837
RF	94	0.874	0.883	0.880	0.747	0.815	0.844
RF	95	0.868	0.881	0.876	0.740	0.812	0.839
RF	96	0.870	0.893	0.885	0.756	0.828	0.849
RF	97	0.872	0.885	0.880	0.748	0.818	0.844

RF	98	0.872	0.880	0.877	0.742	0.811	0.841
RF	99	0.870	0.890	0.883	0.753	0.825	0.847
RF	100	0.859	0.879	0.871	0.729	0.807	0.832
RF	101	0.868	0.883	0.877	0.742	0.814	0.840
RF	102	0.857	0.885	0.875	0.735	0.816	0.836
RF	103	0.859	0.881	0.873	0.732	0.811	0.834
RF	104	0.861	0.875	0.870	0.726	0.803	0.831
RF	105	0.863	0.883	0.876	0.738	0.813	0.838
RF	106	0.866	0.880	0.875	0.737	0.810	0.837
RF	107	0.859	0.887	0.876	0.739	0.818	0.838
RF	108	0.863	0.888	0.879	0.744	0.820	0.841
RF	109	0.866	0.880	0.875	0.737	0.810	0.837
RF	110	0.850	0.885	0.872	0.729	0.814	0.832
RF	111	0.859	0.874	0.868	0.723	0.801	0.829
RF	112	0.870	0.881	0.877	0.742	0.813	0.840
RF	113	0.852	0.883	0.871	0.728	0.811	0.831
RF	114	0.857	0.885	0.875	0.735	0.816	0.836
RF	115	0.857	0.889	0.877	0.740	0.821	0.838
RF	116	0.866	0.885	0.878	0.743	0.817	0.841
RF	117	0.855	0.880	0.871	0.727	0.808	0.831
RF	118	0.870	0.892	0.884	0.754	0.826	0.848
RF	119	0.846	0.879	0.867	0.718	0.805	0.825
RF	120	0.855	0.883	0.872	0.730	0.812	0.833
RF	121	0.863	0.884	0.876	0.739	0.815	0.839
RF	122	0.859	0.871	0.867	0.720	0.798	0.827

RF	123	0.857	0.889	0.877	0.740	0.821	0.838
RF	124	0.866	0.877	0.873	0.733	0.807	0.835
RF	125	0.855	0.893	0.879	0.743	0.826	0.840
RF	126	0.863	0.883	0.876	0.738	0.813	0.838
RF	127	0.861	0.879	0.872	0.731	0.808	0.834
RF	128	0.870	0.880	0.876	0.740	0.811	0.840
RF	129	0.863	0.880	0.874	0.735	0.810	0.836
RF	130	0.872	0.883	0.879	0.745	0.815	0.843
RF	131	0.866	0.881	0.876	0.738	0.812	0.838
RF	132	0.859	0.893	0.880	0.746	0.826	0.842
RF	133	0.866	0.884	0.877	0.741	0.815	0.840
RF	134	0.861	0.885	0.876	0.739	0.816	0.838
RF	135	0.866	0.883	0.876	0.740	0.814	0.839
RF	136	0.868	0.885	0.879	0.745	0.817	0.842
RF	137	0.868	0.881	0.876	0.740	0.812	0.839
RF	138	0.868	0.888	0.880	0.748	0.821	0.844
RF	139	0.859	0.879	0.871	0.729	0.807	0.832
RF	140	0.872	0.881	0.878	0.744	0.813	0.842
RF	141	0.872	0.871	0.871	0.732	0.800	0.835
RF	142	0.874	0.894	0.887	0.761	0.831	0.852
RF	143	0.859	0.889	0.878	0.742	0.821	0.840
RF	144	0.872	0.875	0.874	0.736	0.805	0.837
RF	145	0.881	0.892	0.888	0.764	0.828	0.854
RF	146	0.861	0.880	0.873	0.733	0.810	0.835
RF	147	0.877	0.876	0.876	0.742	0.807	0.841

RF	148	0.863	0.881	0.875	0.736	0.812	0.837
RF	149	0.868	0.884	0.878	0.743	0.816	0.841
RF	150	0.859	0.888	0.877	0.740	0.819	0.839
RF	151	0.879	0.885	0.883	0.754	0.819	0.848
RF	152	0.866	0.889	0.880	0.747	0.822	0.843
RF	153	0.883	0.883	0.883	0.755	0.817	0.849
RF	154	0.863	0.875	0.871	0.728	0.803	0.832
RF	155	0.848	0.885	0.871	0.727	0.814	0.831
RF	156	0.870	0.883	0.878	0.743	0.814	0.841
RF	157	0.861	0.881	0.874	0.734	0.811	0.835
RF	158	0.859	0.888	0.877	0.740	0.819	0.839
RF	159	0.866	0.885	0.878	0.743	0.817	0.841
RF	160	0.868	0.889	0.881	0.749	0.823	0.845
RF	161	0.870	0.880	0.876	0.740	0.811	0.840
RF	162	0.861	0.879	0.872	0.731	0.808	0.834
RF	163	0.874	0.887	0.882	0.752	0.820	0.846
RF	164	0.868	0.877	0.874	0.735	0.807	0.837
RF	165	0.872	0.884	0.880	0.747	0.816	0.843
RF	166	0.868	0.875	0.872	0.732	0.804	0.835
RF	167	0.857	0.880	0.871	0.729	0.809	0.832
RF	168	0.850	0.889	0.875	0.734	0.820	0.835
RF	169	0.857	0.888	0.876	0.738	0.819	0.837
RF	170	0.852	0.885	0.873	0.731	0.815	0.833
RF	171	0.874	0.883	0.880	0.747	0.815	0.844
RF	172	0.877	0.872	0.874	0.737	0.802	0.838

RF	173	0.852	0.876	0.867	0.720	0.803	0.827
RF	174	0.863	0.879	0.873	0.733	0.808	0.835
RF	175	0.863	0.872	0.869	0.725	0.800	0.831
RF	176	0.868	0.877	0.874	0.735	0.807	0.837
RF	177	0.850	0.877	0.867	0.720	0.804	0.827
RF	178	0.855	0.884	0.873	0.732	0.813	0.834
RF	179	0.852	0.876	0.867	0.720	0.803	0.827
RF	180	0.861	0.889	0.879	0.744	0.821	0.841
RF	181	0.868	0.880	0.876	0.738	0.811	0.838
RF	182	0.861	0.877	0.871	0.730	0.806	0.833
RF	183	0.850	0.876	0.867	0.718	0.802	0.826
RF	184	0.859	0.883	0.874	0.734	0.813	0.835
RF	185	0.859	0.887	0.876	0.739	0.818	0.838
RF	186	0.872	0.876	0.875	0.738	0.807	0.838
RF	187	0.870	0.883	0.878	0.743	0.814	0.841
RF	188	0.872	0.877	0.876	0.739	0.808	0.839
RF	189	0.874	0.884	0.880	0.749	0.817	0.845
RF	190	0.868	0.883	0.877	0.742	0.814	0.840
RF	191	0.866	0.890	0.881	0.749	0.824	0.844
RF	192	0.874	0.883	0.880	0.747	0.815	0.844
RF	193	0.861	0.876	0.871	0.728	0.805	0.832
RF	194	0.861	0.879	0.872	0.731	0.808	0.834
RF	195	0.866	0.888	0.880	0.746	0.820	0.842
RF	196	0.872	0.880	0.877	0.742	0.811	0.841
RF	197	0.874	0.871	0.872	0.733	0.800	0.836

RF	198	0.866	0.885	0.878	0.743	0.817	0.841
RF	199	0.861	0.880	0.873	0.733	0.810	0.835
RF	200	0.857	0.885	0.875	0.735	0.816	0.836
RF	201	0.874	0.879	0.877	0.743	0.810	0.841
RF	202	0.866	0.880	0.875	0.737	0.810	0.837
RF	203	0.850	0.884	0.871	0.728	0.813	0.831
RF	204	0.857	0.884	0.874	0.734	0.814	0.835
RF	205	0.848	0.877	0.867	0.718	0.804	0.825
RF	206	0.848	0.881	0.869	0.723	0.809	0.828
RF	207	0.848	0.876	0.866	0.717	0.802	0.824
RF	208	0.852	0.876	0.867	0.720	0.803	0.827
RF	209	0.841	0.876	0.863	0.711	0.801	0.821
RF	210	0.855	0.881	0.871	0.728	0.810	0.832
RF	211	0.846	0.876	0.865	0.715	0.802	0.823
RF	212	0.855	0.883	0.872	0.730	0.812	0.833
RF	213	0.855	0.879	0.870	0.725	0.807	0.830
RF	214	0.863	0.870	0.867	0.722	0.797	0.829
RF	215	0.861	0.879	0.872	0.731	0.808	0.834
RF	216	0.850	0.880	0.869	0.723	0.808	0.828
RF	217	0.863	0.883	0.876	0.738	0.813	0.838
RF	218	0.866	0.881	0.876	0.738	0.812	0.838
RF	219	0.870	0.887	0.880	0.748	0.820	0.844
RF	220	0.857	0.864	0.862	0.710	0.789	0.822
RF	221	0.868	0.872	0.871	0.729	0.801	0.833
RF	222	0.859	0.881	0.873	0.732	0.811	0.834

RF	223	0.870	0.880	0.876	0.740	0.811	0.840
RF	224	0.848	0.881	0.869	0.723	0.809	0.828
RF	225	0.859	0.880	0.872	0.731	0.809	0.833
RF	226	0.872	0.877	0.876	0.739	0.808	0.839
RF	227	0.866	0.881	0.876	0.738	0.812	0.838
RF	228	0.859	0.877	0.871	0.728	0.806	0.832
RF	229	0.857	0.880	0.871	0.729	0.809	0.832
RF	230	0.863	0.876	0.871	0.730	0.805	0.833
RF	231	0.857	0.884	0.874	0.734	0.814	0.835
RF	232	0.857	0.881	0.872	0.730	0.810	0.833
RF	233	0.855	0.875	0.867	0.721	0.802	0.827
RF	234	0.848	0.881	0.869	0.723	0.809	0.828
RF	235	0.852	0.864	0.860	0.707	0.788	0.819
RF	236	0.866	0.884	0.877	0.741	0.815	0.840
RF	237	0.852	0.875	0.867	0.719	0.801	0.826
RF	238	0.866	0.874	0.871	0.729	0.802	0.833
RF	239	0.848	0.880	0.868	0.721	0.807	0.827
RF	240	0.850	0.877	0.867	0.720	0.804	0.827
RF	241	0.850	0.874	0.865	0.715	0.799	0.824
RF	242	0.850	0.883	0.871	0.726	0.811	0.830
RF	243	0.850	0.866	0.860	0.706	0.789	0.819
RF	244	0.863	0.876	0.871	0.730	0.805	0.833
RF	245	0.870	0.890	0.883	0.753	0.825	0.847
RF	246	0.846	0.859	0.854	0.695	0.780	0.812
RF	247	0.852	0.879	0.869	0.723	0.806	0.829

RF	248	0.841	0.880	0.866	0.715	0.806	0.823
RF	249	0.859	0.880	0.872	0.731	0.809	0.833
RF	250	0.841	0.876	0.863	0.711	0.801	0.821
RF	251	0.839	0.885	0.868	0.720	0.812	0.826
RF	252	0.837	0.871	0.858	0.701	0.793	0.815
RF	253	0.844	0.880	0.867	0.717	0.806	0.825
RF	254	0.848	0.883	0.870	0.724	0.811	0.829
RF	255	0.846	0.883	0.869	0.722	0.810	0.828
RF	256	0.848	0.872	0.863	0.712	0.797	0.822
RF	257	0.830	0.877	0.860	0.703	0.800	0.815
RF	258	0.839	0.876	0.862	0.709	0.800	0.819
RF	259	0.848	0.867	0.860	0.706	0.791	0.818
RF	260	0.839	0.884	0.867	0.718	0.811	0.825
RF	261	0.848	0.877	0.867	0.718	0.804	0.825
RF	262	0.850	0.884	0.871	0.728	0.813	0.831
RF	263	0.848	0.871	0.862	0.710	0.795	0.821
RF	264	0.837	0.879	0.863	0.710	0.803	0.820
RF	265	0.826	0.875	0.857	0.696	0.796	0.811
RF	266	0.833	0.870	0.856	0.695	0.791	0.811
RF	267	0.835	0.871	0.857	0.699	0.793	0.813
RF	268	0.846	0.881	0.868	0.721	0.808	0.827
RF	269	0.852	0.877	0.868	0.722	0.805	0.828
RF	270	0.839	0.875	0.862	0.707	0.799	0.818
RF	271	0.817	0.877	0.855	0.691	0.798	0.807
RF	272	0.835	0.874	0.859	0.702	0.796	0.815

RF	273	0.824	0.887	0.863	0.708	0.811	0.817
RF	274	0.828	0.876	0.858	0.699	0.798	0.813
RF	275	0.835	0.872	0.858	0.700	0.795	0.814
RF	276	0.833	0.877	0.861	0.705	0.801	0.816
RF	277	0.841	0.877	0.864	0.712	0.803	0.822
RF	278	0.826	0.883	0.862	0.705	0.806	0.816
RF	279	0.846	0.875	0.864	0.713	0.800	0.822
RF	280	0.841	0.881	0.867	0.717	0.808	0.824
RF	281	0.841	0.868	0.858	0.701	0.791	0.815
RF	282	0.817	0.879	0.856	0.693	0.800	0.808
RF	283	0.824	0.879	0.858	0.699	0.801	0.812
RF	284	0.830	0.881	0.862	0.708	0.806	0.818
RF	285	0.833	0.883	0.864	0.711	0.808	0.820
RF	286	0.837	0.877	0.862	0.709	0.802	0.819
RF	287	0.839	0.874	0.861	0.706	0.797	0.818
RF	288	0.826	0.872	0.855	0.693	0.793	0.809
RF	289	0.824	0.872	0.854	0.691	0.792	0.808
RF	290	0.828	0.877	0.859	0.701	0.800	0.814
RF	291	0.841	0.880	0.866	0.715	0.806	0.823
RF	292	0.826	0.884	0.862	0.707	0.808	0.817
RF	293	0.830	0.879	0.861	0.704	0.802	0.816
RF	294	0.824	0.875	0.856	0.694	0.796	0.810
RF	295	0.846	0.872	0.862	0.710	0.797	0.821
RF	296	0.839	0.872	0.860	0.704	0.795	0.817
RF	297	0.839	0.879	0.864	0.712	0.804	0.821

RF	298	0.833	0.874	0.858	0.700	0.796	0.814
RF	299	0.835	0.876	0.861	0.705	0.800	0.817
RF	300	0.846	0.870	0.861	0.707	0.793	0.819
RF	301	0.846	0.872	0.862	0.710	0.797	0.821
RF	302	0.839	0.872	0.860	0.704	0.795	0.817
RF	303	0.839	0.881	0.866	0.715	0.807	0.823
RF	304	0.837	0.883	0.866	0.715	0.809	0.823
RF	305	0.833	0.876	0.860	0.703	0.799	0.816
RF	306	0.833	0.871	0.857	0.697	0.792	0.812
RF	307	0.839	0.883	0.867	0.717	0.809	0.824
RF	308	0.844	0.875	0.863	0.711	0.800	0.821
RF	309	0.830	0.880	0.862	0.706	0.804	0.817
RF	310	0.837	0.872	0.859	0.702	0.795	0.815
RF	311	0.848	0.876	0.866	0.717	0.802	0.824
RF	312	0.833	0.876	0.860	0.703	0.799	0.816
RF	313	0.841	0.879	0.865	0.714	0.804	0.822
RF	314	0.846	0.870	0.861	0.707	0.793	0.819
RF	315	0.828	0.880	0.861	0.704	0.803	0.816
RF	316	0.835	0.879	0.862	0.708	0.803	0.819
RF	317	0.839	0.871	0.859	0.703	0.794	0.816
RF	318	0.822	0.874	0.854	0.690	0.794	0.807
RF	319	0.815	0.887	0.860	0.701	0.810	0.812
RF	320	0.837	0.868	0.857	0.698	0.790	0.813
RF	321	0.830	0.866	0.853	0.689	0.785	0.807
RF	322	0.837	0.880	0.864	0.712	0.805	0.821

RF	323	0.837	0.868	0.857	0.698	0.790	0.813
RF	324	0.826	0.877	0.858	0.699	0.800	0.813
RF	325	0.839	0.880	0.865	0.714	0.805	0.822
RF	326	0.844	0.872	0.862	0.708	0.796	0.819
RF	327	0.835	0.871	0.857	0.699	0.793	0.813
RF	328	0.819	0.871	0.852	0.685	0.790	0.804
RF	329	0.817	0.874	0.853	0.687	0.793	0.805
RF	330	0.852	0.872	0.865	0.716	0.798	0.824
RF	331	0.828	0.867	0.853	0.688	0.787	0.807
RF	332	0.844	0.871	0.861	0.706	0.795	0.818
RF	333	0.835	0.880	0.863	0.710	0.805	0.819
RF	334	0.841	0.871	0.860	0.705	0.794	0.817
RF	335	0.844	0.876	0.864	0.713	0.801	0.822
RF	336	0.839	0.884	0.867	0.718	0.811	0.825
RF	337	0.826	0.876	0.857	0.697	0.798	0.812
RF	338	0.850	0.874	0.865	0.715	0.799	0.824
RF	339	0.839	0.880	0.865	0.714	0.805	0.822
RF	340	0.841	0.870	0.859	0.703	0.793	0.816
RF	341	0.852	0.874	0.866	0.717	0.800	0.825
RF	342	0.824	0.874	0.855	0.692	0.794	0.809
RF	343	0.830	0.866	0.853	0.689	0.785	0.807
RF	344	0.828	0.880	0.861	0.704	0.803	0.816
RF	345	0.833	0.876	0.860	0.703	0.799	0.816
RF	346	0.852	0.884	0.872	0.730	0.813	0.832
RF	347	0.833	0.864	0.853	0.689	0.784	0.808

RF	348	0.824	0.863	0.848	0.680	0.781	0.802
RF	349	0.846	0.870	0.861	0.707	0.793	0.819
RF	350	0.846	0.879	0.867	0.718	0.805	0.825
RF	351	0.822	0.877	0.857	0.695	0.799	0.810
RF	352	0.824	0.874	0.855	0.692	0.794	0.809
RF	353	0.828	0.870	0.854	0.691	0.790	0.809
RF	354	0.837	0.875	0.861	0.705	0.798	0.817
RF	355	0.830	0.871	0.856	0.695	0.792	0.811
RF	356	0.844	0.875	0.863	0.711	0.800	0.821
RF	357	0.817	0.868	0.849	0.680	0.786	0.801
RF	358	0.819	0.872	0.853	0.687	0.791	0.805
RF	359	0.826	0.863	0.849	0.682	0.781	0.803
RF	360	0.837	0.867	0.856	0.696	0.788	0.812
RF	361	0.841	0.874	0.862	0.708	0.797	0.819
RF	362	0.830	0.874	0.857	0.698	0.795	0.813
RF	363	0.819	0.877	0.856	0.693	0.798	0.809
RF	364	0.826	0.879	0.859	0.701	0.801	0.813
RF	365	0.830	0.885	0.865	0.712	0.811	0.820
RF	366	0.822	0.885	0.862	0.705	0.809	0.815
RF	367	0.828	0.879	0.860	0.702	0.802	0.815
RF	368	0.830	0.872	0.857	0.697	0.794	0.812
RF	369	0.819	0.872	0.853	0.687	0.791	0.805
RF	370	0.826	0.874	0.856	0.694	0.794	0.810
SVM	1	0.764	0.808	0.792	0.564	0.702	0.732
SVM	2	0.767	0.817	0.799	0.576	0.713	0.739

SVM	3	0.771	0.828	0.807	0.592	0.726	0.748
SVM	4	0.780	0.834	0.814	0.607	0.736	0.757
SVM	5	0.775	0.834	0.812	0.604	0.735	0.755
SVM	6	0.773	0.842	0.817	0.611	0.744	0.758
SVM	7	0.762	0.846	0.815	0.606	0.746	0.754
SVM	8	0.738	0.846	0.806	0.584	0.740	0.739
SVM	9	0.747	0.847	0.810	0.594	0.743	0.745
SVM	10	0.758	0.840	0.809	0.594	0.737	0.747
SVM	11	0.762	0.846	0.815	0.606	0.746	0.754
SVM	12	0.751	0.841	0.808	0.590	0.737	0.744
SVM	13	0.740	0.844	0.805	0.583	0.737	0.738
SVM	14	0.744	0.850	0.811	0.595	0.746	0.745
SVM	15	0.742	0.850	0.810	0.593	0.746	0.744
SVM	16	0.733	0.845	0.803	0.579	0.737	0.735
SVM	17	0.729	0.842	0.800	0.572	0.732	0.731
SVM	18	0.749	0.855	0.816	0.605	0.754	0.751
SVM	19	0.731	0.862	0.813	0.598	0.758	0.744
SVM	20	0.740	0.857	0.813	0.599	0.753	0.747
SVM	21	0.733	0.855	0.810	0.592	0.750	0.742
SVM	22	0.725	0.851	0.804	0.579	0.743	0.734
SVM	23	0.751	0.857	0.817	0.609	0.756	0.754
SVM	24	0.767	0.842	0.814	0.605	0.742	0.754
SVM	25	0.751	0.838	0.806	0.587	0.733	0.742
SVM	26	0.744	0.849	0.810	0.593	0.744	0.744
SVM	27	0.751	0.850	0.813	0.601	0.748	0.749

SVM	28	0.742	0.849	0.809	0.591	0.744	0.743
SVM	29	0.756	0.857	0.819	0.612	0.757	0.756
SVM	30	0.740	0.851	0.810	0.593	0.747	0.743
SVM	31	0.760	0.853	0.818	0.612	0.753	0.757
SVM	32	0.780	0.862	0.831	0.640	0.770	0.775
SVM	33	0.811	0.845	0.832	0.647	0.756	0.782
SVM	34	0.804	0.854	0.835	0.652	0.765	0.784
SVM	35	0.795	0.853	0.831	0.642	0.762	0.778
SVM	36	0.802	0.854	0.835	0.650	0.765	0.783
SVM	37	0.808	0.851	0.835	0.653	0.763	0.785
SVM	38	0.808	0.851	0.835	0.653	0.763	0.785
SVM	39	0.815	0.859	0.843	0.668	0.774	0.794
SVM	40	0.822	0.870	0.852	0.686	0.789	0.805
SVM	41	0.813	0.862	0.844	0.669	0.777	0.794
SVM	42	0.808	0.862	0.842	0.665	0.776	0.792
SVM	43	0.813	0.859	0.842	0.666	0.774	0.793
SVM	44	0.813	0.858	0.841	0.664	0.772	0.792
SVM	45	0.811	0.858	0.840	0.662	0.771	0.791
SVM	46	0.819	0.858	0.844	0.670	0.773	0.796
SVM	47	0.815	0.866	0.847	0.675	0.782	0.798
SVM	48	0.815	0.859	0.843	0.668	0.774	0.794
SVM	49	0.813	0.862	0.844	0.669	0.777	0.794
SVM	50	0.813	0.860	0.843	0.667	0.775	0.794
SVM	51	0.808	0.863	0.843	0.666	0.778	0.793
SVM	52	0.811	0.868	0.847	0.675	0.785	0.797

SVM	53	0.802	0.862	0.839	0.659	0.774	0.788
SVM	54	0.806	0.859	0.839	0.660	0.772	0.789
SVM	55	0.828	0.868	0.853	0.690	0.788	0.808
SVM	56	0.837	0.866	0.855	0.695	0.787	0.811
SVM	57	0.828	0.875	0.857	0.698	0.797	0.812
SVM	58	0.830	0.867	0.853	0.690	0.787	0.808
SVM	59	0.828	0.870	0.854	0.691	0.790	0.809
SVM	60	0.828	0.872	0.856	0.695	0.793	0.810
SVM	61	0.833	0.867	0.854	0.692	0.788	0.809
SVM	62	0.835	0.860	0.851	0.686	0.780	0.806
SVM	63	0.835	0.862	0.852	0.688	0.781	0.807
SVM	64	0.846	0.872	0.862	0.710	0.797	0.821
SVM	65	0.846	0.872	0.862	0.710	0.797	0.821
SVM	66	0.844	0.867	0.858	0.702	0.790	0.816
SVM	67	0.846	0.866	0.858	0.702	0.789	0.816
SVM	68	0.846	0.866	0.858	0.702	0.789	0.816
SVM	69	0.846	0.866	0.858	0.702	0.789	0.816
SVM	70	0.848	0.868	0.861	0.707	0.792	0.819
SVM	71	0.848	0.868	0.861	0.707	0.792	0.819
SVM	72	0.841	0.867	0.857	0.700	0.789	0.814
SVM	73	0.846	0.871	0.862	0.708	0.795	0.820
SVM	74	0.846	0.875	0.864	0.713	0.800	0.822
SVM	75	0.841	0.871	0.860	0.705	0.794	0.817
SVM	76	0.839	0.871	0.859	0.703	0.794	0.816
SVM	77	0.835	0.870	0.857	0.697	0.791	0.812

SVM	78	0.826	0.868	0.853	0.688	0.788	0.806
SVM	79	0.824	0.867	0.851	0.685	0.786	0.804
SVM	80	0.848	0.863	0.857	0.701	0.786	0.816
SVM	81	0.850	0.871	0.863	0.712	0.796	0.822
SVM	82	0.852	0.867	0.862	0.710	0.791	0.821
SVM	83	0.848	0.872	0.863	0.712	0.797	0.822
SVM	84	0.846	0.887	0.871	0.727	0.815	0.830
SVM	85	0.846	0.888	0.872	0.729	0.817	0.831
SVM	86	0.846	0.887	0.871	0.727	0.815	0.830
SVM	87	0.846	0.883	0.869	0.722	0.810	0.828
SVM	88	0.846	0.883	0.869	0.722	0.810	0.828
SVM	89	0.846	0.880	0.867	0.719	0.807	0.826
SVM	90	0.852	0.879	0.869	0.723	0.806	0.829
SVM	91	0.841	0.870	0.859	0.703	0.793	0.816
SVM	92	0.839	0.877	0.863	0.710	0.802	0.820
SVM	93	0.844	0.880	0.867	0.717	0.806	0.825
SVM	94	0.841	0.880	0.866	0.715	0.806	0.823
SVM	95	0.837	0.894	0.873	0.729	0.824	0.831
SVM	96	0.839	0.894	0.874	0.731	0.825	0.832
SVM	97	0.837	0.889	0.870	0.723	0.817	0.827
SVM	98	0.835	0.896	0.873	0.729	0.826	0.830
SVM	99	0.841	0.888	0.871	0.725	0.816	0.829
SVM	100	0.837	0.889	0.870	0.723	0.817	0.827
SVM	101	0.837	0.875	0.861	0.705	0.798	0.817
SVM	102	0.833	0.872	0.857	0.698	0.794	0.813

SVM	103	0.833	0.872	0.857	0.698	0.794	0.813
SVM	104	0.833	0.872	0.857	0.698	0.794	0.813
SVM	105	0.837	0.874	0.860	0.704	0.797	0.816
SVM	106	0.839	0.875	0.862	0.707	0.799	0.818
SVM	107	0.841	0.879	0.865	0.714	0.804	0.822
SVM	108	0.833	0.875	0.859	0.702	0.797	0.815
SVM	109	0.837	0.876	0.862	0.707	0.800	0.818
SVM	110	0.835	0.872	0.858	0.700	0.795	0.814
SVM	111	0.835	0.877	0.862	0.707	0.801	0.818
SVM	112	0.833	0.879	0.862	0.706	0.803	0.817
SVM	113	0.833	0.877	0.861	0.705	0.801	0.816
SVM	114	0.837	0.874	0.860	0.704	0.797	0.816
SVM	115	0.830	0.876	0.859	0.701	0.799	0.814
SVM	116	0.841	0.853	0.848	0.683	0.772	0.805
SVM	117	0.852	0.851	0.852	0.691	0.772	0.810
SVM	118	0.852	0.851	0.852	0.691	0.772	0.810
SVM	119	0.848	0.855	0.853	0.692	0.776	0.811
SVM	120	0.848	0.857	0.853	0.694	0.778	0.811
SVM	121	0.844	0.853	0.849	0.685	0.772	0.806
SVM	122	0.844	0.850	0.848	0.682	0.769	0.805
SVM	123	0.837	0.850	0.845	0.676	0.768	0.801
SVM	124	0.837	0.851	0.846	0.678	0.769	0.802
SVM	125	0.833	0.855	0.847	0.678	0.773	0.802
SVM	126	0.833	0.867	0.854	0.692	0.788	0.809
SVM	127	0.835	0.868	0.856	0.696	0.790	0.812

SVM	128	0.835	0.867	0.855	0.694	0.788	0.811
SVM	129	0.837	0.868	0.857	0.698	0.790	0.813
SVM	130	0.833	0.866	0.853	0.691	0.786	0.809
SVM	131	0.833	0.870	0.856	0.695	0.791	0.811
SVM	132	0.835	0.866	0.854	0.693	0.786	0.810
SVM	133	0.844	0.864	0.857	0.699	0.786	0.814
SVM	134	0.844	0.866	0.857	0.700	0.788	0.815
SVM	135	0.841	0.858	0.852	0.689	0.778	0.808
SVM	136	0.846	0.874	0.863	0.712	0.798	0.821
SVM	137	0.844	0.876	0.864	0.713	0.801	0.822
SVM	138	0.844	0.879	0.866	0.716	0.805	0.824
SVM	139	0.844	0.879	0.866	0.716	0.805	0.824
SVM	140	0.848	0.879	0.867	0.720	0.805	0.826
SVM	141	0.872	0.877	0.876	0.739	0.808	0.839
SVM	142	0.877	0.880	0.879	0.746	0.812	0.843
SVM	143	0.883	0.879	0.880	0.750	0.812	0.846
SVM	144	0.881	0.879	0.880	0.748	0.811	0.845
SVM	145	0.881	0.879	0.880	0.748	0.811	0.845
SVM	146	0.872	0.880	0.877	0.742	0.811	0.841
SVM	147	0.872	0.880	0.877	0.742	0.811	0.841
SVM	148	0.874	0.879	0.877	0.743	0.810	0.841
SVM	149	0.874	0.879	0.877	0.743	0.810	0.841
SVM	150	0.881	0.870	0.874	0.738	0.800	0.839
SVM	151	0.879	0.872	0.875	0.739	0.803	0.839
SVM	152	0.877	0.871	0.873	0.735	0.801	0.837

SVM	153	0.877	0.871	0.873	0.735	0.801	0.837
SVM	154	0.872	0.876	0.875	0.738	0.807	0.838
SVM	155	0.868	0.877	0.874	0.735	0.807	0.837
SVM	156	0.868	0.880	0.876	0.738	0.811	0.838
SVM	157	0.870	0.880	0.876	0.740	0.811	0.840
SVM	158	0.863	0.872	0.869	0.725	0.800	0.831
SVM	159	0.861	0.870	0.867	0.720	0.796	0.828
SVM	160	0.863	0.872	0.869	0.725	0.800	0.831
SVM	161	0.863	0.872	0.869	0.725	0.800	0.831
SVM	162	0.868	0.875	0.872	0.732	0.804	0.835
SVM	163	0.866	0.876	0.872	0.732	0.805	0.834
SVM	164	0.866	0.875	0.871	0.730	0.804	0.834
SVM	165	0.868	0.877	0.874	0.735	0.807	0.837
SVM	166	0.850	0.896	0.879	0.742	0.828	0.839
SVM	167	0.850	0.896	0.879	0.742	0.828	0.839
SVM	168	0.848	0.897	0.879	0.742	0.830	0.839
SVM	169	0.850	0.897	0.880	0.744	0.830	0.840
SVM	170	0.848	0.896	0.878	0.740	0.828	0.838
SVM	171	0.848	0.896	0.878	0.740	0.828	0.838
SVM	172	0.848	0.890	0.875	0.734	0.821	0.834
SVM	173	0.848	0.890	0.875	0.734	0.821	0.834
SVM	174	0.848	0.893	0.876	0.737	0.824	0.836
SVM	175	0.850	0.893	0.877	0.739	0.825	0.837
SVM	176	0.852	0.889	0.876	0.736	0.820	0.836
SVM	177	0.850	0.893	0.877	0.739	0.825	0.837

SVM	178	0.846	0.893	0.876	0.735	0.824	0.835
SVM	179	0.846	0.893	0.876	0.735	0.824	0.835
SVM	180	0.848	0.892	0.876	0.735	0.823	0.835
SVM	181	0.848	0.892	0.876	0.735	0.823	0.835
SVM	182	0.848	0.892	0.876	0.735	0.823	0.835
SVM	183	0.852	0.888	0.875	0.734	0.818	0.835
SVM	184	0.852	0.888	0.875	0.734	0.818	0.835
SVM	185	0.848	0.888	0.873	0.731	0.817	0.832
SVM	186	0.852	0.889	0.876	0.736	0.820	0.836
SVM	187	0.857	0.887	0.876	0.737	0.817	0.837
SVM	188	0.859	0.888	0.877	0.740	0.819	0.839
SVM	189	0.857	0.887	0.876	0.737	0.817	0.837
SVM	190	0.859	0.880	0.872	0.731	0.809	0.833
SVM	191	0.857	0.877	0.870	0.726	0.805	0.830
SVM	192	0.855	0.876	0.868	0.722	0.803	0.828
SVM	193	0.859	0.871	0.867	0.720	0.798	0.827
SVM	194	0.863	0.870	0.867	0.722	0.797	0.829
SVM	195	0.863	0.870	0.867	0.722	0.797	0.829
SVM	196	0.866	0.872	0.870	0.727	0.800	0.832
SVM	197	0.861	0.871	0.867	0.722	0.798	0.828
SVM	198	0.868	0.872	0.871	0.729	0.801	0.833
SVM	199	0.872	0.875	0.874	0.736	0.805	0.837
SVM	200	0.868	0.874	0.871	0.731	0.802	0.834
SVM	201	0.866	0.874	0.871	0.729	0.802	0.833
SVM	202	0.863	0.876	0.871	0.730	0.805	0.833

SVM	203	0.859	0.872	0.867	0.721	0.799	0.828
SVM	204	0.852	0.877	0.868	0.722	0.805	0.828
SVM	205	0.863	0.874	0.870	0.727	0.802	0.831
SVM	206	0.861	0.872	0.868	0.723	0.800	0.829
SVM	207	0.861	0.871	0.867	0.722	0.798	0.828
SVM	208	0.861	0.870	0.867	0.720	0.796	0.828
SVM	209	0.859	0.870	0.866	0.718	0.796	0.826
SVM	210	0.861	0.868	0.866	0.719	0.795	0.827
SVM	211	0.863	0.871	0.868	0.724	0.798	0.830
SVM	212	0.863	0.875	0.871	0.728	0.803	0.832
SVM	213	0.861	0.877	0.871	0.730	0.806	0.833
SVM	214	0.852	0.879	0.869	0.723	0.806	0.829
SVM	215	0.859	0.881	0.873	0.732	0.811	0.834
SVM	216	0.859	0.879	0.871	0.729	0.807	0.832
SVM	217	0.857	0.879	0.871	0.727	0.807	0.831
SVM	218	0.855	0.877	0.869	0.724	0.805	0.829
SVM	219	0.857	0.875	0.868	0.723	0.802	0.829
SVM	220	0.859	0.872	0.867	0.721	0.799	0.828
SVM	221	0.859	0.877	0.871	0.728	0.806	0.832
SVM	222	0.859	0.879	0.871	0.729	0.807	0.832
SVM	223	0.861	0.870	0.867	0.720	0.796	0.828
SVM	224	0.861	0.872	0.868	0.723	0.800	0.829
SVM	225	0.859	0.871	0.867	0.720	0.798	0.827
SVM	226	0.868	0.872	0.871	0.729	0.801	0.833
SVM	227	0.868	0.871	0.870	0.728	0.799	0.832

SVM	228	0.868	0.872	0.871	0.729	0.801	0.833
SVM	229	0.868	0.872	0.871	0.729	0.801	0.833
SVM	230	0.868	0.872	0.871	0.729	0.801	0.833
SVM	231	0.868	0.872	0.871	0.729	0.801	0.833
SVM	232	0.868	0.871	0.870	0.728	0.799	0.832
SVM	233	0.861	0.871	0.867	0.722	0.798	0.828
SVM	234	0.863	0.870	0.867	0.722	0.797	0.829
SVM	235	0.863	0.868	0.867	0.721	0.795	0.828
SVM	236	0.861	0.870	0.867	0.720	0.796	0.828
SVM	237	0.861	0.868	0.866	0.719	0.795	0.827
SVM	238	0.859	0.868	0.865	0.717	0.794	0.825
SVM	239	0.835	0.866	0.854	0.693	0.786	0.810
SVM	240	0.852	0.872	0.865	0.716	0.798	0.824
SVM	241	0.857	0.870	0.865	0.717	0.796	0.825
SVM	242	0.855	0.868	0.863	0.713	0.793	0.823
SVM	243	0.855	0.867	0.862	0.712	0.792	0.822
SVM	244	0.855	0.867	0.862	0.712	0.792	0.822
SVM	245	0.850	0.875	0.866	0.717	0.801	0.825
SVM	246	0.852	0.876	0.867	0.720	0.803	0.827
SVM	247	0.852	0.876	0.867	0.720	0.803	0.827
SVM	248	0.852	0.876	0.867	0.720	0.803	0.827
SVM	249	0.850	0.876	0.867	0.718	0.802	0.826
SVM	250	0.835	0.874	0.859	0.702	0.796	0.815
SVM	251	0.837	0.870	0.857	0.699	0.792	0.814
SVM	252	0.839	0.871	0.859	0.703	0.794	0.816

SVM	253	0.852	0.863	0.859	0.705	0.787	0.818
SVM	254	0.850	0.862	0.857	0.702	0.785	0.816
SVM	255	0.850	0.862	0.857	0.702	0.785	0.816
SVM	256	0.850	0.860	0.857	0.700	0.783	0.815
SVM	257	0.850	0.863	0.858	0.703	0.786	0.817
SVM	258	0.855	0.858	0.857	0.701	0.781	0.816
SVM	259	0.855	0.858	0.857	0.701	0.781	0.816
SVM	260	0.855	0.859	0.857	0.702	0.782	0.817
SVM	261	0.855	0.860	0.858	0.704	0.784	0.818
SVM	262	0.852	0.863	0.859	0.705	0.787	0.818
SVM	263	0.855	0.860	0.858	0.704	0.784	0.818
SVM	264	0.855	0.860	0.858	0.704	0.784	0.818
SVM	265	0.844	0.864	0.857	0.699	0.786	0.814
SVM	266	0.841	0.864	0.856	0.697	0.786	0.813
SVM	267	0.839	0.867	0.857	0.698	0.789	0.813
SVM	268	0.844	0.866	0.857	0.700	0.788	0.815
SVM	269	0.839	0.863	0.854	0.693	0.784	0.811
SVM	270	0.839	0.862	0.853	0.692	0.782	0.810
SVM	271	0.841	0.862	0.854	0.694	0.783	0.811
SVM	272	0.844	0.863	0.856	0.697	0.785	0.813
SVM	273	0.839	0.867	0.857	0.698	0.789	0.813
SVM	274	0.839	0.866	0.856	0.696	0.787	0.812
SVM	275	0.841	0.862	0.854	0.694	0.783	0.811
SVM	276	0.841	0.862	0.854	0.694	0.783	0.811
SVM	277	0.839	0.866	0.856	0.696	0.787	0.812

SVM	278	0.844	0.866	0.857	0.700	0.788	0.815
SVM	279	0.846	0.864	0.857	0.701	0.787	0.815
SVM	280	0.846	0.864	0.857	0.701	0.787	0.815
SVM	281	0.839	0.864	0.855	0.695	0.786	0.812
SVM	282	0.839	0.867	0.857	0.698	0.789	0.813
SVM	283	0.839	0.867	0.857	0.698	0.789	0.813
SVM	284	0.839	0.867	0.857	0.698	0.789	0.813
SVM	285	0.852	0.860	0.857	0.702	0.783	0.816
SVM	286	0.850	0.860	0.857	0.700	0.783	0.815
SVM	287	0.850	0.860	0.857	0.700	0.783	0.815
SVM	288	0.850	0.860	0.857	0.700	0.783	0.815
SVM	289	0.852	0.860	0.857	0.702	0.783	0.816
SVM	290	0.852	0.859	0.857	0.700	0.782	0.816
SVM	291	0.841	0.863	0.855	0.695	0.784	0.812
SVM	292	0.841	0.863	0.855	0.695	0.784	0.812
SVM	293	0.848	0.860	0.856	0.698	0.783	0.814
SVM	294	0.848	0.860	0.856	0.698	0.783	0.814
SVM	295	0.850	0.860	0.857	0.700	0.783	0.815
SVM	296	0.852	0.860	0.857	0.702	0.783	0.816
SVM	297	0.844	0.867	0.858	0.702	0.790	0.816
SVM	298	0.844	0.866	0.857	0.700	0.788	0.815
SVM	299	0.846	0.868	0.860	0.705	0.792	0.818
SVM	300	0.846	0.868	0.860	0.705	0.792	0.818
SVM	301	0.846	0.868	0.860	0.705	0.792	0.818
SVM	302	0.844	0.864	0.857	0.699	0.786	0.814

SVM	303	0.844	0.864	0.857	0.699	0.786	0.814
SVM	304	0.844	0.864	0.857	0.699	0.786	0.814
SVM	305	0.844	0.863	0.856	0.697	0.785	0.813
SVM	306	0.844	0.863	0.856	0.697	0.785	0.813
SVM	307	0.846	0.863	0.857	0.699	0.785	0.814
SVM	308	0.846	0.863	0.857	0.699	0.785	0.814
SVM	309	0.844	0.864	0.857	0.699	0.786	0.814
SVM	310	0.844	0.864	0.857	0.699	0.786	0.814
SVM	311	0.844	0.864	0.857	0.699	0.786	0.814
SVM	312	0.844	0.864	0.857	0.699	0.786	0.814
SVM	313	0.841	0.863	0.855	0.695	0.784	0.812
SVM	314	0.841	0.863	0.855	0.695	0.784	0.812
SVM	315	0.839	0.864	0.855	0.695	0.786	0.812
SVM	316	0.841	0.863	0.855	0.695	0.784	0.812
SVM	317	0.846	0.868	0.860	0.705	0.792	0.818
SVM	318	0.846	0.870	0.861	0.707	0.793	0.819
SVM	319	0.846	0.868	0.860	0.705	0.792	0.818
SVM	320	0.846	0.870	0.861	0.707	0.793	0.819
SVM	321	0.846	0.870	0.861	0.707	0.793	0.819
SVM	322	0.848	0.864	0.858	0.703	0.787	0.817
SVM	323	0.846	0.864	0.857	0.701	0.787	0.815
SVM	324	0.846	0.864	0.857	0.701	0.787	0.815
SVM	325	0.846	0.866	0.858	0.702	0.789	0.816
SVM	326	0.846	0.866	0.858	0.702	0.789	0.816
SVM	327	0.844	0.866	0.857	0.700	0.788	0.815

SVM	328	0.844	0.866	0.857	0.700	0.788	0.815
SVM	329	0.839	0.867	0.857	0.698	0.789	0.813
SVM	330	0.835	0.866	0.854	0.693	0.786	0.810
SVM	331	0.839	0.862	0.853	0.692	0.782	0.810
SVM	332	0.839	0.864	0.855	0.695	0.786	0.812
SVM	333	0.839	0.864	0.855	0.695	0.786	0.812
SVM	334	0.841	0.863	0.855	0.695	0.784	0.812
SVM	335	0.837	0.862	0.853	0.690	0.782	0.809
SVM	336	0.844	0.864	0.857	0.699	0.786	0.814
SVM	337	0.841	0.864	0.856	0.697	0.786	0.813
SVM	338	0.841	0.867	0.857	0.700	0.789	0.814
SVM	339	0.839	0.867	0.857	0.698	0.789	0.813
SVM	340	0.839	0.867	0.857	0.698	0.789	0.813
SVM	341	0.839	0.867	0.857	0.698	0.789	0.813
SVM	342	0.841	0.867	0.857	0.700	0.789	0.814
SVM	343	0.841	0.867	0.857	0.700	0.789	0.814
SVM	344	0.841	0.871	0.860	0.705	0.794	0.817
SVM	345	0.841	0.871	0.860	0.705	0.794	0.817
SVM	346	0.841	0.871	0.860	0.705	0.794	0.817
SVM	347	0.855	0.862	0.859	0.705	0.785	0.819
SVM	348	0.850	0.866	0.860	0.706	0.789	0.819
SVM	349	0.850	0.866	0.860	0.706	0.789	0.819
SVM	350	0.848	0.863	0.857	0.701	0.786	0.816
SVM	351	0.850	0.866	0.860	0.706	0.789	0.819
SVM	352	0.850	0.864	0.859	0.705	0.788	0.818

SVM	353	0.861	0.867	0.865	0.717	0.793	0.826
SVM	354	0.859	0.863	0.862	0.711	0.788	0.822
SVM	355	0.861	0.863	0.862	0.713	0.788	0.823
SVM	356	0.857	0.864	0.862	0.710	0.789	0.822
SVM	357	0.855	0.866	0.862	0.710	0.790	0.821
SVM	358	0.855	0.864	0.861	0.708	0.789	0.820
SVM	359	0.857	0.863	0.861	0.709	0.787	0.821
SVM	360	0.857	0.864	0.862	0.710	0.789	0.822
SVM	361	0.857	0.864	0.862	0.710	0.789	0.822
SVM	362	0.859	0.863	0.862	0.711	0.788	0.822
SVM	363	0.857	0.863	0.861	0.709	0.787	0.821
SVM	364	0.855	0.863	0.860	0.707	0.787	0.819
SVM	365	0.857	0.860	0.859	0.706	0.784	0.819
SVM	366	0.859	0.860	0.860	0.708	0.785	0.820
SVM	367	0.852	0.866	0.861	0.708	0.790	0.820
SVM	368	0.855	0.863	0.860	0.707	0.787	0.819
SVM	369	0.855	0.867	0.862	0.712	0.792	0.822
SVM	370	0.852	0.867	0.862	0.710	0.791	0.821

(3) IFS results on the LightGBM feature list

Classification algorithm	Number of features	SN	SP	ACC	MCC	Precision	F1-measure
DT	1	0.742	0.734	0.737	0.463	0.623	0.677
DT	2	0.731	0.752	0.744	0.472	0.636	0.680
DT	3	0.767	0.763	0.764	0.516	0.657	0.707
DT	4	0.762	0.776	0.771	0.526	0.668	0.712

DT	5	0.747	0.759	0.754	0.494	0.647	0.693
DT	6	0.758	0.752	0.754	0.497	0.644	0.696
DT	7	0.773	0.769	0.771	0.529	0.665	0.715
DT	8	0.773	0.789	0.783	0.550	0.684	0.726
DT	9	0.751	0.799	0.781	0.542	0.689	0.719
DT	10	0.780	0.825	0.808	0.597	0.725	0.752
DT	11	0.789	0.787	0.788	0.563	0.687	0.734
DT	12	0.789	0.795	0.793	0.571	0.695	0.739
DT	13	0.804	0.823	0.816	0.616	0.729	0.764
DT	14	0.795	0.794	0.794	0.576	0.696	0.742
DT	15	0.808	0.837	0.826	0.636	0.746	0.776
DT	16	0.778	0.828	0.809	0.598	0.728	0.752
DT	17	0.793	0.824	0.812	0.607	0.727	0.759
DT	18	0.806	0.825	0.818	0.621	0.732	0.767
DT	19	0.800	0.816	0.810	0.604	0.720	0.758
DT	20	0.828	0.833	0.831	0.649	0.746	0.785
DT	21	0.808	0.831	0.822	0.628	0.738	0.772
DT	22	0.815	0.833	0.826	0.637	0.743	0.777
DT	23	0.815	0.842	0.832	0.648	0.754	0.783
DT	24	0.804	0.836	0.824	0.631	0.743	0.772
DT	25	0.767	0.795	0.785	0.551	0.689	0.726
DT	26	0.802	0.836	0.823	0.629	0.743	0.771
DT	27	0.758	0.819	0.796	0.570	0.712	0.734
DT	28	0.767	0.781	0.776	0.536	0.674	0.718
DT	29	0.797	0.806	0.803	0.591	0.708	0.750

DT	30	0.833	0.842	0.839	0.663	0.758	0.793
DT	31	0.782	0.824	0.808	0.597	0.724	0.752
DT	32	0.826	0.858	0.846	0.676	0.775	0.800
DT	33	0.806	0.816	0.812	0.610	0.722	0.762
DT	34	0.789	0.827	0.812	0.606	0.729	0.758
DT	35	0.782	0.807	0.798	0.578	0.706	0.742
DT	36	0.769	0.820	0.801	0.581	0.717	0.742
DT	37	0.780	0.815	0.802	0.585	0.714	0.745
DT	38	0.808	0.814	0.812	0.609	0.720	0.761
DT	39	0.789	0.819	0.808	0.597	0.720	0.753
DT	40	0.775	0.831	0.810	0.599	0.730	0.752
DT	41	0.782	0.823	0.808	0.596	0.723	0.751
DT	42	0.813	0.836	0.827	0.638	0.745	0.778
DT	43	0.764	0.838	0.811	0.598	0.737	0.750
DT	44	0.767	0.807	0.792	0.564	0.702	0.733
DT	45	0.804	0.812	0.809	0.604	0.717	0.758
DT	46	0.791	0.824	0.812	0.605	0.727	0.757
DT	47	0.797	0.823	0.813	0.610	0.727	0.761
DT	48	0.780	0.828	0.810	0.600	0.728	0.753
DT	49	0.782	0.834	0.815	0.609	0.737	0.759
DT	50	0.764	0.840	0.812	0.600	0.738	0.751
DT	51	0.795	0.828	0.816	0.614	0.732	0.762
DT	52	0.797	0.808	0.804	0.594	0.711	0.752
DT	53	0.784	0.829	0.812	0.605	0.731	0.757
DT	54	0.819	0.814	0.816	0.619	0.722	0.768

DT	55	0.791	0.807	0.801	0.586	0.708	0.747
DT	56	0.793	0.794	0.794	0.574	0.695	0.741
DT	57	0.782	0.812	0.801	0.584	0.711	0.745
DT	58	0.760	0.807	0.790	0.558	0.700	0.729
DT	59	0.760	0.815	0.794	0.567	0.708	0.733
DT	60	0.802	0.811	0.808	0.601	0.715	0.756
DT	61	0.784	0.811	0.801	0.585	0.711	0.746
DT	62	0.795	0.817	0.809	0.602	0.721	0.756
DT	63	0.793	0.812	0.805	0.594	0.714	0.752
DT	64	0.773	0.815	0.799	0.579	0.712	0.741
DT	65	0.764	0.825	0.803	0.583	0.721	0.742
DT	66	0.758	0.806	0.788	0.555	0.698	0.727
DT	67	0.767	0.828	0.805	0.588	0.725	0.745
DT	68	0.778	0.808	0.797	0.576	0.706	0.740
DT	69	0.773	0.821	0.803	0.587	0.719	0.745
DT	70	0.786	0.834	0.817	0.613	0.738	0.761
DT	71	0.773	0.837	0.813	0.605	0.737	0.755
DT	72	0.771	0.832	0.809	0.597	0.731	0.750
DT	73	0.775	0.842	0.817	0.613	0.744	0.759
DT	74	0.767	0.812	0.795	0.570	0.707	0.736
DT	75	0.795	0.831	0.817	0.617	0.735	0.764
DT	76	0.780	0.810	0.799	0.579	0.708	0.742
DT	77	0.780	0.823	0.807	0.594	0.722	0.750
DT	78	0.778	0.803	0.794	0.570	0.700	0.737
DT	79	0.797	0.801	0.799	0.585	0.703	0.747

DT	80	0.747	0.845	0.808	0.590	0.740	0.743
DT	81	0.804	0.817	0.812	0.610	0.723	0.761
DT	82	0.791	0.832	0.817	0.614	0.736	0.762
DT	83	0.786	0.849	0.826	0.630	0.755	0.770
DT	84	0.773	0.825	0.806	0.591	0.724	0.748
DT	85	0.786	0.821	0.808	0.598	0.723	0.753
DT	86	0.780	0.823	0.807	0.594	0.722	0.750
DT	87	0.795	0.820	0.811	0.605	0.723	0.758
DT	88	0.804	0.829	0.820	0.623	0.736	0.768
DT	89	0.773	0.823	0.804	0.588	0.721	0.746
DT	90	0.778	0.820	0.804	0.589	0.719	0.747
DT	91	0.797	0.827	0.816	0.614	0.731	0.763
DT	92	0.762	0.823	0.800	0.578	0.718	0.739
DT	93	0.789	0.802	0.797	0.579	0.702	0.743
DT	94	0.769	0.841	0.814	0.605	0.741	0.755
DT	95	0.780	0.811	0.799	0.581	0.709	0.743
DT	96	0.775	0.807	0.795	0.572	0.704	0.738
DT	97	0.762	0.806	0.790	0.559	0.699	0.729
DT	98	0.815	0.821	0.819	0.624	0.730	0.770
DT	99	0.753	0.821	0.796	0.569	0.714	0.733
DT	100	0.800	0.824	0.815	0.613	0.729	0.763
DT	101	0.784	0.832	0.814	0.608	0.734	0.758
DT	102	0.780	0.827	0.809	0.598	0.727	0.752
DT	103	0.806	0.841	0.828	0.638	0.750	0.777
DT	104	0.782	0.810	0.799	0.581	0.709	0.743

DT	105	0.797	0.837	0.822	0.626	0.743	0.769
DT	106	0.767	0.825	0.803	0.585	0.722	0.744
DT	107	0.804	0.802	0.803	0.593	0.706	0.752
DT	108	0.773	0.844	0.817	0.612	0.745	0.759
DT	109	0.758	0.840	0.809	0.594	0.737	0.747
DT	110	0.811	0.823	0.818	0.622	0.730	0.768
DT	111	0.778	0.821	0.805	0.591	0.720	0.748
DT	112	0.800	0.819	0.812	0.607	0.723	0.759
DT	113	0.782	0.832	0.813	0.606	0.733	0.757
DT	114	0.784	0.827	0.811	0.602	0.728	0.755
DT	115	0.767	0.819	0.799	0.578	0.715	0.740
DT	116	0.771	0.812	0.797	0.574	0.709	0.738
DT	117	0.795	0.823	0.812	0.608	0.726	0.759
DT	118	0.800	0.820	0.812	0.609	0.725	0.760
DT	119	0.773	0.825	0.806	0.591	0.724	0.748
DT	120	0.753	0.817	0.794	0.564	0.710	0.731
DT	121	0.740	0.810	0.784	0.544	0.697	0.718
DT	122	0.769	0.811	0.795	0.571	0.706	0.736
DT	123	0.747	0.829	0.799	0.572	0.721	0.734
DT	124	0.751	0.836	0.804	0.584	0.730	0.740
DT	125	0.742	0.821	0.792	0.559	0.711	0.726
DT	126	0.749	0.799	0.781	0.540	0.688	0.717
DT	127	0.778	0.823	0.806	0.592	0.722	0.749
DT	128	0.775	0.821	0.804	0.589	0.720	0.747
DT	129	0.762	0.814	0.794	0.568	0.708	0.734

DT	130	0.767	0.820	0.800	0.579	0.716	0.740
DT	131	0.756	0.819	0.795	0.568	0.712	0.733
DT	132	0.749	0.820	0.794	0.563	0.711	0.730
DT	133	0.733	0.810	0.781	0.538	0.695	0.714
DT	134	0.786	0.828	0.812	0.606	0.730	0.757
DT	135	0.802	0.846	0.830	0.641	0.755	0.778
DT	136	0.756	0.828	0.801	0.578	0.722	0.738
DT	137	0.778	0.786	0.783	0.551	0.683	0.727
DT	138	0.784	0.837	0.817	0.614	0.740	0.761
DT	139	0.756	0.820	0.796	0.569	0.713	0.734
DT	140	0.780	0.803	0.794	0.572	0.701	0.738
DT	141	0.751	0.808	0.787	0.552	0.699	0.724
DT	142	0.800	0.817	0.811	0.606	0.722	0.759
DT	143	0.749	0.832	0.801	0.577	0.725	0.737
DT	144	0.802	0.802	0.802	0.591	0.705	0.751
DT	145	0.784	0.837	0.817	0.614	0.740	0.761
DT	146	0.756	0.824	0.799	0.574	0.718	0.736
DT	147	0.800	0.817	0.811	0.606	0.722	0.759
DT	148	0.731	0.815	0.784	0.542	0.700	0.716
DT	149	0.764	0.820	0.799	0.577	0.715	0.739
DT	150	0.782	0.797	0.791	0.567	0.695	0.736
DT	151	0.780	0.824	0.808	0.595	0.724	0.751
DT	152	0.769	0.819	0.800	0.580	0.715	0.741
DT	153	0.782	0.794	0.790	0.564	0.692	0.734
DT	154	0.795	0.812	0.806	0.596	0.715	0.753

DT	155	0.780	0.824	0.808	0.595	0.724	0.751
DT	156	0.773	0.824	0.805	0.590	0.722	0.747
DT	157	0.764	0.798	0.785	0.552	0.691	0.726
DT	158	0.769	0.847	0.818	0.613	0.749	0.759
DT	159	0.780	0.828	0.810	0.600	0.728	0.753
DT	160	0.762	0.840	0.811	0.598	0.738	0.750
DT	161	0.789	0.798	0.794	0.574	0.698	0.740
DT	162	0.793	0.816	0.808	0.598	0.719	0.754
DT	163	0.760	0.789	0.778	0.538	0.680	0.718
DT	164	0.769	0.807	0.793	0.566	0.702	0.734
DT	165	0.753	0.819	0.794	0.566	0.711	0.732
DT	166	0.830	0.829	0.830	0.647	0.742	0.784
DT	167	0.778	0.814	0.800	0.582	0.712	0.743
DT	168	0.824	0.812	0.817	0.622	0.722	0.770
DT	169	0.780	0.820	0.805	0.591	0.720	0.748
DT	170	0.780	0.801	0.793	0.569	0.698	0.737
DT	171	0.767	0.834	0.809	0.596	0.733	0.749
DT	172	0.786	0.825	0.811	0.603	0.727	0.756
DT	173	0.742	0.823	0.793	0.560	0.712	0.727
DT	174	0.758	0.823	0.799	0.574	0.717	0.737
DT	175	0.740	0.810	0.784	0.544	0.697	0.718
DT	176	0.791	0.832	0.817	0.614	0.736	0.762
DT	177	0.725	0.797	0.770	0.515	0.678	0.701
DT	178	0.771	0.802	0.790	0.563	0.697	0.732
DT	179	0.800	0.817	0.811	0.606	0.722	0.759

DT	180	0.773	0.819	0.802	0.584	0.716	0.744
DT	181	0.780	0.807	0.797	0.576	0.705	0.741
DT	182	0.793	0.815	0.807	0.597	0.717	0.753
DT	183	0.760	0.799	0.785	0.550	0.691	0.724
DT	184	0.767	0.807	0.792	0.564	0.702	0.733
DT	185	0.771	0.823	0.803	0.586	0.720	0.745
DT	186	0.793	0.789	0.790	0.568	0.690	0.738
DT	187	0.800	0.815	0.809	0.603	0.719	0.757
DT	188	0.762	0.807	0.790	0.560	0.700	0.730
DT	189	0.806	0.801	0.803	0.593	0.705	0.752
DT	190	0.782	0.831	0.812	0.605	0.732	0.756
DT	191	0.753	0.807	0.787	0.552	0.698	0.725
DT	192	0.782	0.806	0.797	0.577	0.704	0.741
DT	193	0.775	0.811	0.798	0.577	0.708	0.740
DT	194	0.767	0.807	0.792	0.564	0.702	0.733
DT	195	0.773	0.787	0.782	0.549	0.683	0.725
DT	196	0.762	0.821	0.799	0.577	0.716	0.739
DT	197	0.762	0.811	0.793	0.565	0.705	0.732
DT	198	0.744	0.819	0.791	0.558	0.709	0.726
DT	199	0.729	0.819	0.785	0.544	0.704	0.716
DT	200	0.780	0.795	0.790	0.563	0.693	0.734
DT	201	0.791	0.821	0.810	0.602	0.724	0.756
DT	202	0.775	0.811	0.798	0.577	0.708	0.740
DT	203	0.753	0.806	0.786	0.551	0.697	0.724
DT	204	0.778	0.823	0.806	0.592	0.722	0.749

DT	205	0.771	0.806	0.793	0.567	0.701	0.735
DT	206	0.778	0.807	0.796	0.574	0.705	0.739
DT	207	0.775	0.836	0.813	0.605	0.736	0.755
DT	208	0.762	0.799	0.785	0.552	0.692	0.725
DT	209	0.793	0.814	0.806	0.596	0.716	0.752
DT	210	0.804	0.787	0.794	0.577	0.691	0.743
DT	211	0.786	0.806	0.799	0.581	0.706	0.744
DT	212	0.760	0.790	0.779	0.540	0.682	0.719
DT	213	0.782	0.814	0.802	0.586	0.713	0.746
DT	214	0.742	0.823	0.793	0.560	0.712	0.727
DT	215	0.771	0.814	0.798	0.576	0.710	0.739
DT	216	0.782	0.812	0.801	0.584	0.711	0.745
DT	217	0.749	0.831	0.800	0.575	0.723	0.736
DT	218	0.749	0.795	0.778	0.535	0.684	0.715
DT	219	0.800	0.811	0.807	0.599	0.715	0.755
DT	220	0.769	0.829	0.807	0.592	0.727	0.747
DT	221	0.784	0.815	0.803	0.589	0.715	0.748
DT	222	0.738	0.850	0.808	0.589	0.744	0.741
DT	223	0.751	0.808	0.787	0.552	0.699	0.724
DT	224	0.775	0.789	0.784	0.552	0.685	0.727
DT	225	0.778	0.794	0.788	0.560	0.691	0.732
DT	226	0.769	0.825	0.804	0.587	0.723	0.745
DT	227	0.793	0.811	0.804	0.593	0.713	0.751
DT	228	0.753	0.810	0.789	0.555	0.701	0.726
DT	229	0.762	0.806	0.790	0.559	0.699	0.729

DT	230	0.744	0.808	0.785	0.546	0.697	0.720
DT	231	0.736	0.823	0.790	0.555	0.711	0.723
DT	232	0.760	0.814	0.794	0.566	0.707	0.732
DT	233	0.749	0.817	0.792	0.560	0.708	0.728
DT	234	0.756	0.785	0.774	0.530	0.675	0.713
DT	235	0.751	0.817	0.793	0.562	0.709	0.729
DT	236	0.756	0.799	0.783	0.546	0.690	0.721
DT	237	0.769	0.819	0.800	0.580	0.715	0.741
DT	238	0.751	0.831	0.801	0.577	0.724	0.737
DT	239	0.740	0.815	0.787	0.550	0.703	0.721
DT	240	0.767	0.799	0.787	0.556	0.693	0.728
DT	241	0.751	0.806	0.785	0.549	0.696	0.722
DT	242	0.780	0.795	0.790	0.563	0.693	0.734
DT	243	0.784	0.821	0.808	0.596	0.722	0.752
DT	244	0.753	0.824	0.798	0.572	0.717	0.735
DT	245	0.753	0.806	0.786	0.551	0.697	0.724
DT	246	0.767	0.820	0.800	0.579	0.716	0.740
DT	247	0.780	0.801	0.793	0.569	0.698	0.737
DT	248	0.778	0.803	0.794	0.570	0.700	0.737
DT	249	0.782	0.816	0.803	0.589	0.716	0.747
DT	250	0.795	0.798	0.797	0.580	0.700	0.744
DT	251	0.756	0.780	0.771	0.524	0.670	0.710
DT	252	0.769	0.781	0.776	0.538	0.675	0.719
DT	253	0.751	0.780	0.769	0.520	0.669	0.707
DT	254	0.747	0.790	0.774	0.528	0.678	0.711

DT	255	0.791	0.817	0.808	0.598	0.719	0.753
DT	256	0.786	0.806	0.799	0.581	0.706	0.744
DT	257	0.782	0.819	0.805	0.592	0.719	0.749
DT	258	0.760	0.802	0.786	0.553	0.694	0.726
DT	259	0.760	0.806	0.789	0.557	0.698	0.728
DT	260	0.718	0.799	0.769	0.512	0.679	0.698
DT	261	0.742	0.815	0.788	0.551	0.704	0.722
DT	262	0.762	0.806	0.790	0.559	0.699	0.729
DT	263	0.784	0.801	0.794	0.573	0.699	0.739
DT	264	0.780	0.801	0.793	0.569	0.698	0.737
DT	265	0.760	0.816	0.795	0.569	0.710	0.734
DT	266	0.767	0.802	0.789	0.559	0.696	0.730
DT	267	0.747	0.812	0.788	0.552	0.702	0.724
DT	268	0.780	0.817	0.803	0.588	0.717	0.747
DT	269	0.762	0.804	0.789	0.558	0.698	0.728
DT	270	0.793	0.794	0.794	0.574	0.695	0.741
DT	271	0.756	0.824	0.799	0.574	0.718	0.736
DT	272	0.789	0.794	0.792	0.570	0.694	0.738
DT	273	0.775	0.799	0.790	0.564	0.696	0.733
DT	274	0.760	0.820	0.798	0.573	0.714	0.736
DT	275	0.782	0.821	0.807	0.594	0.722	0.751
DT	276	0.753	0.814	0.791	0.560	0.705	0.728
DT	277	0.767	0.812	0.795	0.570	0.707	0.736
DT	278	0.782	0.812	0.801	0.584	0.711	0.745
DT	279	0.769	0.820	0.801	0.581	0.717	0.742

DT	280	0.789	0.815	0.805	0.593	0.716	0.751
DT	281	0.762	0.823	0.800	0.578	0.718	0.739
DT	282	0.731	0.831	0.794	0.560	0.719	0.725
DT	283	0.775	0.819	0.803	0.586	0.717	0.745
DT	284	0.773	0.797	0.788	0.559	0.692	0.730
DT	285	0.767	0.829	0.806	0.590	0.727	0.746
DT	286	0.764	0.815	0.796	0.571	0.710	0.736
DT	287	0.778	0.808	0.797	0.576	0.706	0.740
DT	288	0.778	0.806	0.795	0.573	0.703	0.738
DT	289	0.769	0.811	0.795	0.571	0.706	0.736
DT	290	0.747	0.806	0.784	0.545	0.695	0.720
DT	291	0.773	0.825	0.806	0.591	0.724	0.748
DT	292	0.764	0.793	0.782	0.546	0.686	0.723
DT	293	0.769	0.825	0.804	0.587	0.723	0.745
DT	294	0.762	0.803	0.788	0.556	0.696	0.728
DT	295	0.800	0.829	0.818	0.619	0.735	0.766
DT	296	0.742	0.823	0.793	0.560	0.712	0.727
DT	297	0.764	0.791	0.781	0.545	0.684	0.722
DT	298	0.760	0.819	0.797	0.572	0.713	0.736
DT	299	0.800	0.799	0.799	0.586	0.702	0.748
DT	300	0.771	0.790	0.783	0.550	0.685	0.725
DT	301	0.740	0.810	0.784	0.544	0.697	0.718
DT	302	0.740	0.824	0.793	0.560	0.713	0.726
DT	303	0.760	0.823	0.799	0.576	0.717	0.738
DT	304	0.806	0.808	0.808	0.602	0.713	0.757

DT	305	0.747	0.804	0.783	0.544	0.693	0.719
DT	306	0.749	0.819	0.793	0.562	0.710	0.729
DT	307	0.716	0.799	0.768	0.510	0.678	0.697
DT	308	0.762	0.816	0.796	0.571	0.710	0.735
DT	309	0.720	0.820	0.783	0.538	0.703	0.712
DT	310	0.773	0.819	0.802	0.584	0.716	0.744
DT	311	0.756	0.815	0.793	0.563	0.707	0.731
DT	312	0.729	0.810	0.780	0.534	0.694	0.711
DT	313	0.767	0.798	0.786	0.554	0.692	0.727
DT	314	0.736	0.795	0.773	0.523	0.680	0.707
DT	315	0.802	0.815	0.810	0.605	0.719	0.758
DT	316	0.736	0.808	0.781	0.538	0.694	0.714
DT	317	0.791	0.825	0.812	0.607	0.728	0.758
DT	318	0.731	0.797	0.772	0.521	0.680	0.705
DT	319	0.769	0.823	0.803	0.584	0.720	0.743
DT	320	0.758	0.801	0.785	0.549	0.692	0.723
DT	321	0.733	0.797	0.773	0.523	0.681	0.706
DT	322	0.760	0.811	0.792	0.563	0.704	0.731
DT	323	0.760	0.824	0.800	0.578	0.719	0.739
DT	324	0.771	0.814	0.798	0.576	0.710	0.739
DT	325	0.756	0.799	0.783	0.546	0.690	0.721
DT	326	0.718	0.804	0.772	0.518	0.685	0.701
DT	327	0.753	0.798	0.781	0.542	0.688	0.719
DT	328	0.756	0.828	0.801	0.578	0.722	0.738
DT	329	0.800	0.821	0.813	0.610	0.726	0.761

DT	330	0.789	0.802	0.797	0.579	0.702	0.743
DT	331	0.775	0.804	0.794	0.569	0.701	0.736
DT	332	0.773	0.807	0.794	0.570	0.703	0.737
DT	333	0.771	0.791	0.784	0.551	0.686	0.726
DT	334	0.742	0.833	0.799	0.573	0.725	0.733
DT	335	0.771	0.824	0.804	0.588	0.722	0.745
DT	336	0.756	0.814	0.792	0.562	0.706	0.730
DT	337	0.764	0.798	0.785	0.552	0.691	0.726
DT	338	0.778	0.807	0.796	0.574	0.705	0.739
DT	339	0.753	0.824	0.798	0.572	0.717	0.735
DT	340	0.747	0.807	0.785	0.547	0.696	0.721
DT	341	0.771	0.816	0.799	0.579	0.713	0.741
DT	342	0.740	0.802	0.779	0.535	0.689	0.713
DT	343	0.756	0.828	0.801	0.578	0.722	0.738
DT	344	0.800	0.803	0.802	0.590	0.706	0.750
DT	345	0.753	0.808	0.788	0.554	0.699	0.725
DT	346	0.784	0.798	0.793	0.570	0.697	0.738
DT	347	0.727	0.794	0.769	0.514	0.676	0.701
DT	348	0.778	0.806	0.795	0.573	0.703	0.738
DT	349	0.778	0.823	0.806	0.592	0.722	0.749
DT	350	0.762	0.819	0.798	0.574	0.713	0.737
DT	351	0.756	0.820	0.796	0.569	0.713	0.734
DT	352	0.742	0.803	0.781	0.538	0.691	0.715
DT	353	0.791	0.785	0.787	0.562	0.685	0.734
DT	354	0.742	0.824	0.794	0.562	0.714	0.728

DT	355	0.758	0.798	0.783	0.546	0.689	0.722
DT	356	0.751	0.819	0.794	0.564	0.710	0.730
DT	357	0.742	0.806	0.782	0.541	0.693	0.717
DT	358	0.775	0.815	0.800	0.581	0.713	0.743
DT	359	0.753	0.808	0.788	0.554	0.699	0.725
DT	360	0.742	0.776	0.763	0.508	0.662	0.700
DT	361	0.789	0.814	0.804	0.592	0.715	0.750
DT	362	0.773	0.811	0.797	0.575	0.708	0.739
DT	363	0.733	0.812	0.783	0.541	0.698	0.715
DT	364	0.749	0.814	0.790	0.556	0.704	0.726
DT	365	0.756	0.820	0.796	0.569	0.713	0.734
DT	366	0.760	0.774	0.769	0.523	0.666	0.710
DT	367	0.793	0.820	0.810	0.603	0.723	0.756
DT	368	0.767	0.802	0.789	0.559	0.696	0.730
DT	369	0.744	0.806	0.783	0.543	0.694	0.718
DT	370	0.744	0.825	0.795	0.565	0.716	0.730
KNN	1	0.729	0.731	0.731	0.448	0.616	0.668
KNN	2	0.769	0.761	0.764	0.517	0.656	0.708
KNN	3	0.830	0.778	0.798	0.592	0.689	0.753
KNN	4	0.797	0.768	0.779	0.550	0.670	0.728
KNN	5	0.861	0.781	0.811	0.623	0.699	0.772
KNN	6	0.857	0.799	0.821	0.638	0.716	0.780
KNN	7	0.868	0.782	0.814	0.630	0.702	0.776
KNN	8	0.859	0.802	0.823	0.643	0.720	0.783
KNN	9	0.883	0.802	0.832	0.665	0.725	0.796

KNN	10	0.857	0.803	0.823	0.642	0.720	0.783
KNN	11	0.874	0.785	0.818	0.639	0.706	0.781
KNN	12	0.877	0.811	0.835	0.669	0.733	0.798
KNN	13	0.879	0.836	0.852	0.698	0.760	0.815
KNN	14	0.859	0.829	0.840	0.673	0.749	0.800
KNN	15	0.877	0.840	0.853	0.700	0.764	0.816
KNN	16	0.872	0.841	0.853	0.697	0.764	0.815
KNN	17	0.863	0.845	0.852	0.694	0.767	0.812
KNN	18	0.892	0.853	0.867	0.728	0.782	0.833
KNN	19	0.879	0.849	0.860	0.712	0.775	0.824
KNN	20	0.863	0.849	0.854	0.698	0.772	0.815
KNN	21	0.883	0.846	0.860	0.713	0.773	0.824
KNN	22	0.859	0.831	0.841	0.674	0.750	0.801
KNN	23	0.855	0.844	0.848	0.685	0.764	0.807
KNN	24	0.888	0.846	0.862	0.717	0.774	0.827
KNN	25	0.866	0.838	0.848	0.689	0.760	0.809
KNN	26	0.863	0.842	0.850	0.691	0.764	0.811
KNN	27	0.885	0.821	0.845	0.688	0.746	0.810
KNN	28	0.899	0.819	0.848	0.697	0.746	0.815
KNN	29	0.899	0.815	0.846	0.693	0.742	0.813
KNN	30	0.892	0.814	0.843	0.686	0.739	0.808
KNN	31	0.892	0.820	0.847	0.693	0.746	0.812
KNN	32	0.890	0.804	0.836	0.674	0.729	0.802
KNN	33	0.859	0.814	0.830	0.655	0.732	0.790
KNN	34	0.892	0.807	0.839	0.679	0.732	0.804

KNN	35	0.813	0.777	0.790	0.574	0.683	0.742
KNN	36	0.800	0.768	0.780	0.552	0.671	0.730
KNN	37	0.804	0.776	0.786	0.564	0.680	0.737
KNN	38	0.819	0.784	0.797	0.587	0.691	0.750
KNN	39	0.804	0.765	0.780	0.553	0.670	0.731
KNN	40	0.808	0.778	0.790	0.571	0.683	0.741
KNN	41	0.822	0.772	0.790	0.577	0.681	0.745
KNN	42	0.808	0.772	0.785	0.564	0.677	0.737
KNN	43	0.833	0.772	0.794	0.587	0.684	0.751
KNN	44	0.822	0.771	0.790	0.575	0.679	0.744
KNN	45	0.828	0.777	0.796	0.588	0.687	0.751
KNN	46	0.804	0.769	0.782	0.558	0.673	0.733
KNN	47	0.826	0.763	0.786	0.571	0.673	0.742
KNN	48	0.817	0.781	0.794	0.582	0.688	0.747
KNN	49	0.835	0.781	0.801	0.598	0.693	0.757
KNN	50	0.808	0.771	0.785	0.563	0.676	0.736
KNN	51	0.828	0.765	0.789	0.576	0.676	0.745
KNN	52	0.817	0.761	0.782	0.562	0.670	0.736
KNN	53	0.819	0.769	0.788	0.572	0.678	0.742
KNN	54	0.824	0.759	0.783	0.565	0.669	0.738
KNN	55	0.819	0.764	0.785	0.566	0.673	0.739
KNN	56	0.826	0.765	0.788	0.574	0.676	0.743
KNN	57	0.835	0.771	0.794	0.588	0.683	0.751
KNN	58	0.819	0.773	0.790	0.576	0.681	0.744
KNN	59	0.826	0.767	0.789	0.575	0.677	0.744

KNN	60	0.802	0.757	0.774	0.543	0.662	0.725
KNN	61	0.822	0.773	0.791	0.578	0.682	0.745
KNN	62	0.813	0.771	0.786	0.567	0.677	0.739
KNN	63	0.782	0.772	0.776	0.540	0.670	0.722
KNN	64	0.813	0.765	0.783	0.562	0.672	0.736
KNN	65	0.797	0.767	0.778	0.549	0.669	0.728
KNN	66	0.786	0.759	0.769	0.530	0.659	0.717
KNN	67	0.782	0.768	0.773	0.536	0.666	0.719
KNN	68	0.826	0.772	0.792	0.581	0.682	0.747
KNN	69	0.804	0.757	0.775	0.545	0.662	0.726
KNN	70	0.800	0.763	0.776	0.547	0.666	0.727
KNN	71	0.817	0.763	0.783	0.563	0.671	0.737
KNN	72	0.808	0.769	0.784	0.562	0.675	0.735
KNN	73	0.808	0.765	0.781	0.558	0.671	0.733
KNN	74	0.793	0.771	0.779	0.549	0.672	0.727
KNN	75	0.806	0.776	0.787	0.566	0.680	0.738
KNN	76	0.793	0.764	0.775	0.542	0.665	0.724
KNN	77	0.817	0.765	0.785	0.566	0.673	0.738
KNN	78	0.775	0.767	0.770	0.528	0.663	0.715
KNN	79	0.811	0.759	0.778	0.553	0.665	0.731
KNN	80	0.793	0.767	0.776	0.545	0.668	0.725
KNN	81	0.793	0.765	0.776	0.543	0.667	0.724
KNN	82	0.797	0.771	0.781	0.553	0.673	0.730
KNN	83	0.802	0.761	0.776	0.547	0.665	0.727
KNN	84	0.782	0.737	0.753	0.503	0.637	0.702

KNN	85	0.773	0.741	0.753	0.499	0.638	0.699
KNN	86	0.797	0.752	0.769	0.534	0.656	0.720
KNN	87	0.771	0.751	0.758	0.508	0.647	0.704
KNN	88	0.775	0.744	0.756	0.505	0.642	0.703
KNN	89	0.771	0.734	0.748	0.490	0.632	0.694
KNN	90	0.784	0.747	0.761	0.516	0.647	0.709
KNN	91	0.806	0.738	0.763	0.527	0.646	0.717
KNN	92	0.769	0.744	0.753	0.499	0.640	0.699
KNN	93	0.789	0.733	0.753	0.505	0.636	0.704
KNN	94	0.784	0.725	0.747	0.493	0.628	0.697
KNN	95	0.797	0.739	0.761	0.520	0.644	0.713
KNN	96	0.811	0.739	0.766	0.533	0.648	0.720
KNN	97	0.789	0.743	0.760	0.516	0.645	0.710
KNN	98	0.808	0.725	0.756	0.516	0.635	0.711
KNN	99	0.795	0.747	0.765	0.526	0.650	0.716
KNN	100	0.815	0.725	0.758	0.522	0.637	0.715
KNN	101	0.800	0.735	0.759	0.518	0.641	0.712
KNN	102	0.764	0.717	0.735	0.467	0.615	0.682
KNN	103	0.780	0.737	0.753	0.501	0.637	0.701
KNN	104	0.789	0.720	0.745	0.492	0.625	0.697
KNN	105	0.789	0.720	0.745	0.492	0.625	0.697
KNN	106	0.793	0.729	0.753	0.506	0.634	0.705
KNN	107	0.786	0.733	0.753	0.503	0.635	0.703
KNN	108	0.800	0.728	0.754	0.510	0.635	0.708
KNN	109	0.786	0.726	0.749	0.497	0.630	0.699

KNN	110	0.782	0.726	0.747	0.493	0.628	0.697
KNN	111	0.780	0.730	0.749	0.494	0.631	0.698
KNN	112	0.811	0.722	0.755	0.516	0.633	0.711
KNN	113	0.800	0.722	0.751	0.505	0.630	0.705
KNN	114	0.819	0.728	0.762	0.529	0.640	0.719
KNN	115	0.778	0.729	0.747	0.491	0.629	0.696
KNN	116	0.802	0.737	0.761	0.522	0.643	0.714
KNN	117	0.800	0.717	0.748	0.500	0.626	0.702
KNN	118	0.797	0.724	0.751	0.504	0.631	0.704
KNN	119	0.811	0.737	0.764	0.530	0.646	0.719
KNN	120	0.778	0.738	0.753	0.500	0.637	0.700
KNN	121	0.791	0.728	0.751	0.502	0.632	0.703
KNN	122	0.795	0.743	0.762	0.522	0.647	0.713
KNN	123	0.806	0.730	0.758	0.519	0.639	0.713
KNN	124	0.800	0.728	0.754	0.510	0.635	0.708
KNN	125	0.780	0.735	0.752	0.500	0.636	0.700
KNN	126	0.775	0.737	0.751	0.497	0.635	0.698
KNN	127	0.800	0.729	0.755	0.512	0.636	0.708
KNN	128	0.811	0.733	0.762	0.526	0.642	0.717
KNN	129	0.797	0.741	0.762	0.522	0.645	0.713
KNN	130	0.789	0.746	0.762	0.519	0.647	0.711
KNN	131	0.795	0.746	0.764	0.525	0.649	0.715
KNN	132	0.808	0.731	0.760	0.523	0.640	0.715
KNN	133	0.806	0.734	0.761	0.523	0.642	0.715
KNN	134	0.780	0.712	0.737	0.476	0.616	0.688

KNN	135	0.811	0.721	0.754	0.514	0.632	0.710
KNN	136	0.782	0.739	0.755	0.506	0.640	0.704
KNN	137	0.797	0.722	0.750	0.503	0.630	0.704
KNN	138	0.800	0.737	0.760	0.520	0.642	0.712
KNN	139	0.806	0.735	0.762	0.525	0.643	0.716
KNN	140	0.795	0.729	0.753	0.508	0.634	0.706
KNN	141	0.791	0.724	0.749	0.498	0.629	0.700
KNN	142	0.804	0.737	0.762	0.524	0.644	0.715
KNN	143	0.808	0.729	0.758	0.520	0.638	0.713
KNN	144	0.786	0.714	0.741	0.485	0.620	0.693
KNN	145	0.800	0.699	0.736	0.482	0.611	0.693
KNN	146	0.817	0.707	0.748	0.506	0.622	0.707
KNN	147	0.795	0.722	0.749	0.501	0.629	0.702
KNN	148	0.819	0.713	0.753	0.515	0.628	0.711
KNN	149	0.817	0.712	0.751	0.512	0.627	0.709
KNN	150	0.806	0.709	0.745	0.499	0.621	0.702
KNN	151	0.817	0.709	0.749	0.509	0.625	0.708
KNN	152	0.780	0.707	0.734	0.471	0.611	0.685
KNN	153	0.793	0.717	0.745	0.494	0.624	0.698
KNN	154	0.795	0.721	0.749	0.500	0.628	0.702
KNN	155	0.775	0.729	0.746	0.489	0.629	0.694
KNN	156	0.793	0.722	0.749	0.499	0.628	0.701
KNN	157	0.771	0.712	0.734	0.468	0.613	0.683
KNN	158	0.804	0.705	0.742	0.493	0.618	0.699
KNN	159	0.811	0.703	0.743	0.496	0.617	0.701

KNN	160	0.784	0.724	0.746	0.492	0.627	0.697
KNN	161	0.815	0.708	0.748	0.506	0.623	0.706
KNN	162	0.822	0.722	0.759	0.526	0.637	0.717
KNN	163	0.797	0.714	0.745	0.495	0.623	0.700
KNN	164	0.811	0.731	0.761	0.525	0.641	0.716
KNN	165	0.808	0.708	0.745	0.499	0.621	0.702
KNN	166	0.804	0.718	0.750	0.505	0.628	0.705
KNN	167	0.797	0.733	0.757	0.514	0.638	0.709
KNN	168	0.784	0.716	0.741	0.484	0.620	0.693
KNN	169	0.808	0.738	0.764	0.529	0.646	0.718
KNN	170	0.808	0.731	0.760	0.523	0.640	0.715
KNN	171	0.786	0.720	0.744	0.490	0.624	0.696
KNN	172	0.811	0.747	0.771	0.541	0.655	0.724
KNN	173	0.808	0.739	0.765	0.531	0.647	0.719
KNN	174	0.793	0.739	0.759	0.516	0.643	0.710
KNN	175	0.808	0.734	0.762	0.525	0.643	0.716
KNN	176	0.784	0.729	0.749	0.497	0.631	0.699
KNN	177	0.797	0.729	0.754	0.510	0.635	0.707
KNN	178	0.817	0.726	0.760	0.526	0.639	0.717
KNN	179	0.822	0.734	0.767	0.538	0.646	0.724
KNN	180	0.839	0.729	0.770	0.549	0.647	0.731
KNN	181	0.819	0.734	0.766	0.536	0.646	0.722
KNN	182	0.811	0.730	0.760	0.524	0.640	0.715
KNN	183	0.784	0.748	0.762	0.517	0.648	0.710
KNN	184	0.813	0.737	0.765	0.532	0.646	0.720

KNN	185	0.804	0.724	0.753	0.511	0.633	0.708
KNN	186	0.811	0.733	0.762	0.526	0.642	0.717
KNN	187	0.815	0.734	0.764	0.532	0.645	0.720
KNN	188	0.773	0.744	0.755	0.503	0.642	0.701
KNN	189	0.795	0.724	0.750	0.502	0.630	0.703
KNN	190	0.800	0.738	0.761	0.521	0.644	0.713
KNN	191	0.804	0.734	0.760	0.521	0.641	0.714
KNN	192	0.786	0.741	0.758	0.511	0.642	0.707
KNN	193	0.806	0.720	0.752	0.509	0.630	0.707
KNN	194	0.808	0.718	0.752	0.510	0.630	0.708
KNN	195	0.811	0.747	0.771	0.541	0.655	0.724
KNN	196	0.789	0.742	0.759	0.515	0.644	0.709
KNN	197	0.813	0.721	0.755	0.516	0.633	0.712
KNN	198	0.808	0.734	0.762	0.525	0.643	0.716
KNN	199	0.804	0.741	0.764	0.528	0.647	0.717
KNN	200	0.800	0.754	0.771	0.537	0.658	0.722
KNN	201	0.806	0.722	0.753	0.511	0.632	0.709
KNN	202	0.789	0.734	0.754	0.507	0.637	0.705
KNN	203	0.813	0.743	0.769	0.539	0.652	0.724
KNN	204	0.802	0.738	0.762	0.523	0.644	0.714
KNN	205	0.800	0.720	0.749	0.503	0.628	0.703
KNN	206	0.802	0.729	0.756	0.514	0.636	0.710
KNN	207	0.811	0.714	0.750	0.508	0.627	0.707
KNN	208	0.815	0.716	0.753	0.513	0.629	0.710
KNN	209	0.800	0.717	0.748	0.500	0.626	0.702

KNN	210	0.804	0.726	0.755	0.513	0.635	0.709
KNN	211	0.815	0.728	0.760	0.525	0.639	0.716
KNN	212	0.797	0.714	0.745	0.495	0.623	0.700
KNN	213	0.813	0.721	0.755	0.516	0.633	0.712
KNN	214	0.868	0.721	0.776	0.569	0.648	0.742
KNN	215	0.795	0.730	0.754	0.509	0.636	0.706
KNN	216	0.786	0.717	0.743	0.487	0.622	0.695
KNN	217	0.789	0.722	0.747	0.495	0.627	0.699
KNN	218	0.767	0.705	0.728	0.457	0.606	0.677
KNN	219	0.753	0.707	0.724	0.446	0.603	0.670
KNN	220	0.767	0.700	0.725	0.452	0.602	0.674
KNN	221	0.764	0.687	0.716	0.437	0.591	0.667
KNN	222	0.784	0.713	0.740	0.481	0.618	0.691
KNN	223	0.760	0.707	0.726	0.452	0.605	0.674
KNN	224	0.760	0.708	0.727	0.453	0.606	0.674
KNN	225	0.775	0.709	0.734	0.469	0.612	0.684
KNN	226	0.760	0.709	0.728	0.455	0.607	0.675
KNN	227	0.780	0.713	0.738	0.477	0.617	0.689
KNN	228	0.775	0.709	0.734	0.469	0.612	0.684
KNN	229	0.771	0.711	0.733	0.466	0.612	0.682
KNN	230	0.775	0.716	0.738	0.476	0.618	0.688
KNN	231	0.769	0.709	0.731	0.463	0.610	0.680
KNN	232	0.731	0.713	0.720	0.432	0.601	0.660
KNN	233	0.778	0.694	0.725	0.456	0.600	0.678
KNN	234	0.782	0.687	0.722	0.454	0.597	0.677

KNN	235	0.767	0.703	0.726	0.454	0.604	0.676
KNN	236	0.778	0.698	0.727	0.460	0.603	0.680
KNN	237	0.751	0.698	0.717	0.434	0.595	0.664
KNN	238	0.773	0.707	0.731	0.464	0.609	0.682
KNN	239	0.795	0.701	0.736	0.480	0.612	0.692
KNN	240	0.758	0.711	0.728	0.454	0.608	0.675
KNN	241	0.771	0.699	0.726	0.455	0.602	0.676
KNN	242	0.758	0.695	0.718	0.438	0.595	0.667
KNN	243	0.778	0.700	0.729	0.462	0.605	0.681
KNN	244	0.784	0.696	0.729	0.465	0.604	0.683
KNN	245	0.749	0.699	0.717	0.434	0.595	0.663
KNN	246	0.802	0.700	0.738	0.485	0.613	0.695
KNN	247	0.778	0.691	0.723	0.453	0.598	0.676
KNN	248	0.791	0.707	0.738	0.481	0.615	0.692
KNN	249	0.786	0.688	0.725	0.459	0.599	0.680
KNN	250	0.791	0.699	0.733	0.473	0.608	0.688
KNN	251	0.764	0.704	0.726	0.454	0.605	0.675
KNN	252	0.771	0.691	0.721	0.447	0.596	0.672
KNN	253	0.747	0.700	0.717	0.433	0.596	0.663
KNN	254	0.762	0.694	0.719	0.441	0.596	0.669
KNN	255	0.797	0.679	0.723	0.461	0.595	0.682
KNN	256	0.749	0.705	0.722	0.440	0.601	0.667
KNN	257	0.764	0.705	0.727	0.455	0.606	0.676
KNN	258	0.793	0.698	0.733	0.474	0.608	0.688
KNN	259	0.762	0.699	0.722	0.446	0.600	0.671

KNN	260	0.767	0.701	0.726	0.453	0.603	0.675
KNN	261	0.760	0.700	0.722	0.445	0.600	0.671
KNN	262	0.797	0.692	0.731	0.473	0.605	0.688
KNN	263	0.795	0.695	0.732	0.474	0.607	0.688
KNN	264	0.758	0.701	0.722	0.445	0.600	0.670
KNN	265	0.778	0.694	0.725	0.456	0.600	0.678
KNN	266	0.775	0.708	0.733	0.468	0.611	0.683
KNN	267	0.778	0.705	0.732	0.467	0.610	0.683
KNN	268	0.778	0.683	0.718	0.445	0.592	0.672
KNN	269	0.758	0.698	0.720	0.441	0.597	0.668
KNN	270	0.742	0.707	0.720	0.435	0.600	0.663
KNN	271	0.793	0.707	0.739	0.483	0.615	0.693
KNN	272	0.797	0.688	0.729	0.470	0.602	0.686
KNN	273	0.778	0.698	0.727	0.460	0.603	0.680
KNN	274	0.771	0.713	0.735	0.469	0.614	0.684
KNN	275	0.793	0.695	0.731	0.472	0.606	0.687
KNN	276	0.791	0.711	0.740	0.485	0.618	0.694
KNN	277	0.762	0.712	0.731	0.459	0.610	0.678
KNN	278	0.762	0.709	0.729	0.457	0.608	0.676
KNN	279	0.760	0.712	0.730	0.457	0.610	0.676
KNN	280	0.778	0.703	0.731	0.465	0.608	0.682
KNN	281	0.780	0.708	0.735	0.472	0.612	0.686
KNN	282	0.780	0.701	0.731	0.466	0.607	0.683
KNN	283	0.751	0.690	0.713	0.427	0.589	0.660
KNN	284	0.756	0.700	0.721	0.441	0.599	0.668

KNN	285	0.758	0.694	0.717	0.437	0.594	0.666
KNN	286	0.769	0.698	0.724	0.451	0.601	0.674
KNN	287	0.786	0.694	0.728	0.464	0.603	0.683
KNN	288	0.773	0.700	0.727	0.458	0.604	0.678
KNN	289	0.782	0.686	0.722	0.452	0.596	0.676
KNN	290	0.784	0.687	0.723	0.456	0.597	0.678
KNN	291	0.767	0.675	0.709	0.427	0.583	0.662
KNN	292	0.747	0.674	0.701	0.407	0.576	0.650
KNN	293	0.786	0.670	0.713	0.441	0.585	0.671
KNN	294	0.760	0.679	0.709	0.425	0.584	0.660
KNN	295	0.760	0.684	0.713	0.430	0.588	0.663
KNN	296	0.780	0.673	0.713	0.437	0.585	0.669
KNN	297	0.747	0.671	0.699	0.404	0.574	0.649
KNN	298	0.784	0.670	0.713	0.439	0.585	0.670
KNN	299	0.744	0.677	0.702	0.407	0.577	0.650
KNN	300	0.778	0.674	0.713	0.437	0.585	0.668
KNN	301	0.780	0.679	0.717	0.444	0.590	0.672
KNN	302	0.756	0.678	0.707	0.419	0.581	0.657
KNN	303	0.791	0.682	0.722	0.457	0.595	0.679
KNN	304	0.756	0.673	0.704	0.414	0.577	0.655
KNN	305	0.793	0.674	0.718	0.451	0.590	0.677
KNN	306	0.749	0.670	0.699	0.405	0.573	0.649
KNN	307	0.767	0.670	0.706	0.422	0.579	0.660
KNN	308	0.778	0.673	0.712	0.435	0.584	0.667
KNN	309	0.778	0.687	0.721	0.449	0.595	0.674

KNN	310	0.797	0.682	0.725	0.463	0.597	0.683
KNN	311	0.791	0.666	0.713	0.442	0.584	0.672
KNN	312	0.784	0.657	0.704	0.427	0.575	0.664
KNN	313	0.778	0.665	0.707	0.428	0.579	0.664
KNN	314	0.780	0.669	0.710	0.434	0.582	0.667
KNN	315	0.800	0.661	0.713	0.445	0.583	0.674
KNN	316	0.773	0.662	0.704	0.421	0.575	0.660
KNN	317	0.758	0.668	0.701	0.411	0.574	0.653
KNN	318	0.767	0.656	0.697	0.408	0.569	0.653
KNN	319	0.771	0.662	0.703	0.419	0.575	0.659
KNN	320	0.775	0.658	0.702	0.419	0.573	0.659
KNN	321	0.775	0.660	0.703	0.421	0.574	0.660
KNN	322	0.758	0.664	0.699	0.407	0.571	0.652
KNN	323	0.797	0.669	0.717	0.451	0.588	0.677
KNN	324	0.762	0.652	0.693	0.400	0.564	0.649
KNN	325	0.758	0.654	0.693	0.398	0.565	0.647
KNN	326	0.789	0.653	0.704	0.427	0.574	0.664
KNN	327	0.771	0.657	0.699	0.414	0.571	0.656
KNN	328	0.778	0.661	0.704	0.424	0.576	0.662
KNN	329	0.780	0.640	0.692	0.406	0.562	0.653
KNN	330	0.795	0.641	0.699	0.422	0.568	0.662
KNN	331	0.775	0.652	0.698	0.413	0.569	0.656
KNN	332	0.780	0.656	0.702	0.421	0.573	0.660
KNN	333	0.744	0.660	0.691	0.391	0.564	0.642
KNN	334	0.782	0.648	0.698	0.416	0.568	0.658

KNN	335	0.758	0.654	0.693	0.398	0.565	0.647
KNN	336	0.771	0.652	0.696	0.409	0.567	0.654
KNN	337	0.778	0.649	0.697	0.413	0.568	0.656
KNN	338	0.782	0.640	0.693	0.408	0.563	0.654
KNN	339	0.751	0.653	0.690	0.391	0.562	0.643
KNN	340	0.769	0.643	0.690	0.398	0.560	0.648
KNN	341	0.771	0.652	0.696	0.409	0.567	0.654
KNN	342	0.762	0.645	0.689	0.394	0.560	0.646
KNN	343	0.784	0.677	0.717	0.445	0.589	0.673
KNN	344	0.767	0.669	0.705	0.421	0.578	0.659
KNN	345	0.800	0.678	0.723	0.462	0.595	0.682
KNN	346	0.793	0.683	0.724	0.460	0.597	0.681
KNN	347	0.793	0.674	0.718	0.451	0.590	0.677
KNN	348	0.791	0.681	0.722	0.456	0.594	0.679
KNN	349	0.782	0.691	0.725	0.457	0.600	0.679
KNN	350	0.811	0.681	0.729	0.475	0.600	0.690
KNN	351	0.795	0.686	0.726	0.465	0.600	0.684
KNN	352	0.804	0.687	0.731	0.475	0.603	0.689
KNN	353	0.749	0.705	0.722	0.440	0.601	0.667
KNN	354	0.797	0.684	0.726	0.466	0.599	0.684
KNN	355	0.793	0.698	0.733	0.474	0.608	0.688
KNN	356	0.784	0.692	0.726	0.461	0.601	0.681
KNN	357	0.747	0.698	0.716	0.430	0.594	0.661
KNN	358	0.800	0.692	0.732	0.476	0.606	0.689
KNN	359	0.780	0.692	0.725	0.457	0.600	0.678

KNN	360	0.782	0.688	0.723	0.455	0.598	0.677
KNN	361	0.784	0.696	0.729	0.465	0.604	0.683
KNN	362	0.804	0.691	0.733	0.478	0.606	0.691
KNN	363	0.775	0.694	0.724	0.454	0.600	0.676
KNN	364	0.802	0.686	0.729	0.471	0.602	0.687
KNN	365	0.793	0.688	0.727	0.465	0.601	0.684
KNN	366	0.780	0.698	0.728	0.462	0.604	0.681
KNN	367	0.817	0.677	0.729	0.477	0.599	0.692
KNN	368	0.782	0.687	0.722	0.454	0.597	0.677
KNN	369	0.771	0.699	0.726	0.455	0.602	0.676
KNN	370	0.769	0.691	0.720	0.445	0.596	0.671
RF	1	0.729	0.743	0.738	0.461	0.627	0.674
RF	2	0.795	0.778	0.785	0.559	0.680	0.733
RF	3	0.800	0.814	0.808	0.602	0.717	0.756
RF	4	0.813	0.824	0.820	0.625	0.732	0.770
RF	5	0.813	0.827	0.821	0.628	0.735	0.772
RF	6	0.815	0.845	0.834	0.651	0.757	0.785
RF	7	0.830	0.879	0.861	0.704	0.802	0.816
RF	8	0.822	0.877	0.857	0.695	0.799	0.810
RF	9	0.833	0.876	0.860	0.703	0.799	0.816
RF	10	0.839	0.881	0.866	0.715	0.807	0.823
RF	11	0.852	0.883	0.871	0.728	0.811	0.831
RF	12	0.855	0.884	0.873	0.732	0.813	0.834
RF	13	0.850	0.898	0.880	0.745	0.832	0.841
RF	14	0.861	0.897	0.884	0.753	0.832	0.846

RF	15	0.872	0.904	0.892	0.771	0.843	0.857
RF	16	0.890	0.907	0.901	0.790	0.851	0.870
RF	17	0.892	0.904	0.899	0.787	0.846	0.868
RF	18	0.881	0.906	0.897	0.781	0.847	0.864
RF	19	0.885	0.904	0.897	0.782	0.845	0.865
RF	20	0.881	0.907	0.898	0.783	0.849	0.865
RF	21	0.890	0.905	0.899	0.787	0.847	0.868
RF	22	0.903	0.915	0.911	0.811	0.863	0.883
RF	23	0.874	0.914	0.899	0.785	0.857	0.866
RF	24	0.892	0.915	0.907	0.802	0.862	0.877
RF	25	0.899	0.909	0.905	0.799	0.854	0.876
RF	26	0.896	0.914	0.907	0.804	0.860	0.878
RF	27	0.879	0.910	0.898	0.784	0.853	0.866
RF	28	0.877	0.906	0.895	0.777	0.847	0.861
RF	29	0.879	0.906	0.896	0.779	0.847	0.863
RF	30	0.877	0.906	0.895	0.777	0.847	0.861
RF	31	0.874	0.913	0.898	0.784	0.856	0.865
RF	32	0.877	0.910	0.898	0.782	0.852	0.864
RF	33	0.870	0.919	0.901	0.788	0.864	0.867
RF	34	0.877	0.909	0.897	0.781	0.850	0.863
RF	35	0.881	0.909	0.898	0.784	0.851	0.866
RF	36	0.881	0.914	0.902	0.791	0.858	0.870
RF	37	0.879	0.913	0.900	0.787	0.856	0.867
RF	38	0.879	0.914	0.901	0.789	0.858	0.868
RF	39	0.883	0.918	0.905	0.798	0.864	0.874

RF	40	0.883	0.917	0.904	0.796	0.862	0.873
RF	41	0.881	0.911	0.900	0.788	0.855	0.868
RF	42	0.885	0.905	0.898	0.783	0.846	0.865
RF	43	0.885	0.909	0.900	0.788	0.852	0.868
RF	44	0.881	0.917	0.903	0.794	0.862	0.871
RF	45	0.883	0.907	0.898	0.785	0.850	0.866
RF	46	0.892	0.898	0.896	0.781	0.839	0.864
RF	47	0.874	0.913	0.898	0.784	0.856	0.865
RF	48	0.883	0.911	0.901	0.790	0.855	0.869
RF	49	0.888	0.910	0.902	0.792	0.854	0.870
RF	50	0.888	0.907	0.900	0.788	0.850	0.869
RF	51	0.888	0.910	0.902	0.792	0.854	0.870
RF	52	0.883	0.913	0.902	0.791	0.857	0.870
RF	53	0.896	0.901	0.899	0.788	0.843	0.869
RF	54	0.877	0.902	0.893	0.773	0.841	0.859
RF	55	0.888	0.909	0.901	0.790	0.852	0.869
RF	56	0.894	0.906	0.902	0.793	0.849	0.871
RF	57	0.883	0.911	0.901	0.790	0.855	0.869
RF	58	0.879	0.909	0.898	0.783	0.851	0.865
RF	59	0.894	0.905	0.901	0.791	0.848	0.870
RF	60	0.894	0.910	0.904	0.797	0.855	0.874
RF	61	0.888	0.917	0.906	0.800	0.863	0.875
RF	62	0.892	0.909	0.903	0.794	0.853	0.872
RF	63	0.868	0.909	0.894	0.773	0.849	0.858
RF	64	0.885	0.907	0.899	0.787	0.850	0.867

RF	65	0.877	0.915	0.901	0.789	0.860	0.868
RF	66	0.883	0.914	0.903	0.793	0.859	0.871
RF	67	0.885	0.913	0.903	0.793	0.857	0.871
RF	68	0.885	0.913	0.903	0.793	0.857	0.871
RF	69	0.894	0.913	0.906	0.801	0.858	0.876
RF	70	0.879	0.914	0.901	0.789	0.858	0.868
RF	71	0.890	0.914	0.905	0.798	0.860	0.874
RF	72	0.881	0.909	0.898	0.784	0.851	0.866
RF	73	0.874	0.907	0.895	0.777	0.848	0.861
RF	74	0.879	0.902	0.894	0.775	0.842	0.860
RF	75	0.892	0.914	0.906	0.800	0.860	0.876
RF	76	0.881	0.906	0.897	0.781	0.847	0.864
RF	77	0.885	0.906	0.898	0.785	0.848	0.866
RF	78	0.879	0.905	0.895	0.778	0.845	0.862
RF	79	0.877	0.909	0.897	0.781	0.850	0.863
RF	80	0.881	0.905	0.896	0.780	0.846	0.863
RF	81	0.892	0.904	0.899	0.787	0.846	0.868
RF	82	0.883	0.909	0.899	0.786	0.851	0.867
RF	83	0.894	0.900	0.898	0.785	0.841	0.867
RF	84	0.877	0.898	0.890	0.768	0.836	0.856
RF	85	0.888	0.917	0.906	0.800	0.863	0.875
RF	86	0.883	0.909	0.899	0.786	0.851	0.867
RF	87	0.881	0.909	0.898	0.784	0.851	0.866
RF	88	0.885	0.909	0.900	0.788	0.852	0.868
RF	89	0.892	0.909	0.903	0.794	0.853	0.872

RF	90	0.892	0.907	0.902	0.792	0.851	0.871
RF	91	0.903	0.911	0.908	0.806	0.858	0.880
RF	92	0.888	0.900	0.895	0.779	0.840	0.863
RF	93	0.881	0.904	0.895	0.778	0.844	0.862
RF	94	0.877	0.910	0.898	0.782	0.852	0.864
RF	95	0.879	0.897	0.890	0.768	0.835	0.856
RF	96	0.879	0.910	0.898	0.784	0.853	0.866
RF	97	0.874	0.910	0.897	0.780	0.852	0.863
RF	98	0.877	0.911	0.898	0.784	0.854	0.865
RF	99	0.890	0.913	0.904	0.797	0.858	0.874
RF	100	0.899	0.900	0.899	0.788	0.841	0.869
RF	101	0.870	0.902	0.890	0.767	0.840	0.855
RF	102	0.868	0.893	0.884	0.754	0.828	0.847
RF	103	0.881	0.909	0.898	0.784	0.851	0.866
RF	104	0.883	0.897	0.892	0.772	0.835	0.859
RF	105	0.868	0.909	0.894	0.773	0.849	0.858
RF	106	0.877	0.902	0.893	0.773	0.841	0.859
RF	107	0.866	0.900	0.887	0.760	0.836	0.851
RF	108	0.868	0.907	0.893	0.772	0.847	0.857
RF	109	0.870	0.901	0.889	0.765	0.839	0.854
RF	110	0.868	0.909	0.894	0.773	0.849	0.858
RF	111	0.866	0.902	0.889	0.763	0.840	0.852
RF	112	0.870	0.909	0.894	0.775	0.849	0.860
RF	113	0.877	0.906	0.895	0.777	0.847	0.861
RF	114	0.868	0.901	0.889	0.764	0.838	0.853

RF	115	0.846	0.905	0.883	0.750	0.840	0.843
RF	116	0.863	0.902	0.888	0.761	0.839	0.851
RF	117	0.879	0.911	0.899	0.786	0.854	0.866
RF	118	0.866	0.905	0.890	0.766	0.843	0.854
RF	119	0.874	0.900	0.890	0.768	0.838	0.856
RF	120	0.879	0.902	0.894	0.775	0.842	0.860
RF	121	0.885	0.906	0.898	0.785	0.848	0.866
RF	122	0.874	0.897	0.889	0.764	0.834	0.854
RF	123	0.874	0.901	0.891	0.769	0.839	0.857
RF	124	0.872	0.904	0.892	0.771	0.843	0.857
RF	125	0.861	0.897	0.884	0.753	0.832	0.846
RF	126	0.885	0.896	0.892	0.772	0.834	0.859
RF	127	0.872	0.905	0.893	0.772	0.844	0.858
RF	128	0.881	0.894	0.889	0.767	0.832	0.856
RF	129	0.877	0.893	0.887	0.762	0.829	0.852
RF	130	0.852	0.900	0.882	0.749	0.834	0.843
RF	131	0.874	0.897	0.889	0.764	0.834	0.854
RF	132	0.859	0.904	0.887	0.759	0.841	0.850
RF	133	0.874	0.892	0.885	0.758	0.827	0.850
RF	134	0.874	0.894	0.887	0.761	0.831	0.852
RF	135	0.885	0.894	0.891	0.771	0.832	0.858
RF	136	0.877	0.896	0.889	0.765	0.833	0.854
RF	137	0.872	0.902	0.891	0.769	0.841	0.856
RF	138	0.874	0.894	0.887	0.761	0.831	0.852
RF	139	0.868	0.894	0.885	0.756	0.829	0.848

RF	140	0.883	0.904	0.896	0.780	0.844	0.863
RF	141	0.883	0.905	0.897	0.782	0.846	0.864
RF	142	0.870	0.892	0.884	0.754	0.826	0.848
RF	143	0.863	0.900	0.886	0.758	0.836	0.849
RF	144	0.861	0.890	0.880	0.745	0.823	0.842
RF	145	0.857	0.897	0.882	0.749	0.831	0.844
RF	146	0.863	0.896	0.884	0.753	0.831	0.847
RF	147	0.877	0.893	0.887	0.762	0.829	0.852
RF	148	0.872	0.897	0.888	0.763	0.834	0.853
RF	149	0.861	0.890	0.880	0.745	0.823	0.842
RF	150	0.861	0.901	0.886	0.758	0.837	0.849
RF	151	0.883	0.893	0.889	0.767	0.830	0.856
RF	152	0.859	0.892	0.880	0.745	0.825	0.841
RF	153	0.872	0.887	0.881	0.750	0.820	0.845
RF	154	0.877	0.897	0.889	0.766	0.834	0.855
RF	155	0.866	0.893	0.883	0.752	0.827	0.846
RF	156	0.866	0.898	0.886	0.758	0.834	0.850
RF	157	0.863	0.890	0.880	0.747	0.824	0.843
RF	158	0.855	0.897	0.881	0.747	0.831	0.843
RF	159	0.866	0.894	0.884	0.754	0.829	0.847
RF	160	0.859	0.897	0.883	0.751	0.832	0.845
RF	161	0.857	0.889	0.877	0.740	0.821	0.838
RF	162	0.874	0.893	0.886	0.760	0.829	0.851
RF	163	0.870	0.887	0.880	0.748	0.820	0.844
RF	164	0.874	0.881	0.879	0.746	0.814	0.843

RF	165	0.881	0.901	0.894	0.775	0.840	0.860
RF	166	0.870	0.893	0.885	0.756	0.828	0.849
RF	167	0.861	0.885	0.876	0.739	0.816	0.838
RF	168	0.877	0.885	0.882	0.752	0.819	0.847
RF	169	0.863	0.892	0.881	0.749	0.825	0.844
RF	170	0.877	0.896	0.889	0.765	0.833	0.854
RF	171	0.852	0.894	0.879	0.742	0.827	0.839
RF	172	0.863	0.901	0.887	0.760	0.838	0.850
RF	173	0.866	0.892	0.882	0.751	0.826	0.845
RF	174	0.861	0.888	0.878	0.742	0.820	0.840
RF	175	0.861	0.890	0.880	0.745	0.823	0.842
RF	176	0.877	0.894	0.888	0.763	0.831	0.853
RF	177	0.850	0.898	0.880	0.745	0.832	0.841
RF	178	0.885	0.890	0.889	0.766	0.827	0.855
RF	179	0.863	0.890	0.880	0.747	0.824	0.843
RF	180	0.852	0.893	0.878	0.741	0.825	0.839
RF	181	0.852	0.884	0.872	0.730	0.813	0.832
RF	182	0.872	0.881	0.878	0.744	0.813	0.842
RF	183	0.861	0.898	0.885	0.755	0.834	0.847
RF	184	0.863	0.896	0.884	0.753	0.831	0.847
RF	185	0.868	0.887	0.880	0.746	0.819	0.843
RF	186	0.868	0.888	0.880	0.748	0.821	0.844
RF	187	0.872	0.888	0.882	0.752	0.822	0.846
RF	188	0.857	0.880	0.871	0.729	0.809	0.832
RF	189	0.859	0.880	0.872	0.731	0.809	0.833

RF	190	0.859	0.892	0.880	0.745	0.825	0.841
RF	191	0.861	0.893	0.881	0.748	0.827	0.844
RF	192	0.859	0.889	0.878	0.742	0.821	0.840
RF	193	0.870	0.884	0.879	0.745	0.816	0.842
RF	194	0.870	0.884	0.879	0.745	0.816	0.842
RF	195	0.868	0.879	0.875	0.737	0.809	0.837
RF	196	0.879	0.885	0.883	0.754	0.819	0.848
RF	197	0.852	0.885	0.873	0.731	0.815	0.833
RF	198	0.872	0.888	0.882	0.752	0.822	0.846
RF	199	0.870	0.883	0.878	0.743	0.814	0.841
RF	200	0.874	0.887	0.882	0.752	0.820	0.846
RF	201	0.868	0.888	0.880	0.748	0.821	0.844
RF	202	0.859	0.897	0.883	0.751	0.832	0.845
RF	203	0.868	0.887	0.880	0.746	0.819	0.843
RF	204	0.872	0.884	0.880	0.747	0.816	0.843
RF	205	0.872	0.881	0.878	0.744	0.813	0.842
RF	206	0.852	0.880	0.870	0.725	0.808	0.830
RF	207	0.861	0.889	0.879	0.744	0.821	0.841
RF	208	0.870	0.887	0.880	0.748	0.820	0.844
RF	209	0.861	0.885	0.876	0.739	0.816	0.838
RF	210	0.868	0.875	0.872	0.732	0.804	0.835
RF	211	0.852	0.877	0.868	0.722	0.805	0.828
RF	212	0.855	0.887	0.875	0.735	0.817	0.835
RF	213	0.861	0.887	0.877	0.740	0.818	0.839
RF	214	0.855	0.887	0.875	0.735	0.817	0.835

RF	215	0.870	0.881	0.877	0.742	0.813	0.840
RF	216	0.852	0.884	0.872	0.730	0.813	0.832
RF	217	0.855	0.884	0.873	0.732	0.813	0.834
RF	218	0.859	0.877	0.871	0.728	0.806	0.832
RF	219	0.855	0.884	0.873	0.732	0.813	0.834
RF	220	0.835	0.880	0.863	0.710	0.805	0.819
RF	221	0.859	0.884	0.875	0.735	0.814	0.836
RF	222	0.850	0.885	0.872	0.729	0.814	0.832
RF	223	0.852	0.880	0.870	0.725	0.808	0.830
RF	224	0.855	0.881	0.871	0.728	0.810	0.832
RF	225	0.868	0.887	0.880	0.746	0.819	0.843
RF	226	0.852	0.877	0.868	0.722	0.805	0.828
RF	227	0.837	0.876	0.862	0.707	0.800	0.818
RF	228	0.848	0.889	0.874	0.732	0.819	0.833
RF	229	0.846	0.874	0.863	0.712	0.798	0.821
RF	230	0.859	0.875	0.869	0.725	0.802	0.830
RF	231	0.848	0.884	0.871	0.726	0.812	0.830
RF	232	0.866	0.881	0.876	0.738	0.812	0.838
RF	233	0.848	0.877	0.867	0.718	0.804	0.825
RF	234	0.852	0.883	0.871	0.728	0.811	0.831
RF	235	0.846	0.880	0.867	0.719	0.807	0.826
RF	236	0.830	0.879	0.861	0.704	0.802	0.816
RF	237	0.848	0.885	0.871	0.727	0.814	0.831
RF	238	0.850	0.887	0.873	0.731	0.816	0.833
RF	239	0.844	0.880	0.867	0.717	0.806	0.825

RF	240	0.822	0.881	0.859	0.700	0.804	0.813
RF	241	0.839	0.880	0.865	0.714	0.805	0.822
RF	242	0.846	0.880	0.867	0.719	0.807	0.826
RF	243	0.848	0.883	0.870	0.724	0.811	0.829
RF	244	0.844	0.879	0.866	0.716	0.805	0.824
RF	245	0.841	0.881	0.867	0.717	0.808	0.824
RF	246	0.859	0.877	0.871	0.728	0.806	0.832
RF	247	0.852	0.880	0.870	0.725	0.808	0.830
RF	248	0.846	0.881	0.868	0.721	0.808	0.827
RF	249	0.848	0.876	0.866	0.717	0.802	0.824
RF	250	0.846	0.881	0.868	0.721	0.808	0.827
RF	251	0.846	0.881	0.868	0.721	0.808	0.827
RF	252	0.855	0.880	0.871	0.727	0.808	0.831
RF	253	0.839	0.881	0.866	0.715	0.807	0.823
RF	254	0.841	0.885	0.869	0.722	0.813	0.827
RF	255	0.846	0.888	0.872	0.729	0.817	0.831
RF	256	0.855	0.876	0.868	0.722	0.803	0.828
RF	257	0.861	0.877	0.871	0.730	0.806	0.833
RF	258	0.848	0.877	0.867	0.718	0.804	0.825
RF	259	0.841	0.876	0.863	0.711	0.801	0.821
RF	260	0.844	0.879	0.866	0.716	0.805	0.824
RF	261	0.857	0.876	0.869	0.724	0.804	0.829
RF	262	0.846	0.880	0.867	0.719	0.807	0.826
RF	263	0.859	0.883	0.874	0.734	0.813	0.835
RF	264	0.855	0.880	0.871	0.727	0.808	0.831

RF	265	0.835	0.872	0.858	0.700	0.795	0.814
RF	266	0.835	0.885	0.867	0.716	0.812	0.823
RF	267	0.852	0.874	0.866	0.717	0.800	0.825
RF	268	0.852	0.883	0.871	0.728	0.811	0.831
RF	269	0.850	0.875	0.866	0.717	0.801	0.825
RF	270	0.844	0.872	0.862	0.708	0.796	0.819
RF	271	0.848	0.872	0.863	0.712	0.797	0.822
RF	272	0.855	0.876	0.868	0.722	0.803	0.828
RF	273	0.857	0.880	0.871	0.729	0.809	0.832
RF	274	0.841	0.874	0.862	0.708	0.797	0.819
RF	275	0.850	0.881	0.870	0.725	0.809	0.829
RF	276	0.846	0.883	0.869	0.722	0.810	0.828
RF	277	0.846	0.893	0.876	0.735	0.824	0.835
RF	278	0.844	0.884	0.869	0.722	0.811	0.827
RF	279	0.846	0.875	0.864	0.713	0.800	0.822
RF	280	0.848	0.866	0.859	0.704	0.789	0.817
RF	281	0.848	0.884	0.871	0.726	0.812	0.830
RF	282	0.830	0.880	0.862	0.706	0.804	0.817
RF	283	0.835	0.881	0.864	0.711	0.806	0.820
RF	284	0.850	0.880	0.869	0.723	0.808	0.828
RF	285	0.839	0.879	0.864	0.712	0.804	0.821
RF	286	0.852	0.884	0.872	0.730	0.813	0.832
RF	287	0.852	0.872	0.865	0.716	0.798	0.824
RF	288	0.848	0.871	0.862	0.710	0.795	0.821
RF	289	0.846	0.884	0.870	0.724	0.812	0.828

RF	290	0.844	0.876	0.864	0.713	0.801	0.822
RF	291	0.835	0.871	0.857	0.699	0.793	0.813
RF	292	0.837	0.879	0.863	0.710	0.803	0.820
RF	293	0.835	0.877	0.862	0.707	0.801	0.818
RF	294	0.833	0.864	0.853	0.689	0.784	0.808
RF	295	0.841	0.876	0.863	0.711	0.801	0.821
RF	296	0.839	0.877	0.863	0.710	0.802	0.820
RF	297	0.830	0.881	0.862	0.708	0.806	0.818
RF	298	0.841	0.876	0.863	0.711	0.801	0.821
RF	299	0.828	0.867	0.853	0.688	0.787	0.807
RF	300	0.835	0.876	0.861	0.705	0.800	0.817
RF	301	0.830	0.885	0.865	0.712	0.811	0.820
RF	302	0.835	0.874	0.859	0.702	0.796	0.815
RF	303	0.837	0.870	0.857	0.699	0.792	0.814
RF	304	0.828	0.872	0.856	0.695	0.793	0.810
RF	305	0.837	0.887	0.868	0.720	0.814	0.825
RF	306	0.835	0.881	0.864	0.711	0.806	0.820
RF	307	0.824	0.875	0.856	0.694	0.796	0.810
RF	308	0.833	0.876	0.860	0.703	0.799	0.816
RF	309	0.835	0.880	0.863	0.710	0.805	0.819
RF	310	0.824	0.872	0.854	0.691	0.792	0.808
RF	311	0.839	0.874	0.861	0.706	0.797	0.818
RF	312	0.824	0.866	0.850	0.683	0.784	0.803
RF	313	0.850	0.881	0.870	0.725	0.809	0.829
RF	314	0.846	0.879	0.867	0.718	0.805	0.825

RF	315	0.837	0.867	0.856	0.696	0.788	0.812
RF	316	0.826	0.874	0.856	0.694	0.794	0.810
RF	317	0.833	0.879	0.862	0.706	0.803	0.817
RF	318	0.839	0.875	0.862	0.707	0.799	0.818
RF	319	0.835	0.880	0.863	0.710	0.805	0.819
RF	320	0.824	0.870	0.853	0.688	0.789	0.806
RF	321	0.835	0.874	0.859	0.702	0.796	0.815
RF	322	0.835	0.864	0.853	0.691	0.785	0.809
RF	323	0.833	0.866	0.853	0.691	0.786	0.809
RF	324	0.826	0.875	0.857	0.696	0.796	0.811
RF	325	0.828	0.870	0.854	0.691	0.790	0.809
RF	326	0.826	0.872	0.855	0.693	0.793	0.809
RF	327	0.850	0.862	0.857	0.702	0.785	0.816
RF	328	0.839	0.876	0.862	0.709	0.800	0.819
RF	329	0.833	0.868	0.855	0.694	0.789	0.810
RF	330	0.824	0.875	0.856	0.694	0.796	0.810
RF	331	0.830	0.875	0.858	0.700	0.797	0.813
RF	332	0.830	0.875	0.858	0.700	0.797	0.813
RF	333	0.837	0.874	0.860	0.704	0.797	0.816
RF	334	0.826	0.871	0.854	0.691	0.791	0.808
RF	335	0.837	0.879	0.863	0.710	0.803	0.820
RF	336	0.844	0.871	0.861	0.706	0.795	0.818
RF	337	0.830	0.880	0.862	0.706	0.804	0.817
RF	338	0.852	0.871	0.864	0.714	0.796	0.823
RF	339	0.844	0.876	0.864	0.713	0.801	0.822

RF	340	0.835	0.872	0.858	0.700	0.795	0.814
RF	341	0.844	0.890	0.873	0.730	0.820	0.832
RF	342	0.828	0.876	0.858	0.699	0.798	0.813
RF	343	0.835	0.867	0.855	0.694	0.788	0.811
RF	344	0.839	0.880	0.865	0.714	0.805	0.822
RF	345	0.846	0.871	0.862	0.708	0.795	0.820
RF	346	0.837	0.871	0.858	0.701	0.793	0.815
RF	347	0.844	0.875	0.863	0.711	0.800	0.821
RF	348	0.837	0.876	0.862	0.707	0.800	0.818
RF	349	0.846	0.870	0.861	0.707	0.793	0.819
RF	350	0.833	0.859	0.849	0.683	0.778	0.804
RF	351	0.844	0.868	0.859	0.703	0.791	0.817
RF	352	0.841	0.870	0.859	0.703	0.793	0.816
RF	353	0.844	0.862	0.855	0.696	0.783	0.812
RF	354	0.822	0.876	0.856	0.694	0.797	0.809
RF	355	0.841	0.884	0.868	0.720	0.811	0.826
RF	356	0.844	0.879	0.866	0.716	0.805	0.824
RF	357	0.817	0.883	0.858	0.698	0.805	0.811
RF	358	0.833	0.867	0.854	0.692	0.788	0.809
RF	359	0.835	0.879	0.862	0.708	0.803	0.819
RF	360	0.839	0.867	0.857	0.698	0.789	0.813
RF	361	0.837	0.872	0.859	0.702	0.795	0.815
RF	362	0.835	0.872	0.858	0.700	0.795	0.814
RF	363	0.822	0.874	0.854	0.690	0.794	0.807
RF	364	0.828	0.876	0.858	0.699	0.798	0.813

RF	365	0.837	0.870	0.857	0.699	0.792	0.814
RF	366	0.830	0.881	0.862	0.708	0.806	0.818
RF	367	0.837	0.885	0.867	0.718	0.812	0.824
RF	368	0.839	0.862	0.853	0.692	0.782	0.810
RF	369	0.830	0.876	0.859	0.701	0.799	0.814
RF	370	0.833	0.864	0.853	0.689	0.784	0.808
SVM	1	0.764	0.808	0.792	0.564	0.702	0.732
SVM	2	0.767	0.817	0.799	0.576	0.713	0.739
SVM	3	0.771	0.828	0.807	0.592	0.726	0.748
SVM	4	0.769	0.821	0.802	0.583	0.718	0.743
SVM	5	0.769	0.833	0.809	0.596	0.732	0.750
SVM	6	0.758	0.841	0.810	0.596	0.738	0.748
SVM	7	0.778	0.850	0.823	0.624	0.754	0.766
SVM	8	0.813	0.859	0.842	0.666	0.774	0.793
SVM	9	0.815	0.859	0.843	0.668	0.774	0.794
SVM	10	0.813	0.850	0.836	0.655	0.762	0.787
SVM	11	0.806	0.855	0.837	0.655	0.767	0.786
SVM	12	0.822	0.854	0.842	0.667	0.769	0.794
SVM	13	0.835	0.879	0.862	0.708	0.803	0.819
SVM	14	0.846	0.875	0.864	0.713	0.800	0.822
SVM	15	0.872	0.874	0.873	0.735	0.803	0.836
SVM	16	0.859	0.875	0.869	0.725	0.802	0.830
SVM	17	0.866	0.870	0.868	0.724	0.797	0.830
SVM	18	0.841	0.871	0.860	0.705	0.794	0.817
SVM	19	0.841	0.875	0.862	0.709	0.799	0.820

SVM	20	0.863	0.875	0.871	0.728	0.803	0.832
SVM	21	0.855	0.877	0.869	0.724	0.805	0.829
SVM	22	0.881	0.901	0.894	0.775	0.840	0.860
SVM	23	0.879	0.894	0.889	0.765	0.831	0.854
SVM	24	0.883	0.894	0.890	0.769	0.832	0.857
SVM	25	0.896	0.897	0.897	0.783	0.837	0.866
SVM	26	0.896	0.897	0.897	0.783	0.837	0.866
SVM	27	0.892	0.902	0.898	0.786	0.844	0.867
SVM	28	0.892	0.898	0.896	0.781	0.839	0.864
SVM	29	0.894	0.901	0.898	0.786	0.842	0.868
SVM	30	0.890	0.900	0.896	0.781	0.840	0.864
SVM	31	0.883	0.898	0.893	0.774	0.837	0.860
SVM	32	0.896	0.900	0.898	0.787	0.841	0.868
SVM	33	0.894	0.902	0.899	0.788	0.844	0.868
SVM	34	0.892	0.902	0.898	0.786	0.844	0.867
SVM	35	0.896	0.896	0.896	0.782	0.836	0.865
SVM	36	0.896	0.896	0.896	0.782	0.836	0.865
SVM	37	0.888	0.892	0.890	0.770	0.829	0.857
SVM	38	0.881	0.896	0.890	0.769	0.833	0.857
SVM	39	0.883	0.894	0.890	0.769	0.832	0.857
SVM	40	0.890	0.896	0.894	0.776	0.835	0.861
SVM	41	0.892	0.893	0.893	0.775	0.832	0.861
SVM	42	0.892	0.893	0.893	0.775	0.832	0.861
SVM	43	0.892	0.898	0.896	0.781	0.839	0.864
SVM	44	0.894	0.900	0.898	0.785	0.841	0.867

SVM	45	0.894	0.900	0.898	0.785	0.841	0.867
SVM	46	0.894	0.900	0.898	0.785	0.841	0.867
SVM	47	0.894	0.900	0.898	0.785	0.841	0.867
SVM	48	0.899	0.898	0.898	0.787	0.840	0.868
SVM	49	0.892	0.897	0.895	0.780	0.837	0.864
SVM	50	0.892	0.893	0.893	0.775	0.832	0.861
SVM	51	0.894	0.894	0.894	0.778	0.834	0.863
SVM	52	0.894	0.893	0.894	0.777	0.832	0.862
SVM	53	0.888	0.892	0.890	0.770	0.829	0.857
SVM	54	0.894	0.894	0.894	0.778	0.834	0.863
SVM	55	0.894	0.893	0.894	0.777	0.832	0.862
SVM	56	0.890	0.888	0.889	0.767	0.824	0.856
SVM	57	0.890	0.887	0.888	0.765	0.823	0.855
SVM	58	0.899	0.885	0.890	0.771	0.823	0.859
SVM	59	0.890	0.888	0.889	0.767	0.824	0.856
SVM	60	0.894	0.883	0.887	0.765	0.819	0.855
SVM	61	0.896	0.884	0.889	0.768	0.821	0.857
SVM	62	0.896	0.884	0.889	0.768	0.821	0.857
SVM	63	0.894	0.879	0.885	0.760	0.814	0.852
SVM	64	0.892	0.879	0.884	0.758	0.813	0.851
SVM	65	0.885	0.880	0.882	0.754	0.814	0.848
SVM	66	0.890	0.885	0.887	0.764	0.821	0.854
SVM	67	0.890	0.879	0.883	0.756	0.813	0.850
SVM	68	0.885	0.879	0.881	0.752	0.812	0.847
SVM	69	0.885	0.883	0.884	0.757	0.817	0.850

SVM	70	0.885	0.881	0.883	0.755	0.815	0.849
SVM	71	0.877	0.885	0.882	0.752	0.819	0.847
SVM	72	0.881	0.880	0.880	0.750	0.813	0.846
SVM	73	0.883	0.883	0.883	0.755	0.817	0.849
SVM	74	0.881	0.883	0.882	0.753	0.816	0.847
SVM	75	0.883	0.872	0.876	0.743	0.804	0.842
SVM	76	0.883	0.872	0.876	0.743	0.804	0.842
SVM	77	0.885	0.871	0.876	0.743	0.802	0.842
SVM	78	0.883	0.871	0.876	0.741	0.802	0.841
SVM	79	0.877	0.867	0.871	0.731	0.796	0.834
SVM	80	0.883	0.867	0.873	0.737	0.797	0.838
SVM	81	0.872	0.874	0.873	0.735	0.803	0.836
SVM	82	0.874	0.874	0.874	0.737	0.804	0.838
SVM	83	0.861	0.867	0.865	0.717	0.793	0.826
SVM	84	0.866	0.877	0.873	0.733	0.807	0.835
SVM	85	0.866	0.877	0.873	0.733	0.807	0.835
SVM	86	0.861	0.877	0.871	0.730	0.806	0.833
SVM	87	0.868	0.876	0.873	0.734	0.806	0.836
SVM	88	0.868	0.875	0.872	0.732	0.804	0.835
SVM	89	0.868	0.875	0.872	0.732	0.804	0.835
SVM	90	0.870	0.877	0.875	0.737	0.808	0.838
SVM	91	0.874	0.880	0.878	0.744	0.812	0.842
SVM	92	0.872	0.880	0.877	0.742	0.811	0.841
SVM	93	0.879	0.880	0.880	0.748	0.813	0.844
SVM	94	0.874	0.880	0.878	0.744	0.812	0.842

SVM	95	0.874	0.881	0.879	0.746	0.814	0.843
SVM	96	0.870	0.877	0.875	0.737	0.808	0.838
SVM	97	0.866	0.887	0.879	0.744	0.819	0.842
SVM	98	0.863	0.885	0.877	0.741	0.817	0.839
SVM	99	0.863	0.885	0.877	0.741	0.817	0.839
SVM	100	0.863	0.888	0.879	0.744	0.820	0.841
SVM	101	0.861	0.889	0.879	0.744	0.821	0.841
SVM	102	0.863	0.889	0.880	0.746	0.822	0.842
SVM	103	0.866	0.889	0.880	0.747	0.822	0.843
SVM	104	0.866	0.890	0.881	0.749	0.824	0.844
SVM	105	0.872	0.894	0.886	0.759	0.830	0.851
SVM	106	0.872	0.896	0.887	0.761	0.832	0.852
SVM	107	0.868	0.898	0.887	0.760	0.835	0.851
SVM	108	0.866	0.896	0.885	0.755	0.831	0.848
SVM	109	0.866	0.896	0.885	0.755	0.831	0.848
SVM	110	0.866	0.896	0.885	0.755	0.831	0.848
SVM	111	0.866	0.898	0.886	0.758	0.834	0.850
SVM	112	0.863	0.898	0.885	0.757	0.834	0.848
SVM	113	0.861	0.900	0.885	0.756	0.835	0.848
SVM	114	0.857	0.892	0.879	0.743	0.824	0.840
SVM	115	0.859	0.892	0.880	0.745	0.825	0.841
SVM	116	0.855	0.890	0.877	0.739	0.822	0.838
SVM	117	0.857	0.896	0.881	0.748	0.829	0.843
SVM	118	0.857	0.896	0.881	0.748	0.829	0.843
SVM	119	0.863	0.889	0.880	0.746	0.822	0.842

SVM	120	0.861	0.889	0.879	0.744	0.821	0.841
SVM	121	0.863	0.893	0.882	0.750	0.827	0.845
SVM	122	0.866	0.893	0.883	0.752	0.827	0.846
SVM	123	0.859	0.893	0.880	0.746	0.826	0.842
SVM	124	0.839	0.887	0.869	0.721	0.814	0.826
SVM	125	0.839	0.887	0.869	0.721	0.814	0.826
SVM	126	0.833	0.887	0.867	0.716	0.813	0.823
SVM	127	0.835	0.887	0.867	0.718	0.813	0.824
SVM	128	0.835	0.885	0.867	0.716	0.812	0.823
SVM	129	0.833	0.883	0.864	0.711	0.808	0.820
SVM	130	0.833	0.883	0.864	0.711	0.808	0.820
SVM	131	0.850	0.893	0.877	0.739	0.825	0.837
SVM	132	0.855	0.892	0.878	0.741	0.824	0.839
SVM	133	0.855	0.890	0.877	0.739	0.822	0.838
SVM	134	0.852	0.889	0.876	0.736	0.820	0.836
SVM	135	0.852	0.889	0.876	0.736	0.820	0.836
SVM	136	0.855	0.885	0.874	0.733	0.815	0.834
SVM	137	0.855	0.890	0.877	0.739	0.822	0.838
SVM	138	0.855	0.892	0.878	0.741	0.824	0.839
SVM	139	0.855	0.890	0.877	0.739	0.822	0.838
SVM	140	0.852	0.890	0.876	0.738	0.822	0.837
SVM	141	0.852	0.881	0.871	0.727	0.810	0.830
SVM	142	0.857	0.883	0.873	0.732	0.812	0.834
SVM	143	0.861	0.890	0.880	0.745	0.823	0.842
SVM	144	0.857	0.888	0.876	0.738	0.819	0.837

SVM	145	0.859	0.890	0.879	0.743	0.823	0.841
SVM	146	0.859	0.890	0.879	0.743	0.823	0.841
SVM	147	0.859	0.889	0.878	0.742	0.821	0.840
SVM	148	0.859	0.889	0.878	0.742	0.821	0.840
SVM	149	0.859	0.890	0.879	0.743	0.823	0.841
SVM	150	0.859	0.884	0.875	0.735	0.814	0.836
SVM	151	0.861	0.883	0.875	0.736	0.813	0.836
SVM	152	0.861	0.883	0.875	0.736	0.813	0.836
SVM	153	0.863	0.887	0.878	0.742	0.818	0.840
SVM	154	0.863	0.885	0.877	0.741	0.817	0.839
SVM	155	0.866	0.885	0.878	0.743	0.817	0.841
SVM	156	0.866	0.885	0.878	0.743	0.817	0.841
SVM	157	0.863	0.887	0.878	0.742	0.818	0.840
SVM	158	0.863	0.887	0.878	0.742	0.818	0.840
SVM	159	0.863	0.887	0.878	0.742	0.818	0.840
SVM	160	0.861	0.883	0.875	0.736	0.813	0.836
SVM	161	0.852	0.885	0.873	0.731	0.815	0.833
SVM	162	0.852	0.887	0.874	0.733	0.816	0.834
SVM	163	0.863	0.887	0.878	0.742	0.818	0.840
SVM	164	0.861	0.876	0.871	0.728	0.805	0.832
SVM	165	0.859	0.880	0.872	0.731	0.809	0.833
SVM	166	0.859	0.883	0.874	0.734	0.813	0.835
SVM	167	0.859	0.883	0.874	0.734	0.813	0.835
SVM	168	0.866	0.879	0.874	0.735	0.809	0.836
SVM	169	0.866	0.877	0.873	0.733	0.807	0.835

SVM	170	0.868	0.877	0.874	0.735	0.807	0.837
SVM	171	0.868	0.875	0.872	0.732	0.804	0.835
SVM	172	0.861	0.874	0.869	0.725	0.801	0.830
SVM	173	0.857	0.874	0.867	0.721	0.800	0.828
SVM	174	0.857	0.876	0.869	0.724	0.804	0.829
SVM	175	0.859	0.876	0.870	0.726	0.804	0.831
SVM	176	0.863	0.874	0.870	0.727	0.802	0.831
SVM	177	0.863	0.874	0.870	0.727	0.802	0.831
SVM	178	0.863	0.875	0.871	0.728	0.803	0.832
SVM	179	0.863	0.874	0.870	0.727	0.802	0.831
SVM	180	0.863	0.874	0.870	0.727	0.802	0.831
SVM	181	0.866	0.885	0.878	0.743	0.817	0.841
SVM	182	0.870	0.881	0.877	0.742	0.813	0.840
SVM	183	0.870	0.880	0.876	0.740	0.811	0.840
SVM	184	0.870	0.880	0.876	0.740	0.811	0.840
SVM	185	0.868	0.884	0.878	0.743	0.816	0.841
SVM	186	0.868	0.884	0.878	0.743	0.816	0.841
SVM	187	0.870	0.884	0.879	0.745	0.816	0.842
SVM	188	0.872	0.871	0.871	0.732	0.800	0.835
SVM	189	0.872	0.871	0.871	0.732	0.800	0.835
SVM	190	0.872	0.871	0.871	0.732	0.800	0.835
SVM	191	0.868	0.871	0.870	0.728	0.799	0.832
SVM	192	0.868	0.871	0.870	0.728	0.799	0.832
SVM	193	0.877	0.871	0.873	0.735	0.801	0.837
SVM	194	0.877	0.874	0.875	0.738	0.804	0.839

SVM	195	0.872	0.876	0.875	0.738	0.807	0.838
SVM	196	0.872	0.877	0.876	0.739	0.808	0.839
SVM	197	0.872	0.877	0.876	0.739	0.808	0.839
SVM	198	0.870	0.874	0.872	0.733	0.803	0.835
SVM	199	0.877	0.876	0.876	0.742	0.807	0.841
SVM	200	0.872	0.876	0.875	0.738	0.807	0.838
SVM	201	0.870	0.875	0.873	0.734	0.804	0.836
SVM	202	0.870	0.874	0.872	0.733	0.803	0.835
SVM	203	0.874	0.866	0.869	0.727	0.794	0.832
SVM	204	0.868	0.867	0.867	0.723	0.794	0.829
SVM	205	0.874	0.867	0.870	0.729	0.796	0.833
SVM	206	0.863	0.871	0.868	0.724	0.798	0.830
SVM	207	0.863	0.864	0.864	0.716	0.790	0.825
SVM	208	0.866	0.867	0.867	0.721	0.794	0.828
SVM	209	0.863	0.870	0.867	0.722	0.797	0.829
SVM	210	0.872	0.870	0.871	0.730	0.798	0.834
SVM	211	0.872	0.867	0.869	0.727	0.795	0.832
SVM	212	0.874	0.864	0.868	0.726	0.792	0.831
SVM	213	0.874	0.864	0.868	0.726	0.792	0.831
SVM	214	0.872	0.864	0.867	0.724	0.792	0.830
SVM	215	0.870	0.864	0.867	0.722	0.792	0.829
SVM	216	0.868	0.862	0.864	0.717	0.788	0.826
SVM	217	0.866	0.857	0.860	0.709	0.781	0.821
SVM	218	0.870	0.872	0.871	0.731	0.801	0.834
SVM	219	0.870	0.872	0.871	0.731	0.801	0.834

SVM	220	0.872	0.870	0.871	0.730	0.798	0.834
SVM	221	0.874	0.864	0.868	0.726	0.792	0.831
SVM	222	0.877	0.866	0.870	0.729	0.794	0.834
SVM	223	0.877	0.864	0.869	0.728	0.793	0.833
SVM	224	0.877	0.867	0.871	0.731	0.796	0.834
SVM	225	0.877	0.866	0.870	0.729	0.794	0.834
SVM	226	0.877	0.866	0.870	0.729	0.794	0.834
SVM	227	0.877	0.868	0.871	0.732	0.798	0.835
SVM	228	0.874	0.877	0.876	0.741	0.809	0.840
SVM	229	0.877	0.879	0.878	0.745	0.811	0.842
SVM	230	0.879	0.879	0.879	0.746	0.811	0.844
SVM	231	0.879	0.879	0.879	0.746	0.811	0.844
SVM	232	0.874	0.875	0.875	0.738	0.805	0.838
SVM	233	0.874	0.875	0.875	0.738	0.805	0.838
SVM	234	0.885	0.872	0.877	0.745	0.804	0.843
SVM	235	0.885	0.872	0.877	0.745	0.804	0.843
SVM	236	0.885	0.872	0.877	0.745	0.804	0.843
SVM	237	0.885	0.872	0.877	0.745	0.804	0.843
SVM	238	0.885	0.872	0.877	0.745	0.804	0.843
SVM	239	0.885	0.872	0.877	0.745	0.804	0.843
SVM	240	0.883	0.872	0.876	0.743	0.804	0.842
SVM	241	0.881	0.872	0.876	0.741	0.803	0.840
SVM	242	0.883	0.872	0.876	0.743	0.804	0.842
SVM	243	0.883	0.874	0.877	0.744	0.805	0.842
SVM	244	0.883	0.874	0.877	0.744	0.805	0.842

SVM	245	0.883	0.872	0.876	0.743	0.804	0.842
SVM	246	0.879	0.872	0.875	0.739	0.803	0.839
SVM	247	0.879	0.871	0.874	0.737	0.801	0.838
SVM	248	0.877	0.871	0.873	0.735	0.801	0.837
SVM	249	0.877	0.871	0.873	0.735	0.801	0.837
SVM	250	0.877	0.871	0.873	0.735	0.801	0.837
SVM	251	0.877	0.871	0.873	0.735	0.801	0.837
SVM	252	0.877	0.868	0.871	0.732	0.798	0.835
SVM	253	0.877	0.868	0.871	0.732	0.798	0.835
SVM	254	0.877	0.870	0.872	0.734	0.799	0.836
SVM	255	0.877	0.870	0.872	0.734	0.799	0.836
SVM	256	0.877	0.870	0.872	0.734	0.799	0.836
SVM	257	0.879	0.868	0.872	0.734	0.798	0.836
SVM	258	0.877	0.871	0.873	0.735	0.801	0.837
SVM	259	0.877	0.871	0.873	0.735	0.801	0.837
SVM	260	0.879	0.872	0.875	0.739	0.803	0.839
SVM	261	0.879	0.871	0.874	0.737	0.801	0.838
SVM	262	0.879	0.868	0.872	0.734	0.798	0.836
SVM	263	0.881	0.870	0.874	0.738	0.800	0.839
SVM	264	0.881	0.870	0.874	0.738	0.800	0.839
SVM	265	0.881	0.870	0.874	0.738	0.800	0.839
SVM	266	0.881	0.870	0.874	0.738	0.800	0.839
SVM	267	0.874	0.874	0.874	0.737	0.804	0.838
SVM	268	0.874	0.875	0.875	0.738	0.805	0.838
SVM	269	0.874	0.875	0.875	0.738	0.805	0.838

SVM	270	0.877	0.874	0.875	0.738	0.804	0.839
SVM	271	0.877	0.874	0.875	0.738	0.804	0.839
SVM	272	0.877	0.872	0.874	0.737	0.802	0.838
SVM	273	0.877	0.872	0.874	0.737	0.802	0.838
SVM	274	0.870	0.874	0.872	0.733	0.803	0.835
SVM	275	0.870	0.874	0.872	0.733	0.803	0.835
SVM	276	0.870	0.872	0.871	0.731	0.801	0.834
SVM	277	0.872	0.868	0.870	0.729	0.797	0.833
SVM	278	0.872	0.867	0.869	0.727	0.795	0.832
SVM	279	0.866	0.867	0.867	0.721	0.794	0.828
SVM	280	0.863	0.871	0.868	0.724	0.798	0.830
SVM	281	0.859	0.875	0.869	0.725	0.802	0.830
SVM	282	0.859	0.874	0.868	0.723	0.801	0.829
SVM	283	0.863	0.874	0.870	0.727	0.802	0.831
SVM	284	0.863	0.875	0.871	0.728	0.803	0.832
SVM	285	0.866	0.874	0.871	0.729	0.802	0.833
SVM	286	0.866	0.874	0.871	0.729	0.802	0.833
SVM	287	0.868	0.874	0.871	0.731	0.802	0.834
SVM	288	0.868	0.875	0.872	0.732	0.804	0.835
SVM	289	0.861	0.871	0.867	0.722	0.798	0.828
SVM	290	0.868	0.864	0.866	0.720	0.791	0.828
SVM	291	0.848	0.876	0.866	0.717	0.802	0.824
SVM	292	0.848	0.875	0.865	0.715	0.800	0.824
SVM	293	0.848	0.874	0.864	0.713	0.799	0.823
SVM	294	0.848	0.874	0.864	0.713	0.799	0.823

SVM	295	0.848	0.874	0.864	0.713	0.799	0.823
SVM	296	0.848	0.874	0.864	0.713	0.799	0.823
SVM	297	0.848	0.876	0.866	0.717	0.802	0.824
SVM	298	0.850	0.876	0.867	0.718	0.802	0.826
SVM	299	0.844	0.876	0.864	0.713	0.801	0.822
SVM	300	0.846	0.871	0.862	0.708	0.795	0.820
SVM	301	0.846	0.871	0.862	0.708	0.795	0.820
SVM	302	0.848	0.871	0.862	0.710	0.795	0.821
SVM	303	0.848	0.871	0.862	0.710	0.795	0.821
SVM	304	0.848	0.872	0.863	0.712	0.797	0.822
SVM	305	0.844	0.872	0.862	0.708	0.796	0.819
SVM	306	0.844	0.872	0.862	0.708	0.796	0.819
SVM	307	0.848	0.874	0.864	0.713	0.799	0.823
SVM	308	0.848	0.875	0.865	0.715	0.800	0.824
SVM	309	0.848	0.875	0.865	0.715	0.800	0.824
SVM	310	0.844	0.877	0.865	0.714	0.803	0.823
SVM	311	0.850	0.874	0.865	0.715	0.799	0.824
SVM	312	0.855	0.871	0.865	0.716	0.797	0.825
SVM	313	0.852	0.872	0.865	0.716	0.798	0.824
SVM	314	0.852	0.871	0.864	0.714	0.796	0.823
SVM	315	0.848	0.870	0.862	0.709	0.794	0.820
SVM	316	0.850	0.872	0.864	0.714	0.798	0.823
SVM	317	0.852	0.871	0.864	0.714	0.796	0.823
SVM	318	0.861	0.870	0.867	0.720	0.796	0.828
SVM	319	0.857	0.872	0.867	0.720	0.799	0.827

SVM	320	0.855	0.872	0.866	0.718	0.798	0.826
SVM	321	0.855	0.872	0.866	0.718	0.798	0.826
SVM	322	0.855	0.872	0.866	0.718	0.798	0.826
SVM	323	0.850	0.871	0.863	0.712	0.796	0.822
SVM	324	0.841	0.872	0.861	0.706	0.796	0.818
SVM	325	0.841	0.874	0.862	0.708	0.797	0.819
SVM	326	0.844	0.870	0.860	0.705	0.793	0.818
SVM	327	0.844	0.870	0.860	0.705	0.793	0.818
SVM	328	0.841	0.870	0.859	0.703	0.793	0.816
SVM	329	0.841	0.870	0.859	0.703	0.793	0.816
SVM	330	0.841	0.870	0.859	0.703	0.793	0.816
SVM	331	0.861	0.874	0.869	0.725	0.801	0.830
SVM	332	0.855	0.868	0.863	0.713	0.793	0.823
SVM	333	0.852	0.868	0.862	0.711	0.793	0.822
SVM	334	0.868	0.870	0.869	0.726	0.798	0.831
SVM	335	0.870	0.866	0.867	0.724	0.793	0.830
SVM	336	0.870	0.866	0.867	0.724	0.793	0.830
SVM	337	0.870	0.866	0.867	0.724	0.793	0.830
SVM	338	0.868	0.868	0.868	0.725	0.796	0.830
SVM	339	0.868	0.867	0.867	0.723	0.794	0.829
SVM	340	0.866	0.867	0.867	0.721	0.794	0.828
SVM	341	0.859	0.863	0.862	0.711	0.788	0.822
SVM	342	0.859	0.863	0.862	0.711	0.788	0.822
SVM	343	0.861	0.859	0.860	0.708	0.784	0.821
SVM	344	0.855	0.862	0.859	0.705	0.785	0.819

SVM	345	0.855	0.862	0.859	0.705	0.785	0.819
SVM	346	0.859	0.845	0.850	0.690	0.766	0.810
SVM	347	0.859	0.845	0.850	0.690	0.766	0.810
SVM	348	0.857	0.847	0.851	0.691	0.769	0.810
SVM	349	0.857	0.847	0.851	0.691	0.769	0.810
SVM	350	0.857	0.847	0.851	0.691	0.769	0.810
SVM	351	0.850	0.853	0.852	0.691	0.774	0.810
SVM	352	0.848	0.853	0.851	0.689	0.773	0.809
SVM	353	0.844	0.862	0.855	0.696	0.783	0.812
SVM	354	0.852	0.867	0.862	0.710	0.791	0.821
SVM	355	0.852	0.862	0.858	0.704	0.785	0.817
SVM	356	0.852	0.862	0.858	0.704	0.785	0.817
SVM	357	0.850	0.862	0.857	0.702	0.785	0.816
SVM	358	0.852	0.864	0.860	0.707	0.788	0.819
SVM	359	0.852	0.864	0.860	0.707	0.788	0.819
SVM	360	0.852	0.866	0.861	0.708	0.790	0.820
SVM	361	0.852	0.863	0.859	0.705	0.787	0.818
SVM	362	0.846	0.866	0.858	0.702	0.789	0.816
SVM	363	0.846	0.866	0.858	0.702	0.789	0.816
SVM	364	0.859	0.868	0.865	0.717	0.794	0.825
SVM	365	0.857	0.866	0.862	0.712	0.791	0.822
SVM	366	0.857	0.868	0.864	0.715	0.794	0.824
SVM	367	0.857	0.868	0.864	0.715	0.794	0.824
SVM	368	0.857	0.868	0.864	0.715	0.794	0.824
SVM	369	0.855	0.868	0.863	0.713	0.793	0.823

SVM	370	0.852	0.867	0.862	0.710	0.791	0.821

(4) IFS results on the LASSO feature list

**Classification algorithm**	**Number of features**	**SN**	**SP**	**ACC**	**MCC**	**Precision**	**F1-measure**
DT	1	0.621	0.561	0.583	0.176	0.456	0.526
DT	2	0.562	0.597	0.584	0.154	0.452	0.501
DT	3	0.604	0.596	0.599	0.193	0.469	0.528
DT	4	0.562	0.596	0.583	0.153	0.451	0.500
DT	5	0.584	0.592	0.589	0.170	0.458	0.514
DT	6	0.599	0.585	0.591	0.178	0.461	0.521
DT	7	0.562	0.584	0.576	0.141	0.444	0.496
DT	8	0.617	0.635	0.628	0.244	0.500	0.552
DT	9	0.586	0.662	0.634	0.242	0.507	0.543
DT	10	0.619	0.661	0.645	0.272	0.519	0.565
DT	11	0.615	0.661	0.644	0.268	0.518	0.562
DT	12	0.621	0.628	0.626	0.242	0.497	0.552
DT	13	0.573	0.636	0.613	0.203	0.482	0.524
DT	14	0.566	0.652	0.620	0.213	0.490	0.526
DT	15	0.588	0.665	0.636	0.247	0.510	0.546
DT	16	0.619	0.643	0.634	0.254	0.506	0.557
DT	17	0.623	0.631	0.628	0.247	0.500	0.555
DT	18	0.634	0.643	0.640	0.269	0.512	0.567
DT	19	0.612	0.677	0.653	0.282	0.529	0.567
DT	20	0.586	0.634	0.616	0.213	0.486	0.531
DT	21	0.588	0.670	0.640	0.252	0.513	0.548

DT	22	0.617	0.677	0.654	0.286	0.530	0.570
DT	23	0.634	0.643	0.640	0.269	0.512	0.567
DT	24	0.597	0.652	0.631	0.242	0.504	0.546
DT	25	0.619	0.682	0.658	0.294	0.535	0.574
DT	26	0.604	0.684	0.654	0.282	0.531	0.565
DT	27	0.641	0.677	0.663	0.309	0.540	0.586
DT	28	0.648	0.651	0.649	0.289	0.523	0.579
DT	29	0.628	0.721	0.686	0.343	0.571	0.598
DT	30	0.628	0.686	0.664	0.306	0.542	0.582
DT	31	0.617	0.700	0.669	0.311	0.549	0.581
DT	32	0.650	0.691	0.676	0.332	0.555	0.598
DT	33	0.650	0.660	0.656	0.300	0.531	0.584
DT	34	0.685	0.664	0.672	0.338	0.547	0.608
DT	35	0.659	0.675	0.669	0.324	0.546	0.597
DT	36	0.663	0.690	0.680	0.343	0.558	0.606
DT	37	0.656	0.675	0.668	0.322	0.545	0.595
DT	38	0.656	0.671	0.666	0.318	0.542	0.594
DT	39	0.661	0.692	0.681	0.344	0.560	0.606
DT	40	0.689	0.716	0.706	0.395	0.589	0.636
DT	41	0.676	0.713	0.699	0.380	0.583	0.626
DT	42	0.685	0.711	0.701	0.385	0.583	0.630
DT	43	0.667	0.726	0.704	0.385	0.591	0.627
DT	44	0.639	0.679	0.664	0.310	0.541	0.586
DT	45	0.674	0.707	0.695	0.371	0.576	0.621
DT	46	0.654	0.692	0.678	0.338	0.557	0.602

DT	47	0.681	0.681	0.681	0.351	0.558	0.613
DT	48	0.676	0.677	0.676	0.343	0.553	0.609
DT	49	0.650	0.704	0.684	0.346	0.565	0.605
DT	50	0.654	0.682	0.672	0.327	0.549	0.597
DT	51	0.676	0.695	0.688	0.361	0.567	0.617
DT	52	0.670	0.691	0.683	0.351	0.562	0.611
DT	53	0.659	0.716	0.695	0.366	0.578	0.616
DT	54	0.639	0.682	0.666	0.312	0.543	0.587
DT	55	0.628	0.703	0.675	0.324	0.556	0.589
DT	56	0.696	0.709	0.704	0.394	0.586	0.636
DT	57	0.667	0.707	0.692	0.365	0.574	0.617
DT	58	0.656	0.714	0.693	0.363	0.576	0.614
DT	59	0.707	0.748	0.733	0.446	0.625	0.663
DT	60	0.718	0.682	0.695	0.387	0.572	0.637
DT	61	0.689	0.698	0.695	0.376	0.574	0.627
DT	62	0.685	0.682	0.683	0.356	0.560	0.616
DT	63	0.678	0.712	0.699	0.381	0.582	0.627
DT	64	0.696	0.691	0.693	0.376	0.571	0.628
DT	65	0.667	0.721	0.701	0.380	0.586	0.624
DT	66	0.674	0.718	0.702	0.383	0.586	0.627
DT	67	0.716	0.709	0.712	0.413	0.593	0.649
DT	68	0.707	0.666	0.681	0.361	0.556	0.623
DT	69	0.670	0.707	0.693	0.367	0.575	0.619
DT	70	0.687	0.711	0.702	0.388	0.584	0.632
DT	71	0.663	0.709	0.692	0.363	0.574	0.616

DT	72	0.643	0.717	0.690	0.353	0.574	0.606
DT	73	0.674	0.701	0.691	0.366	0.572	0.619
DT	74	0.687	0.718	0.707	0.396	0.591	0.635
DT	75	0.665	0.711	0.694	0.367	0.576	0.618
DT	76	0.694	0.729	0.716	0.413	0.602	0.645
DT	77	0.663	0.696	0.684	0.350	0.564	0.609
DT	78	0.698	0.688	0.692	0.375	0.570	0.628
DT	79	0.674	0.674	0.674	0.338	0.550	0.606
DT	80	0.672	0.713	0.698	0.376	0.581	0.623
DT	81	0.674	0.709	0.696	0.374	0.578	0.623
DT	82	0.650	0.724	0.696	0.366	0.582	0.614
DT	83	0.665	0.690	0.681	0.345	0.559	0.608
DT	84	0.685	0.739	0.719	0.416	0.609	0.645
DT	85	0.683	0.699	0.693	0.371	0.573	0.623
DT	86	0.628	0.722	0.687	0.344	0.572	0.599
DT	87	0.672	0.707	0.694	0.369	0.575	0.620
DT	88	0.661	0.721	0.699	0.374	0.584	0.620
DT	89	0.678	0.718	0.704	0.387	0.588	0.630
DT	90	0.676	0.711	0.698	0.377	0.580	0.625
DT	91	0.663	0.691	0.681	0.345	0.559	0.607
DT	92	0.689	0.755	0.731	0.437	0.625	0.655
DT	93	0.703	0.708	0.706	0.399	0.587	0.640
DT	94	0.639	0.716	0.687	0.348	0.571	0.603
DT	95	0.687	0.730	0.714	0.408	0.601	0.641
DT	96	0.705	0.679	0.689	0.372	0.565	0.627

DT	97	0.687	0.713	0.704	0.390	0.586	0.633
DT	98	0.665	0.717	0.698	0.374	0.582	0.621
DT	99	0.700	0.720	0.713	0.409	0.597	0.644
DT	100	0.687	0.739	0.720	0.418	0.609	0.646
DT	101	0.714	0.725	0.721	0.427	0.606	0.655
DT	102	0.670	0.726	0.705	0.387	0.591	0.628
DT	103	0.705	0.705	0.705	0.399	0.586	0.640
DT	104	0.705	0.743	0.729	0.438	0.619	0.659
DT	105	0.685	0.711	0.701	0.385	0.583	0.630
DT	106	0.665	0.709	0.693	0.366	0.575	0.617
DT	107	0.678	0.716	0.702	0.385	0.586	0.629
DT	108	0.689	0.698	0.695	0.376	0.574	0.627
DT	109	0.694	0.679	0.685	0.362	0.561	0.621
DT	110	0.667	0.725	0.704	0.384	0.589	0.626
DT	111	0.656	0.725	0.699	0.374	0.585	0.619
DT	112	0.714	0.718	0.717	0.420	0.600	0.652
DT	113	0.643	0.724	0.694	0.360	0.579	0.610
DT	114	0.670	0.714	0.698	0.375	0.581	0.622
DT	115	0.676	0.720	0.704	0.387	0.588	0.629
DT	116	0.678	0.709	0.698	0.378	0.580	0.625
DT	117	0.659	0.709	0.690	0.359	0.573	0.613
DT	118	0.652	0.721	0.695	0.365	0.580	0.614
DT	119	0.683	0.698	0.692	0.370	0.572	0.622
DT	120	0.683	0.712	0.701	0.385	0.584	0.629
DT	121	0.683	0.714	0.703	0.387	0.586	0.631

DT	122	0.685	0.664	0.672	0.338	0.547	0.608
DT	123	0.678	0.712	0.699	0.381	0.582	0.627
DT	124	0.665	0.731	0.707	0.389	0.594	0.628
DT	125	0.659	0.716	0.695	0.366	0.578	0.616
DT	126	0.661	0.711	0.692	0.363	0.575	0.615
DT	127	0.692	0.705	0.700	0.386	0.581	0.632
DT	128	0.683	0.713	0.702	0.386	0.585	0.630
DT	129	0.685	0.698	0.693	0.372	0.573	0.624
DT	130	0.670	0.713	0.697	0.374	0.580	0.622
DT	131	0.703	0.716	0.711	0.407	0.594	0.644
DT	132	0.667	0.725	0.704	0.384	0.589	0.626
DT	133	0.687	0.711	0.702	0.388	0.584	0.632
DT	134	0.678	0.721	0.705	0.390	0.590	0.631
DT	135	0.648	0.711	0.687	0.350	0.570	0.606
DT	136	0.676	0.696	0.689	0.362	0.569	0.618
DT	137	0.703	0.722	0.715	0.414	0.600	0.647
DT	138	0.707	0.716	0.713	0.412	0.596	0.647
DT	139	0.683	0.721	0.707	0.394	0.592	0.634
DT	140	0.661	0.711	0.692	0.363	0.575	0.615
DT	141	0.700	0.726	0.717	0.416	0.602	0.648
DT	142	0.696	0.707	0.703	0.392	0.584	0.635
DT	143	0.692	0.711	0.704	0.392	0.586	0.634
DT	144	0.648	0.716	0.690	0.356	0.574	0.609
DT	145	0.698	0.698	0.698	0.384	0.577	0.632
DT	146	0.648	0.698	0.679	0.337	0.559	0.600

DT	147	0.676	0.694	0.687	0.360	0.566	0.616
DT	148	0.670	0.711	0.695	0.371	0.578	0.620
DT	149	0.720	0.718	0.719	0.427	0.602	0.656
DT	150	0.665	0.707	0.691	0.363	0.573	0.616
DT	151	0.696	0.724	0.713	0.409	0.598	0.644
DT	152	0.687	0.714	0.704	0.392	0.588	0.634
DT	153	0.674	0.728	0.708	0.393	0.594	0.632
DT	154	0.683	0.743	0.721	0.418	0.611	0.645
DT	155	0.670	0.724	0.704	0.385	0.589	0.627
DT	156	0.672	0.722	0.704	0.385	0.589	0.628
DT	157	0.714	0.729	0.723	0.431	0.609	0.657
DT	158	0.672	0.730	0.708	0.394	0.596	0.631
DT	159	0.703	0.725	0.717	0.417	0.602	0.648
DT	160	0.676	0.733	0.712	0.401	0.600	0.636
DT	161	0.716	0.698	0.704	0.401	0.583	0.643
DT	162	0.659	0.718	0.696	0.369	0.581	0.617
DT	163	0.663	0.711	0.693	0.365	0.576	0.616
DT	164	0.681	0.720	0.705	0.391	0.590	0.632
DT	165	0.672	0.728	0.707	0.391	0.593	0.630
DT	166	0.672	0.731	0.709	0.395	0.597	0.632
DT	167	0.670	0.726	0.705	0.387	0.591	0.628
DT	168	0.659	0.738	0.708	0.390	0.598	0.627
DT	169	0.692	0.728	0.714	0.409	0.600	0.643
DT	170	0.654	0.737	0.706	0.384	0.595	0.623
DT	171	0.676	0.757	0.727	0.427	0.623	0.648

DT	172	0.692	0.699	0.696	0.380	0.576	0.629
DT	173	0.672	0.705	0.693	0.368	0.574	0.619
DT	174	0.683	0.705	0.697	0.378	0.578	0.626
DT	175	0.698	0.721	0.713	0.409	0.597	0.644
DT	176	0.711	0.728	0.722	0.428	0.607	0.655
DT	177	0.670	0.730	0.708	0.392	0.595	0.630
DT	178	0.700	0.747	0.730	0.438	0.621	0.658
DT	179	0.716	0.681	0.694	0.384	0.570	0.635
DT	180	0.692	0.716	0.707	0.397	0.590	0.637
DT	181	0.683	0.726	0.710	0.400	0.596	0.637
DT	182	0.659	0.724	0.699	0.374	0.585	0.620
DT	183	0.676	0.724	0.706	0.391	0.592	0.631
DT	184	0.681	0.726	0.709	0.398	0.595	0.635
DT	185	0.674	0.720	0.703	0.385	0.587	0.628
DT	186	0.678	0.714	0.701	0.383	0.584	0.628
DT	187	0.700	0.733	0.721	0.423	0.608	0.651
DT	188	0.634	0.696	0.673	0.323	0.553	0.591
DT	189	0.665	0.729	0.705	0.386	0.592	0.627
DT	190	0.707	0.734	0.724	0.431	0.611	0.656
DT	191	0.700	0.699	0.699	0.388	0.579	0.634
DT	192	0.661	0.724	0.700	0.376	0.586	0.621
DT	193	0.685	0.712	0.702	0.387	0.585	0.631
DT	194	0.672	0.729	0.708	0.392	0.595	0.631
DT	195	0.621	0.700	0.671	0.315	0.551	0.584
DT	196	0.698	0.728	0.717	0.416	0.603	0.647

DT	197	0.650	0.703	0.683	0.344	0.564	0.604
DT	198	0.678	0.722	0.706	0.392	0.591	0.632
DT	199	0.661	0.728	0.703	0.381	0.589	0.623
DT	200	0.654	0.709	0.689	0.355	0.571	0.610
DT	201	0.659	0.724	0.699	0.374	0.585	0.620
DT	202	0.672	0.722	0.704	0.385	0.589	0.628
DT	203	0.674	0.721	0.704	0.386	0.588	0.628
DT	204	0.663	0.735	0.708	0.391	0.597	0.628
DT	205	0.652	0.729	0.700	0.374	0.587	0.618
DT	206	0.652	0.726	0.699	0.371	0.585	0.617
DT	207	0.661	0.711	0.692	0.363	0.575	0.615
DT	208	0.683	0.718	0.705	0.392	0.589	0.633
DT	209	0.643	0.733	0.699	0.370	0.588	0.614
DT	210	0.681	0.726	0.709	0.398	0.595	0.635
DT	211	0.676	0.704	0.694	0.370	0.575	0.621
DT	212	0.707	0.718	0.714	0.414	0.598	0.648
DT	213	0.694	0.731	0.717	0.416	0.605	0.646
DT	214	0.698	0.705	0.703	0.393	0.584	0.636
DT	215	0.685	0.721	0.708	0.396	0.592	0.635
DT	216	0.645	0.724	0.695	0.362	0.580	0.611
DT	217	0.678	0.687	0.684	0.355	0.562	0.615
DT	218	0.698	0.728	0.717	0.416	0.603	0.647
DT	219	0.648	0.709	0.686	0.349	0.569	0.606
DT	220	0.685	0.738	0.718	0.414	0.607	0.644
DT	221	0.727	0.748	0.740	0.464	0.631	0.676

DT	222	0.672	0.748	0.720	0.413	0.612	0.641
DT	223	0.716	0.763	0.745	0.469	0.641	0.676
DT	224	0.720	0.747	0.737	0.457	0.628	0.671
DT	225	0.694	0.735	0.720	0.420	0.608	0.648
DT	226	0.683	0.743	0.721	0.418	0.611	0.645
DT	227	0.678	0.743	0.719	0.414	0.610	0.642
DT	228	0.696	0.760	0.736	0.448	0.632	0.662
DT	229	0.727	0.729	0.728	0.444	0.613	0.665
DT	230	0.652	0.743	0.709	0.389	0.600	0.625
DT	231	0.676	0.737	0.714	0.405	0.603	0.638
DT	232	0.714	0.752	0.738	0.456	0.630	0.669
DT	233	0.689	0.752	0.729	0.434	0.622	0.654
DT	234	0.683	0.741	0.719	0.415	0.609	0.644
DT	235	0.663	0.735	0.708	0.391	0.597	0.628
DT	236	0.654	0.748	0.713	0.397	0.606	0.629
DT	237	0.718	0.759	0.744	0.467	0.638	0.676
DT	238	0.705	0.754	0.735	0.449	0.629	0.665
DT	239	0.674	0.761	0.729	0.429	0.626	0.649
DT	240	0.676	0.726	0.708	0.394	0.594	0.632
DT	241	0.687	0.759	0.732	0.439	0.628	0.656
DT	242	0.700	0.744	0.728	0.436	0.619	0.657
DT	243	0.705	0.735	0.724	0.430	0.612	0.655
DT	244	0.652	0.744	0.710	0.391	0.602	0.626
DT	245	0.707	0.759	0.740	0.457	0.634	0.669
DT	246	0.661	0.754	0.719	0.409	0.613	0.636

DT	247	0.683	0.759	0.731	0.435	0.626	0.653
DT	248	0.667	0.744	0.716	0.405	0.607	0.636
DT	249	0.685	0.748	0.725	0.425	0.617	0.649
DT	250	0.678	0.725	0.708	0.394	0.593	0.633
DT	251	0.705	0.744	0.730	0.440	0.620	0.660
DT	252	0.711	0.734	0.726	0.435	0.613	0.659
DT	253	0.674	0.733	0.711	0.398	0.599	0.634
DT	254	0.703	0.769	0.744	0.464	0.643	0.672
DT	255	0.645	0.761	0.718	0.403	0.616	0.630
DT	256	0.694	0.743	0.725	0.428	0.615	0.652
DT	257	0.665	0.765	0.728	0.426	0.627	0.645
DT	258	0.672	0.738	0.713	0.402	0.603	0.635
DT	259	0.714	0.747	0.735	0.451	0.625	0.667
DT	260	0.694	0.744	0.726	0.429	0.616	0.653
DT	261	0.685	0.757	0.731	0.435	0.626	0.654
DT	262	0.711	0.739	0.729	0.440	0.618	0.661
DT	263	0.670	0.750	0.720	0.413	0.613	0.640
DT	264	0.740	0.754	0.749	0.482	0.640	0.686
DT	265	0.692	0.744	0.725	0.427	0.616	0.651
DT	266	0.672	0.735	0.712	0.399	0.600	0.634
DT	267	0.700	0.757	0.736	0.450	0.631	0.664
DT	268	0.711	0.747	0.734	0.448	0.625	0.665
DT	269	0.663	0.739	0.711	0.395	0.601	0.630
DT	270	0.692	0.731	0.717	0.413	0.604	0.645
DT	271	0.720	0.733	0.728	0.442	0.615	0.663

DT	272	0.700	0.731	0.720	0.422	0.607	0.650
DT	273	0.727	0.737	0.733	0.452	0.620	0.669
DT	274	0.694	0.747	0.727	0.432	0.619	0.654
DT	275	0.703	0.755	0.735	0.449	0.629	0.664
DT	276	0.692	0.769	0.740	0.454	0.640	0.665
DT	277	0.700	0.767	0.742	0.459	0.640	0.669
DT	278	0.720	0.738	0.731	0.447	0.619	0.666
DT	279	0.692	0.737	0.720	0.419	0.609	0.647
DT	280	0.698	0.739	0.724	0.428	0.613	0.653
DT	281	0.727	0.752	0.743	0.468	0.635	0.678
DT	282	0.738	0.725	0.730	0.450	0.614	0.670
DT	283	0.676	0.751	0.723	0.420	0.616	0.645
DT	284	0.707	0.725	0.718	0.421	0.603	0.651
DT	285	0.678	0.755	0.726	0.426	0.621	0.648
DT	286	0.714	0.741	0.731	0.444	0.620	0.663
DT	287	0.714	0.756	0.740	0.460	0.634	0.672
DT	288	0.718	0.751	0.739	0.459	0.631	0.671
DT	289	0.681	0.755	0.727	0.428	0.622	0.650
DT	290	0.678	0.728	0.709	0.397	0.596	0.634
DT	291	0.707	0.752	0.735	0.450	0.628	0.665
DT	292	0.716	0.759	0.743	0.465	0.637	0.674
DT	293	0.707	0.771	0.747	0.470	0.646	0.675
DT	294	0.696	0.759	0.735	0.447	0.631	0.662
DT	295	0.687	0.729	0.713	0.407	0.600	0.641
DT	296	0.678	0.730	0.711	0.400	0.598	0.636

DT	297	0.643	0.735	0.701	0.373	0.590	0.615
DT	298	0.696	0.757	0.735	0.445	0.629	0.661
DT	299	0.716	0.739	0.731	0.444	0.619	0.664
DT	300	0.641	0.759	0.715	0.396	0.611	0.626
DT	301	0.683	0.750	0.725	0.425	0.618	0.649
DT	302	0.692	0.739	0.722	0.422	0.611	0.649
DT	303	0.698	0.755	0.734	0.445	0.628	0.661
DT	304	0.742	0.759	0.753	0.490	0.646	0.691
DT	305	0.687	0.737	0.718	0.415	0.607	0.645
DT	306	0.683	0.743	0.721	0.418	0.611	0.645
DT	307	0.676	0.730	0.710	0.398	0.597	0.634
DT	308	0.689	0.748	0.726	0.430	0.619	0.652
DT	309	0.703	0.704	0.704	0.395	0.584	0.638
DT	310	0.648	0.748	0.711	0.391	0.604	0.625
DT	311	0.705	0.741	0.727	0.435	0.617	0.658
DT	312	0.687	0.735	0.717	0.414	0.606	0.644
DT	313	0.707	0.748	0.733	0.446	0.625	0.663
DT	314	0.705	0.752	0.735	0.448	0.627	0.664
DT	315	0.694	0.722	0.712	0.406	0.597	0.642
DT	316	0.678	0.742	0.718	0.412	0.609	0.642
DT	317	0.676	0.718	0.703	0.385	0.587	0.628
DT	318	0.736	0.728	0.731	0.450	0.615	0.670
DT	319	0.683	0.733	0.714	0.407	0.602	0.640
DT	320	0.694	0.726	0.714	0.410	0.600	0.644
DT	321	0.683	0.737	0.717	0.411	0.605	0.642

DT	322	0.711	0.726	0.721	0.426	0.606	0.655
DT	323	0.694	0.755	0.732	0.441	0.626	0.658
DT	324	0.742	0.728	0.733	0.457	0.617	0.674
DT	325	0.707	0.733	0.723	0.429	0.610	0.655
DT	326	0.689	0.733	0.717	0.413	0.604	0.644
DT	327	0.689	0.739	0.721	0.420	0.610	0.647
DT	328	0.694	0.744	0.726	0.429	0.616	0.653
DT	329	0.678	0.743	0.719	0.414	0.610	0.642
DT	330	0.703	0.721	0.714	0.413	0.598	0.646
DT	331	0.696	0.739	0.723	0.426	0.612	0.652
DT	332	0.689	0.739	0.721	0.420	0.610	0.647
DT	333	0.711	0.741	0.730	0.442	0.619	0.662
DT	334	0.729	0.735	0.733	0.452	0.620	0.670
DT	335	0.736	0.734	0.735	0.457	0.621	0.673
DT	336	0.711	0.733	0.725	0.433	0.612	0.658
DT	337	0.694	0.773	0.744	0.461	0.644	0.668
DT	338	0.720	0.733	0.728	0.442	0.615	0.663
DT	339	0.652	0.738	0.706	0.384	0.596	0.623
DT	340	0.711	0.748	0.735	0.450	0.626	0.666
DT	341	0.703	0.726	0.717	0.418	0.603	0.649
DT	342	0.692	0.768	0.740	0.453	0.638	0.664
DT	343	0.709	0.729	0.722	0.427	0.608	0.654
DT	344	0.694	0.742	0.724	0.427	0.614	0.651
DT	345	0.749	0.765	0.759	0.503	0.654	0.698
DT	346	0.678	0.765	0.733	0.438	0.631	0.654

DT	347	0.698	0.743	0.726	0.432	0.617	0.655
DT	348	0.698	0.748	0.730	0.438	0.622	0.658
DT	349	0.714	0.739	0.730	0.442	0.618	0.663
DT	350	0.716	0.759	0.743	0.465	0.637	0.674
DT	351	0.720	0.760	0.745	0.471	0.640	0.678
DT	352	0.725	0.757	0.745	0.472	0.639	0.679
DT	353	0.729	0.781	0.762	0.501	0.663	0.695
DT	354	0.703	0.743	0.728	0.436	0.618	0.658
DT	355	0.714	0.774	0.752	0.480	0.652	0.681
DT	356	0.722	0.751	0.740	0.463	0.632	0.674
DT	357	0.709	0.741	0.729	0.440	0.618	0.661
DT	358	0.729	0.746	0.740	0.463	0.629	0.676
DT	359	0.742	0.757	0.752	0.488	0.644	0.690
DT	360	0.736	0.755	0.748	0.479	0.640	0.684
DT	361	0.720	0.748	0.738	0.458	0.629	0.671
DT	362	0.722	0.772	0.753	0.485	0.652	0.685
DT	363	0.725	0.798	0.771	0.516	0.680	0.701
DT	364	0.676	0.769	0.735	0.440	0.634	0.655
DT	365	0.747	0.774	0.764	0.510	0.662	0.702
DT	366	0.722	0.767	0.750	0.480	0.647	0.683
DT	367	0.740	0.787	0.770	0.519	0.673	0.705
DT	368	0.736	0.791	0.771	0.519	0.676	0.705
DT	369	0.747	0.780	0.767	0.516	0.667	0.705
DT	370	0.769	0.808	0.794	0.568	0.704	0.735
KNN	1	0.489	0.593	0.554	0.080	0.416	0.449

KNN	2	0.634	0.622	0.627	0.248	0.498	0.558
KNN	3	0.672	0.619	0.639	0.281	0.511	0.580
KNN	4	0.707	0.634	0.661	0.329	0.533	0.608
KNN	5	0.709	0.626	0.657	0.324	0.529	0.606
KNN	6	0.720	0.618	0.656	0.327	0.527	0.609
KNN	7	0.700	0.653	0.671	0.342	0.545	0.613
KNN	8	0.727	0.639	0.672	0.354	0.544	0.622
KNN	9	0.729	0.635	0.670	0.352	0.542	0.622
KNN	10	0.744	0.668	0.696	0.398	0.570	0.646
KNN	11	0.744	0.666	0.695	0.397	0.569	0.645
KNN	12	0.749	0.683	0.708	0.418	0.583	0.656
KNN	13	0.747	0.699	0.717	0.432	0.595	0.662
KNN	14	0.767	0.683	0.714	0.435	0.589	0.666
KNN	15	0.767	0.684	0.715	0.436	0.590	0.667
KNN	16	0.767	0.679	0.712	0.431	0.586	0.664
KNN	17	0.756	0.653	0.691	0.395	0.563	0.645
KNN	18	0.771	0.652	0.696	0.409	0.567	0.654
KNN	19	0.767	0.668	0.704	0.420	0.577	0.658
KNN	20	0.753	0.656	0.692	0.395	0.564	0.645
KNN	21	0.782	0.679	0.717	0.446	0.591	0.673
KNN	22	0.778	0.682	0.717	0.444	0.591	0.672
KNN	23	0.762	0.674	0.707	0.422	0.581	0.659
KNN	24	0.804	0.673	0.722	0.461	0.593	0.682
KNN	25	0.764	0.665	0.702	0.415	0.575	0.656
KNN	26	0.784	0.657	0.704	0.427	0.575	0.664

KNN	27	0.811	0.671	0.723	0.466	0.594	0.685
KNN	28	0.795	0.688	0.728	0.467	0.602	0.685
KNN	29	0.780	0.658	0.704	0.424	0.575	0.662
KNN	30	0.780	0.677	0.715	0.441	0.588	0.670
KNN	31	0.815	0.674	0.726	0.473	0.597	0.689
KNN	32	0.791	0.668	0.713	0.443	0.585	0.672
KNN	33	0.797	0.677	0.722	0.458	0.593	0.680
KNN	34	0.756	0.670	0.702	0.412	0.576	0.653
KNN	35	0.782	0.661	0.706	0.428	0.577	0.664
KNN	36	0.808	0.649	0.708	0.443	0.577	0.673
KNN	37	0.769	0.669	0.706	0.423	0.579	0.660
KNN	38	0.789	0.656	0.705	0.430	0.576	0.665
KNN	39	0.771	0.657	0.699	0.414	0.571	0.656
KNN	40	0.800	0.653	0.708	0.438	0.577	0.670
KNN	41	0.784	0.658	0.705	0.428	0.576	0.664
KNN	42	0.780	0.662	0.706	0.427	0.577	0.664
KNN	43	0.815	0.653	0.713	0.453	0.582	0.679
KNN	44	0.758	0.605	0.662	0.351	0.532	0.625
KNN	45	0.764	0.609	0.667	0.361	0.536	0.630
KNN	46	0.756	0.604	0.660	0.348	0.530	0.623
KNN	47	0.773	0.606	0.668	0.368	0.538	0.634
KNN	48	0.789	0.606	0.674	0.383	0.542	0.643
KNN	49	0.789	0.592	0.665	0.370	0.534	0.636
KNN	50	0.769	0.614	0.672	0.371	0.541	0.635
KNN	51	0.773	0.610	0.671	0.371	0.540	0.636

KNN	52	0.749	0.617	0.666	0.354	0.536	0.625
KNN	53	0.758	0.627	0.676	0.372	0.546	0.635
KNN	54	0.762	0.608	0.665	0.358	0.535	0.629
KNN	55	0.767	0.619	0.674	0.373	0.544	0.636
KNN	56	0.767	0.652	0.695	0.404	0.566	0.651
KNN	57	0.797	0.622	0.687	0.406	0.555	0.655
KNN	58	0.786	0.648	0.699	0.420	0.569	0.661
KNN	59	0.813	0.635	0.701	0.434	0.569	0.669
KNN	60	0.804	0.645	0.704	0.435	0.573	0.669
KNN	61	0.813	0.634	0.700	0.432	0.568	0.668
KNN	62	0.824	0.644	0.711	0.453	0.578	0.679
KNN	63	0.806	0.635	0.699	0.427	0.567	0.665
KNN	64	0.793	0.643	0.699	0.421	0.568	0.662
KNN	65	0.793	0.632	0.692	0.412	0.561	0.657
KNN	66	0.815	0.652	0.713	0.452	0.581	0.678
KNN	67	0.784	0.658	0.705	0.428	0.576	0.664
KNN	68	0.786	0.644	0.697	0.416	0.567	0.659
KNN	69	0.813	0.649	0.710	0.447	0.578	0.676
KNN	70	0.789	0.651	0.702	0.425	0.572	0.663
KNN	71	0.782	0.647	0.697	0.414	0.567	0.657
KNN	72	0.791	0.649	0.702	0.426	0.572	0.664
KNN	73	0.813	0.645	0.708	0.443	0.576	0.674
KNN	74	0.786	0.648	0.699	0.420	0.569	0.661
KNN	75	0.835	0.639	0.712	0.459	0.578	0.683
KNN	76	0.806	0.644	0.704	0.436	0.573	0.670

KNN	77	0.804	0.651	0.708	0.440	0.577	0.672
KNN	78	0.813	0.656	0.714	0.453	0.583	0.679
KNN	79	0.795	0.654	0.707	0.435	0.577	0.669
KNN	80	0.828	0.653	0.718	0.466	0.586	0.686
KNN	81	0.806	0.645	0.705	0.437	0.574	0.670
KNN	82	0.802	0.645	0.704	0.433	0.572	0.668
KNN	83	0.808	0.643	0.704	0.437	0.573	0.670
KNN	84	0.815	0.658	0.717	0.458	0.585	0.681
KNN	85	0.784	0.652	0.701	0.422	0.571	0.661
KNN	86	0.824	0.654	0.717	0.463	0.585	0.684
KNN	87	0.811	0.652	0.711	0.447	0.580	0.676
KNN	88	0.808	0.636	0.700	0.430	0.568	0.667
KNN	89	0.789	0.649	0.701	0.423	0.571	0.662
KNN	90	0.802	0.638	0.699	0.425	0.567	0.664
KNN	91	0.824	0.649	0.714	0.458	0.582	0.682
KNN	92	0.797	0.647	0.703	0.429	0.572	0.666
KNN	93	0.800	0.636	0.697	0.422	0.565	0.662
KNN	94	0.811	0.639	0.703	0.435	0.571	0.670
KNN	95	0.802	0.648	0.705	0.435	0.574	0.669
KNN	96	0.815	0.652	0.713	0.452	0.581	0.678
KNN	97	0.804	0.638	0.699	0.427	0.568	0.665
KNN	98	0.811	0.658	0.715	0.454	0.584	0.679
KNN	99	0.839	0.625	0.704	0.450	0.570	0.679
KNN	100	0.786	0.649	0.700	0.421	0.570	0.661
KNN	101	0.808	0.635	0.699	0.429	0.567	0.667

KNN	102	0.844	0.645	0.719	0.474	0.585	0.691
KNN	103	0.778	0.641	0.692	0.405	0.562	0.652
KNN	104	0.806	0.639	0.701	0.431	0.569	0.667
KNN	105	0.833	0.647	0.716	0.464	0.582	0.685
KNN	106	0.806	0.641	0.703	0.433	0.571	0.668
KNN	107	0.808	0.639	0.702	0.433	0.570	0.668
KNN	108	0.830	0.640	0.711	0.456	0.577	0.681
KNN	109	0.822	0.614	0.691	0.423	0.558	0.664
KNN	110	0.815	0.638	0.704	0.438	0.571	0.672
KNN	111	0.817	0.635	0.703	0.438	0.570	0.671
KNN	112	0.780	0.652	0.699	0.417	0.570	0.659
KNN	113	0.793	0.639	0.696	0.418	0.565	0.660
KNN	114	0.786	0.645	0.698	0.417	0.568	0.659
KNN	115	0.830	0.630	0.704	0.446	0.570	0.676
KNN	116	0.808	0.652	0.710	0.445	0.579	0.675
KNN	117	0.830	0.632	0.706	0.449	0.572	0.677
KNN	118	0.819	0.648	0.712	0.452	0.579	0.679
KNN	119	0.808	0.649	0.708	0.443	0.577	0.673
KNN	120	0.859	0.639	0.721	0.483	0.585	0.696
KNN	121	0.824	0.644	0.711	0.453	0.578	0.679
KNN	122	0.839	0.660	0.726	0.483	0.593	0.695
KNN	123	0.824	0.657	0.719	0.465	0.587	0.686
KNN	124	0.824	0.649	0.714	0.458	0.582	0.682
KNN	125	0.819	0.658	0.718	0.462	0.587	0.684
KNN	126	0.839	0.660	0.726	0.483	0.593	0.695

KNN	127	0.822	0.662	0.722	0.468	0.590	0.687
KNN	128	0.837	0.653	0.722	0.475	0.588	0.691
KNN	129	0.819	0.651	0.713	0.455	0.581	0.680
KNN	130	0.822	0.654	0.717	0.461	0.585	0.683
KNN	131	0.819	0.657	0.717	0.461	0.586	0.683
KNN	132	0.833	0.652	0.719	0.469	0.586	0.688
KNN	133	0.837	0.640	0.713	0.463	0.579	0.685
KNN	134	0.782	0.653	0.701	0.421	0.572	0.660
KNN	135	0.813	0.658	0.716	0.456	0.585	0.680
KNN	136	0.804	0.653	0.709	0.442	0.578	0.673
KNN	137	0.835	0.661	0.726	0.480	0.593	0.694
KNN	138	0.835	0.647	0.717	0.466	0.583	0.687
KNN	139	0.826	0.668	0.726	0.477	0.595	0.692
KNN	140	0.855	0.660	0.732	0.498	0.598	0.704
KNN	141	0.800	0.686	0.728	0.469	0.601	0.686
KNN	142	0.822	0.669	0.726	0.474	0.595	0.690
KNN	143	0.855	0.675	0.742	0.513	0.609	0.711
KNN	144	0.828	0.666	0.726	0.478	0.595	0.692
KNN	145	0.819	0.684	0.735	0.487	0.606	0.697
KNN	146	0.800	0.691	0.731	0.474	0.605	0.689
KNN	147	0.811	0.690	0.735	0.484	0.607	0.694
KNN	148	0.815	0.678	0.729	0.476	0.600	0.691
KNN	149	0.795	0.691	0.730	0.470	0.604	0.686
KNN	150	0.813	0.684	0.732	0.481	0.604	0.693
KNN	151	0.830	0.682	0.737	0.495	0.607	0.701

KNN	152	0.815	0.688	0.735	0.487	0.608	0.696
KNN	153	0.797	0.696	0.734	0.477	0.608	0.690
KNN	154	0.835	0.688	0.743	0.506	0.613	0.707
KNN	155	0.833	0.682	0.738	0.497	0.608	0.703
KNN	156	0.808	0.675	0.725	0.468	0.596	0.686
KNN	157	0.822	0.688	0.738	0.493	0.609	0.700
KNN	158	0.828	0.688	0.740	0.499	0.611	0.703
KNN	159	0.813	0.691	0.736	0.487	0.609	0.696
KNN	160	0.846	0.698	0.753	0.525	0.623	0.718
KNN	161	0.804	0.683	0.728	0.471	0.600	0.687
KNN	162	0.830	0.688	0.741	0.501	0.612	0.705
KNN	163	0.828	0.696	0.745	0.507	0.617	0.707
KNN	164	0.806	0.683	0.729	0.473	0.601	0.689
KNN	165	0.817	0.700	0.744	0.500	0.617	0.703
KNN	166	0.797	0.703	0.738	0.484	0.614	0.693
KNN	167	0.808	0.687	0.732	0.479	0.605	0.692
KNN	168	0.786	0.694	0.728	0.464	0.603	0.683
KNN	169	0.826	0.700	0.747	0.509	0.620	0.708
KNN	170	0.817	0.691	0.738	0.491	0.610	0.699
KNN	171	0.817	0.698	0.742	0.498	0.615	0.702
KNN	172	0.789	0.699	0.732	0.471	0.608	0.686
KNN	173	0.791	0.699	0.733	0.473	0.608	0.688
KNN	174	0.784	0.696	0.729	0.465	0.604	0.683
KNN	175	0.824	0.696	0.744	0.503	0.616	0.705
KNN	176	0.782	0.701	0.731	0.468	0.608	0.684

KNN	177	0.815	0.694	0.739	0.492	0.612	0.699
KNN	178	0.824	0.662	0.722	0.470	0.591	0.688
KNN	179	0.808	0.679	0.727	0.471	0.599	0.688
KNN	180	0.846	0.682	0.743	0.510	0.611	0.710
KNN	181	0.819	0.669	0.725	0.472	0.594	0.689
KNN	182	0.824	0.665	0.724	0.473	0.593	0.689
KNN	183	0.824	0.688	0.739	0.495	0.610	0.701
KNN	184	0.841	0.681	0.740	0.505	0.609	0.707
KNN	185	0.850	0.660	0.731	0.494	0.597	0.701
KNN	186	0.780	0.679	0.717	0.444	0.590	0.672
KNN	187	0.778	0.673	0.712	0.435	0.584	0.667
KNN	188	0.806	0.679	0.726	0.469	0.598	0.687
KNN	189	0.775	0.694	0.724	0.454	0.600	0.676
KNN	190	0.822	0.678	0.731	0.483	0.602	0.695
KNN	191	0.839	0.678	0.738	0.500	0.607	0.704
KNN	192	0.782	0.691	0.725	0.457	0.600	0.679
KNN	193	0.789	0.679	0.720	0.452	0.593	0.677
KNN	194	0.800	0.683	0.726	0.467	0.599	0.685
KNN	195	0.780	0.691	0.724	0.455	0.599	0.678
KNN	196	0.802	0.688	0.731	0.474	0.604	0.689
KNN	197	0.797	0.670	0.717	0.452	0.589	0.677
KNN	198	0.800	0.677	0.722	0.460	0.594	0.682
KNN	199	0.769	0.674	0.709	0.428	0.583	0.663
KNN	200	0.806	0.678	0.726	0.468	0.597	0.686
KNN	201	0.795	0.684	0.726	0.464	0.599	0.683

KNN	202	0.800	0.674	0.721	0.458	0.592	0.680
KNN	203	0.819	0.677	0.730	0.480	0.600	0.693
KNN	204	0.808	0.678	0.726	0.470	0.598	0.687
KNN	205	0.760	0.691	0.717	0.436	0.593	0.666
KNN	206	0.791	0.690	0.727	0.465	0.601	0.683
KNN	207	0.795	0.687	0.727	0.466	0.601	0.684
KNN	208	0.773	0.692	0.722	0.450	0.598	0.674
KNN	209	0.797	0.698	0.735	0.479	0.609	0.691
KNN	210	0.806	0.687	0.731	0.477	0.604	0.691
KNN	211	0.800	0.668	0.717	0.452	0.587	0.677
KNN	212	0.771	0.682	0.715	0.438	0.589	0.668
KNN	213	0.786	0.675	0.717	0.446	0.589	0.674
KNN	214	0.780	0.694	0.726	0.458	0.601	0.679
KNN	215	0.784	0.678	0.717	0.447	0.590	0.674
KNN	216	0.767	0.698	0.723	0.449	0.600	0.673
KNN	217	0.822	0.695	0.742	0.499	0.614	0.703
KNN	218	0.764	0.692	0.719	0.442	0.595	0.669
KNN	219	0.795	0.684	0.726	0.464	0.599	0.683
KNN	220	0.791	0.688	0.726	0.463	0.600	0.683
KNN	221	0.791	0.696	0.731	0.471	0.606	0.686
KNN	222	0.786	0.696	0.730	0.467	0.605	0.684
KNN	223	0.775	0.694	0.724	0.454	0.600	0.676
KNN	224	0.778	0.696	0.726	0.458	0.602	0.679
KNN	225	0.795	0.687	0.727	0.466	0.601	0.684
KNN	226	0.786	0.670	0.713	0.441	0.585	0.671

KNN	227	0.773	0.688	0.720	0.446	0.595	0.672
KNN	228	0.769	0.696	0.723	0.450	0.600	0.674
KNN	229	0.786	0.699	0.731	0.469	0.607	0.685
KNN	230	0.789	0.703	0.735	0.475	0.611	0.688
KNN	231	0.791	0.695	0.731	0.470	0.605	0.686
KNN	232	0.784	0.679	0.718	0.448	0.591	0.674
KNN	233	0.800	0.687	0.729	0.470	0.602	0.687
KNN	234	0.780	0.700	0.730	0.464	0.606	0.682
KNN	235	0.780	0.682	0.718	0.446	0.592	0.673
KNN	236	0.791	0.690	0.727	0.465	0.601	0.683
KNN	237	0.795	0.694	0.731	0.473	0.606	0.688
KNN	238	0.782	0.684	0.721	0.451	0.595	0.676
KNN	239	0.778	0.654	0.700	0.418	0.571	0.659
KNN	240	0.769	0.671	0.708	0.426	0.581	0.662
KNN	241	0.802	0.674	0.722	0.460	0.593	0.682
KNN	242	0.769	0.664	0.703	0.418	0.575	0.658
KNN	243	0.764	0.658	0.698	0.409	0.570	0.653
KNN	244	0.749	0.666	0.697	0.401	0.570	0.648
KNN	245	0.749	0.670	0.699	0.405	0.573	0.649
KNN	246	0.771	0.662	0.703	0.419	0.575	0.659
KNN	247	0.789	0.660	0.708	0.433	0.578	0.667
KNN	248	0.778	0.669	0.709	0.431	0.582	0.665
KNN	249	0.778	0.661	0.704	0.424	0.576	0.662
KNN	250	0.758	0.669	0.702	0.412	0.575	0.654
KNN	251	0.791	0.671	0.716	0.447	0.588	0.674

KNN	252	0.800	0.668	0.717	0.452	0.587	0.677
KNN	253	0.791	0.668	0.713	0.443	0.585	0.672
KNN	254	0.784	0.656	0.704	0.425	0.574	0.663
KNN	255	0.786	0.665	0.710	0.436	0.581	0.669
KNN	256	0.775	0.658	0.702	0.419	0.573	0.659
KNN	257	0.791	0.662	0.710	0.438	0.581	0.670
KNN	258	0.793	0.648	0.702	0.426	0.571	0.664
KNN	259	0.744	0.660	0.691	0.391	0.564	0.642
KNN	260	0.773	0.653	0.698	0.412	0.569	0.655
KNN	261	0.767	0.657	0.698	0.409	0.570	0.654
KNN	262	0.804	0.660	0.713	0.448	0.583	0.676
KNN	263	0.791	0.654	0.705	0.430	0.575	0.666
KNN	264	0.789	0.658	0.707	0.432	0.577	0.667
KNN	265	0.762	0.654	0.695	0.403	0.566	0.650
KNN	266	0.778	0.654	0.700	0.418	0.571	0.659
KNN	267	0.762	0.665	0.701	0.413	0.574	0.655
KNN	268	0.795	0.652	0.705	0.432	0.575	0.667
KNN	269	0.780	0.664	0.707	0.429	0.578	0.664
KNN	270	0.789	0.651	0.702	0.425	0.572	0.663
KNN	271	0.773	0.652	0.697	0.411	0.568	0.655
KNN	272	0.806	0.656	0.712	0.447	0.581	0.675
KNN	273	0.784	0.660	0.706	0.429	0.577	0.665
KNN	274	0.784	0.653	0.702	0.423	0.572	0.662
KNN	275	0.793	0.645	0.700	0.424	0.570	0.663
KNN	276	0.795	0.651	0.704	0.431	0.574	0.667

KNN	277	0.778	0.649	0.697	0.413	0.568	0.656
KNN	278	0.760	0.657	0.695	0.403	0.567	0.650
KNN	279	0.771	0.649	0.695	0.406	0.565	0.652
KNN	280	0.797	0.669	0.717	0.451	0.588	0.677
KNN	281	0.797	0.681	0.724	0.462	0.596	0.682
KNN	282	0.795	0.661	0.711	0.441	0.581	0.672
KNN	283	0.767	0.682	0.713	0.434	0.588	0.665
KNN	284	0.789	0.681	0.721	0.453	0.594	0.677
KNN	285	0.800	0.671	0.719	0.455	0.590	0.679
KNN	286	0.782	0.678	0.717	0.445	0.590	0.672
KNN	287	0.822	0.682	0.734	0.487	0.605	0.697
KNN	288	0.808	0.691	0.735	0.483	0.608	0.694
KNN	289	0.758	0.677	0.707	0.420	0.581	0.658
KNN	290	0.771	0.696	0.724	0.452	0.600	0.675
KNN	291	0.824	0.671	0.728	0.479	0.597	0.693
KNN	292	0.797	0.681	0.724	0.462	0.596	0.682
KNN	293	0.782	0.681	0.718	0.447	0.592	0.674
KNN	294	0.789	0.684	0.723	0.457	0.597	0.679
KNN	295	0.762	0.673	0.706	0.420	0.580	0.658
KNN	296	0.817	0.678	0.730	0.479	0.600	0.692
KNN	297	0.806	0.674	0.723	0.464	0.594	0.684
KNN	298	0.769	0.675	0.710	0.429	0.584	0.663
KNN	299	0.786	0.678	0.718	0.449	0.591	0.675
KNN	300	0.771	0.684	0.717	0.440	0.591	0.669
KNN	301	0.784	0.675	0.716	0.444	0.588	0.672

KNN	302	0.797	0.692	0.731	0.473	0.605	0.688
KNN	303	0.769	0.691	0.720	0.445	0.596	0.671
KNN	304	0.804	0.669	0.719	0.457	0.590	0.680
KNN	305	0.789	0.682	0.722	0.455	0.595	0.678
KNN	306	0.782	0.681	0.718	0.447	0.592	0.674
KNN	307	0.784	0.671	0.713	0.440	0.586	0.670
KNN	308	0.793	0.682	0.723	0.459	0.596	0.681
KNN	309	0.780	0.684	0.720	0.449	0.594	0.674
KNN	310	0.806	0.671	0.722	0.462	0.592	0.683
KNN	311	0.793	0.671	0.717	0.449	0.588	0.675
KNN	312	0.800	0.671	0.719	0.455	0.590	0.679
KNN	313	0.808	0.683	0.730	0.475	0.602	0.690
KNN	314	0.797	0.670	0.717	0.452	0.589	0.677
KNN	315	0.791	0.687	0.726	0.462	0.599	0.682
KNN	316	0.815	0.677	0.728	0.475	0.599	0.690
KNN	317	0.782	0.678	0.717	0.445	0.590	0.672
KNN	318	0.791	0.666	0.713	0.442	0.584	0.672
KNN	319	0.802	0.670	0.719	0.456	0.590	0.680
KNN	320	0.760	0.679	0.709	0.425	0.584	0.660
KNN	321	0.786	0.678	0.718	0.449	0.591	0.675
KNN	322	0.784	0.683	0.721	0.452	0.594	0.676
KNN	323	0.773	0.677	0.713	0.435	0.586	0.667
KNN	324	0.802	0.683	0.727	0.469	0.600	0.686
KNN	325	0.778	0.675	0.713	0.438	0.586	0.669
KNN	326	0.791	0.669	0.714	0.444	0.586	0.673

KNN	327	0.780	0.682	0.718	0.446	0.592	0.673
KNN	328	0.806	0.678	0.726	0.468	0.597	0.686
KNN	329	0.778	0.677	0.714	0.439	0.587	0.669
KNN	330	0.791	0.674	0.717	0.449	0.589	0.675
KNN	331	0.782	0.683	0.720	0.450	0.594	0.675
KNN	332	0.784	0.681	0.719	0.449	0.592	0.675
KNN	333	0.769	0.674	0.709	0.428	0.583	0.663
KNN	334	0.791	0.700	0.734	0.475	0.610	0.688
KNN	335	0.815	0.656	0.715	0.455	0.584	0.680
KNN	336	0.800	0.670	0.718	0.454	0.589	0.679
KNN	337	0.808	0.675	0.725	0.468	0.596	0.686
KNN	338	0.760	0.678	0.708	0.423	0.583	0.660
KNN	339	0.780	0.671	0.712	0.436	0.584	0.668
KNN	340	0.800	0.660	0.712	0.444	0.582	0.673
KNN	341	0.817	0.673	0.726	0.474	0.596	0.690
KNN	342	0.797	0.674	0.720	0.456	0.592	0.679
KNN	343	0.822	0.678	0.731	0.483	0.602	0.695
KNN	344	0.802	0.684	0.728	0.470	0.601	0.687
KNN	345	0.804	0.679	0.726	0.467	0.597	0.685
KNN	346	0.775	0.665	0.706	0.426	0.578	0.662
KNN	347	0.793	0.694	0.731	0.470	0.605	0.686
KNN	348	0.778	0.688	0.722	0.451	0.596	0.675
KNN	349	0.791	0.670	0.715	0.445	0.587	0.674
KNN	350	0.793	0.671	0.717	0.449	0.588	0.675
KNN	351	0.782	0.673	0.713	0.440	0.586	0.670

KNN	352	0.815	0.666	0.722	0.465	0.591	0.685
KNN	353	0.822	0.679	0.732	0.484	0.603	0.695
KNN	354	0.797	0.691	0.731	0.472	0.604	0.688
KNN	355	0.769	0.679	0.713	0.433	0.587	0.665
KNN	356	0.791	0.686	0.725	0.461	0.598	0.681
KNN	357	0.778	0.690	0.722	0.452	0.597	0.676
KNN	358	0.806	0.679	0.726	0.469	0.598	0.687
KNN	359	0.800	0.677	0.722	0.460	0.594	0.682
KNN	360	0.767	0.675	0.709	0.427	0.583	0.662
KNN	361	0.800	0.684	0.727	0.468	0.600	0.686
KNN	362	0.804	0.696	0.736	0.484	0.610	0.694
KNN	363	0.786	0.695	0.729	0.465	0.604	0.683
KNN	364	0.786	0.682	0.721	0.453	0.594	0.677
KNN	365	0.771	0.699	0.726	0.455	0.602	0.676
KNN	366	0.773	0.683	0.717	0.441	0.591	0.670
KNN	367	0.769	0.682	0.714	0.436	0.589	0.667
KNN	368	0.773	0.695	0.724	0.453	0.600	0.676
KNN	369	0.775	0.692	0.723	0.452	0.599	0.676
KNN	370	0.780	0.700	0.730	0.464	0.606	0.682
RF	1	0.621	0.536	0.568	0.152	0.442	0.516
RF	2	0.599	0.656	0.635	0.248	0.507	0.549
RF	3	0.615	0.653	0.639	0.260	0.512	0.559
RF	4	0.619	0.628	0.625	0.240	0.496	0.551
RF	5	0.689	0.653	0.667	0.332	0.541	0.606
RF	6	0.652	0.640	0.645	0.283	0.517	0.577

RF	7	0.650	0.671	0.663	0.312	0.539	0.589
RF	8	0.623	0.698	0.670	0.314	0.550	0.584
RF	9	0.661	0.714	0.695	0.367	0.578	0.617
RF	10	0.694	0.704	0.700	0.387	0.581	0.633
RF	11	0.694	0.711	0.704	0.394	0.587	0.636
RF	12	0.703	0.720	0.713	0.411	0.597	0.646
RF	13	0.698	0.731	0.719	0.420	0.606	0.649
RF	14	0.716	0.731	0.726	0.436	0.612	0.660
RF	15	0.703	0.738	0.725	0.431	0.613	0.655
RF	16	0.722	0.751	0.740	0.463	0.632	0.674
RF	17	0.714	0.750	0.736	0.453	0.628	0.668
RF	18	0.696	0.752	0.731	0.440	0.625	0.658
RF	19	0.692	0.726	0.713	0.408	0.599	0.642
RF	20	0.696	0.754	0.732	0.441	0.626	0.659
RF	21	0.689	0.747	0.726	0.428	0.617	0.651
RF	22	0.696	0.777	0.747	0.467	0.649	0.672
RF	23	0.685	0.768	0.737	0.447	0.636	0.660
RF	24	0.698	0.769	0.743	0.460	0.642	0.669
RF	25	0.698	0.761	0.738	0.452	0.634	0.665
RF	26	0.725	0.773	0.755	0.489	0.654	0.688
RF	27	0.727	0.787	0.765	0.507	0.669	0.697
RF	28	0.733	0.774	0.759	0.498	0.658	0.694
RF	29	0.740	0.773	0.761	0.503	0.659	0.697
RF	30	0.747	0.791	0.775	0.529	0.679	0.711
RF	31	0.742	0.787	0.771	0.521	0.674	0.706

RF	32	0.736	0.782	0.765	0.509	0.667	0.699
RF	33	0.749	0.784	0.771	0.522	0.672	0.708
RF	34	0.747	0.777	0.766	0.513	0.665	0.703
RF	35	0.744	0.778	0.766	0.513	0.665	0.703
RF	36	0.731	0.769	0.755	0.491	0.652	0.690
RF	37	0.744	0.778	0.766	0.513	0.665	0.703
RF	38	0.749	0.784	0.771	0.522	0.672	0.708
RF	39	0.762	0.795	0.783	0.547	0.688	0.723
RF	40	0.740	0.812	0.785	0.547	0.700	0.719
RF	41	0.764	0.810	0.793	0.565	0.704	0.733
RF	42	0.778	0.803	0.794	0.570	0.700	0.737
RF	43	0.764	0.816	0.797	0.573	0.711	0.737
RF	44	0.771	0.793	0.785	0.552	0.688	0.727
RF	45	0.760	0.794	0.781	0.544	0.686	0.721
RF	46	0.758	0.799	0.784	0.548	0.691	0.723
RF	47	0.756	0.801	0.784	0.547	0.692	0.722
RF	48	0.767	0.798	0.786	0.554	0.692	0.727
RF	49	0.762	0.804	0.789	0.558	0.698	0.728
RF	50	0.760	0.804	0.788	0.556	0.697	0.727
RF	51	0.775	0.801	0.791	0.565	0.697	0.734
RF	52	0.775	0.803	0.793	0.568	0.700	0.736
RF	53	0.771	0.814	0.798	0.576	0.710	0.739
RF	54	0.786	0.820	0.808	0.597	0.721	0.752
RF	55	0.747	0.806	0.784	0.545	0.695	0.720
RF	56	0.789	0.815	0.805	0.593	0.716	0.751

RF	57	0.775	0.824	0.806	0.592	0.723	0.748
RF	58	0.778	0.817	0.803	0.586	0.716	0.746
RF	59	0.767	0.820	0.800	0.579	0.716	0.740
RF	60	0.784	0.816	0.804	0.591	0.716	0.749
RF	61	0.764	0.824	0.802	0.582	0.720	0.741
RF	62	0.771	0.811	0.796	0.573	0.707	0.738
RF	63	0.769	0.819	0.800	0.580	0.715	0.741
RF	64	0.762	0.821	0.799	0.577	0.716	0.739
RF	65	0.778	0.821	0.805	0.591	0.720	0.748
RF	66	0.775	0.823	0.805	0.590	0.721	0.747
RF	67	0.771	0.824	0.804	0.588	0.722	0.745
RF	68	0.756	0.823	0.798	0.572	0.716	0.735
RF	69	0.762	0.821	0.799	0.577	0.716	0.739
RF	70	0.771	0.823	0.803	0.586	0.720	0.745
RF	71	0.778	0.817	0.803	0.586	0.716	0.746
RF	72	0.756	0.811	0.790	0.559	0.703	0.728
RF	73	0.764	0.821	0.800	0.579	0.717	0.740
RF	74	0.753	0.817	0.794	0.564	0.710	0.731
RF	75	0.767	0.820	0.800	0.579	0.716	0.740
RF	76	0.749	0.821	0.794	0.565	0.713	0.730
RF	77	0.782	0.820	0.806	0.593	0.720	0.750
RF	78	0.767	0.821	0.801	0.581	0.718	0.741
RF	79	0.762	0.817	0.797	0.572	0.712	0.736
RF	80	0.764	0.832	0.807	0.591	0.729	0.746
RF	81	0.769	0.819	0.800	0.580	0.715	0.741

RF	82	0.753	0.816	0.793	0.563	0.708	0.730
RF	83	0.758	0.816	0.794	0.567	0.709	0.733
RF	84	0.762	0.820	0.799	0.575	0.715	0.738
RF	85	0.742	0.814	0.787	0.550	0.702	0.722
RF	86	0.767	0.821	0.801	0.581	0.718	0.741
RF	87	0.775	0.808	0.796	0.574	0.705	0.739
RF	88	0.778	0.816	0.802	0.585	0.715	0.745
RF	89	0.771	0.831	0.808	0.595	0.729	0.749
RF	90	0.773	0.820	0.803	0.585	0.718	0.744
RF	91	0.767	0.825	0.803	0.585	0.722	0.744
RF	92	0.751	0.833	0.803	0.581	0.727	0.739
RF	93	0.760	0.816	0.795	0.569	0.710	0.734
RF	94	0.753	0.827	0.799	0.575	0.720	0.736
RF	95	0.749	0.829	0.799	0.574	0.722	0.735
RF	96	0.767	0.823	0.802	0.582	0.719	0.742
RF	97	0.758	0.820	0.797	0.571	0.714	0.735
RF	98	0.762	0.828	0.803	0.584	0.724	0.742
RF	99	0.751	0.820	0.794	0.565	0.712	0.731
RF	100	0.758	0.825	0.800	0.577	0.720	0.738
RF	101	0.760	0.825	0.801	0.579	0.720	0.740
RF	102	0.758	0.819	0.796	0.570	0.712	0.734
RF	103	0.756	0.820	0.796	0.569	0.713	0.734
RF	104	0.771	0.824	0.804	0.588	0.722	0.745
RF	105	0.751	0.819	0.794	0.564	0.710	0.730
RF	106	0.760	0.814	0.794	0.566	0.707	0.732

RF	107	0.773	0.824	0.805	0.590	0.722	0.747
RF	108	0.762	0.824	0.801	0.580	0.719	0.740
RF	109	0.749	0.812	0.789	0.554	0.702	0.725
RF	110	0.778	0.814	0.800	0.582	0.712	0.743
RF	111	0.771	0.814	0.798	0.576	0.710	0.739
RF	112	0.751	0.823	0.796	0.568	0.715	0.733
RF	113	0.762	0.817	0.797	0.572	0.712	0.736
RF	114	0.760	0.821	0.799	0.575	0.716	0.737
RF	115	0.758	0.817	0.795	0.568	0.711	0.733
RF	116	0.778	0.803	0.794	0.570	0.700	0.737
RF	117	0.751	0.816	0.792	0.561	0.707	0.729
RF	118	0.767	0.819	0.799	0.578	0.715	0.740
RF	119	0.756	0.836	0.806	0.587	0.731	0.743
RF	120	0.751	0.819	0.794	0.564	0.710	0.730
RF	121	0.760	0.814	0.794	0.566	0.707	0.732
RF	122	0.762	0.815	0.795	0.569	0.709	0.735
RF	123	0.771	0.819	0.801	0.582	0.716	0.742
RF	124	0.758	0.808	0.790	0.558	0.701	0.728
RF	125	0.758	0.812	0.792	0.562	0.705	0.730
RF	126	0.762	0.812	0.794	0.566	0.706	0.733
RF	127	0.760	0.823	0.799	0.576	0.717	0.738
RF	128	0.778	0.806	0.795	0.573	0.703	0.738
RF	129	0.764	0.817	0.798	0.574	0.713	0.738
RF	130	0.773	0.817	0.801	0.582	0.715	0.743
RF	131	0.760	0.821	0.799	0.575	0.716	0.737

RF	132	0.767	0.828	0.805	0.588	0.725	0.745
RF	133	0.769	0.828	0.806	0.590	0.726	0.747
RF	134	0.769	0.814	0.797	0.574	0.709	0.738
RF	135	0.780	0.819	0.804	0.590	0.718	0.748
RF	136	0.773	0.833	0.811	0.600	0.733	0.752
RF	137	0.773	0.816	0.800	0.581	0.713	0.742
RF	138	0.764	0.814	0.795	0.570	0.708	0.735
RF	139	0.782	0.807	0.798	0.578	0.706	0.742
RF	140	0.775	0.825	0.807	0.593	0.724	0.749
RF	141	0.769	0.820	0.801	0.581	0.717	0.742
RF	142	0.773	0.824	0.805	0.590	0.722	0.747
RF	143	0.773	0.824	0.805	0.590	0.722	0.747
RF	144	0.782	0.832	0.813	0.606	0.733	0.757
RF	145	0.771	0.819	0.801	0.582	0.716	0.742
RF	146	0.782	0.820	0.806	0.593	0.720	0.750
RF	147	0.773	0.831	0.809	0.597	0.730	0.751
RF	148	0.782	0.812	0.801	0.584	0.711	0.745
RF	149	0.782	0.820	0.806	0.593	0.720	0.750
RF	150	0.769	0.827	0.805	0.589	0.724	0.746
RF	151	0.778	0.821	0.805	0.591	0.720	0.748
RF	152	0.784	0.824	0.809	0.599	0.725	0.753
RF	153	0.762	0.816	0.796	0.571	0.710	0.735
RF	154	0.789	0.821	0.809	0.600	0.723	0.754
RF	155	0.771	0.832	0.809	0.597	0.731	0.750
RF	156	0.778	0.824	0.807	0.593	0.723	0.749

RF	157	0.769	0.827	0.805	0.589	0.724	0.746
RF	158	0.780	0.832	0.812	0.604	0.733	0.756
RF	159	0.782	0.817	0.804	0.590	0.717	0.748
RF	160	0.767	0.828	0.805	0.588	0.725	0.745
RF	161	0.775	0.834	0.812	0.604	0.735	0.755
RF	162	0.778	0.831	0.811	0.601	0.731	0.753
RF	163	0.780	0.824	0.808	0.595	0.724	0.751
RF	164	0.771	0.820	0.802	0.583	0.717	0.743
RF	165	0.789	0.817	0.807	0.596	0.719	0.752
RF	166	0.771	0.820	0.802	0.583	0.717	0.743
RF	167	0.789	0.827	0.812	0.606	0.729	0.758
RF	168	0.793	0.827	0.814	0.610	0.730	0.760
RF	169	0.791	0.829	0.815	0.611	0.733	0.761
RF	170	0.806	0.825	0.818	0.621	0.732	0.767
RF	171	0.782	0.824	0.808	0.597	0.724	0.752
RF	172	0.789	0.820	0.808	0.599	0.722	0.754
RF	173	0.802	0.827	0.817	0.618	0.732	0.766
RF	174	0.786	0.829	0.813	0.607	0.732	0.758
RF	175	0.800	0.828	0.817	0.618	0.733	0.765
RF	176	0.793	0.814	0.806	0.596	0.716	0.752
RF	177	0.797	0.825	0.815	0.613	0.730	0.762
RF	178	0.789	0.820	0.808	0.599	0.722	0.754
RF	179	0.786	0.820	0.808	0.597	0.721	0.752
RF	180	0.778	0.824	0.807	0.593	0.723	0.749
RF	181	0.782	0.823	0.808	0.596	0.723	0.751

RF	182	0.782	0.824	0.808	0.597	0.724	0.752
RF	183	0.767	0.814	0.796	0.572	0.709	0.737
RF	184	0.791	0.827	0.813	0.608	0.730	0.759
RF	185	0.800	0.823	0.814	0.612	0.727	0.762
RF	186	0.780	0.821	0.806	0.592	0.721	0.749
RF	187	0.775	0.828	0.808	0.596	0.727	0.751
RF	188	0.778	0.821	0.805	0.591	0.720	0.748
RF	189	0.775	0.824	0.806	0.592	0.723	0.748
RF	190	0.767	0.831	0.807	0.591	0.728	0.747
RF	191	0.769	0.823	0.803	0.584	0.720	0.743
RF	192	0.773	0.828	0.808	0.594	0.727	0.749
RF	193	0.775	0.821	0.804	0.589	0.720	0.747
RF	194	0.778	0.820	0.804	0.589	0.719	0.747
RF	195	0.762	0.832	0.806	0.589	0.728	0.745
RF	196	0.780	0.834	0.814	0.607	0.736	0.757
RF	197	0.758	0.819	0.796	0.570	0.712	0.734
RF	198	0.773	0.821	0.803	0.587	0.719	0.745
RF	199	0.758	0.821	0.798	0.573	0.715	0.736
RF	200	0.771	0.827	0.806	0.591	0.725	0.747
RF	201	0.771	0.832	0.809	0.597	0.731	0.750
RF	202	0.762	0.820	0.799	0.575	0.715	0.738
RF	203	0.769	0.814	0.797	0.574	0.709	0.738
RF	204	0.760	0.821	0.799	0.575	0.716	0.737
RF	205	0.749	0.823	0.795	0.566	0.714	0.731
RF	206	0.758	0.823	0.799	0.574	0.717	0.737

RF	207	0.775	0.825	0.807	0.593	0.724	0.749
RF	208	0.771	0.819	0.801	0.582	0.716	0.742
RF	209	0.760	0.823	0.799	0.576	0.717	0.738
RF	210	0.769	0.827	0.805	0.589	0.724	0.746
RF	211	0.760	0.827	0.802	0.581	0.722	0.740
RF	212	0.773	0.816	0.800	0.581	0.713	0.742
RF	213	0.760	0.828	0.803	0.582	0.723	0.741
RF	214	0.764	0.821	0.800	0.579	0.717	0.740
RF	215	0.756	0.824	0.799	0.574	0.718	0.736
RF	216	0.773	0.812	0.798	0.576	0.709	0.740
RF	217	0.771	0.832	0.809	0.597	0.731	0.750
RF	218	0.758	0.819	0.796	0.570	0.712	0.734
RF	219	0.753	0.819	0.794	0.566	0.711	0.732
RF	220	0.793	0.840	0.822	0.625	0.745	0.768
RF	221	0.793	0.837	0.821	0.622	0.742	0.767
RF	222	0.793	0.833	0.818	0.618	0.738	0.764
RF	223	0.793	0.838	0.821	0.624	0.744	0.768
RF	224	0.789	0.836	0.818	0.617	0.740	0.763
RF	225	0.789	0.837	0.819	0.618	0.741	0.764
RF	226	0.786	0.829	0.813	0.607	0.732	0.758
RF	227	0.802	0.820	0.813	0.611	0.725	0.762
RF	228	0.797	0.831	0.818	0.619	0.736	0.765
RF	229	0.811	0.834	0.826	0.635	0.743	0.776
RF	230	0.811	0.829	0.822	0.629	0.737	0.772
RF	231	0.793	0.834	0.819	0.619	0.739	0.765

RF	232	0.793	0.849	0.828	0.636	0.756	0.774
RF	233	0.791	0.832	0.817	0.614	0.736	0.762
RF	234	0.800	0.837	0.823	0.628	0.744	0.771
RF	235	0.782	0.846	0.822	0.623	0.751	0.766
RF	236	0.804	0.845	0.830	0.641	0.754	0.778
RF	237	0.800	0.838	0.824	0.630	0.745	0.772
RF	238	0.806	0.827	0.819	0.622	0.733	0.768
RF	239	0.791	0.841	0.822	0.625	0.746	0.768
RF	240	0.793	0.832	0.817	0.616	0.736	0.764
RF	241	0.789	0.841	0.821	0.623	0.746	0.767
RF	242	0.804	0.842	0.828	0.638	0.751	0.777
RF	243	0.789	0.831	0.815	0.611	0.734	0.760
RF	244	0.795	0.847	0.828	0.636	0.755	0.775
RF	245	0.795	0.838	0.822	0.626	0.744	0.769
RF	246	0.793	0.832	0.817	0.616	0.736	0.764
RF	247	0.800	0.850	0.831	0.643	0.759	0.779
RF	248	0.819	0.840	0.832	0.649	0.752	0.784
RF	249	0.795	0.823	0.812	0.608	0.726	0.759
RF	250	0.800	0.846	0.829	0.639	0.755	0.776
RF	251	0.778	0.837	0.815	0.609	0.738	0.758
RF	252	0.793	0.834	0.819	0.619	0.739	0.765
RF	253	0.786	0.827	0.812	0.604	0.729	0.756
RF	254	0.784	0.829	0.812	0.605	0.731	0.757
RF	255	0.786	0.837	0.818	0.616	0.741	0.763
RF	256	0.806	0.834	0.824	0.631	0.742	0.773

RF	257	0.780	0.829	0.811	0.601	0.730	0.754
RF	258	0.789	0.837	0.819	0.618	0.741	0.764
RF	259	0.802	0.829	0.819	0.621	0.735	0.767
RF	260	0.791	0.837	0.820	0.620	0.742	0.765
RF	261	0.782	0.845	0.821	0.622	0.749	0.765
RF	262	0.789	0.828	0.813	0.608	0.731	0.758
RF	263	0.786	0.837	0.818	0.616	0.741	0.763
RF	264	0.786	0.837	0.818	0.616	0.741	0.763
RF	265	0.758	0.841	0.810	0.596	0.738	0.748
RF	266	0.806	0.833	0.823	0.629	0.741	0.772
RF	267	0.791	0.842	0.823	0.626	0.748	0.769
RF	268	0.791	0.829	0.815	0.611	0.733	0.761
RF	269	0.793	0.834	0.819	0.619	0.739	0.765
RF	270	0.780	0.832	0.812	0.604	0.733	0.756
RF	271	0.784	0.838	0.818	0.616	0.742	0.762
RF	272	0.797	0.837	0.822	0.626	0.743	0.769
RF	273	0.797	0.832	0.819	0.620	0.737	0.766
RF	274	0.795	0.833	0.819	0.620	0.738	0.766
RF	275	0.786	0.831	0.814	0.609	0.733	0.759
RF	276	0.789	0.834	0.817	0.615	0.738	0.763
RF	277	0.795	0.829	0.817	0.615	0.734	0.763
RF	278	0.786	0.834	0.817	0.613	0.738	0.761
RF	279	0.793	0.844	0.825	0.630	0.750	0.771
RF	280	0.797	0.831	0.818	0.619	0.736	0.765
RF	281	0.795	0.831	0.817	0.617	0.735	0.764

RF	282	0.800	0.831	0.819	0.621	0.736	0.767
RF	283	0.797	0.831	0.818	0.619	0.736	0.765
RF	284	0.800	0.827	0.817	0.616	0.732	0.764
RF	285	0.811	0.832	0.824	0.632	0.740	0.774
RF	286	0.808	0.824	0.818	0.621	0.731	0.768
RF	287	0.804	0.831	0.821	0.625	0.737	0.769
RF	288	0.789	0.832	0.816	0.612	0.735	0.761
RF	289	0.795	0.833	0.819	0.620	0.738	0.766
RF	290	0.797	0.825	0.815	0.613	0.730	0.762
RF	291	0.789	0.816	0.806	0.595	0.717	0.751
RF	292	0.806	0.827	0.819	0.622	0.733	0.768
RF	293	0.786	0.829	0.813	0.607	0.732	0.758
RF	294	0.780	0.842	0.819	0.617	0.745	0.762
RF	295	0.786	0.833	0.816	0.612	0.736	0.760
RF	296	0.808	0.829	0.821	0.627	0.737	0.771
RF	297	0.806	0.836	0.825	0.632	0.744	0.774
RF	298	0.797	0.823	0.813	0.610	0.727	0.761
RF	299	0.780	0.827	0.809	0.598	0.727	0.752
RF	300	0.802	0.825	0.817	0.617	0.731	0.765
RF	301	0.797	0.829	0.817	0.617	0.734	0.765
RF	302	0.793	0.824	0.812	0.607	0.727	0.759
RF	303	0.793	0.832	0.817	0.616	0.736	0.764
RF	304	0.775	0.831	0.810	0.599	0.730	0.752
RF	305	0.786	0.833	0.816	0.612	0.736	0.760
RF	306	0.797	0.828	0.817	0.616	0.733	0.764

RF	307	0.780	0.832	0.812	0.604	0.733	0.756
RF	308	0.782	0.820	0.806	0.593	0.720	0.750
RF	309	0.789	0.829	0.814	0.609	0.732	0.759
RF	310	0.784	0.823	0.808	0.598	0.724	0.753
RF	311	0.793	0.825	0.813	0.609	0.729	0.759
RF	312	0.782	0.834	0.815	0.609	0.737	0.759
RF	313	0.789	0.836	0.818	0.617	0.740	0.763
RF	314	0.784	0.841	0.820	0.619	0.745	0.764
RF	315	0.780	0.832	0.812	0.604	0.733	0.756
RF	316	0.813	0.842	0.831	0.646	0.753	0.782
RF	317	0.797	0.836	0.821	0.625	0.742	0.769
RF	318	0.800	0.825	0.816	0.615	0.730	0.763
RF	319	0.793	0.823	0.812	0.606	0.726	0.758
RF	320	0.795	0.834	0.820	0.621	0.740	0.766
RF	321	0.789	0.828	0.813	0.608	0.731	0.758
RF	322	0.771	0.829	0.808	0.594	0.728	0.749
RF	323	0.791	0.832	0.817	0.614	0.736	0.762
RF	324	0.767	0.832	0.808	0.593	0.730	0.748
RF	325	0.789	0.824	0.811	0.603	0.726	0.756
RF	326	0.791	0.832	0.817	0.614	0.736	0.762
RF	327	0.782	0.829	0.812	0.603	0.730	0.755
RF	328	0.789	0.829	0.814	0.609	0.732	0.759
RF	329	0.802	0.832	0.821	0.624	0.738	0.769
RF	330	0.791	0.845	0.825	0.629	0.751	0.770
RF	331	0.789	0.832	0.816	0.612	0.735	0.761

RF	332	0.791	0.847	0.826	0.632	0.754	0.772
RF	333	0.767	0.828	0.805	0.588	0.725	0.745
RF	334	0.793	0.844	0.825	0.630	0.750	0.771
RF	335	0.782	0.832	0.813	0.606	0.733	0.757
RF	336	0.791	0.834	0.818	0.617	0.739	0.764
RF	337	0.795	0.833	0.819	0.620	0.738	0.766
RF	338	0.791	0.840	0.821	0.623	0.745	0.767
RF	339	0.811	0.841	0.830	0.642	0.751	0.780
RF	340	0.786	0.845	0.823	0.625	0.750	0.768
RF	341	0.815	0.838	0.830	0.643	0.749	0.781
RF	342	0.802	0.836	0.823	0.629	0.743	0.771
RF	343	0.808	0.850	0.835	0.651	0.761	0.784
RF	344	0.795	0.840	0.823	0.627	0.746	0.770
RF	345	0.804	0.836	0.824	0.631	0.743	0.772
RF	346	0.797	0.833	0.820	0.622	0.739	0.767
RF	347	0.795	0.833	0.819	0.620	0.738	0.766
RF	348	0.795	0.841	0.824	0.629	0.747	0.771
RF	349	0.800	0.863	0.839	0.659	0.776	0.787
RF	350	0.806	0.841	0.828	0.638	0.750	0.777
RF	351	0.802	0.851	0.833	0.647	0.762	0.781
RF	352	0.817	0.859	0.844	0.669	0.775	0.795
RF	353	0.815	0.838	0.830	0.643	0.749	0.781
RF	354	0.804	0.854	0.835	0.652	0.765	0.784
RF	355	0.786	0.860	0.833	0.644	0.769	0.778
RF	356	0.782	0.850	0.825	0.628	0.755	0.768

RF	357	0.775	0.849	0.821	0.620	0.752	0.764
RF	358	0.793	0.850	0.829	0.637	0.758	0.775
RF	359	0.804	0.854	0.835	0.652	0.765	0.784
RF	360	0.778	0.847	0.821	0.621	0.751	0.764
RF	361	0.804	0.858	0.838	0.656	0.770	0.787
RF	362	0.804	0.854	0.835	0.652	0.765	0.784
RF	363	0.815	0.863	0.845	0.672	0.779	0.797
RF	364	0.802	0.857	0.836	0.653	0.768	0.784
RF	365	0.813	0.868	0.848	0.676	0.785	0.799
RF	366	0.819	0.867	0.849	0.681	0.785	0.802
RF	367	0.808	0.858	0.839	0.660	0.771	0.789
RF	368	0.815	0.860	0.844	0.669	0.776	0.795
RF	369	0.808	0.867	0.845	0.671	0.783	0.795
RF	370	0.833	0.870	0.856	0.695	0.791	0.811
SVM	1	0.410	0.700	0.592	0.112	0.447	0.428
SVM	2	0.570	0.602	0.591	0.168	0.459	0.509
SVM	3	0.566	0.602	0.589	0.163	0.457	0.506
SVM	4	0.648	0.570	0.599	0.210	0.471	0.545
SVM	5	0.678	0.532	0.586	0.204	0.462	0.550
SVM	6	0.674	0.528	0.582	0.196	0.458	0.545
SVM	7	0.674	0.618	0.639	0.282	0.511	0.581
SVM	8	0.621	0.647	0.637	0.260	0.510	0.560
SVM	9	0.667	0.648	0.655	0.305	0.529	0.590
SVM	10	0.711	0.641	0.667	0.341	0.540	0.614
SVM	11	0.698	0.628	0.654	0.316	0.527	0.600

SVM	12	0.665	0.651	0.656	0.306	0.530	0.590
SVM	13	0.674	0.660	0.665	0.323	0.540	0.599
SVM	14	0.681	0.661	0.668	0.331	0.543	0.604
SVM	15	0.700	0.677	0.686	0.365	0.562	0.624
SVM	16	0.705	0.681	0.690	0.374	0.566	0.628
SVM	17	0.714	0.657	0.678	0.359	0.552	0.622
SVM	18	0.709	0.660	0.678	0.357	0.552	0.621
SVM	19	0.689	0.666	0.675	0.345	0.550	0.612
SVM	20	0.692	0.661	0.672	0.341	0.547	0.611
SVM	21	0.687	0.665	0.673	0.341	0.548	0.610
SVM	22	0.689	0.695	0.693	0.374	0.572	0.625
SVM	23	0.694	0.683	0.687	0.366	0.565	0.623
SVM	24	0.705	0.695	0.699	0.388	0.578	0.635
SVM	25	0.720	0.691	0.702	0.399	0.580	0.642
SVM	26	0.718	0.695	0.704	0.401	0.582	0.643
SVM	27	0.747	0.703	0.719	0.436	0.598	0.664
SVM	28	0.753	0.716	0.730	0.455	0.611	0.675
SVM	29	0.740	0.716	0.725	0.443	0.606	0.667
SVM	30	0.740	0.711	0.722	0.437	0.602	0.664
SVM	31	0.747	0.709	0.723	0.442	0.603	0.667
SVM	32	0.751	0.707	0.723	0.444	0.602	0.669
SVM	33	0.747	0.698	0.716	0.430	0.594	0.661
SVM	34	0.709	0.670	0.685	0.367	0.560	0.626
SVM	35	0.718	0.668	0.686	0.373	0.561	0.630
SVM	36	0.727	0.661	0.686	0.375	0.559	0.632

SVM	37	0.738	0.670	0.695	0.395	0.570	0.643
SVM	38	0.731	0.675	0.696	0.394	0.571	0.642
SVM	39	0.749	0.694	0.714	0.428	0.591	0.661
SVM	40	0.747	0.696	0.715	0.429	0.593	0.661
SVM	41	0.742	0.696	0.713	0.425	0.591	0.658
SVM	42	0.747	0.704	0.720	0.437	0.599	0.665
SVM	43	0.738	0.701	0.715	0.426	0.594	0.658
SVM	44	0.722	0.718	0.720	0.429	0.603	0.657
SVM	45	0.727	0.712	0.717	0.426	0.599	0.657
SVM	46	0.722	0.709	0.714	0.419	0.595	0.653
SVM	47	0.707	0.716	0.713	0.412	0.596	0.647
SVM	48	0.725	0.716	0.719	0.428	0.601	0.657
SVM	49	0.716	0.708	0.711	0.412	0.592	0.648
SVM	50	0.718	0.712	0.714	0.418	0.596	0.651
SVM	51	0.714	0.704	0.708	0.406	0.588	0.645
SVM	52	0.722	0.701	0.709	0.411	0.589	0.649
SVM	53	0.751	0.721	0.732	0.458	0.614	0.676
SVM	54	0.780	0.733	0.750	0.497	0.633	0.699
SVM	55	0.791	0.744	0.762	0.519	0.647	0.712
SVM	56	0.771	0.760	0.764	0.517	0.655	0.709
SVM	57	0.764	0.748	0.754	0.499	0.643	0.698
SVM	58	0.771	0.769	0.770	0.527	0.664	0.714
SVM	59	0.767	0.765	0.766	0.519	0.659	0.709
SVM	60	0.753	0.754	0.753	0.494	0.644	0.694
SVM	61	0.747	0.750	0.749	0.484	0.638	0.688

SVM	62	0.744	0.750	0.748	0.482	0.638	0.687
SVM	63	0.749	0.757	0.754	0.494	0.646	0.694
SVM	64	0.769	0.746	0.754	0.500	0.642	0.699
SVM	65	0.769	0.771	0.770	0.526	0.665	0.713
SVM	66	0.773	0.776	0.775	0.536	0.671	0.719
SVM	67	0.771	0.777	0.775	0.535	0.672	0.718
SVM	68	0.773	0.778	0.776	0.539	0.674	0.720
SVM	69	0.771	0.781	0.777	0.540	0.676	0.720
SVM	70	0.767	0.780	0.775	0.534	0.673	0.717
SVM	71	0.767	0.773	0.771	0.527	0.667	0.713
SVM	72	0.764	0.781	0.775	0.534	0.674	0.716
SVM	73	0.762	0.778	0.772	0.529	0.671	0.713
SVM	74	0.762	0.780	0.773	0.530	0.672	0.714
SVM	75	0.764	0.777	0.772	0.529	0.670	0.714
SVM	76	0.771	0.774	0.773	0.533	0.669	0.716
SVM	77	0.775	0.768	0.771	0.530	0.664	0.715
SVM	78	0.775	0.768	0.771	0.530	0.664	0.715
SVM	79	0.775	0.769	0.771	0.531	0.665	0.716
SVM	80	0.771	0.772	0.771	0.530	0.667	0.715
SVM	81	0.769	0.769	0.769	0.525	0.663	0.712
SVM	82	0.769	0.769	0.769	0.525	0.663	0.712
SVM	83	0.767	0.773	0.771	0.527	0.667	0.713
SVM	84	0.769	0.771	0.770	0.526	0.665	0.713
SVM	85	0.764	0.771	0.768	0.522	0.663	0.710
SVM	86	0.767	0.768	0.767	0.522	0.662	0.710

SVM	87	0.778	0.774	0.776	0.539	0.671	0.720
SVM	88	0.778	0.776	0.776	0.540	0.672	0.721
SVM	89	0.784	0.776	0.779	0.546	0.674	0.725
SVM	90	0.773	0.773	0.773	0.533	0.669	0.717
SVM	91	0.775	0.780	0.778	0.542	0.676	0.722
SVM	92	0.771	0.781	0.777	0.540	0.676	0.720
SVM	93	0.773	0.777	0.776	0.537	0.672	0.719
SVM	94	0.769	0.777	0.774	0.533	0.671	0.717
SVM	95	0.760	0.776	0.770	0.524	0.667	0.711
SVM	96	0.764	0.777	0.772	0.529	0.670	0.714
SVM	97	0.764	0.776	0.771	0.528	0.669	0.713
SVM	98	0.767	0.772	0.770	0.526	0.665	0.712
SVM	99	0.753	0.774	0.767	0.516	0.664	0.706
SVM	100	0.749	0.778	0.767	0.517	0.667	0.705
SVM	101	0.775	0.769	0.771	0.531	0.665	0.716
SVM	102	0.775	0.771	0.772	0.533	0.667	0.717
SVM	103	0.775	0.771	0.772	0.533	0.667	0.717
SVM	104	0.764	0.772	0.769	0.524	0.665	0.711
SVM	105	0.764	0.769	0.767	0.521	0.662	0.710
SVM	106	0.762	0.771	0.767	0.520	0.663	0.709
SVM	107	0.769	0.773	0.771	0.529	0.667	0.714
SVM	108	0.767	0.774	0.771	0.529	0.668	0.714
SVM	109	0.758	0.771	0.766	0.516	0.662	0.706
SVM	110	0.780	0.751	0.762	0.516	0.650	0.709
SVM	111	0.778	0.754	0.762	0.517	0.651	0.709

SVM	112	0.780	0.751	0.762	0.516	0.650	0.709
SVM	113	0.775	0.760	0.766	0.521	0.657	0.711
SVM	114	0.780	0.754	0.763	0.519	0.652	0.710
SVM	115	0.769	0.761	0.764	0.517	0.656	0.708
SVM	116	0.767	0.760	0.762	0.513	0.654	0.706
SVM	117	0.767	0.765	0.766	0.519	0.659	0.709
SVM	118	0.753	0.767	0.762	0.508	0.656	0.702
SVM	119	0.742	0.768	0.758	0.499	0.654	0.696
SVM	120	0.751	0.763	0.758	0.502	0.652	0.698
SVM	121	0.747	0.763	0.757	0.498	0.651	0.695
SVM	122	0.756	0.767	0.762	0.510	0.657	0.703
SVM	123	0.764	0.772	0.769	0.524	0.665	0.711
SVM	124	0.749	0.767	0.760	0.504	0.655	0.699
SVM	125	0.744	0.764	0.757	0.497	0.651	0.695
SVM	126	0.751	0.768	0.762	0.507	0.657	0.701
SVM	127	0.749	0.767	0.760	0.504	0.655	0.699
SVM	128	0.744	0.782	0.768	0.517	0.669	0.705
SVM	129	0.767	0.772	0.770	0.526	0.665	0.712
SVM	130	0.767	0.774	0.771	0.529	0.668	0.714
SVM	131	0.758	0.769	0.765	0.515	0.660	0.706
SVM	132	0.764	0.760	0.762	0.511	0.653	0.705
SVM	133	0.756	0.768	0.763	0.511	0.658	0.704
SVM	134	0.751	0.763	0.758	0.502	0.652	0.698
SVM	135	0.760	0.764	0.762	0.511	0.656	0.704
SVM	136	0.762	0.767	0.765	0.516	0.659	0.707

SVM	137	0.769	0.768	0.768	0.524	0.662	0.712
SVM	138	0.769	0.769	0.769	0.525	0.663	0.712
SVM	139	0.762	0.765	0.764	0.515	0.658	0.706
SVM	140	0.764	0.765	0.765	0.517	0.658	0.707
SVM	141	0.771	0.763	0.766	0.520	0.658	0.710
SVM	142	0.773	0.761	0.766	0.521	0.657	0.711
SVM	143	0.780	0.763	0.769	0.528	0.660	0.715
SVM	144	0.771	0.767	0.768	0.524	0.662	0.712
SVM	145	0.782	0.754	0.764	0.521	0.653	0.711
SVM	146	0.780	0.765	0.771	0.531	0.663	0.717
SVM	147	0.782	0.771	0.775	0.539	0.669	0.721
SVM	148	0.778	0.748	0.759	0.511	0.647	0.706
SVM	149	0.775	0.750	0.759	0.510	0.647	0.705
SVM	150	0.780	0.750	0.761	0.515	0.648	0.708
SVM	151	0.791	0.776	0.781	0.552	0.676	0.729
SVM	152	0.789	0.773	0.779	0.547	0.673	0.726
SVM	153	0.789	0.772	0.778	0.546	0.672	0.725
SVM	154	0.786	0.776	0.780	0.548	0.675	0.726
SVM	155	0.784	0.777	0.780	0.548	0.676	0.726
SVM	156	0.782	0.782	0.782	0.551	0.680	0.727
SVM	157	0.773	0.785	0.781	0.546	0.680	0.724
SVM	158	0.782	0.776	0.778	0.544	0.674	0.724
SVM	159	0.784	0.772	0.776	0.542	0.670	0.723
SVM	160	0.784	0.773	0.777	0.543	0.672	0.724
SVM	161	0.806	0.769	0.783	0.560	0.674	0.734

SVM	162	0.806	0.772	0.785	0.562	0.677	0.736
SVM	163	0.800	0.771	0.781	0.555	0.673	0.731
SVM	164	0.795	0.767	0.777	0.547	0.669	0.726
SVM	165	0.800	0.767	0.779	0.551	0.670	0.729
SVM	166	0.795	0.768	0.778	0.548	0.670	0.727
SVM	167	0.806	0.767	0.781	0.557	0.672	0.733
SVM	168	0.806	0.763	0.779	0.553	0.668	0.731
SVM	169	0.806	0.771	0.784	0.561	0.675	0.735
SVM	170	0.811	0.763	0.781	0.557	0.669	0.733
SVM	171	0.813	0.764	0.782	0.560	0.671	0.735
SVM	172	0.813	0.764	0.782	0.560	0.671	0.735
SVM	173	0.806	0.756	0.775	0.546	0.662	0.727
SVM	174	0.813	0.756	0.777	0.552	0.664	0.731
SVM	175	0.815	0.755	0.777	0.553	0.663	0.731
SVM	176	0.817	0.754	0.777	0.554	0.663	0.732
SVM	177	0.806	0.764	0.780	0.554	0.669	0.731
SVM	178	0.800	0.755	0.771	0.538	0.659	0.722
SVM	179	0.800	0.756	0.772	0.540	0.660	0.723
SVM	180	0.802	0.754	0.771	0.539	0.658	0.723
SVM	181	0.806	0.755	0.774	0.545	0.661	0.726
SVM	182	0.802	0.750	0.769	0.535	0.655	0.721
SVM	183	0.802	0.752	0.771	0.538	0.657	0.722
SVM	184	0.800	0.761	0.776	0.545	0.665	0.726
SVM	185	0.804	0.759	0.776	0.547	0.664	0.727
SVM	186	0.778	0.773	0.775	0.537	0.670	0.720

SVM	187	0.771	0.772	0.771	0.530	0.667	0.715
SVM	188	0.764	0.774	0.771	0.527	0.667	0.713
SVM	189	0.758	0.780	0.771	0.526	0.671	0.711
SVM	190	0.760	0.782	0.774	0.531	0.674	0.714
SVM	191	0.744	0.780	0.767	0.514	0.667	0.703
SVM	192	0.744	0.780	0.767	0.514	0.667	0.703
SVM	193	0.744	0.780	0.767	0.514	0.667	0.703
SVM	194	0.753	0.778	0.769	0.521	0.668	0.708
SVM	195	0.771	0.767	0.768	0.524	0.662	0.712
SVM	196	0.780	0.769	0.773	0.535	0.667	0.719
SVM	197	0.780	0.769	0.773	0.535	0.667	0.719
SVM	198	0.784	0.771	0.776	0.541	0.669	0.722
SVM	199	0.782	0.767	0.772	0.534	0.665	0.719
SVM	200	0.780	0.767	0.771	0.532	0.664	0.717
SVM	201	0.780	0.767	0.771	0.532	0.664	0.717
SVM	202	0.786	0.765	0.773	0.537	0.665	0.720
SVM	203	0.791	0.772	0.779	0.548	0.672	0.727
SVM	204	0.789	0.772	0.778	0.546	0.672	0.725
SVM	205	0.789	0.773	0.779	0.547	0.673	0.726
SVM	206	0.782	0.769	0.774	0.537	0.667	0.720
SVM	207	0.782	0.769	0.774	0.537	0.667	0.720
SVM	208	0.782	0.773	0.776	0.541	0.671	0.722
SVM	209	0.782	0.773	0.776	0.541	0.671	0.722
SVM	210	0.784	0.774	0.778	0.545	0.673	0.724
SVM	211	0.784	0.771	0.776	0.541	0.669	0.722

SVM	212	0.786	0.772	0.777	0.544	0.671	0.724
SVM	213	0.786	0.774	0.779	0.547	0.674	0.726
SVM	214	0.786	0.771	0.776	0.543	0.670	0.723
SVM	215	0.784	0.771	0.776	0.541	0.669	0.722
SVM	216	0.793	0.807	0.802	0.588	0.709	0.748
SVM	217	0.793	0.807	0.802	0.588	0.709	0.748
SVM	218	0.793	0.807	0.802	0.588	0.709	0.748
SVM	219	0.795	0.801	0.799	0.583	0.702	0.746
SVM	220	0.819	0.823	0.821	0.630	0.732	0.773
SVM	221	0.815	0.831	0.825	0.634	0.740	0.776
SVM	222	0.813	0.836	0.827	0.638	0.745	0.778
SVM	223	0.813	0.836	0.827	0.638	0.745	0.778
SVM	224	0.813	0.836	0.827	0.638	0.745	0.778
SVM	225	0.813	0.836	0.827	0.638	0.745	0.778
SVM	226	0.815	0.836	0.828	0.640	0.746	0.779
SVM	227	0.813	0.836	0.827	0.638	0.745	0.778
SVM	228	0.813	0.836	0.827	0.638	0.745	0.778
SVM	229	0.813	0.834	0.826	0.637	0.744	0.777
SVM	230	0.815	0.833	0.826	0.637	0.743	0.777
SVM	231	0.817	0.836	0.829	0.642	0.746	0.780
SVM	232	0.817	0.833	0.827	0.639	0.743	0.779
SVM	233	0.813	0.832	0.825	0.634	0.741	0.775
SVM	234	0.830	0.840	0.836	0.659	0.754	0.790
SVM	235	0.826	0.838	0.834	0.653	0.752	0.787
SVM	236	0.828	0.836	0.833	0.652	0.749	0.787

SVM	237	0.835	0.836	0.835	0.658	0.750	0.790
SVM	238	0.830	0.829	0.830	0.647	0.742	0.784
SVM	239	0.826	0.838	0.834	0.653	0.752	0.787
SVM	240	0.826	0.838	0.834	0.653	0.752	0.787
SVM	241	0.811	0.838	0.828	0.639	0.748	0.778
SVM	242	0.817	0.837	0.830	0.644	0.748	0.781
SVM	243	0.817	0.837	0.830	0.644	0.748	0.781
SVM	244	0.817	0.837	0.830	0.644	0.748	0.781
SVM	245	0.817	0.838	0.830	0.645	0.749	0.782
SVM	246	0.817	0.838	0.830	0.645	0.749	0.782
SVM	247	0.817	0.840	0.831	0.647	0.751	0.783
SVM	248	0.815	0.836	0.828	0.640	0.746	0.779
SVM	249	0.808	0.831	0.822	0.628	0.738	0.772
SVM	250	0.808	0.831	0.822	0.628	0.738	0.772
SVM	251	0.813	0.832	0.825	0.634	0.741	0.775
SVM	252	0.817	0.831	0.826	0.636	0.741	0.777
SVM	253	0.817	0.831	0.826	0.636	0.741	0.777
SVM	254	0.817	0.829	0.825	0.635	0.739	0.776
SVM	255	0.817	0.831	0.826	0.636	0.741	0.777
SVM	256	0.813	0.832	0.825	0.634	0.741	0.775
SVM	257	0.817	0.831	0.826	0.636	0.741	0.777
SVM	258	0.815	0.832	0.826	0.636	0.741	0.776
SVM	259	0.819	0.837	0.830	0.646	0.748	0.782
SVM	260	0.817	0.838	0.830	0.645	0.749	0.782
SVM	261	0.819	0.838	0.831	0.647	0.750	0.783

SVM	262	0.815	0.837	0.829	0.642	0.747	0.780
SVM	263	0.815	0.837	0.829	0.642	0.747	0.780
SVM	264	0.822	0.834	0.830	0.645	0.746	0.782
SVM	265	0.813	0.829	0.823	0.631	0.738	0.774
SVM	266	0.815	0.829	0.824	0.633	0.739	0.775
SVM	267	0.815	0.829	0.824	0.633	0.739	0.775
SVM	268	0.815	0.828	0.823	0.631	0.737	0.774
SVM	269	0.822	0.828	0.826	0.637	0.739	0.778
SVM	270	0.822	0.827	0.825	0.636	0.737	0.777
SVM	271	0.824	0.827	0.826	0.638	0.738	0.778
SVM	272	0.826	0.827	0.826	0.640	0.738	0.780
SVM	273	0.828	0.827	0.827	0.642	0.739	0.781
SVM	274	0.822	0.827	0.825	0.636	0.737	0.777
SVM	275	0.822	0.827	0.825	0.636	0.737	0.777
SVM	276	0.819	0.829	0.826	0.637	0.740	0.777
SVM	277	0.819	0.828	0.825	0.635	0.738	0.777
SVM	278	0.822	0.828	0.826	0.637	0.739	0.778
SVM	279	0.822	0.828	0.826	0.637	0.739	0.778
SVM	280	0.822	0.814	0.817	0.621	0.723	0.769
SVM	281	0.822	0.814	0.817	0.621	0.723	0.769
SVM	282	0.822	0.814	0.817	0.621	0.723	0.769
SVM	283	0.822	0.814	0.817	0.621	0.723	0.769
SVM	284	0.819	0.812	0.815	0.618	0.721	0.767
SVM	285	0.819	0.812	0.815	0.618	0.721	0.767
SVM	286	0.819	0.808	0.812	0.614	0.717	0.765

SVM	287	0.833	0.812	0.820	0.630	0.724	0.775
SVM	288	0.835	0.815	0.822	0.635	0.727	0.777
SVM	289	0.833	0.808	0.817	0.626	0.720	0.772
SVM	290	0.833	0.808	0.817	0.626	0.720	0.772
SVM	291	0.833	0.811	0.819	0.629	0.723	0.774
SVM	292	0.833	0.810	0.818	0.627	0.721	0.773
SVM	293	0.833	0.810	0.818	0.627	0.721	0.773
SVM	294	0.830	0.810	0.817	0.625	0.721	0.772
SVM	295	0.830	0.810	0.817	0.625	0.721	0.772
SVM	296	0.830	0.807	0.816	0.622	0.718	0.770
SVM	297	0.830	0.810	0.817	0.625	0.721	0.772
SVM	298	0.830	0.810	0.817	0.625	0.721	0.772
SVM	299	0.828	0.811	0.817	0.625	0.722	0.771
SVM	300	0.828	0.810	0.817	0.623	0.720	0.770
SVM	301	0.824	0.810	0.815	0.619	0.719	0.768
SVM	302	0.830	0.808	0.817	0.624	0.719	0.771
SVM	303	0.830	0.808	0.817	0.624	0.719	0.771
SVM	304	0.828	0.814	0.819	0.627	0.724	0.773
SVM	305	0.824	0.811	0.816	0.621	0.721	0.769
SVM	306	0.824	0.810	0.815	0.619	0.719	0.768
SVM	307	0.824	0.810	0.815	0.619	0.719	0.768
SVM	308	0.826	0.811	0.817	0.623	0.721	0.770
SVM	309	0.826	0.812	0.817	0.624	0.723	0.771
SVM	310	0.833	0.810	0.818	0.627	0.721	0.773
SVM	311	0.833	0.810	0.818	0.627	0.721	0.773

SVM	312	0.835	0.808	0.818	0.628	0.721	0.773
SVM	313	0.833	0.807	0.817	0.624	0.719	0.771
SVM	314	0.833	0.807	0.817	0.624	0.719	0.771
SVM	315	0.835	0.806	0.817	0.625	0.718	0.772
SVM	316	0.835	0.806	0.817	0.625	0.718	0.772
SVM	317	0.835	0.804	0.816	0.623	0.716	0.771
SVM	318	0.826	0.806	0.813	0.617	0.716	0.767
SVM	319	0.830	0.808	0.817	0.624	0.719	0.771
SVM	320	0.830	0.808	0.817	0.624	0.719	0.771
SVM	321	0.828	0.810	0.817	0.623	0.720	0.770
SVM	322	0.828	0.810	0.817	0.623	0.720	0.770
SVM	323	0.828	0.810	0.817	0.623	0.720	0.770
SVM	324	0.828	0.810	0.817	0.623	0.720	0.770
SVM	325	0.833	0.821	0.826	0.640	0.734	0.780
SVM	326	0.833	0.820	0.825	0.639	0.733	0.779
SVM	327	0.833	0.819	0.824	0.637	0.731	0.779
SVM	328	0.828	0.817	0.821	0.632	0.729	0.775
SVM	329	0.828	0.817	0.821	0.632	0.729	0.775
SVM	330	0.835	0.831	0.832	0.652	0.745	0.787
SVM	331	0.837	0.829	0.832	0.653	0.744	0.788
SVM	332	0.837	0.829	0.832	0.653	0.744	0.788
SVM	333	0.837	0.828	0.831	0.651	0.742	0.787
SVM	334	0.835	0.829	0.831	0.651	0.743	0.786
SVM	335	0.828	0.831	0.830	0.646	0.743	0.783
SVM	336	0.828	0.831	0.830	0.646	0.743	0.783

SVM	337	0.828	0.831	0.830	0.646	0.743	0.783
SVM	338	0.830	0.834	0.833	0.653	0.748	0.787
SVM	339	0.830	0.833	0.832	0.651	0.747	0.786
SVM	340	0.830	0.837	0.835	0.656	0.751	0.789
SVM	341	0.826	0.828	0.827	0.641	0.740	0.780
SVM	342	0.826	0.825	0.826	0.638	0.737	0.779
SVM	343	0.833	0.819	0.824	0.637	0.731	0.779
SVM	344	0.839	0.819	0.826	0.643	0.733	0.782
SVM	345	0.839	0.819	0.826	0.643	0.733	0.782
SVM	346	0.841	0.820	0.828	0.647	0.735	0.784
SVM	347	0.841	0.821	0.829	0.648	0.736	0.785
SVM	348	0.841	0.821	0.829	0.648	0.736	0.785
SVM	349	0.841	0.821	0.829	0.648	0.736	0.785
SVM	350	0.848	0.823	0.832	0.655	0.739	0.790
SVM	351	0.848	0.824	0.833	0.657	0.740	0.791
SVM	352	0.848	0.823	0.832	0.655	0.739	0.790
SVM	353	0.848	0.820	0.830	0.653	0.736	0.788
SVM	354	0.846	0.823	0.831	0.653	0.738	0.789
SVM	355	0.826	0.842	0.836	0.658	0.756	0.789
SVM	356	0.830	0.847	0.841	0.667	0.763	0.795
SVM	357	0.830	0.853	0.844	0.673	0.769	0.799
SVM	358	0.833	0.853	0.845	0.675	0.770	0.800
SVM	359	0.835	0.854	0.847	0.679	0.772	0.802
SVM	360	0.833	0.853	0.845	0.675	0.770	0.800
SVM	361	0.830	0.853	0.844	0.673	0.769	0.799

SVM	362	0.824	0.853	0.842	0.668	0.768	0.795
SVM	363	0.824	0.854	0.843	0.669	0.770	0.796
SVM	364	0.826	0.854	0.844	0.671	0.770	0.797
SVM	365	0.830	0.854	0.845	0.675	0.771	0.800
SVM	366	0.837	0.854	0.848	0.681	0.772	0.803
SVM	367	0.841	0.863	0.855	0.695	0.784	0.812
SVM	368	0.848	0.867	0.860	0.706	0.791	0.818
SVM	369	0.859	0.870	0.866	0.718	0.796	0.826
SVM	370	0.852	0.867	0.862	0.710	0.791	0.821
