# Supplementary material for: Identification of Smoking-Associated Transcriptome Aberration in Blood with Machine Learning Methods
Source: Biomed Res Int. 2023 Jan 4;2023:5333361. doi: 10.1155/2023/5333361 (PMC9833906; doi:10.1155/2023/5333361)
Supplement: Supplementary Materials — Table S1: feature ranking results obtained by mRMR, MCFS, LightGBM, and LASSO methods. Table S2: IFS results on different feature lists. Table S3: intersection of the optimal feature subsets extracted from mRMR, MCFS, LightGBM, and LASSO feature lists. The features that appear in 4, 3, 2, and 1 optimal feature subsets are shown. Table S4: classification rules generated by the optimal DT model. Table S5: GO and KEGG enrichment results after merging the optimal feature subsets of the four feature ranking algorithms. [file 5333361.f1.zip › Table S3 (1).pdf]

Table S3: Intersection of the optimal feature subsets extracted from mRMR, MCFS, LightGBM, and LASSO feature lists. The features that appear in 4, 3, 2, and 1 optimal feature subsets are shown.

Features in four optimal feature subsets	Features in three optimal feature subsets	Features in two optimal feature subsets	Features in one optimal feature subset
ENST00000297785	ENST00000324907	ENST00000633685	ENST00000528808
ENST00000359228	ENST00000631690	ENST00000395002	ENST00000262158
ENST00000464591	ENST00000442677	ENST00000476232	ENST00000648973
ENST00000622663	ENST00000424873	ENST00000272224	ENST00000376840
ENST00000509152	ENST00000324079	ENST00000593178	ENST00000394718
ENST00000400072	ENST00000377712	ENST00000623011	ENST00000294435
ENST00000316418	ENST00000311597	ENST00000615525	ENST00000368034
ENST00000393590	ENST00000482769	ENST00000312143	ENST00000380490
ENST00000339223	ENST00000594028	ENST00000244745	ENST00000367721
ENST00000341184	ENST00000611977	ENST00000490251	ENST00000216341
ENST00000441556	ENST00000616259	ENST00000390270	ENST00000436743
ENST00000586582	ENST00000404989	ENST00000473185	ENST00000306051
ENST00000308478	ENST00000295633	ENST00000440480	ENST00000424347
ENST00000367467	ENST00000580335	ENST00000478742	ENST00000258807
ENST00000392054	ENST00000612073	ENST00000618026	ENST00000272163
	ENST00000392040	ENST00000507007	ENST00000480769
	ENST00000632136	ENST00000396276	ENST00000367688
	ENST00000390549	ENST00000426706	ENST00000617889
	ENST00000390547	ENST00000443723	ENST00000366899

	ENST00000641136	ENST00000298527	ENST00000613947
	ENST00000497872	ENST00000498435	ENST00000619589
	ENST00000390539	ENST00000633092	ENST00000369718
	ENST00000390323	ENST00000491761	ENST00000373209
	ENST00000390321	ENST00000316623	ENST00000433557
	ENST00000390237	ENST00000489175	ENST00000319211
	ENST00000632774	ENST00000362032	ENST00000367255
	ENST00000631869	ENST00000641095	ENST00000469439
	ENST00000610349	ENST00000621803	ENST00000177694
	ENST00000448155	ENST00000613640	ENST00000394684
	ENST00000390625	ENST00000390601	ENST00000378867
	ENST00000480492	ENST00000468494	ENST00000540271
	ENST00000611391	ENST00000390256	ENST00000392993
	ENST00000492167	ENST00000390629	ENST00000480757
	ENST00000483158	ENST00000390624	ENST00000235382
	ENST00000496168	ENST00000618644	ENST00000420843
	ENST00000390304	ENST00000454421	ENST00000399220
	ENST00000390305	ENST00000390598	ENST00000555619
	ENST00000464162	ENST00000390283	ENST00000379287
	ENST00000390606	ENST00000390594	ENST00000322019
	ENST00000390243	ENST00000434710	ENST00000498165
	ENST00000390252	ENST00000492446	ENST00000310954
	ENST00000390309	ENST00000491977	ENST00000489171
	ENST00000390306	ENST00000493819	ENST00000262094
	ENST00000390319	ENST00000479981	ENST00000228705

	ENST00000390308	ENST00000390312	ENST00000506073
	ENST00000473726	ENST00000620395	ENST00000248598
	ENST00000390285	ENST00000390310	ENST00000643024
	ENST00000390294	ENST00000357325	ENST00000216117
	ENST00000390290	ENST00000556751	ENST00000381031
	ENST00000390314	ENST00000612503	ENST00000342456
	ENST00000541272	ENST00000368237	ENST00000393158
	ENST00000313401	ENST00000321935	ENST00000321348
	ENST00000396789	ENST00000264808	ENST00000565135
	ENST00000446507	ENST00000550402	ENST00000284311
	ENST00000342032	ENST00000305097	
	ENST00000274605	ENST00000621600	
	ENST00000534952	ENST00000414455	
	ENST00000281821	ENST00000443956	
	ENST00000280258	ENST00000553927	
	ENST00000367929	ENST00000264377	
	ENST00000536374	ENST00000262139	
	ENST00000265022	ENST00000267814	
	ENST00000558711	ENST00000564734	
	ENST00000558197	ENST00000526004	
	ENST00000616417	ENST00000260526	
	ENST00000380672	ENST00000482518	
	ENST00000547327	ENST00000285379	
	ENST00000296029	ENST00000616430	
	ENST00000307428	ENST00000532234	

	ENST00000367256	ENST00000278919	
	ENST00000396625	ENST00000493550	
	ENST00000396578	ENST00000453044	
	ENST00000329099	ENST00000341935	
	ENST00000394329	ENST00000537147	
	ENST00000396618	ENST00000005178	
	ENST00000643697	ENST00000199389	
	ENST00000422987	ENST00000367245	
	ENST00000451085	ENST00000250360	
	ENST00000550772	ENST00000444393	
	ENST00000321535	ENST00000264424	
	ENST00000309575	ENST00000484726	
	ENST00000375448	ENST00000296028	
	ENST00000367434	ENST00000291232	
	ENST00000467942	ENST00000548358	
	ENST00000543780	ENST00000615863	
	ENST00000276974	ENST00000439754	
	ENST00000298223	ENST00000650242	
	ENST00000305141	ENST00000448387	
	ENST00000526893	ENST00000359357	
	ENST00000560582	ENST00000466595	
	ENST00000513778	ENST00000461872	
	ENST00000221307	ENST00000466159	
	ENST00000290902	ENST00000302125	
	ENST00000509697	ENST00000503771	

	ENST00000519554	ENST00000255409	
	ENST00000259089	ENST00000472064	
	ENST00000468856	ENST00000371528	
	ENST00000610495	ENST00000340342	
	ENST00000411764	ENST00000291576	
	ENST00000618003	ENST00000256447	
	ENST00000523272	ENST00000359062	
	ENST00000631065	ENST00000379757	
	ENST00000522551	ENST00000622044	
	ENST00000574371	ENST00000261381	
	ENST00000405943	ENST00000226279	
	ENST00000561418	ENST00000264246	
	ENST00000416931	ENST00000053243	
	ENST00000506927	ENST00000379982	
	ENST00000559610	ENST00000240100	
	ENST00000634222	ENST00000525499	
	ENST00000290866	ENST00000334529	
	ENST00000244174	ENST00000584793	
	ENST00000360851	ENST00000483295	
	ENST00000636279	ENST00000464302	
	ENST00000633446	ENST00000330953	
	ENST00000480786	ENST00000498146	
	ENST00000390325	ENST00000480697	
	ENST00000390548	ENST00000571489	
	ENST00000438425	ENST00000505763	

	ENST00000393203	ENST00000286732	
	ENST00000507411	ENST00000357484	
	ENST00000617175	ENST00000284984	
	ENST00000392055	ENST00000464589	
	ENST00000422622	ENST00000477714	
	ENST00000464835	ENST00000514989	
	ENST00000367051	ENST00000277225	
	ENST00000487272	ENST00000264790	
	ENST00000321016	ENST00000373095	
	ENST00000430223	ENST00000622749	
	ENST00000620457	ENST00000423064	
	ENST00000648322	ENST00000429492	
	ENST00000617716	ENST00000263382	
	ENST00000529814	ENST00000331289	
	ENST00000598234	ENST00000637526	
		ENST00000451998	
		ENST00000610261	
		ENST00000502981	
		ENST00000526097	
		ENST00000403687	
		ENST00000646615	
		ENST00000635923	
		ENST00000521304	
		ENST00000587916	
		ENST00000481799	

		ENST00000513886	
		ENST00000261651	
		ENST00000580919	
		ENST00000555838	
		ENST00000600255	
		ENST00000420836	
		ENST00000508643	
		ENST00000604204	
		ENST00000544802	
		ENST00000561158	
		ENST00000414273	
		ENST00000471490	
		ENST00000200457	
		ENST00000607161	
		ENST00000307407	
		ENST00000618889	
		ENST00000573760	
		ENST00000503004	
		ENST00000507874	
		ENST00000450871	
		ENST00000307851	
		ENST00000259989	
		ENST00000415351	
