# Supplementary material for: Identification of Smoking-Associated Transcriptome Aberration in Blood with Machine Learning Methods
Source: Biomed Res Int. 2023 Jan 4;2023:5333361. doi: 10.1155/2023/5333361 (PMC9833906; doi:10.1155/2023/5333361)
Supplement: Supplementary Materials — Table S1: feature ranking results obtained by mRMR, MCFS, LightGBM, and LASSO methods. Table S2: IFS results on different feature lists. Table S3: intersection of the optimal feature subsets extracted from mRMR, MCFS, LightGBM, and LASSO feature lists. The features that appear in 4, 3, 2, and 1 optimal feature subsets are shown. Table S4: classification rules generated by the optimal DT model. Table S5: GO and KEGG enrichment results after merging the optimal feature subsets of the four feature ranking algorithms. [file 5333361.f1.zip › Table S4 (1).pdf]

**Table S4:** Classification rules generated by the optimal DT model

(1) Rules on mRMR feature ranking results

| Rules_0 | passed counts:445 |
|---|---|
| node_0: feature_name=ENST00000284311 | feature_id[0].value <= threshold=179.44091796875 |
| node_1: feature_name=ENST00000390539 | feature_id[222].value <= threshold=11.91484260559082 |
| node_2: feature_name=ENST00000586582 | feature_id[123].value <= threshold=17.368224143981934 |
| node_3: feature_name=ENST00000284984 | feature_id[292].value > threshold=11.201291561126709 |
| node_23: feature_name=ENST00000276974 | feature_id[6].value <= threshold=75.58467483520508 |
| node_24: feature_name=ENST00000464302 | feature_id[285].value > threshold=25.290170669555664 |
| node_28: feature_name=ENST00000650242 | feature_id[234].value > threshold=1287.966552734375 |
| node_32: feature_name=ENST00000491977 | feature_id[29].value <= threshold=22.76432514190674 |
| node_33: feature_name=ENST00000611977 | feature_id[43].value > threshold=0.45197004079818726 |
| node_35: feature_name=ENST00000503771 | feature_id[193].value <= threshold=320.5686798095703 |
| node_36: feature_name=ENST00000307407 | feature_id[281].value > threshold=4.887194871902466 |
| node_38: feature_name=ENST00000321935 | feature_id[238].value > threshold=22.123645782470703 |
| node_40: feature_name=ENST00000492167 | feature_id[111].value <= threshold=833.0198974609375 |
| node_41: feature_name=ENST00000005178 | feature_id[118].value > threshold=29.375121116638184 |
| node_43: feature_name=ENST00000411764 | feature_id[20].value <= threshold=186.49674224853516 |
| node_44: feature_name=ENST00000395002 | feature_id[256].value > threshold=13.3489990234375 |
| node_48: feature_name=ENST00000636279 | feature_id[103].value <= threshold=9.28081226348877 |
| node_49: feature_name=ENST00000244174 | feature_id[34].value <= threshold=10.21084976196289 |
| node_50: feature_name=ENST00000393203 | feature_id[91].value <= threshold=21.360187530517578 |
| node_51: feature_name=ENST00000278919 | feature_id[121].value <= threshold=45.06623077392578 |
| Class: former_smokers | |
| | |

| | |
|---|---|
| Rules_1 | passed counts:190 |
| node_0: feature_name=ENST00000284311 | feature_id[0].value > threshold=179.44091796875 |
| node_92: feature_name=ENST00000396276 | feature_id[298].value <= threshold=957.0642700195312 |
| node_93: feature_name=ENST00000316418 | feature_id[181].value > threshold=3.5686380863189697 |
| node_111: feature_name=ENST00000309575 | feature_id[257].value <= threshold=375.328125 |
| node_112: feature_name=ENST00000367467 | feature_id[107].value > threshold=21.34785747528076 |
| node_116: feature_name=ENST00000571489 | feature_id[279].value <= threshold=56.20589256286621 |
| node_117: feature_name=ENST00000635923 | feature_id[171].value <= threshold=328.1167755126953 |
| node_118: feature_name=ENST00000334529 | feature_id[148].value > threshold=60.1170597076416 |
| node_120: feature_name=ENST00000635923 | feature_id[171].value > threshold=37.20250701904297 |
| node_124: feature_name=ENST00000498146 | feature_id[143].value > threshold=0.441722571849823 |
| node_128: feature_name=ENST00000260526 | feature_id[236].value <= threshold=21.659213066101074 |
| node_129: feature_name=ENST00000390325 | feature_id[17].value > threshold=17.23688793182373 |
| Class: current_smokers | |
| | |
| Rules_2 | passed counts:61 |
| node_0: feature_name=ENST00000284311 | feature_id[0].value > threshold=179.44091796875 |
| node_92: feature_name=ENST00000396276 | feature_id[298].value > threshold=957.0642700195312 |
| node_150: feature_name=ENST00000284311 | feature_id[0].value <= threshold=364.31639099121094 |
| node_151: feature_name=ENST00000276974 | feature_id[6].value <= threshold=1.20855313539505 |
| node_152: feature_name=ENST00000441556 | feature_id[147].value <= threshold=8.403407096862793 |
| node_153: feature_name=ENST00000390304 | feature_id[81].value <= threshold=9.798711776733398 |
| node_154: feature_name=ENST00000390312 | feature_id[78].value <= threshold=759.6060485839844 |
| Class: former_smokers | |
| | |
| Rules_3 | passed counts:57 |

| | |
|---|---|
| node_0: feature_name=ENST00000284311 | feature_id[0].value <= threshold=179.44091796875 |
| node_1: feature_name=ENST00000390539 | feature_id[222].value <= threshold=11.91484260559082 |
| node_2: feature_name=ENST00000586582 | feature_id[123].value <= threshold=17.368224143981934 |
| node_3: feature_name=ENST00000284984 | feature_id[292].value <= threshold=11.201291561126709 |
| node_4: feature_name=ENST00000586582 | feature_id[123].value <= threshold=5.24660587310791 |
| node_5: feature_name=ENST00000244174 | feature_id[34].value <= threshold=2.29075288772583 |
| node_6: feature_name=ENST00000618889 | feature_id[237].value <= threshold=6.870025634765625 |
| node_7: feature_name=ENST00000453044 | feature_id[174].value <= threshold=72.99653625488281 |
| Class: former_smokers | |
| | |
| Rules_4 | passed counts:55 |
| node_0: feature_name=ENST00000284311 | feature_id[0].value > threshold=179.44091796875 |
| node_92: feature_name=ENST00000396276 | feature_id[298].value > threshold=957.0642700195312 |
| node_150: feature_name=ENST00000284311 | feature_id[0].value > threshold=364.31639099121094 |
| node_170: feature_name=ENST00000339223 | feature_id[10].value > threshold=33.90394592285156 |
| node_176: feature_name=ENST00000513886 | feature_id[39].value <= threshold=141.72513961791992 |
| node_177: feature_name=ENST00000443723 | feature_id[117].value <= threshold=55.98150062561035 |
| node_178: feature_name=ENST00000498146 | feature_id[143].value > threshold=2.1133410930633545 |
| Class: current_smokers | |
| | |
| Rules_5 | passed counts:41 |
| node_0: feature_name=ENST00000284311 | feature_id[0].value <= threshold=179.44091796875 |
| node_1: feature_name=ENST00000390539 | feature_id[222].value <= threshold=11.91484260559082 |
| node_2: feature_name=ENST00000586582 | feature_id[123].value <= threshold=17.368224143981934 |
| node_3: feature_name=ENST00000284984 | feature_id[292].value > threshold=11.201291561126709 |
| node_23: feature_name=ENST00000276974 | feature_id[6].value <= threshold=75.58467483520508 |

| | |
|---|---|
| node_24: feature_name=ENST00000464302 | feature_id[285].value > threshold=25.290170669555664 |
| node_28: feature_name=ENST00000650242 | feature_id[234].value > threshold=1287.966552734375 |
| node_32: feature_name=ENST00000491977 | feature_id[29].value <= threshold=22.76432514190674 |
| node_33: feature_name=ENST00000611977 | feature_id[43].value > threshold=0.45197004079818726 |
| node_35: feature_name=ENST00000503771 | feature_id[193].value <= threshold=320.5686798095703 |
| node_36: feature_name=ENST00000307407 | feature_id[281].value > threshold=4.887194871902466 |
| node_38: feature_name=ENST00000321935 | feature_id[238].value > threshold=22.123645782470703 |
| node_40: feature_name=ENST00000492167 | feature_id[111].value <= threshold=833.0198974609375 |
| node_41: feature_name=ENST00000005178 | feature_id[118].value > threshold=29.375121116638184 |
| node_43: feature_name=ENST00000411764 | feature_id[20].value <= threshold=186.49674224853516 |
| node_44: feature_name=ENST00000395002 | feature_id[256].value > threshold=13.3489990234375 |
| node_48: feature_name=ENST00000636279 | feature_id[103].value <= threshold=9.28081226348877 |
| node_49: feature_name=ENST00000244174 | feature_id[34].value <= threshold=10.21084976196289 |
| node_50: feature_name=ENST00000393203 | feature_id[91].value > threshold=21.360187530517578 |
| node_56: feature_name=ENST00000286732 | feature_id[131].value > threshold=1.1582629084587097 |
| node_58: feature_name=ENST00000643697 | feature_id[132].value <= threshold=12.256080150604248 |
| node_59: feature_name=ENST00000390606 | feature_id[293].value <= threshold=15.513253211975098 |
| Class: former_smokers | |
| | |
| Rules_6 | passed counts:37 |
| node_0: feature_name=ENST00000284311 | feature_id[0].value > threshold=179.44091796875 |
| node_92: feature_name=ENST00000396276 | feature_id[298].value <= threshold=957.0642700195312 |
| node_93: feature_name=ENST00000316418 | feature_id[181].value <= threshold=3.5686380863189697 |
| node_94: feature_name=ENST00000312143 | feature_id[46].value <= threshold=80.9970703125 |
| node_95: feature_name=ENST00000610349 | feature_id[275].value > threshold=6.537376165390015 |

| | |
|---|---|
| node_97: feature_name=ENST00000423064 | feature_id[240].value <= threshold=22.101216316223145 |
| node_98: feature_name=ENST00000613640 | feature_id[250].value <= threshold=205.0992202758789 |
| Class: current_smokers | |
| | |
| Rules_7 | passed counts:20 |
| node_0: feature_name=ENST00000284311 | feature_id[0].value > threshold=179.44091796875 |
| node_92: feature_name=ENST00000396276 | feature_id[298].value <= threshold=957.0642700195312 |
| node_93: feature_name=ENST00000316418 | feature_id[181].value > threshold=3.5686380863189697 |
| node_111: feature_name=ENST00000309575 | feature_id[257].value > threshold=375.328125 |
| node_143: feature_name=ENST00000296029 | feature_id[18].value <= threshold=753.8509216308594 |
| node_144: feature_name=ENST00000548358 | feature_id[115].value > threshold=17.743536949157715 |
| node_146: feature_name=ENST00000316623 | feature_id[58].value <= threshold=36.71030235290527 |
| Class: current_smokers | |
| | |
| Rules_8 | passed counts:18 |
| node_0: feature_name=ENST00000284311 | feature_id[0].value > threshold=179.44091796875 |
| node_92: feature_name=ENST00000396276 | feature_id[298].value <= threshold=957.0642700195312 |
| node_93: feature_name=ENST00000316418 | feature_id[181].value <= threshold=3.5686380863189697 |
| node_94: feature_name=ENST00000312143 | feature_id[46].value > threshold=80.9970703125 |
| node_102: feature_name=ENST00000507007 | feature_id[177].value > threshold=8.112542390823364 |
| node_108: feature_name=ENST00000261651 | feature_id[151].value > threshold=0.5338447093963623 |
| Class: former_smokers | |
| | |
| Rules_9 | passed counts:16 |
| node_0: feature_name=ENST00000284311 | feature_id[0].value <= threshold=179.44091796875 |
| node_1: feature_name=ENST00000390539 | feature_id[222].value > threshold=11.91484260559082 |

| | |
|---|---|
| node_81: feature_name=ENST00000297785 | feature_id[83].value <= threshold=280.75682067871094 |
| node_82: feature_name=ENST00000536374 | feature_id[276].value <= threshold=36.55038642883301 |
| Class: current_smokers | |
| | |
| Rules_10 | passed counts:15 |
| node_0: feature_name=ENST00000284311 | feature_id[0].value <= threshold=179.44091796875 |
| node_1: feature_name=ENST00000390539 | feature_id[222].value > threshold=11.91484260559082 |
| node_81: feature_name=ENST00000297785 | feature_id[83].value > threshold=280.75682067871094 |
| Class: former_smokers | |
| | |
| Rules_11 | passed counts:15 |
| node_0: feature_name=ENST00000284311 | feature_id[0].value <= threshold=179.44091796875 |
| node_1: feature_name=ENST00000390539 | feature_id[222].value > threshold=11.91484260559082 |
| node_81: feature_name=ENST00000297785 | feature_id[83].value <= threshold=280.75682067871094 |
| node_82: feature_name=ENST00000536374 | feature_id[276].value > threshold=36.55038642883301 |
| node_84: feature_name=ENST00000573760 | feature_id[84].value > threshold=11.246893405914307 |
| node_86: feature_name=ENST00000598234 | feature_id[119].value > threshold=185.54674530029297 |
| node_88: feature_name=ENST00000633685 | feature_id[201].value <= threshold=52.94426918029785 |
| Class: current_smokers | |
| | |
| Rules_12 | passed counts:14 |
| node_0: feature_name=ENST00000284311 | feature_id[0].value > threshold=179.44091796875 |
| node_92: feature_name=ENST00000396276 | feature_id[298].value > threshold=957.0642700195312 |
| node_150: feature_name=ENST00000284311 | feature_id[0].value > threshold=364.31639099121094 |
| node_170: feature_name=ENST00000339223 | feature_id[10].value <= threshold=33.90394592285156 |
| node_171: feature_name=ENST00000297785 | feature_id[83].value > threshold=208.11764526367188 |

| | |
|---|---|
| node_173: feature_name=ENST00000529814 | feature_id[209].value <= threshold=149.9900665283203 |
| Class: former_smokers | |
| | |
| Rules_13 | passed counts:14 |
| node_0: feature_name=ENST00000284311 | feature_id[0].value <= threshold=179.44091796875 |
| node_1: feature_name=ENST00000390539 | feature_id[222].value <= threshold=11.91484260559082 |
| node_2: feature_name=ENST00000586582 | feature_id[123].value <= threshold=17.368224143981934 |
| node_3: feature_name=ENST00000284984 | feature_id[292].value <= threshold=11.201291561126709 |
| node_4: feature_name=ENST00000586582 | feature_id[123].value > threshold=5.24660587310791 |
| node_18: feature_name=ENST00000611977 | feature_id[43].value <= threshold=12.043715476989746 |
| node_19: feature_name=ENST00000483158 | feature_id[197].value > threshold=6.121511459350586 |
| Class: current_smokers | |
| | |
| Rules_14 | passed counts:13 |
| node_0: feature_name=ENST00000284311 | feature_id[0].value > threshold=179.44091796875 |
| node_92: feature_name=ENST00000396276 | feature_id[298].value > threshold=957.0642700195312 |
| node_150: feature_name=ENST00000284311 | feature_id[0].value <= threshold=364.31639099121094 |
| node_151: feature_name=ENST00000276974 | feature_id[6].value > threshold=1.20855313539505 |
| node_161: feature_name=ENST00000641136 | feature_id[30].value > threshold=5.980504512786865 |
| node_165: feature_name=ENST00000396618 | feature_id[106].value <= threshold=42.30185317993164 |
| Class: current_smokers | |
| | |
| Rules_15 | passed counts:12 |
| node_0: feature_name=ENST00000284311 | feature_id[0].value <= threshold=179.44091796875 |
| node_1: feature_name=ENST00000390539 | feature_id[222].value <= threshold=11.91484260559082 |
| node_2: feature_name=ENST00000586582 | feature_id[123].value > threshold=17.368224143981934 |

| | |
|---|---|
| node_78: feature_name=ENST00000324907 | feature_id[8].value <= threshold=9.99325180053711 |
| Class: current_smokers | |
| | |
| Rules_16 | passed counts:11 |
| node_0: feature_name=ENST00000284311 | feature_id[0].value <= threshold=179.44091796875 |
| node_1: feature_name=ENST00000390539 | feature_id[222].value > threshold=11.91484260559082 |
| node_81: feature_name=ENST00000297785 | feature_id[83].value <= threshold=280.75682067871094 |
| node_82: feature_name=ENST00000536374 | feature_id[276].value > threshold=36.55038642883301 |
| node_84: feature_name=ENST00000573760 | feature_id[84].value <= threshold=11.246893405914307 |
| Class: former_smokers | |
| | |
| Rules_17 | passed counts:10 |
| node_0: feature_name=ENST00000284311 | feature_id[0].value <= threshold=179.44091796875 |
| node_1: feature_name=ENST00000390539 | feature_id[222].value <= threshold=11.91484260559082 |
| node_2: feature_name=ENST00000586582 | feature_id[123].value <= threshold=17.368224143981934 |
| node_3: feature_name=ENST00000284984 | feature_id[292].value <= threshold=11.201291561126709 |
| node_4: feature_name=ENST00000586582 | feature_id[123].value <= threshold=5.24660587310791 |
| node_5: feature_name=ENST00000244174 | feature_id[34].value > threshold=2.29075288772583 |
| node_13: feature_name=ENST00000284311 | feature_id[0].value > threshold=71.21495056152344 |
| node_15: feature_name=ENST00000377712 | feature_id[94].value > threshold=0.4905405640602112 |
| Class: current_smokers | |
| | |
| Rules_18 | passed counts:9 |
| node_0: feature_name=ENST00000284311 | feature_id[0].value > threshold=179.44091796875 |
| node_92: feature_name=ENST00000396276 | feature_id[298].value > threshold=957.0642700195312 |
| node_150: feature_name=ENST00000284311 | feature_id[0].value <= threshold=364.31639099121094 |

| | |
|---|---|
| node_151: feature_name=ENST00000276974 | feature_id[6].value > threshold=1.20855313539505 |
| node_161: feature_name=ENST00000641136 | feature_id[30].value <= threshold=5.980504512786865 |
| node_162: feature_name=ENST00000430223 | feature_id[183].value > threshold=155.0065689086914 |
| Class: former_smokers | |
| | |
| Rules_19 | passed counts:9 |
| node_0: feature_name=ENST00000284311 | feature_id[0].value <= threshold=179.44091796875 |
| node_1: feature_name=ENST00000390539 | feature_id[222].value <= threshold=11.91484260559082 |
| node_2: feature_name=ENST00000586582 | feature_id[123].value <= threshold=17.368224143981934 |
| node_3: feature_name=ENST00000284984 | feature_id[292].value > threshold=11.201291561126709 |
| node_23: feature_name=ENST00000276974 | feature_id[6].value <= threshold=75.58467483520508 |
| node_24: feature_name=ENST00000464302 | feature_id[285].value > threshold=25.290170669555664 |
| node_28: feature_name=ENST00000650242 | feature_id[234].value > threshold=1287.966552734375 |
| node_32: feature_name=ENST00000491977 | feature_id[29].value <= threshold=22.76432514190674 |
| node_33: feature_name=ENST00000611977 | feature_id[43].value > threshold=0.45197004079818726 |
| node_35: feature_name=ENST00000503771 | feature_id[193].value <= threshold=320.5686798095703 |
| node_36: feature_name=ENST00000307407 | feature_id[281].value > threshold=4.887194871902466 |
| node_38: feature_name=ENST00000321935 | feature_id[238].value > threshold=22.123645782470703 |
| node_40: feature_name=ENST00000492167 | feature_id[111].value <= threshold=833.0198974609375 |
| node_41: feature_name=ENST00000005178 | feature_id[118].value > threshold=29.375121116638184 |
| node_43: feature_name=ENST00000411764 | feature_id[20].value <= threshold=186.49674224853516 |
| node_44: feature_name=ENST00000395002 | feature_id[256].value > threshold=13.3489990234375 |
| node_48: feature_name=ENST00000636279 | feature_id[103].value <= threshold=9.28081226348877 |
| node_49: feature_name=ENST00000244174 | feature_id[34].value > threshold=10.21084976196289 |
| node_63: feature_name=ENST00000558197 | feature_id[4].value > threshold=2.4969643354415894 |

| | |
|---|---|
| Class: former_smokers | |
| | |
| Rules_20 | passed counts:7 |
| node_0: feature_name=ENST00000284311 | feature_id[0].value > threshold=179.44091796875 |
| node_92: feature_name=ENST00000396276 | feature_id[298].value <= threshold=957.0642700195312 |
| node_93: feature_name=ENST00000316418 | feature_id[181].value <= threshold=3.5686380863189697 |
| node_94: feature_name=ENST00000312143 | feature_id[46].value > threshold=80.9970703125 |
| node_102: feature_name=ENST00000507007 | feature_id[177].value <= threshold=8.112542390823364 |
| node_103: feature_name=ENST00000367467 | feature_id[107].value > threshold=39.29389572143555 |
| Class: current_smokers | |
| | |
| Rules_21 | passed counts:6 |
| node_0: feature_name=ENST00000284311 | feature_id[0].value > threshold=179.44091796875 |
| node_92: feature_name=ENST00000396276 | feature_id[298].value <= threshold=957.0642700195312 |
| node_93: feature_name=ENST00000316418 | feature_id[181].value > threshold=3.5686380863189697 |
| node_111: feature_name=ENST00000309575 | feature_id[257].value > threshold=375.328125 |
| node_143: feature_name=ENST00000296029 | feature_id[18].value > threshold=753.8509216308594 |
| Class: former_smokers | |
| | |
| Rules_22 | passed counts:6 |
| node_0: feature_name=ENST00000284311 | feature_id[0].value <= threshold=179.44091796875 |
| node_1: feature_name=ENST00000390539 | feature_id[222].value <= threshold=11.91484260559082 |
| node_2: feature_name=ENST00000586582 | feature_id[123].value <= threshold=17.368224143981934 |
| node_3: feature_name=ENST00000284984 | feature_id[292].value <= threshold=11.201291561126709 |
| node_4: feature_name=ENST00000586582 | feature_id[123].value <= threshold=5.24660587310791 |
| node_5: feature_name=ENST00000244174 | feature_id[34].value > threshold=2.29075288772583 |

| | |
|---|---|
| node_13: feature_name=ENST00000284311 | feature_id[0].value <= threshold=71.21495056152344 |
| Class: former_smokers | |
| | |
| Rules_23 | passed counts:5 |
| node_0: feature_name=ENST00000284311 | feature_id[0].value > threshold=179.44091796875 |
| node_92: feature_name=ENST00000396276 | feature_id[298].value > threshold=957.0642700195312 |
| node_150: feature_name=ENST00000284311 | feature_id[0].value > threshold=364.31639099121094 |
| node_170: feature_name=ENST00000339223 | feature_id[10].value <= threshold=33.90394592285156 |
| node_171: feature_name=ENST00000297785 | feature_id[83].value <= threshold=208.11764526367188 |
| Class: current_smokers | |
| | |
| Rules_24 | passed counts:5 |
| node_0: feature_name=ENST00000284311 | feature_id[0].value > threshold=179.44091796875 |
| node_92: feature_name=ENST00000396276 | feature_id[298].value <= threshold=957.0642700195312 |
| node_93: feature_name=ENST00000316418 | feature_id[181].value > threshold=3.5686380863189697 |
| node_111: feature_name=ENST00000309575 | feature_id[257].value <= threshold=375.328125 |
| node_112: feature_name=ENST00000367467 | feature_id[107].value > threshold=21.34785747528076 |
| node_116: feature_name=ENST00000571489 | feature_id[279].value <= threshold=56.20589256286621 |
| node_117: feature_name=ENST00000635923 | feature_id[171].value > threshold=328.1167755126953 |
| node_137: feature_name=ENST00000507411 | feature_id[82].value > threshold=57.23691940307617 |
| Class: current_smokers | |
| | |
| Rules_25 | passed counts:5 |
| node_0: feature_name=ENST00000284311 | feature_id[0].value > threshold=179.44091796875 |
| node_92: feature_name=ENST00000396276 | feature_id[298].value <= threshold=957.0642700195312 |
| node_93: feature_name=ENST00000316418 | feature_id[181].value <= threshold=3.5686380863189697 |

| | |
|---|---|
| node_94: feature_name=ENST00000312143 | feature_id[46].value > threshold=80.9970703125 |
| node_102: feature_name=ENST00000507007 | feature_id[177].value <= threshold=8.112542390823364 |
| node_103: feature_name=ENST00000367467 | feature_id[107].value <= threshold=39.29389572143555 |
| node_104: feature_name=ENST00000308478 | feature_id[2].value <= threshold=83.28301239013672 |
| Class: former_smokers | |
| | |
| Rules_26 | passed counts:5 |
| node_0: feature_name=ENST00000284311 | feature_id[0].value > threshold=179.44091796875 |
| node_92: feature_name=ENST00000396276 | feature_id[298].value <= threshold=957.0642700195312 |
| node_93: feature_name=ENST00000316418 | feature_id[181].value <= threshold=3.5686380863189697 |
| node_94: feature_name=ENST00000312143 | feature_id[46].value <= threshold=80.9970703125 |
| node_95: feature_name=ENST00000610349 | feature_id[275].value <= threshold=6.537376165390015 |
| Class: former_smokers | |
| | |
| Rules_27 | passed counts:5 |
| node_0: feature_name=ENST00000284311 | feature_id[0].value <= threshold=179.44091796875 |
| node_1: feature_name=ENST00000390539 | feature_id[222].value > threshold=11.91484260559082 |
| node_81: feature_name=ENST00000297785 | feature_id[83].value <= threshold=280.75682067871094 |
| node_82: feature_name=ENST00000536374 | feature_id[276].value > threshold=36.55038642883301 |
| node_84: feature_name=ENST00000573760 | feature_id[84].value > threshold=11.246893405914307 |
| node_86: feature_name=ENST00000598234 | feature_id[119].value <= threshold=185.54674530029297 |
| Class: former_smokers | |
| | |
| Rules_28 | passed counts:5 |
| node_0: feature_name=ENST00000284311 | feature_id[0].value <= threshold=179.44091796875 |
| node_1: feature_name=ENST00000390539 | feature_id[222].value <= threshold=11.91484260559082 |

| | |
|---|---|
| node_2: feature_name=ENST00000586582 | feature_id[123].value <= threshold=17.368224143981934 |
| node_3: feature_name=ENST00000284984 | feature_id[292].value <= threshold=11.201291561126709 |
| node_4: feature_name=ENST00000586582 | feature_id[123].value > threshold=5.24660587310791 |
| node_18: feature_name=ENST00000611977 | feature_id[43].value > threshold=12.043715476989746 |
| Class: former_smokers | |
| | |
| Rules_29 | passed counts:4 |
| node_0: feature_name=ENST00000284311 | feature_id[0].value > threshold=179.44091796875 |
| node_92: feature_name=ENST00000396276 | feature_id[298].value > threshold=957.0642700195312 |
| node_150: feature_name=ENST00000284311 | feature_id[0].value <= threshold=364.31639099121094 |
| node_151: feature_name=ENST00000276974 | feature_id[6].value <= threshold=1.20855313539505 |
| node_152: feature_name=ENST00000441556 | feature_id[147].value > threshold=8.403407096862793 |
| node_158: feature_name=ENST00000464835 | feature_id[129].value <= threshold=14.235955238342285 |
| Class: current_smokers | |
| | |
| Rules_30 | passed counts:4 |
| node_0: feature_name=ENST00000284311 | feature_id[0].value > threshold=179.44091796875 |
| node_92: feature_name=ENST00000396276 | feature_id[298].value <= threshold=957.0642700195312 |
| node_93: feature_name=ENST00000316418 | feature_id[181].value > threshold=3.5686380863189697 |
| node_111: feature_name=ENST00000309575 | feature_id[257].value > threshold=375.328125 |
| node_143: feature_name=ENST00000296029 | feature_id[18].value <= threshold=753.8509216308594 |
| node_144: feature_name=ENST00000548358 | feature_id[115].value <= threshold=17.743536949157715 |
| Class: former_smokers | |
| | |
| Rules_31 | passed counts:4 |
| node_0: feature_name=ENST00000284311 | feature_id[0].value > threshold=179.44091796875 |

| | |
|---|---|
| node_92: feature_name=ENST00000396276 | feature_id[298].value <= threshold=957.0642700195312 |
| node_93: feature_name=ENST00000316418 | feature_id[181].value > threshold=3.5686380863189697 |
| node_111: feature_name=ENST00000309575 | feature_id[257].value <= threshold=375.328125 |
| node_112: feature_name=ENST00000367467 | feature_id[107].value <= threshold=21.34785747528076 |
| node_113: feature_name=ENST00000390285 | feature_id[25].value <= threshold=7.0215864181518555 |
| Class: former_smokers | |
| | |
| Rules_32 | passed counts:4 |
| node_0: feature_name=ENST00000284311 | feature_id[0].value <= threshold=179.44091796875 |
| node_1: feature_name=ENST00000390539 | feature_id[222].value <= threshold=11.91484260559082 |
| node_2: feature_name=ENST00000586582 | feature_id[123].value > threshold=17.368224143981934 |
| node_78: feature_name=ENST00000324907 | feature_id[8].value > threshold=9.99325180053711 |
| Class: former_smokers | |
| | |
| Rules_33 | passed counts:3 |
| node_0: feature_name=ENST00000284311 | feature_id[0].value > threshold=179.44091796875 |
| node_92: feature_name=ENST00000396276 | feature_id[298].value > threshold=957.0642700195312 |
| node_150: feature_name=ENST00000284311 | feature_id[0].value > threshold=364.31639099121094 |
| node_170: feature_name=ENST00000339223 | feature_id[10].value > threshold=33.90394592285156 |
| node_176: feature_name=ENST00000513886 | feature_id[39].value > threshold=141.72513961791992 |
| Class: former_smokers | |
| | |
| Rules_34 | passed counts:3 |
| node_0: feature_name=ENST00000284311 | feature_id[0].value > threshold=179.44091796875 |
| node_92: feature_name=ENST00000396276 | feature_id[298].value <= threshold=957.0642700195312 |
| node_93: feature_name=ENST00000316418 | feature_id[181].value > threshold=3.5686380863189697 |

| | |
|---|---|
| node_111: feature_name=ENST00000309575 | feature_id[257].value <= threshold=375.328125 |
| node_112: feature_name=ENST00000367467 | feature_id[107].value > threshold=21.34785747528076 |
| node_116: feature_name=ENST00000571489 | feature_id[279].value <= threshold=56.20589256286621 |
| node_117: feature_name=ENST00000635923 | feature_id[171].value > threshold=328.1167755126953 |
| node_137: feature_name=ENST00000507411 | feature_id[82].value <= threshold=57.23691940307617 |
| Class: former_smokers | |
| | |
| Rules_35 | passed counts:3 |
| node_0: feature_name=ENST00000284311 | feature_id[0].value > threshold=179.44091796875 |
| node_92: feature_name=ENST00000396276 | feature_id[298].value <= threshold=957.0642700195312 |
| node_93: feature_name=ENST00000316418 | feature_id[181].value > threshold=3.5686380863189697 |
| node_111: feature_name=ENST00000309575 | feature_id[257].value <= threshold=375.328125 |
| node_112: feature_name=ENST00000367467 | feature_id[107].value > threshold=21.34785747528076 |
| node_116: feature_name=ENST00000571489 | feature_id[279].value <= threshold=56.20589256286621 |
| node_117: feature_name=ENST00000635923 | feature_id[171].value <= threshold=328.1167755126953 |
| node_118: feature_name=ENST00000334529 | feature_id[148].value > threshold=60.1170597076416 |
| node_120: feature_name=ENST00000635923 | feature_id[171].value > threshold=37.20250701904297 |
| node_124: feature_name=ENST00000498146 | feature_id[143].value > threshold=0.441722571849823 |
| node_128: feature_name=ENST00000260526 | feature_id[236].value <= threshold=21.659213066101074 |
| node_129: feature_name=ENST00000390325 | feature_id[17].value <= threshold=17.23688793182373 |
| node_130: feature_name=ENST00000573760 | feature_id[84].value > threshold=10.882764339447021 |
| Class: current_smokers | |
| | |
| Rules_36 | passed counts:3 |
| node_0: feature_name=ENST00000284311 | feature_id[0].value > threshold=179.44091796875 |
| node_92: feature_name=ENST00000396276 | feature_id[298].value <= threshold=957.0642700195312 |

| | |
|---|---|
| node_93: feature_name=ENST00000316418 | feature_id[181].value > threshold=3.5686380863189697 |
| node_111: feature_name=ENST00000309575 | feature_id[257].value <= threshold=375.328125 |
| node_112: feature_name=ENST00000367467 | feature_id[107].value <= threshold=21.34785747528076 |
| node_113: feature_name=ENST00000390285 | feature_id[25].value > threshold=7.0215864181518555 |
| Class: current_smokers | |
| | |
| Rules_37 | passed counts:3 |
| node_0: feature_name=ENST00000284311 | feature_id[0].value <= threshold=179.44091796875 |
| node_1: feature_name=ENST00000390539 | feature_id[222].value > threshold=11.91484260559082 |
| node_81: feature_name=ENST00000297785 | feature_id[83].value <= threshold=280.75682067871094 |
| node_82: feature_name=ENST00000536374 | feature_id[276].value > threshold=36.55038642883301 |
| node_84: feature_name=ENST00000573760 | feature_id[84].value > threshold=11.246893405914307 |
| node_86: feature_name=ENST00000598234 | feature_id[119].value > threshold=185.54674530029297 |
| node_88: feature_name=ENST00000633685 | feature_id[201].value > threshold=52.94426918029785 |
| Class: former_smokers | |
| | |
| Rules_38 | passed counts:3 |
| node_0: feature_name=ENST00000284311 | feature_id[0].value <= threshold=179.44091796875 |
| node_1: feature_name=ENST00000390539 | feature_id[222].value <= threshold=11.91484260559082 |
| node_2: feature_name=ENST00000586582 | feature_id[123].value <= threshold=17.368224143981934 |
| node_3: feature_name=ENST00000284984 | feature_id[292].value > threshold=11.201291561126709 |
| node_23: feature_name=ENST00000276974 | feature_id[6].value > threshold=75.58467483520508 |
| node_75: feature_name=ENST00000415351 | feature_id[216].value <= threshold=3.5765366554260254 |
| Class: current_smokers | |
| | |
| Rules_39 | passed counts:3 |

| | |
|---|---|
| node_0: feature_name=ENST00000284311 | feature_id[0].value <= threshold=179.44091796875 |
| node_1: feature_name=ENST00000390539 | feature_id[222].value <= threshold=11.91484260559082 |
| node_2: feature_name=ENST00000586582 | feature_id[123].value <= threshold=17.368224143981934 |
| node_3: feature_name=ENST00000284984 | feature_id[292].value > threshold=11.201291561126709 |
| node_23: feature_name=ENST00000276974 | feature_id[6].value <= threshold=75.58467483520508 |
| node_24: feature_name=ENST00000464302 | feature_id[285].value > threshold=25.290170669555664 |
| node_28: feature_name=ENST00000650242 | feature_id[234].value > threshold=1287.966552734375 |
| node_32: feature_name=ENST00000491977 | feature_id[29].value > threshold=22.76432514190674 |
| node_72: feature_name=ENST00000610261 | feature_id[260].value > threshold=57.027976989746094 |
| Class: former_smokers | |
| | |
| Rules_40 | passed counts:3 |
| node_0: feature_name=ENST00000284311 | feature_id[0].value <= threshold=179.44091796875 |
| node_1: feature_name=ENST00000390539 | feature_id[222].value <= threshold=11.91484260559082 |
| node_2: feature_name=ENST00000586582 | feature_id[123].value <= threshold=17.368224143981934 |
| node_3: feature_name=ENST00000284984 | feature_id[292].value > threshold=11.201291561126709 |
| node_23: feature_name=ENST00000276974 | feature_id[6].value <= threshold=75.58467483520508 |
| node_24: feature_name=ENST00000464302 | feature_id[285].value > threshold=25.290170669555664 |
| node_28: feature_name=ENST00000650242 | feature_id[234].value > threshold=1287.966552734375 |
| node_32: feature_name=ENST00000491977 | feature_id[29].value > threshold=22.76432514190674 |
| node_72: feature_name=ENST00000610261 | feature_id[260].value <= threshold=57.027976989746094 |
| Class: current_smokers | |
| | |
| Rules_41 | passed counts:3 |
| node_0: feature_name=ENST00000284311 | feature_id[0].value <= threshold=179.44091796875 |
| node_1: feature_name=ENST00000390539 | feature_id[222].value <= threshold=11.91484260559082 |

| | |
|---|---|
| node_2: feature_name=ENST00000586582 | feature_id[123].value <= threshold=17.368224143981934 |
| node_3: feature_name=ENST00000284984 | feature_id[292].value > threshold=11.201291561126709 |
| node_23: feature_name=ENST00000276974 | feature_id[6].value <= threshold=75.58467483520508 |
| node_24: feature_name=ENST00000464302 | feature_id[285].value > threshold=25.290170669555664 |
| node_28: feature_name=ENST00000650242 | feature_id[234].value > threshold=1287.966552734375 |
| node_32: feature_name=ENST00000491977 | feature_id[29].value <= threshold=22.76432514190674 |
| node_33: feature_name=ENST00000611977 | feature_id[43].value > threshold=0.45197004079818726 |
| node_35: feature_name=ENST00000503771 | feature_id[193].value <= threshold=320.5686798095703 |
| node_36: feature_name=ENST00000307407 | feature_id[281].value > threshold=4.887194871902466 |
| node_38: feature_name=ENST00000321935 | feature_id[238].value > threshold=22.123645782470703 |
| node_40: feature_name=ENST00000492167 | feature_id[111].value <= threshold=833.0198974609375 |
| node_41: feature_name=ENST00000005178 | feature_id[118].value > threshold=29.375121116638184 |
| node_43: feature_name=ENST00000411764 | feature_id[20].value <= threshold=186.49674224853516 |
| node_44: feature_name=ENST00000395002 | feature_id[256].value > threshold=13.3489990234375 |
| node_48: feature_name=ENST00000636279 | feature_id[103].value <= threshold=9.28081226348877 |
| node_49: feature_name=ENST00000244174 | feature_id[34].value <= threshold=10.21084976196289 |
| node_50: feature_name=ENST00000393203 | feature_id[91].value <= threshold=21.360187530517578 |
| node_51: feature_name=ENST00000278919 | feature_id[121].value > threshold=45.06623077392578 |
| node_53: feature_name=ENST00000607161 | feature_id[195].value > threshold=7.280014753341675 |
| Class: former_smokers | |
| | |
| Rules_42 | passed counts:3 |
| node_0: feature_name=ENST00000284311 | feature_id[0].value <= threshold=179.44091796875 |
| node_1: feature_name=ENST00000390539 | feature_id[222].value <= threshold=11.91484260559082 |
| node_2: feature_name=ENST00000586582 | feature_id[123].value <= threshold=17.368224143981934 |

| | |
|---|---|
| node_3: feature_name=ENST00000284984 | feature_id[292].value > threshold=11.201291561126709 |
| node_23: feature_name=ENST00000276974 | feature_id[6].value <= threshold=75.58467483520508 |
| node_24: feature_name=ENST00000464302 | feature_id[285].value > threshold=25.290170669555664 |
| node_28: feature_name=ENST00000650242 | feature_id[234].value <= threshold=1287.966552734375 |
| node_29: feature_name=ENST00000375448 | feature_id[68].value > threshold=524.4175415039062 |
| Class: current_smokers | |
| | |
| Rules_43 | passed counts:3 |
| node_0: feature_name=ENST00000284311 | feature_id[0].value <= threshold=179.44091796875 |
| node_1: feature_name=ENST00000390539 | feature_id[222].value <= threshold=11.91484260559082 |
| node_2: feature_name=ENST00000586582 | feature_id[123].value <= threshold=17.368224143981934 |
| node_3: feature_name=ENST00000284984 | feature_id[292].value > threshold=11.201291561126709 |
| node_23: feature_name=ENST00000276974 | feature_id[6].value <= threshold=75.58467483520508 |
| node_24: feature_name=ENST00000464302 | feature_id[285].value <= threshold=25.290170669555664 |
| node_25: feature_name=ENST00000390305 | feature_id[272].value > threshold=11.122980833053589 |
| Class: current_smokers | |
| | |
| Rules_44 | passed counts:2 |
| node_0: feature_name=ENST00000284311 | feature_id[0].value > threshold=179.44091796875 |
| node_92: feature_name=ENST00000396276 | feature_id[298].value > threshold=957.0642700195312 |
| node_150: feature_name=ENST00000284311 | feature_id[0].value <= threshold=364.31639099121094 |
| node_151: feature_name=ENST00000276974 | feature_id[6].value > threshold=1.20855313539505 |
| node_161: feature_name=ENST00000641136 | feature_id[30].value > threshold=5.980504512786865 |
| node_165: feature_name=ENST00000396618 | feature_id[106].value > threshold=42.30185317993164 |
| node_167: feature_name=ENST00000548358 | feature_id[115].value <= threshold=34.7273063659668 |
| Class: former_smokers | |

| | |
|---|---|
| Rules_45 | passed counts:2 |
| node_0: feature_name=ENST00000284311 | feature_id[0].value > threshold=179.44091796875 |
| node_92: feature_name=ENST00000396276 | feature_id[298].value > threshold=957.0642700195312 |
| node_150: feature_name=ENST00000284311 | feature_id[0].value <= threshold=364.31639099121094 |
| node_151: feature_name=ENST00000276974 | feature_id[6].value > threshold=1.20855313539505 |
| node_161: feature_name=ENST00000641136 | feature_id[30].value <= threshold=5.980504512786865 |
| node_162: feature_name=ENST00000430223 | feature_id[183].value <= threshold=155.0065689086914 |
| Class: current_smokers | |
| | |
| Rules_46 | passed counts:2 |
| node_0: feature_name=ENST00000284311 | feature_id[0].value > threshold=179.44091796875 |
| node_92: feature_name=ENST00000396276 | feature_id[298].value > threshold=957.0642700195312 |
| node_150: feature_name=ENST00000284311 | feature_id[0].value <= threshold=364.31639099121094 |
| node_151: feature_name=ENST00000276974 | feature_id[6].value <= threshold=1.20855313539505 |
| node_152: feature_name=ENST00000441556 | feature_id[147].value > threshold=8.403407096862793 |
| node_158: feature_name=ENST00000464835 | feature_id[129].value > threshold=14.235955238342285 |
| Class: former_smokers | |
| | |
| Rules_47 | passed counts:2 |
| node_0: feature_name=ENST00000284311 | feature_id[0].value > threshold=179.44091796875 |
| node_92: feature_name=ENST00000396276 | feature_id[298].value > threshold=957.0642700195312 |
| node_150: feature_name=ENST00000284311 | feature_id[0].value <= threshold=364.31639099121094 |
| node_151: feature_name=ENST00000276974 | feature_id[6].value <= threshold=1.20855313539505 |
| node_152: feature_name=ENST00000441556 | feature_id[147].value <= threshold=8.403407096862793 |
| node_153: feature_name=ENST00000390304 | feature_id[81].value > threshold=9.798711776733398 |

| | |
|---|---|
| Class: current_smokers | |
| | |
| Rules_48 | passed counts:2 |
| node_0: feature_name=ENST00000284311 | feature_id[0].value > threshold=179.44091796875 |
| node_92: feature_name=ENST00000396276 | feature_id[298].value <= threshold=957.0642700195312 |
| node_93: feature_name=ENST00000316418 | feature_id[181].value > threshold=3.5686380863189697 |
| node_111: feature_name=ENST00000309575 | feature_id[257].value > threshold=375.328125 |
| node_143: feature_name=ENST00000296029 | feature_id[18].value <= threshold=753.8509216308594 |
| node_144: feature_name=ENST00000548358 | feature_id[115].value > threshold=17.743536949157715 |
| node_146: feature_name=ENST00000316623 | feature_id[58].value > threshold=36.71030235290527 |
| Class: former_smokers | |
| | |
| Rules_49 | passed counts:2 |
| node_0: feature_name=ENST00000284311 | feature_id[0].value > threshold=179.44091796875 |
| node_92: feature_name=ENST00000396276 | feature_id[298].value <= threshold=957.0642700195312 |
| node_93: feature_name=ENST00000316418 | feature_id[181].value > threshold=3.5686380863189697 |
| node_111: feature_name=ENST00000309575 | feature_id[257].value <= threshold=375.328125 |
| node_112: feature_name=ENST00000367467 | feature_id[107].value > threshold=21.34785747528076 |
| node_116: feature_name=ENST00000571489 | feature_id[279].value > threshold=56.20589256286621 |
| node_140: feature_name=ENST00000529814 | feature_id[209].value > threshold=103.67559623718262 |
| Class: former_smokers | |
| | |
| Rules_50 | passed counts:2 |
| node_0: feature_name=ENST00000284311 | feature_id[0].value > threshold=179.44091796875 |
| node_92: feature_name=ENST00000396276 | feature_id[298].value <= threshold=957.0642700195312 |
| node_93: feature_name=ENST00000316418 | feature_id[181].value <= threshold=3.5686380863189697 |

| | |
|---|---|
| node_94: feature_name=ENST00000312143 | feature_id[46].value > threshold=80.9970703125 |
| node_102: feature_name=ENST00000507007 | feature_id[177].value <= threshold=8.112542390823364 |
| node_103: feature_name=ENST00000367467 | feature_id[107].value <= threshold=39.29389572143555 |
| node_104: feature_name=ENST00000308478 | feature_id[2].value > threshold=83.28301239013672 |
| Class: current_smokers | |
| | |
| Rules_51 | passed counts:2 |
| node_0: feature_name=ENST00000284311 | feature_id[0].value > threshold=179.44091796875 |
| node_92: feature_name=ENST00000396276 | feature_id[298].value <= threshold=957.0642700195312 |
| node_93: feature_name=ENST00000316418 | feature_id[181].value <= threshold=3.5686380863189697 |
| node_94: feature_name=ENST00000312143 | feature_id[46].value <= threshold=80.9970703125 |
| node_95: feature_name=ENST00000610349 | feature_id[275].value > threshold=6.537376165390015 |
| node_97: feature_name=ENST00000423064 | feature_id[240].value > threshold=22.101216316223145 |
| Class: former_smokers | |
| | |
| Rules_52 | passed counts:2 |
| node_0: feature_name=ENST00000284311 | feature_id[0].value <= threshold=179.44091796875 |
| node_1: feature_name=ENST00000390539 | feature_id[222].value <= threshold=11.91484260559082 |
| node_2: feature_name=ENST00000586582 | feature_id[123].value <= threshold=17.368224143981934 |
| node_3: feature_name=ENST00000284984 | feature_id[292].value > threshold=11.201291561126709 |
| node_23: feature_name=ENST00000276974 | feature_id[6].value <= threshold=75.58467483520508 |
| node_24: feature_name=ENST00000464302 | feature_id[285].value > threshold=25.290170669555664 |
| node_28: feature_name=ENST00000650242 | feature_id[234].value > threshold=1287.966552734375 |
| node_32: feature_name=ENST00000491977 | feature_id[29].value <= threshold=22.76432514190674 |
| node_33: feature_name=ENST00000611977 | feature_id[43].value > threshold=0.45197004079818726 |
| node_35: feature_name=ENST00000503771 | feature_id[193].value <= threshold=320.5686798095703 |

| | |
|---|---|
| node_36: feature_name=ENST00000307407 | feature_id[281].value > threshold=4.887194871902466 |
| node_38: feature_name=ENST00000321935 | feature_id[238].value > threshold=22.123645782470703 |
| node_40: feature_name=ENST00000492167 | feature_id[111].value <= threshold=833.0198974609375 |
| node_41: feature_name=ENST00000005178 | feature_id[118].value > threshold=29.375121116638184 |
| node_43: feature_name=ENST00000411764 | feature_id[20].value <= threshold=186.49674224853516 |
| node_44: feature_name=ENST00000395002 | feature_id[256].value > threshold=13.3489990234375 |
| node_48: feature_name=ENST00000636279 | feature_id[103].value <= threshold=9.28081226348877 |
| node_49: feature_name=ENST00000244174 | feature_id[34].value > threshold=10.21084976196289 |
| node_63: feature_name=ENST00000558197 | feature_id[4].value <= threshold=2.4969643354415894 |
| Class: current_smokers | |
| | |
| Rules_53 | passed counts:2 |
| node_0: feature_name=ENST00000284311 | feature_id[0].value <= threshold=179.44091796875 |
| node_1: feature_name=ENST00000390539 | feature_id[222].value <= threshold=11.91484260559082 |
| node_2: feature_name=ENST00000586582 | feature_id[123].value <= threshold=17.368224143981934 |
| node_3: feature_name=ENST00000284984 | feature_id[292].value > threshold=11.201291561126709 |
| node_23: feature_name=ENST00000276974 | feature_id[6].value <= threshold=75.58467483520508 |
| node_24: feature_name=ENST00000464302 | feature_id[285].value > threshold=25.290170669555664 |
| node_28: feature_name=ENST00000650242 | feature_id[234].value > threshold=1287.966552734375 |
| node_32: feature_name=ENST00000491977 | feature_id[29].value <= threshold=22.76432514190674 |
| node_33: feature_name=ENST00000611977 | feature_id[43].value > threshold=0.45197004079818726 |
| node_35: feature_name=ENST00000503771 | feature_id[193].value <= threshold=320.5686798095703 |
| node_36: feature_name=ENST00000307407 | feature_id[281].value > threshold=4.887194871902466 |
| node_38: feature_name=ENST00000321935 | feature_id[238].value > threshold=22.123645782470703 |
| node_40: feature_name=ENST00000492167 | feature_id[111].value <= threshold=833.0198974609375 |

| | |
|---|---|
| node_41: feature_name=ENST00000005178 | feature_id[118].value > threshold=29.375121116638184 |
| node_43: feature_name=ENST00000411764 | feature_id[20].value <= threshold=186.49674224853516 |
| node_44: feature_name=ENST00000395002 | feature_id[256].value > threshold=13.3489990234375 |
| node_48: feature_name=ENST00000636279 | feature_id[103].value <= threshold=9.28081226348877 |
| node_49: feature_name=ENST00000244174 | feature_id[34].value <= threshold=10.21084976196289 |
| node_50: feature_name=ENST00000393203 | feature_id[91].value > threshold=21.360187530517578 |
| node_56: feature_name=ENST00000286732 | feature_id[131].value <= threshold=1.1582629084587097 |
| Class: current_smokers | |
| | |
| Rules_54 | passed counts:2 |
| node_0: feature_name=ENST00000284311 | feature_id[0].value <= threshold=179.44091796875 |
| node_1: feature_name=ENST00000390539 | feature_id[222].value <= threshold=11.91484260559082 |
| node_2: feature_name=ENST00000586582 | feature_id[123].value <= threshold=17.368224143981934 |
| node_3: feature_name=ENST00000284984 | feature_id[292].value > threshold=11.201291561126709 |
| node_23: feature_name=ENST00000276974 | feature_id[6].value <= threshold=75.58467483520508 |
| node_24: feature_name=ENST00000464302 | feature_id[285].value > threshold=25.290170669555664 |
| node_28: feature_name=ENST00000650242 | feature_id[234].value <= threshold=1287.966552734375 |
| node_29: feature_name=ENST00000375448 | feature_id[68].value <= threshold=524.4175415039062 |
| Class: former_smokers | |
| | |
| Rules_55 | passed counts:2 |
| node_0: feature_name=ENST00000284311 | feature_id[0].value <= threshold=179.44091796875 |
| node_1: feature_name=ENST00000390539 | feature_id[222].value <= threshold=11.91484260559082 |
| node_2: feature_name=ENST00000586582 | feature_id[123].value <= threshold=17.368224143981934 |
| node_3: feature_name=ENST00000284984 | feature_id[292].value <= threshold=11.201291561126709 |
| node_4: feature_name=ENST00000586582 | feature_id[123].value <= threshold=5.24660587310791 |

| | |
|---|---|
| node_5: feature_name=ENST00000244174 | feature_id[34].value > threshold=2.29075288772583 |
| node_13: feature_name=ENST00000284311 | feature_id[0].value > threshold=71.21495056152344 |
| node_15: feature_name=ENST00000377712 | feature_id[94].value <= threshold=0.4905405640602112 |
| Class: former_smokers | |
| | |
| Rules_56 | passed counts:2 |
| node_0: feature_name=ENST00000284311 | feature_id[0].value <= threshold=179.44091796875 |
| node_1: feature_name=ENST00000390539 | feature_id[222].value <= threshold=11.91484260559082 |
| node_2: feature_name=ENST00000586582 | feature_id[123].value <= threshold=17.368224143981934 |
| node_3: feature_name=ENST00000284984 | feature_id[292].value <= threshold=11.201291561126709 |
| node_4: feature_name=ENST00000586582 | feature_id[123].value <= threshold=5.24660587310791 |
| node_5: feature_name=ENST00000244174 | feature_id[34].value <= threshold=2.29075288772583 |
| node_6: feature_name=ENST00000618889 | feature_id[237].value > threshold=6.870025634765625 |
| node_10: feature_name=ENST00000526097 | feature_id[88].value > threshold=14.827675819396973 |
| Class: current_smokers | |
| | |
| Rules_57 | passed counts:1 |
| node_0: feature_name=ENST00000284311 | feature_id[0].value > threshold=179.44091796875 |
| node_92: feature_name=ENST00000396276 | feature_id[298].value > threshold=957.0642700195312 |
| node_150: feature_name=ENST00000284311 | feature_id[0].value > threshold=364.31639099121094 |
| node_170: feature_name=ENST00000339223 | feature_id[10].value > threshold=33.90394592285156 |
| node_176: feature_name=ENST00000513886 | feature_id[39].value <= threshold=141.72513961791992 |
| node_177: feature_name=ENST00000443723 | feature_id[117].value > threshold=55.98150062561035 |
| Class: former_smokers | |
| | |
| Rules_58 | passed counts:1 |

| | |
|---|---|
| node_0: feature_name=ENST00000284311 | feature_id[0].value > threshold=179.44091796875 |
| node_92: feature_name=ENST00000396276 | feature_id[298].value > threshold=957.0642700195312 |
| node_150: feature_name=ENST00000284311 | feature_id[0].value > threshold=364.31639099121094 |
| node_170: feature_name=ENST00000339223 | feature_id[10].value > threshold=33.90394592285156 |
| node_176: feature_name=ENST00000513886 | feature_id[39].value <= threshold=141.72513961791992 |
| node_177: feature_name=ENST00000443723 | feature_id[117].value <= threshold=55.98150062561035 |
| node_178: feature_name=ENST00000498146 | feature_id[143].value <= threshold=2.1133410930633545 |
| Class: former_smokers | |
| | |
| Rules_59 | passed counts:1 |
| node_0: feature_name=ENST00000284311 | feature_id[0].value > threshold=179.44091796875 |
| node_92: feature_name=ENST00000396276 | feature_id[298].value > threshold=957.0642700195312 |
| node_150: feature_name=ENST00000284311 | feature_id[0].value > threshold=364.31639099121094 |
| node_170: feature_name=ENST00000339223 | feature_id[10].value <= threshold=33.90394592285156 |
| node_171: feature_name=ENST00000297785 | feature_id[83].value > threshold=208.11764526367188 |
| node_173: feature_name=ENST00000529814 | feature_id[209].value > threshold=149.9900665283203 |
| Class: current_smokers | |
| | |
| Rules_60 | passed counts:1 |
| node_0: feature_name=ENST00000284311 | feature_id[0].value > threshold=179.44091796875 |
| node_92: feature_name=ENST00000396276 | feature_id[298].value > threshold=957.0642700195312 |
| node_150: feature_name=ENST00000284311 | feature_id[0].value <= threshold=364.31639099121094 |
| node_151: feature_name=ENST00000276974 | feature_id[6].value > threshold=1.20855313539505 |
| node_161: feature_name=ENST00000641136 | feature_id[30].value > threshold=5.980504512786865 |
| node_165: feature_name=ENST00000396618 | feature_id[106].value > threshold=42.30185317993164 |
| node_167: feature_name=ENST00000548358 | feature_id[115].value > threshold=34.7273063659668 |

| | |
|---|---|
| Class: current_smokers | |
| | |
| Rules_61 | passed counts:1 |
| node_0: feature_name=ENST00000284311 | feature_id[0].value > threshold=179.44091796875 |
| node_92: feature_name=ENST00000396276 | feature_id[298].value > threshold=957.0642700195312 |
| node_150: feature_name=ENST00000284311 | feature_id[0].value <= threshold=364.31639099121094 |
| node_151: feature_name=ENST00000276974 | feature_id[6].value <= threshold=1.20855313539505 |
| node_152: feature_name=ENST00000441556 | feature_id[147].value <= threshold=8.403407096862793 |
| node_153: feature_name=ENST00000390304 | feature_id[81].value <= threshold=9.798711776733398 |
| node_154: feature_name=ENST00000390312 | feature_id[78].value > threshold=759.6060485839844 |
| Class: current_smokers | |
| | |
| Rules_62 | passed counts:1 |
| node_0: feature_name=ENST00000284311 | feature_id[0].value > threshold=179.44091796875 |
| node_92: feature_name=ENST00000396276 | feature_id[298].value <= threshold=957.0642700195312 |
| node_93: feature_name=ENST00000316418 | feature_id[181].value > threshold=3.5686380863189697 |
| node_111: feature_name=ENST00000309575 | feature_id[257].value <= threshold=375.328125 |
| node_112: feature_name=ENST00000367467 | feature_id[107].value > threshold=21.34785747528076 |
| node_116: feature_name=ENST00000571489 | feature_id[279].value > threshold=56.20589256286621 |
| node_140: feature_name=ENST00000529814 | feature_id[209].value <= threshold=103.67559623718262 |
| Class: current_smokers | |
| | |
| Rules_63 | passed counts:1 |
| node_0: feature_name=ENST00000284311 | feature_id[0].value > threshold=179.44091796875 |
| node_92: feature_name=ENST00000396276 | feature_id[298].value <= threshold=957.0642700195312 |
| node_93: feature_name=ENST00000316418 | feature_id[181].value > threshold=3.5686380863189697 |

| | |
|---|---|
| node_111: feature_name=ENST00000309575 | feature_id[257].value <= threshold=375.328125 |
| node_112: feature_name=ENST00000367467 | feature_id[107].value > threshold=21.34785747528076 |
| node_116: feature_name=ENST00000571489 | feature_id[279].value <= threshold=56.20589256286621 |
| node_117: feature_name=ENST00000635923 | feature_id[171].value <= threshold=328.1167755126953 |
| node_118: feature_name=ENST00000334529 | feature_id[148].value > threshold=60.1170597076416 |
| node_120: feature_name=ENST00000635923 | feature_id[171].value > threshold=37.20250701904297 |
| node_124: feature_name=ENST00000498146 | feature_id[143].value > threshold=0.441722571849823 |
| node_128: feature_name=ENST00000260526 | feature_id[236].value > threshold=21.659213066101074 |
| node_134: feature_name=ENST00000632136 | feature_id[41].value > threshold=155.27672576904297 |
| Class: former_smokers | |
| | |
| Rules_64 | passed counts:1 |
| node_0: feature_name=ENST00000284311 | feature_id[0].value > threshold=179.44091796875 |
| node_92: feature_name=ENST00000396276 | feature_id[298].value <= threshold=957.0642700195312 |
| node_93: feature_name=ENST00000316418 | feature_id[181].value > threshold=3.5686380863189697 |
| node_111: feature_name=ENST00000309575 | feature_id[257].value <= threshold=375.328125 |
| node_112: feature_name=ENST00000367467 | feature_id[107].value > threshold=21.34785747528076 |
| node_116: feature_name=ENST00000571489 | feature_id[279].value <= threshold=56.20589256286621 |
| node_117: feature_name=ENST00000635923 | feature_id[171].value <= threshold=328.1167755126953 |
| node_118: feature_name=ENST00000334529 | feature_id[148].value > threshold=60.1170597076416 |
| node_120: feature_name=ENST00000635923 | feature_id[171].value > threshold=37.20250701904297 |
| node_124: feature_name=ENST00000498146 | feature_id[143].value > threshold=0.441722571849823 |
| node_128: feature_name=ENST00000260526 | feature_id[236].value > threshold=21.659213066101074 |
| node_134: feature_name=ENST00000632136 | feature_id[41].value <= threshold=155.27672576904297 |
| Class: current_smokers | |
| | |

| | |
|---|---|
| Rules_65 | passed counts:1 |
| node_0: feature_name=ENST00000284311 | feature_id[0].value > threshold=179.44091796875 |
| node_92: feature_name=ENST00000396276 | feature_id[298].value <= threshold=957.0642700195312 |
| node_93: feature_name=ENST00000316418 | feature_id[181].value > threshold=3.5686380863189697 |
| node_111: feature_name=ENST00000309575 | feature_id[257].value <= threshold=375.328125 |
| node_112: feature_name=ENST00000367467 | feature_id[107].value > threshold=21.34785747528076 |
| node_116: feature_name=ENST00000571489 | feature_id[279].value <= threshold=56.20589256286621 |
| node_117: feature_name=ENST00000635923 | feature_id[171].value <= threshold=328.1167755126953 |
| node_118: feature_name=ENST00000334529 | feature_id[148].value > threshold=60.1170597076416 |
| node_120: feature_name=ENST00000635923 | feature_id[171].value > threshold=37.20250701904297 |
| node_124: feature_name=ENST00000498146 | feature_id[143].value > threshold=0.441722571849823 |
| node_128: feature_name=ENST00000260526 | feature_id[236].value <= threshold=21.659213066101074 |
| node_129: feature_name=ENST00000390325 | feature_id[17].value <= threshold=17.23688793182373 |
| node_130: feature_name=ENST00000573760 | feature_id[84].value <= threshold=10.882764339447021 |
| Class: former_smokers | |
| | |
| Rules_66 | passed counts:1 |
| node_0: feature_name=ENST00000284311 | feature_id[0].value > threshold=179.44091796875 |
| node_92: feature_name=ENST00000396276 | feature_id[298].value <= threshold=957.0642700195312 |
| node_93: feature_name=ENST00000316418 | feature_id[181].value > threshold=3.5686380863189697 |
| node_111: feature_name=ENST00000309575 | feature_id[257].value <= threshold=375.328125 |
| node_112: feature_name=ENST00000367467 | feature_id[107].value > threshold=21.34785747528076 |
| node_116: feature_name=ENST00000571489 | feature_id[279].value <= threshold=56.20589256286621 |
| node_117: feature_name=ENST00000635923 | feature_id[171].value <= threshold=328.1167755126953 |
| node_118: feature_name=ENST00000334529 | feature_id[148].value > threshold=60.1170597076416 |

| | |
|---|---|
| node_120: feature_name=ENST00000635923 | feature_id[171].value > threshold=37.20250701904297 |
| node_124: feature_name=ENST00000498146 | feature_id[143].value <= threshold=0.441722571849823 |
| node_125: feature_name=ENST00000622044 | feature_id[164].value > threshold=10.146403312683105 |
| Class: current_smokers | |
| | |
| Rules_67 | passed counts:1 |
| node_0: feature_name=ENST00000284311 | feature_id[0].value > threshold=179.44091796875 |
| node_92: feature_name=ENST00000396276 | feature_id[298].value <= threshold=957.0642700195312 |
| node_93: feature_name=ENST00000316418 | feature_id[181].value > threshold=3.5686380863189697 |
| node_111: feature_name=ENST00000309575 | feature_id[257].value <= threshold=375.328125 |
| node_112: feature_name=ENST00000367467 | feature_id[107].value > threshold=21.34785747528076 |
| node_116: feature_name=ENST00000571489 | feature_id[279].value <= threshold=56.20589256286621 |
| node_117: feature_name=ENST00000635923 | feature_id[171].value <= threshold=328.1167755126953 |
| node_118: feature_name=ENST00000334529 | feature_id[148].value > threshold=60.1170597076416 |
| node_120: feature_name=ENST00000635923 | feature_id[171].value > threshold=37.20250701904297 |
| node_124: feature_name=ENST00000498146 | feature_id[143].value <= threshold=0.441722571849823 |
| node_125: feature_name=ENST00000622044 | feature_id[164].value <= threshold=10.146403312683105 |
| Class: former_smokers | |
| | |
| Rules_68 | passed counts:1 |
| node_0: feature_name=ENST00000284311 | feature_id[0].value > threshold=179.44091796875 |
| node_92: feature_name=ENST00000396276 | feature_id[298].value <= threshold=957.0642700195312 |
| node_93: feature_name=ENST00000316418 | feature_id[181].value > threshold=3.5686380863189697 |
| node_111: feature_name=ENST00000309575 | feature_id[257].value <= threshold=375.328125 |
| node_112: feature_name=ENST00000367467 | feature_id[107].value > threshold=21.34785747528076 |
| node_116: feature_name=ENST00000571489 | feature_id[279].value <= threshold=56.20589256286621 |

| | |
|---|---|
| node_117: feature_name=ENST00000635923 | feature_id[171].value <= threshold=328.1167755126953 |
| node_118: feature_name=ENST00000334529 | feature_id[148].value > threshold=60.1170597076416 |
| node_120: feature_name=ENST00000635923 | feature_id[171].value <= threshold=37.20250701904297 |
| node_121: feature_name=ENST00000311597 | feature_id[80].value > threshold=7.991077899932861 |
| Class: current_smokers | |
| | |
| Rules_69 | passed counts:1 |
| node_0: feature_name=ENST00000284311 | feature_id[0].value > threshold=179.44091796875 |
| node_92: feature_name=ENST00000396276 | feature_id[298].value <= threshold=957.0642700195312 |
| node_93: feature_name=ENST00000316418 | feature_id[181].value > threshold=3.5686380863189697 |
| node_111: feature_name=ENST00000309575 | feature_id[257].value <= threshold=375.328125 |
| node_112: feature_name=ENST00000367467 | feature_id[107].value > threshold=21.34785747528076 |
| node_116: feature_name=ENST00000571489 | feature_id[279].value <= threshold=56.20589256286621 |
| node_117: feature_name=ENST00000635923 | feature_id[171].value <= threshold=328.1167755126953 |
| node_118: feature_name=ENST00000334529 | feature_id[148].value > threshold=60.1170597076416 |
| node_120: feature_name=ENST00000635923 | feature_id[171].value <= threshold=37.20250701904297 |
| node_121: feature_name=ENST00000311597 | feature_id[80].value <= threshold=7.991077899932861 |
| Class: former_smokers | |
| | |
| Rules_70 | passed counts:1 |
| node_0: feature_name=ENST00000284311 | feature_id[0].value > threshold=179.44091796875 |
| node_92: feature_name=ENST00000396276 | feature_id[298].value <= threshold=957.0642700195312 |
| node_93: feature_name=ENST00000316418 | feature_id[181].value > threshold=3.5686380863189697 |
| node_111: feature_name=ENST00000309575 | feature_id[257].value <= threshold=375.328125 |
| node_112: feature_name=ENST00000367467 | feature_id[107].value > threshold=21.34785747528076 |
| node_116: feature_name=ENST00000571489 | feature_id[279].value <= threshold=56.20589256286621 |

| | |
|---|---|
| node_117: feature_name=ENST00000635923 | feature_id[171].value <= threshold=328.1167755126953 |
| node_118: feature_name=ENST00000334529 | feature_id[148].value <= threshold=60.1170597076416 |
| Class: former_smokers | |
| | |
| Rules_71 | passed counts:1 |
| node_0: feature_name=ENST00000284311 | feature_id[0].value > threshold=179.44091796875 |
| node_92: feature_name=ENST00000396276 | feature_id[298].value <= threshold=957.0642700195312 |
| node_93: feature_name=ENST00000316418 | feature_id[181].value <= threshold=3.5686380863189697 |
| node_94: feature_name=ENST00000312143 | feature_id[46].value > threshold=80.9970703125 |
| node_102: feature_name=ENST00000507007 | feature_id[177].value > threshold=8.112542390823364 |
| node_108: feature_name=ENST00000261651 | feature_id[151].value <= threshold=0.5338447093963623 |
| Class: current_smokers | |
| | |
| Rules_72 | passed counts:1 |
| node_0: feature_name=ENST00000284311 | feature_id[0].value > threshold=179.44091796875 |
| node_92: feature_name=ENST00000396276 | feature_id[298].value <= threshold=957.0642700195312 |
| node_93: feature_name=ENST00000316418 | feature_id[181].value <= threshold=3.5686380863189697 |
| node_94: feature_name=ENST00000312143 | feature_id[46].value <= threshold=80.9970703125 |
| node_95: feature_name=ENST00000610349 | feature_id[275].value > threshold=6.537376165390015 |
| node_97: feature_name=ENST00000423064 | feature_id[240].value <= threshold=22.101216316223145 |
| node_98: feature_name=ENST00000613640 | feature_id[250].value > threshold=205.0992202758789 |
| Class: former_smokers | |
| | |
| Rules_73 | passed counts:1 |
| node_0: feature_name=ENST00000284311 | feature_id[0].value <= threshold=179.44091796875 |
| node_1: feature_name=ENST00000390539 | feature_id[222].value <= threshold=11.91484260559082 |

| | |
|---|---|
| node_2: feature_name=ENST00000586582 | feature_id[123].value <= threshold=17.368224143981934 |
| node_3: feature_name=ENST00000284984 | feature_id[292].value > threshold=11.201291561126709 |
| node_23: feature_name=ENST00000276974 | feature_id[6].value > threshold=75.58467483520508 |
| node_75: feature_name=ENST00000415351 | feature_id[216].value > threshold=3.5765366554260254 |
| Class: former_smokers | |
| | |
| Rules_74 | passed counts:1 |
| node_0: feature_name=ENST00000284311 | feature_id[0].value <= threshold=179.44091796875 |
| node_1: feature_name=ENST00000390539 | feature_id[222].value <= threshold=11.91484260559082 |
| node_2: feature_name=ENST00000586582 | feature_id[123].value <= threshold=17.368224143981934 |
| node_3: feature_name=ENST00000284984 | feature_id[292].value > threshold=11.201291561126709 |
| node_23: feature_name=ENST00000276974 | feature_id[6].value <= threshold=75.58467483520508 |
| node_24: feature_name=ENST00000464302 | feature_id[285].value > threshold=25.290170669555664 |
| node_28: feature_name=ENST00000650242 | feature_id[234].value > threshold=1287.966552734375 |
| node_32: feature_name=ENST00000491977 | feature_id[29].value <= threshold=22.76432514190674 |
| node_33: feature_name=ENST00000611977 | feature_id[43].value > threshold=0.45197004079818726 |
| node_35: feature_name=ENST00000503771 | feature_id[193].value > threshold=320.5686798095703 |
| Class: current_smokers | |
| | |
| Rules_75 | passed counts:1 |
| node_0: feature_name=ENST00000284311 | feature_id[0].value <= threshold=179.44091796875 |
| node_1: feature_name=ENST00000390539 | feature_id[222].value <= threshold=11.91484260559082 |
| node_2: feature_name=ENST00000586582 | feature_id[123].value <= threshold=17.368224143981934 |
| node_3: feature_name=ENST00000284984 | feature_id[292].value > threshold=11.201291561126709 |
| node_23: feature_name=ENST00000276974 | feature_id[6].value <= threshold=75.58467483520508 |
| node_24: feature_name=ENST00000464302 | feature_id[285].value > threshold=25.290170669555664 |

| | |
|---|---|
| node_28: feature_name=ENST00000650242 | feature_id[234].value > threshold=1287.966552734375 |
| node_32: feature_name=ENST00000491977 | feature_id[29].value <= threshold=22.76432514190674 |
| node_33: feature_name=ENST00000611977 | feature_id[43].value > threshold=0.45197004079818726 |
| node_35: feature_name=ENST00000503771 | feature_id[193].value <= threshold=320.5686798095703 |
| node_36: feature_name=ENST00000307407 | feature_id[281].value > threshold=4.887194871902466 |
| node_38: feature_name=ENST00000321935 | feature_id[238].value > threshold=22.123645782470703 |
| node_40: feature_name=ENST00000492167 | feature_id[111].value > threshold=833.0198974609375 |
| Class: current_smokers | |
| | |
| Rules_76 | passed counts:1 |
| node_0: feature_name=ENST00000284311 | feature_id[0].value <= threshold=179.44091796875 |
| node_1: feature_name=ENST00000390539 | feature_id[222].value <= threshold=11.91484260559082 |
| node_2: feature_name=ENST00000586582 | feature_id[123].value <= threshold=17.368224143981934 |
| node_3: feature_name=ENST00000284984 | feature_id[292].value > threshold=11.201291561126709 |
| node_23: feature_name=ENST00000276974 | feature_id[6].value <= threshold=75.58467483520508 |
| node_24: feature_name=ENST00000464302 | feature_id[285].value > threshold=25.290170669555664 |
| node_28: feature_name=ENST00000650242 | feature_id[234].value > threshold=1287.966552734375 |
| node_32: feature_name=ENST00000491977 | feature_id[29].value <= threshold=22.76432514190674 |
| node_33: feature_name=ENST00000611977 | feature_id[43].value > threshold=0.45197004079818726 |
| node_35: feature_name=ENST00000503771 | feature_id[193].value <= threshold=320.5686798095703 |
| node_36: feature_name=ENST00000307407 | feature_id[281].value > threshold=4.887194871902466 |
| node_38: feature_name=ENST00000321935 | feature_id[238].value > threshold=22.123645782470703 |
| node_40: feature_name=ENST00000492167 | feature_id[111].value <= threshold=833.0198974609375 |
| node_41: feature_name=ENST00000005178 | feature_id[118].value > threshold=29.375121116638184 |
| node_43: feature_name=ENST00000411764 | feature_id[20].value > threshold=186.49674224853516 |

| | |
|---|---|
| Class: current_smokers | |
| | |
| Rules_77 | passed counts:1 |
| node_0: feature_name=ENST00000284311 | feature_id[0].value <= threshold=179.44091796875 |
| node_1: feature_name=ENST00000390539 | feature_id[222].value <= threshold=11.91484260559082 |
| node_2: feature_name=ENST00000586582 | feature_id[123].value <= threshold=17.368224143981934 |
| node_3: feature_name=ENST00000284984 | feature_id[292].value > threshold=11.201291561126709 |
| node_23: feature_name=ENST00000276974 | feature_id[6].value <= threshold=75.58467483520508 |
| node_24: feature_name=ENST00000464302 | feature_id[285].value > threshold=25.290170669555664 |
| node_28: feature_name=ENST00000650242 | feature_id[234].value > threshold=1287.966552734375 |
| node_32: feature_name=ENST00000491977 | feature_id[29].value <= threshold=22.76432514190674 |
| node_33: feature_name=ENST00000611977 | feature_id[43].value > threshold=0.45197004079818726 |
| node_35: feature_name=ENST00000503771 | feature_id[193].value <= threshold=320.5686798095703 |
| node_36: feature_name=ENST00000307407 | feature_id[281].value > threshold=4.887194871902466 |
| node_38: feature_name=ENST00000321935 | feature_id[238].value > threshold=22.123645782470703 |
| node_40: feature_name=ENST00000492167 | feature_id[111].value <= threshold=833.0198974609375 |
| node_41: feature_name=ENST00000005178 | feature_id[118].value > threshold=29.375121116638184 |
| node_43: feature_name=ENST00000411764 | feature_id[20].value <= threshold=186.49674224853516 |
| node_44: feature_name=ENST00000395002 | feature_id[256].value > threshold=13.3489990234375 |
| node_48: feature_name=ENST00000636279 | feature_id[103].value > threshold=9.28081226348877 |
| node_66: feature_name=ENST00000543780 | feature_id[242].value > threshold=157.6930694580078 |
| Class: former_smokers | |
| | |
| Rules_78 | passed counts:1 |
| node_0: feature_name=ENST00000284311 | feature_id[0].value <= threshold=179.44091796875 |
| node_1: feature_name=ENST00000390539 | feature_id[222].value <= threshold=11.91484260559082 |

| | |
|---|---|
| node_2: feature_name=ENST00000586582 | feature_id[123].value <= threshold=17.368224143981934 |
| node_3: feature_name=ENST00000284984 | feature_id[292].value > threshold=11.201291561126709 |
| node_23: feature_name=ENST00000276974 | feature_id[6].value <= threshold=75.58467483520508 |
| node_24: feature_name=ENST00000464302 | feature_id[285].value > threshold=25.290170669555664 |
| node_28: feature_name=ENST00000650242 | feature_id[234].value > threshold=1287.966552734375 |
| node_32: feature_name=ENST00000491977 | feature_id[29].value <= threshold=22.76432514190674 |
| node_33: feature_name=ENST00000611977 | feature_id[43].value > threshold=0.45197004079818726 |
| node_35: feature_name=ENST00000503771 | feature_id[193].value <= threshold=320.5686798095703 |
| node_36: feature_name=ENST00000307407 | feature_id[281].value > threshold=4.887194871902466 |
| node_38: feature_name=ENST00000321935 | feature_id[238].value > threshold=22.123645782470703 |
| node_40: feature_name=ENST00000492167 | feature_id[111].value <= threshold=833.0198974609375 |
| node_41: feature_name=ENST00000005178 | feature_id[118].value > threshold=29.375121116638184 |
| node_43: feature_name=ENST00000411764 | feature_id[20].value <= threshold=186.49674224853516 |
| node_44: feature_name=ENST00000395002 | feature_id[256].value > threshold=13.3489990234375 |
| node_48: feature_name=ENST00000636279 | feature_id[103].value > threshold=9.28081226348877 |
| node_66: feature_name=ENST00000543780 | feature_id[242].value <= threshold=157.6930694580078 |
| Class: current_smokers | |
| | |
| Rules_79 | passed counts:1 |
| node_0: feature_name=ENST00000284311 | feature_id[0].value <= threshold=179.44091796875 |
| node_1: feature_name=ENST00000390539 | feature_id[222].value <= threshold=11.91484260559082 |
| node_2: feature_name=ENST00000586582 | feature_id[123].value <= threshold=17.368224143981934 |
| node_3: feature_name=ENST00000284984 | feature_id[292].value > threshold=11.201291561126709 |
| node_23: feature_name=ENST00000276974 | feature_id[6].value <= threshold=75.58467483520508 |
| node_24: feature_name=ENST00000464302 | feature_id[285].value > threshold=25.290170669555664 |

| | |
|---|---|
| node_28: feature_name=ENST00000650242 | feature_id[234].value > threshold=1287.966552734375 |
| node_32: feature_name=ENST00000491977 | feature_id[29].value <= threshold=22.76432514190674 |
| node_33: feature_name=ENST00000611977 | feature_id[43].value > threshold=0.45197004079818726 |
| node_35: feature_name=ENST00000503771 | feature_id[193].value <= threshold=320.5686798095703 |
| node_36: feature_name=ENST00000307407 | feature_id[281].value > threshold=4.887194871902466 |
| node_38: feature_name=ENST00000321935 | feature_id[238].value > threshold=22.123645782470703 |
| node_40: feature_name=ENST00000492167 | feature_id[111].value <= threshold=833.0198974609375 |
| node_41: feature_name=ENST00000005178 | feature_id[118].value > threshold=29.375121116638184 |
| node_43: feature_name=ENST00000411764 | feature_id[20].value <= threshold=186.49674224853516 |
| node_44: feature_name=ENST00000395002 | feature_id[256].value > threshold=13.3489990234375 |
| node_48: feature_name=ENST00000636279 | feature_id[103].value <= threshold=9.28081226348877 |
| node_49: feature_name=ENST00000244174 | feature_id[34].value <= threshold=10.21084976196289 |
| node_50: feature_name=ENST00000393203 | feature_id[91].value > threshold=21.360187530517578 |
| node_56: feature_name=ENST00000286732 | feature_id[131].value > threshold=1.1582629084587097 |
| node_58: feature_name=ENST00000643697 | feature_id[132].value > threshold=12.256080150604248 |
| Class: current_smokers | |
| | |
| Rules_80 | passed counts:1 |
| node_0: feature_name=ENST00000284311 | feature_id[0].value <= threshold=179.44091796875 |
| node_1: feature_name=ENST00000390539 | feature_id[222].value <= threshold=11.91484260559082 |
| node_2: feature_name=ENST00000586582 | feature_id[123].value <= threshold=17.368224143981934 |
| node_3: feature_name=ENST00000284984 | feature_id[292].value > threshold=11.201291561126709 |
| node_23: feature_name=ENST00000276974 | feature_id[6].value <= threshold=75.58467483520508 |
| node_24: feature_name=ENST00000464302 | feature_id[285].value > threshold=25.290170669555664 |
| node_28: feature_name=ENST00000650242 | feature_id[234].value > threshold=1287.966552734375 |

| | |
|---|---|
| node_32: feature_name=ENST00000491977 | feature_id[29].value <= threshold=22.76432514190674 |
| node_33: feature_name=ENST00000611977 | feature_id[43].value > threshold=0.45197004079818726 |
| node_35: feature_name=ENST00000503771 | feature_id[193].value <= threshold=320.5686798095703 |
| node_36: feature_name=ENST00000307407 | feature_id[281].value > threshold=4.887194871902466 |
| node_38: feature_name=ENST00000321935 | feature_id[238].value > threshold=22.123645782470703 |
| node_40: feature_name=ENST00000492167 | feature_id[111].value <= threshold=833.0198974609375 |
| node_41: feature_name=ENST00000005178 | feature_id[118].value > threshold=29.375121116638184 |
| node_43: feature_name=ENST00000411764 | feature_id[20].value <= threshold=186.49674224853516 |
| node_44: feature_name=ENST00000395002 | feature_id[256].value > threshold=13.3489990234375 |
| node_48: feature_name=ENST00000636279 | feature_id[103].value <= threshold=9.28081226348877 |
| node_49: feature_name=ENST00000244174 | feature_id[34].value <= threshold=10.21084976196289 |
| node_50: feature_name=ENST00000393203 | feature_id[91].value > threshold=21.360187530517578 |
| node_56: feature_name=ENST00000286732 | feature_id[131].value > threshold=1.1582629084587097 |
| node_58: feature_name=ENST00000643697 | feature_id[132].value <= threshold=12.256080150604248 |
| node_59: feature_name=ENST00000390606 | feature_id[293].value > threshold=15.513253211975098 |
| Class: current_smokers | |
| | |
| Rules_81 | passed counts:1 |
| node_0: feature_name=ENST00000284311 | feature_id[0].value <= threshold=179.44091796875 |
| node_1: feature_name=ENST00000390539 | feature_id[222].value <= threshold=11.91484260559082 |
| node_2: feature_name=ENST00000586582 | feature_id[123].value <= threshold=17.368224143981934 |
| node_3: feature_name=ENST00000284984 | feature_id[292].value > threshold=11.201291561126709 |
| node_23: feature_name=ENST00000276974 | feature_id[6].value <= threshold=75.58467483520508 |
| node_24: feature_name=ENST00000464302 | feature_id[285].value > threshold=25.290170669555664 |
| node_28: feature_name=ENST00000650242 | feature_id[234].value > threshold=1287.966552734375 |

| | |
|---|---|
| node_32: feature_name=ENST00000491977 | feature_id[29].value <= threshold=22.76432514190674 |
| node_33: feature_name=ENST00000611977 | feature_id[43].value > threshold=0.45197004079818726 |
| node_35: feature_name=ENST00000503771 | feature_id[193].value <= threshold=320.5686798095703 |
| node_36: feature_name=ENST00000307407 | feature_id[281].value > threshold=4.887194871902466 |
| node_38: feature_name=ENST00000321935 | feature_id[238].value > threshold=22.123645782470703 |
| node_40: feature_name=ENST00000492167 | feature_id[111].value <= threshold=833.0198974609375 |
| node_41: feature_name=ENST00000005178 | feature_id[118].value > threshold=29.375121116638184 |
| node_43: feature_name=ENST00000411764 | feature_id[20].value <= threshold=186.49674224853516 |
| node_44: feature_name=ENST00000395002 | feature_id[256].value > threshold=13.3489990234375 |
| node_48: feature_name=ENST00000636279 | feature_id[103].value <= threshold=9.28081226348877 |
| node_49: feature_name=ENST00000244174 | feature_id[34].value <= threshold=10.21084976196289 |
| node_50: feature_name=ENST00000393203 | feature_id[91].value <= threshold=21.360187530517578 |
| node_51: feature_name=ENST00000278919 | feature_id[121].value > threshold=45.06623077392578 |
| node_53: feature_name=ENST00000607161 | feature_id[195].value <= threshold=7.280014753341675 |
| Class: current_smokers | |
| | |
| Rules_82 | passed counts:1 |
| node_0: feature_name=ENST00000284311 | feature_id[0].value <= threshold=179.44091796875 |
| node_1: feature_name=ENST00000390539 | feature_id[222].value <= threshold=11.91484260559082 |
| node_2: feature_name=ENST00000586582 | feature_id[123].value <= threshold=17.368224143981934 |
| node_3: feature_name=ENST00000284984 | feature_id[292].value > threshold=11.201291561126709 |
| node_23: feature_name=ENST00000276974 | feature_id[6].value <= threshold=75.58467483520508 |
| node_24: feature_name=ENST00000464302 | feature_id[285].value > threshold=25.290170669555664 |
| node_28: feature_name=ENST00000650242 | feature_id[234].value > threshold=1287.966552734375 |
| node_32: feature_name=ENST00000491977 | feature_id[29].value <= threshold=22.76432514190674 |

| | |
|---|---|
| node_33: feature_name=ENST00000611977 | feature_id[43].value > threshold=0.45197004079818726 |
| node_35: feature_name=ENST00000503771 | feature_id[193].value <= threshold=320.5686798095703 |
| node_36: feature_name=ENST00000307407 | feature_id[281].value > threshold=4.887194871902466 |
| node_38: feature_name=ENST00000321935 | feature_id[238].value > threshold=22.123645782470703 |
| node_40: feature_name=ENST00000492167 | feature_id[111].value <= threshold=833.0198974609375 |
| node_41: feature_name=ENST00000005178 | feature_id[118].value > threshold=29.375121116638184 |
| node_43: feature_name=ENST00000411764 | feature_id[20].value <= threshold=186.49674224853516 |
| node_44: feature_name=ENST00000395002 | feature_id[256].value <= threshold=13.3489990234375 |
| node_45: feature_name=ENST00000507007 | feature_id[177].value > threshold=5.1506428718566895 |
| Class: current_smokers | |
| | |
| Rules_83 | passed counts:1 |
| node_0: feature_name=ENST00000284311 | feature_id[0].value <= threshold=179.44091796875 |
| node_1: feature_name=ENST00000390539 | feature_id[222].value <= threshold=11.91484260559082 |
| node_2: feature_name=ENST00000586582 | feature_id[123].value <= threshold=17.368224143981934 |
| node_3: feature_name=ENST00000284984 | feature_id[292].value > threshold=11.201291561126709 |
| node_23: feature_name=ENST00000276974 | feature_id[6].value <= threshold=75.58467483520508 |
| node_24: feature_name=ENST00000464302 | feature_id[285].value > threshold=25.290170669555664 |
| node_28: feature_name=ENST00000650242 | feature_id[234].value > threshold=1287.966552734375 |
| node_32: feature_name=ENST00000491977 | feature_id[29].value <= threshold=22.76432514190674 |
| node_33: feature_name=ENST00000611977 | feature_id[43].value > threshold=0.45197004079818726 |
| node_35: feature_name=ENST00000503771 | feature_id[193].value <= threshold=320.5686798095703 |
| node_36: feature_name=ENST00000307407 | feature_id[281].value > threshold=4.887194871902466 |
| node_38: feature_name=ENST00000321935 | feature_id[238].value > threshold=22.123645782470703 |
| node_40: feature_name=ENST00000492167 | feature_id[111].value <= threshold=833.0198974609375 |

| | |
|---|---|
| node_41: feature_name=ENST00000005178 | feature_id[118].value > threshold=29.375121116638184 |
| node_43: feature_name=ENST00000411764 | feature_id[20].value <= threshold=186.49674224853516 |
| node_44: feature_name=ENST00000395002 | feature_id[256].value <= threshold=13.3489990234375 |
| node_45: feature_name=ENST00000507007 | feature_id[177].value <= threshold=5.1506428718566895 |
| Class: former_smokers | |
| | |
| Rules_84 | passed counts:1 |
| node_0: feature_name=ENST00000284311 | feature_id[0].value <= threshold=179.44091796875 |
| node_1: feature_name=ENST00000390539 | feature_id[222].value <= threshold=11.91484260559082 |
| node_2: feature_name=ENST00000586582 | feature_id[123].value <= threshold=17.368224143981934 |
| node_3: feature_name=ENST00000284984 | feature_id[292].value > threshold=11.201291561126709 |
| node_23: feature_name=ENST00000276974 | feature_id[6].value <= threshold=75.58467483520508 |
| node_24: feature_name=ENST00000464302 | feature_id[285].value > threshold=25.290170669555664 |
| node_28: feature_name=ENST00000650242 | feature_id[234].value > threshold=1287.966552734375 |
| node_32: feature_name=ENST00000491977 | feature_id[29].value <= threshold=22.76432514190674 |
| node_33: feature_name=ENST00000611977 | feature_id[43].value > threshold=0.45197004079818726 |
| node_35: feature_name=ENST00000503771 | feature_id[193].value <= threshold=320.5686798095703 |
| node_36: feature_name=ENST00000307407 | feature_id[281].value > threshold=4.887194871902466 |
| node_38: feature_name=ENST00000321935 | feature_id[238].value > threshold=22.123645782470703 |
| node_40: feature_name=ENST00000492167 | feature_id[111].value <= threshold=833.0198974609375 |
| node_41: feature_name=ENST00000005178 | feature_id[118].value <= threshold=29.375121116638184 |
| Class: current_smokers | |
| | |
| Rules_85 | passed counts:1 |
| node_0: feature_name=ENST00000284311 | feature_id[0].value <= threshold=179.44091796875 |
| node_1: feature_name=ENST00000390539 | feature_id[222].value <= threshold=11.91484260559082 |

| | |
|---|---|
| node_2: feature_name=ENST00000586582 | feature_id[123].value <= threshold=17.368224143981934 |
| node_3: feature_name=ENST00000284984 | feature_id[292].value > threshold=11.201291561126709 |
| node_23: feature_name=ENST00000276974 | feature_id[6].value <= threshold=75.58467483520508 |
| node_24: feature_name=ENST00000464302 | feature_id[285].value > threshold=25.290170669555664 |
| node_28: feature_name=ENST00000650242 | feature_id[234].value > threshold=1287.966552734375 |
| node_32: feature_name=ENST00000491977 | feature_id[29].value <= threshold=22.76432514190674 |
| node_33: feature_name=ENST00000611977 | feature_id[43].value > threshold=0.45197004079818726 |
| node_35: feature_name=ENST00000503771 | feature_id[193].value <= threshold=320.5686798095703 |
| node_36: feature_name=ENST00000307407 | feature_id[281].value > threshold=4.887194871902466 |
| node_38: feature_name=ENST00000321935 | feature_id[238].value <= threshold=22.123645782470703 |
| Class: current_smokers | |
| | |
| Rules_86 | passed counts:1 |
| node_0: feature_name=ENST00000284311 | feature_id[0].value <= threshold=179.44091796875 |
| node_1: feature_name=ENST00000390539 | feature_id[222].value <= threshold=11.91484260559082 |
| node_2: feature_name=ENST00000586582 | feature_id[123].value <= threshold=17.368224143981934 |
| node_3: feature_name=ENST00000284984 | feature_id[292].value > threshold=11.201291561126709 |
| node_23: feature_name=ENST00000276974 | feature_id[6].value <= threshold=75.58467483520508 |
| node_24: feature_name=ENST00000464302 | feature_id[285].value > threshold=25.290170669555664 |
| node_28: feature_name=ENST00000650242 | feature_id[234].value > threshold=1287.966552734375 |
| node_32: feature_name=ENST00000491977 | feature_id[29].value <= threshold=22.76432514190674 |
| node_33: feature_name=ENST00000611977 | feature_id[43].value > threshold=0.45197004079818726 |
| node_35: feature_name=ENST00000503771 | feature_id[193].value <= threshold=320.5686798095703 |
| node_36: feature_name=ENST00000307407 | feature_id[281].value <= threshold=4.887194871902466 |
| Class: current_smokers | |
| | |

| | |
|---|---|
| Rules_87 | passed counts:1 |
| node_0: feature_name=ENST00000284311 | feature_id[0].value <= threshold=179.44091796875 |
| node_1: feature_name=ENST00000390539 | feature_id[222].value <= threshold=11.91484260559082 |
| node_2: feature_name=ENST00000586582 | feature_id[123].value <= threshold=17.368224143981934 |
| node_3: feature_name=ENST00000284984 | feature_id[292].value > threshold=11.201291561126709 |
| node_23: feature_name=ENST00000276974 | feature_id[6].value <= threshold=75.58467483520508 |
| node_24: feature_name=ENST00000464302 | feature_id[285].value > threshold=25.290170669555664 |
| node_28: feature_name=ENST00000650242 | feature_id[234].value > threshold=1287.966552734375 |
| node_32: feature_name=ENST00000491977 | feature_id[29].value <= threshold=22.76432514190674 |
| node_33: feature_name=ENST00000611977 | feature_id[43].value <= threshold=0.45197004079818726 |
| Class: current_smokers | |
| | |
| Rules_88 | passed counts:1 |
| node_0: feature_name=ENST00000284311 | feature_id[0].value <= threshold=179.44091796875 |
| node_1: feature_name=ENST00000390539 | feature_id[222].value <= threshold=11.91484260559082 |
| node_2: feature_name=ENST00000586582 | feature_id[123].value <= threshold=17.368224143981934 |
| node_3: feature_name=ENST00000284984 | feature_id[292].value > threshold=11.201291561126709 |
| node_23: feature_name=ENST00000276974 | feature_id[6].value <= threshold=75.58467483520508 |
| node_24: feature_name=ENST00000464302 | feature_id[285].value <= threshold=25.290170669555664 |
| node_25: feature_name=ENST00000390305 | feature_id[272].value <= threshold=11.122980833053589 |
| Class: former_smokers | |
| | |
| Rules_89 | passed counts:1 |
| node_0: feature_name=ENST00000284311 | feature_id[0].value <= threshold=179.44091796875 |
| node_1: feature_name=ENST00000390539 | feature_id[222].value <= threshold=11.91484260559082 |
| node_2: feature_name=ENST00000586582 | feature_id[123].value <= threshold=17.368224143981934 |

| | |
|---|---|
| node_3: feature_name=ENST00000284984 | feature_id[292].value <= threshold=11.201291561126709 |
| node_4: feature_name=ENST00000586582 | feature_id[123].value > threshold=5.24660587310791 |
| node_18: feature_name=ENST00000611977 | feature_id[43].value <= threshold=12.043715476989746 |
| node_19: feature_name=ENST00000483158 | feature_id[197].value <= threshold=6.121511459350586 |
| Class: former_smokers | |
| | |
| Rules_90 | passed counts:1 |
| node_0: feature_name=ENST00000284311 | feature_id[0].value <= threshold=179.44091796875 |
| node_1: feature_name=ENST00000390539 | feature_id[222].value <= threshold=11.91484260559082 |
| node_2: feature_name=ENST00000586582 | feature_id[123].value <= threshold=17.368224143981934 |
| node_3: feature_name=ENST00000284984 | feature_id[292].value <= threshold=11.201291561126709 |
| node_4: feature_name=ENST00000586582 | feature_id[123].value <= threshold=5.24660587310791 |
| node_5: feature_name=ENST00000244174 | feature_id[34].value <= threshold=2.29075288772583 |
| node_6: feature_name=ENST00000618889 | feature_id[237].value > threshold=6.870025634765625 |
| node_10: feature_name=ENST00000526097 | feature_id[88].value <= threshold=14.827675819396973 |
| Class: former_smokers | |
| | |
| Rules_91 | passed counts:1 |
| node_0: feature_name=ENST00000284311 | feature_id[0].value <= threshold=179.44091796875 |
| node_1: feature_name=ENST00000390539 | feature_id[222].value <= threshold=11.91484260559082 |
| node_2: feature_name=ENST00000586582 | feature_id[123].value <= threshold=17.368224143981934 |
| node_3: feature_name=ENST00000284984 | feature_id[292].value <= threshold=11.201291561126709 |
| node_4: feature_name=ENST00000586582 | feature_id[123].value <= threshold=5.24660587310791 |
| node_5: feature_name=ENST00000244174 | feature_id[34].value <= threshold=2.29075288772583 |
| node_6: feature_name=ENST00000618889 | feature_id[237].value <= threshold=6.870025634765625 |
| node_7: feature_name=ENST00000453044 | feature_id[174].value > threshold=72.99653625488281 |

| | |
|---|---|
| Class: current_smokers | |

(2) Rules on MCFS feature ranking results

| | |
|---|---|
| Rules_0 | passed counts:360 |
| node_0: feature_name=ENST00000284311 | feature_id[0].value <= threshold=179.44091796875 |
| node_1: feature_name=ENST00000390539 | feature_id[5].value <= threshold=11.91484260559082 |
| node_2: feature_name=ENST00000586582 | feature_id[3].value <= threshold=17.368224143981934 |
| node_3: feature_name=ENST00000280258 | feature_id[56].value > threshold=75.14949798583984 |
| node_39: feature_name=ENST00000438425 | feature_id[40].value <= threshold=37.27690362930298 |
| node_40: feature_name=ENST00000393590 | feature_id[57].value <= threshold=16.18181800842285 |
| node_41: feature_name=ENST00000316418 | feature_id[2].value <= threshold=7.755321025848389 |
| node_42: feature_name=ENST00000390606 | feature_id[27].value <= threshold=15.668272972106934 |
| node_43: feature_name=ENST00000441556 | feature_id[24].value <= threshold=9.982987880706787 |
| node_44: feature_name=ENST00000610495 | feature_id[53].value > threshold=8.27418327331543 |
| node_48: feature_name=ENST00000441556 | feature_id[24].value <= threshold=5.98947286605835 |
| node_49: feature_name=ENST00000284311 | feature_id[0].value <= threshold=173.78553009033203 |
| Class: former_smokers | |
| | |
| Rules_1 | passed counts:172 |
| node_0: feature_name=ENST00000284311 | feature_id[0].value > threshold=179.44091796875 |
| node_102: feature_name=ENST00000316418 | feature_id[2].value > threshold=3.560240387916565 |
| node_136: feature_name=ENST00000380672 | feature_id[32].value <= threshold=31.704580307006836 |
| node_137: feature_name=ENST00000308478 | feature_id[1].value > threshold=50.736541748046875 |
| node_155: feature_name=ENST00000390309 | feature_id[41].value > threshold=8.32264518737793 |
| node_159: feature_name=ENST00000580335 | feature_id[63].value > threshold=1.7440648078918457 |

| | |
|---|---|
| node_161: feature_name=ENST00000422622 | feature_id[52].value <= threshold=76.81717681884766 |
| node_162: feature_name=ENST00000634222 | feature_id[36].value > threshold=1.5202934741973877 |
| node_164: feature_name=ENST00000393590 | feature_id[57].value <= threshold=15.50696325302124 |
| node_165: feature_name=ENST00000438425 | feature_id[40].value <= threshold=38.6440544128418 |
| node_166: feature_name=ENST00000367434 | feature_id[21].value <= threshold=178.1705780029297 |
| node_167: feature_name=ENST00000441556 | feature_id[24].value <= threshold=20.893842697143555 |
| node_168: feature_name=ENST00000280258 | feature_id[56].value <= threshold=250.8539047241211 |
| Class: current_smokers | |
| | |
| Rules_2 | passed counts:96 |
| node_0: feature_name=ENST00000284311 | feature_id[0].value <= threshold=179.44091796875 |
| node_1: feature_name=ENST00000390539 | feature_id[5].value <= threshold=11.91484260559082 |
| node_2: feature_name=ENST00000586582 | feature_id[3].value <= threshold=17.368224143981934 |
| node_3: feature_name=ENST00000280258 | feature_id[56].value <= threshold=75.14949798583984 |
| node_4: feature_name=ENST00000284311 | feature_id[0].value <= threshold=100.91744613647461 |
| node_5: feature_name=ENST00000492167 | feature_id[23].value <= threshold=164.934326171875 |
| node_6: feature_name=ENST00000616417 | feature_id[39].value > threshold=0.00022576878836844116 |
| node_8: feature_name=ENST00000276974 | feature_id[49].value <= threshold=19.783329486846924 |
| node_9: feature_name=ENST00000636279 | feature_id[48].value <= threshold=8.218245029449463 |
| node_10: feature_name=ENST00000464835 | feature_id[6].value <= threshold=17.562889099121094 |
| node_11: feature_name=ENST00000390323 | feature_id[22].value > threshold=50.83455848693848 |
| node_15: feature_name=ENST00000367467 | feature_id[9].value <= threshold=75.31536102294922 |
| Class: former_smokers | |
| | |
| Rules_3 | passed counts:41 |
| node_0: feature_name=ENST00000284311 | feature_id[0].value > threshold=179.44091796875 |

| | |
|---|---|
| node_102: feature_name=ENST00000316418 | feature_id[2].value <= threshold=3.560240387916565 |
| node_103: feature_name=ENST00000522551 | feature_id[60].value <= threshold=57.16539001464844 |
| node_104: feature_name=ENST00000367467 | feature_id[9].value <= threshold=43.146501541137695 |
| node_105: feature_name=ENST00000480786 | feature_id[62].value <= threshold=3.7895997762680054 |
| node_106: feature_name=ENST00000390285 | feature_id[42].value > threshold=1.2267921566963196 |
| Class: former_smokers | |
| | |
| Rules_4 | passed counts:38 |
| node_0: feature_name=ENST00000284311 | feature_id[0].value > threshold=179.44091796875 |
| node_102: feature_name=ENST00000316418 | feature_id[2].value > threshold=3.560240387916565 |
| node_136: feature_name=ENST00000380672 | feature_id[32].value > threshold=31.704580307006836 |
| node_184: feature_name=ENST00000339223 | feature_id[13].value > threshold=37.65940284729004 |
| node_200: feature_name=ENST00000359228 | feature_id[31].value <= threshold=14.81732177734375 |
| node_201: feature_name=ENST00000464835 | feature_id[6].value > threshold=3.9680298566818237 |
| node_205: feature_name=ENST00000390305 | feature_id[14].value <= threshold=446.3670883178711 |
| node_206: feature_name=ENST00000523272 | feature_id[20].value <= threshold=15.061781883239746 |
| node_207: feature_name=ENST00000390285 | feature_id[42].value > threshold=0.7228579521179199 |
| Class: current_smokers | |
| | |
| Rules_5 | passed counts:34 |
| node_0: feature_name=ENST00000284311 | feature_id[0].value <= threshold=179.44091796875 |
| node_1: feature_name=ENST00000390539 | feature_id[5].value <= threshold=11.91484260559082 |
| node_2: feature_name=ENST00000586582 | feature_id[3].value <= threshold=17.368224143981934 |
| node_3: feature_name=ENST00000280258 | feature_id[56].value > threshold=75.14949798583984 |
| node_39: feature_name=ENST00000438425 | feature_id[40].value <= threshold=37.27690362930298 |
| node_40: feature_name=ENST00000393590 | feature_id[57].value <= threshold=16.18181800842285 |

| | |
|---|---|
| node_41: feature_name=ENST00000316418 | feature_id[2].value > threshold=7.755321025848389 |
| node_65: feature_name=ENST00000244174 | feature_id[10].value <= threshold=3.6251673698425293 |
| node_66: feature_name=ENST00000586582 | feature_id[3].value <= threshold=12.385812759399414 |
| Class: former_smokers | |
| | |
| Rules_6 | passed counts:30 |
| node_0: feature_name=ENST00000284311 | feature_id[0].value > threshold=179.44091796875 |
| node_102: feature_name=ENST00000316418 | feature_id[2].value > threshold=3.560240387916565 |
| node_136: feature_name=ENST00000380672 | feature_id[32].value <= threshold=31.704580307006836 |
| node_137: feature_name=ENST00000308478 | feature_id[1].value <= threshold=50.736541748046875 |
| node_138: feature_name=ENST00000610495 | feature_id[53].value <= threshold=247.7474594116211 |
| node_139: feature_name=ENST00000367929 | feature_id[58].value <= threshold=112.91791152954102 |
| node_140: feature_name=ENST00000390290 | feature_id[46].value > threshold=23.118605613708496 |
| Class: current_smokers | |
| | |
| Rules_7 | passed counts:23 |
| node_0: feature_name=ENST00000284311 | feature_id[0].value > threshold=179.44091796875 |
| node_102: feature_name=ENST00000316418 | feature_id[2].value <= threshold=3.560240387916565 |
| node_103: feature_name=ENST00000522551 | feature_id[60].value > threshold=57.16539001464844 |
| node_125: feature_name=ENST00000390323 | feature_id[22].value > threshold=204.28018188476562 |
| node_127: feature_name=ENST00000280258 | feature_id[56].value <= threshold=121.94679641723633 |
| Class: current_smokers | |
| | |
| Rules_8 | passed counts:22 |
| node_0: feature_name=ENST00000284311 | feature_id[0].value <= threshold=179.44091796875 |
| node_1: feature_name=ENST00000390539 | feature_id[5].value <= threshold=11.91484260559082 |

| | |
|---|---|
| node_2: feature_name=ENST00000586582 | feature_id[3].value <= threshold=17.368224143981934 |
| node_3: feature_name=ENST00000280258 | feature_id[56].value <= threshold=75.14949798583984 |
| node_4: feature_name=ENST00000284311 | feature_id[0].value > threshold=100.91744613647461 |
| node_24: feature_name=ENST00000367467 | feature_id[9].value > threshold=35.77326202392578 |
| node_30: feature_name=ENST00000616417 | feature_id[39].value <= threshold=164.16122436523438 |
| node_31: feature_name=ENST00000359228 | feature_id[31].value <= threshold=8.815360069274902 |
| node_32: feature_name=ENST00000308478 | feature_id[1].value > threshold=6.604248523712158 |
| Class: current_smokers | |
| | |
| Rules_9 | passed counts:21 |
| node_0: feature_name=ENST00000284311 | feature_id[0].value > threshold=179.44091796875 |
| node_102: feature_name=ENST00000316418 | feature_id[2].value > threshold=3.560240387916565 |
| node_136: feature_name=ENST00000380672 | feature_id[32].value > threshold=31.704580307006836 |
| node_184: feature_name=ENST00000339223 | feature_id[13].value <= threshold=37.65940284729004 |
| node_185: feature_name=ENST00000341184 | feature_id[19].value <= threshold=27.130505561828613 |
| node_186: feature_name=ENST00000522551 | feature_id[60].value <= threshold=45.17411804199219 |
| node_187: feature_name=ENST00000392040 | feature_id[61].value > threshold=24.49085807800293 |
| Class: former_smokers | |
| | |
| Rules_10 | passed counts:21 |
| node_0: feature_name=ENST00000284311 | feature_id[0].value > threshold=179.44091796875 |
| node_102: feature_name=ENST00000316418 | feature_id[2].value <= threshold=3.560240387916565 |
| node_103: feature_name=ENST00000522551 | feature_id[60].value <= threshold=57.16539001464844 |
| node_104: feature_name=ENST00000367467 | feature_id[9].value > threshold=43.146501541137695 |
| node_112: feature_name=ENST00000339223 | feature_id[13].value > threshold=13.673684120178223 |
| node_114: feature_name=ENST00000380672 | feature_id[32].value <= threshold=51.63601493835449 |

| | |
|---|---|
| node_115: feature_name=ENST00000464162 | feature_id[45].value <= threshold=23.323281288146973 |
| node_116: feature_name=ENST00000396625 | feature_id[33].value > threshold=14.464738368988037 |
| Class: current_smokers | |
| | |
| Rules_11 | passed counts:21 |
| node_0: feature_name=ENST00000284311 | feature_id[0].value <= threshold=179.44091796875 |
| node_1: feature_name=ENST00000390539 | feature_id[5].value <= threshold=11.91484260559082 |
| node_2: feature_name=ENST00000586582 | feature_id[3].value <= threshold=17.368224143981934 |
| node_3: feature_name=ENST00000280258 | feature_id[56].value > threshold=75.14949798583984 |
| node_39: feature_name=ENST00000438425 | feature_id[40].value <= threshold=37.27690362930298 |
| node_40: feature_name=ENST00000393590 | feature_id[57].value <= threshold=16.18181800842285 |
| node_41: feature_name=ENST00000316418 | feature_id[2].value <= threshold=7.755321025848389 |
| node_42: feature_name=ENST00000390606 | feature_id[27].value <= threshold=15.668272972106934 |
| node_43: feature_name=ENST00000441556 | feature_id[24].value <= threshold=9.982987880706787 |
| node_44: feature_name=ENST00000610495 | feature_id[53].value > threshold=8.27418327331543 |
| node_48: feature_name=ENST00000441556 | feature_id[24].value > threshold=5.98947286605835 |
| node_54: feature_name=ENST00000464162 | feature_id[45].value <= threshold=10.004804372787476 |
| node_55: feature_name=ENST00000390319 | feature_id[59].value > threshold=4.869982719421387 |
| Class: former_smokers | |
| | |
| Rules_12 | passed counts:20 |
| node_0: feature_name=ENST00000284311 | feature_id[0].value <= threshold=179.44091796875 |
| node_1: feature_name=ENST00000390539 | feature_id[5].value <= threshold=11.91484260559082 |
| node_2: feature_name=ENST00000586582 | feature_id[3].value <= threshold=17.368224143981934 |
| node_3: feature_name=ENST00000280258 | feature_id[56].value <= threshold=75.14949798583984 |
| node_4: feature_name=ENST00000284311 | feature_id[0].value > threshold=100.91744613647461 |

| | |
|---|---|
| node_24: feature_name=ENST00000367467 | feature_id[9].value <= threshold=35.77326202392578 |
| node_25: feature_name=ENST00000360851 | feature_id[47].value <= threshold=13.60571002960205 |
| node_26: feature_name=ENST00000523272 | feature_id[20].value <= threshold=2.3025037050247192 |
| Class: former_smokers | |
| | |
| Rules_13 | passed counts:16 |
| node_0: feature_name=ENST00000284311 | feature_id[0].value > threshold=179.44091796875 |
| node_102: feature_name=ENST00000316418 | feature_id[2].value > threshold=3.560240387916565 |
| node_136: feature_name=ENST00000380672 | feature_id[32].value <= threshold=31.704580307006836 |
| node_137: feature_name=ENST00000308478 | feature_id[1].value <= threshold=50.736541748046875 |
| node_138: feature_name=ENST00000610495 | feature_id[53].value <= threshold=247.7474594116211 |
| node_139: feature_name=ENST00000367929 | feature_id[58].value <= threshold=112.91791152954102 |
| node_140: feature_name=ENST00000390290 | feature_id[46].value <= threshold=23.118605613708496 |
| node_141: feature_name=ENST00000308478 | feature_id[1].value <= threshold=40.582645416259766 |
| node_142: feature_name=ENST00000244174 | feature_id[10].value <= threshold=14.476964473724365 |
| node_143: feature_name=ENST00000390549 | feature_id[28].value > threshold=15.814601421356201 |
| Class: current_smokers | |
| | |
| Rules_14 | passed counts:16 |
| node_0: feature_name=ENST00000284311 | feature_id[0].value <= threshold=179.44091796875 |
| node_1: feature_name=ENST00000390539 | feature_id[5].value > threshold=11.91484260559082 |
| node_79: feature_name=ENST00000522551 | feature_id[60].value > threshold=95.3083724975586 |
| node_95: feature_name=ENST00000487272 | feature_id[8].value <= threshold=20.848535537719727 |
| node_96: feature_name=ENST00000367434 | feature_id[21].value > threshold=8.783787488937378 |
| Class: former_smokers | |
| | |

| | |
|---|---|
| Rules_15 | passed counts:16 |
| node_0: feature_name=ENST00000284311 | feature_id[0].value <= threshold=179.44091796875 |
| node_1: feature_name=ENST00000390539 | feature_id[5].value > threshold=11.91484260559082 |
| node_79: feature_name=ENST00000522551 | feature_id[60].value <= threshold=95.3083724975586 |
| node_80: feature_name=ENST00000610495 | feature_id[53].value <= threshold=54.46980857849121 |
| node_81: feature_name=ENST00000284311 | feature_id[0].value > threshold=45.77398490905762 |
| Class: current_smokers | |
| | |
| Rules_16 | passed counts:11 |
| node_0: feature_name=ENST00000284311 | feature_id[0].value > threshold=179.44091796875 |
| node_102: feature_name=ENST00000316418 | feature_id[2].value > threshold=3.560240387916565 |
| node_136: feature_name=ENST00000380672 | feature_id[32].value > threshold=31.704580307006836 |
| node_184: feature_name=ENST00000339223 | feature_id[13].value <= threshold=37.65940284729004 |
| node_185: feature_name=ENST00000341184 | feature_id[19].value > threshold=27.130505561828613 |
| node_193: feature_name=ENST00000464162 | feature_id[45].value <= threshold=10.271081447601318 |
| node_194: feature_name=ENST00000634222 | feature_id[36].value <= threshold=73.65023803710938 |
| Class: current_smokers | |
| | |
| Rules_17 | passed counts:11 |
| node_0: feature_name=ENST00000284311 | feature_id[0].value <= threshold=179.44091796875 |
| node_1: feature_name=ENST00000390539 | feature_id[5].value <= threshold=11.91484260559082 |
| node_2: feature_name=ENST00000586582 | feature_id[3].value > threshold=17.368224143981934 |
| node_74: feature_name=ENST00000316418 | feature_id[2].value > threshold=3.9801650047302246 |
| Class: current_smokers | |
| | |
| Rules_18 | passed counts:10 |

| | |
|---|---|
| node_0: feature_name=ENST00000284311 | feature_id[0].value > threshold=179.44091796875 |
| node_102: feature_name=ENST00000316418 | feature_id[2].value > threshold=3.560240387916565 |
| node_136: feature_name=ENST00000380672 | feature_id[32].value <= threshold=31.704580307006836 |
| node_137: feature_name=ENST00000308478 | feature_id[1].value > threshold=50.736541748046875 |
| node_155: feature_name=ENST00000390309 | feature_id[41].value > threshold=8.32264518737793 |
| node_159: feature_name=ENST00000580335 | feature_id[63].value > threshold=1.7440648078918457 |
| node_161: feature_name=ENST00000422622 | feature_id[52].value <= threshold=76.81717681884766 |
| node_162: feature_name=ENST00000634222 | feature_id[36].value > threshold=1.5202934741973877 |
| node_164: feature_name=ENST00000393590 | feature_id[57].value <= threshold=15.50696325302124 |
| node_165: feature_name=ENST00000438425 | feature_id[40].value <= threshold=38.6440544128418 |
| node_166: feature_name=ENST00000367434 | feature_id[21].value <= threshold=178.1705780029297 |
| node_167: feature_name=ENST00000441556 | feature_id[24].value <= threshold=20.893842697143555 |
| node_168: feature_name=ENST00000280258 | feature_id[56].value > threshold=250.8539047241211 |
| node_170: feature_name=ENST00000483158 | feature_id[43].value > threshold=57.99545478820801 |
| Class: current_smokers | |
| | |
| Rules_19 | passed counts:10 |
| node_0: feature_name=ENST00000284311 | feature_id[0].value <= threshold=179.44091796875 |
| node_1: feature_name=ENST00000390539 | feature_id[5].value <= threshold=11.91484260559082 |
| node_2: feature_name=ENST00000586582 | feature_id[3].value <= threshold=17.368224143981934 |
| node_3: feature_name=ENST00000280258 | feature_id[56].value > threshold=75.14949798583984 |
| node_39: feature_name=ENST00000438425 | feature_id[40].value <= threshold=37.27690362930298 |
| node_40: feature_name=ENST00000393590 | feature_id[57].value <= threshold=16.18181800842285 |
| node_41: feature_name=ENST00000316418 | feature_id[2].value <= threshold=7.755321025848389 |
| node_42: feature_name=ENST00000390606 | feature_id[27].value <= threshold=15.668272972106934 |

| | |
|---|---|
| node_43: feature_name=ENST00000441556 | feature_id[24].value > threshold=9.982987880706787 |
| node_59: feature_name=ENST00000586582 | feature_id[3].value <= threshold=4.869513511657715 |
| Class: former_smokers | |
| | |
| Rules_20 | passed counts:9 |
| node_0: feature_name=ENST00000284311 | feature_id[0].value > threshold=179.44091796875 |
| node_102: feature_name=ENST00000316418 | feature_id[2].value > threshold=3.560240387916565 |
| node_136: feature_name=ENST00000380672 | feature_id[32].value > threshold=31.704580307006836 |
| node_184: feature_name=ENST00000339223 | feature_id[13].value <= threshold=37.65940284729004 |
| node_185: feature_name=ENST00000341184 | feature_id[19].value > threshold=27.130505561828613 |
| node_193: feature_name=ENST00000464162 | feature_id[45].value > threshold=10.271081447601318 |
| node_197: feature_name=ENST00000244174 | feature_id[10].value <= threshold=10.046055316925049 |
| Class: former_smokers | |
| | |
| Rules_21 | passed counts:9 |
| node_0: feature_name=ENST00000284311 | feature_id[0].value <= threshold=179.44091796875 |
| node_1: feature_name=ENST00000390539 | feature_id[5].value > threshold=11.91484260559082 |
| node_79: feature_name=ENST00000522551 | feature_id[60].value <= threshold=95.3083724975586 |
| node_80: feature_name=ENST00000610495 | feature_id[53].value > threshold=54.46980857849121 |
| node_86: feature_name=ENST00000451085 | feature_id[7].value <= threshold=6.878771543502808 |
| Class: former_smokers | |
| | |
| Rules_22 | passed counts:8 |
| node_0: feature_name=ENST00000284311 | feature_id[0].value > threshold=179.44091796875 |
| node_102: feature_name=ENST00000316418 | feature_id[2].value <= threshold=3.560240387916565 |
| node_103: feature_name=ENST00000522551 | feature_id[60].value <= threshold=57.16539001464844 |

| | |
|---|---|
| node_104: feature_name=ENST00000367467 | feature_id[9].value > threshold=43.146501541137695 |
| node_112: feature_name=ENST00000339223 | feature_id[13].value <= threshold=13.673684120178223 |
| Class: former_smokers | |
| | |
| Rules_23 | passed counts:8 |
| node_0: feature_name=ENST00000284311 | feature_id[0].value <= threshold=179.44091796875 |
| node_1: feature_name=ENST00000390539 | feature_id[5].value > threshold=11.91484260559082 |
| node_79: feature_name=ENST00000522551 | feature_id[60].value <= threshold=95.3083724975586 |
| node_80: feature_name=ENST00000610495 | feature_id[53].value > threshold=54.46980857849121 |
| node_86: feature_name=ENST00000451085 | feature_id[7].value > threshold=6.878771543502808 |
| node_88: feature_name=ENST00000622663 | feature_id[51].value > threshold=3.760047197341919 |
| node_92: feature_name=ENST00000284311 | feature_id[0].value > threshold=81.37762069702148 |
| Class: current_smokers | |
| | |
| Rules_24 | passed counts:8 |
| node_0: feature_name=ENST00000284311 | feature_id[0].value <= threshold=179.44091796875 |
| node_1: feature_name=ENST00000390539 | feature_id[5].value <= threshold=11.91484260559082 |
| node_2: feature_name=ENST00000586582 | feature_id[3].value <= threshold=17.368224143981934 |
| node_3: feature_name=ENST00000280258 | feature_id[56].value > threshold=75.14949798583984 |
| node_39: feature_name=ENST00000438425 | feature_id[40].value <= threshold=37.27690362930298 |
| node_40: feature_name=ENST00000393590 | feature_id[57].value <= threshold=16.18181800842285 |
| node_41: feature_name=ENST00000316418 | feature_id[2].value <= threshold=7.755321025848389 |
| node_42: feature_name=ENST00000390606 | feature_id[27].value <= threshold=15.668272972106934 |
| node_43: feature_name=ENST00000441556 | feature_id[24].value <= threshold=9.982987880706787 |
| node_44: feature_name=ENST00000610495 | feature_id[53].value > threshold=8.27418327331543 |
| node_48: feature_name=ENST00000441556 | feature_id[24].value <= threshold=5.98947286605835 |

| | |
|---|---|
| node_49: feature_name=ENST00000284311 | feature_id[0].value > threshold=173.78553009033203 |
| node_51: feature_name=ENST00000464162 | feature_id[45].value > threshold=0.546977698802948 |
| Class: former_smokers | |
| | |
| Rules_25 | passed counts:7 |
| node_0: feature_name=ENST00000284311 | feature_id[0].value > threshold=179.44091796875 |
| node_102: feature_name=ENST00000316418 | feature_id[2].value > threshold=3.560240387916565 |
| node_136: feature_name=ENST00000380672 | feature_id[32].value <= threshold=31.704580307006836 |
| node_137: feature_name=ENST00000308478 | feature_id[1].value <= threshold=50.736541748046875 |
| node_138: feature_name=ENST00000610495 | feature_id[53].value <= threshold=247.7474594116211 |
| node_139: feature_name=ENST00000367929 | feature_id[58].value <= threshold=112.91791152954102 |
| node_140: feature_name=ENST00000390290 | feature_id[46].value <= threshold=23.118605613708496 |
| node_141: feature_name=ENST00000308478 | feature_id[1].value > threshold=40.582645416259766 |
| node_147: feature_name=ENST00000483158 | feature_id[43].value > threshold=42.28260803222656 |
| Class: former_smokers | |
| | |
| Rules_26 | passed counts:7 |
| node_0: feature_name=ENST00000284311 | feature_id[0].value > threshold=179.44091796875 |
| node_102: feature_name=ENST00000316418 | feature_id[2].value <= threshold=3.560240387916565 |
| node_103: feature_name=ENST00000522551 | feature_id[60].value > threshold=57.16539001464844 |
| node_125: feature_name=ENST00000390323 | feature_id[22].value > threshold=204.28018188476562 |
| node_127: feature_name=ENST00000280258 | feature_id[56].value > threshold=121.94679641723633 |
| node_129: feature_name=ENST00000396625 | feature_id[33].value > threshold=28.375680923461914 |
| node_133: feature_name=ENST00000390237 | feature_id[54].value <= threshold=5609.633544921875 |
| Class: current_smokers | |
| | |

| | |
|---|---|
| Rules_27 | passed counts:7 |
| node_0: feature_name=ENST00000284311 | feature_id[0].value > threshold=179.44091796875 |
| node_102: feature_name=ENST00000316418 | feature_id[2].value <= threshold=3.560240387916565 |
| node_103: feature_name=ENST00000522551 | feature_id[60].value <= threshold=57.16539001464844 |
| node_104: feature_name=ENST00000367467 | feature_id[9].value > threshold=43.146501541137695 |
| node_112: feature_name=ENST00000339223 | feature_id[13].value > threshold=13.673684120178223 |
| node_114: feature_name=ENST00000380672 | feature_id[32].value > threshold=51.63601493835449 |
| node_122: feature_name=ENST00000390319 | feature_id[59].value <= threshold=108.81984329223633 |
| Class: former_smokers | |
| | |
| Rules_28 | passed counts:6 |
| node_0: feature_name=ENST00000284311 | feature_id[0].value > threshold=179.44091796875 |
| node_102: feature_name=ENST00000316418 | feature_id[2].value > threshold=3.560240387916565 |
| node_136: feature_name=ENST00000380672 | feature_id[32].value > threshold=31.704580307006836 |
| node_184: feature_name=ENST00000339223 | feature_id[13].value > threshold=37.65940284729004 |
| node_200: feature_name=ENST00000359228 | feature_id[31].value > threshold=14.81732177734375 |
| node_212: feature_name=ENST00000339223 | feature_id[13].value > threshold=46.98575019836426 |
| Class: former_smokers | |
| | |
| Rules_29 | passed counts:6 |
| node_0: feature_name=ENST00000284311 | feature_id[0].value > threshold=179.44091796875 |
| node_102: feature_name=ENST00000316418 | feature_id[2].value > threshold=3.560240387916565 |
| node_136: feature_name=ENST00000380672 | feature_id[32].value <= threshold=31.704580307006836 |
| node_137: feature_name=ENST00000308478 | feature_id[1].value <= threshold=50.736541748046875 |
| node_138: feature_name=ENST00000610495 | feature_id[53].value > threshold=247.7474594116211 |
| Class: former_smokers | |

| | |
|---|---|
| Rules_30 | passed counts:6 |
| node_0: feature_name=ENST00000284311 | feature_id[0].value > threshold=179.44091796875 |
| node_102: feature_name=ENST00000316418 | feature_id[2].value > threshold=3.560240387916565 |
| node_136: feature_name=ENST00000380672 | feature_id[32].value <= threshold=31.704580307006836 |
| node_137: feature_name=ENST00000308478 | feature_id[1].value <= threshold=50.736541748046875 |
| node_138: feature_name=ENST00000610495 | feature_id[53].value <= threshold=247.7474594116211 |
| node_139: feature_name=ENST00000367929 | feature_id[58].value > threshold=112.91791152954102 |
| node_151: feature_name=ENST00000367434 | feature_id[21].value > threshold=33.11489295959473 |
| Class: former_smokers | |
| | |
| Rules_31 | passed counts:6 |
| node_0: feature_name=ENST00000284311 | feature_id[0].value > threshold=179.44091796875 |
| node_102: feature_name=ENST00000316418 | feature_id[2].value <= threshold=3.560240387916565 |
| node_103: feature_name=ENST00000522551 | feature_id[60].value > threshold=57.16539001464844 |
| node_125: feature_name=ENST00000390323 | feature_id[22].value > threshold=204.28018188476562 |
| node_127: feature_name=ENST00000280258 | feature_id[56].value > threshold=121.94679641723633 |
| node_129: feature_name=ENST00000396625 | feature_id[33].value <= threshold=28.375680923461914 |
| node_130: feature_name=ENST00000390252 | feature_id[25].value > threshold=59.50105094909668 |
| Class: former_smokers | |
| | |
| Rules_32 | passed counts:6 |
| node_0: feature_name=ENST00000284311 | feature_id[0].value <= threshold=179.44091796875 |
| node_1: feature_name=ENST00000390539 | feature_id[5].value <= threshold=11.91484260559082 |
| node_2: feature_name=ENST00000586582 | feature_id[3].value <= threshold=17.368224143981934 |
| node_3: feature_name=ENST00000280258 | feature_id[56].value <= threshold=75.14949798583984 |

| | |
|---|---|
| node_4: feature_name=ENST00000284311 | feature_id[0].value <= threshold=100.91744613647461 |
| node_5: feature_name=ENST00000492167 | feature_id[23].value <= threshold=164.934326171875 |
| node_6: feature_name=ENST00000616417 | feature_id[39].value > threshold=0.00022576878836844116 |
| node_8: feature_name=ENST00000276974 | feature_id[49].value <= threshold=19.783329486846924 |
| node_9: feature_name=ENST00000636279 | feature_id[48].value <= threshold=8.218245029449463 |
| node_10: feature_name=ENST00000464835 | feature_id[6].value <= threshold=17.562889099121094 |
| node_11: feature_name=ENST00000390323 | feature_id[22].value <= threshold=50.83455848693848 |
| node_12: feature_name=ENST00000473726 | feature_id[26].value <= threshold=11.481990814208984 |
| Class: former_smokers | |
| | |
| Rules_33 | passed counts:5 |
| node_0: feature_name=ENST00000284311 | feature_id[0].value > threshold=179.44091796875 |
| node_102: feature_name=ENST00000316418 | feature_id[2].value <= threshold=3.560240387916565 |
| node_103: feature_name=ENST00000522551 | feature_id[60].value > threshold=57.16539001464844 |
| node_125: feature_name=ENST00000390323 | feature_id[22].value <= threshold=204.28018188476562 |
| Class: former_smokers | |
| | |
| Rules_34 | passed counts:5 |
| node_0: feature_name=ENST00000284311 | feature_id[0].value <= threshold=179.44091796875 |
| node_1: feature_name=ENST00000390539 | feature_id[5].value > threshold=11.91484260559082 |
| node_79: feature_name=ENST00000522551 | feature_id[60].value <= threshold=95.3083724975586 |
| node_80: feature_name=ENST00000610495 | feature_id[53].value > threshold=54.46980857849121 |
| node_86: feature_name=ENST00000451085 | feature_id[7].value > threshold=6.878771543502808 |
| node_88: feature_name=ENST00000622663 | feature_id[51].value <= threshold=3.760047197341919 |
| node_89: feature_name=ENST00000339223 | feature_id[13].value <= threshold=28.996588706970215 |
| Class: former_smokers | |

| | |
|---|---|
| | |
| Rules_35 | passed counts:5 |
| node_0: feature_name=ENST00000284311 | feature_id[0].value <= threshold=179.44091796875 |
| node_1: feature_name=ENST00000390539 | feature_id[5].value <= threshold=11.91484260559082 |
| node_2: feature_name=ENST00000586582 | feature_id[3].value <= threshold=17.368224143981934 |
| node_3: feature_name=ENST00000280258 | feature_id[56].value > threshold=75.14949798583984 |
| node_39: feature_name=ENST00000438425 | feature_id[40].value <= threshold=37.27690362930298 |
| node_40: feature_name=ENST00000393590 | feature_id[57].value <= threshold=16.18181800842285 |
| node_41: feature_name=ENST00000316418 | feature_id[2].value > threshold=7.755321025848389 |
| node_65: feature_name=ENST00000244174 | feature_id[10].value > threshold=3.6251673698425293 |
| node_69: feature_name=ENST00000394329 | feature_id[55].value > threshold=39.62116050720215 |
| Class: former_smokers | |
| | |
| Rules_36 | passed counts:5 |
| node_0: feature_name=ENST00000284311 | feature_id[0].value <= threshold=179.44091796875 |
| node_1: feature_name=ENST00000390539 | feature_id[5].value <= threshold=11.91484260559082 |
| node_2: feature_name=ENST00000586582 | feature_id[3].value <= threshold=17.368224143981934 |
| node_3: feature_name=ENST00000280258 | feature_id[56].value > threshold=75.14949798583984 |
| node_39: feature_name=ENST00000438425 | feature_id[40].value <= threshold=37.27690362930298 |
| node_40: feature_name=ENST00000393590 | feature_id[57].value <= threshold=16.18181800842285 |
| node_41: feature_name=ENST00000316418 | feature_id[2].value > threshold=7.755321025848389 |
| node_65: feature_name=ENST00000244174 | feature_id[10].value > threshold=3.6251673698425293 |
| node_69: feature_name=ENST00000394329 | feature_id[55].value <= threshold=39.62116050720215 |
| Class: current_smokers | |
| | |
| Rules_37 | passed counts:5 |

| | |
|---|---|
| node_0: feature_name=ENST00000284311 | feature_id[0].value <= threshold=179.44091796875 |
| node_1: feature_name=ENST00000390539 | feature_id[5].value <= threshold=11.91484260559082 |
| node_2: feature_name=ENST00000586582 | feature_id[3].value <= threshold=17.368224143981934 |
| node_3: feature_name=ENST00000280258 | feature_id[56].value <= threshold=75.14949798583984 |
| node_4: feature_name=ENST00000284311 | feature_id[0].value > threshold=100.91744613647461 |
| node_24: feature_name=ENST00000367467 | feature_id[9].value > threshold=35.77326202392578 |
| node_30: feature_name=ENST00000616417 | feature_id[39].value <= threshold=164.16122436523438 |
| node_31: feature_name=ENST00000359228 | feature_id[31].value > threshold=8.815360069274902 |
| node_35: feature_name=ENST00000390323 | feature_id[22].value > threshold=79.46425247192383 |
| Class: former_smokers | |
| | |
| Rules_38 | passed counts:4 |
| node_0: feature_name=ENST00000284311 | feature_id[0].value > threshold=179.44091796875 |
| node_102: feature_name=ENST00000316418 | feature_id[2].value > threshold=3.560240387916565 |
| node_136: feature_name=ENST00000380672 | feature_id[32].value <= threshold=31.704580307006836 |
| node_137: feature_name=ENST00000308478 | feature_id[1].value > threshold=50.736541748046875 |
| node_155: feature_name=ENST00000390309 | feature_id[41].value <= threshold=8.32264518737793 |
| node_156: feature_name=ENST00000610495 | feature_id[53].value <= threshold=142.00611877441406 |
| Class: current_smokers | |
| | |
| Rules_39 | passed counts:4 |
| node_0: feature_name=ENST00000284311 | feature_id[0].value <= threshold=179.44091796875 |
| node_1: feature_name=ENST00000390539 | feature_id[5].value <= threshold=11.91484260559082 |
| node_2: feature_name=ENST00000586582 | feature_id[3].value > threshold=17.368224143981934 |
| node_74: feature_name=ENST00000316418 | feature_id[2].value <= threshold=3.9801650047302246 |
| node_75: feature_name=ENST00000308478 | feature_id[1].value > threshold=9.707509994506836 |

| Class: former_smokers | |
|---|---|
| | |
| Rules_40 | passed counts:4 |
| node_0: feature_name=ENST00000284311 | feature_id[0].value <= threshold=179.44091796875 |
| node_1: feature_name=ENST00000390539 | feature_id[5].value <= threshold=11.91484260559082 |
| node_2: feature_name=ENST00000586582 | feature_id[3].value <= threshold=17.368224143981934 |
| node_3: feature_name=ENST00000280258 | feature_id[56].value > threshold=75.14949798583984 |
| node_39: feature_name=ENST00000438425 | feature_id[40].value <= threshold=37.27690362930298 |
| node_40: feature_name=ENST00000393590 | feature_id[57].value <= threshold=16.18181800842285 |
| node_41: feature_name=ENST00000316418 | feature_id[2].value <= threshold=7.755321025848389 |
| node_42: feature_name=ENST00000390606 | feature_id[27].value > threshold=15.668272972106934 |
| node_62: feature_name=ENST00000280258 | feature_id[56].value > threshold=138.78301239013672 |
| Class: former_smokers | |
| | |
| Rules_41 | passed counts:4 |
| node_0: feature_name=ENST00000284311 | feature_id[0].value <= threshold=179.44091796875 |
| node_1: feature_name=ENST00000390539 | feature_id[5].value <= threshold=11.91484260559082 |
| node_2: feature_name=ENST00000586582 | feature_id[3].value <= threshold=17.368224143981934 |
| node_3: feature_name=ENST00000280258 | feature_id[56].value <= threshold=75.14949798583984 |
| node_4: feature_name=ENST00000284311 | feature_id[0].value > threshold=100.91744613647461 |
| node_24: feature_name=ENST00000367467 | feature_id[9].value > threshold=35.77326202392578 |
| node_30: feature_name=ENST00000616417 | feature_id[39].value > threshold=164.16122436523438 |
| Class: former_smokers | |
| | |
| Rules_42 | passed counts:4 |
| node_0: feature_name=ENST00000284311 | feature_id[0].value <= threshold=179.44091796875 |

| | |
|---|---|
| node_1: feature_name=ENST00000390539 | feature_id[5].value <= threshold=11.91484260559082 |
| node_2: feature_name=ENST00000586582 | feature_id[3].value <= threshold=17.368224143981934 |
| node_3: feature_name=ENST00000280258 | feature_id[56].value <= threshold=75.14949798583984 |
| node_4: feature_name=ENST00000284311 | feature_id[0].value <= threshold=100.91744613647461 |
| node_5: feature_name=ENST00000492167 | feature_id[23].value > threshold=164.934326171875 |
| Class: current_smokers | |
| | |
| Rules_43 | passed counts:3 |
| node_0: feature_name=ENST00000284311 | feature_id[0].value > threshold=179.44091796875 |
| node_102: feature_name=ENST00000316418 | feature_id[2].value > threshold=3.560240387916565 |
| node_136: feature_name=ENST00000380672 | feature_id[32].value > threshold=31.704580307006836 |
| node_184: feature_name=ENST00000339223 | feature_id[13].value > threshold=37.65940284729004 |
| node_200: feature_name=ENST00000359228 | feature_id[31].value <= threshold=14.81732177734375 |
| node_201: feature_name=ENST00000464835 | feature_id[6].value <= threshold=3.9680298566818237 |
| node_202: feature_name=ENST00000316418 | feature_id[2].value <= threshold=13.935326099395752 |
| Class: former_smokers | |
| | |
| Rules_44 | passed counts:3 |
| node_0: feature_name=ENST00000284311 | feature_id[0].value > threshold=179.44091796875 |
| node_102: feature_name=ENST00000316418 | feature_id[2].value > threshold=3.560240387916565 |
| node_136: feature_name=ENST00000380672 | feature_id[32].value <= threshold=31.704580307006836 |
| node_137: feature_name=ENST00000308478 | feature_id[1].value > threshold=50.736541748046875 |
| node_155: feature_name=ENST00000390309 | feature_id[41].value <= threshold=8.32264518737793 |
| node_156: feature_name=ENST00000610495 | feature_id[53].value > threshold=142.00611877441406 |
| Class: former_smokers | |
| | |

| | |
|---|---|
| Rules_45 | passed counts:3 |
| node_0: feature_name=ENST00000284311 | feature_id[0].value > threshold=179.44091796875 |
| node_102: feature_name=ENST00000316418 | feature_id[2].value > threshold=3.560240387916565 |
| node_136: feature_name=ENST00000380672 | feature_id[32].value <= threshold=31.704580307006836 |
| node_137: feature_name=ENST00000308478 | feature_id[1].value <= threshold=50.736541748046875 |
| node_138: feature_name=ENST00000610495 | feature_id[53].value <= threshold=247.7474594116211 |
| node_139: feature_name=ENST00000367929 | feature_id[58].value > threshold=112.91791152954102 |
| node_151: feature_name=ENST00000367434 | feature_id[21].value <= threshold=33.11489295959473 |
| Class: current_smokers | |
| | |
| Rules_46 | passed counts:3 |
| node_0: feature_name=ENST00000284311 | feature_id[0].value > threshold=179.44091796875 |
| node_102: feature_name=ENST00000316418 | feature_id[2].value <= threshold=3.560240387916565 |
| node_103: feature_name=ENST00000522551 | feature_id[60].value <= threshold=57.16539001464844 |
| node_104: feature_name=ENST00000367467 | feature_id[9].value > threshold=43.146501541137695 |
| node_112: feature_name=ENST00000339223 | feature_id[13].value > threshold=13.673684120178223 |
| node_114: feature_name=ENST00000380672 | feature_id[32].value <= threshold=51.63601493835449 |
| node_115: feature_name=ENST00000464162 | feature_id[45].value <= threshold=23.323281288146973 |
| node_116: feature_name=ENST00000396625 | feature_id[33].value <= threshold=14.464738368988037 |
| node_117: feature_name=ENST00000547327 | feature_id[35].value > threshold=3.7538559436798096 |
| Class: former_smokers | |
| | |
| Rules_47 | passed counts:3 |
| node_0: feature_name=ENST00000284311 | feature_id[0].value > threshold=179.44091796875 |
| node_102: feature_name=ENST00000316418 | feature_id[2].value <= threshold=3.560240387916565 |
| node_103: feature_name=ENST00000522551 | feature_id[60].value <= threshold=57.16539001464844 |

| | |
|---|---|
| node_104: feature_name=ENST00000367467 | feature_id[9].value <= threshold=43.146501541137695 |
| node_105: feature_name=ENST00000480786 | feature_id[62].value > threshold=3.7895997762680054 |
| node_109: feature_name=ENST00000390252 | feature_id[25].value <= threshold=82.72151565551758 |
| Class: current_smokers | |
| | |
| Rules_48 | passed counts:3 |
| node_0: feature_name=ENST00000284311 | feature_id[0].value <= threshold=179.44091796875 |
| node_1: feature_name=ENST00000390539 | feature_id[5].value > threshold=11.91484260559082 |
| node_79: feature_name=ENST00000522551 | feature_id[60].value > threshold=95.3083724975586 |
| node_95: feature_name=ENST00000487272 | feature_id[8].value > threshold=20.848535537719727 |
| node_99: feature_name=ENST00000280258 | feature_id[56].value <= threshold=249.81621551513672 |
| Class: current_smokers | |
| | |
| Rules_49 | passed counts:3 |
| node_0: feature_name=ENST00000284311 | feature_id[0].value <= threshold=179.44091796875 |
| node_1: feature_name=ENST00000390539 | feature_id[5].value <= threshold=11.91484260559082 |
| node_2: feature_name=ENST00000586582 | feature_id[3].value <= threshold=17.368224143981934 |
| node_3: feature_name=ENST00000280258 | feature_id[56].value > threshold=75.14949798583984 |
| node_39: feature_name=ENST00000438425 | feature_id[40].value <= threshold=37.27690362930298 |
| node_40: feature_name=ENST00000393590 | feature_id[57].value <= threshold=16.18181800842285 |
| node_41: feature_name=ENST00000316418 | feature_id[2].value <= threshold=7.755321025848389 |
| node_42: feature_name=ENST00000390606 | feature_id[27].value <= threshold=15.668272972106934 |
| node_43: feature_name=ENST00000441556 | feature_id[24].value > threshold=9.982987880706787 |
| node_59: feature_name=ENST00000586582 | feature_id[3].value > threshold=4.869513511657715 |
| Class: current_smokers | |
| | |

| | |
|---|---|
| Rules_50 | passed counts:3 |
| node_0: feature_name=ENST00000284311 | feature_id[0].value <= threshold=179.44091796875 |
| node_1: feature_name=ENST00000390539 | feature_id[5].value <= threshold=11.91484260559082 |
| node_2: feature_name=ENST00000586582 | feature_id[3].value <= threshold=17.368224143981934 |
| node_3: feature_name=ENST00000280258 | feature_id[56].value <= threshold=75.14949798583984 |
| node_4: feature_name=ENST00000284311 | feature_id[0].value > threshold=100.91744613647461 |
| node_24: feature_name=ENST00000367467 | feature_id[9].value <= threshold=35.77326202392578 |
| node_25: feature_name=ENST00000360851 | feature_id[47].value > threshold=13.60571002960205 |
| Class: current_smokers | |
| | |
| Rules_51 | passed counts:3 |
| node_0: feature_name=ENST00000284311 | feature_id[0].value <= threshold=179.44091796875 |
| node_1: feature_name=ENST00000390539 | feature_id[5].value <= threshold=11.91484260559082 |
| node_2: feature_name=ENST00000586582 | feature_id[3].value <= threshold=17.368224143981934 |
| node_3: feature_name=ENST00000280258 | feature_id[56].value <= threshold=75.14949798583984 |
| node_4: feature_name=ENST00000284311 | feature_id[0].value <= threshold=100.91744613647461 |
| node_5: feature_name=ENST00000492167 | feature_id[23].value <= threshold=164.934326171875 |
| node_6: feature_name=ENST00000616417 | feature_id[39].value > threshold=0.00022576878836844116 |
| node_8: feature_name=ENST00000276974 | feature_id[49].value <= threshold=19.783329486846924 |
| node_9: feature_name=ENST00000636279 | feature_id[48].value <= threshold=8.218245029449463 |
| node_10: feature_name=ENST00000464835 | feature_id[6].value <= threshold=17.562889099121094 |
| node_11: feature_name=ENST00000390323 | feature_id[22].value > threshold=50.83455848693848 |
| node_15: feature_name=ENST00000367467 | feature_id[9].value > threshold=75.31536102294922 |
| node_17: feature_name=ENST00000396625 | feature_id[33].value > threshold=5.065143823623657 |
| Class: former_smokers | |
| | |

| | |
|---|---|
| Rules_52 | passed counts:2 |
| node_0: feature_name=ENST00000284311 | feature_id[0].value > threshold=179.44091796875 |
| node_102: feature_name=ENST00000316418 | feature_id[2].value > threshold=3.560240387916565 |
| node_136: feature_name=ENST00000380672 | feature_id[32].value > threshold=31.704580307006836 |
| node_184: feature_name=ENST00000339223 | feature_id[13].value > threshold=37.65940284729004 |
| node_200: feature_name=ENST00000359228 | feature_id[31].value > threshold=14.81732177734375 |
| node_212: feature_name=ENST00000339223 | feature_id[13].value <= threshold=46.98575019836426 |
| Class: current_smokers | |
| | |
| Rules_53 | passed counts:2 |
| node_0: feature_name=ENST00000284311 | feature_id[0].value > threshold=179.44091796875 |
| node_102: feature_name=ENST00000316418 | feature_id[2].value > threshold=3.560240387916565 |
| node_136: feature_name=ENST00000380672 | feature_id[32].value > threshold=31.704580307006836 |
| node_184: feature_name=ENST00000339223 | feature_id[13].value <= threshold=37.65940284729004 |
| node_185: feature_name=ENST00000341184 | feature_id[19].value <= threshold=27.130505561828613 |
| node_186: feature_name=ENST00000522551 | feature_id[60].value > threshold=45.17411804199219 |
| Class: current_smokers | |
| | |
| Rules_54 | passed counts:2 |
| node_0: feature_name=ENST00000284311 | feature_id[0].value > threshold=179.44091796875 |
| node_102: feature_name=ENST00000316418 | feature_id[2].value > threshold=3.560240387916565 |
| node_136: feature_name=ENST00000380672 | feature_id[32].value <= threshold=31.704580307006836 |
| node_137: feature_name=ENST00000308478 | feature_id[1].value > threshold=50.736541748046875 |
| node_155: feature_name=ENST00000390309 | feature_id[41].value > threshold=8.32264518737793 |
| node_159: feature_name=ENST00000580335 | feature_id[63].value > threshold=1.7440648078918457 |
| node_161: feature_name=ENST00000422622 | feature_id[52].value <= threshold=76.81717681884766 |

| | |
|---|---|
| node_162: feature_name=ENST00000634222 | feature_id[36].value > threshold=1.5202934741973877 |
| node_164: feature_name=ENST00000393590 | feature_id[57].value <= threshold=15.50696325302124 |
| node_165: feature_name=ENST00000438425 | feature_id[40].value <= threshold=38.6440544128418 |
| node_166: feature_name=ENST00000367434 | feature_id[21].value <= threshold=178.1705780029297 |
| node_167: feature_name=ENST00000441556 | feature_id[24].value > threshold=20.893842697143555 |
| node_173: feature_name=ENST00000586582 | feature_id[3].value > threshold=20.026033401489258 |
| Class: current_smokers | |
| | |
| Rules_55 | passed counts:2 |
| node_0: feature_name=ENST00000284311 | feature_id[0].value > threshold=179.44091796875 |
| node_102: feature_name=ENST00000316418 | feature_id[2].value > threshold=3.560240387916565 |
| node_136: feature_name=ENST00000380672 | feature_id[32].value <= threshold=31.704580307006836 |
| node_137: feature_name=ENST00000308478 | feature_id[1].value <= threshold=50.736541748046875 |
| node_138: feature_name=ENST00000610495 | feature_id[53].value <= threshold=247.7474594116211 |
| node_139: feature_name=ENST00000367929 | feature_id[58].value <= threshold=112.91791152954102 |
| node_140: feature_name=ENST00000390290 | feature_id[46].value <= threshold=23.118605613708496 |
| node_141: feature_name=ENST00000308478 | feature_id[1].value > threshold=40.582645416259766 |
| node_147: feature_name=ENST00000483158 | feature_id[43].value <= threshold=42.28260803222656 |
| Class: current_smokers | |
| | |
| Rules_56 | passed counts:2 |
| node_0: feature_name=ENST00000284311 | feature_id[0].value > threshold=179.44091796875 |
| node_102: feature_name=ENST00000316418 | feature_id[2].value <= threshold=3.560240387916565 |
| node_103: feature_name=ENST00000522551 | feature_id[60].value <= threshold=57.16539001464844 |
| node_104: feature_name=ENST00000367467 | feature_id[9].value > threshold=43.146501541137695 |
| node_112: feature_name=ENST00000339223 | feature_id[13].value > threshold=13.673684120178223 |

| | |
|---|---|
| node_114: feature_name=ENST00000380672 | feature_id[32].value <= threshold=51.63601493835449 |
| node_115: feature_name=ENST00000464162 | feature_id[45].value > threshold=23.323281288146973 |
| Class: former_smokers | |
| | |
| Rules_57 | passed counts:2 |
| node_0: feature_name=ENST00000284311 | feature_id[0].value > threshold=179.44091796875 |
| node_102: feature_name=ENST00000316418 | feature_id[2].value <= threshold=3.560240387916565 |
| node_103: feature_name=ENST00000522551 | feature_id[60].value <= threshold=57.16539001464844 |
| node_104: feature_name=ENST00000367467 | feature_id[9].value > threshold=43.146501541137695 |
| node_112: feature_name=ENST00000339223 | feature_id[13].value > threshold=13.673684120178223 |
| node_114: feature_name=ENST00000380672 | feature_id[32].value <= threshold=51.63601493835449 |
| node_115: feature_name=ENST00000464162 | feature_id[45].value <= threshold=23.323281288146973 |
| node_116: feature_name=ENST00000396625 | feature_id[33].value <= threshold=14.464738368988037 |
| node_117: feature_name=ENST00000547327 | feature_id[35].value <= threshold=3.7538559436798096 |
| Class: current_smokers | |
| | |
| Rules_58 | passed counts:2 |
| node_0: feature_name=ENST00000284311 | feature_id[0].value <= threshold=179.44091796875 |
| node_1: feature_name=ENST00000390539 | feature_id[5].value > threshold=11.91484260559082 |
| node_79: feature_name=ENST00000522551 | feature_id[60].value <= threshold=95.3083724975586 |
| node_80: feature_name=ENST00000610495 | feature_id[53].value > threshold=54.46980857849121 |
| node_86: feature_name=ENST00000451085 | feature_id[7].value > threshold=6.878771543502808 |
| node_88: feature_name=ENST00000622663 | feature_id[51].value <= threshold=3.760047197341919 |
| node_89: feature_name=ENST00000339223 | feature_id[13].value > threshold=28.996588706970215 |
| Class: current_smokers | |
| | |

| | |
|---|---|
| Rules_59 | passed counts:2 |
| node_0: feature_name=ENST00000284311 | feature_id[0].value <= threshold=179.44091796875 |
| node_1: feature_name=ENST00000390539 | feature_id[5].value > threshold=11.91484260559082 |
| node_79: feature_name=ENST00000522551 | feature_id[60].value <= threshold=95.3083724975586 |
| node_80: feature_name=ENST00000610495 | feature_id[53].value <= threshold=54.46980857849121 |
| node_81: feature_name=ENST00000284311 | feature_id[0].value <= threshold=45.77398490905762 |
| node_82: feature_name=ENST00000390308 | feature_id[16].value > threshold=10.502037525177002 |
| Class: former_smokers | |
| | |
| Rules_60 | passed counts:2 |
| node_0: feature_name=ENST00000284311 | feature_id[0].value <= threshold=179.44091796875 |
| node_1: feature_name=ENST00000390539 | feature_id[5].value <= threshold=11.91484260559082 |
| node_2: feature_name=ENST00000586582 | feature_id[3].value <= threshold=17.368224143981934 |
| node_3: feature_name=ENST00000280258 | feature_id[56].value > threshold=75.14949798583984 |
| node_39: feature_name=ENST00000438425 | feature_id[40].value <= threshold=37.27690362930298 |
| node_40: feature_name=ENST00000393590 | feature_id[57].value <= threshold=16.18181800842285 |
| node_41: feature_name=ENST00000316418 | feature_id[2].value <= threshold=7.755321025848389 |
| node_42: feature_name=ENST00000390606 | feature_id[27].value > threshold=15.668272972106934 |
| node_62: feature_name=ENST00000280258 | feature_id[56].value <= threshold=138.78301239013672 |
| Class: current_smokers | |
| | |
| Rules_61 | passed counts:2 |
| node_0: feature_name=ENST00000284311 | feature_id[0].value <= threshold=179.44091796875 |
| node_1: feature_name=ENST00000390539 | feature_id[5].value <= threshold=11.91484260559082 |
| node_2: feature_name=ENST00000586582 | feature_id[3].value <= threshold=17.368224143981934 |
| node_3: feature_name=ENST00000280258 | feature_id[56].value <= threshold=75.14949798583984 |

| | |
|---|---|
| node_4: feature_name=ENST00000284311 | feature_id[0].value > threshold=100.91744613647461 |
| node_24: feature_name=ENST00000367467 | feature_id[9].value > threshold=35.77326202392578 |
| node_30: feature_name=ENST00000616417 | feature_id[39].value <= threshold=164.16122436523438 |
| node_31: feature_name=ENST00000359228 | feature_id[31].value <= threshold=8.815360069274902 |
| node_32: feature_name=ENST00000308478 | feature_id[1].value <= threshold=6.604248523712158 |
| Class: former_smokers | |
| | |
| Rules_62 | passed counts:2 |
| node_0: feature_name=ENST00000284311 | feature_id[0].value <= threshold=179.44091796875 |
| node_1: feature_name=ENST00000390539 | feature_id[5].value <= threshold=11.91484260559082 |
| node_2: feature_name=ENST00000586582 | feature_id[3].value <= threshold=17.368224143981934 |
| node_3: feature_name=ENST00000280258 | feature_id[56].value <= threshold=75.14949798583984 |
| node_4: feature_name=ENST00000284311 | feature_id[0].value <= threshold=100.91744613647461 |
| node_5: feature_name=ENST00000492167 | feature_id[23].value <= threshold=164.934326171875 |
| node_6: feature_name=ENST00000616417 | feature_id[39].value > threshold=0.00022576878836844116 |
| node_8: feature_name=ENST00000276974 | feature_id[49].value <= threshold=19.783329486846924 |
| node_9: feature_name=ENST00000636279 | feature_id[48].value <= threshold=8.218245029449463 |
| node_10: feature_name=ENST00000464835 | feature_id[6].value <= threshold=17.562889099121094 |
| node_11: feature_name=ENST00000390323 | feature_id[22].value <= threshold=50.83455848693848 |
| node_12: feature_name=ENST00000473726 | feature_id[26].value > threshold=11.481990814208984 |
| Class: current_smokers | |
| | |
| Rules_63 | passed counts:1 |
| node_0: feature_name=ENST00000284311 | feature_id[0].value > threshold=179.44091796875 |
| node_102: feature_name=ENST00000316418 | feature_id[2].value > threshold=3.560240387916565 |
| node_136: feature_name=ENST00000380672 | feature_id[32].value > threshold=31.704580307006836 |

| | |
|---|---|
| node_184: feature_name=ENST00000339223 | feature_id[13].value > threshold=37.65940284729004 |
| node_200: feature_name=ENST00000359228 | feature_id[31].value <= threshold=14.81732177734375 |
| node_201: feature_name=ENST00000464835 | feature_id[6].value > threshold=3.9680298566818237 |
| node_205: feature_name=ENST00000390305 | feature_id[14].value > threshold=446.3670883178711 |
| Class: former_smokers | |
| | |
| Rules_64 | passed counts:1 |
| node_0: feature_name=ENST00000284311 | feature_id[0].value > threshold=179.44091796875 |
| node_102: feature_name=ENST00000316418 | feature_id[2].value > threshold=3.560240387916565 |
| node_136: feature_name=ENST00000380672 | feature_id[32].value > threshold=31.704580307006836 |
| node_184: feature_name=ENST00000339223 | feature_id[13].value > threshold=37.65940284729004 |
| node_200: feature_name=ENST00000359228 | feature_id[31].value <= threshold=14.81732177734375 |
| node_201: feature_name=ENST00000464835 | feature_id[6].value > threshold=3.9680298566818237 |
| node_205: feature_name=ENST00000390305 | feature_id[14].value <= threshold=446.3670883178711 |
| node_206: feature_name=ENST00000523272 | feature_id[20].value > threshold=15.061781883239746 |
| Class: former_smokers | |
| | |
| Rules_65 | passed counts:1 |
| node_0: feature_name=ENST00000284311 | feature_id[0].value > threshold=179.44091796875 |
| node_102: feature_name=ENST00000316418 | feature_id[2].value > threshold=3.560240387916565 |
| node_136: feature_name=ENST00000380672 | feature_id[32].value > threshold=31.704580307006836 |
| node_184: feature_name=ENST00000339223 | feature_id[13].value > threshold=37.65940284729004 |
| node_200: feature_name=ENST00000359228 | feature_id[31].value <= threshold=14.81732177734375 |
| node_201: feature_name=ENST00000464835 | feature_id[6].value > threshold=3.9680298566818237 |
| node_205: feature_name=ENST00000390305 | feature_id[14].value <= threshold=446.3670883178711 |
| node_206: feature_name=ENST00000523272 | feature_id[20].value <= threshold=15.061781883239746 |

| | |
|---|---|
| node_207: feature_name=ENST00000390285 | feature_id[42].value <= threshold=0.7228579521179199 |
| Class: former_smokers | |
| | |
| Rules_66 | passed counts:1 |
| node_0: feature_name=ENST00000284311 | feature_id[0].value > threshold=179.44091796875 |
| node_102: feature_name=ENST00000316418 | feature_id[2].value > threshold=3.560240387916565 |
| node_136: feature_name=ENST00000380672 | feature_id[32].value > threshold=31.704580307006836 |
| node_184: feature_name=ENST00000339223 | feature_id[13].value > threshold=37.65940284729004 |
| node_200: feature_name=ENST00000359228 | feature_id[31].value <= threshold=14.81732177734375 |
| node_201: feature_name=ENST00000464835 | feature_id[6].value <= threshold=3.9680298566818237 |
| node_202: feature_name=ENST00000316418 | feature_id[2].value > threshold=13.935326099395752 |
| Class: current_smokers | |
| | |
| Rules_67 | passed counts:1 |
| node_0: feature_name=ENST00000284311 | feature_id[0].value > threshold=179.44091796875 |
| node_102: feature_name=ENST00000316418 | feature_id[2].value > threshold=3.560240387916565 |
| node_136: feature_name=ENST00000380672 | feature_id[32].value > threshold=31.704580307006836 |
| node_184: feature_name=ENST00000339223 | feature_id[13].value <= threshold=37.65940284729004 |
| node_185: feature_name=ENST00000341184 | feature_id[19].value > threshold=27.130505561828613 |
| node_193: feature_name=ENST00000464162 | feature_id[45].value > threshold=10.271081447601318 |
| node_197: feature_name=ENST00000244174 | feature_id[10].value > threshold=10.046055316925049 |
| Class: current_smokers | |
| | |
| Rules_68 | passed counts:1 |
| node_0: feature_name=ENST00000284311 | feature_id[0].value > threshold=179.44091796875 |
| node_102: feature_name=ENST00000316418 | feature_id[2].value > threshold=3.560240387916565 |

| | |
|---|---|
| node_136: feature_name=ENST00000380672 | feature_id[32].value > threshold=31.704580307006836 |
| node_184: feature_name=ENST00000339223 | feature_id[13].value <= threshold=37.65940284729004 |
| node_185: feature_name=ENST00000341184 | feature_id[19].value > threshold=27.130505561828613 |
| node_193: feature_name=ENST00000464162 | feature_id[45].value <= threshold=10.271081447601318 |
| node_194: feature_name=ENST00000634222 | feature_id[36].value > threshold=73.65023803710938 |
| Class: former_smokers | |
| | |
| Rules_69 | passed counts:1 |
| node_0: feature_name=ENST00000284311 | feature_id[0].value > threshold=179.44091796875 |
| node_102: feature_name=ENST00000316418 | feature_id[2].value > threshold=3.560240387916565 |
| node_136: feature_name=ENST00000380672 | feature_id[32].value > threshold=31.704580307006836 |
| node_184: feature_name=ENST00000339223 | feature_id[13].value <= threshold=37.65940284729004 |
| node_185: feature_name=ENST00000341184 | feature_id[19].value <= threshold=27.130505561828613 |
| node_186: feature_name=ENST00000522551 | feature_id[60].value <= threshold=45.17411804199219 |
| node_187: feature_name=ENST00000392040 | feature_id[61].value <= threshold=24.49085807800293 |
| node_188: feature_name=ENST00000390549 | feature_id[28].value > threshold=40.540077209472656 |
| Class: current_smokers | |
| | |
| Rules_70 | passed counts:1 |
| node_0: feature_name=ENST00000284311 | feature_id[0].value > threshold=179.44091796875 |
| node_102: feature_name=ENST00000316418 | feature_id[2].value > threshold=3.560240387916565 |
| node_136: feature_name=ENST00000380672 | feature_id[32].value > threshold=31.704580307006836 |
| node_184: feature_name=ENST00000339223 | feature_id[13].value <= threshold=37.65940284729004 |
| node_185: feature_name=ENST00000341184 | feature_id[19].value <= threshold=27.130505561828613 |
| node_186: feature_name=ENST00000522551 | feature_id[60].value <= threshold=45.17411804199219 |
| node_187: feature_name=ENST00000392040 | feature_id[61].value <= threshold=24.49085807800293 |

| | |
|---|---|
| node_188: feature_name=ENST00000390549 | feature_id[28].value <= threshold=40.540077209472656 |
| Class: former_smokers | |
| | |
| Rules_71 | passed counts:1 |
| node_0: feature_name=ENST00000284311 | feature_id[0].value > threshold=179.44091796875 |
| node_102: feature_name=ENST00000316418 | feature_id[2].value > threshold=3.560240387916565 |
| node_136: feature_name=ENST00000380672 | feature_id[32].value <= threshold=31.704580307006836 |
| node_137: feature_name=ENST00000308478 | feature_id[1].value > threshold=50.736541748046875 |
| node_155: feature_name=ENST00000390309 | feature_id[41].value > threshold=8.32264518737793 |
| node_159: feature_name=ENST00000580335 | feature_id[63].value > threshold=1.7440648078918457 |
| node_161: feature_name=ENST00000422622 | feature_id[52].value > threshold=76.81717681884766 |
| Class: former_smokers | |
| | |
| Rules_72 | passed counts:1 |
| node_0: feature_name=ENST00000284311 | feature_id[0].value > threshold=179.44091796875 |
| node_102: feature_name=ENST00000316418 | feature_id[2].value > threshold=3.560240387916565 |
| node_136: feature_name=ENST00000380672 | feature_id[32].value <= threshold=31.704580307006836 |
| node_137: feature_name=ENST00000308478 | feature_id[1].value > threshold=50.736541748046875 |
| node_155: feature_name=ENST00000390309 | feature_id[41].value > threshold=8.32264518737793 |
| node_159: feature_name=ENST00000580335 | feature_id[63].value > threshold=1.7440648078918457 |
| node_161: feature_name=ENST00000422622 | feature_id[52].value <= threshold=76.81717681884766 |
| node_162: feature_name=ENST00000634222 | feature_id[36].value > threshold=1.5202934741973877 |
| node_164: feature_name=ENST00000393590 | feature_id[57].value > threshold=15.50696325302124 |
| Class: former_smokers | |
| | |
| Rules_73 | passed counts:1 |

| | |
|---|---|
| node_0: feature_name=ENST00000284311 | feature_id[0].value > threshold=179.44091796875 |
| node_102: feature_name=ENST00000316418 | feature_id[2].value > threshold=3.560240387916565 |
| node_136: feature_name=ENST00000380672 | feature_id[32].value <= threshold=31.704580307006836 |
| node_137: feature_name=ENST00000308478 | feature_id[1].value > threshold=50.736541748046875 |
| node_155: feature_name=ENST00000390309 | feature_id[41].value > threshold=8.32264518737793 |
| node_159: feature_name=ENST00000580335 | feature_id[63].value > threshold=1.7440648078918457 |
| node_161: feature_name=ENST00000422622 | feature_id[52].value <= threshold=76.81717681884766 |
| node_162: feature_name=ENST00000634222 | feature_id[36].value > threshold=1.5202934741973877 |
| node_164: feature_name=ENST00000393590 | feature_id[57].value <= threshold=15.50696325302124 |
| node_165: feature_name=ENST00000438425 | feature_id[40].value > threshold=38.6440544128418 |
| node_179: feature_name=ENST00000523272 | feature_id[20].value > threshold=5.900441408157349 |
| Class: former_smokers | |
| | |
| Rules_74 | passed counts:1 |
| node_0: feature_name=ENST00000284311 | feature_id[0].value > threshold=179.44091796875 |
| node_102: feature_name=ENST00000316418 | feature_id[2].value > threshold=3.560240387916565 |
| node_136: feature_name=ENST00000380672 | feature_id[32].value <= threshold=31.704580307006836 |
| node_137: feature_name=ENST00000308478 | feature_id[1].value > threshold=50.736541748046875 |
| node_155: feature_name=ENST00000390309 | feature_id[41].value > threshold=8.32264518737793 |
| node_159: feature_name=ENST00000580335 | feature_id[63].value > threshold=1.7440648078918457 |
| node_161: feature_name=ENST00000422622 | feature_id[52].value <= threshold=76.81717681884766 |
| node_162: feature_name=ENST00000634222 | feature_id[36].value > threshold=1.5202934741973877 |
| node_164: feature_name=ENST00000393590 | feature_id[57].value <= threshold=15.50696325302124 |
| node_165: feature_name=ENST00000438425 | feature_id[40].value > threshold=38.6440544128418 |
| node_179: feature_name=ENST00000523272 | feature_id[20].value <= threshold=5.900441408157349 |

| Class: current_smokers | |
|---|---|
| | |
| Rules_75 | passed counts:1 |
| node_0: feature_name=ENST00000284311 | feature_id[0].value > threshold=179.44091796875 |
| node_102: feature_name=ENST00000316418 | feature_id[2].value > threshold=3.560240387916565 |
| node_136: feature_name=ENST00000380672 | feature_id[32].value <= threshold=31.704580307006836 |
| node_137: feature_name=ENST00000308478 | feature_id[1].value > threshold=50.736541748046875 |
| node_155: feature_name=ENST00000390309 | feature_id[41].value > threshold=8.32264518737793 |
| node_159: feature_name=ENST00000580335 | feature_id[63].value > threshold=1.7440648078918457 |
| node_161: feature_name=ENST00000422622 | feature_id[52].value <= threshold=76.81717681884766 |
| node_162: feature_name=ENST00000634222 | feature_id[36].value > threshold=1.5202934741973877 |
| node_164: feature_name=ENST00000393590 | feature_id[57].value <= threshold=15.50696325302124 |
| node_165: feature_name=ENST00000438425 | feature_id[40].value <= threshold=38.6440544128418 |
| node_166: feature_name=ENST00000367434 | feature_id[21].value > threshold=178.1705780029297 |
| node_176: feature_name=ENST00000390319 | feature_id[59].value > threshold=160.97486114501953 |
| Class: current_smokers | |
| | |
| Rules_76 | passed counts:1 |
| node_0: feature_name=ENST00000284311 | feature_id[0].value > threshold=179.44091796875 |
| node_102: feature_name=ENST00000316418 | feature_id[2].value > threshold=3.560240387916565 |
| node_136: feature_name=ENST00000380672 | feature_id[32].value <= threshold=31.704580307006836 |
| node_137: feature_name=ENST00000308478 | feature_id[1].value > threshold=50.736541748046875 |
| node_155: feature_name=ENST00000390309 | feature_id[41].value > threshold=8.32264518737793 |
| node_159: feature_name=ENST00000580335 | feature_id[63].value > threshold=1.7440648078918457 |
| node_161: feature_name=ENST00000422622 | feature_id[52].value <= threshold=76.81717681884766 |
| node_162: feature_name=ENST00000634222 | feature_id[36].value > threshold=1.5202934741973877 |

| | |
|---|---|
| node_164: feature_name=ENST00000393590 | feature_id[57].value <= threshold=15.50696325302124 |
| node_165: feature_name=ENST00000438425 | feature_id[40].value <= threshold=38.6440544128418 |
| node_166: feature_name=ENST00000367434 | feature_id[21].value > threshold=178.1705780029297 |
| node_176: feature_name=ENST00000390319 | feature_id[59].value <= threshold=160.97486114501953 |
| Class: former_smokers | |
| | |
| Rules_77 | passed counts:1 |
| node_0: feature_name=ENST00000284311 | feature_id[0].value > threshold=179.44091796875 |
| node_102: feature_name=ENST00000316418 | feature_id[2].value > threshold=3.560240387916565 |
| node_136: feature_name=ENST00000380672 | feature_id[32].value <= threshold=31.704580307006836 |
| node_137: feature_name=ENST00000308478 | feature_id[1].value > threshold=50.736541748046875 |
| node_155: feature_name=ENST00000390309 | feature_id[41].value > threshold=8.32264518737793 |
| node_159: feature_name=ENST00000580335 | feature_id[63].value > threshold=1.7440648078918457 |
| node_161: feature_name=ENST00000422622 | feature_id[52].value <= threshold=76.81717681884766 |
| node_162: feature_name=ENST00000634222 | feature_id[36].value > threshold=1.5202934741973877 |
| node_164: feature_name=ENST00000393590 | feature_id[57].value <= threshold=15.50696325302124 |
| node_165: feature_name=ENST00000438425 | feature_id[40].value <= threshold=38.6440544128418 |
| node_166: feature_name=ENST00000367434 | feature_id[21].value <= threshold=178.1705780029297 |
| node_167: feature_name=ENST00000441556 | feature_id[24].value > threshold=20.893842697143555 |
| node_173: feature_name=ENST00000586582 | feature_id[3].value <= threshold=20.026033401489258 |
| Class: former_smokers | |
| | |
| Rules_78 | passed counts:1 |
| node_0: feature_name=ENST00000284311 | feature_id[0].value > threshold=179.44091796875 |
| node_102: feature_name=ENST00000316418 | feature_id[2].value > threshold=3.560240387916565 |
| node_136: feature_name=ENST00000380672 | feature_id[32].value <= threshold=31.704580307006836 |

| | |
|---|---|
| node_137: feature_name=ENST00000308478 | feature_id[1].value > threshold=50.736541748046875 |
| node_155: feature_name=ENST00000390309 | feature_id[41].value > threshold=8.32264518737793 |
| node_159: feature_name=ENST00000580335 | feature_id[63].value > threshold=1.7440648078918457 |
| node_161: feature_name=ENST00000422622 | feature_id[52].value <= threshold=76.81717681884766 |
| node_162: feature_name=ENST00000634222 | feature_id[36].value > threshold=1.5202934741973877 |
| node_164: feature_name=ENST00000393590 | feature_id[57].value <= threshold=15.50696325302124 |
| node_165: feature_name=ENST00000438425 | feature_id[40].value <= threshold=38.6440544128418 |
| node_166: feature_name=ENST00000367434 | feature_id[21].value <= threshold=178.1705780029297 |
| node_167: feature_name=ENST00000441556 | feature_id[24].value <= threshold=20.893842697143555 |
| node_168: feature_name=ENST00000280258 | feature_id[56].value > threshold=250.8539047241211 |
| node_170: feature_name=ENST00000483158 | feature_id[43].value <= threshold=57.99545478820801 |
| Class: former_smokers | |
| | |
| Rules_79 | passed counts:1 |
| node_0: feature_name=ENST00000284311 | feature_id[0].value > threshold=179.44091796875 |
| node_102: feature_name=ENST00000316418 | feature_id[2].value > threshold=3.560240387916565 |
| node_136: feature_name=ENST00000380672 | feature_id[32].value <= threshold=31.704580307006836 |
| node_137: feature_name=ENST00000308478 | feature_id[1].value > threshold=50.736541748046875 |
| node_155: feature_name=ENST00000390309 | feature_id[41].value > threshold=8.32264518737793 |
| node_159: feature_name=ENST00000580335 | feature_id[63].value > threshold=1.7440648078918457 |
| node_161: feature_name=ENST00000422622 | feature_id[52].value <= threshold=76.81717681884766 |
| node_162: feature_name=ENST00000634222 | feature_id[36].value <= threshold=1.5202934741973877 |
| Class: former_smokers | |
| | |
| Rules_80 | passed counts:1 |
| node_0: feature_name=ENST00000284311 | feature_id[0].value > threshold=179.44091796875 |

| | |
|---|---|
| node_102: feature_name=ENST00000316418 | feature_id[2].value > threshold=3.560240387916565 |
| node_136: feature_name=ENST00000380672 | feature_id[32].value <= threshold=31.704580307006836 |
| node_137: feature_name=ENST00000308478 | feature_id[1].value > threshold=50.736541748046875 |
| node_155: feature_name=ENST00000390309 | feature_id[41].value > threshold=8.32264518737793 |
| node_159: feature_name=ENST00000580335 | feature_id[63].value <= threshold=1.7440648078918457 |
| Class: former_smokers | |
| | |
| Rules_81 | passed counts:1 |
| node_0: feature_name=ENST00000284311 | feature_id[0].value > threshold=179.44091796875 |
| node_102: feature_name=ENST00000316418 | feature_id[2].value > threshold=3.560240387916565 |
| node_136: feature_name=ENST00000380672 | feature_id[32].value <= threshold=31.704580307006836 |
| node_137: feature_name=ENST00000308478 | feature_id[1].value <= threshold=50.736541748046875 |
| node_138: feature_name=ENST00000610495 | feature_id[53].value <= threshold=247.7474594116211 |
| node_139: feature_name=ENST00000367929 | feature_id[58].value <= threshold=112.91791152954102 |
| node_140: feature_name=ENST00000390290 | feature_id[46].value <= threshold=23.118605613708496 |
| node_141: feature_name=ENST00000308478 | feature_id[1].value <= threshold=40.582645416259766 |
| node_142: feature_name=ENST00000244174 | feature_id[10].value > threshold=14.476964473724365 |
| Class: former_smokers | |
| | |
| Rules_82 | passed counts:1 |
| node_0: feature_name=ENST00000284311 | feature_id[0].value > threshold=179.44091796875 |
| node_102: feature_name=ENST00000316418 | feature_id[2].value > threshold=3.560240387916565 |
| node_136: feature_name=ENST00000380672 | feature_id[32].value <= threshold=31.704580307006836 |
| node_137: feature_name=ENST00000308478 | feature_id[1].value <= threshold=50.736541748046875 |
| node_138: feature_name=ENST00000610495 | feature_id[53].value <= threshold=247.7474594116211 |
| node_139: feature_name=ENST00000367929 | feature_id[58].value <= threshold=112.91791152954102 |

| | |
|---|---|
| node_140: feature_name=ENST00000390290 | feature_id[46].value <= threshold=23.118605613708496 |
| node_141: feature_name=ENST00000308478 | feature_id[1].value <= threshold=40.582645416259766 |
| node_142: feature_name=ENST00000244174 | feature_id[10].value <= threshold=14.476964473724365 |
| node_143: feature_name=ENST00000390549 | feature_id[28].value <= threshold=15.814601421356201 |
| Class: former_smokers | |
| | |
| Rules_83 | passed counts:1 |
| node_0: feature_name=ENST00000284311 | feature_id[0].value > threshold=179.44091796875 |
| node_102: feature_name=ENST00000316418 | feature_id[2].value <= threshold=3.560240387916565 |
| node_103: feature_name=ENST00000522551 | feature_id[60].value > threshold=57.16539001464844 |
| node_125: feature_name=ENST00000390323 | feature_id[22].value > threshold=204.28018188476562 |
| node_127: feature_name=ENST00000280258 | feature_id[56].value > threshold=121.94679641723633 |
| node_129: feature_name=ENST00000396625 | feature_id[33].value > threshold=28.375680923461914 |
| node_133: feature_name=ENST00000390237 | feature_id[54].value > threshold=5609.633544921875 |
| Class: former_smokers | |
| | |
| Rules_84 | passed counts:1 |
| node_0: feature_name=ENST00000284311 | feature_id[0].value > threshold=179.44091796875 |
| node_102: feature_name=ENST00000316418 | feature_id[2].value <= threshold=3.560240387916565 |
| node_103: feature_name=ENST00000522551 | feature_id[60].value > threshold=57.16539001464844 |
| node_125: feature_name=ENST00000390323 | feature_id[22].value > threshold=204.28018188476562 |
| node_127: feature_name=ENST00000280258 | feature_id[56].value > threshold=121.94679641723633 |
| node_129: feature_name=ENST00000396625 | feature_id[33].value <= threshold=28.375680923461914 |
| node_130: feature_name=ENST00000390252 | feature_id[25].value <= threshold=59.50105094909668 |
| Class: current_smokers | |
| | |

| | |
|---|---|
| Rules_85 | passed counts:1 |
| node_0: feature_name=ENST00000284311 | feature_id[0].value > threshold=179.44091796875 |
| node_102: feature_name=ENST00000316418 | feature_id[2].value <= threshold=3.560240387916565 |
| node_103: feature_name=ENST00000522551 | feature_id[60].value <= threshold=57.16539001464844 |
| node_104: feature_name=ENST00000367467 | feature_id[9].value > threshold=43.146501541137695 |
| node_112: feature_name=ENST00000339223 | feature_id[13].value > threshold=13.673684120178223 |
| node_114: feature_name=ENST00000380672 | feature_id[32].value > threshold=51.63601493835449 |
| node_122: feature_name=ENST00000390319 | feature_id[59].value > threshold=108.81984329223633 |
| Class: current_smokers | |
| | |
| Rules_86 | passed counts:1 |
| node_0: feature_name=ENST00000284311 | feature_id[0].value > threshold=179.44091796875 |
| node_102: feature_name=ENST00000316418 | feature_id[2].value <= threshold=3.560240387916565 |
| node_103: feature_name=ENST00000522551 | feature_id[60].value <= threshold=57.16539001464844 |
| node_104: feature_name=ENST00000367467 | feature_id[9].value <= threshold=43.146501541137695 |
| node_105: feature_name=ENST00000480786 | feature_id[62].value > threshold=3.7895997762680054 |
| node_109: feature_name=ENST00000390252 | feature_id[25].value > threshold=82.72151565551758 |
| Class: former_smokers | |
| | |
| Rules_87 | passed counts:1 |
| node_0: feature_name=ENST00000284311 | feature_id[0].value > threshold=179.44091796875 |
| node_102: feature_name=ENST00000316418 | feature_id[2].value <= threshold=3.560240387916565 |
| node_103: feature_name=ENST00000522551 | feature_id[60].value <= threshold=57.16539001464844 |
| node_104: feature_name=ENST00000367467 | feature_id[9].value <= threshold=43.146501541137695 |
| node_105: feature_name=ENST00000480786 | feature_id[62].value <= threshold=3.7895997762680054 |
| node_106: feature_name=ENST00000390285 | feature_id[42].value <= threshold=1.2267921566963196 |

| | |
|---|---|
| Class: current_smokers | |
| | |
| Rules_88 | passed counts:1 |
| node_0: feature_name=ENST00000284311 | feature_id[0].value <= threshold=179.44091796875 |
| node_1: feature_name=ENST00000390539 | feature_id[5].value > threshold=11.91484260559082 |
| node_79: feature_name=ENST00000522551 | feature_id[60].value > threshold=95.3083724975586 |
| node_95: feature_name=ENST00000487272 | feature_id[8].value > threshold=20.848535537719727 |
| node_99: feature_name=ENST00000280258 | feature_id[56].value > threshold=249.81621551513672 |
| Class: former_smokers | |
| | |
| Rules_89 | passed counts:1 |
| node_0: feature_name=ENST00000284311 | feature_id[0].value <= threshold=179.44091796875 |
| node_1: feature_name=ENST00000390539 | feature_id[5].value > threshold=11.91484260559082 |
| node_79: feature_name=ENST00000522551 | feature_id[60].value > threshold=95.3083724975586 |
| node_95: feature_name=ENST00000487272 | feature_id[8].value <= threshold=20.848535537719727 |
| node_96: feature_name=ENST00000367434 | feature_id[21].value <= threshold=8.783787488937378 |
| Class: current_smokers | |
| | |
| Rules_90 | passed counts:1 |
| node_0: feature_name=ENST00000284311 | feature_id[0].value <= threshold=179.44091796875 |
| node_1: feature_name=ENST00000390539 | feature_id[5].value > threshold=11.91484260559082 |
| node_79: feature_name=ENST00000522551 | feature_id[60].value <= threshold=95.3083724975586 |
| node_80: feature_name=ENST00000610495 | feature_id[53].value > threshold=54.46980857849121 |
| node_86: feature_name=ENST00000451085 | feature_id[7].value > threshold=6.878771543502808 |
| node_88: feature_name=ENST00000622663 | feature_id[51].value > threshold=3.760047197341919 |
| node_92: feature_name=ENST00000284311 | feature_id[0].value <= threshold=81.37762069702148 |

| | |
|---|---|
| Class: former_smokers | |
| | |
| Rules_91 | passed counts:1 |
| node_0: feature_name=ENST00000284311 | feature_id[0].value <= threshold=179.44091796875 |
| node_1: feature_name=ENST00000390539 | feature_id[5].value > threshold=11.91484260559082 |
| node_79: feature_name=ENST00000522551 | feature_id[60].value <= threshold=95.3083724975586 |
| node_80: feature_name=ENST00000610495 | feature_id[53].value <= threshold=54.46980857849121 |
| node_81: feature_name=ENST00000284311 | feature_id[0].value <= threshold=45.77398490905762 |
| node_82: feature_name=ENST00000390308 | feature_id[16].value <= threshold=10.502037525177002 |
| Class: current_smokers | |
| | |
| Rules_92 | passed counts:1 |
| node_0: feature_name=ENST00000284311 | feature_id[0].value <= threshold=179.44091796875 |
| node_1: feature_name=ENST00000390539 | feature_id[5].value <= threshold=11.91484260559082 |
| node_2: feature_name=ENST00000586582 | feature_id[3].value > threshold=17.368224143981934 |
| node_74: feature_name=ENST00000316418 | feature_id[2].value <= threshold=3.9801650047302246 |
| node_75: feature_name=ENST00000308478 | feature_id[1].value <= threshold=9.707509994506836 |
| Class: current_smokers | |
| | |
| Rules_93 | passed counts:1 |
| node_0: feature_name=ENST00000284311 | feature_id[0].value <= threshold=179.44091796875 |
| node_1: feature_name=ENST00000390539 | feature_id[5].value <= threshold=11.91484260559082 |
| node_2: feature_name=ENST00000586582 | feature_id[3].value <= threshold=17.368224143981934 |
| node_3: feature_name=ENST00000280258 | feature_id[56].value > threshold=75.14949798583984 |
| node_39: feature_name=ENST00000438425 | feature_id[40].value > threshold=37.27690362930298 |
| Class: current_smokers | |

| | |
|---|---|
| | |
| Rules_94 | passed counts:1 |
| node_0: feature_name=ENST00000284311 | feature_id[0].value <= threshold=179.44091796875 |
| node_1: feature_name=ENST00000390539 | feature_id[5].value <= threshold=11.91484260559082 |
| node_2: feature_name=ENST00000586582 | feature_id[3].value <= threshold=17.368224143981934 |
| node_3: feature_name=ENST00000280258 | feature_id[56].value > threshold=75.14949798583984 |
| node_39: feature_name=ENST00000438425 | feature_id[40].value <= threshold=37.27690362930298 |
| node_40: feature_name=ENST00000393590 | feature_id[57].value > threshold=16.18181800842285 |
| Class: current_smokers | |
| | |
| Rules_95 | passed counts:1 |
| node_0: feature_name=ENST00000284311 | feature_id[0].value <= threshold=179.44091796875 |
| node_1: feature_name=ENST00000390539 | feature_id[5].value <= threshold=11.91484260559082 |
| node_2: feature_name=ENST00000586582 | feature_id[3].value <= threshold=17.368224143981934 |
| node_3: feature_name=ENST00000280258 | feature_id[56].value > threshold=75.14949798583984 |
| node_39: feature_name=ENST00000438425 | feature_id[40].value <= threshold=37.27690362930298 |
| node_40: feature_name=ENST00000393590 | feature_id[57].value <= threshold=16.18181800842285 |
| node_41: feature_name=ENST00000316418 | feature_id[2].value > threshold=7.755321025848389 |
| node_65: feature_name=ENST00000244174 | feature_id[10].value <= threshold=3.6251673698425293 |
| node_66: feature_name=ENST00000586582 | feature_id[3].value > threshold=12.385812759399414 |
| Class: current_smokers | |
| | |
| Rules_96 | passed counts:1 |
| node_0: feature_name=ENST00000284311 | feature_id[0].value <= threshold=179.44091796875 |
| node_1: feature_name=ENST00000390539 | feature_id[5].value <= threshold=11.91484260559082 |
| node_2: feature_name=ENST00000586582 | feature_id[3].value <= threshold=17.368224143981934 |

| | |
|---|---|
| node_3: feature_name=ENST00000280258 | feature_id[56].value > threshold=75.14949798583984 |
| node_39: feature_name=ENST00000438425 | feature_id[40].value <= threshold=37.27690362930298 |
| node_40: feature_name=ENST00000393590 | feature_id[57].value <= threshold=16.18181800842285 |
| node_41: feature_name=ENST00000316418 | feature_id[2].value <= threshold=7.755321025848389 |
| node_42: feature_name=ENST00000390606 | feature_id[27].value <= threshold=15.668272972106934 |
| node_43: feature_name=ENST00000441556 | feature_id[24].value <= threshold=9.982987880706787 |
| node_44: feature_name=ENST00000610495 | feature_id[53].value > threshold=8.27418327331543 |
| node_48: feature_name=ENST00000441556 | feature_id[24].value > threshold=5.98947286605835 |
| node_54: feature_name=ENST00000464162 | feature_id[45].value > threshold=10.004804372787476 |
| Class: current_smokers | |
| | |
| Rules_97 | passed counts:1 |
| node_0: feature_name=ENST00000284311 | feature_id[0].value <= threshold=179.44091796875 |
| node_1: feature_name=ENST00000390539 | feature_id[5].value <= threshold=11.91484260559082 |
| node_2: feature_name=ENST00000586582 | feature_id[3].value <= threshold=17.368224143981934 |
| node_3: feature_name=ENST00000280258 | feature_id[56].value > threshold=75.14949798583984 |
| node_39: feature_name=ENST00000438425 | feature_id[40].value <= threshold=37.27690362930298 |
| node_40: feature_name=ENST00000393590 | feature_id[57].value <= threshold=16.18181800842285 |
| node_41: feature_name=ENST00000316418 | feature_id[2].value <= threshold=7.755321025848389 |
| node_42: feature_name=ENST00000390606 | feature_id[27].value <= threshold=15.668272972106934 |
| node_43: feature_name=ENST00000441556 | feature_id[24].value <= threshold=9.982987880706787 |
| node_44: feature_name=ENST00000610495 | feature_id[53].value > threshold=8.27418327331543 |
| node_48: feature_name=ENST00000441556 | feature_id[24].value > threshold=5.98947286605835 |
| node_54: feature_name=ENST00000464162 | feature_id[45].value <= threshold=10.004804372787476 |
| node_55: feature_name=ENST00000390319 | feature_id[59].value <= threshold=4.869982719421387 |

| Class: current_smokers | |
|---|---|
| | |
| Rules_98 | passed counts:1 |
| node_0: feature_name=ENST00000284311 | feature_id[0].value <= threshold=179.44091796875 |
| node_1: feature_name=ENST00000390539 | feature_id[5].value <= threshold=11.91484260559082 |
| node_2: feature_name=ENST00000586582 | feature_id[3].value <= threshold=17.368224143981934 |
| node_3: feature_name=ENST00000280258 | feature_id[56].value > threshold=75.14949798583984 |
| node_39: feature_name=ENST00000438425 | feature_id[40].value <= threshold=37.27690362930298 |
| node_40: feature_name=ENST00000393590 | feature_id[57].value <= threshold=16.18181800842285 |
| node_41: feature_name=ENST00000316418 | feature_id[2].value <= threshold=7.755321025848389 |
| node_42: feature_name=ENST00000390606 | feature_id[27].value <= threshold=15.668272972106934 |
| node_43: feature_name=ENST00000441556 | feature_id[24].value <= threshold=9.982987880706787 |
| node_44: feature_name=ENST00000610495 | feature_id[53].value > threshold=8.27418327331543 |
| node_48: feature_name=ENST00000441556 | feature_id[24].value <= threshold=5.98947286605835 |
| node_49: feature_name=ENST00000284311 | feature_id[0].value > threshold=173.78553009033203 |
| node_51: feature_name=ENST00000464162 | feature_id[45].value <= threshold=0.546977698802948 |
| Class: current_smokers | |
| | |
| Rules_99 | passed counts:1 |
| node_0: feature_name=ENST00000284311 | feature_id[0].value <= threshold=179.44091796875 |
| node_1: feature_name=ENST00000390539 | feature_id[5].value <= threshold=11.91484260559082 |
| node_2: feature_name=ENST00000586582 | feature_id[3].value <= threshold=17.368224143981934 |
| node_3: feature_name=ENST00000280258 | feature_id[56].value > threshold=75.14949798583984 |
| node_39: feature_name=ENST00000438425 | feature_id[40].value <= threshold=37.27690362930298 |
| node_40: feature_name=ENST00000393590 | feature_id[57].value <= threshold=16.18181800842285 |
| node_41: feature_name=ENST00000316418 | feature_id[2].value <= threshold=7.755321025848389 |

| | |
|---|---|
| node_42: feature_name=ENST00000390606 | feature_id[27].value <= threshold=15.668272972106934 |
| node_43: feature_name=ENST00000441556 | feature_id[24].value <= threshold=9.982987880706787 |
| node_44: feature_name=ENST00000610495 | feature_id[53].value <= threshold=8.27418327331543 |
| node_45: feature_name=ENST00000610495 | feature_id[53].value > threshold=7.197072744369507 |
| Class: current_smokers | |
| | |
| Rules_100 | passed counts:1 |
| node_0: feature_name=ENST00000284311 | feature_id[0].value <= threshold=179.44091796875 |
| node_1: feature_name=ENST00000390539 | feature_id[5].value <= threshold=11.91484260559082 |
| node_2: feature_name=ENST00000586582 | feature_id[3].value <= threshold=17.368224143981934 |
| node_3: feature_name=ENST00000280258 | feature_id[56].value > threshold=75.14949798583984 |
| node_39: feature_name=ENST00000438425 | feature_id[40].value <= threshold=37.27690362930298 |
| node_40: feature_name=ENST00000393590 | feature_id[57].value <= threshold=16.18181800842285 |
| node_41: feature_name=ENST00000316418 | feature_id[2].value <= threshold=7.755321025848389 |
| node_42: feature_name=ENST00000390606 | feature_id[27].value <= threshold=15.668272972106934 |
| node_43: feature_name=ENST00000441556 | feature_id[24].value <= threshold=9.982987880706787 |
| node_44: feature_name=ENST00000610495 | feature_id[53].value <= threshold=8.27418327331543 |
| node_45: feature_name=ENST00000610495 | feature_id[53].value <= threshold=7.197072744369507 |
| Class: former_smokers | |
| | |
| Rules_101 | passed counts:1 |
| node_0: feature_name=ENST00000284311 | feature_id[0].value <= threshold=179.44091796875 |
| node_1: feature_name=ENST00000390539 | feature_id[5].value <= threshold=11.91484260559082 |
| node_2: feature_name=ENST00000586582 | feature_id[3].value <= threshold=17.368224143981934 |
| node_3: feature_name=ENST00000280258 | feature_id[56].value <= threshold=75.14949798583984 |
| node_4: feature_name=ENST00000284311 | feature_id[0].value > threshold=100.91744613647461 |

| | |
|---|---|
| node_24: feature_name=ENST00000367467 | feature_id[9].value > threshold=35.77326202392578 |
| node_30: feature_name=ENST00000616417 | feature_id[39].value <= threshold=164.16122436523438 |
| node_31: feature_name=ENST00000359228 | feature_id[31].value > threshold=8.815360069274902 |
| node_35: feature_name=ENST00000390323 | feature_id[22].value <= threshold=79.46425247192383 |
| Class: current_smokers | |
| | |
| Rules_102 | passed counts:1 |
| node_0: feature_name=ENST00000284311 | feature_id[0].value <= threshold=179.44091796875 |
| node_1: feature_name=ENST00000390539 | feature_id[5].value <= threshold=11.91484260559082 |
| node_2: feature_name=ENST00000586582 | feature_id[3].value <= threshold=17.368224143981934 |
| node_3: feature_name=ENST00000280258 | feature_id[56].value <= threshold=75.14949798583984 |
| node_4: feature_name=ENST00000284311 | feature_id[0].value > threshold=100.91744613647461 |
| node_24: feature_name=ENST00000367467 | feature_id[9].value <= threshold=35.77326202392578 |
| node_25: feature_name=ENST00000360851 | feature_id[47].value <= threshold=13.60571002960205 |
| node_26: feature_name=ENST00000523272 | feature_id[20].value > threshold=2.3025037050247192 |
| Class: current_smokers | |
| | |
| Rules_103 | passed counts:1 |
| node_0: feature_name=ENST00000284311 | feature_id[0].value <= threshold=179.44091796875 |
| node_1: feature_name=ENST00000390539 | feature_id[5].value <= threshold=11.91484260559082 |
| node_2: feature_name=ENST00000586582 | feature_id[3].value <= threshold=17.368224143981934 |
| node_3: feature_name=ENST00000280258 | feature_id[56].value <= threshold=75.14949798583984 |
| node_4: feature_name=ENST00000284311 | feature_id[0].value <= threshold=100.91744613647461 |
| node_5: feature_name=ENST00000492167 | feature_id[23].value <= threshold=164.934326171875 |
| node_6: feature_name=ENST00000616417 | feature_id[39].value > threshold=0.00022576878836844116 |
| node_8: feature_name=ENST00000276974 | feature_id[49].value > threshold=19.783329486846924 |

| | |
|---|---|
| Class: current_smokers | |
| | |
| Rules_104 | passed counts:1 |
| node_0: feature_name=ENST00000284311 | feature_id[0].value <= threshold=179.44091796875 |
| node_1: feature_name=ENST00000390539 | feature_id[5].value <= threshold=11.91484260559082 |
| node_2: feature_name=ENST00000586582 | feature_id[3].value <= threshold=17.368224143981934 |
| node_3: feature_name=ENST00000280258 | feature_id[56].value <= threshold=75.14949798583984 |
| node_4: feature_name=ENST00000284311 | feature_id[0].value <= threshold=100.91744613647461 |
| node_5: feature_name=ENST00000492167 | feature_id[23].value <= threshold=164.934326171875 |
| node_6: feature_name=ENST00000616417 | feature_id[39].value > threshold=0.00022576878836844116 |
| node_8: feature_name=ENST00000276974 | feature_id[49].value <= threshold=19.783329486846924 |
| node_9: feature_name=ENST00000636279 | feature_id[48].value > threshold=8.218245029449463 |
| Class: current_smokers | |
| | |
| Rules_105 | passed counts:1 |
| node_0: feature_name=ENST00000284311 | feature_id[0].value <= threshold=179.44091796875 |
| node_1: feature_name=ENST00000390539 | feature_id[5].value <= threshold=11.91484260559082 |
| node_2: feature_name=ENST00000586582 | feature_id[3].value <= threshold=17.368224143981934 |
| node_3: feature_name=ENST00000280258 | feature_id[56].value <= threshold=75.14949798583984 |
| node_4: feature_name=ENST00000284311 | feature_id[0].value <= threshold=100.91744613647461 |
| node_5: feature_name=ENST00000492167 | feature_id[23].value <= threshold=164.934326171875 |
| node_6: feature_name=ENST00000616417 | feature_id[39].value > threshold=0.00022576878836844116 |
| node_8: feature_name=ENST00000276974 | feature_id[49].value <= threshold=19.783329486846924 |
| node_9: feature_name=ENST00000636279 | feature_id[48].value <= threshold=8.218245029449463 |
| node_10: feature_name=ENST00000464835 | feature_id[6].value > threshold=17.562889099121094 |
| Class: current_smokers | |

| | |
|---|---|
| Rules_106 | passed counts:1 |
| node_0: feature_name=ENST00000284311 | feature_id[0].value <= threshold=179.44091796875 |
| node_1: feature_name=ENST00000390539 | feature_id[5].value <= threshold=11.91484260559082 |
| node_2: feature_name=ENST00000586582 | feature_id[3].value <= threshold=17.368224143981934 |
| node_3: feature_name=ENST00000280258 | feature_id[56].value <= threshold=75.14949798583984 |
| node_4: feature_name=ENST00000284311 | feature_id[0].value <= threshold=100.91744613647461 |
| node_5: feature_name=ENST00000492167 | feature_id[23].value <= threshold=164.934326171875 |
| node_6: feature_name=ENST00000616417 | feature_id[39].value > threshold=0.00022576878836844116 |
| node_8: feature_name=ENST00000276974 | feature_id[49].value <= threshold=19.783329486846924 |
| node_9: feature_name=ENST00000636279 | feature_id[48].value <= threshold=8.218245029449463 |
| node_10: feature_name=ENST00000464835 | feature_id[6].value <= threshold=17.562889099121094 |
| node_11: feature_name=ENST00000390323 | feature_id[22].value > threshold=50.83455848693848 |
| node_15: feature_name=ENST00000367467 | feature_id[9].value > threshold=75.31536102294922 |
| node_17: feature_name=ENST00000396625 | feature_id[33].value <= threshold=5.065143823623657 |
| Class: current_smokers | |
| | |
| Rules_107 | passed counts:1 |
| node_0: feature_name=ENST00000284311 | feature_id[0].value <= threshold=179.44091796875 |
| node_1: feature_name=ENST00000390539 | feature_id[5].value <= threshold=11.91484260559082 |
| node_2: feature_name=ENST00000586582 | feature_id[3].value <= threshold=17.368224143981934 |
| node_3: feature_name=ENST00000280258 | feature_id[56].value <= threshold=75.14949798583984 |
| node_4: feature_name=ENST00000284311 | feature_id[0].value <= threshold=100.91744613647461 |
| node_5: feature_name=ENST00000492167 | feature_id[23].value <= threshold=164.934326171875 |
| node_6: feature_name=ENST00000616417 | feature_id[39].value <= threshold=0.00022576878836844116 |
| Class: current_smokers | |

(3) Rules on LightGBM feature ranking results

| | |
|---|---|
| Rules_0 | passed counts:303 |
| node_0: feature_name=ENST00000284311 | feature_id[0].value <= threshold=179.44091796875 |
| node_1: feature_name=ENST00000586582 | feature_id[4].value <= threshold=8.390757083892822 |
| node_2: feature_name=ENST00000611977 | feature_id[28].value > threshold=10.182801246643066 |
| node_52: feature_name=ENST00000284311 | feature_id[0].value <= threshold=171.63440704345703 |
| node_53: feature_name=ENST00000529814 | feature_id[22].value <= threshold=208.89944458007812 |
| node_54: feature_name=ENST00000464591 | feature_id[14].value > threshold=95.9560661315918 |
| node_68: feature_name=ENST00000308478 | feature_id[1].value <= threshold=118.57913589477539 |
| node_69: feature_name=ENST00000393203 | feature_id[24].value <= threshold=20.071932792663574 |
| node_70: feature_name=ENST00000341184 | feature_id[20].value <= threshold=55.79721641540527 |
| node_71: feature_name=ENST00000430223 | feature_id[17].value <= threshold=201.39640045166016 |
| Class: former_smokers | |
| | |
| Rules_1 | passed counts:140 |
| node_0: feature_name=ENST00000284311 | feature_id[0].value > threshold=179.44091796875 |
| node_126: feature_name=ENST00000316418 | feature_id[2].value > threshold=3.560240387916565 |
| node_164: feature_name=ENST00000359228 | feature_id[6].value <= threshold=13.078589916229248 |
| node_165: feature_name=ENST00000430223 | feature_id[17].value <= threshold=160.21102142333984 |
| node_166: feature_name=ENST00000426706 | feature_id[26].value <= threshold=108.34806823730469 |
| node_167: feature_name=ENST00000308478 | feature_id[1].value > threshold=50.736541748046875 |
| node_183: feature_name=ENST00000316418 | feature_id[2].value > threshold=3.9712209701538086 |
| node_187: feature_name=ENST00000297785 | feature_id[12].value <= threshold=286.35499572753906 |
| Class: current_smokers | |
| | |
| Rules_2 | passed counts:91 |

| | |
|---|---|
| node_0: feature_name=ENST00000284311 | feature_id[0].value <= threshold=179.44091796875 |
| node_1: feature_name=ENST00000586582 | feature_id[4].value <= threshold=8.390757083892822 |
| node_2: feature_name=ENST00000611977 | feature_id[28].value <= threshold=10.182801246643066 |
| node_3: feature_name=ENST00000284311 | feature_id[0].value <= threshold=119.33577728271484 |
| node_4: feature_name=ENST00000464591 | feature_id[14].value > threshold=92.34555053710938 |
| node_12: feature_name=ENST00000321016 | feature_id[25].value <= threshold=35.220298767089844 |
| node_13: feature_name=ENST00000392054 | feature_id[9].value <= threshold=134.09820556640625 |
| node_14: feature_name=ENST00000430223 | feature_id[17].value > threshold=3.632150173187256 |
| node_18: feature_name=ENST00000359228 | feature_id[6].value <= threshold=18.617298126220703 |
| node_19: feature_name=ENST00000598234 | feature_id[10].value <= threshold=934.7086486816406 |
| node_20: feature_name=ENST00000464835 | feature_id[29].value <= threshold=11.865158081054688 |
| Class: former_smokers | |
| | |
| Rules_3 | passed counts:45 |
| node_0: feature_name=ENST00000284311 | feature_id[0].value > threshold=179.44091796875 |
| node_126: feature_name=ENST00000316418 | feature_id[2].value <= threshold=3.560240387916565 |
| node_127: feature_name=ENST00000617716 | feature_id[19].value > threshold=107.3355827331543 |
| node_147: feature_name=ENST00000284311 | feature_id[0].value <= threshold=491.4850158691406 |
| node_148: feature_name=ENST00000464591 | feature_id[14].value > threshold=175.9096450805664 |
| node_154: feature_name=ENST00000586582 | feature_id[4].value <= threshold=13.973906993865967 |
| node_155: feature_name=ENST00000284311 | feature_id[0].value > threshold=187.64857482910156 |
| Class: former_smokers | |
| | |
| Rules_4 | passed counts:43 |
| node_0: feature_name=ENST00000284311 | feature_id[0].value <= threshold=179.44091796875 |
| node_1: feature_name=ENST00000586582 | feature_id[4].value > threshold=8.390757083892822 |

| | |
|---|---|
| node_103: feature_name=ENST00000464591 | feature_id[14].value > threshold=124.4748764038086 |
| node_115: feature_name=ENST00000367467 | feature_id[3].value <= threshold=118.28409957885742 |
| node_116: feature_name=ENST00000633685 | feature_id[7].value > threshold=18.791728973388672 |
| node_120: feature_name=ENST00000308478 | feature_id[1].value <= threshold=90.40320205688477 |
| Class: former_smokers | |
| | |
| Rules_5 | passed counts:40 |
| node_0: feature_name=ENST00000284311 | feature_id[0].value > threshold=179.44091796875 |
| node_126: feature_name=ENST00000316418 | feature_id[2].value > threshold=3.560240387916565 |
| node_164: feature_name=ENST00000359228 | feature_id[6].value <= threshold=13.078589916229248 |
| node_165: feature_name=ENST00000430223 | feature_id[17].value <= threshold=160.21102142333984 |
| node_166: feature_name=ENST00000426706 | feature_id[26].value <= threshold=108.34806823730469 |
| node_167: feature_name=ENST00000308478 | feature_id[1].value <= threshold=50.736541748046875 |
| node_168: feature_name=ENST00000341184 | feature_id[20].value > threshold=9.95589017868042 |
| node_170: feature_name=ENST00000367467 | feature_id[3].value > threshold=25.33358860015869 |
| node_174: feature_name=ENST00000586582 | feature_id[4].value <= threshold=40.279008865356445 |
| node_175: feature_name=ENST00000308478 | feature_id[1].value <= threshold=49.975419998168945 |
| node_176: feature_name=ENST00000648322 | feature_id[21].value <= threshold=3363.9830322265625 |
| Class: current_smokers | |
| | |
| Rules_6 | passed counts:38 |
| node_0: feature_name=ENST00000284311 | feature_id[0].value <= threshold=179.44091796875 |
| node_1: feature_name=ENST00000586582 | feature_id[4].value <= threshold=8.390757083892822 |
| node_2: feature_name=ENST00000611977 | feature_id[28].value > threshold=10.182801246643066 |
| node_52: feature_name=ENST00000284311 | feature_id[0].value <= threshold=171.63440704345703 |
| node_53: feature_name=ENST00000529814 | feature_id[22].value <= threshold=208.89944458007812 |

| | |
|---|---|
| node_54: feature_name=ENST00000464591 | feature_id[14].value <= threshold=95.9560661315918 |
| node_55: feature_name=ENST00000316418 | feature_id[2].value <= threshold=8.205271005630493 |
| node_56: feature_name=ENST00000509152 | feature_id[15].value > threshold=3.3449586629867554 |
| node_58: feature_name=ENST00000339223 | feature_id[5].value <= threshold=29.682518005371094 |
| node_59: feature_name=ENST00000441556 | feature_id[16].value <= threshold=5.501528263092041 |
| Class: former_smokers | |
| | |
| Rules_7 | passed counts:36 |
| node_0: feature_name=ENST00000284311 | feature_id[0].value > threshold=179.44091796875 |
| node_126: feature_name=ENST00000316418 | feature_id[2].value > threshold=3.560240387916565 |
| node_164: feature_name=ENST00000359228 | feature_id[6].value <= threshold=13.078589916229248 |
| node_165: feature_name=ENST00000430223 | feature_id[17].value > threshold=160.21102142333984 |
| node_201: feature_name=ENST00000284311 | feature_id[0].value > threshold=375.6001434326172 |
| node_215: feature_name=ENST00000620457 | feature_id[11].value <= threshold=250.35345458984375 |
| node_216: feature_name=ENST00000400072 | feature_id[18].value > threshold=0.45605596899986267 |
| Class: current_smokers | |
| | |
| Rules_8 | passed counts:34 |
| node_0: feature_name=ENST00000284311 | feature_id[0].value > threshold=179.44091796875 |
| node_126: feature_name=ENST00000316418 | feature_id[2].value <= threshold=3.560240387916565 |
| node_127: feature_name=ENST00000617716 | feature_id[19].value <= threshold=107.3355827331543 |
| node_128: feature_name=ENST00000423064 | feature_id[31].value <= threshold=26.33747100830078 |
| node_129: feature_name=ENST00000509152 | feature_id[15].value <= threshold=18.67320442199707 |
| node_130: feature_name=ENST00000308478 | feature_id[1].value > threshold=36.34898567199707 |
| node_136: feature_name=ENST00000633685 | feature_id[7].value <= threshold=75.36634826660156 |
| node_137: feature_name=ENST00000392054 | feature_id[9].value <= threshold=96.49601745605469 |

| | |
|---|---|
| Class: current_smokers | |
| | |
| Rules_9 | passed counts:33 |
| node_0: feature_name=ENST00000284311 | feature_id[0].value <= threshold=179.44091796875 |
| node_1: feature_name=ENST00000586582 | feature_id[4].value <= threshold=8.390757083892822 |
| node_2: feature_name=ENST00000611977 | feature_id[28].value > threshold=10.182801246643066 |
| node_52: feature_name=ENST00000284311 | feature_id[0].value <= threshold=171.63440704345703 |
| node_53: feature_name=ENST00000529814 | feature_id[22].value <= threshold=208.89944458007812 |
| node_54: feature_name=ENST00000464591 | feature_id[14].value > threshold=95.9560661315918 |
| node_68: feature_name=ENST00000308478 | feature_id[1].value <= threshold=118.57913589477539 |
| node_69: feature_name=ENST00000393203 | feature_id[24].value > threshold=20.071932792663574 |
| node_79: feature_name=ENST00000633685 | feature_id[7].value > threshold=3.006709098815918 |
| node_81: feature_name=ENST00000509152 | feature_id[15].value > threshold=4.73750114440918 |
| node_83: feature_name=ENST00000430223 | feature_id[17].value > threshold=72.67713928222656 |
| Class: former_smokers | |
| | |
| Rules_10 | passed counts:26 |
| node_0: feature_name=ENST00000284311 | feature_id[0].value > threshold=179.44091796875 |
| node_126: feature_name=ENST00000316418 | feature_id[2].value > threshold=3.560240387916565 |
| node_164: feature_name=ENST00000359228 | feature_id[6].value > threshold=13.078589916229248 |
| node_222: feature_name=ENST00000308478 | feature_id[1].value <= threshold=110.56229400634766 |
| node_223: feature_name=ENST00000611977 | feature_id[28].value > threshold=10.789980411529541 |
| node_227: feature_name=ENST00000284311 | feature_id[0].value > threshold=193.57646942138672 |
| node_229: feature_name=ENST00000321016 | feature_id[25].value <= threshold=25.449864387512207 |
| Class: former_smokers | |
| | |

| | |
|---|---|
| Rules_11 | passed counts:23 |
| node_0: feature_name=ENST00000284311 | feature_id[0].value <= threshold=179.44091796875 |
| node_1: feature_name=ENST00000586582 | feature_id[4].value > threshold=8.390757083892822 |
| node_103: feature_name=ENST00000464591 | feature_id[14].value <= threshold=124.4748764038086 |
| node_104: feature_name=ENST00000316418 | feature_id[2].value > threshold=1.4633999466896057 |
| node_110: feature_name=ENST00000284311 | feature_id[0].value > threshold=36.39381790161133 |
| node_112: feature_name=ENST00000611977 | feature_id[28].value <= threshold=26.018301963806152 |
| Class: current_smokers | |
| | |
| Rules_12 | passed counts:20 |
| node_0: feature_name=ENST00000284311 | feature_id[0].value <= threshold=179.44091796875 |
| node_1: feature_name=ENST00000586582 | feature_id[4].value <= threshold=8.390757083892822 |
| node_2: feature_name=ENST00000611977 | feature_id[28].value <= threshold=10.182801246643066 |
| node_3: feature_name=ENST00000284311 | feature_id[0].value > threshold=119.33577728271484 |
| node_33: feature_name=ENST00000321016 | feature_id[25].value <= threshold=10.15928602218628 |
| node_34: feature_name=ENST00000622663 | feature_id[8].value <= threshold=10.966196537017822 |
| node_35: feature_name=ENST00000423064 | feature_id[31].value > threshold=9.336776733398438 |
| node_39: feature_name=ENST00000422622 | feature_id[27].value <= threshold=32.34660339355469 |
| node_40: feature_name=ENST00000367467 | feature_id[3].value > threshold=13.330858707427979 |
| Class: former_smokers | |
| | |
| Rules_13 | passed counts:18 |
| node_0: feature_name=ENST00000284311 | feature_id[0].value > threshold=179.44091796875 |
| node_126: feature_name=ENST00000316418 | feature_id[2].value > threshold=3.560240387916565 |
| node_164: feature_name=ENST00000359228 | feature_id[6].value > threshold=13.078589916229248 |
| node_222: feature_name=ENST00000308478 | feature_id[1].value > threshold=110.56229400634766 |

| Class: current_smokers | |
|---|---|
| | |
| Rules_14 | passed counts:18 |
| node_0: feature_name=ENST00000284311 | feature_id[0].value > threshold=179.44091796875 |
| node_126: feature_name=ENST00000316418 | feature_id[2].value > threshold=3.560240387916565 |
| node_164: feature_name=ENST00000359228 | feature_id[6].value <= threshold=13.078589916229248 |
| node_165: feature_name=ENST00000430223 | feature_id[17].value <= threshold=160.21102142333984 |
| node_166: feature_name=ENST00000426706 | feature_id[26].value <= threshold=108.34806823730469 |
| node_167: feature_name=ENST00000308478 | feature_id[1].value > threshold=50.736541748046875 |
| node_183: feature_name=ENST00000316418 | feature_id[2].value > threshold=3.9712209701538086 |
| node_187: feature_name=ENST00000297785 | feature_id[12].value > threshold=286.35499572753906 |
| node_189: feature_name=ENST00000339223 | feature_id[5].value > threshold=22.9443302154541 |
| Class: current_smokers | |
| | |
| Rules_15 | passed counts:16 |
| node_0: feature_name=ENST00000284311 | feature_id[0].value <= threshold=179.44091796875 |
| node_1: feature_name=ENST00000586582 | feature_id[4].value <= threshold=8.390757083892822 |
| node_2: feature_name=ENST00000611977 | feature_id[28].value <= threshold=10.182801246643066 |
| node_3: feature_name=ENST00000284311 | feature_id[0].value <= threshold=119.33577728271484 |
| node_4: feature_name=ENST00000464591 | feature_id[14].value <= threshold=92.34555053710938 |
| node_5: feature_name=ENST00000284311 | feature_id[0].value <= threshold=53.63719367980957 |
| Class: former_smokers | |
| | |
| Rules_16 | passed counts:15 |
| node_0: feature_name=ENST00000284311 | feature_id[0].value > threshold=179.44091796875 |
| node_126: feature_name=ENST00000316418 | feature_id[2].value > threshold=3.560240387916565 |

| | |
|---|---|
| node_164: feature_name=ENST00000359228 | feature_id[6].value <= threshold=13.078589916229248 |
| node_165: feature_name=ENST00000430223 | feature_id[17].value > threshold=160.21102142333984 |
| node_201: feature_name=ENST00000284311 | feature_id[0].value <= threshold=375.6001434326172 |
| node_202: feature_name=ENST00000414455 | feature_id[23].value > threshold=11.5049147605896 |
| node_208: feature_name=ENST00000341184 | feature_id[20].value <= threshold=41.354347229003906 |
| Class: former_smokers | |
| | |
| Rules_17 | passed counts:11 |
| node_0: feature_name=ENST00000284311 | feature_id[0].value <= threshold=179.44091796875 |
| node_1: feature_name=ENST00000586582 | feature_id[4].value <= threshold=8.390757083892822 |
| node_2: feature_name=ENST00000611977 | feature_id[28].value > threshold=10.182801246643066 |
| node_52: feature_name=ENST00000284311 | feature_id[0].value <= threshold=171.63440704345703 |
| node_53: feature_name=ENST00000529814 | feature_id[22].value <= threshold=208.89944458007812 |
| node_54: feature_name=ENST00000464591 | feature_id[14].value > threshold=95.9560661315918 |
| node_68: feature_name=ENST00000308478 | feature_id[1].value <= threshold=118.57913589477539 |
| node_69: feature_name=ENST00000393203 | feature_id[24].value <= threshold=20.071932792663574 |
| node_70: feature_name=ENST00000341184 | feature_id[20].value <= threshold=55.79721641540527 |
| node_71: feature_name=ENST00000430223 | feature_id[17].value > threshold=201.39640045166016 |
| node_73: feature_name=ENST00000619589 | feature_id[30].value > threshold=169.5354232788086 |
| Class: former_smokers | |
| | |
| Rules_18 | passed counts:10 |
| node_0: feature_name=ENST00000284311 | feature_id[0].value <= threshold=179.44091796875 |
| node_1: feature_name=ENST00000586582 | feature_id[4].value <= threshold=8.390757083892822 |
| node_2: feature_name=ENST00000611977 | feature_id[28].value <= threshold=10.182801246643066 |
| node_3: feature_name=ENST00000284311 | feature_id[0].value > threshold=119.33577728271484 |

| | |
|---|---|
| node_33: feature_name=ENST00000321016 | feature_id[25].value > threshold=10.15928602218628 |
| node_49: feature_name=ENST00000464835 | feature_id[29].value > threshold=0.5748887062072754 |
| Class: current_smokers | |
| | |
| Rules_19 | passed counts:9 |
| node_0: feature_name=ENST00000284311 | feature_id[0].value > threshold=179.44091796875 |
| node_126: feature_name=ENST00000316418 | feature_id[2].value > threshold=3.560240387916565 |
| node_164: feature_name=ENST00000359228 | feature_id[6].value <= threshold=13.078589916229248 |
| node_165: feature_name=ENST00000430223 | feature_id[17].value <= threshold=160.21102142333984 |
| node_166: feature_name=ENST00000426706 | feature_id[26].value > threshold=108.34806823730469 |
| node_192: feature_name=ENST00000620457 | feature_id[11].value <= threshold=5.781731128692627 |
| Class: current_smokers | |
| | |
| Rules_20 | passed counts:9 |
| node_0: feature_name=ENST00000284311 | feature_id[0].value <= threshold=179.44091796875 |
| node_1: feature_name=ENST00000586582 | feature_id[4].value <= threshold=8.390757083892822 |
| node_2: feature_name=ENST00000611977 | feature_id[28].value > threshold=10.182801246643066 |
| node_52: feature_name=ENST00000284311 | feature_id[0].value > threshold=171.63440704345703 |
| node_96: feature_name=ENST00000619589 | feature_id[30].value > threshold=149.08203125 |
| node_98: feature_name=ENST00000297785 | feature_id[12].value > threshold=164.83590698242188 |
| Class: former_smokers | |
| | |
| Rules_21 | passed counts:9 |
| node_0: feature_name=ENST00000284311 | feature_id[0].value <= threshold=179.44091796875 |
| node_1: feature_name=ENST00000586582 | feature_id[4].value <= threshold=8.390757083892822 |
| node_2: feature_name=ENST00000611977 | feature_id[28].value > threshold=10.182801246643066 |

| | |
|---|---|
| node_52: feature_name=ENST00000284311 | feature_id[0].value <= threshold=171.63440704345703 |
| node_53: feature_name=ENST00000529814 | feature_id[22].value > threshold=208.89944458007812 |
| node_91: feature_name=ENST00000633685 | feature_id[7].value > threshold=12.04007339477539 |
| Class: former_smokers | |
| | |
| Rules_22 | passed counts:9 |
| node_0: feature_name=ENST00000284311 | feature_id[0].value <= threshold=179.44091796875 |
| node_1: feature_name=ENST00000586582 | feature_id[4].value <= threshold=8.390757083892822 |
| node_2: feature_name=ENST00000611977 | feature_id[28].value <= threshold=10.182801246643066 |
| node_3: feature_name=ENST00000284311 | feature_id[0].value <= threshold=119.33577728271484 |
| node_4: feature_name=ENST00000464591 | feature_id[14].value <= threshold=92.34555053710938 |
| node_5: feature_name=ENST00000284311 | feature_id[0].value > threshold=53.63719367980957 |
| node_7: feature_name=ENST00000622663 | feature_id[8].value > threshold=0.7117926478385925 |
| Class: current_smokers | |
| | |
| Rules_23 | passed counts:8 |
| node_0: feature_name=ENST00000284311 | feature_id[0].value > threshold=179.44091796875 |
| node_126: feature_name=ENST00000316418 | feature_id[2].value > threshold=3.560240387916565 |
| node_164: feature_name=ENST00000359228 | feature_id[6].value <= threshold=13.078589916229248 |
| node_165: feature_name=ENST00000430223 | feature_id[17].value <= threshold=160.21102142333984 |
| node_166: feature_name=ENST00000426706 | feature_id[26].value > threshold=108.34806823730469 |
| node_192: feature_name=ENST00000620457 | feature_id[11].value > threshold=5.781731128692627 |
| node_194: feature_name=ENST00000620457 | feature_id[11].value > threshold=23.991544723510742 |
| node_196: feature_name=ENST00000598234 | feature_id[10].value > threshold=334.1088409423828 |
| Class: current_smokers | |
| | |

| | |
|---|---|
| Rules_24 | passed counts:7 |
| node_0: feature_name=ENST00000284311 | feature_id[0].value > threshold=179.44091796875 |
| node_126: feature_name=ENST00000316418 | feature_id[2].value > threshold=3.560240387916565 |
| node_164: feature_name=ENST00000359228 | feature_id[6].value <= threshold=13.078589916229248 |
| node_165: feature_name=ENST00000430223 | feature_id[17].value <= threshold=160.21102142333984 |
| node_166: feature_name=ENST00000426706 | feature_id[26].value > threshold=108.34806823730469 |
| node_192: feature_name=ENST00000620457 | feature_id[11].value > threshold=5.781731128692627 |
| node_194: feature_name=ENST00000620457 | feature_id[11].value <= threshold=23.991544723510742 |
| Class: former_smokers | |
| | |
| Rules_25 | passed counts:7 |
| node_0: feature_name=ENST00000284311 | feature_id[0].value > threshold=179.44091796875 |
| node_126: feature_name=ENST00000316418 | feature_id[2].value <= threshold=3.560240387916565 |
| node_127: feature_name=ENST00000617716 | feature_id[19].value <= threshold=107.3355827331543 |
| node_128: feature_name=ENST00000423064 | feature_id[31].value <= threshold=26.33747100830078 |
| node_129: feature_name=ENST00000509152 | feature_id[15].value <= threshold=18.67320442199707 |
| node_130: feature_name=ENST00000308478 | feature_id[1].value <= threshold=36.34898567199707 |
| node_131: feature_name=ENST00000598234 | feature_id[10].value <= threshold=310.17881774902344 |
| node_132: feature_name=ENST00000422622 | feature_id[27].value > threshold=4.31829833984375 |
| Class: former_smokers | |
| | |
| Rules_26 | passed counts:7 |
| node_0: feature_name=ENST00000284311 | feature_id[0].value <= threshold=179.44091796875 |
| node_1: feature_name=ENST00000586582 | feature_id[4].value <= threshold=8.390757083892822 |
| node_2: feature_name=ENST00000611977 | feature_id[28].value <= threshold=10.182801246643066 |
| node_3: feature_name=ENST00000284311 | feature_id[0].value <= threshold=119.33577728271484 |

| | |
|---|---|
| node_4: feature_name=ENST00000464591 | feature_id[14].value <= threshold=92.34555053710938 |
| node_5: feature_name=ENST00000284311 | feature_id[0].value > threshold=53.63719367980957 |
| node_7: feature_name=ENST00000622663 | feature_id[8].value <= threshold=0.7117926478385925 |
| node_8: feature_name=ENST00000393203 | feature_id[24].value <= threshold=11.876175880432129 |
| Class: former_smokers | |
| | |
| Rules_27 | passed counts:6 |
| node_0: feature_name=ENST00000284311 | feature_id[0].value > threshold=179.44091796875 |
| node_126: feature_name=ENST00000316418 | feature_id[2].value > threshold=3.560240387916565 |
| node_164: feature_name=ENST00000359228 | feature_id[6].value <= threshold=13.078589916229248 |
| node_165: feature_name=ENST00000430223 | feature_id[17].value > threshold=160.21102142333984 |
| node_201: feature_name=ENST00000284311 | feature_id[0].value <= threshold=375.6001434326172 |
| node_202: feature_name=ENST00000414455 | feature_id[23].value <= threshold=11.5049147605896 |
| node_203: feature_name=ENST00000620457 | feature_id[11].value <= threshold=45.874977111816406 |
| Class: current_smokers | |
| | |
| Rules_28 | passed counts:6 |
| node_0: feature_name=ENST00000284311 | feature_id[0].value > threshold=179.44091796875 |
| node_126: feature_name=ENST00000316418 | feature_id[2].value <= threshold=3.560240387916565 |
| node_127: feature_name=ENST00000617716 | feature_id[19].value > threshold=107.3355827331543 |
| node_147: feature_name=ENST00000284311 | feature_id[0].value <= threshold=491.4850158691406 |
| node_148: feature_name=ENST00000464591 | feature_id[14].value <= threshold=175.9096450805664 |
| node_149: feature_name=ENST00000308478 | feature_id[1].value <= threshold=86.52909469604492 |
| node_150: feature_name=ENST00000509152 | feature_id[15].value <= threshold=26.248973846435547 |
| Class: former_smokers | |
| | |

| | |
|---|---|
| Rules_29 | passed counts:6 |
| node_0: feature_name=ENST00000284311 | feature_id[0].value > threshold=179.44091796875 |
| node_126: feature_name=ENST00000316418 | feature_id[2].value <= threshold=3.560240387916565 |
| node_127: feature_name=ENST00000617716 | feature_id[19].value <= threshold=107.3355827331543 |
| node_128: feature_name=ENST00000423064 | feature_id[31].value > threshold=26.33747100830078 |
| Class: former_smokers | |
| | |
| Rules_30 | passed counts:6 |
| node_0: feature_name=ENST00000284311 | feature_id[0].value > threshold=179.44091796875 |
| node_126: feature_name=ENST00000316418 | feature_id[2].value <= threshold=3.560240387916565 |
| node_127: feature_name=ENST00000617716 | feature_id[19].value <= threshold=107.3355827331543 |
| node_128: feature_name=ENST00000423064 | feature_id[31].value <= threshold=26.33747100830078 |
| node_129: feature_name=ENST00000509152 | feature_id[15].value > threshold=18.67320442199707 |
| node_143: feature_name=ENST00000367467 | feature_id[3].value <= threshold=56.50407028198242 |
| Class: former_smokers | |
| | |
| Rules_31 | passed counts:6 |
| node_0: feature_name=ENST00000284311 | feature_id[0].value > threshold=179.44091796875 |
| node_126: feature_name=ENST00000316418 | feature_id[2].value <= threshold=3.560240387916565 |
| node_127: feature_name=ENST00000617716 | feature_id[19].value <= threshold=107.3355827331543 |
| node_128: feature_name=ENST00000423064 | feature_id[31].value <= threshold=26.33747100830078 |
| node_129: feature_name=ENST00000509152 | feature_id[15].value <= threshold=18.67320442199707 |
| node_130: feature_name=ENST00000308478 | feature_id[1].value <= threshold=36.34898567199707 |
| node_131: feature_name=ENST00000598234 | feature_id[10].value > threshold=310.17881774902344 |
| Class: current_smokers | |
| | |

| | |
|---|---|
| Rules_32 | passed counts:6 |
| node_0: feature_name=ENST00000284311 | feature_id[0].value <= threshold=179.44091796875 |
| node_1: feature_name=ENST00000586582 | feature_id[4].value <= threshold=8.390757083892822 |
| node_2: feature_name=ENST00000611977 | feature_id[28].value <= threshold=10.182801246643066 |
| node_3: feature_name=ENST00000284311 | feature_id[0].value <= threshold=119.33577728271484 |
| node_4: feature_name=ENST00000464591 | feature_id[14].value > threshold=92.34555053710938 |
| node_12: feature_name=ENST00000321016 | feature_id[25].value <= threshold=35.220298767089844 |
| node_13: feature_name=ENST00000392054 | feature_id[9].value <= threshold=134.09820556640625 |
| node_14: feature_name=ENST00000430223 | feature_id[17].value > threshold=3.632150173187256 |
| node_18: feature_name=ENST00000359228 | feature_id[6].value <= threshold=18.617298126220703 |
| node_19: feature_name=ENST00000598234 | feature_id[10].value <= threshold=934.7086486816406 |
| node_20: feature_name=ENST00000464835 | feature_id[29].value > threshold=11.865158081054688 |
| node_22: feature_name=ENST00000464835 | feature_id[29].value > threshold=12.369301795959473 |
| Class: former_smokers | |
| | |
| Rules_33 | passed counts:5 |
| node_0: feature_name=ENST00000284311 | feature_id[0].value > threshold=179.44091796875 |
| node_126: feature_name=ENST00000316418 | feature_id[2].value <= threshold=3.560240387916565 |
| node_127: feature_name=ENST00000617716 | feature_id[19].value > threshold=107.3355827331543 |
| node_147: feature_name=ENST00000284311 | feature_id[0].value > threshold=491.4850158691406 |
| node_161: feature_name=ENST00000367467 | feature_id[3].value > threshold=29.699271202087402 |
| Class: current_smokers | |
| | |
| Rules_34 | passed counts:5 |
| node_0: feature_name=ENST00000284311 | feature_id[0].value <= threshold=179.44091796875 |
| node_1: feature_name=ENST00000586582 | feature_id[4].value > threshold=8.390757083892822 |

| | |
|---|---|
| node_103: feature_name=ENST00000464591 | feature_id[14].value <= threshold=124.4748764038086 |
| node_104: feature_name=ENST00000316418 | feature_id[2].value <= threshold=1.4633999466896057 |
| node_105: feature_name=ENST00000620457 | feature_id[11].value > threshold=11.180674076080322 |
| Class: former_smokers | |
| | |
| Rules_35 | passed counts:5 |
| node_0: feature_name=ENST00000284311 | feature_id[0].value <= threshold=179.44091796875 |
| node_1: feature_name=ENST00000586582 | feature_id[4].value <= threshold=8.390757083892822 |
| node_2: feature_name=ENST00000611977 | feature_id[28].value <= threshold=10.182801246643066 |
| node_3: feature_name=ENST00000284311 | feature_id[0].value > threshold=119.33577728271484 |
| node_33: feature_name=ENST00000321016 | feature_id[25].value <= threshold=10.15928602218628 |
| node_34: feature_name=ENST00000622663 | feature_id[8].value > threshold=10.966196537017822 |
| node_46: feature_name=ENST00000414455 | feature_id[23].value <= threshold=33.80800819396973 |
| Class: current_smokers | |
| | |
| Rules_36 | passed counts:4 |
| node_0: feature_name=ENST00000284311 | feature_id[0].value > threshold=179.44091796875 |
| node_126: feature_name=ENST00000316418 | feature_id[2].value > threshold=3.560240387916565 |
| node_164: feature_name=ENST00000359228 | feature_id[6].value > threshold=13.078589916229248 |
| node_222: feature_name=ENST00000308478 | feature_id[1].value <= threshold=110.56229400634766 |
| node_223: feature_name=ENST00000611977 | feature_id[28].value <= threshold=10.789980411529541 |
| node_224: feature_name=ENST00000400072 | feature_id[18].value > threshold=0.9979191720485687 |
| Class: current_smokers | |
| | |
| Rules_37 | passed counts:4 |
| node_0: feature_name=ENST00000284311 | feature_id[0].value > threshold=179.44091796875 |

| | |
|---|---|
| node_126: feature_name=ENST00000316418 | feature_id[2].value > threshold=3.560240387916565 |
| node_164: feature_name=ENST00000359228 | feature_id[6].value <= threshold=13.078589916229248 |
| node_165: feature_name=ENST00000430223 | feature_id[17].value <= threshold=160.21102142333984 |
| node_166: feature_name=ENST00000426706 | feature_id[26].value > threshold=108.34806823730469 |
| node_192: feature_name=ENST00000620457 | feature_id[11].value > threshold=5.781731128692627 |
| node_194: feature_name=ENST00000620457 | feature_id[11].value > threshold=23.991544723510742 |
| node_196: feature_name=ENST00000598234 | feature_id[10].value <= threshold=334.1088409423828 |
| node_197: feature_name=ENST00000633685 | feature_id[7].value > threshold=24.863945960998535 |
| Class: former_smokers | |
| | |
| Rules_38 | passed counts:4 |
| node_0: feature_name=ENST00000284311 | feature_id[0].value > threshold=179.44091796875 |
| node_126: feature_name=ENST00000316418 | feature_id[2].value <= threshold=3.560240387916565 |
| node_127: feature_name=ENST00000617716 | feature_id[19].value > threshold=107.3355827331543 |
| node_147: feature_name=ENST00000284311 | feature_id[0].value <= threshold=491.4850158691406 |
| node_148: feature_name=ENST00000464591 | feature_id[14].value <= threshold=175.9096450805664 |
| node_149: feature_name=ENST00000308478 | feature_id[1].value > threshold=86.52909469604492 |
| Class: current_smokers | |
| | |
| Rules_39 | passed counts:4 |
| node_0: feature_name=ENST00000284311 | feature_id[0].value <= threshold=179.44091796875 |
| node_1: feature_name=ENST00000586582 | feature_id[4].value > threshold=8.390757083892822 |
| node_103: feature_name=ENST00000464591 | feature_id[14].value > threshold=124.4748764038086 |
| node_115: feature_name=ENST00000367467 | feature_id[3].value > threshold=118.28409957885742 |
| Class: current_smokers | |
| | |

| | |
|---|---|
| Rules_40 | passed counts:4 |
| node_0: feature_name=ENST00000284311 | feature_id[0].value <= threshold=179.44091796875 |
| node_1: feature_name=ENST00000586582 | feature_id[4].value > threshold=8.390757083892822 |
| node_103: feature_name=ENST00000464591 | feature_id[14].value > threshold=124.4748764038086 |
| node_115: feature_name=ENST00000367467 | feature_id[3].value <= threshold=118.28409957885742 |
| node_116: feature_name=ENST00000633685 | feature_id[7].value <= threshold=18.791728973388672 |
| node_117: feature_name=ENST00000392054 | feature_id[9].value > threshold=30.339560508728027 |
| Class: current_smokers | |
| | |
| Rules_41 | passed counts:4 |
| node_0: feature_name=ENST00000284311 | feature_id[0].value <= threshold=179.44091796875 |
| node_1: feature_name=ENST00000586582 | feature_id[4].value <= threshold=8.390757083892822 |
| node_2: feature_name=ENST00000611977 | feature_id[28].value > threshold=10.182801246643066 |
| node_52: feature_name=ENST00000284311 | feature_id[0].value > threshold=171.63440704345703 |
| node_96: feature_name=ENST00000619589 | feature_id[30].value <= threshold=149.08203125 |
| Class: current_smokers | |
| | |
| Rules_42 | passed counts:4 |
| node_0: feature_name=ENST00000284311 | feature_id[0].value <= threshold=179.44091796875 |
| node_1: feature_name=ENST00000586582 | feature_id[4].value <= threshold=8.390757083892822 |
| node_2: feature_name=ENST00000611977 | feature_id[28].value > threshold=10.182801246643066 |
| node_52: feature_name=ENST00000284311 | feature_id[0].value <= threshold=171.63440704345703 |
| node_53: feature_name=ENST00000529814 | feature_id[22].value > threshold=208.89944458007812 |
| node_91: feature_name=ENST00000633685 | feature_id[7].value <= threshold=12.04007339477539 |
| node_92: feature_name=ENST00000611977 | feature_id[28].value > threshold=10.762482643127441 |
| Class: current_smokers | |

| | |
|---|---|
| Rules_43 | passed counts:4 |
| node_0: feature_name=ENST00000284311 | feature_id[0].value <= threshold=179.44091796875 |
| node_1: feature_name=ENST00000586582 | feature_id[4].value <= threshold=8.390757083892822 |
| node_2: feature_name=ENST00000611977 | feature_id[28].value > threshold=10.182801246643066 |
| node_52: feature_name=ENST00000284311 | feature_id[0].value <= threshold=171.63440704345703 |
| node_53: feature_name=ENST00000529814 | feature_id[22].value <= threshold=208.89944458007812 |
| node_54: feature_name=ENST00000464591 | feature_id[14].value <= threshold=95.9560661315918 |
| node_55: feature_name=ENST00000316418 | feature_id[2].value > threshold=8.205271005630493 |
| node_65: feature_name=ENST00000367467 | feature_id[3].value > threshold=31.65692138671875 |
| Class: current_smokers | |
| | |
| Rules_44 | passed counts:3 |
| node_0: feature_name=ENST00000284311 | feature_id[0].value > threshold=179.44091796875 |
| node_126: feature_name=ENST00000316418 | feature_id[2].value > threshold=3.560240387916565 |
| node_164: feature_name=ENST00000359228 | feature_id[6].value <= threshold=13.078589916229248 |
| node_165: feature_name=ENST00000430223 | feature_id[17].value > threshold=160.21102142333984 |
| node_201: feature_name=ENST00000284311 | feature_id[0].value > threshold=375.6001434326172 |
| node_215: feature_name=ENST00000620457 | feature_id[11].value <= threshold=250.35345458984375 |
| node_216: feature_name=ENST00000400072 | feature_id[18].value <= threshold=0.45605596899986267 |
| node_217: feature_name=ENST00000308478 | feature_id[1].value <= threshold=101.69314575195312 |
| Class: current_smokers | |
| | |
| Rules_45 | passed counts:3 |
| node_0: feature_name=ENST00000284311 | feature_id[0].value > threshold=179.44091796875 |
| node_126: feature_name=ENST00000316418 | feature_id[2].value > threshold=3.560240387916565 |

| | |
|---|---|
| node_164: feature_name=ENST00000359228 | feature_id[6].value <= threshold=13.078589916229248 |
| node_165: feature_name=ENST00000430223 | feature_id[17].value > threshold=160.21102142333984 |
| node_201: feature_name=ENST00000284311 | feature_id[0].value <= threshold=375.6001434326172 |
| node_202: feature_name=ENST00000414455 | feature_id[23].value > threshold=11.5049147605896 |
| node_208: feature_name=ENST00000341184 | feature_id[20].value > threshold=41.354347229003906 |
| node_210: feature_name=ENST00000316418 | feature_id[2].value > threshold=14.636570453643799 |
| Class: current_smokers | |
| | |
| Rules_46 | passed counts:3 |
| node_0: feature_name=ENST00000284311 | feature_id[0].value > threshold=179.44091796875 |
| node_126: feature_name=ENST00000316418 | feature_id[2].value > threshold=3.560240387916565 |
| node_164: feature_name=ENST00000359228 | feature_id[6].value <= threshold=13.078589916229248 |
| node_165: feature_name=ENST00000430223 | feature_id[17].value > threshold=160.21102142333984 |
| node_201: feature_name=ENST00000284311 | feature_id[0].value <= threshold=375.6001434326172 |
| node_202: feature_name=ENST00000414455 | feature_id[23].value > threshold=11.5049147605896 |
| node_208: feature_name=ENST00000341184 | feature_id[20].value > threshold=41.354347229003906 |
| node_210: feature_name=ENST00000316418 | feature_id[2].value <= threshold=14.636570453643799 |
| node_211: feature_name=ENST00000464591 | feature_id[14].value > threshold=182.17203521728516 |
| Class: former_smokers | |
| | |
| Rules_47 | passed counts:3 |
| node_0: feature_name=ENST00000284311 | feature_id[0].value > threshold=179.44091796875 |
| node_126: feature_name=ENST00000316418 | feature_id[2].value > threshold=3.560240387916565 |
| node_164: feature_name=ENST00000359228 | feature_id[6].value <= threshold=13.078589916229248 |
| node_165: feature_name=ENST00000430223 | feature_id[17].value > threshold=160.21102142333984 |
| node_201: feature_name=ENST00000284311 | feature_id[0].value <= threshold=375.6001434326172 |

| | |
|---|---|
| node_202: feature_name=ENST00000414455 | feature_id[23].value <= threshold=11.5049147605896 |
| node_203: feature_name=ENST00000620457 | feature_id[11].value > threshold=45.874977111816406 |
| node_205: feature_name=ENST00000393590 | feature_id[13].value > threshold=0.6489666104316711 |
| Class: former_smokers | |
| | |
| Rules_48 | passed counts:3 |
| node_0: feature_name=ENST00000284311 | feature_id[0].value > threshold=179.44091796875 |
| node_126: feature_name=ENST00000316418 | feature_id[2].value > threshold=3.560240387916565 |
| node_164: feature_name=ENST00000359228 | feature_id[6].value <= threshold=13.078589916229248 |
| node_165: feature_name=ENST00000430223 | feature_id[17].value <= threshold=160.21102142333984 |
| node_166: feature_name=ENST00000426706 | feature_id[26].value <= threshold=108.34806823730469 |
| node_167: feature_name=ENST00000308478 | feature_id[1].value <= threshold=50.736541748046875 |
| node_168: feature_name=ENST00000341184 | feature_id[20].value > threshold=9.95589017868042 |
| node_170: feature_name=ENST00000367467 | feature_id[3].value <= threshold=25.33358860015869 |
| node_171: feature_name=ENST00000586582 | feature_id[4].value > threshold=3.621730923652649 |
| Class: current_smokers | |
| | |
| Rules_49 | passed counts:3 |
| node_0: feature_name=ENST00000284311 | feature_id[0].value > threshold=179.44091796875 |
| node_126: feature_name=ENST00000316418 | feature_id[2].value > threshold=3.560240387916565 |
| node_164: feature_name=ENST00000359228 | feature_id[6].value <= threshold=13.078589916229248 |
| node_165: feature_name=ENST00000430223 | feature_id[17].value <= threshold=160.21102142333984 |
| node_166: feature_name=ENST00000426706 | feature_id[26].value <= threshold=108.34806823730469 |
| node_167: feature_name=ENST00000308478 | feature_id[1].value <= threshold=50.736541748046875 |
| node_168: feature_name=ENST00000341184 | feature_id[20].value > threshold=9.95589017868042 |
| node_170: feature_name=ENST00000367467 | feature_id[3].value <= threshold=25.33358860015869 |

| | |
|---|---|
| node_171: feature_name=ENST00000586582 | feature_id[4].value <= threshold=3.621730923652649 |
| Class: former_smokers | |
| | |
| Rules_50 | passed counts:3 |
| node_0: feature_name=ENST00000284311 | feature_id[0].value > threshold=179.44091796875 |
| node_126: feature_name=ENST00000316418 | feature_id[2].value > threshold=3.560240387916565 |
| node_164: feature_name=ENST00000359228 | feature_id[6].value <= threshold=13.078589916229248 |
| node_165: feature_name=ENST00000430223 | feature_id[17].value <= threshold=160.21102142333984 |
| node_166: feature_name=ENST00000426706 | feature_id[26].value <= threshold=108.34806823730469 |
| node_167: feature_name=ENST00000308478 | feature_id[1].value <= threshold=50.736541748046875 |
| node_168: feature_name=ENST00000341184 | feature_id[20].value <= threshold=9.95589017868042 |
| Class: former_smokers | |
| | |
| Rules_51 | passed counts:3 |
| node_0: feature_name=ENST00000284311 | feature_id[0].value > threshold=179.44091796875 |
| node_126: feature_name=ENST00000316418 | feature_id[2].value <= threshold=3.560240387916565 |
| node_127: feature_name=ENST00000617716 | feature_id[19].value <= threshold=107.3355827331543 |
| node_128: feature_name=ENST00000423064 | feature_id[31].value <= threshold=26.33747100830078 |
| node_129: feature_name=ENST00000509152 | feature_id[15].value > threshold=18.67320442199707 |
| node_143: feature_name=ENST00000367467 | feature_id[3].value > threshold=56.50407028198242 |
| Class: current_smokers | |
| | |
| Rules_52 | passed counts:3 |
| node_0: feature_name=ENST00000284311 | feature_id[0].value <= threshold=179.44091796875 |
| node_1: feature_name=ENST00000586582 | feature_id[4].value > threshold=8.390757083892822 |
| node_103: feature_name=ENST00000464591 | feature_id[14].value > threshold=124.4748764038086 |

| | |
|---|---|
| node_115: feature_name=ENST00000367467 | feature_id[3].value <= threshold=118.28409957885742 |
| node_116: feature_name=ENST00000633685 | feature_id[7].value > threshold=18.791728973388672 |
| node_120: feature_name=ENST00000308478 | feature_id[1].value > threshold=90.40320205688477 |
| node_122: feature_name=ENST00000297785 | feature_id[12].value > threshold=284.8216323852539 |
| Class: former_smokers | |
| | |
| Rules_53 | passed counts:3 |
| node_0: feature_name=ENST00000284311 | feature_id[0].value <= threshold=179.44091796875 |
| node_1: feature_name=ENST00000586582 | feature_id[4].value > threshold=8.390757083892822 |
| node_103: feature_name=ENST00000464591 | feature_id[14].value > threshold=124.4748764038086 |
| node_115: feature_name=ENST00000367467 | feature_id[3].value <= threshold=118.28409957885742 |
| node_116: feature_name=ENST00000633685 | feature_id[7].value <= threshold=18.791728973388672 |
| node_117: feature_name=ENST00000392054 | feature_id[9].value <= threshold=30.339560508728027 |
| Class: former_smokers | |
| | |
| Rules_54 | passed counts:3 |
| node_0: feature_name=ENST00000284311 | feature_id[0].value <= threshold=179.44091796875 |
| node_1: feature_name=ENST00000586582 | feature_id[4].value > threshold=8.390757083892822 |
| node_103: feature_name=ENST00000464591 | feature_id[14].value <= threshold=124.4748764038086 |
| node_104: feature_name=ENST00000316418 | feature_id[2].value <= threshold=1.4633999466896057 |
| node_105: feature_name=ENST00000620457 | feature_id[11].value <= threshold=11.180674076080322 |
| node_106: feature_name=ENST00000400072 | feature_id[18].value > threshold=0.9992019534111023 |
| Class: current_smokers | |
| | |
| Rules_55 | passed counts:3 |
| node_0: feature_name=ENST00000284311 | feature_id[0].value <= threshold=179.44091796875 |

| | |
|---|---|
| node_1: feature_name=ENST00000586582 | feature_id[4].value <= threshold=8.390757083892822 |
| node_2: feature_name=ENST00000611977 | feature_id[28].value <= threshold=10.182801246643066 |
| node_3: feature_name=ENST00000284311 | feature_id[0].value > threshold=119.33577728271484 |
| node_33: feature_name=ENST00000321016 | feature_id[25].value <= threshold=10.15928602218628 |
| node_34: feature_name=ENST00000622663 | feature_id[8].value <= threshold=10.966196537017822 |
| node_35: feature_name=ENST00000423064 | feature_id[31].value <= threshold=9.336776733398438 |
| node_36: feature_name=ENST00000316418 | feature_id[2].value > threshold=4.905639350414276 |
| Class: current_smokers | |
| | |
| Rules_56 | passed counts:2 |
| node_0: feature_name=ENST00000284311 | feature_id[0].value > threshold=179.44091796875 |
| node_126: feature_name=ENST00000316418 | feature_id[2].value > threshold=3.560240387916565 |
| node_164: feature_name=ENST00000359228 | feature_id[6].value > threshold=13.078589916229248 |
| node_222: feature_name=ENST00000308478 | feature_id[1].value <= threshold=110.56229400634766 |
| node_223: feature_name=ENST00000611977 | feature_id[28].value <= threshold=10.789980411529541 |
| node_224: feature_name=ENST00000400072 | feature_id[18].value <= threshold=0.9979191720485687 |
| Class: former_smokers | |
| | |
| Rules_57 | passed counts:2 |
| node_0: feature_name=ENST00000284311 | feature_id[0].value > threshold=179.44091796875 |
| node_126: feature_name=ENST00000316418 | feature_id[2].value > threshold=3.560240387916565 |
| node_164: feature_name=ENST00000359228 | feature_id[6].value <= threshold=13.078589916229248 |
| node_165: feature_name=ENST00000430223 | feature_id[17].value > threshold=160.21102142333984 |
| node_201: feature_name=ENST00000284311 | feature_id[0].value > threshold=375.6001434326172 |
| node_215: feature_name=ENST00000620457 | feature_id[11].value <= threshold=250.35345458984375 |
| node_216: feature_name=ENST00000400072 | feature_id[18].value <= threshold=0.45605596899986267 |

| | |
|---|---|
| node_217: feature_name=ENST00000308478 | feature_id[1].value > threshold=101.69314575195312 |
| Class: former_smokers | |
| | |
| Rules_58 | passed counts:2 |
| node_0: feature_name=ENST00000284311 | feature_id[0].value > threshold=179.44091796875 |
| node_126: feature_name=ENST00000316418 | feature_id[2].value > threshold=3.560240387916565 |
| node_164: feature_name=ENST00000359228 | feature_id[6].value <= threshold=13.078589916229248 |
| node_165: feature_name=ENST00000430223 | feature_id[17].value <= threshold=160.21102142333984 |
| node_166: feature_name=ENST00000426706 | feature_id[26].value > threshold=108.34806823730469 |
| node_192: feature_name=ENST00000620457 | feature_id[11].value > threshold=5.781731128692627 |
| node_194: feature_name=ENST00000620457 | feature_id[11].value > threshold=23.991544723510742 |
| node_196: feature_name=ENST00000598234 | feature_id[10].value <= threshold=334.1088409423828 |
| node_197: feature_name=ENST00000633685 | feature_id[7].value <= threshold=24.863945960998535 |
| Class: current_smokers | |
| | |
| Rules_59 | passed counts:2 |
| node_0: feature_name=ENST00000284311 | feature_id[0].value > threshold=179.44091796875 |
| node_126: feature_name=ENST00000316418 | feature_id[2].value > threshold=3.560240387916565 |
| node_164: feature_name=ENST00000359228 | feature_id[6].value <= threshold=13.078589916229248 |
| node_165: feature_name=ENST00000430223 | feature_id[17].value <= threshold=160.21102142333984 |
| node_166: feature_name=ENST00000426706 | feature_id[26].value <= threshold=108.34806823730469 |
| node_167: feature_name=ENST00000308478 | feature_id[1].value > threshold=50.736541748046875 |
| node_183: feature_name=ENST00000316418 | feature_id[2].value > threshold=3.9712209701538086 |
| node_187: feature_name=ENST00000297785 | feature_id[12].value > threshold=286.35499572753906 |
| node_189: feature_name=ENST00000339223 | feature_id[5].value <= threshold=22.9443302154541 |
| Class: former_smokers | |

| | |
|---|---|
| Rules_60 | passed counts:2 |
| node_0: feature_name=ENST00000284311 | feature_id[0].value > threshold=179.44091796875 |
| node_126: feature_name=ENST00000316418 | feature_id[2].value > threshold=3.560240387916565 |
| node_164: feature_name=ENST00000359228 | feature_id[6].value <= threshold=13.078589916229248 |
| node_165: feature_name=ENST00000430223 | feature_id[17].value <= threshold=160.21102142333984 |
| node_166: feature_name=ENST00000426706 | feature_id[26].value <= threshold=108.34806823730469 |
| node_167: feature_name=ENST00000308478 | feature_id[1].value > threshold=50.736541748046875 |
| node_183: feature_name=ENST00000316418 | feature_id[2].value <= threshold=3.9712209701538086 |
| node_184: feature_name=ENST00000392054 | feature_id[9].value <= threshold=22.55953884124756 |
| Class: current_smokers | |
| | |
| Rules_61 | passed counts:2 |
| node_0: feature_name=ENST00000284311 | feature_id[0].value > threshold=179.44091796875 |
| node_126: feature_name=ENST00000316418 | feature_id[2].value <= threshold=3.560240387916565 |
| node_127: feature_name=ENST00000617716 | feature_id[19].value > threshold=107.3355827331543 |
| node_147: feature_name=ENST00000284311 | feature_id[0].value <= threshold=491.4850158691406 |
| node_148: feature_name=ENST00000464591 | feature_id[14].value <= threshold=175.9096450805664 |
| node_149: feature_name=ENST00000308478 | feature_id[1].value <= threshold=86.52909469604492 |
| node_150: feature_name=ENST00000509152 | feature_id[15].value > threshold=26.248973846435547 |
| Class: current_smokers | |
| | |
| Rules_62 | passed counts:2 |
| node_0: feature_name=ENST00000284311 | feature_id[0].value > threshold=179.44091796875 |
| node_126: feature_name=ENST00000316418 | feature_id[2].value <= threshold=3.560240387916565 |
| node_127: feature_name=ENST00000617716 | feature_id[19].value <= threshold=107.3355827331543 |

| | |
|---|---|
| node_128: feature_name=ENST00000423064 | feature_id[31].value <= threshold=26.33747100830078 |
| node_129: feature_name=ENST00000509152 | feature_id[15].value <= threshold=18.67320442199707 |
| node_130: feature_name=ENST00000308478 | feature_id[1].value <= threshold=36.34898567199707 |
| node_131: feature_name=ENST00000598234 | feature_id[10].value <= threshold=310.17881774902344 |
| node_132: feature_name=ENST00000422622 | feature_id[27].value <= threshold=4.31829833984375 |
| Class: current_smokers | |
| | |
| Rules_63 | passed counts:2 |
| node_0: feature_name=ENST00000284311 | feature_id[0].value <= threshold=179.44091796875 |
| node_1: feature_name=ENST00000586582 | feature_id[4].value > threshold=8.390757083892822 |
| node_103: feature_name=ENST00000464591 | feature_id[14].value > threshold=124.4748764038086 |
| node_115: feature_name=ENST00000367467 | feature_id[3].value <= threshold=118.28409957885742 |
| node_116: feature_name=ENST00000633685 | feature_id[7].value > threshold=18.791728973388672 |
| node_120: feature_name=ENST00000308478 | feature_id[1].value > threshold=90.40320205688477 |
| node_122: feature_name=ENST00000297785 | feature_id[12].value <= threshold=284.8216323852539 |
| Class: current_smokers | |
| | |
| Rules_64 | passed counts:2 |
| node_0: feature_name=ENST00000284311 | feature_id[0].value <= threshold=179.44091796875 |
| node_1: feature_name=ENST00000586582 | feature_id[4].value <= threshold=8.390757083892822 |
| node_2: feature_name=ENST00000611977 | feature_id[28].value > threshold=10.182801246643066 |
| node_52: feature_name=ENST00000284311 | feature_id[0].value > threshold=171.63440704345703 |
| node_96: feature_name=ENST00000619589 | feature_id[30].value > threshold=149.08203125 |
| node_98: feature_name=ENST00000297785 | feature_id[12].value <= threshold=164.83590698242188 |
| node_99: feature_name=ENST00000633685 | feature_id[7].value <= threshold=49.19912910461426 |
| Class: current_smokers | |

| | |
|---|---|
| Rules_65 | passed counts:2 |
| node_0: feature_name=ENST00000284311 | feature_id[0].value <= threshold=179.44091796875 |
| node_1: feature_name=ENST00000586582 | feature_id[4].value <= threshold=8.390757083892822 |
| node_2: feature_name=ENST00000611977 | feature_id[28].value > threshold=10.182801246643066 |
| node_52: feature_name=ENST00000284311 | feature_id[0].value <= threshold=171.63440704345703 |
| node_53: feature_name=ENST00000529814 | feature_id[22].value <= threshold=208.89944458007812 |
| node_54: feature_name=ENST00000464591 | feature_id[14].value > threshold=95.9560661315918 |
| node_68: feature_name=ENST00000308478 | feature_id[1].value > threshold=118.57913589477539 |
| node_88: feature_name=ENST00000393590 | feature_id[13].value > threshold=1.0553319454193115 |
| Class: former_smokers | |
| | |
| Rules_66 | passed counts:2 |
| node_0: feature_name=ENST00000284311 | feature_id[0].value <= threshold=179.44091796875 |
| node_1: feature_name=ENST00000586582 | feature_id[4].value <= threshold=8.390757083892822 |
| node_2: feature_name=ENST00000611977 | feature_id[28].value > threshold=10.182801246643066 |
| node_52: feature_name=ENST00000284311 | feature_id[0].value <= threshold=171.63440704345703 |
| node_53: feature_name=ENST00000529814 | feature_id[22].value <= threshold=208.89944458007812 |
| node_54: feature_name=ENST00000464591 | feature_id[14].value > threshold=95.9560661315918 |
| node_68: feature_name=ENST00000308478 | feature_id[1].value <= threshold=118.57913589477539 |
| node_69: feature_name=ENST00000393203 | feature_id[24].value > threshold=20.071932792663574 |
| node_79: feature_name=ENST00000633685 | feature_id[7].value <= threshold=3.006709098815918 |
| Class: current_smokers | |
| | |
| Rules_67 | passed counts:2 |
| node_0: feature_name=ENST00000284311 | feature_id[0].value <= threshold=179.44091796875 |

| | |
|---|---|
| node_1: feature_name=ENST00000586582 | feature_id[4].value <= threshold=8.390757083892822 |
| node_2: feature_name=ENST00000611977 | feature_id[28].value > threshold=10.182801246643066 |
| node_52: feature_name=ENST00000284311 | feature_id[0].value <= threshold=171.63440704345703 |
| node_53: feature_name=ENST00000529814 | feature_id[22].value <= threshold=208.89944458007812 |
| node_54: feature_name=ENST00000464591 | feature_id[14].value > threshold=95.9560661315918 |
| node_68: feature_name=ENST00000308478 | feature_id[1].value <= threshold=118.57913589477539 |
| node_69: feature_name=ENST00000393203 | feature_id[24].value <= threshold=20.071932792663574 |
| node_70: feature_name=ENST00000341184 | feature_id[20].value > threshold=55.79721641540527 |
| node_76: feature_name=ENST00000622663 | feature_id[8].value > threshold=1.9858230352401733 |
| Class: former_smokers | |
| | |
| Rules_68 | passed counts:2 |
| node_0: feature_name=ENST00000284311 | feature_id[0].value <= threshold=179.44091796875 |
| node_1: feature_name=ENST00000586582 | feature_id[4].value <= threshold=8.390757083892822 |
| node_2: feature_name=ENST00000611977 | feature_id[28].value <= threshold=10.182801246643066 |
| node_3: feature_name=ENST00000284311 | feature_id[0].value > threshold=119.33577728271484 |
| node_33: feature_name=ENST00000321016 | feature_id[25].value <= threshold=10.15928602218628 |
| node_34: feature_name=ENST00000622663 | feature_id[8].value <= threshold=10.966196537017822 |
| node_35: feature_name=ENST00000423064 | feature_id[31].value > threshold=9.336776733398438 |
| node_39: feature_name=ENST00000422622 | feature_id[27].value <= threshold=32.34660339355469 |
| node_40: feature_name=ENST00000367467 | feature_id[3].value <= threshold=13.330858707427979 |
| node_41: feature_name=ENST00000441556 | feature_id[16].value <= threshold=1.4600719213485718 |
| Class: former_smokers | |
| | |
| Rules_69 | passed counts:2 |
| node_0: feature_name=ENST00000284311 | feature_id[0].value <= threshold=179.44091796875 |

| | |
|---|---|
| node_1: feature_name=ENST00000586582 | feature_id[4].value <= threshold=8.390757083892822 |
| node_2: feature_name=ENST00000611977 | feature_id[28].value <= threshold=10.182801246643066 |
| node_3: feature_name=ENST00000284311 | feature_id[0].value <= threshold=119.33577728271484 |
| node_4: feature_name=ENST00000464591 | feature_id[14].value > threshold=92.34555053710938 |
| node_12: feature_name=ENST00000321016 | feature_id[25].value <= threshold=35.220298767089844 |
| node_13: feature_name=ENST00000392054 | feature_id[9].value <= threshold=134.09820556640625 |
| node_14: feature_name=ENST00000430223 | feature_id[17].value > threshold=3.632150173187256 |
| node_18: feature_name=ENST00000359228 | feature_id[6].value <= threshold=18.617298126220703 |
| node_19: feature_name=ENST00000598234 | feature_id[10].value <= threshold=934.7086486816406 |
| node_20: feature_name=ENST00000464835 | feature_id[29].value > threshold=11.865158081054688 |
| node_22: feature_name=ENST00000464835 | feature_id[29].value <= threshold=12.369301795959473 |
| Class: current_smokers | |
| | |
| Rules_70 | passed counts:2 |
| node_0: feature_name=ENST00000284311 | feature_id[0].value <= threshold=179.44091796875 |
| node_1: feature_name=ENST00000586582 | feature_id[4].value <= threshold=8.390757083892822 |
| node_2: feature_name=ENST00000611977 | feature_id[28].value <= threshold=10.182801246643066 |
| node_3: feature_name=ENST00000284311 | feature_id[0].value <= threshold=119.33577728271484 |
| node_4: feature_name=ENST00000464591 | feature_id[14].value <= threshold=92.34555053710938 |
| node_5: feature_name=ENST00000284311 | feature_id[0].value > threshold=53.63719367980957 |
| node_7: feature_name=ENST00000622663 | feature_id[8].value <= threshold=0.7117926478385925 |
| node_8: feature_name=ENST00000393203 | feature_id[24].value > threshold=11.876175880432129 |
| Class: current_smokers | |
| | |
| Rules_71 | passed counts:1 |
| node_0: feature_name=ENST00000284311 | feature_id[0].value > threshold=179.44091796875 |

| | |
|---|---|
| node_126: feature_name=ENST00000316418 | feature_id[2].value > threshold=3.560240387916565 |
| node_164: feature_name=ENST00000359228 | feature_id[6].value > threshold=13.078589916229248 |
| node_222: feature_name=ENST00000308478 | feature_id[1].value <= threshold=110.56229400634766 |
| node_223: feature_name=ENST00000611977 | feature_id[28].value > threshold=10.789980411529541 |
| node_227: feature_name=ENST0000284311 | feature_id[0].value > threshold=193.57646942138672 |
| node_229: feature_name=ENST00000321016 | feature_id[25].value > threshold=25.449864387512207 |
| node_231: feature_name=ENST00000619589 | feature_id[30].value > threshold=432.6465148925781 |
| Class: former_smokers | |
| | |
| Rules_72 | passed counts:1 |
| node_0: feature_name=ENST00000284311 | feature_id[0].value > threshold=179.44091796875 |
| node_126: feature_name=ENST00000316418 | feature_id[2].value > threshold=3.560240387916565 |
| node_164: feature_name=ENST00000359228 | feature_id[6].value > threshold=13.078589916229248 |
| node_222: feature_name=ENST00000308478 | feature_id[1].value <= threshold=110.56229400634766 |
| node_223: feature_name=ENST00000611977 | feature_id[28].value > threshold=10.789980411529541 |
| node_227: feature_name=ENST0000284311 | feature_id[0].value > threshold=193.57646942138672 |
| node_229: feature_name=ENST00000321016 | feature_id[25].value > threshold=25.449864387512207 |
| node_231: feature_name=ENST00000619589 | feature_id[30].value <= threshold=432.6465148925781 |
| Class: current_smokers | |
| | |
| Rules_73 | passed counts:1 |
| node_0: feature_name=ENST00000284311 | feature_id[0].value > threshold=179.44091796875 |
| node_126: feature_name=ENST00000316418 | feature_id[2].value > threshold=3.560240387916565 |
| node_164: feature_name=ENST00000359228 | feature_id[6].value > threshold=13.078589916229248 |
| node_222: feature_name=ENST00000308478 | feature_id[1].value <= threshold=110.56229400634766 |
| node_223: feature_name=ENST00000611977 | feature_id[28].value > threshold=10.789980411529541 |

| | |
|---|---|
| node_227: feature_name=ENST00000284311 | feature_id[0].value <= threshold=193.57646942138672 |
| Class: current_smokers | |
| | |
| Rules_74 | passed counts:1 |
| node_0: feature_name=ENST00000284311 | feature_id[0].value > threshold=179.44091796875 |
| node_126: feature_name=ENST00000316418 | feature_id[2].value > threshold=3.560240387916565 |
| node_164: feature_name=ENST00000359228 | feature_id[6].value <= threshold=13.078589916229248 |
| node_165: feature_name=ENST00000430223 | feature_id[17].value > threshold=160.21102142333984 |
| node_201: feature_name=ENST00000284311 | feature_id[0].value > threshold=375.6001434326172 |
| node_215: feature_name=ENST00000620457 | feature_id[11].value > threshold=250.35345458984375 |
| Class: former_smokers | |
| | |
| Rules_75 | passed counts:1 |
| node_0: feature_name=ENST00000284311 | feature_id[0].value > threshold=179.44091796875 |
| node_126: feature_name=ENST00000316418 | feature_id[2].value > threshold=3.560240387916565 |
| node_164: feature_name=ENST00000359228 | feature_id[6].value <= threshold=13.078589916229248 |
| node_165: feature_name=ENST00000430223 | feature_id[17].value > threshold=160.21102142333984 |
| node_201: feature_name=ENST00000284311 | feature_id[0].value <= threshold=375.6001434326172 |
| node_202: feature_name=ENST00000414455 | feature_id[23].value > threshold=11.5049147605896 |
| node_208: feature_name=ENST00000341184 | feature_id[20].value > threshold=41.354347229003906 |
| node_210: feature_name=ENST00000316418 | feature_id[2].value <= threshold=14.636570453643799 |
| node_211: feature_name=ENST00000464591 | feature_id[14].value <= threshold=182.17203521728516 |
| Class: current_smokers | |
| | |
| Rules_76 | passed counts:1 |
| node_0: feature_name=ENST00000284311 | feature_id[0].value > threshold=179.44091796875 |

| | |
|---|---|
| node_126: feature_name=ENST00000316418 | feature_id[2].value > threshold=3.560240387916565 |
| node_164: feature_name=ENST00000359228 | feature_id[6].value <= threshold=13.078589916229248 |
| node_165: feature_name=ENST00000430223 | feature_id[17].value > threshold=160.21102142333984 |
| node_201: feature_name=ENST00000284311 | feature_id[0].value <= threshold=375.6001434326172 |
| node_202: feature_name=ENST00000414455 | feature_id[23].value <= threshold=11.5049147605896 |
| node_203: feature_name=ENST00000620457 | feature_id[11].value > threshold=45.874977111816406 |
| node_205: feature_name=ENST00000393590 | feature_id[13].value <= threshold=0.6489666104316711 |
| Class: current_smokers | |
| | |
| Rules_77 | passed counts:1 |
| node_0: feature_name=ENST00000284311 | feature_id[0].value > threshold=179.44091796875 |
| node_126: feature_name=ENST00000316418 | feature_id[2].value > threshold=3.560240387916565 |
| node_164: feature_name=ENST00000359228 | feature_id[6].value <= threshold=13.078589916229248 |
| node_165: feature_name=ENST00000430223 | feature_id[17].value <= threshold=160.21102142333984 |
| node_166: feature_name=ENST00000426706 | feature_id[26].value <= threshold=108.34806823730469 |
| node_167: feature_name=ENST00000308478 | feature_id[1].value > threshold=50.736541748046875 |
| node_183: feature_name=ENST00000316418 | feature_id[2].value <= threshold=3.9712209701538086 |
| node_184: feature_name=ENST00000392054 | feature_id[9].value > threshold=22.55953884124756 |
| Class: former_smokers | |
| | |
| Rules_78 | passed counts:1 |
| node_0: feature_name=ENST00000284311 | feature_id[0].value > threshold=179.44091796875 |
| node_126: feature_name=ENST00000316418 | feature_id[2].value > threshold=3.560240387916565 |
| node_164: feature_name=ENST00000359228 | feature_id[6].value <= threshold=13.078589916229248 |
| node_165: feature_name=ENST00000430223 | feature_id[17].value <= threshold=160.21102142333984 |
| node_166: feature_name=ENST00000426706 | feature_id[26].value <= threshold=108.34806823730469 |

| | |
|---|---|
| node_167: feature_name=ENST00000308478 | feature_id[1].value <= threshold=50.736541748046875 |
| node_168: feature_name=ENST00000341184 | feature_id[20].value > threshold=9.95589017868042 |
| node_170: feature_name=ENST00000367467 | feature_id[3].value > threshold=25.33358860015869 |
| node_174: feature_name=ENST00000586582 | feature_id[4].value > threshold=40.279008865356445 |
| Class: former_smokers | |
| | |
| Rules_79 | passed counts:1 |
| node_0: feature_name=ENST00000284311 | feature_id[0].value > threshold=179.44091796875 |
| node_126: feature_name=ENST00000316418 | feature_id[2].value > threshold=3.560240387916565 |
| node_164: feature_name=ENST00000359228 | feature_id[6].value <= threshold=13.078589916229248 |
| node_165: feature_name=ENST00000430223 | feature_id[17].value <= threshold=160.21102142333984 |
| node_166: feature_name=ENST00000426706 | feature_id[26].value <= threshold=108.34806823730469 |
| node_167: feature_name=ENST00000308478 | feature_id[1].value <= threshold=50.736541748046875 |
| node_168: feature_name=ENST00000341184 | feature_id[20].value > threshold=9.95589017868042 |
| node_170: feature_name=ENST00000367467 | feature_id[3].value > threshold=25.33358860015869 |
| node_174: feature_name=ENST00000586582 | feature_id[4].value <= threshold=40.279008865356445 |
| node_175: feature_name=ENST00000308478 | feature_id[1].value > threshold=49.975419998168945 |
| Class: former_smokers | |
| | |
| Rules_80 | passed counts:1 |
| node_0: feature_name=ENST00000284311 | feature_id[0].value > threshold=179.44091796875 |
| node_126: feature_name=ENST00000316418 | feature_id[2].value > threshold=3.560240387916565 |
| node_164: feature_name=ENST00000359228 | feature_id[6].value <= threshold=13.078589916229248 |
| node_165: feature_name=ENST00000430223 | feature_id[17].value <= threshold=160.21102142333984 |
| node_166: feature_name=ENST00000426706 | feature_id[26].value <= threshold=108.34806823730469 |
| node_167: feature_name=ENST00000308478 | feature_id[1].value <= threshold=50.736541748046875 |

| | |
|---|---|
| node_168: feature_name=ENST00000341184 | feature_id[20].value > threshold=9.95589017868042 |
| node_170: feature_name=ENST00000367467 | feature_id[3].value > threshold=25.33358860015869 |
| node_174: feature_name=ENST00000586582 | feature_id[4].value <= threshold=40.279008865356445 |
| node_175: feature_name=ENST00000308478 | feature_id[1].value <= threshold=49.975419998168945 |
| node_176: feature_name=ENST00000648322 | feature_id[21].value > threshold=3363.9830322265625 |
| node_178: feature_name=ENST00000339223 | feature_id[5].value > threshold=45.06431770324707 |
| Class: current_smokers | |
| | |
| Rules_81 | passed counts:1 |
| node_0: feature_name=ENST00000284311 | feature_id[0].value > threshold=179.44091796875 |
| node_126: feature_name=ENST00000316418 | feature_id[2].value > threshold=3.560240387916565 |
| node_164: feature_name=ENST00000359228 | feature_id[6].value <= threshold=13.078589916229248 |
| node_165: feature_name=ENST00000430223 | feature_id[17].value <= threshold=160.21102142333984 |
| node_166: feature_name=ENST00000426706 | feature_id[26].value <= threshold=108.34806823730469 |
| node_167: feature_name=ENST00000308478 | feature_id[1].value <= threshold=50.736541748046875 |
| node_168: feature_name=ENST00000341184 | feature_id[20].value > threshold=9.95589017868042 |
| node_170: feature_name=ENST00000367467 | feature_id[3].value > threshold=25.33358860015869 |
| node_174: feature_name=ENST00000586582 | feature_id[4].value <= threshold=40.279008865356445 |
| node_175: feature_name=ENST00000308478 | feature_id[1].value <= threshold=49.975419998168945 |
| node_176: feature_name=ENST00000648322 | feature_id[21].value > threshold=3363.9830322265625 |
| node_178: feature_name=ENST00000339223 | feature_id[5].value <= threshold=45.06431770324707 |
| Class: former_smokers | |
| | |
| Rules_82 | passed counts:1 |
| node_0: feature_name=ENST00000284311 | feature_id[0].value > threshold=179.44091796875 |
| node_126: feature_name=ENST00000316418 | feature_id[2].value <= threshold=3.560240387916565 |

| | |
|---|---|
| node_127: feature_name=ENST00000617716 | feature_id[19].value > threshold=107.3355827331543 |
| node_147: feature_name=ENST00000284311 | feature_id[0].value > threshold=491.4850158691406 |
| node_161: feature_name=ENST00000367467 | feature_id[3].value <= threshold=29.699271202087402 |
| Class: former_smokers | |
| | |
| Rules_83 | passed counts:1 |
| node_0: feature_name=ENST00000284311 | feature_id[0].value > threshold=179.44091796875 |
| node_126: feature_name=ENST00000316418 | feature_id[2].value <= threshold=3.560240387916565 |
| node_127: feature_name=ENST00000617716 | feature_id[19].value > threshold=107.3355827331543 |
| node_147: feature_name=ENST00000284311 | feature_id[0].value <= threshold=491.4850158691406 |
| node_148: feature_name=ENST00000464591 | feature_id[14].value > threshold=175.9096450805664 |
| node_154: feature_name=ENST00000586582 | feature_id[4].value > threshold=13.973906993865967 |
| Class: current_smokers | |
| | |
| Rules_84 | passed counts:1 |
| node_0: feature_name=ENST00000284311 | feature_id[0].value > threshold=179.44091796875 |
| node_126: feature_name=ENST00000316418 | feature_id[2].value <= threshold=3.560240387916565 |
| node_127: feature_name=ENST00000617716 | feature_id[19].value > threshold=107.3355827331543 |
| node_147: feature_name=ENST00000284311 | feature_id[0].value <= threshold=491.4850158691406 |
| node_148: feature_name=ENST00000464591 | feature_id[14].value > threshold=175.9096450805664 |
| node_154: feature_name=ENST00000586582 | feature_id[4].value <= threshold=13.973906993865967 |
| node_155: feature_name=ENST00000284311 | feature_id[0].value <= threshold=187.64857482910156 |
| node_156: feature_name=ENST00000464591 | feature_id[14].value > threshold=230.5096664428711 |
| Class: current_smokers | |
| | |
| Rules_85 | passed counts:1 |

| | |
|---|---|
| node_0: feature_name=ENST00000284311 | feature_id[0].value > threshold=179.44091796875 |
| node_126: feature_name=ENST00000316418 | feature_id[2].value <= threshold=3.560240387916565 |
| node_127: feature_name=ENST00000617716 | feature_id[19].value > threshold=107.3355827331543 |
| node_147: feature_name=ENST00000284311 | feature_id[0].value <= threshold=491.4850158691406 |
| node_148: feature_name=ENST00000464591 | feature_id[14].value > threshold=175.9096450805664 |
| node_154: feature_name=ENST00000586582 | feature_id[4].value <= threshold=13.973906993865967 |
| node_155: feature_name=ENST00000284311 | feature_id[0].value <= threshold=187.64857482910156 |
| node_156: feature_name=ENST00000464591 | feature_id[14].value <= threshold=230.5096664428711 |
| Class: former_smokers | |
| | |
| Rules_86 | passed counts:1 |
| node_0: feature_name=ENST00000284311 | feature_id[0].value > threshold=179.44091796875 |
| node_126: feature_name=ENST00000316418 | feature_id[2].value <= threshold=3.560240387916565 |
| node_127: feature_name=ENST00000617716 | feature_id[19].value <= threshold=107.3355827331543 |
| node_128: feature_name=ENST00000423064 | feature_id[31].value <= threshold=26.33747100830078 |
| node_129: feature_name=ENST00000509152 | feature_id[15].value <= threshold=18.67320442199707 |
| node_130: feature_name=ENST00000308478 | feature_id[1].value > threshold=36.34898567199707 |
| node_136: feature_name=ENST00000633685 | feature_id[7].value > threshold=75.36634826660156 |
| Class: former_smokers | |
| | |
| Rules_87 | passed counts:1 |
| node_0: feature_name=ENST00000284311 | feature_id[0].value > threshold=179.44091796875 |
| node_126: feature_name=ENST00000316418 | feature_id[2].value <= threshold=3.560240387916565 |
| node_127: feature_name=ENST00000617716 | feature_id[19].value <= threshold=107.3355827331543 |
| node_128: feature_name=ENST00000423064 | feature_id[31].value <= threshold=26.33747100830078 |
| node_129: feature_name=ENST00000509152 | feature_id[15].value <= threshold=18.67320442199707 |

| | |
|---|---|
| node_130: feature_name=ENST00000308478 | feature_id[1].value > threshold=36.34898567199707 |
| node_136: feature_name=ENST00000633685 | feature_id[7].value <= threshold=75.36634826660156 |
| node_137: feature_name=ENST00000392054 | feature_id[9].value > threshold=96.49601745605469 |
| node_139: feature_name=ENST00000359228 | feature_id[6].value > threshold=1.3470295667648315 |
| Class: former_smokers | |
| | |
| Rules_88 | passed counts:1 |
| node_0: feature_name=ENST00000284311 | feature_id[0].value > threshold=179.44091796875 |
| node_126: feature_name=ENST00000316418 | feature_id[2].value <= threshold=3.560240387916565 |
| node_127: feature_name=ENST00000617716 | feature_id[19].value <= threshold=107.3355827331543 |
| node_128: feature_name=ENST00000423064 | feature_id[31].value <= threshold=26.33747100830078 |
| node_129: feature_name=ENST00000509152 | feature_id[15].value <= threshold=18.67320442199707 |
| node_130: feature_name=ENST00000308478 | feature_id[1].value > threshold=36.34898567199707 |
| node_136: feature_name=ENST00000633685 | feature_id[7].value <= threshold=75.36634826660156 |
| node_137: feature_name=ENST00000392054 | feature_id[9].value > threshold=96.49601745605469 |
| node_139: feature_name=ENST00000359228 | feature_id[6].value <= threshold=1.3470295667648315 |
| Class: current_smokers | |
| | |
| Rules_89 | passed counts:1 |
| node_0: feature_name=ENST00000284311 | feature_id[0].value <= threshold=179.44091796875 |
| node_1: feature_name=ENST00000586582 | feature_id[4].value > threshold=8.390757083892822 |
| node_103: feature_name=ENST00000464591 | feature_id[14].value <= threshold=124.4748764038086 |
| node_104: feature_name=ENST00000316418 | feature_id[2].value > threshold=1.4633999466896057 |
| node_110: feature_name=ENST00000284311 | feature_id[0].value > threshold=36.39381790161133 |
| node_112: feature_name=ENST00000611977 | feature_id[28].value > threshold=26.018301963806152 |
| Class: former_smokers | |

| | |
|---|---|
| Rules_90 | passed counts:1 |
| node_0: feature_name=ENST00000284311 | feature_id[0].value <= threshold=179.44091796875 |
| node_1: feature_name=ENST00000586582 | feature_id[4].value > threshold=8.390757083892822 |
| node_103: feature_name=ENST00000464591 | feature_id[14].value <= threshold=124.4748764038086 |
| node_104: feature_name=ENST00000316418 | feature_id[2].value > threshold=1.4633999466896057 |
| node_110: feature_name=ENST00000284311 | feature_id[0].value <= threshold=36.39381790161133 |
| Class: former_smokers | |
| | |
| Rules_91 | passed counts:1 |
| node_0: feature_name=ENST00000284311 | feature_id[0].value <= threshold=179.44091796875 |
| node_1: feature_name=ENST00000586582 | feature_id[4].value > threshold=8.390757083892822 |
| node_103: feature_name=ENST00000464591 | feature_id[14].value <= threshold=124.4748764038086 |
| node_104: feature_name=ENST00000316418 | feature_id[2].value <= threshold=1.4633999466896057 |
| node_105: feature_name=ENST00000620457 | feature_id[11].value <= threshold=11.180674076080322 |
| node_106: feature_name=ENST00000400072 | feature_id[18].value <= threshold=0.9992019534111023 |
| Class: former_smokers | |
| | |
| Rules_92 | passed counts:1 |
| node_0: feature_name=ENST00000284311 | feature_id[0].value <= threshold=179.44091796875 |
| node_1: feature_name=ENST00000586582 | feature_id[4].value <= threshold=8.390757083892822 |
| node_2: feature_name=ENST00000611977 | feature_id[28].value > threshold=10.182801246643066 |
| node_52: feature_name=ENST00000284311 | feature_id[0].value > threshold=171.63440704345703 |
| node_96: feature_name=ENST00000619589 | feature_id[30].value > threshold=149.08203125 |
| node_98: feature_name=ENST00000297785 | feature_id[12].value <= threshold=164.83590698242188 |
| node_99: feature_name=ENST00000633685 | feature_id[7].value > threshold=49.19912910461426 |

| | |
|---|---|
| Class: former_smokers | |
| | |
| Rules_93 | passed counts:1 |
| node_0: feature_name=ENST00000284311 | feature_id[0].value <= threshold=179.44091796875 |
| node_1: feature_name=ENST00000586582 | feature_id[4].value <= threshold=8.390757083892822 |
| node_2: feature_name=ENST00000611977 | feature_id[28].value > threshold=10.182801246643066 |
| node_52: feature_name=ENST00000284311 | feature_id[0].value <= threshold=171.63440704345703 |
| node_53: feature_name=ENST00000529814 | feature_id[22].value > threshold=208.89944458007812 |
| node_91: feature_name=ENST00000633685 | feature_id[7].value <= threshold=12.04007339477539 |
| node_92: feature_name=ENST00000611977 | feature_id[28].value <= threshold=10.762482643127441 |
| Class: former_smokers | |
| | |
| Rules_94 | passed counts:1 |
| node_0: feature_name=ENST00000284311 | feature_id[0].value <= threshold=179.44091796875 |
| node_1: feature_name=ENST00000586582 | feature_id[4].value <= threshold=8.390757083892822 |
| node_2: feature_name=ENST00000611977 | feature_id[28].value > threshold=10.182801246643066 |
| node_52: feature_name=ENST00000284311 | feature_id[0].value <= threshold=171.63440704345703 |
| node_53: feature_name=ENST00000529814 | feature_id[22].value <= threshold=208.89944458007812 |
| node_54: feature_name=ENST00000464591 | feature_id[14].value > threshold=95.9560661315918 |
| node_68: feature_name=ENST00000308478 | feature_id[1].value > threshold=118.57913589477539 |
| node_88: feature_name=ENST00000393590 | feature_id[13].value <= threshold=1.0553319454193115 |
| Class: current_smokers | |
| | |
| Rules_95 | passed counts:1 |
| node_0: feature_name=ENST00000284311 | feature_id[0].value <= threshold=179.44091796875 |
| node_1: feature_name=ENST00000586582 | feature_id[4].value <= threshold=8.390757083892822 |

| | |
|---|---|
| node_2: feature_name=ENST00000611977 | feature_id[28].value > threshold=10.182801246643066 |
| node_52: feature_name=ENST00000284311 | feature_id[0].value <= threshold=171.63440704345703 |
| node_53: feature_name=ENST00000529814 | feature_id[22].value <= threshold=208.89944458007812 |
| node_54: feature_name=ENST00000464591 | feature_id[14].value > threshold=95.9560661315918 |
| node_68: feature_name=ENST00000308478 | feature_id[1].value <= threshold=118.57913589477539 |
| node_69: feature_name=ENST00000393203 | feature_id[24].value > threshold=20.071932792663574 |
| node_79: feature_name=ENST00000633685 | feature_id[7].value > threshold=3.006709098815918 |
| node_81: feature_name=ENST00000509152 | feature_id[15].value > threshold=4.73750114440918 |
| node_83: feature_name=ENST00000430223 | feature_id[17].value <= threshold=72.67713928222656 |
| node_84: feature_name=ENST00000430223 | feature_id[17].value > threshold=62.10454177856445 |
| Class: current_smokers | |
| | |
| Rules_96 | passed counts:1 |
| node_0: feature_name=ENST00000284311 | feature_id[0].value <= threshold=179.44091796875 |
| node_1: feature_name=ENST00000586582 | feature_id[4].value <= threshold=8.390757083892822 |
| node_2: feature_name=ENST00000611977 | feature_id[28].value > threshold=10.182801246643066 |
| node_52: feature_name=ENST00000284311 | feature_id[0].value <= threshold=171.63440704345703 |
| node_53: feature_name=ENST00000529814 | feature_id[22].value <= threshold=208.89944458007812 |
| node_54: feature_name=ENST00000464591 | feature_id[14].value > threshold=95.9560661315918 |
| node_68: feature_name=ENST00000308478 | feature_id[1].value <= threshold=118.57913589477539 |
| node_69: feature_name=ENST00000393203 | feature_id[24].value > threshold=20.071932792663574 |
| node_79: feature_name=ENST00000633685 | feature_id[7].value > threshold=3.006709098815918 |
| node_81: feature_name=ENST00000509152 | feature_id[15].value > threshold=4.73750114440918 |
| node_83: feature_name=ENST00000430223 | feature_id[17].value <= threshold=72.67713928222656 |
| node_84: feature_name=ENST00000430223 | feature_id[17].value <= threshold=62.10454177856445 |

| | |
|---|---|
| Class: former_smokers | |
| | |
| Rules_97 | passed counts:1 |
| node_0: feature_name=ENST00000284311 | feature_id[0].value <= threshold=179.44091796875 |
| node_1: feature_name=ENST00000586582 | feature_id[4].value <= threshold=8.390757083892822 |
| node_2: feature_name=ENST00000611977 | feature_id[28].value > threshold=10.182801246643066 |
| node_52: feature_name=ENST00000284311 | feature_id[0].value <= threshold=171.63440704345703 |
| node_53: feature_name=ENST00000529814 | feature_id[22].value <= threshold=208.89944458007812 |
| node_54: feature_name=ENST00000464591 | feature_id[14].value > threshold=95.9560661315918 |
| node_68: feature_name=ENST00000308478 | feature_id[1].value <= threshold=118.57913589477539 |
| node_69: feature_name=ENST00000393203 | feature_id[24].value > threshold=20.071932792663574 |
| node_79: feature_name=ENST00000633685 | feature_id[7].value > threshold=3.006709098815918 |
| node_81: feature_name=ENST00000509152 | feature_id[15].value <= threshold=4.73750114440918 |
| Class: current_smokers | |
| | |
| Rules_98 | passed counts:1 |
| node_0: feature_name=ENST00000284311 | feature_id[0].value <= threshold=179.44091796875 |
| node_1: feature_name=ENST00000586582 | feature_id[4].value <= threshold=8.390757083892822 |
| node_2: feature_name=ENST00000611977 | feature_id[28].value > threshold=10.182801246643066 |
| node_52: feature_name=ENST00000284311 | feature_id[0].value <= threshold=171.63440704345703 |
| node_53: feature_name=ENST00000529814 | feature_id[22].value <= threshold=208.89944458007812 |
| node_54: feature_name=ENST00000464591 | feature_id[14].value > threshold=95.9560661315918 |
| node_68: feature_name=ENST00000308478 | feature_id[1].value <= threshold=118.57913589477539 |
| node_69: feature_name=ENST00000393203 | feature_id[24].value <= threshold=20.071932792663574 |
| node_70: feature_name=ENST00000341184 | feature_id[20].value > threshold=55.79721641540527 |
| node_76: feature_name=ENST00000622663 | feature_id[8].value <= threshold=1.9858230352401733 |

| | |
|---|---|
| Class: current_smokers | |
| | |
| Rules_99 | passed counts:1 |
| node_0: feature_name=ENST00000284311 | feature_id[0].value <= threshold=179.44091796875 |
| node_1: feature_name=ENST00000586582 | feature_id[4].value <= threshold=8.390757083892822 |
| node_2: feature_name=ENST00000611977 | feature_id[28].value > threshold=10.182801246643066 |
| node_52: feature_name=ENST00000284311 | feature_id[0].value <= threshold=171.63440704345703 |
| node_53: feature_name=ENST00000529814 | feature_id[22].value <= threshold=208.89944458007812 |
| node_54: feature_name=ENST00000464591 | feature_id[14].value > threshold=95.9560661315918 |
| node_68: feature_name=ENST00000308478 | feature_id[1].value <= threshold=118.57913589477539 |
| node_69: feature_name=ENST00000393203 | feature_id[24].value <= threshold=20.071932792663574 |
| node_70: feature_name=ENST00000341184 | feature_id[20].value <= threshold=55.79721641540527 |
| node_71: feature_name=ENST00000430223 | feature_id[17].value > threshold=201.39640045166016 |
| node_73: feature_name=ENST00000619589 | feature_id[30].value <= threshold=169.5354232788086 |
| Class: current_smokers | |
| | |
| Rules_100 | passed counts:1 |
| node_0: feature_name=ENST00000284311 | feature_id[0].value <= threshold=179.44091796875 |
| node_1: feature_name=ENST00000586582 | feature_id[4].value <= threshold=8.390757083892822 |
| node_2: feature_name=ENST00000611977 | feature_id[28].value > threshold=10.182801246643066 |
| node_52: feature_name=ENST00000284311 | feature_id[0].value <= threshold=171.63440704345703 |
| node_53: feature_name=ENST00000529814 | feature_id[22].value <= threshold=208.89944458007812 |
| node_54: feature_name=ENST00000464591 | feature_id[14].value <= threshold=95.9560661315918 |
| node_55: feature_name=ENST00000316418 | feature_id[2].value > threshold=8.205271005630493 |
| node_65: feature_name=ENST00000367467 | feature_id[3].value <= threshold=31.65692138671875 |
| Class: former_smokers | |

| | |
|---|---|
| Rules_101 | passed counts:1 |
| node_0: feature_name=ENST00000284311 | feature_id[0].value <= threshold=179.44091796875 |
| node_1: feature_name=ENST00000586582 | feature_id[4].value <= threshold=8.390757083892822 |
| node_2: feature_name=ENST00000611977 | feature_id[28].value > threshold=10.182801246643066 |
| node_52: feature_name=ENST00000284311 | feature_id[0].value <= threshold=171.63440704345703 |
| node_53: feature_name=ENST00000529814 | feature_id[22].value <= threshold=208.89944458007812 |
| node_54: feature_name=ENST00000464591 | feature_id[14].value <= threshold=95.9560661315918 |
| node_55: feature_name=ENST00000316418 | feature_id[2].value <= threshold=8.205271005630493 |
| node_56: feature_name=ENST00000509152 | feature_id[15].value > threshold=3.3449586629867554 |
| node_58: feature_name=ENST00000339223 | feature_id[5].value > threshold=29.682518005371094 |
| Class: current_smokers | |
| | |
| Rules_102 | passed counts:1 |
| node_0: feature_name=ENST00000284311 | feature_id[0].value <= threshold=179.44091796875 |
| node_1: feature_name=ENST00000586582 | feature_id[4].value <= threshold=8.390757083892822 |
| node_2: feature_name=ENST00000611977 | feature_id[28].value > threshold=10.182801246643066 |
| node_52: feature_name=ENST00000284311 | feature_id[0].value <= threshold=171.63440704345703 |
| node_53: feature_name=ENST00000529814 | feature_id[22].value <= threshold=208.89944458007812 |
| node_54: feature_name=ENST00000464591 | feature_id[14].value <= threshold=95.9560661315918 |
| node_55: feature_name=ENST00000316418 | feature_id[2].value <= threshold=8.205271005630493 |
| node_56: feature_name=ENST00000509152 | feature_id[15].value > threshold=3.3449586629867554 |
| node_58: feature_name=ENST00000339223 | feature_id[5].value <= threshold=29.682518005371094 |
| node_59: feature_name=ENST00000441556 | feature_id[16].value > threshold=5.501528263092041 |
| node_61: feature_name=ENST00000359228 | feature_id[6].value > threshold=0.4900493323802948 |
| Class: former_smokers | |

| | |
|---|---|
| Rules_103 | passed counts:1 |
| node_0: feature_name=ENST00000284311 | feature_id[0].value <= threshold=179.44091796875 |
| node_1: feature_name=ENST00000586582 | feature_id[4].value <= threshold=8.390757083892822 |
| node_2: feature_name=ENST00000611977 | feature_id[28].value > threshold=10.182801246643066 |
| node_52: feature_name=ENST00000284311 | feature_id[0].value <= threshold=171.63440704345703 |
| node_53: feature_name=ENST00000529814 | feature_id[22].value <= threshold=208.89944458007812 |
| node_54: feature_name=ENST00000464591 | feature_id[14].value <= threshold=95.9560661315918 |
| node_55: feature_name=ENST00000316418 | feature_id[2].value <= threshold=8.205271005630493 |
| node_56: feature_name=ENST00000509152 | feature_id[15].value > threshold=3.3449586629867554 |
| node_58: feature_name=ENST00000339223 | feature_id[5].value <= threshold=29.682518005371094 |
| node_59: feature_name=ENST00000441556 | feature_id[16].value > threshold=5.501528263092041 |
| node_61: feature_name=ENST00000359228 | feature_id[6].value <= threshold=0.4900493323802948 |
| Class: current_smokers | |
| | |
| Rules_104 | passed counts:1 |
| node_0: feature_name=ENST00000284311 | feature_id[0].value <= threshold=179.44091796875 |
| node_1: feature_name=ENST00000586582 | feature_id[4].value <= threshold=8.390757083892822 |
| node_2: feature_name=ENST00000611977 | feature_id[28].value > threshold=10.182801246643066 |
| node_52: feature_name=ENST00000284311 | feature_id[0].value <= threshold=171.63440704345703 |
| node_53: feature_name=ENST00000529814 | feature_id[22].value <= threshold=208.89944458007812 |
| node_54: feature_name=ENST00000464591 | feature_id[14].value <= threshold=95.9560661315918 |
| node_55: feature_name=ENST00000316418 | feature_id[2].value <= threshold=8.205271005630493 |
| node_56: feature_name=ENST00000509152 | feature_id[15].value <= threshold=3.3449586629867554 |
| Class: current_smokers | |
| | |

| | |
|---|---|
| Rules_105 | passed counts:1 |
| node_0: feature_name=ENST00000284311 | feature_id[0].value <= threshold=179.44091796875 |
| node_1: feature_name=ENST00000586582 | feature_id[4].value <= threshold=8.390757083892822 |
| node_2: feature_name=ENST00000611977 | feature_id[28].value <= threshold=10.182801246643066 |
| node_3: feature_name=ENST00000284311 | feature_id[0].value > threshold=119.33577728271484 |
| node_33: feature_name=ENST00000321016 | feature_id[25].value > threshold=10.15928602218628 |
| node_49: feature_name=ENST00000464835 | feature_id[29].value <= threshold=0.5748887062072754 |
| Class: former_smokers | |
| | |
| Rules_106 | passed counts:1 |
| node_0: feature_name=ENST00000284311 | feature_id[0].value <= threshold=179.44091796875 |
| node_1: feature_name=ENST00000586582 | feature_id[4].value <= threshold=8.390757083892822 |
| node_2: feature_name=ENST00000611977 | feature_id[28].value <= threshold=10.182801246643066 |
| node_3: feature_name=ENST00000284311 | feature_id[0].value > threshold=119.33577728271484 |
| node_33: feature_name=ENST00000321016 | feature_id[25].value <= threshold=10.15928602218628 |
| node_34: feature_name=ENST00000622663 | feature_id[8].value > threshold=10.966196537017822 |
| node_46: feature_name=ENST00000414455 | feature_id[23].value > threshold=33.80800819396973 |
| Class: former_smokers | |
| | |
| Rules_107 | passed counts:1 |
| node_0: feature_name=ENST00000284311 | feature_id[0].value <= threshold=179.44091796875 |
| node_1: feature_name=ENST00000586582 | feature_id[4].value <= threshold=8.390757083892822 |
| node_2: feature_name=ENST00000611977 | feature_id[28].value <= threshold=10.182801246643066 |
| node_3: feature_name=ENST00000284311 | feature_id[0].value > threshold=119.33577728271484 |
| node_33: feature_name=ENST00000321016 | feature_id[25].value <= threshold=10.15928602218628 |
| node_34: feature_name=ENST00000622663 | feature_id[8].value <= threshold=10.966196537017822 |

| | |
|---|---|
| node_35: feature_name=ENST00000423064 | feature_id[31].value > threshold=9.336776733398438 |
| node_39: feature_name=ENST00000422622 | feature_id[27].value > threshold=32.34660339355469 |
| Class: current_smokers | |
| | |
| Rules_108 | passed counts:1 |
| node_0: feature_name=ENST00000284311 | feature_id[0].value <= threshold=179.44091796875 |
| node_1: feature_name=ENST00000586582 | feature_id[4].value <= threshold=8.390757083892822 |
| node_2: feature_name=ENST00000611977 | feature_id[28].value <= threshold=10.182801246643066 |
| node_3: feature_name=ENST00000284311 | feature_id[0].value > threshold=119.33577728271484 |
| node_33: feature_name=ENST00000321016 | feature_id[25].value <= threshold=10.15928602218628 |
| node_34: feature_name=ENST00000622663 | feature_id[8].value <= threshold=10.966196537017822 |
| node_35: feature_name=ENST00000423064 | feature_id[31].value > threshold=9.336776733398438 |
| node_39: feature_name=ENST00000422622 | feature_id[27].value <= threshold=32.34660339355469 |
| node_40: feature_name=ENST00000367467 | feature_id[3].value <= threshold=13.330858707427979 |
| node_41: feature_name=ENST00000441556 | feature_id[16].value > threshold=1.4600719213485718 |
| Class: current_smokers | |
| | |
| Rules_109 | passed counts:1 |
| node_0: feature_name=ENST00000284311 | feature_id[0].value <= threshold=179.44091796875 |
| node_1: feature_name=ENST00000586582 | feature_id[4].value <= threshold=8.390757083892822 |
| node_2: feature_name=ENST00000611977 | feature_id[28].value <= threshold=10.182801246643066 |
| node_3: feature_name=ENST00000284311 | feature_id[0].value > threshold=119.33577728271484 |
| node_33: feature_name=ENST00000321016 | feature_id[25].value <= threshold=10.15928602218628 |
| node_34: feature_name=ENST00000622663 | feature_id[8].value <= threshold=10.966196537017822 |
| node_35: feature_name=ENST00000423064 | feature_id[31].value <= threshold=9.336776733398438 |
| node_36: feature_name=ENST00000316418 | feature_id[2].value <= threshold=4.905639350414276 |

| | |
|---|---|
| Class: former_smokers | |
| | |
| Rules_110 | passed counts:1 |
| node_0: feature_name=ENST00000284311 | feature_id[0].value <= threshold=179.44091796875 |
| node_1: feature_name=ENST00000586582 | feature_id[4].value <= threshold=8.390757083892822 |
| node_2: feature_name=ENST00000611977 | feature_id[28].value <= threshold=10.182801246643066 |
| node_3: feature_name=ENST00000284311 | feature_id[0].value <= threshold=119.33577728271484 |
| node_4: feature_name=ENST00000464591 | feature_id[14].value > threshold=92.34555053710938 |
| node_12: feature_name=ENST00000321016 | feature_id[25].value > threshold=35.220298767089844 |
| Class: current_smokers | |
| | |
| Rules_111 | passed counts:1 |
| node_0: feature_name=ENST00000284311 | feature_id[0].value <= threshold=179.44091796875 |
| node_1: feature_name=ENST00000586582 | feature_id[4].value <= threshold=8.390757083892822 |
| node_2: feature_name=ENST00000611977 | feature_id[28].value <= threshold=10.182801246643066 |
| node_3: feature_name=ENST00000284311 | feature_id[0].value <= threshold=119.33577728271484 |
| node_4: feature_name=ENST00000464591 | feature_id[14].value > threshold=92.34555053710938 |
| node_12: feature_name=ENST00000321016 | feature_id[25].value <= threshold=35.220298767089844 |
| node_13: feature_name=ENST00000392054 | feature_id[9].value > threshold=134.09820556640625 |
| Class: current_smokers | |
| | |
| Rules_112 | passed counts:1 |
| node_0: feature_name=ENST00000284311 | feature_id[0].value <= threshold=179.44091796875 |
| node_1: feature_name=ENST00000586582 | feature_id[4].value <= threshold=8.390757083892822 |
| node_2: feature_name=ENST00000611977 | feature_id[28].value <= threshold=10.182801246643066 |
| node_3: feature_name=ENST00000284311 | feature_id[0].value <= threshold=119.33577728271484 |

| | |
|---|---|
| node_4: feature_name=ENST00000464591 | feature_id[14].value > threshold=92.34555053710938 |
| node_12: feature_name=ENST00000321016 | feature_id[25].value <= threshold=35.220298767089844 |
| node_13: feature_name=ENST00000392054 | feature_id[9].value <= threshold=134.09820556640625 |
| node_14: feature_name=ENST00000430223 | feature_id[17].value > threshold=3.632150173187256 |
| node_18: feature_name=ENST00000359228 | feature_id[6].value > threshold=18.617298126220703 |
| node_28: feature_name=ENST00000622663 | feature_id[8].value > threshold=1.2085195779800415 |
| Class: former_smokers | |
| | |
| Rules_113 | passed counts:1 |
| node_0: feature_name=ENST00000284311 | feature_id[0].value <= threshold=179.44091796875 |
| node_1: feature_name=ENST00000586582 | feature_id[4].value <= threshold=8.390757083892822 |
| node_2: feature_name=ENST00000611977 | feature_id[28].value <= threshold=10.182801246643066 |
| node_3: feature_name=ENST00000284311 | feature_id[0].value <= threshold=119.33577728271484 |
| node_4: feature_name=ENST00000464591 | feature_id[14].value > threshold=92.34555053710938 |
| node_12: feature_name=ENST00000321016 | feature_id[25].value <= threshold=35.220298767089844 |
| node_13: feature_name=ENST00000392054 | feature_id[9].value <= threshold=134.09820556640625 |
| node_14: feature_name=ENST00000430223 | feature_id[17].value > threshold=3.632150173187256 |
| node_18: feature_name=ENST00000359228 | feature_id[6].value > threshold=18.617298126220703 |
| node_28: feature_name=ENST00000622663 | feature_id[8].value <= threshold=1.2085195779800415 |
| Class: current_smokers | |
| | |
| Rules_114 | passed counts:1 |
| node_0: feature_name=ENST00000284311 | feature_id[0].value <= threshold=179.44091796875 |
| node_1: feature_name=ENST00000586582 | feature_id[4].value <= threshold=8.390757083892822 |
| node_2: feature_name=ENST00000611977 | feature_id[28].value <= threshold=10.182801246643066 |
| node_3: feature_name=ENST00000284311 | feature_id[0].value <= threshold=119.33577728271484 |

| | |
|---|---|
| node_4: feature_name=ENST00000464591 | feature_id[14].value > threshold=92.34555053710938 |
| node_12: feature_name=ENST00000321016 | feature_id[25].value <= threshold=35.220298767089844 |
| node_13: feature_name=ENST00000392054 | feature_id[9].value <= threshold=134.09820556640625 |
| node_14: feature_name=ENST00000430223 | feature_id[17].value > threshold=3.632150173187256 |
| node_18: feature_name=ENST00000359228 | feature_id[6].value <= threshold=18.617298126220703 |
| node_19: feature_name=ENST00000598234 | feature_id[10].value > threshold=934.7086486816406 |
| node_25: feature_name=ENST00000316418 | feature_id[2].value > threshold=2.8290149569511414 |
| Class: current_smokers | |
| | |
| Rules_115 | passed counts:1 |
| node_0: feature_name=ENST00000284311 | feature_id[0].value <= threshold=179.44091796875 |
| node_1: feature_name=ENST00000586582 | feature_id[4].value <= threshold=8.390757083892822 |
| node_2: feature_name=ENST00000611977 | feature_id[28].value <= threshold=10.182801246643066 |
| node_3: feature_name=ENST00000284311 | feature_id[0].value <= threshold=119.33577728271484 |
| node_4: feature_name=ENST00000464591 | feature_id[14].value > threshold=92.34555053710938 |
| node_12: feature_name=ENST00000321016 | feature_id[25].value <= threshold=35.220298767089844 |
| node_13: feature_name=ENST00000392054 | feature_id[9].value <= threshold=134.09820556640625 |
| node_14: feature_name=ENST00000430223 | feature_id[17].value > threshold=3.632150173187256 |
| node_18: feature_name=ENST00000359228 | feature_id[6].value <= threshold=18.617298126220703 |
| node_19: feature_name=ENST00000598234 | feature_id[10].value > threshold=934.7086486816406 |
| node_25: feature_name=ENST00000316418 | feature_id[2].value <= threshold=2.8290149569511414 |
| Class: former_smokers | |
| | |
| Rules_116 | passed counts:1 |
| node_0: feature_name=ENST00000284311 | feature_id[0].value <= threshold=179.44091796875 |
| node_1: feature_name=ENST00000586582 | feature_id[4].value <= threshold=8.390757083892822 |

| | |
|---|---|
| node_2: feature_name=ENST00000611977 | feature_id[28].value <= threshold=10.182801246643066 |
| node_3: feature_name=ENST00000284311 | feature_id[0].value <= threshold=119.33577728271484 |
| node_4: feature_name=ENST00000464591 | feature_id[14].value > threshold=92.34555053710938 |
| node_12: feature_name=ENST00000321016 | feature_id[25].value <= threshold=35.220298767089844 |
| node_13: feature_name=ENST00000392054 | feature_id[9].value <= threshold=134.09820556640625 |
| node_14: feature_name=ENST00000430223 | feature_id[17].value <= threshold=3.632150173187256 |
| node_15: feature_name=ENST00000619589 | feature_id[30].value > threshold=79.59464263916016 |
| Class: former_smokers | |
| | |
| Rules_117 | passed counts:1 |
| node_0: feature_name=ENST00000284311 | feature_id[0].value <= threshold=179.44091796875 |
| node_1: feature_name=ENST00000586582 | feature_id[4].value <= threshold=8.390757083892822 |
| node_2: feature_name=ENST00000611977 | feature_id[28].value <= threshold=10.182801246643066 |
| node_3: feature_name=ENST00000284311 | feature_id[0].value <= threshold=119.33577728271484 |
| node_4: feature_name=ENST00000464591 | feature_id[14].value > threshold=92.34555053710938 |
| node_12: feature_name=ENST00000321016 | feature_id[25].value <= threshold=35.220298767089844 |
| node_13: feature_name=ENST00000392054 | feature_id[9].value <= threshold=134.09820556640625 |
| node_14: feature_name=ENST00000430223 | feature_id[17].value <= threshold=3.632150173187256 |
| node_15: feature_name=ENST00000619589 | feature_id[30].value <= threshold=79.59464263916016 |
| Class: current_smokers | |

(4) Rules on LASSO feature ranking results

| | |
|---|---|
| Rules_0 | passed counts:415 |
| node_0: feature_name=ENST00000284311 | feature_id[369].value <= threshold=179.44091796875 |
| node_1: feature_name=ENST00000390539 | feature_id[61].value <= threshold=11.91484260559082 |

| | |
|---|---|
| node_2: feature_name=ENST00000586582 | feature_id[365].value <= threshold=17.368224143981934 |
| node_3: feature_name=ENST00000280258 | feature_id[147].value > threshold=75.14949798583984 |
| node_35: feature_name=ENST00000395002 | feature_id[2].value > threshold=25.20395278930664 |
| node_37: feature_name=ENST00000650242 | feature_id[185].value > threshold=1343.1289672851562 |
| node_41: feature_name=ENST00000290866 | feature_id[335].value <= threshold=12.690921306610107 |
| node_42: feature_name=ENST00000509697 | feature_id[272].value > threshold=2.719158351421356 |
| node_44: feature_name=ENST00000321016 | feature_id[364].value <= threshold=35.50823783874512 |
| node_45: feature_name=ENST00000394718 | feature_id[110].value > threshold=15.798832416534424 |
| node_49: feature_name=ENST00000610495 | feature_id[298].value > threshold=8.27418327331543 |
| node_53: feature_name=ENST00000502981 | feature_id[292].value > threshold=2.636757969856262 |
| node_57: feature_name=ENST00000392054 | feature_id[368].value <= threshold=134.09820556640625 |
| node_58: feature_name=ENST00000278919 | feature_id[149].value <= threshold=45.06623077392578 |
| node_59: feature_name=ENST00000610495 | feature_id[298].value > threshold=22.136420249938965 |
| node_63: feature_name=ENST00000393590 | feature_id[350].value <= threshold=9.31120491027832 |
| Class: former_smokers | |
| | |
| Rules_1 | passed counts:181 |
| node_0: feature_name=ENST00000284311 | feature_id[369].value > threshold=179.44091796875 |
| node_98: feature_name=ENST00000396276 | feature_id[33].value <= threshold=957.0642700195312 |
| node_99: feature_name=ENST00000316418 | feature_id[348].value > threshold=3.5686380863189697 |
| node_115: feature_name=ENST00000309575 | feature_id[233].value <= threshold=375.328125 |
| node_116: feature_name=ENST00000367467 | feature_id[367].value > threshold=21.34785747528076 |
| node_120: feature_name=ENST00000571489 | feature_id[244].value <= threshold=56.20589256286621 |
| node_121: feature_name=ENST00000635923 | feature_id[304].value <= threshold=328.1167755126953 |
| node_122: feature_name=ENST00000506927 | feature_id[329].value > threshold=24.940939903259277 |

| | |
|---|---|
| node_124: feature_name=ENST00000555619 | feature_id[254].value > threshold=105.99102401733398 |
| node_128: feature_name=ENST00000380672 | feature_id[170].value <= threshold=33.95133399963379 |
| Class: current_smokers | |
| | |
| Rules_2 | passed counts:103 |
| node_0: feature_name=ENST00000284311 | feature_id[369].value <= threshold=179.44091796875 |
| node_1: feature_name=ENST00000390539 | feature_id[61].value <= threshold=11.91484260559082 |
| node_2: feature_name=ENST00000586582 | feature_id[365].value <= threshold=17.368224143981934 |
| node_3: feature_name=ENST00000280258 | feature_id[147].value <= threshold=75.14949798583984 |
| node_4: feature_name=ENST00000284311 | feature_id[369].value <= threshold=100.91744613647461 |
| node_5: feature_name=ENST00000492167 | feature_id[84].value <= threshold=164.934326171875 |
| node_6: feature_name=ENST00000329099 | feature_id[211].value > threshold=289.5108947753906 |
| node_8: feature_name=ENST00000491977 | feature_id[91].value <= threshold=5.64292573928833 |
| node_9: feature_name=ENST00000483295 | feature_id[237].value > threshold=82.8672981262207 |
| node_11: feature_name=ENST00000276974 | feature_id[251].value <= threshold=19.783329486846924 |
| node_12: feature_name=ENST00000390256 | feature_id[69].value <= threshold=4.867078065872192 |
| Class: former_smokers | |
| | |
| Rules_3 | passed counts:62 |
| node_0: feature_name=ENST00000284311 | feature_id[369].value > threshold=179.44091796875 |
| node_98: feature_name=ENST00000396276 | feature_id[33].value > threshold=957.0642700195312 |
| node_148: feature_name=ENST00000284311 | feature_id[369].value <= threshold=364.31639099121094 |
| node_149: feature_name=ENST00000276974 | feature_id[251].value <= threshold=1.20855313539505 |
| node_150: feature_name=ENST00000420843 | feature_id[247].value > threshold=338.93580627441406 |
| node_152: feature_name=ENST00000643024 | feature_id[281].value > threshold=18.539386749267578 |
| node_154: feature_name=ENST00000367051 | feature_id[359].value <= threshold=176.94873046875 |

| | |
|---|---|
| Class: former_smokers | |
| | |
| Rules_4 | passed counts:55 |
| node_0: feature_name=ENST00000284311 | feature_id[369].value > threshold=179.44091796875 |
| node_98: feature_name=ENST00000396276 | feature_id[33].value > threshold=957.0642700195312 |
| node_148: feature_name=ENST00000284311 | feature_id[369].value > threshold=364.31639099121094 |
| node_168: feature_name=ENST00000339223 | feature_id[361].value > threshold=33.90394592285156 |
| node_174: feature_name=ENST00000513886 | feature_id[309].value <= threshold=141.72513961791992 |
| node_175: feature_name=ENST00000390305 | feature_id[88].value <= threshold=495.3319091796875 |
| node_176: feature_name=ENST00000291576 | feature_id[206].value <= threshold=301.24168395996094 |
| Class: current_smokers | |
| | |
| Rules_5 | passed counts:37 |
| node_0: feature_name=ENST00000284311 | feature_id[369].value > threshold=179.44091796875 |
| node_98: feature_name=ENST00000396276 | feature_id[33].value <= threshold=957.0642700195312 |
| node_99: feature_name=ENST00000316418 | feature_id[348].value <= threshold=3.5686380863189697 |
| node_100: feature_name=ENST00000312143 | feature_id[18].value <= threshold=80.9970703125 |
| node_101: feature_name=ENST00000610349 | feature_id[73].value > threshold=6.537376165390015 |
| node_103: feature_name=ENST00000423064 | feature_id[280].value <= threshold=22.101216316223145 |
| node_104: feature_name=ENST00000513886 | feature_id[309].value <= threshold=133.05960845947266 |
| Class: current_smokers | |
| | |
| Rules_6 | passed counts:27 |
| node_0: feature_name=ENST00000284311 | feature_id[369].value <= threshold=179.44091796875 |
| node_1: feature_name=ENST00000390539 | feature_id[61].value > threshold=11.91484260559082 |
| node_83: feature_name=ENST00000565135 | feature_id[314].value > threshold=43.40609931945801 |

| | |
|---|---|
| node_87: feature_name=ENST00000544802 | feature_id[324].value > threshold=338.4650421142578 |
| node_89: feature_name=ENST00000600255 | feature_id[316].value > threshold=10.34920597076416 |
| node_95: feature_name=ENST00000390594 | feature_id[79].value > threshold=2.376459836959839 |
| Class: former_smokers | |
| | |
| Rules_7 | passed counts:21 |
| node_0: feature_name=ENST00000284311 | feature_id[369].value <= threshold=179.44091796875 |
| node_1: feature_name=ENST00000390539 | feature_id[61].value <= threshold=11.91484260559082 |
| node_2: feature_name=ENST00000586582 | feature_id[365].value <= threshold=17.368224143981934 |
| node_3: feature_name=ENST00000280258 | feature_id[147].value <= threshold=75.14949798583984 |
| node_4: feature_name=ENST00000284311 | feature_id[369].value > threshold=100.91744613647461 |
| node_22: feature_name=ENST00000367467 | feature_id[367].value > threshold=35.77326202392578 |
| node_28: feature_name=ENST00000404989 | feature_id[45].value <= threshold=50.12705612182617 |
| node_29: feature_name=ENST00000573760 | feature_id[340].value > threshold=8.433976173400879 |
| Class: current_smokers | |
| | |
| Rules_8 | passed counts:20 |
| node_0: feature_name=ENST00000284311 | feature_id[369].value > threshold=179.44091796875 |
| node_98: feature_name=ENST00000396276 | feature_id[33].value <= threshold=957.0642700195312 |
| node_99: feature_name=ENST00000316418 | feature_id[348].value > threshold=3.5686380863189697 |
| node_115: feature_name=ENST00000309575 | feature_id[233].value > threshold=375.328125 |
| node_141: feature_name=ENST00000296029 | feature_id[176].value <= threshold=753.8509216308594 |
| node_142: feature_name=ENST00000548358 | feature_id[180].value > threshold=17.743536949157715 |
| node_144: feature_name=ENST00000392055 | feature_id[356].value <= threshold=109.69507598876953 |
| Class: current_smokers | |
| | |

| | |
|---|---|
| Rules_9 | passed counts:20 |
| node_0: feature_name=ENST00000284311 | feature_id[369].value > threshold=179.44091796875 |
| node_98: feature_name=ENST00000396276 | feature_id[33].value <= threshold=957.0642700195312 |
| node_99: feature_name=ENST00000316418 | feature_id[348].value <= threshold=3.5686380863189697 |
| node_100: feature_name=ENST00000312143 | feature_id[18].value > threshold=80.9970703125 |
| node_108: feature_name=ENST00000424347 | feature_id[157].value > threshold=279.8864288330078 |
| node_112: feature_name=ENST00000306051 | feature_id[144].value <= threshold=437.27049255371094 |
| Class: former_smokers | |
| | |
| Rules_10 | passed counts:20 |
| node_0: feature_name=ENST00000284311 | feature_id[369].value <= threshold=179.44091796875 |
| node_1: feature_name=ENST00000390539 | feature_id[61].value <= threshold=11.91484260559082 |
| node_2: feature_name=ENST00000586582 | feature_id[365].value <= threshold=17.368224143981934 |
| node_3: feature_name=ENST00000280258 | feature_id[147].value <= threshold=75.14949798583984 |
| node_4: feature_name=ENST00000284311 | feature_id[369].value > threshold=100.91744613647461 |
| node_22: feature_name=ENST00000367467 | feature_id[367].value <= threshold=35.77326202392578 |
| node_23: feature_name=ENST00000503004 | feature_id[349].value > threshold=100.92071533203125 |
| node_25: feature_name=ENST00000396618 | feature_id[216].value <= threshold=11.128355741500854 |
| Class: former_smokers | |
| | |
| Rules_11 | passed counts:16 |
| node_0: feature_name=ENST00000284311 | feature_id[369].value <= threshold=179.44091796875 |
| node_1: feature_name=ENST00000390539 | feature_id[61].value > threshold=11.91484260559082 |
| node_83: feature_name=ENST00000565135 | feature_id[314].value <= threshold=43.40609931945801 |
| node_84: feature_name=ENST00000330953 | feature_id[240].value <= threshold=589.2798614501953 |
| Class: current_smokers | |

| | |
|---|---|
| | |
| Rules_12 | passed counts:14 |
| node_0: feature_name=ENST00000284311 | feature_id[369].value > threshold=179.44091796875 |
| node_98: feature_name=ENST00000396276 | feature_id[33].value > threshold=957.0642700195312 |
| node_148: feature_name=ENST00000284311 | feature_id[369].value > threshold=364.31639099121094 |
| node_168: feature_name=ENST00000339223 | feature_id[361].value <= threshold=33.90394592285156 |
| node_169: feature_name=ENST00000297785 | feature_id[3].value > threshold=208.11764526367188 |
| node_171: feature_name=ENST00000393203 | feature_id[352].value > threshold=4.1026811599731445 |
| Class: former_smokers | |
| | |
| Rules_13 | passed counts:13 |
| node_0: feature_name=ENST00000284311 | feature_id[369].value > threshold=179.44091796875 |
| node_98: feature_name=ENST00000396276 | feature_id[33].value > threshold=957.0642700195312 |
| node_148: feature_name=ENST00000284311 | feature_id[369].value <= threshold=364.31639099121094 |
| node_149: feature_name=ENST00000276974 | feature_id[251].value > threshold=1.20855313539505 |
| node_159: feature_name=ENST00000641136 | feature_id[58].value > threshold=5.980504512786865 |
| node_163: feature_name=ENST00000390624 | feature_id[71].value > threshold=11.1699538230896 |
| Class: current_smokers | |
| | |
| Rules_14 | passed counts:13 |
| node_0: feature_name=ENST00000284311 | feature_id[369].value > threshold=179.44091796875 |
| node_98: feature_name=ENST00000396276 | feature_id[33].value <= threshold=957.0642700195312 |
| node_99: feature_name=ENST00000316418 | feature_id[348].value > threshold=3.5686380863189697 |
| node_115: feature_name=ENST00000309575 | feature_id[233].value <= threshold=375.328125 |
| node_116: feature_name=ENST00000367467 | feature_id[367].value > threshold=21.34785747528076 |
| node_120: feature_name=ENST00000571489 | feature_id[244].value <= threshold=56.20589256286621 |

| | |
|---|---|
| node_121: feature_name=ENST00000635923 | feature_id[304].value <= threshold=328.1167755126953 |
| node_122: feature_name=ENST00000506927 | feature_id[329].value > threshold=24.940939903259277 |
| node_124: feature_name=ENST00000555619 | feature_id[254].value > threshold=105.99102401733398 |
| node_128: feature_name=ENST00000380672 | feature_id[170].value > threshold=33.95133399963379 |
| node_130: feature_name=ENST00000439754 | feature_id[183].value > threshold=341.5655822753906 |
| Class: current_smokers | |
| | |
| Rules_15 | passed counts:12 |
| node_0: feature_name=ENST00000284311 | feature_id[369].value <= threshold=179.44091796875 |
| node_1: feature_name=ENST00000390539 | feature_id[61].value <= threshold=11.91484260559082 |
| node_2: feature_name=ENST00000586582 | feature_id[365].value > threshold=17.368224143981934 |
| node_80: feature_name=ENST00000324907 | feature_id[4].value <= threshold=9.99325180053711 |
| Class: current_smokers | |
| | |
| Rules_16 | passed counts:9 |
| node_0: feature_name=ENST00000284311 | feature_id[369].value > threshold=179.44091796875 |
| node_98: feature_name=ENST00000396276 | feature_id[33].value > threshold=957.0642700195312 |
| node_148: feature_name=ENST00000284311 | feature_id[369].value <= threshold=364.31639099121094 |
| node_149: feature_name=ENST00000276974 | feature_id[251].value > threshold=1.20855313539505 |
| node_159: feature_name=ENST00000641136 | feature_id[58].value <= threshold=5.980504512786865 |
| node_160: feature_name=ENST00000414455 | feature_id[126].value > threshold=35.92243194580078 |
| Class: former_smokers | |
| | |
| Rules_17 | passed counts:8 |
| node_0: feature_name=ENST00000284311 | feature_id[369].value > threshold=179.44091796875 |
| node_98: feature_name=ENST00000396276 | feature_id[33].value <= threshold=957.0642700195312 |

| | |
|---|---|
| node_99: feature_name=ENST00000316418 | feature_id[348].value <= threshold=3.5686380863189697 |
| node_100: feature_name=ENST00000312143 | feature_id[18].value > threshold=80.9970703125 |
| node_108: feature_name=ENST00000424347 | feature_id[157].value <= threshold=279.8864288330078 |
| node_109: feature_name=ENST00000612503 | feature_id[113].value <= threshold=19.23927879333496 |
| Class: current_smokers | |
| | |
| Rules_18 | passed counts:7 |
| node_0: feature_name=ENST00000284311 | feature_id[369].value <= threshold=179.44091796875 |
| node_1: feature_name=ENST00000390539 | feature_id[61].value > threshold=11.91484260559082 |
| node_83: feature_name=ENST00000565135 | feature_id[314].value > threshold=43.40609931945801 |
| node_87: feature_name=ENST00000544802 | feature_id[324].value > threshold=338.4650421142578 |
| node_89: feature_name=ENST00000600255 | feature_id[316].value <= threshold=10.34920597076416 |
| node_90: feature_name=ENST00000443723 | feature_id[35].value <= threshold=3.926640510559082 |
| Class: current_smokers | |
| | |
| Rules_19 | passed counts:7 |
| node_0: feature_name=ENST00000284311 | feature_id[369].value <= threshold=179.44091796875 |
| node_1: feature_name=ENST00000390539 | feature_id[61].value <= threshold=11.91484260559082 |
| node_2: feature_name=ENST00000586582 | feature_id[365].value <= threshold=17.368224143981934 |
| node_3: feature_name=ENST00000280258 | feature_id[147].value > threshold=75.14949798583984 |
| node_35: feature_name=ENST00000395002 | feature_id[2].value > threshold=25.20395278930664 |
| node_37: feature_name=ENST00000650242 | feature_id[185].value > threshold=1343.1289672851562 |
| node_41: feature_name=ENST00000290866 | feature_id[335].value <= threshold=12.690921306610107 |
| node_42: feature_name=ENST00000509697 | feature_id[272].value > threshold=2.719158351421356 |
| node_44: feature_name=ENST00000321016 | feature_id[364].value <= threshold=35.50823783874512 |
| node_45: feature_name=ENST00000394718 | feature_id[110].value > threshold=15.798832416534424 |

| | |
|---|---|
| node_49: feature_name=ENST00000610495 | feature_id[298].value > threshold=8.27418327331543 |
| node_53: feature_name=ENST00000502981 | feature_id[292].value > threshold=2.636757969856262 |
| node_57: feature_name=ENST00000392054 | feature_id[368].value <= threshold=134.09820556640625 |
| node_58: feature_name=ENST00000278919 | feature_id[149].value <= threshold=45.06623077392578 |
| node_59: feature_name=ENST00000610495 | feature_id[298].value > threshold=22.136420249938965 |
| node_63: feature_name=ENST00000393590 | feature_id[350].value > threshold=9.31120491027832 |
| node_65: feature_name=ENST00000548358 | feature_id[180].value > threshold=16.836580276489258 |
| Class: former_smokers | |
| | |
| Rules_20 | passed counts:6 |
| node_0: feature_name=ENST00000284311 | feature_id[369].value > threshold=179.44091796875 |
| node_98: feature_name=ENST00000396276 | feature_id[33].value <= threshold=957.0642700195312 |
| node_99: feature_name=ENST00000316418 | feature_id[348].value > threshold=3.5686380863189697 |
| node_115: feature_name=ENST00000309575 | feature_id[233].value > threshold=375.328125 |
| node_141: feature_name=ENST00000296029 | feature_id[176].value > threshold=753.8509216308594 |
| Class: former_smokers | |
| | |
| Rules_21 | passed counts:6 |
| node_0: feature_name=ENST00000284311 | feature_id[369].value <= threshold=179.44091796875 |
| node_1: feature_name=ENST00000390539 | feature_id[61].value > threshold=11.91484260559082 |
| node_83: feature_name=ENST00000565135 | feature_id[314].value > threshold=43.40609931945801 |
| node_87: feature_name=ENST00000544802 | feature_id[324].value > threshold=338.4650421142578 |
| node_89: feature_name=ENST00000600255 | feature_id[316].value <= threshold=10.34920597076416 |
| node_90: feature_name=ENST00000443723 | feature_id[35].value > threshold=3.926640510559082 |
| node_92: feature_name=ENST00000565135 | feature_id[314].value > threshold=46.06411552429199 |
| Class: former_smokers | |

| | |
|---|---|
| Rules_22 | passed counts:6 |
| node_0: feature_name=ENST00000284311 | feature_id[369].value <= threshold=179.44091796875 |
| node_1: feature_name=ENST00000390539 | feature_id[61].value > threshold=11.91484260559082 |
| node_83: feature_name=ENST00000565135 | feature_id[314].value > threshold=43.40609931945801 |
| node_87: feature_name=ENST00000544802 | feature_id[324].value <= threshold=338.4650421142578 |
| Class: current_smokers | |
| | |
| Rules_23 | passed counts:6 |
| node_0: feature_name=ENST00000284311 | feature_id[369].value <= threshold=179.44091796875 |
| node_1: feature_name=ENST00000390539 | feature_id[61].value <= threshold=11.91484260559082 |
| node_2: feature_name=ENST00000586582 | feature_id[365].value <= threshold=17.368224143981934 |
| node_3: feature_name=ENST00000280258 | feature_id[147].value <= threshold=75.14949798583984 |
| node_4: feature_name=ENST00000284311 | feature_id[369].value > threshold=100.91744613647461 |
| node_22: feature_name=ENST00000367467 | feature_id[367].value > threshold=35.77326202392578 |
| node_28: feature_name=ENST00000404989 | feature_id[45].value > threshold=50.12705612182617 |
| Class: former_smokers | |
| | |
| Rules_24 | passed counts:5 |
| node_0: feature_name=ENST00000284311 | feature_id[369].value > threshold=179.44091796875 |
| node_98: feature_name=ENST00000396276 | feature_id[33].value > threshold=957.0642700195312 |
| node_148: feature_name=ENST00000284311 | feature_id[369].value > threshold=364.31639099121094 |
| node_168: feature_name=ENST00000339223 | feature_id[361].value <= threshold=33.90394592285156 |
| node_169: feature_name=ENST00000297785 | feature_id[3].value <= threshold=208.11764526367188 |
| Class: current_smokers | |
| | |

| | |
|---|---|
| Rules_25 | passed counts:5 |
| node_0: feature_name=ENST00000284311 | feature_id[369].value > threshold=179.44091796875 |
| node_98: feature_name=ENST00000396276 | feature_id[33].value <= threshold=957.0642700195312 |
| node_99: feature_name=ENST00000316418 | feature_id[348].value > threshold=3.5686380863189697 |
| node_115: feature_name=ENST00000309575 | feature_id[233].value <= threshold=375.328125 |
| node_116: feature_name=ENST00000367467 | feature_id[367].value > threshold=21.34785747528076 |
| node_120: feature_name=ENST00000571489 | feature_id[244].value <= threshold=56.20589256286621 |
| node_121: feature_name=ENST00000635923 | feature_id[304].value > threshold=328.1167755126953 |
| node_135: feature_name=ENST00000390548 | feature_id[346].value > threshold=3.1817190051078796 |
| Class: current_smokers | |
| | |
| Rules_26 | passed counts:5 |
| node_0: feature_name=ENST00000284311 | feature_id[369].value > threshold=179.44091796875 |
| node_98: feature_name=ENST00000396276 | feature_id[33].value <= threshold=957.0642700195312 |
| node_99: feature_name=ENST00000316418 | feature_id[348].value <= threshold=3.5686380863189697 |
| node_100: feature_name=ENST00000312143 | feature_id[18].value <= threshold=80.9970703125 |
| node_101: feature_name=ENST00000610349 | feature_id[73].value <= threshold=6.537376165390015 |
| Class: former_smokers | |
| | |
| Rules_27 | passed counts:5 |
| node_0: feature_name=ENST00000284311 | feature_id[369].value <= threshold=179.44091796875 |
| node_1: feature_name=ENST00000390539 | feature_id[61].value <= threshold=11.91484260559082 |
| node_2: feature_name=ENST00000586582 | feature_id[365].value <= threshold=17.368224143981934 |
| node_3: feature_name=ENST00000280258 | feature_id[147].value > threshold=75.14949798583984 |
| node_35: feature_name=ENST00000395002 | feature_id[2].value > threshold=25.20395278930664 |
| node_37: feature_name=ENST00000650242 | feature_id[185].value > threshold=1343.1289672851562 |

| | |
|---|---|
| node_41: feature_name=ENST00000290866 | feature_id[335].value > threshold=12.690921306610107 |
| node_77: feature_name=ENST00000477714 | feature_id[260].value > threshold=12.307570934295654 |
| Class: former_smokers | |
| | |
| Rules_28 | passed counts:5 |
| node_0: feature_name=ENST00000284311 | feature_id[369].value <= threshold=179.44091796875 |
| node_1: feature_name=ENST00000390539 | feature_id[61].value <= threshold=11.91484260559082 |
| node_2: feature_name=ENST00000586582 | feature_id[365].value <= threshold=17.368224143981934 |
| node_3: feature_name=ENST00000280258 | feature_id[147].value > threshold=75.14949798583984 |
| node_35: feature_name=ENST00000395002 | feature_id[2].value > threshold=25.20395278930664 |
| node_37: feature_name=ENST00000650242 | feature_id[185].value > threshold=1343.1289672851562 |
| node_41: feature_name=ENST00000290866 | feature_id[335].value <= threshold=12.690921306610107 |
| node_42: feature_name=ENST00000509697 | feature_id[272].value > threshold=2.719158351421356 |
| node_44: feature_name=ENST00000321016 | feature_id[364].value <= threshold=35.50823783874512 |
| node_45: feature_name=ENST00000394718 | feature_id[110].value > threshold=15.798832416534424 |
| node_49: feature_name=ENST00000610495 | feature_id[298].value > threshold=8.27418327331543 |
| node_53: feature_name=ENST00000502981 | feature_id[292].value > threshold=2.636757969856262 |
| node_57: feature_name=ENST00000392054 | feature_id[368].value <= threshold=134.09820556640625 |
| node_58: feature_name=ENST00000278919 | feature_id[149].value <= threshold=45.06623077392578 |
| node_59: feature_name=ENST00000610495 | feature_id[298].value <= threshold=22.136420249938965 |
| node_60: feature_name=ENST00000380490 | feature_id[121].value <= threshold=1292.0121765136719 |
| Class: former_smokers | |
| | |
| Rules_29 | passed counts:5 |
| node_0: feature_name=ENST00000284311 | feature_id[369].value <= threshold=179.44091796875 |
| node_1: feature_name=ENST00000390539 | feature_id[61].value <= threshold=11.91484260559082 |

| | |
|---|---|
| node_2: feature_name=ENST00000586582 | feature_id[365].value <= threshold=17.368224143981934 |
| node_3: feature_name=ENST00000280258 | feature_id[147].value <= threshold=75.14949798583984 |
| node_4: feature_name=ENST00000284311 | feature_id[369].value > threshold=100.91744613647461 |
| node_22: feature_name=ENST00000367467 | feature_id[367].value > threshold=35.77326202392578 |
| node_28: feature_name=ENST00000404989 | feature_id[45].value <= threshold=50.12705612182617 |
| node_29: feature_name=ENST00000573760 | feature_id[340].value <= threshold=8.433976173400879 |
| node_30: feature_name=ENST00000448155 | feature_id[75].value <= threshold=34.63613796234131 |
| Class: former_smokers | |
| | |
| Rules_30 | passed counts:4 |
| node_0: feature_name=ENST00000284311 | feature_id[369].value > threshold=179.44091796875 |
| node_98: feature_name=ENST00000396276 | feature_id[33].value <= threshold=957.0642700195312 |
| node_99: feature_name=ENST00000316418 | feature_id[348].value > threshold=3.5686380863189697 |
| node_115: feature_name=ENST00000309575 | feature_id[233].value > threshold=375.328125 |
| node_141: feature_name=ENST00000296029 | feature_id[176].value <= threshold=753.8509216308594 |
| node_142: feature_name=ENST00000548358 | feature_id[180].value <= threshold=17.743536949157715 |
| Class: former_smokers | |
| | |
| Rules_31 | passed counts:4 |
| node_0: feature_name=ENST00000284311 | feature_id[369].value > threshold=179.44091796875 |
| node_98: feature_name=ENST00000396276 | feature_id[33].value <= threshold=957.0642700195312 |
| node_99: feature_name=ENST00000316418 | feature_id[348].value > threshold=3.5686380863189697 |
| node_115: feature_name=ENST00000309575 | feature_id[233].value <= threshold=375.328125 |
| node_116: feature_name=ENST00000367467 | feature_id[367].value <= threshold=21.34785747528076 |
| node_117: feature_name=ENST00000513886 | feature_id[309].value <= threshold=21.074685096740723 |
| Class: former_smokers | |

| | |
|---|---|
| Rules_32 | passed counts:4 |
| node_0: feature_name=ENST00000284311 | feature_id[369].value <= threshold=179.44091796875 |
| node_1: feature_name=ENST00000390539 | feature_id[61].value <= threshold=11.91484260559082 |
| node_2: feature_name=ENST00000586582 | feature_id[365].value > threshold=17.368224143981934 |
| node_80: feature_name=ENST00000324907 | feature_id[4].value > threshold=9.99325180053711 |
| Class: former_smokers | |
| | |
| Rules_33 | passed counts:4 |
| node_0: feature_name=ENST00000284311 | feature_id[369].value <= threshold=179.44091796875 |
| node_1: feature_name=ENST00000390539 | feature_id[61].value <= threshold=11.91484260559082 |
| node_2: feature_name=ENST00000586582 | feature_id[365].value <= threshold=17.368224143981934 |
| node_3: feature_name=ENST00000280258 | feature_id[147].value > threshold=75.14949798583984 |
| node_35: feature_name=ENST00000395002 | feature_id[2].value > threshold=25.20395278930664 |
| node_37: feature_name=ENST00000650242 | feature_id[185].value > threshold=1343.1289672851562 |
| node_41: feature_name=ENST00000290866 | feature_id[335].value <= threshold=12.690921306610107 |
| node_42: feature_name=ENST00000509697 | feature_id[272].value > threshold=2.719158351421356 |
| node_44: feature_name=ENST00000321016 | feature_id[364].value <= threshold=35.50823783874512 |
| node_45: feature_name=ENST00000394718 | feature_id[110].value > threshold=15.798832416534424 |
| node_49: feature_name=ENST00000610495 | feature_id[298].value > threshold=8.27418327331543 |
| node_53: feature_name=ENST00000502981 | feature_id[292].value > threshold=2.636757969856262 |
| node_57: feature_name=ENST00000392054 | feature_id[368].value <= threshold=134.09820556640625 |
| node_58: feature_name=ENST00000278919 | feature_id[149].value > threshold=45.06623077392578 |
| node_68: feature_name=ENST00000390306 | feature_id[97].value > threshold=12.087755918502808 |
| Class: former_smokers | |
| | |

| | |
|---|---|
| Rules_34 | passed counts:4 |
| node_0: feature_name=ENST00000284311 | feature_id[369].value <= threshold=179.44091796875 |
| node_1: feature_name=ENST00000390539 | feature_id[61].value <= threshold=11.91484260559082 |
| node_2: feature_name=ENST00000586582 | feature_id[365].value <= threshold=17.368224143981934 |
| node_3: feature_name=ENST00000280258 | feature_id[147].value <= threshold=75.14949798583984 |
| node_4: feature_name=ENST00000284311 | feature_id[369].value <= threshold=100.91744613647461 |
| node_5: feature_name=ENST00000492167 | feature_id[84].value > threshold=164.934326171875 |
| Class: current_smokers | |
| | |
| Rules_35 | passed counts:3 |
| node_0: feature_name=ENST00000284311 | feature_id[369].value > threshold=179.44091796875 |
| node_98: feature_name=ENST00000396276 | feature_id[33].value > threshold=957.0642700195312 |
| node_148: feature_name=ENST00000284311 | feature_id[369].value > threshold=364.31639099121094 |
| node_168: feature_name=ENST00000339223 | feature_id[361].value > threshold=33.90394592285156 |
| node_174: feature_name=ENST00000513886 | feature_id[309].value > threshold=141.72513961791992 |
| Class: former_smokers | |
| | |
| Rules_36 | passed counts:3 |
| node_0: feature_name=ENST00000284311 | feature_id[369].value > threshold=179.44091796875 |
| node_98: feature_name=ENST00000396276 | feature_id[33].value > threshold=957.0642700195312 |
| node_148: feature_name=ENST00000284311 | feature_id[369].value <= threshold=364.31639099121094 |
| node_149: feature_name=ENST00000276974 | feature_id[251].value <= threshold=1.20855313539505 |
| node_150: feature_name=ENST00000420843 | feature_id[247].value <= threshold=338.93580627441406 |
| Class: current_smokers | |
| | |
| Rules_37 | passed counts:3 |

| | |
|---|---|
| node_0: feature_name=ENST00000284311 | feature_id[369].value > threshold=179.44091796875 |
| node_98: feature_name=ENST00000396276 | feature_id[33].value <= threshold=957.0642700195312 |
| node_99: feature_name=ENST00000316418 | feature_id[348].value > threshold=3.5686380863189697 |
| node_115: feature_name=ENST00000309575 | feature_id[233].value <= threshold=375.328125 |
| node_116: feature_name=ENST00000367467 | feature_id[367].value > threshold=21.34785747528076 |
| node_120: feature_name=ENST00000571489 | feature_id[244].value <= threshold=56.20589256286621 |
| node_121: feature_name=ENST00000635923 | feature_id[304].value > threshold=328.1167755126953 |
| node_135: feature_name=ENST00000390548 | feature_id[346].value <= threshold=3.1817190051078796 |
| Class: former_smokers | |
| | |
| Rules_38 | passed counts:3 |
| node_0: feature_name=ENST00000284311 | feature_id[369].value > threshold=179.44091796875 |
| node_98: feature_name=ENST00000396276 | feature_id[33].value <= threshold=957.0642700195312 |
| node_99: feature_name=ENST00000316418 | feature_id[348].value > threshold=3.5686380863189697 |
| node_115: feature_name=ENST00000309575 | feature_id[233].value <= threshold=375.328125 |
| node_116: feature_name=ENST00000367467 | feature_id[367].value > threshold=21.34785747528076 |
| node_120: feature_name=ENST00000571489 | feature_id[244].value <= threshold=56.20589256286621 |
| node_121: feature_name=ENST00000635923 | feature_id[304].value <= threshold=328.1167755126953 |
| node_122: feature_name=ENST00000506927 | feature_id[329].value > threshold=24.940939903259277 |
| node_124: feature_name=ENST00000555619 | feature_id[254].value > threshold=105.99102401733398 |
| node_128: feature_name=ENST00000380672 | feature_id[170].value > threshold=33.95133399963379 |
| node_130: feature_name=ENST00000439754 | feature_id[183].value <= threshold=341.5655822753906 |
| node_131: feature_name=ENST00000637526 | feature_id[286].value > threshold=120.73594665527344 |
| Class: former_smokers | |
| | |
| Rules_39 | passed counts:3 |

| | |
|---|---|
| node_0: feature_name=ENST00000284311 | feature_id[369].value > threshold=179.44091796875 |
| node_98: feature_name=ENST00000396276 | feature_id[33].value <= threshold=957.0642700195312 |
| node_99: feature_name=ENST00000316418 | feature_id[348].value > threshold=3.5686380863189697 |
| node_115: feature_name=ENST00000309575 | feature_id[233].value <= threshold=375.328125 |
| node_116: feature_name=ENST00000367467 | feature_id[367].value <= threshold=21.34785747528076 |
| node_117: feature_name=ENST00000513886 | feature_id[309].value > threshold=21.074685096740723 |
| Class: current_smokers | |
| | |
| Rules_40 | passed counts:3 |
| node_0: feature_name=ENST00000284311 | feature_id[369].value > threshold=179.44091796875 |
| node_98: feature_name=ENST00000396276 | feature_id[33].value <= threshold=957.0642700195312 |
| node_99: feature_name=ENST00000316418 | feature_id[348].value <= threshold=3.5686380863189697 |
| node_100: feature_name=ENST00000312143 | feature_id[18].value > threshold=80.9970703125 |
| node_108: feature_name=ENST00000424347 | feature_id[157].value <= threshold=279.8864288330078 |
| node_109: feature_name=ENST00000612503 | feature_id[113].value > threshold=19.23927879333496 |
| Class: former_smokers | |
| | |
| Rules_41 | passed counts:3 |
| node_0: feature_name=ENST00000284311 | feature_id[369].value <= threshold=179.44091796875 |
| node_1: feature_name=ENST00000390539 | feature_id[61].value <= threshold=11.91484260559082 |
| node_2: feature_name=ENST00000586582 | feature_id[365].value <= threshold=17.368224143981934 |
| node_3: feature_name=ENST00000280258 | feature_id[147].value > threshold=75.14949798583984 |
| node_35: feature_name=ENST00000395002 | feature_id[2].value > threshold=25.20395278930664 |
| node_37: feature_name=ENST00000650242 | feature_id[185].value > threshold=1343.1289672851562 |
| node_41: feature_name=ENST00000290866 | feature_id[335].value > threshold=12.690921306610107 |
| node_77: feature_name=ENST00000477714 | feature_id[260].value <= threshold=12.307570934295654 |

| | |
|---|---|
| Class: current_smokers | |
| | |
| Rules_42 | passed counts:3 |
| node_0: feature_name=ENST00000284311 | feature_id[369].value <= threshold=179.44091796875 |
| node_1: feature_name=ENST00000390539 | feature_id[61].value <= threshold=11.91484260559082 |
| node_2: feature_name=ENST00000586582 | feature_id[365].value <= threshold=17.368224143981934 |
| node_3: feature_name=ENST00000280258 | feature_id[147].value > threshold=75.14949798583984 |
| node_35: feature_name=ENST00000395002 | feature_id[2].value <= threshold=25.20395278930664 |
| Class: current_smokers | |
| | |
| Rules_43 | passed counts:3 |
| node_0: feature_name=ENST00000284311 | feature_id[369].value <= threshold=179.44091796875 |
| node_1: feature_name=ENST00000390539 | feature_id[61].value <= threshold=11.91484260559082 |
| node_2: feature_name=ENST00000586582 | feature_id[365].value <= threshold=17.368224143981934 |
| node_3: feature_name=ENST00000280258 | feature_id[147].value <= threshold=75.14949798583984 |
| node_4: feature_name=ENST00000284311 | feature_id[369].value > threshold=100.91744613647461 |
| node_22: feature_name=ENST00000367467 | feature_id[367].value <= threshold=35.77326202392578 |
| node_23: feature_name=ENST00000503004 | feature_id[349].value <= threshold=100.92071533203125 |
| Class: current_smokers | |
| | |
| Rules_44 | passed counts:2 |
| node_0: feature_name=ENST00000284311 | feature_id[369].value > threshold=179.44091796875 |
| node_98: feature_name=ENST00000396276 | feature_id[33].value > threshold=957.0642700195312 |
| node_148: feature_name=ENST00000284311 | feature_id[369].value <= threshold=364.31639099121094 |
| node_149: feature_name=ENST00000276974 | feature_id[251].value > threshold=1.20855313539505 |
| node_159: feature_name=ENST00000641136 | feature_id[58].value > threshold=5.980504512786865 |

| | |
|---|---|
| node_163: feature_name=ENST00000390624 | feature_id[71].value <= threshold=11.1699538230896 |
| node_164: feature_name=ENST00000492446 | feature_id[83].value > threshold=0.6932542324066162 |
| Class: former_smokers | |
| | |
| Rules_45 | passed counts:2 |
| node_0: feature_name=ENST00000284311 | feature_id[369].value > threshold=179.44091796875 |
| node_98: feature_name=ENST00000396276 | feature_id[33].value > threshold=957.0642700195312 |
| node_148: feature_name=ENST00000284311 | feature_id[369].value <= threshold=364.31639099121094 |
| node_149: feature_name=ENST00000276974 | feature_id[251].value > threshold=1.20855313539505 |
| node_159: feature_name=ENST00000641136 | feature_id[58].value <= threshold=5.980504512786865 |
| node_160: feature_name=ENST00000414455 | feature_id[126].value <= threshold=35.92243194580078 |
| Class: current_smokers | |
| | |
| Rules_46 | passed counts:2 |
| node_0: feature_name=ENST00000284311 | feature_id[369].value > threshold=179.44091796875 |
| node_98: feature_name=ENST00000396276 | feature_id[33].value > threshold=957.0642700195312 |
| node_148: feature_name=ENST00000284311 | feature_id[369].value <= threshold=364.31639099121094 |
| node_149: feature_name=ENST00000276974 | feature_id[251].value <= threshold=1.20855313539505 |
| node_150: feature_name=ENST00000420843 | feature_id[247].value > threshold=338.93580627441406 |
| node_152: feature_name=ENST00000643024 | feature_id[281].value > threshold=18.539386749267578 |
| node_154: feature_name=ENST00000367051 | feature_id[359].value > threshold=176.94873046875 |
| node_156: feature_name=ENST00000526097 | feature_id[294].value <= threshold=40.1702938079834 |
| Class: current_smokers | |
| | |
| Rules_47 | passed counts:2 |
| node_0: feature_name=ENST00000284311 | feature_id[369].value > threshold=179.44091796875 |

| | |
|---|---|
| node_98: feature_name=ENST00000396276 | feature_id[33].value > threshold=957.0642700195312 |
| node_148: feature_name=ENST00000284311 | feature_id[369].value <= threshold=364.31639099121094 |
| node_149: feature_name=ENST00000276974 | feature_id[251].value <= threshold=1.20855313539505 |
| node_150: feature_name=ENST00000420843 | feature_id[247].value > threshold=338.93580627441406 |
| node_152: feature_name=ENST00000643024 | feature_id[281].value <= threshold=18.539386749267578 |
| Class: current_smokers | |
| | |
| Rules_48 | passed counts:2 |
| node_0: feature_name=ENST00000284311 | feature_id[369].value > threshold=179.44091796875 |
| node_98: feature_name=ENST00000396276 | feature_id[33].value <= threshold=957.0642700195312 |
| node_99: feature_name=ENST00000316418 | feature_id[348].value > threshold=3.5686380863189697 |
| node_115: feature_name=ENST00000309575 | feature_id[233].value > threshold=375.328125 |
| node_141: feature_name=ENST00000296029 | feature_id[176].value <= threshold=753.8509216308594 |
| node_142: feature_name=ENST00000548358 | feature_id[180].value > threshold=17.743536949157715 |
| node_144: feature_name=ENST00000392055 | feature_id[356].value > threshold=109.69507598876953 |
| Class: former_smokers | |
| | |
| Rules_49 | passed counts:2 |
| node_0: feature_name=ENST00000284311 | feature_id[369].value > threshold=179.44091796875 |
| node_98: feature_name=ENST00000396276 | feature_id[33].value <= threshold=957.0642700195312 |
| node_99: feature_name=ENST00000316418 | feature_id[348].value > threshold=3.5686380863189697 |
| node_115: feature_name=ENST00000309575 | feature_id[233].value <= threshold=375.328125 |
| node_116: feature_name=ENST00000367467 | feature_id[367].value > threshold=21.34785747528076 |
| node_120: feature_name=ENST00000571489 | feature_id[244].value > threshold=56.20589256286621 |
| node_138: feature_name=ENST00000422622 | feature_id[357].value <= threshold=9.033727169036865 |
| Class: former_smokers | |

| | |
|---|---|
| Rules_50 | passed counts:2 |
| node_0: feature_name=ENST00000284311 | feature_id[369].value > threshold=179.44091796875 |
| node_98: feature_name=ENST00000396276 | feature_id[33].value <= threshold=957.0642700195312 |
| node_99: feature_name=ENST00000316418 | feature_id[348].value <= threshold=3.5686380863189697 |
| node_100: feature_name=ENST00000312143 | feature_id[18].value > threshold=80.9970703125 |
| node_108: feature_name=ENST00000424347 | feature_id[157].value > threshold=279.8864288330078 |
| node_112: feature_name=ENST00000306051 | feature_id[144].value > threshold=437.27049255371094 |
| Class: current_smokers | |
| | |
| Rules_51 | passed counts:2 |
| node_0: feature_name=ENST00000284311 | feature_id[369].value > threshold=179.44091796875 |
| node_98: feature_name=ENST00000396276 | feature_id[33].value <= threshold=957.0642700195312 |
| node_99: feature_name=ENST00000316418 | feature_id[348].value <= threshold=3.5686380863189697 |
| node_100: feature_name=ENST00000312143 | feature_id[18].value <= threshold=80.9970703125 |
| node_101: feature_name=ENST00000610349 | feature_id[73].value > threshold=6.537376165390015 |
| node_103: feature_name=ENST00000423064 | feature_id[280].value > threshold=22.101216316223145 |
| Class: former_smokers | |
| | |
| Rules_52 | passed counts:2 |
| node_0: feature_name=ENST00000284311 | feature_id[369].value <= threshold=179.44091796875 |
| node_1: feature_name=ENST00000390539 | feature_id[61].value <= threshold=11.91484260559082 |
| node_2: feature_name=ENST00000586582 | feature_id[365].value <= threshold=17.368224143981934 |
| node_3: feature_name=ENST00000280258 | feature_id[147].value > threshold=75.14949798583984 |
| node_35: feature_name=ENST00000395002 | feature_id[2].value > threshold=25.20395278930664 |
| node_37: feature_name=ENST00000650242 | feature_id[185].value > threshold=1343.1289672851562 |

| | |
|---|---|
| node_41: feature_name=ENST00000290866 | feature_id[335].value <= threshold=12.690921306610107 |
| node_42: feature_name=ENST00000509697 | feature_id[272].value > threshold=2.719158351421356 |
| node_44: feature_name=ENST00000321016 | feature_id[364].value <= threshold=35.50823783874512 |
| node_45: feature_name=ENST00000394718 | feature_id[110].value > threshold=15.798832416534424 |
| node_49: feature_name=ENST00000610495 | feature_id[298].value > threshold=8.27418327331543 |
| node_53: feature_name=ENST00000502981 | feature_id[292].value > threshold=2.636757969856262 |
| node_57: feature_name=ENST00000392054 | feature_id[368].value > threshold=134.09820556640625 |
| node_71: feature_name=ENST00000390305 | feature_id[88].value > threshold=6.945567846298218 |
| Class: former_smokers | |
| | |
| Rules_53 | passed counts:2 |
| node_0: feature_name=ENST00000284311 | feature_id[369].value <= threshold=179.44091796875 |
| node_1: feature_name=ENST00000390539 | feature_id[61].value <= threshold=11.91484260559082 |
| node_2: feature_name=ENST00000586582 | feature_id[365].value <= threshold=17.368224143981934 |
| node_3: feature_name=ENST00000280258 | feature_id[147].value > threshold=75.14949798583984 |
| node_35: feature_name=ENST00000395002 | feature_id[2].value > threshold=25.20395278930664 |
| node_37: feature_name=ENST00000650242 | feature_id[185].value <= threshold=1343.1289672851562 |
| node_38: feature_name=ENST00000260526 | feature_id[139].value <= threshold=10.508224964141846 |
| Class: current_smokers | |
| | |
| Rules_54 | passed counts:2 |
| node_0: feature_name=ENST00000284311 | feature_id[369].value <= threshold=179.44091796875 |
| node_1: feature_name=ENST00000390539 | feature_id[61].value <= threshold=11.91484260559082 |
| node_2: feature_name=ENST00000586582 | feature_id[365].value <= threshold=17.368224143981934 |
| node_3: feature_name=ENST00000280258 | feature_id[147].value <= threshold=75.14949798583984 |
| node_4: feature_name=ENST00000284311 | feature_id[369].value > threshold=100.91744613647461 |

| | |
|---|---|
| node_22: feature_name=ENST00000367467 | feature_id[367].value > threshold=35.77326202392578 |
| node_28: feature_name=ENST00000404989 | feature_id[45].value <= threshold=50.12705612182617 |
| node_29: feature_name=ENST00000573760 | feature_id[340].value <= threshold=8.433976173400879 |
| node_30: feature_name=ENST00000448155 | feature_id[75].value > threshold=34.63613796234131 |
| Class: current_smokers | |
| | |
| Rules_55 | passed counts:2 |
| node_0: feature_name=ENST00000284311 | feature_id[369].value <= threshold=179.44091796875 |
| node_1: feature_name=ENST00000390539 | feature_id[61].value <= threshold=11.91484260559082 |
| node_2: feature_name=ENST00000586582 | feature_id[365].value <= threshold=17.368224143981934 |
| node_3: feature_name=ENST00000280258 | feature_id[147].value <= threshold=75.14949798583984 |
| node_4: feature_name=ENST00000284311 | feature_id[369].value <= threshold=100.91744613647461 |
| node_5: feature_name=ENST00000492167 | feature_id[84].value <= threshold=164.934326171875 |
| node_6: feature_name=ENST00000329099 | feature_id[211].value > threshold=289.5108947753906 |
| node_8: feature_name=ENST00000491977 | feature_id[91].value > threshold=5.64292573928833 |
| node_18: feature_name=ENST00000226279 | feature_id[218].value > threshold=79.78742218017578 |
| Class: current_smokers | |
| | |
| Rules_56 | passed counts:2 |
| node_0: feature_name=ENST00000284311 | feature_id[369].value <= threshold=179.44091796875 |
| node_1: feature_name=ENST00000390539 | feature_id[61].value <= threshold=11.91484260559082 |
| node_2: feature_name=ENST00000586582 | feature_id[365].value <= threshold=17.368224143981934 |
| node_3: feature_name=ENST00000280258 | feature_id[147].value <= threshold=75.14949798583984 |
| node_4: feature_name=ENST00000284311 | feature_id[369].value <= threshold=100.91744613647461 |
| node_5: feature_name=ENST00000492167 | feature_id[84].value <= threshold=164.934326171875 |
| node_6: feature_name=ENST00000329099 | feature_id[211].value <= threshold=289.5108947753906 |

| | |
|---|---|
| Class: current_smokers | |
| | |
| Rules_57 | passed counts:1 |
| node_0: feature_name=ENST00000284311 | feature_id[369].value > threshold=179.44091796875 |
| node_98: feature_name=ENST00000396276 | feature_id[33].value > threshold=957.0642700195312 |
| node_148: feature_name=ENST00000284311 | feature_id[369].value > threshold=364.31639099121094 |
| node_168: feature_name=ENST00000339223 | feature_id[361].value > threshold=33.90394592285156 |
| node_174: feature_name=ENST00000513886 | feature_id[309].value <= threshold=141.72513961791992 |
| node_175: feature_name=ENST00000390305 | feature_id[88].value > threshold=495.3319091796875 |
| Class: former_smokers | |
| | |
| Rules_58 | passed counts:1 |
| node_0: feature_name=ENST00000284311 | feature_id[369].value > threshold=179.44091796875 |
| node_98: feature_name=ENST00000396276 | feature_id[33].value > threshold=957.0642700195312 |
| node_148: feature_name=ENST00000284311 | feature_id[369].value > threshold=364.31639099121094 |
| node_168: feature_name=ENST00000339223 | feature_id[361].value > threshold=33.90394592285156 |
| node_174: feature_name=ENST00000513886 | feature_id[309].value <= threshold=141.72513961791992 |
| node_175: feature_name=ENST00000390305 | feature_id[88].value <= threshold=495.3319091796875 |
| node_176: feature_name=ENST00000291576 | feature_id[206].value > threshold=301.24168395996094 |
| Class: former_smokers | |
| | |
| Rules_59 | passed counts:1 |
| node_0: feature_name=ENST00000284311 | feature_id[369].value > threshold=179.44091796875 |
| node_98: feature_name=ENST00000396276 | feature_id[33].value > threshold=957.0642700195312 |
| node_148: feature_name=ENST00000284311 | feature_id[369].value > threshold=364.31639099121094 |
| node_168: feature_name=ENST00000339223 | feature_id[361].value <= threshold=33.90394592285156 |

| | |
|---|---|
| node_169: feature_name=ENST00000297785 | feature_id[3].value > threshold=208.11764526367188 |
| node_171: feature_name=ENST00000393203 | feature_id[352].value <= threshold=4.1026811599731445 |
| Class: current_smokers | |
| | |
| Rules_60 | passed counts:1 |
| node_0: feature_name=ENST00000284311 | feature_id[369].value > threshold=179.44091796875 |
| node_98: feature_name=ENST00000396276 | feature_id[33].value > threshold=957.0642700195312 |
| node_148: feature_name=ENST00000284311 | feature_id[369].value <= threshold=364.31639099121094 |
| node_149: feature_name=ENST00000276974 | feature_id[251].value > threshold=1.20855313539505 |
| node_159: feature_name=ENST00000641136 | feature_id[58].value > threshold=5.980504512786865 |
| node_163: feature_name=ENST00000390624 | feature_id[71].value <= threshold=11.1699538230896 |
| node_164: feature_name=ENST00000492446 | feature_id[83].value <= threshold=0.6932542324066162 |
| Class: current_smokers | |
| | |
| Rules_61 | passed counts:1 |
| node_0: feature_name=ENST00000284311 | feature_id[369].value > threshold=179.44091796875 |
| node_98: feature_name=ENST00000396276 | feature_id[33].value > threshold=957.0642700195312 |
| node_148: feature_name=ENST00000284311 | feature_id[369].value <= threshold=364.31639099121094 |
| node_149: feature_name=ENST00000276974 | feature_id[251].value <= threshold=1.20855313539505 |
| node_150: feature_name=ENST00000420843 | feature_id[247].value > threshold=338.93580627441406 |
| node_152: feature_name=ENST00000643024 | feature_id[281].value > threshold=18.539386749267578 |
| node_154: feature_name=ENST00000367051 | feature_id[359].value > threshold=176.94873046875 |
| node_156: feature_name=ENST00000526097 | feature_id[294].value > threshold=40.1702938079834 |
| Class: former_smokers | |
| | |
| Rules_62 | passed counts:1 |

| | |
|---|---|
| node_0: feature_name=ENST00000284311 | feature_id[369].value > threshold=179.44091796875 |
| node_98: feature_name=ENST00000396276 | feature_id[33].value <= threshold=957.0642700195312 |
| node_99: feature_name=ENST00000316418 | feature_id[348].value > threshold=3.5686380863189697 |
| node_115: feature_name=ENST00000309575 | feature_id[233].value <= threshold=375.328125 |
| node_116: feature_name=ENST00000367467 | feature_id[367].value > threshold=21.34785747528076 |
| node_120: feature_name=ENST00000571489 | feature_id[244].value > threshold=56.20589256286621 |
| node_138: feature_name=ENST00000422622 | feature_id[357].value > threshold=9.033727169036865 |
| Class: current_smokers | |
| | |
| Rules_63 | passed counts:1 |
| node_0: feature_name=ENST00000284311 | feature_id[369].value > threshold=179.44091796875 |
| node_98: feature_name=ENST00000396276 | feature_id[33].value <= threshold=957.0642700195312 |
| node_99: feature_name=ENST00000316418 | feature_id[348].value > threshold=3.5686380863189697 |
| node_115: feature_name=ENST00000309575 | feature_id[233].value <= threshold=375.328125 |
| node_116: feature_name=ENST00000367467 | feature_id[367].value > threshold=21.34785747528076 |
| node_120: feature_name=ENST00000571489 | feature_id[244].value <= threshold=56.20589256286621 |
| node_121: feature_name=ENST00000635923 | feature_id[304].value <= threshold=328.1167755126953 |
| node_122: feature_name=ENST00000506927 | feature_id[329].value > threshold=24.940939903259277 |
| node_124: feature_name=ENST00000555619 | feature_id[254].value > threshold=105.99102401733398 |
| node_128: feature_name=ENST00000380672 | feature_id[170].value > threshold=33.95133399963379 |
| node_130: feature_name=ENST00000439754 | feature_id[183].value <= threshold=341.5655822753906 |
| node_131: feature_name=ENST00000637526 | feature_id[286].value <= threshold=120.73594665527344 |
| Class: current_smokers | |
| | |
| Rules_64 | passed counts:1 |
| node_0: feature_name=ENST00000284311 | feature_id[369].value > threshold=179.44091796875 |

| | |
|---|---|
| node_98: feature_name=ENST00000396276 | feature_id[33].value <= threshold=957.0642700195312 |
| node_99: feature_name=ENST00000316418 | feature_id[348].value > threshold=3.5686380863189697 |
| node_115: feature_name=ENST00000309575 | feature_id[233].value <= threshold=375.328125 |
| node_116: feature_name=ENST00000367467 | feature_id[367].value > threshold=21.34785747528076 |
| node_120: feature_name=ENST00000571489 | feature_id[244].value <= threshold=56.20589256286621 |
| node_121: feature_name=ENST00000635923 | feature_id[304].value <= threshold=328.1167755126953 |
| node_122: feature_name=ENST00000506927 | feature_id[329].value > threshold=24.940939903259277 |
| node_124: feature_name=ENST00000555619 | feature_id[254].value <= threshold=105.99102401733398 |
| node_125: feature_name=ENST00000635923 | feature_id[304].value > threshold=144.43426895141602 |
| Class: former_smokers | |
| | |
| Rules_65 | passed counts:1 |
| node_0: feature_name=ENST00000284311 | feature_id[369].value > threshold=179.44091796875 |
| node_98: feature_name=ENST00000396276 | feature_id[33].value <= threshold=957.0642700195312 |
| node_99: feature_name=ENST00000316418 | feature_id[348].value > threshold=3.5686380863189697 |
| node_115: feature_name=ENST00000309575 | feature_id[233].value <= threshold=375.328125 |
| node_116: feature_name=ENST00000367467 | feature_id[367].value > threshold=21.34785747528076 |
| node_120: feature_name=ENST00000571489 | feature_id[244].value <= threshold=56.20589256286621 |
| node_121: feature_name=ENST00000635923 | feature_id[304].value <= threshold=328.1167755126953 |
| node_122: feature_name=ENST00000506927 | feature_id[329].value > threshold=24.940939903259277 |
| node_124: feature_name=ENST00000555619 | feature_id[254].value <= threshold=105.99102401733398 |
| node_125: feature_name=ENST00000635923 | feature_id[304].value <= threshold=144.43426895141602 |
| Class: current_smokers | |
| | |
| Rules_66 | passed counts:1 |
| node_0: feature_name=ENST00000284311 | feature_id[369].value > threshold=179.44091796875 |

| | |
|---|---|
| node_98: feature_name=ENST00000396276 | feature_id[33].value <= threshold=957.0642700195312 |
| node_99: feature_name=ENST00000316418 | feature_id[348].value > threshold=3.5686380863189697 |
| node_115: feature_name=ENST00000309575 | feature_id[233].value <= threshold=375.328125 |
| node_116: feature_name=ENST00000367467 | feature_id[367].value > threshold=21.34785747528076 |
| node_120: feature_name=ENST00000571489 | feature_id[244].value <= threshold=56.20589256286621 |
| node_121: feature_name=ENST00000635923 | feature_id[304].value <= threshold=328.1167755126953 |
| node_122: feature_name=ENST00000506927 | feature_id[329].value <= threshold=24.940939903259277 |
| Class: former_smokers | |
| | |
| Rules_67 | passed counts:1 |
| node_0: feature_name=ENST00000284311 | feature_id[369].value > threshold=179.44091796875 |
| node_98: feature_name=ENST00000396276 | feature_id[33].value <= threshold=957.0642700195312 |
| node_99: feature_name=ENST00000316418 | feature_id[348].value <= threshold=3.5686380863189697 |
| node_100: feature_name=ENST00000312143 | feature_id[18].value <= threshold=80.9970703125 |
| node_101: feature_name=ENST00000610349 | feature_id[73].value > threshold=6.537376165390015 |
| node_103: feature_name=ENST00000423064 | feature_id[280].value <= threshold=22.101216316223145 |
| node_104: feature_name=ENST00000513886 | feature_id[309].value > threshold=133.05960845947266 |
| Class: former_smokers | |
| | |
| Rules_68 | passed counts:1 |
| node_0: feature_name=ENST00000284311 | feature_id[369].value <= threshold=179.44091796875 |
| node_1: feature_name=ENST00000390539 | feature_id[61].value > threshold=11.91484260559082 |
| node_83: feature_name=ENST00000565135 | feature_id[314].value > threshold=43.40609931945801 |
| node_87: feature_name=ENST00000544802 | feature_id[324].value > threshold=338.4650421142578 |
| node_89: feature_name=ENST00000600255 | feature_id[316].value > threshold=10.34920597076416 |
| node_95: feature_name=ENST00000390594 | feature_id[79].value <= threshold=2.376459836959839 |

| | |
|---|---|
| Class: current_smokers | |
| | |
| Rules_69 | passed counts:1 |
| node_0: feature_name=ENST00000284311 | feature_id[369].value <= threshold=179.44091796875 |
| node_1: feature_name=ENST00000390539 | feature_id[61].value > threshold=11.91484260559082 |
| node_83: feature_name=ENST00000565135 | feature_id[314].value > threshold=43.40609931945801 |
| node_87: feature_name=ENST00000544802 | feature_id[324].value > threshold=338.4650421142578 |
| node_89: feature_name=ENST00000600255 | feature_id[316].value <= threshold=10.34920597076416 |
| node_90: feature_name=ENST00000443723 | feature_id[35].value > threshold=3.926640510559082 |
| node_92: feature_name=ENST00000565135 | feature_id[314].value <= threshold=46.06411552429199 |
| Class: current_smokers | |
| | |
| Rules_70 | passed counts:1 |
| node_0: feature_name=ENST00000284311 | feature_id[369].value <= threshold=179.44091796875 |
| node_1: feature_name=ENST00000390539 | feature_id[61].value > threshold=11.91484260559082 |
| node_83: feature_name=ENST00000565135 | feature_id[314].value <= threshold=43.40609931945801 |
| node_84: feature_name=ENST00000330953 | feature_id[240].value > threshold=589.2798614501953 |
| Class: former_smokers | |
| | |
| Rules_71 | passed counts:1 |
| node_0: feature_name=ENST00000284311 | feature_id[369].value <= threshold=179.44091796875 |
| node_1: feature_name=ENST00000390539 | feature_id[61].value <= threshold=11.91484260559082 |
| node_2: feature_name=ENST00000586582 | feature_id[365].value <= threshold=17.368224143981934 |
| node_3: feature_name=ENST00000280258 | feature_id[147].value > threshold=75.14949798583984 |
| node_35: feature_name=ENST00000395002 | feature_id[2].value > threshold=25.20395278930664 |
| node_37: feature_name=ENST00000650242 | feature_id[185].value > threshold=1343.1289672851562 |

| | |
|---|---|
| node_41: feature_name=ENST00000290866 | feature_id[335].value <= threshold=12.690921306610107 |
| node_42: feature_name=ENST00000509697 | feature_id[272].value > threshold=2.719158351421356 |
| node_44: feature_name=ENST00000321016 | feature_id[364].value > threshold=35.50823783874512 |
| node_74: feature_name=ENST00000259089 | feature_id[293].value > threshold=63.914669036865234 |
| Class: former_smokers | |
| | |
| Rules_72 | passed counts:1 |
| node_0: feature_name=ENST00000284311 | feature_id[369].value <= threshold=179.44091796875 |
| node_1: feature_name=ENST00000390539 | feature_id[61].value <= threshold=11.91484260559082 |
| node_2: feature_name=ENST00000586582 | feature_id[365].value <= threshold=17.368224143981934 |
| node_3: feature_name=ENST00000280258 | feature_id[147].value > threshold=75.14949798583984 |
| node_35: feature_name=ENST00000395002 | feature_id[2].value > threshold=25.20395278930664 |
| node_37: feature_name=ENST00000650242 | feature_id[185].value > threshold=1343.1289672851562 |
| node_41: feature_name=ENST00000290866 | feature_id[335].value <= threshold=12.690921306610107 |
| node_42: feature_name=ENST00000509697 | feature_id[272].value > threshold=2.719158351421356 |
| node_44: feature_name=ENST00000321016 | feature_id[364].value > threshold=35.50823783874512 |
| node_74: feature_name=ENST00000259089 | feature_id[293].value <= threshold=63.914669036865234 |
| Class: current_smokers | |
| | |
| Rules_73 | passed counts:1 |
| node_0: feature_name=ENST00000284311 | feature_id[369].value <= threshold=179.44091796875 |
| node_1: feature_name=ENST00000390539 | feature_id[61].value <= threshold=11.91484260559082 |
| node_2: feature_name=ENST00000586582 | feature_id[365].value <= threshold=17.368224143981934 |
| node_3: feature_name=ENST00000280258 | feature_id[147].value > threshold=75.14949798583984 |
| node_35: feature_name=ENST00000395002 | feature_id[2].value > threshold=25.20395278930664 |
| node_37: feature_name=ENST00000650242 | feature_id[185].value > threshold=1343.1289672851562 |

| | |
|---|---|
| node_41: feature_name=ENST00000290866 | feature_id[335].value <= threshold=12.690921306610107 |
| node_42: feature_name=ENST00000509697 | feature_id[272].value > threshold=2.719158351421356 |
| node_44: feature_name=ENST00000321016 | feature_id[364].value <= threshold=35.50823783874512 |
| node_45: feature_name=ENST00000394718 | feature_id[110].value > threshold=15.798832416534424 |
| node_49: feature_name=ENST00000610495 | feature_id[298].value > threshold=8.27418327331543 |
| node_53: feature_name=ENST00000502981 | feature_id[292].value > threshold=2.636757969856262 |
| node_57: feature_name=ENST00000392054 | feature_id[368].value > threshold=134.09820556640625 |
| node_71: feature_name=ENST00000390305 | feature_id[88].value <= threshold=6.945567846298218 |
| Class: current_smokers | |
| | |
| Rules_74 | passed counts:1 |
| node_0: feature_name=ENST00000284311 | feature_id[369].value <= threshold=179.44091796875 |
| node_1: feature_name=ENST00000390539 | feature_id[61].value <= threshold=11.91484260559082 |
| node_2: feature_name=ENST00000586582 | feature_id[365].value <= threshold=17.368224143981934 |
| node_3: feature_name=ENST00000280258 | feature_id[147].value > threshold=75.14949798583984 |
| node_35: feature_name=ENST00000395002 | feature_id[2].value > threshold=25.20395278930664 |
| node_37: feature_name=ENST00000650242 | feature_id[185].value > threshold=1343.1289672851562 |
| node_41: feature_name=ENST00000290866 | feature_id[335].value <= threshold=12.690921306610107 |
| node_42: feature_name=ENST00000509697 | feature_id[272].value > threshold=2.719158351421356 |
| node_44: feature_name=ENST00000321016 | feature_id[364].value <= threshold=35.50823783874512 |
| node_45: feature_name=ENST00000394718 | feature_id[110].value > threshold=15.798832416534424 |
| node_49: feature_name=ENST00000610495 | feature_id[298].value > threshold=8.27418327331543 |
| node_53: feature_name=ENST00000502981 | feature_id[292].value > threshold=2.636757969856262 |
| node_57: feature_name=ENST00000392054 | feature_id[368].value <= threshold=134.09820556640625 |
| node_58: feature_name=ENST00000278919 | feature_id[149].value > threshold=45.06623077392578 |

| | |
|---|---|
| node_68: feature_name=ENST00000390306 | feature_id[97].value <= threshold=12.087755918502808 |
| Class: current_smokers | |
| | |
| Rules_75 | passed counts:1 |
| node_0: feature_name=ENST00000284311 | feature_id[369].value <= threshold=179.44091796875 |
| node_1: feature_name=ENST00000390539 | feature_id[61].value <= threshold=11.91484260559082 |
| node_2: feature_name=ENST00000586582 | feature_id[365].value <= threshold=17.368224143981934 |
| node_3: feature_name=ENST00000280258 | feature_id[147].value > threshold=75.14949798583984 |
| node_35: feature_name=ENST00000395002 | feature_id[2].value > threshold=25.20395278930664 |
| node_37: feature_name=ENST00000650242 | feature_id[185].value > threshold=1343.1289672851562 |
| node_41: feature_name=ENST00000290866 | feature_id[335].value <= threshold=12.690921306610107 |
| node_42: feature_name=ENST00000509697 | feature_id[272].value > threshold=2.719158351421356 |
| node_44: feature_name=ENST00000321016 | feature_id[364].value <= threshold=35.50823783874512 |
| node_45: feature_name=ENST00000394718 | feature_id[110].value > threshold=15.798832416534424 |
| node_49: feature_name=ENST00000610495 | feature_id[298].value > threshold=8.27418327331543 |
| node_53: feature_name=ENST00000502981 | feature_id[292].value > threshold=2.636757969856262 |
| node_57: feature_name=ENST00000392054 | feature_id[368].value <= threshold=134.09820556640625 |
| node_58: feature_name=ENST00000278919 | feature_id[149].value <= threshold=45.06623077392578 |
| node_59: feature_name=ENST00000610495 | feature_id[298].value > threshold=22.136420249938965 |
| node_63: feature_name=ENST00000393590 | feature_id[350].value > threshold=9.31120491027832 |
| node_65: feature_name=ENST00000548358 | feature_id[180].value <= threshold=16.836580276489258 |
| Class: current_smokers | |
| | |
| Rules_76 | passed counts:1 |
| node_0: feature_name=ENST00000284311 | feature_id[369].value <= threshold=179.44091796875 |
| node_1: feature_name=ENST00000390539 | feature_id[61].value <= threshold=11.91484260559082 |

| | |
|---|---|
| node_2: feature_name=ENST00000586582 | feature_id[365].value <= threshold=17.368224143981934 |
| node_3: feature_name=ENST00000280258 | feature_id[147].value > threshold=75.14949798583984 |
| node_35: feature_name=ENST00000395002 | feature_id[2].value > threshold=25.20395278930664 |
| node_37: feature_name=ENST00000650242 | feature_id[185].value > threshold=1343.1289672851562 |
| node_41: feature_name=ENST00000290866 | feature_id[335].value <= threshold=12.690921306610107 |
| node_42: feature_name=ENST00000509697 | feature_id[272].value > threshold=2.719158351421356 |
| node_44: feature_name=ENST00000321016 | feature_id[364].value <= threshold=35.50823783874512 |
| node_45: feature_name=ENST00000394718 | feature_id[110].value > threshold=15.798832416534424 |
| node_49: feature_name=ENST00000610495 | feature_id[298].value > threshold=8.27418327331543 |
| node_53: feature_name=ENST00000502981 | feature_id[292].value > threshold=2.636757969856262 |
| node_57: feature_name=ENST00000392054 | feature_id[368].value <= threshold=134.09820556640625 |
| node_58: feature_name=ENST00000278919 | feature_id[149].value <= threshold=45.06623077392578 |
| node_59: feature_name=ENST00000610495 | feature_id[298].value <= threshold=22.136420249938965 |
| node_60: feature_name=ENST00000380490 | feature_id[121].value > threshold=1292.0121765136719 |
| Class: current_smokers | |
| | |
| Rules_77 | passed counts:1 |
| node_0: feature_name=ENST00000284311 | feature_id[369].value <= threshold=179.44091796875 |
| node_1: feature_name=ENST00000390539 | feature_id[61].value <= threshold=11.91484260559082 |
| node_2: feature_name=ENST00000586582 | feature_id[365].value <= threshold=17.368224143981934 |
| node_3: feature_name=ENST00000280258 | feature_id[147].value > threshold=75.14949798583984 |
| node_35: feature_name=ENST00000395002 | feature_id[2].value > threshold=25.20395278930664 |
| node_37: feature_name=ENST00000650242 | feature_id[185].value > threshold=1343.1289672851562 |
| node_41: feature_name=ENST00000290866 | feature_id[335].value <= threshold=12.690921306610107 |
| node_42: feature_name=ENST00000509697 | feature_id[272].value > threshold=2.719158351421356 |

| | |
|---|---|
| node_44: feature_name=ENST00000321016 | feature_id[364].value <= threshold=35.50823783874512 |
| node_45: feature_name=ENST00000394718 | feature_id[110].value > threshold=15.798832416534424 |
| node_49: feature_name=ENST00000610495 | feature_id[298].value > threshold=8.27418327331543 |
| node_53: feature_name=ENST00000502981 | feature_id[292].value <= threshold=2.636757969856262 |
| node_54: feature_name=ENST00000340342 | feature_id[205].value > threshold=47.64341354370117 |
| Class: former_smokers | |
| | |
| Rules_78 | passed counts:1 |
| node_0: feature_name=ENST00000284311 | feature_id[369].value <= threshold=179.44091796875 |
| node_1: feature_name=ENST00000390539 | feature_id[61].value <= threshold=11.91484260559082 |
| node_2: feature_name=ENST00000586582 | feature_id[365].value <= threshold=17.368224143981934 |
| node_3: feature_name=ENST00000280258 | feature_id[147].value > threshold=75.14949798583984 |
| node_35: feature_name=ENST00000395002 | feature_id[2].value > threshold=25.20395278930664 |
| node_37: feature_name=ENST00000650242 | feature_id[185].value > threshold=1343.1289672851562 |
| node_41: feature_name=ENST00000290866 | feature_id[335].value <= threshold=12.690921306610107 |
| node_42: feature_name=ENST00000509697 | feature_id[272].value > threshold=2.719158351421356 |
| node_44: feature_name=ENST00000321016 | feature_id[364].value <= threshold=35.50823783874512 |
| node_45: feature_name=ENST00000394718 | feature_id[110].value > threshold=15.798832416534424 |
| node_49: feature_name=ENST00000610495 | feature_id[298].value > threshold=8.27418327331543 |
| node_53: feature_name=ENST00000502981 | feature_id[292].value <= threshold=2.636757969856262 |
| node_54: feature_name=ENST00000340342 | feature_id[205].value <= threshold=47.64341354370117 |
| Class: current_smokers | |
| | |
| Rules_79 | passed counts:1 |
| node_0: feature_name=ENST00000284311 | feature_id[369].value <= threshold=179.44091796875 |
| node_1: feature_name=ENST00000390539 | feature_id[61].value <= threshold=11.91484260559082 |

| | |
|---|---|
| node_2: feature_name=ENST00000586582 | feature_id[365].value <= threshold=17.368224143981934 |
| node_3: feature_name=ENST00000280258 | feature_id[147].value > threshold=75.14949798583984 |
| node_35: feature_name=ENST00000395002 | feature_id[2].value > threshold=25.20395278930664 |
| node_37: feature_name=ENST00000650242 | feature_id[185].value > threshold=1343.1289672851562 |
| node_41: feature_name=ENST00000290866 | feature_id[335].value <= threshold=12.690921306610107 |
| node_42: feature_name=ENST00000509697 | feature_id[272].value > threshold=2.719158351421356 |
| node_44: feature_name=ENST00000321016 | feature_id[364].value <= threshold=35.50823783874512 |
| node_45: feature_name=ENST00000394718 | feature_id[110].value > threshold=15.798832416534424 |
| node_49: feature_name=ENST00000610495 | feature_id[298].value <= threshold=8.27418327331543 |
| node_50: feature_name=ENST00000262139 | feature_id[132].value > threshold=56.27672004699707 |
| Class: current_smokers | |
| | |
| Rules_80 | passed counts:1 |
| node_0: feature_name=ENST00000284311 | feature_id[369].value <= threshold=179.44091796875 |
| node_1: feature_name=ENST00000390539 | feature_id[61].value <= threshold=11.91484260559082 |
| node_2: feature_name=ENST00000586582 | feature_id[365].value <= threshold=17.368224143981934 |
| node_3: feature_name=ENST00000280258 | feature_id[147].value > threshold=75.14949798583984 |
| node_35: feature_name=ENST00000395002 | feature_id[2].value > threshold=25.20395278930664 |
| node_37: feature_name=ENST00000650242 | feature_id[185].value > threshold=1343.1289672851562 |
| node_41: feature_name=ENST00000290866 | feature_id[335].value <= threshold=12.690921306610107 |
| node_42: feature_name=ENST00000509697 | feature_id[272].value > threshold=2.719158351421356 |
| node_44: feature_name=ENST00000321016 | feature_id[364].value <= threshold=35.50823783874512 |
| node_45: feature_name=ENST00000394718 | feature_id[110].value > threshold=15.798832416534424 |
| node_49: feature_name=ENST00000610495 | feature_id[298].value <= threshold=8.27418327331543 |
| node_50: feature_name=ENST00000262139 | feature_id[132].value <= threshold=56.27672004699707 |

| | |
|---|---|
| Class: former_smokers | |
| | |
| Rules_81 | passed counts:1 |
| node_0: feature_name=ENST00000284311 | feature_id[369].value <= threshold=179.44091796875 |
| node_1: feature_name=ENST00000390539 | feature_id[61].value <= threshold=11.91484260559082 |
| node_2: feature_name=ENST00000586582 | feature_id[365].value <= threshold=17.368224143981934 |
| node_3: feature_name=ENST00000280258 | feature_id[147].value > threshold=75.14949798583984 |
| node_35: feature_name=ENST00000395002 | feature_id[2].value > threshold=25.20395278930664 |
| node_37: feature_name=ENST00000650242 | feature_id[185].value > threshold=1343.1289672851562 |
| node_41: feature_name=ENST00000290866 | feature_id[335].value <= threshold=12.690921306610107 |
| node_42: feature_name=ENST00000509697 | feature_id[272].value > threshold=2.719158351421356 |
| node_44: feature_name=ENST00000321016 | feature_id[364].value <= threshold=35.50823783874512 |
| node_45: feature_name=ENST00000394718 | feature_id[110].value <= threshold=15.798832416534424 |
| node_46: feature_name=ENST00000555619 | feature_id[254].value > threshold=406.4545593261719 |
| Class: current_smokers | |
| | |
| Rules_82 | passed counts:1 |
| node_0: feature_name=ENST00000284311 | feature_id[369].value <= threshold=179.44091796875 |
| node_1: feature_name=ENST00000390539 | feature_id[61].value <= threshold=11.91484260559082 |
| node_2: feature_name=ENST00000586582 | feature_id[365].value <= threshold=17.368224143981934 |
| node_3: feature_name=ENST00000280258 | feature_id[147].value > threshold=75.14949798583984 |
| node_35: feature_name=ENST00000395002 | feature_id[2].value > threshold=25.20395278930664 |
| node_37: feature_name=ENST00000650242 | feature_id[185].value > threshold=1343.1289672851562 |
| node_41: feature_name=ENST00000290866 | feature_id[335].value <= threshold=12.690921306610107 |
| node_42: feature_name=ENST00000509697 | feature_id[272].value > threshold=2.719158351421356 |
| node_44: feature_name=ENST00000321016 | feature_id[364].value <= threshold=35.50823783874512 |

| | |
|---|---|
| node_45: feature_name=ENST00000394718 | feature_id[110].value <= threshold=15.798832416534424 |
| node_46: feature_name=ENST00000555619 | feature_id[254].value <= threshold=406.4545593261719 |
| Class: former_smokers | |
| | |
| Rules_83 | passed counts:1 |
| node_0: feature_name=ENST00000284311 | feature_id[369].value <= threshold=179.44091796875 |
| node_1: feature_name=ENST00000390539 | feature_id[61].value <= threshold=11.91484260559082 |
| node_2: feature_name=ENST00000586582 | feature_id[365].value <= threshold=17.368224143981934 |
| node_3: feature_name=ENST00000280258 | feature_id[147].value > threshold=75.14949798583984 |
| node_35: feature_name=ENST00000395002 | feature_id[2].value > threshold=25.20395278930664 |
| node_37: feature_name=ENST00000650242 | feature_id[185].value > threshold=1343.1289672851562 |
| node_41: feature_name=ENST00000290866 | feature_id[335].value <= threshold=12.690921306610107 |
| node_42: feature_name=ENST00000509697 | feature_id[272].value <= threshold=2.719158351421356 |
| Class: current_smokers | |
| | |
| Rules_84 | passed counts:1 |
| node_0: feature_name=ENST00000284311 | feature_id[369].value <= threshold=179.44091796875 |
| node_1: feature_name=ENST00000390539 | feature_id[61].value <= threshold=11.91484260559082 |
| node_2: feature_name=ENST00000586582 | feature_id[365].value <= threshold=17.368224143981934 |
| node_3: feature_name=ENST00000280258 | feature_id[147].value > threshold=75.14949798583984 |
| node_35: feature_name=ENST00000395002 | feature_id[2].value > threshold=25.20395278930664 |
| node_37: feature_name=ENST00000650242 | feature_id[185].value <= threshold=1343.1289672851562 |
| node_38: feature_name=ENST00000260526 | feature_id[139].value > threshold=10.508224964141846 |
| Class: former_smokers | |
| | |
| Rules_85 | passed counts:1 |

| | |
|---|---|
| node_0: feature_name=ENST00000284311 | feature_id[369].value <= threshold=179.44091796875 |
| node_1: feature_name=ENST00000390539 | feature_id[61].value <= threshold=11.91484260559082 |
| node_2: feature_name=ENST00000586582 | feature_id[365].value <= threshold=17.368224143981934 |
| node_3: feature_name=ENST00000280258 | feature_id[147].value <= threshold=75.14949798583984 |
| node_4: feature_name=ENST00000284311 | feature_id[369].value > threshold=100.91744613647461 |
| node_22: feature_name=ENST00000367467 | feature_id[367].value <= threshold=35.77326202392578 |
| node_23: feature_name=ENST00000503004 | feature_id[349].value > threshold=100.92071533203125 |
| node_25: feature_name=ENST00000396618 | feature_id[216].value > threshold=11.128355741500854 |
| Class: current_smokers | |
| | |
| Rules_86 | passed counts:1 |
| node_0: feature_name=ENST00000284311 | feature_id[369].value <= threshold=179.44091796875 |
| node_1: feature_name=ENST00000390539 | feature_id[61].value <= threshold=11.91484260559082 |
| node_2: feature_name=ENST00000586582 | feature_id[365].value <= threshold=17.368224143981934 |
| node_3: feature_name=ENST00000280258 | feature_id[147].value <= threshold=75.14949798583984 |
| node_4: feature_name=ENST00000284311 | feature_id[369].value <= threshold=100.91744613647461 |
| node_5: feature_name=ENST00000492167 | feature_id[84].value <= threshold=164.934326171875 |
| node_6: feature_name=ENST00000329099 | feature_id[211].value > threshold=289.5108947753906 |
| node_8: feature_name=ENST00000491977 | feature_id[91].value > threshold=5.64292573928833 |
| node_18: feature_name=ENST00000226279 | feature_id[218].value <= threshold=79.78742218017578 |
| Class: former_smokers | |
| | |
| Rules_87 | passed counts:1 |
| node_0: feature_name=ENST00000284311 | feature_id[369].value <= threshold=179.44091796875 |
| node_1: feature_name=ENST00000390539 | feature_id[61].value <= threshold=11.91484260559082 |
| node_2: feature_name=ENST00000586582 | feature_id[365].value <= threshold=17.368224143981934 |

| | |
|---|---|
| node_3: feature_name=ENST00000280258 | feature_id[147].value <= threshold=75.14949798583984 |
| node_4: feature_name=ENST00000284311 | feature_id[369].value <= threshold=100.91744613647461 |
| node_5: feature_name=ENST00000492167 | feature_id[84].value <= threshold=164.934326171875 |
| node_6: feature_name=ENST00000329099 | feature_id[211].value > threshold=289.5108947753906 |
| node_8: feature_name=ENST00000491977 | feature_id[91].value <= threshold=5.64292573928833 |
| node_9: feature_name=ENST00000483295 | feature_id[237].value > threshold=82.8672981262207 |
| node_11: feature_name=ENST00000276974 | feature_id[251].value > threshold=19.783329486846924 |
| Class: current_smokers | |
| | |
| Rules_88 | passed counts:1 |
| node_0: feature_name=ENST00000284311 | feature_id[369].value <= threshold=179.44091796875 |
| node_1: feature_name=ENST00000390539 | feature_id[61].value <= threshold=11.91484260559082 |
| node_2: feature_name=ENST00000586582 | feature_id[365].value <= threshold=17.368224143981934 |
| node_3: feature_name=ENST00000280258 | feature_id[147].value <= threshold=75.14949798583984 |
| node_4: feature_name=ENST00000284311 | feature_id[369].value <= threshold=100.91744613647461 |
| node_5: feature_name=ENST00000492167 | feature_id[84].value <= threshold=164.934326171875 |
| node_6: feature_name=ENST00000329099 | feature_id[211].value > threshold=289.5108947753906 |
| node_8: feature_name=ENST00000491977 | feature_id[91].value <= threshold=5.64292573928833 |
| node_9: feature_name=ENST00000483295 | feature_id[237].value > threshold=82.8672981262207 |
| node_11: feature_name=ENST00000276974 | feature_id[251].value <= threshold=19.783329486846924 |
| node_12: feature_name=ENST00000390256 | feature_id[69].value > threshold=4.867078065872192 |
| node_14: feature_name=ENST00000472064 | feature_id[203].value > threshold=44.19396781921387 |
| Class: current_smokers | |
| | |
| Rules_89 | passed counts:1 |
| node_0: feature_name=ENST00000284311 | feature_id[369].value <= threshold=179.44091796875 |

| | |
|---|---|
| node_1: feature_name=ENST00000390539 | feature_id[61].value <= threshold=11.91484260559082 |
| node_2: feature_name=ENST00000586582 | feature_id[365].value <= threshold=17.368224143981934 |
| node_3: feature_name=ENST00000280258 | feature_id[147].value <= threshold=75.14949798583984 |
| node_4: feature_name=ENST00000284311 | feature_id[369].value <= threshold=100.91744613647461 |
| node_5: feature_name=ENST00000492167 | feature_id[84].value <= threshold=164.934326171875 |
| node_6: feature_name=ENST00000329099 | feature_id[211].value > threshold=289.5108947753906 |
| node_8: feature_name=ENST00000491977 | feature_id[91].value <= threshold=5.64292573928833 |
| node_9: feature_name=ENST00000483295 | feature_id[237].value > threshold=82.8672981262207 |
| node_11: feature_name=ENST00000276974 | feature_id[251].value <= threshold=19.783329486846924 |
| node_12: feature_name=ENST00000390256 | feature_id[69].value > threshold=4.867078065872192 |
| node_14: feature_name=ENST00000472064 | feature_id[203].value <= threshold=44.19396781921387 |
| Class: former_smokers | |
| | |
| Rules_90 | passed counts:1 |
| node_0: feature_name=ENST00000284311 | feature_id[369].value <= threshold=179.44091796875 |
| node_1: feature_name=ENST00000390539 | feature_id[61].value <= threshold=11.91484260559082 |
| node_2: feature_name=ENST00000586582 | feature_id[365].value <= threshold=17.368224143981934 |
| node_3: feature_name=ENST00000280258 | feature_id[147].value <= threshold=75.14949798583984 |
| node_4: feature_name=ENST00000284311 | feature_id[369].value <= threshold=100.91744613647461 |
| node_5: feature_name=ENST00000492167 | feature_id[84].value <= threshold=164.934326171875 |
| node_6: feature_name=ENST00000329099 | feature_id[211].value > threshold=289.5108947753906 |
| node_8: feature_name=ENST00000491977 | feature_id[91].value <= threshold=5.64292573928833 |
| node_9: feature_name=ENST00000483295 | feature_id[237].value <= threshold=82.8672981262207 |
| Class: current_smokers | |
