# Supplementary material for: Identification of Smoking-Associated Transcriptome Aberration in Blood with Machine Learning Methods
Source: Biomed Res Int. 2023 Jan 4;2023:5333361. doi: 10.1155/2023/5333361 (PMC9833906; doi:10.1155/2023/5333361)
Supplement: Supplementary Materials — Table S1: feature ranking results obtained by mRMR, MCFS, LightGBM, and LASSO methods. Table S2: IFS results on different feature lists. Table S3: intersection of the optimal feature subsets extracted from mRMR, MCFS, LightGBM, and LASSO feature lists. The features that appear in 4, 3, 2, and 1 optimal feature subsets are shown. Table S4: classification rules generated by the optimal DT model. Table S5: GO and KEGG enrichment results after merging the optimal feature subsets of the four feature ranking algorithms. [file 5333361.f1.zip › Table S5 (1).pdf]

Table S5: GO and KEGG enrichment results after merging the optimal feature subsets of the four feature ranking algorithms.

(1) Gene ontology enrichment results

ONTOLOGY	ID	Description	GeneRatio	BgRatio	pvalue	p.adjust	qvalue
BP	GO:0006958	complement activation, classical pathway	41/112	141/18862	5.12E-60	8.32E-57	7.61E-57
BP	GO:0002455	humoral immune response mediated by circulating immunoglobulin	41/112	156/18862	6.08E-58	4.94E-55	4.52E-55
BP	GO:0006956	complement activation	41/112	178/18862	2.73E-55	1.48E-52	1.35E-52
BP	GO:0016064	immunoglobulin mediated immune response	43/112	223/18862	1.84E-54	7.48E-52	6.84E-52
BP	GO:0019724	B cell mediated immunity	43/112	226/18862	3.44E-54	1.12E-51	1.02E-51
BP	GO:0002449	lymphocyte mediated immunity	48/112	360/18862	5.22E-53	1.42E-50	1.29E-50
BP	GO:0002460	adaptive immune response based on somatic recombination of immune receptors built from immunoglobulin superfamily domains	47/112	367/18862	5.84E-51	1.36E-48	1.24E-48

BP	GO:0030449	regulation of complement activation	33/112	115/18862	9.26E-48	1.81E-45	1.66E-45
BP	GO:0002431	Fc receptor mediated stimulatory signaling pathway	35/112	145/18862	1.00E-47	1.81E-45	1.66E-45
BP	GO:0002920	regulation of humoral immune response	33/112	138/18862	8.76E-45	1.42E-42	1.30E-42
BP	GO:0002433	immune response-regulating cell surface receptor signaling pathway involved in phagocytosis	33/112	139/18862	1.14E-44	1.55E-42	1.42E-42
BP	GO:0038096	Fc-gamma receptor signaling pathway involved in phagocytosis	33/112	139/18862	1.14E-44	1.55E-42	1.42E-42
BP	GO:0038094	Fc-gamma receptor signaling pathway	33/112	140/18862	1.49E-44	1.86E-42	1.71E-42
BP	GO:0006959	humoral immune response	43/112	380/18862	6.02E-44	7.00E-42	6.40E-42
BP	GO:0006909	phagocytosis	42/112	381/18862	2.26E-42	2.45E-40	2.24E-40
BP	GO:0002429	immune response-activating cell surface receptor signaling pathway	44/112	481/18862	6.70E-41	6.42E-39	5.87E-39

BP	GO:0002757	immune response-activating signal transduction	44/112	481/18862	6.70E-41	6.42E-39	5.87E-39
BP	GO:0002377	immunoglobulin production	32/112	198/18862	1.81E-37	1.63E-35	1.49E-35
BP	GO:0002697	regulation of immune effector process	41/112	465/18862	2.82E-37	2.42E-35	2.21E-35
BP	GO:0038095	Fc-epsilon receptor signaling pathway	30/112	169/18862	2.13E-36	1.73E-34	1.59E-34
BP	GO:0038093	Fc receptor signaling pathway	33/112	239/18862	2.74E-36	2.12E-34	1.94E-34
BP	GO:0002440	production of molecular mediator of immune response	34/112	286/18862	3.98E-35	2.94E-33	2.69E-33
BP	GO:0006898	receptor-mediated endocytosis	32/112	328/18862	3.22E-30	2.28E-28	2.08E-28
BP	GO:0006910	phagocytosis, recognition	19/112	98/18862	5.97E-24	4.05E-22	3.70E-22
BP	GO:0050871	positive regulation of B cell activation	21/112	150/18862	2.98E-23	1.94E-21	1.77E-21
BP	GO:0050853	B cell receptor signaling pathway	20/112	134/18862	9.33E-23	5.84E-21	5.34E-21
BP	GO:0050864	regulation of B cell activation	22/112	193/18862	2.61E-22	1.57E-20	1.44E-20
BP	GO:0008037	cell recognition	23/112	232/18862	6.85E-22	3.98E-20	3.64E-20

BP	GO:0006911	phagocytosis, engulfment	19/112	126/18862	9.61E-22	5.39E-20	4.93E-20
BP	GO:0099024	plasma membrane invagination	19/112	135/18862	3.77E-21	2.05E-19	1.87E-19
BP	GO:0010324	membrane invagination	19/112	143/18862	1.17E-20	6.16E-19	5.63E-19
BP	GO:0042113	B cell activation	23/112	326/18862	1.55E-18	7.88E-17	7.21E-17
BP	GO:0051251	positive regulation of lymphocyte activation	23/112	356/18862	1.10E-17	5.40E-16	4.94E-16
BP	GO:0002696	positive regulation of leukocyte activation	24/112	401/18862	1.14E-17	5.43E-16	4.97E-16
BP	GO:0050867	positive regulation of cell activation	24/112	412/18862	2.11E-17	9.80E-16	8.96E-16
BP	GO:0042742	defense response to bacterium	22/112	344/18862	7.53E-17	3.40E-15	3.11E-15
BP	GO:0050851	antigen receptor-mediated signaling pathway	21/112	323/18862	2.94E-16	1.29E-14	1.18E-14
BP	GO:0003094	glomerular filtration	4/112	24/18862	1.14E-05	0.000489	0.0004473
BP	GO:0097205	renal filtration	4/112	25/18862	1.35E-05	0.0005646	0.0005165
BP	GO:0002507	tolerance induction	4/112	28/18862	2.16E-05	0.0008789	0.000804

BP	GO:0002707	negative regulation of lymphocyte mediated immunity	4/112	45/18862	0.0001455	0.005773	0.0052813
BP	GO:0002715	regulation of natural killer cell mediated immunity	4/112	48/18862	0.0001874	0.0072597	0.0066414
BP	GO:0002703	regulation of leukocyte mediated immunity	7/112	209/18862	0.0002514	0.0095128	0.0087026
BP	GO:0002704	negative regulation of leukocyte mediated immunity	4/112	53/18862	0.0002757	0.0101935	0.0093253
BP	GO:0002706	regulation of lymphocyte mediated immunity	6/112	157/18862	0.0003497	0.0126451	0.0115681
BP	GO:0007200	phospholipase C-activating G protein-coupled receptor signaling pathway	5/112	103/18862	0.000372	0.0130382	0.0119277
BP	GO:0002418	immune response to tumor cell	3/112	24/18862	0.0003766	0.0130382	0.0119277
BP	GO:0002228	natural killer cell mediated immunity	4/112	67/18862	0.000677	0.0229481	0.0209935
BP	GO:0003014	renal system process	5/112	118/18862	0.0006929	0.0230073	0.0210477

BP	GO:0002347	response to tumor cell	3/112	31/18862	0.0008116	0.0264082	0.0241589
BP	GO:0002698	negative regulation of immune effector process	5/112	125/18862	0.0008988	0.0286749	0.0262326
BP	GO:0001895	retina homeostasis	4/112	80/18862	0.0013169	0.0412028	0.0376934
BP	GO:0002291	T cell activation via T cell receptor contact with antigen bound to MHC molecule on antigen presenting cell	2/112	10/18862	0.0015244	0.0459283	0.0420164
BP	GO:0002725	negative regulation of T cell cytokine production	2/112	10/18862	0.0015244	0.0459283	0.0420164
BP	GO:0019730	antimicrobial humoral response	5/112	142/18862	0.001586	0.0469155	0.0429195
CC	GO:0019814	immunoglobulin complex	49/115	167/19520	1.19E-72	1.61E-70	1.56E-70
CC	GO:0042571	immunoglobulin complex, circulating	20/115	77/19520	5.01E-28	3.40E-26	3.29E-26
CC	GO:0072562	blood microparticle	19/115	146/19520	1.59E-20	7.21E-19	6.97E-19

CC	GO:0009897	external side of plasma membrane	23/115	402/19520	1.41E-16	4.81E-15	4.65E-15
MF	GO:0003823	antigen binding	38/100	165/18337	6.86E-53	1.21E-50	1.16E-50
MF	GO:0034987	immunoglobulin receptor binding	19/100	79/18337	1.11E-26	9.74E-25	9.38E-25

(2) KEGG enrichment results

ID	Description	GeneRatio	BgRatio	pvalue	p.adjust	qvalue
hsa05330	Allograft rejection	4/36	38/8112	2.17E-05	0.0021226	0.0018923
hsa05320	Autoimmune thyroid disease	4/36	53/8112	8.19E-05	0.0040154	0.0035798
hsa04650	Natural killer cell mediated cytotoxicity	5/36	131/8112	0.0002566	0.0064594	0.0057586
hsa04060	Cytokine-cytokine receptor interaction	7/36	295/8112	0.0002636	0.0064594	0.0057586
hsa05332	Graft-versus-host disease	3/36	42/8112	0.0008183	0.0132461	0.0118091
hsa04940	Type I diabetes mellitus	3/36	43/8112	0.000877	0.0132461	0.0118091
hsa04061	Viral protein interaction with cytokine and cytokine receptor	4/36	100/8112	0.0009461	0.0132461	0.0118091
hsa05416	Viral myocarditis	3/36	60/8112	0.0023092	0.0282881	0.0252192
hsa04080	Neuroactive ligand-receptor interaction	6/36	353/8112	0.0041878	0.0456003	0.0406533
